# Mechanistic Insights into Binding of Ligands with Thiazolidinedione Warhead to Human Histone Deacetylase 4

**DOI:** 10.3390/ph14101032

**Published:** 2021-10-11

**Authors:** Markus Schweipert, Niklas Jänsch, Neha Upadhyay, Kalpana Tilekar, Ewelina Wozny, Sidra Basheer, Eva Wurster, Marlene Müller, Ramaa C S, Franz-Josef Meyer-Almes

**Affiliations:** 1 Department of Chemical Engineering and Biotechnology, University of Applied Sciences, 64295 Darmstadt, Germany; markus.schweipert@h-da.de (M.S.); niklas.jaensch@h-da.de (N.J.); ewelina.wozny@stud.h-da.de (E.W.); sidra.basheer@stud.h-da.de (S.B.); eva.wurster@stud.h-da.de (E.W.); marlene.mueller@stud.h-da.de (M.M.); 2 Department of Pharmaceutical Chemistry, Bharati Vidyapeeth’s College of Pharmacy, Navi Mumbai 400614, India; upadhyayneha16@gmail.com (N.U.); kalpana.tilekar@gmail.com (K.T.)

**Keywords:** Human Histone Deacetylase 4 (HDAC4), thiazolidinediones, binding mechanism, mutational study

## Abstract

Recently, we have reported that non-hydroxamate thiazolidinedione (TZD) analogs are capable of inhibiting human deacetylase 4 (HDAC4). This study aims at the dissection of the molecular determinants and kinetics of the molecular recognition of TZD ligands by HDAC4. For this purpose, a structure activity relationship analysis of 225 analogs was combined with a comprehensive study of the enzyme and binding kinetics of a variety of HDAC4 mutant variants. The experimental data were rationalized by docking to the two major conformations of HDAC4. TZD ligands are competitive inhibitors and bind via a two-step mechanism involving principal molecular recognition and induced fit. The residence time of 24 g is (34 ± 3) min and thus much larger than that of the canonical pan-HDAC inhibitor SAHA ((5 ± 2) min). Importantly, the binding kinetics can be tuned by varying the structure of the CAP group.

## 1. Introduction

HDAC4 is a class IIa zinc-dependent histone deacetylase (HDAC) which is highly expressed in the brain, heart, and skeletal muscle and plays a major role in tissue growth and physiological development [1]. With a length of 972 to 1084 amino acids and a molecular weight of ca. 120 kDa, it is one of the biggest HDACs across all four HDAC classes [2]. For this study, the catalytic domain of HDAC4 (cdHDAC4) was used, which consists of 410 amino acids (human HDAC4 T648-T1057) and has a molecular weight of 44.2 kDa. In vivo as well as in vitro HDAC4 shows an exceptionally low to nonexistent deacetylation activity towards natural acetylated substrates due to tyrosine to histidine mutation located in the active site. Therefore, enzymatic activity is not the primary biological function of HDAC4. Like all members of class IIa HDACs, HDAC4 has a highly flexible structural zinc binding domain (sZBD) with a second zinc atom in addition to the catalytic zinc in the enzyme’s active site. Because of the sZBD HDAC4 can adopt two distinct conformations with different types of inhibitors (open and closed) in x-ray crystal structures [3,4]. In the open conformation the sZBD is flipped out of the globular protein structure and therefore far away from HDAC4’s catalytic site. For the closed conformation, this is not the case [3]. HDAC4 can shuttle between nucleus and cytoplasm, which is its primary biological function [5]. With the help of nuclear receptor co-repressor (NCoR) as well as silencing mediator for retinoid or thyroid-hormone receptors (SMRT) HDAC4 can shuttle HDAC3 between nucleus and cytoplasm and therefore plays a key role in the distribution of enzymatically active HDAC3 in mammal cells. For this process, the sZBD serves as a scaffold to bind the SMRT/NCoR protein complex, which subsequently binds HDAC3. The sZBD is essential for the recognition of the SMRT/NCoR protein complex and can only bind in its closed conformation, which is believed to be the biologically relevant conformation [6]. Several nephrological and neurodegenerative diseases, [7,8] as well as cancer types like breast cancer are related to HDAC4 making the protein an attractive drug target [1,2,8,9]. Furthermore, studies showed that inhibition of HDAC4 activity in animal models can reduce symptoms of Huntington’s disease, which may be a potential treatment for this yet incurable disease [10,11]. Most of present HDAC inhibitors contain a hydroxamic acid as zinc binding group and are more or less unselective inhibitors of all zinc-dependent HDACs. This applies also to HDAC inhibitory drugs like Vorinostat [12], Belinostat [13], and Panobinostat [14], which are approved for the treatment of cutaneous T-cell lymphoma. Nowadays, hydroxamic acids are not only considered as a source of unselectivity but also under suspicion for their mutagenic potential [15]. Therefore, alternative zinc binding groups are highly desired. 1,3-Thiazolidine-2,4-dione (TZD) containing compounds, also known as glitazones, were originally developed by Takeda Pharmaceutical in Japan as drugs for the treatment of type 2 diabetes mellitus. TZD ligands act via activation of the gamma type of peroxisome proliferator-activated receptors (PPARγ) in the nucleus [16,17]. Furthermore, some TZD ligands are capable to inhibit aldose reductase (ALR2), protein tyrosine phosphatase 1B (PTP1B) and α-glucosidase [18]. Very recently, we reported on TZD-containing ligands, which are capable to inhibit HDAC4 [18,19]. Enzyme activity assays of the HDAC family demonstrated activity of TZD ligands against HDAC4 or HDAC8 depending on the substitution pattern. Furthermore, some TZD containing compounds exhibited also activity towards other protein families such as the glucose transporters GLUT1, GLUT4 and GLUT5 [20,21]. Importantly, some of the dual targeting TZD ligands show in vivo effects by drastically lowering the viability of K562 chronic myeloid leukaemia cell lines resulting in rapid cell death as well as anti-tumor effects in tumor xenograft models [18]. Although the activity of TZD ligands towards HDAC4 has been described very recently, their mode of action is still uncharted [18]. This study focuses on the elucidation of the detailed mechanism of interaction between TZD ligands and HDAC4. An extensive structure activity relationship (SAR) analysis was carried out to dissect the structural elements, which are important for the potent and selective inhibition of HDAC4 by TZD ligands. The binding mode was analyzed by Michaelis Menten kinetics [22]. Combined with a comprehensive mutational study we were able to assess the impact of particular amino acids on substrate affinity and binding constant of TZD ligands. The binding kinetics of selected TZD analogs were measured to determine the binding mechanism and important kinetic constants like the residence time of the compounds on the HDAC4 target. Finally, docking was applied to rationalize the experimental binding data and predict binding poses of TZD ligands.

## 2. Results and Discussion

### 2.1. Chemistry

#### Synthesis of Compounds PB1–PB9 and GB1–GB36

**Scheme 1:**
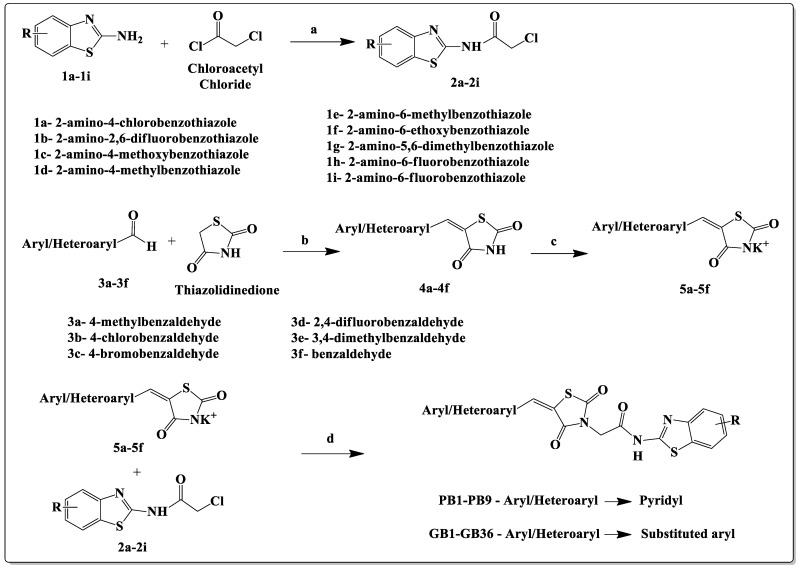
Synthesis of compounds (PB1–PB9 and GB1–GB36). Reagents and conditions: (a) K_2_CO_3_, DCM, Stir at RT for 24 h. (b) Acetic acid, Sodium acetate, reflux 3–6 h; (c) EtOH, KOH, reflux 1–2 h. (d) Acetone, reflux 6–10 h.

The chloracetylated amide (**2a**–**2i**) intermediates were synthesized by condensing differently substituted benzothiazoleamines (**1a**–**1i**) with chloroacetylchloride by procedure previously reported elsewhere (Scheme 1) [23,24]. To brief, chloroacetyl chloride was dropwise added to a chilled solution of substituted amines (**1a**–**1i**) and potassium carbonate in dichloromethane (DCM) solvent, and allowed to stir overnight. The crude was collected by evaporating the solvent under vacuum and recrystallized with ethanol. This choloracetylated intermediate was common for the synthesis of both sets of compounds. All the Knoevenagel intermediates (**4a**–**4f**) were obtained by procedure previously reported [23,24]. Thiazolidine-2,4-dione (5 gm, 0.04 moles) was refluxed for 3–6 h by intermittent stirring with pyridine-2-carboxaldehyde (5 gm, 0.07 moles) (for PB set) in the presence of sodium acetate (3 gm, 0.003 moles), and acetic acid (10 mL). The reaction mixture was allowed to cool and crystalline crude obtained was collected by vacuum filter and washed with water and air dried to obtain the respective pyridyl Knoevenagel intermediate (**4**). This intermediate was used for the synthesis of PB set of compounds.

Potassium hydroxide (2.5 gm, 0.044 moles) in ethanol was added to the pyridyl Knoevenagel intermediate (**4**) (5.0 gm, 0.025 moles) in a flat bottom flask and this mixture was refluxed with stirring for 3–4 h. After cooling the reaction mixture, crude salt was obtained by filtering under vacuum pump, washed with cold ethanol, and air dried to obtain intermediate **5**. Final compounds (**PB1**–**PB9**) were obtained by refluxing the two intermediates in equimolar ratio, **2a**–**2i** (0.044 moles) and **5** (0.044 moles) in acetone for 6–8 h. The reaction was monitored for completion by TLC using hexane: ethylacetate mobile phase. The reaction mixture was poured into crushed ice and precipitated solid was filtered under vacuum and this residue was purified by column chromatography by using ethyl acetate: hexane mobile phase in the ratio of 10:90 to 40:60.

For the synthesis of GB set of compounds, thiazolidine-2,4-dione (5 gm, 0.04 moles) was refluxed for 3–6 h with intermittent stirring with differently substituted aldehydes (**3a**–**3f**, Scheme 1) (5 gm, 0.07 moles) in the presence of sodium acetate (3 gm, 0.003 moles), and acetic acid (10 mL). Upon cooling, a crystalline crude was obtained which was collected by vacuum filter and washed with water, and air dried to obtain the respective Knoevenagel intermediates (**4a**–**4f**). The potassium salts were prepared as per previously reported procedure [23,24]. Potassium hydroxide (2.5 gm, 0.044 moles) in ethanol was added to Knoevenagel intermediates (**4a**–**4f**) (5.0 gm, 0.025 moles) in a flat bottom flask and this mixture was refluxed with stirring for 3–4 h. After cooling, the crude salts were obtained by filtering under vacuum pump, washed with cold ethanol, and air dried to obtain **5a**–**5f**. Final compounds (**GB1**–**GB36**) were obtained by refluxing the two intermediates in equimolar ratio, **2a**–**2i** (0.044 moles) and **5a**–**5f** (0.044 moles) in acetone for 6–8 h. The reaction was monitored for completion by TLC using hexane: ethylacetate mobile phase. The reaction mixture was poured into crushed ice and precipitated solid was filtered under vacuum and this residue was purified by column chromatography by using ethyl acetate: hexane mobile phase in the ration of 10:90 to 40:60.

### 2.2. Elucidation of the Mechanism of Action of TZD Ligands

#### 2.2.1. An Elongated Ligand Structure with Terminal TZD Group Is Crucial for HDAC4 Activity

**Figure 1:**
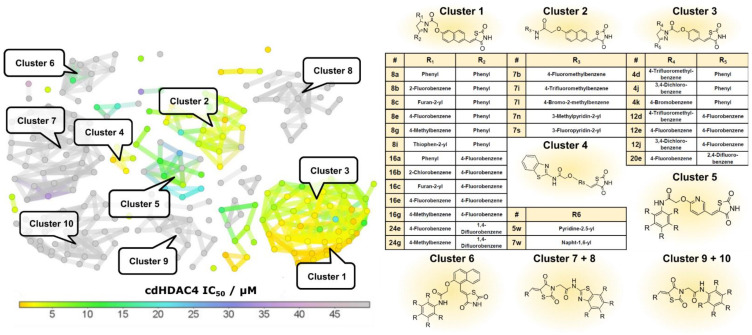
Similarity map for structural activity relationship analysis for 225 TZD containing compounds. The similarity map revealed ten clusters with varying inhibitory potencies towards cdHDAC4_wt_. Marker color and connecting lines highlight inhibitory activity and similarity pairs, respectively. Filled gray circles represent inactive TZD ligands with IC_50_-values > 50 µM.

This study evaluated 223 TZD ligand analogs including newly synthesized compounds PB1-PB9 and GB1-GB36 with different substitution patterns (Table S1). Many of the TZD ligands have been published very recently to be HDAC4 or HDAC8 inhibitors [18,19,25]. This large ensemble of TZD ligands was utilized to derive a SAR and identify ligand moieties crucial for binding to the catalytic domain of human wild type histone deacetylase 4 (cdHDAC4_wt_). To gain a thorough understanding of the binding the enzymatic activity of cdHDAC4_wt_ was tested in the presence of TZD ligands with different types of linkers between the TZD moiety and the CAP group and different CAP groups with varying substitution patterns. The linkers have different attachment points that determine the overall elongated or kinked structure of the ligands. 97 out of 223 TZD ligands exhibited IC_50_-values under 50 µM against cdHDAC4_wt_. This data was used for SAR analysis. By utilizing the DataWarrior program (www.openmolecules.org, accessed on 8 March 2021) and its integrated similarity analysis algorithm, a similarity map of all tested TZD ligands was created that produced ten clusters of structurally similar compounds (Figure 1). Clusters 1–4 included the most potent TZD ligands with IC_50_-values below 2 µM. The members of these clusters differed in CAP group and linker type that connected CAP group and TZD moiety. CAP groups of potent TZD ligands consisted of dihydropyrazole that was decorated either by two differently substituted phenyl rings, furan or thiophene (cluster 1 and 3), single differently substituted phenyl or pyridine rings (cluster 2) or benzothiazoles (cluster 4). Different substitutions at those CAP groups as well as different linker types (e.g., naphthalene, phenyl, pyridine), further tuned individual TZD ligand affinity towards cdHDAC4_wt_ within the clusters. TZD ligand **8b** in cluster 1 showed the lowest IC_50_-value of 330 nM (Table 1). The common feature of all potent clusters was a terminal TZD moiety in an elongated overall structure. Cluster 5 contained TZD ligands with moderate activities, which were similar to the compounds in cluster 4. The benzothiazole moiety in cluster 5 compounds was replaced with differently substituted phenyl moieties in cluster 4 analogs. Essentially inactive inhibitors in cluster 6 demonstrated the importance of linker arrangement. The only difference between cluster 6 and cluster 2 was a 1,2- versus 2,6-connection of the naphthyl linker, respectively. Nearly all non-potent TZD ligands contained a TZD moiety in the molecule’s center indicating that a central sterically hindered TZD moiety was not able to bind to cdHDAC4_wt_ (Cluster 7–10) (Figure 1). In these analogs, the TZD moiety served as a linker between different moieties such as benzothiazoles, differently substituted phenyls, and pyridines. The most active TZD ligands in clusters 1–4 were all elongated compounds with terminal sterically unhindered TZD group and selected to elucidate the binding mode and mechanism to cdHDAC4_wt_. 

#### 2.2.2. TZD Ligands Are Competitive Inhibitors

**Figure 2:**
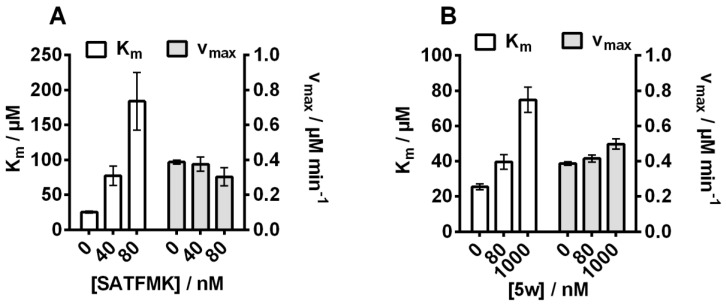
Michaelis Menten parameters for the determination of the mode of action with increasing compound concentration. K_m_- and V_max_-values of (**A**) SATFMK and (**B**) TZD ligand **5w** showed increasing and stagnating values, respectively, as expected for a competitive inhibitor. Shown data represent means and standard deviations, *N* = 3.

Encouraged by promising cdHDAC4_wt_ activity of the TZD ligands despite the absence of a canonical zinc binding group the mode of action was analyzed by applying Michaelis Menten kinetics to determine, whether TZD ligands are competitive inhibitors and thus bind at the active site, or otherwise bind at an allosteric site of the enzyme. 9,9,9-Trifluoro-8-oxo-N-phenyl-nonanamide (SATFMK), a trifluoromethylketone analog of SAHA (Vorinostat) and known reversible and competitive inhibitor, was used as control. Higher concentrations of both, SATFMK and the representative TZD ligand **5w**, produced increasing K_m_ values but showed essentially unchanged maximum enzyme velocity, v_max_ (Figure 2). Therefore, TZD ligand **5w** binds as a competitive inhibitor within the active site of cdHDAC4_wt_. The complete set of Michaelis Menten data is available in the supplementary information (Figure S1).

#### 2.2.3. Binding Kinetics and Mechanism of TZD Ligands Depend on the CAP-Group

**Figure 3:**
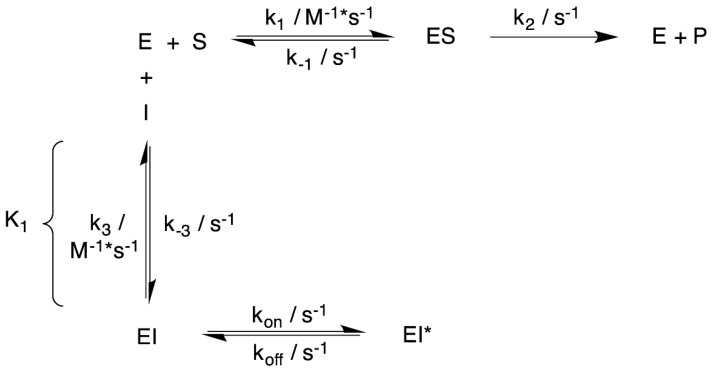
Induced fit binding model based on Michaelis Menten enzyme kinetics. Enzyme (E) and Substrate (S) are forming an enzyme-substrate complex (ES) in a reversible manner. Subsequently the substrate is irreversibly converted into the product (P), releasing free enzyme in the process. During an induced fit binding mode the inhibitor (I) and the enzyme form an initial enzyme-inhibitor complex (EI) under rapid equilibrium conditions, followed by an enzyme isomerization resulting in a tight binding enzyme-inhibitor complex (EI*). Note that k_2_ as well as the isomerization rates (k_on_ and k_off_) are not dose dependent.

**Figure 4:**
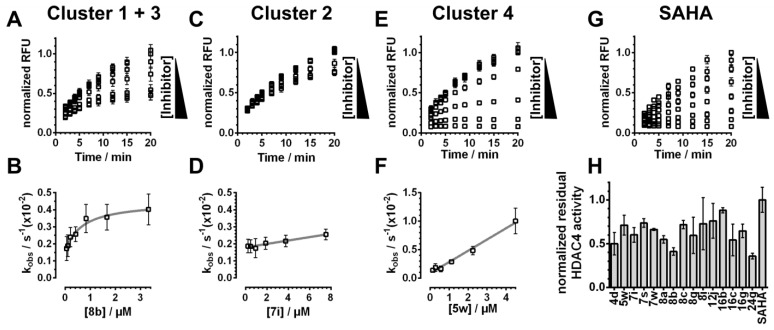
Association kinetics of HDAC4 and TZD ligands from different structural clusters**.** The association kinetics correlates with the chemical structure of TZD ligands and is substantially different between compound clusters 1–4. (**A**–**F**) Cluster 1 + 3, cluster 2 and cluster 4 are represented by TZD ligands **8b**, **7i** and **5w**, respectively. (**G**) Association kinetics of reference compound SAHA. (**H**) Residual cdHDAC4_wt_ activity 15 min after rapid dilution of all 15 tested TZD ligands and SAHA. Shown data represent means and standard deviations, *N* = 3.

Binding kinetics of potential drugs are crucial for in vivo activity [26]. Therefore association kinetic measurements based on Michaelis Menten enzyme kinetics (Figure 3) were performed for selected compounds including 15 representative TZD ligands (**4d**, **5w**, **7i**, **7s**, **7w**, **8a**, **8b**, **8c**, **8g**, **8i**, **12j**, **16b**, **16c**, **16g**, and **24g**) from structural clusters 1–4 having IC_50_-values under 2 µM towards cdHDAC4_wt_. The kinetic progression curves revealed a slow association behavior of all tested compounds (Table 1 and Figure 4A,C,E). The association kinetics did not reveal significant differences between clusters 1 and 3 (**4d**, **8a**, **8b**, **8c**, **8g**, **8i**, **12j**, **16b**, **16c**, **16g,** and **24g**). The data were analyzed by plotting the rate, k_obs_, by which the enzyme velocity is slowed down, versus inhibitor concentration [27]. All compounds from clusters 1 and 3 showed a saturating behavior of k_obs_ with increasing ligand concentrations (Figure 4B and Figure S2). The saturating curve progression of the fitted rate values indicated a two-step mechanism, in which the first step was significantly faster than the second step. Although the tested TZD ligands of clusters 1 and 3 exhibited a certain degree of structural diversity, all compounds display similar plateaus of rate constants, suggesting that the rate was limited by conformational changes of the initially formed protein-ligand complex. Consequently, the rate constants were fitted to an induced fit kinetic model, which considers the formation of an initial encounter complex of compound and enzyme under rapid equilibrium conditions followed by a slower rate-limiting subsequent isomerization of the enzyme (Figure 3) [27]. The details about the application of the fitting equations for the association as well as dissociation rate calculations are described in the methods section. 

The equilibrium constant, K_1_, for the initial encounter complex were determined for TZD ligands **8b**, **8i**, **24g**, **8g**, **8a**, **4d** and ranged from 0.65 µM (**8b**) to 3 µM (**8g**) (Table 1). However, because of the rapid equilibrium condition and comparably low temporal resolution of the applied manual semi continuous kinetic assay, K_1_-values for TZD ligands **8c**, **16b**, **16c**, **16g** and **12j** could not be determined due to large error values. For these compounds, a faster automated kinetic assay (e.g., stopped flow) may be more useful for resolving the initial association step. Furthermore, k_on_ rate constants were determined for all TZD ligands in cluster 1 and 3. Said compounds exhibited similar k_on_-values between (2.1 ± 0.9) × 10^−3^ s^−1^ (**8c**) and (5.4 ± 0.5) × 10^−3^ s^−1^ (**8i**) (Table 1). Only TZD ligand **4d** showed a moderately deviating k_on_-value of (8 ± 1) × 10^−3^ s^−1^. Within clusters 1 and 3 differently substituted CAP groups did not show a significant influence on k_on_-values. However, larger variations of the CAP group had a strong influence on the binding behavior of TZD ligands. By plotting the gained k_obs_-values versus the respective TZD ligand concentration, one could clearly distinguish between clusters 1/3 and clusters 2 or cluster 4 (Figure 4 and Figure S2). The already mentioned saturating behavior of plotted k_obs_-values was only found with clusters 1 and 3, which contained TZD ligands with dihydropyrazole that was substituted by two phenyl rings, differently substituted phenyl rings, furan or thiophene as CAP group. By plotting k_obs_-values against the corresponding ligand concentration the *y*-axis intercept marked the dissociation rate (k_off_). Here, the tested TZD ligands had values between 5.4 × 10^−4^ s^−1^ and 1.8 × 10^−3^ s^−1^ resulting in residence times (residence time = 1/k_off_) between (9 ± 3) min (**12j**) and (31 ± 17) min (**24g**) (Table 1). Unfortunately, the residence times of TZD ligands **16g** and **16c** could not be determined with this method due to large error values. The uncertainty in the determination of the *y*-axis intercept was attributed to errors at low TZD ligand concentration. The reciprocal transformation of k_off_-values to residence times further enhanced these errors. Therefore, additional reversibility tests were carried out via rapid dilution providing residual cdHDAC4_wt_ activities between 36% (**24g**) and 88% (**16b**) 15 min after rapid dilution of fully inhibited enzyme-inhibitor complexes (Figure 4H). Under the assumption of first order dissociation behavior, the corresponding residence times were calculated to be between 7 min and 34 min. With this method, the residence times of TZD ligands **16g** and **16c** were determined to be (15 ± 2) min and (21 ± 7) min, respectively (Table 1). Within the margin of error, the long residence times calculated by association kinetic measurements were in good agreement with the data obtained from rapid dilution experiments (Table 1). The TZD ligands of cluster 1 and 3 showed a significantly different kinetic behavior compared to reference compound SAHA and TZD ligands of clusters 2 and 4. Members of the latter clusters did not show a saturating behavior of k_obs_ plotted versus the respective TZD ligand concentration and, therefore, the rate constants were not fitted to the equation considering enzyme isomerization (Figure 4D,F and Figure S2). Linear regression of plotted k_obs_-values of TZD ligands **7i** and **7s** of cluster 2 calculated k_3_-values from slopes of (100 ± 30) M^−1^s^−1^ and (210 ± 40) M^−1^s^−1^ and y-intercepts of (1.8 × 10^−3^) s^−1^ and (1.6 × 10^−3^) s^−1^. Residence times of (9.3 ± 0.5) min and (10 ± 1) min were calculated from the corresponding k_−3_ rates equal to the *y*-axis intercept, respectively.

The small slope of k_obs_ for cluster 2 TZD ligands indicated slow binding behavior, which was confirmed by the association progress curves and did not reach plateaus within the assay’s 20 min timeframe even at 10-fold IC_50_ concentration (Figure 4C,D and Figure S2). Cluster 1 and 3 as well as cluster 4 did not show such slow binding behavior. In rapid dilution reversibility experiments TZD ligands **7i** and **7s** of cluster 2 revealed residence times of (17 ± 3) min and (11 ± 1) min, respectively. In the case of TZD ligand **7s** the residence times calculated from association kinetics and reversibility experiments were in agreement (Table 1). For TZD ligand **7i** the confidence intervals of residence times obtained from both methods did not overlap, but, with values of (9.3 ± 0.5) min and (17 ± 3) min, was in the same order of magnitude. TZD ligands **5w** and **7w** of cluster 4 exhibited also linear behavior of k_obs_ vs. ligand concentration with a slope (k_3_) of (1900 ± 100) M^−1^s^−1^ and (180 ± 30) M^−1^s^−1^ and y-intercepts of (8 ± 3) × 10^−4^ s^−1^ and (2.0 ± 0.2) × 10^−3^ s^−1^, resulting in residence times of (19 ± 7) min and (8.4 ± 0.8) min, respectively (Figure 4F and Figure S2).

**Table 1:** Kinetic parameters of TZD ligand binding to cdHDAC4_wt_.

TZD Ligand	Cluster No.	IC_50_/µM	K_1_/µM	k_on_/s (×10^−2^)	k_off_/s (×10^−2^)	k_3_/M^−1^ s^−1^	k_−3_/s^−1^ (×10^−2^)	RT (KF)/min	RT (RD)/min
**8b**	1	0.33 ± 0.02	0.65 ± 0.47	0.30 ± 0.06	0.15 ± 0.04	-	-	11 ± 3	29 ± 3
**8i**	1	0.34 ± 0.04	0.65 ± 0.31	0.54 ± 0.05	0.12 ± 0.03	-	-	14 ± 4	13 ± 8
**16b**	1	0.42 ± 0.02	n.d. *	0.24 ± 0.11	0.17 ± 0.03	-	-	9.8 ± 1.7	7 ± 1
**16g**	1	0.46 ± 0.06	n.d. *	0.51 ± 0.11	n.d. *	-	-	n.d. *	15 ± 2
**24g**	1	0.71 ± 0.04	1.3 ± 0.7	0.47 ± 0.06	0.05 ± 0.03	-	-	31 ± 17	34 ± 3
**8g**	1	0.76 ± 0.08	3 ± 2	0.38 ± 0.07	0.16 ± 0.03	-	-	10 ± 2	18 ± 7
**8a**	1	0.77 ± 0.09	2.4 ± 1.8	0.49 ± 0.14	0.15 ± 0.06	-	-	11 ± 4	19 ± 2
**7s**	2	0.78 ± 0.08	n.d. *	-	-	210 ± 40	0.16 ± 0.02	10 ± 1	11 ± 1
**4d**	3	0.79 ± 0.04	1.7 ± 0.7	0.8 ± 0.1	0.06 ± 0.03	-	-	26 ± 11	23 ± 6
**12j**	3	0.83 ± 0.02	n.d. *	0.31 ± 0.07	0.18 ± 0.05	-	-	9 ± 3	11 ± 5
**5w**	4	0.90 ± 0.08	n.d. *	-	-	1900 ± 100	0.08 ± 0.03	19 ± 7	12 ± 3
**8c**	1	1.2 ± 0.1	n.d. *	0.21 ± 0.09	0.14 ± 0.07	-	-	12 ± 6	12 ± 1
**7i**	2	1.3 ± 0.1	n.d. *	-	-	100 ± 30	0.18 ± 0.01	9.3 ± 0.5	17 ± 3
**16c**	1	1.4 ± 0.2	n.d. *	0.35 ± 0.18	n.d. *	-	-	n.d. *	21 ± 7
**7w**	4	1.6 ± 0.2	n.d. *	-	-	180 ± 30	0.20 ± 0.02	8.4 ± 0.8	13.9 ± 0.3
**SAHA**	-	40 ± 2	n.d. *	n.d. *	n.d. *	n.d. *	n.d. *	n.d. *	5 ± 2

Equilibrium and rate constants refer to the reaction scheme in Figure 3. RD = rapid dilution, RT = residence time, KF = kinetic fit of one step (k_−3_^−1^) and two step (k_off_^−1^) model. Shown data represent means and standard deviations, *N* = 3.

Compared with cluster 2 TZD ligands of cluster 4 showed slightly faster association rates, and equilibrium was reached within the assay’s timeframe. In rapid dilution experiments said TZD ligands had very similar residence times of (12 ± 3) min and (13.9 ± 0.3) min for TZD ligand **5w** and **7w**, respectively. These values were essentially in agreement with residence times calculated from association kinetics (Table 1). TZD ligands of clusters 2 and 4 (i.e., benzothiazoles and different substituted phenyl and pyridine rings as CAP groups) like SAHA exhibited linear dependency of k_obs_ from ligand concentration, consistent with a single binding step mechanism lacking the second rate limiting step (e.g., enzyme isomerization). The presence of bulkier dihydropyrazole CAP groups (i.e., CAP groups of cluster pair 1 and 3) seemed to be responsible for the observed saturation of k_obs_ at high ligand concentration. It should be noted that a saturation of k_obs_-values may also have occurred at drastically higher concentrations of TZD ligands in clusters 2 and 4, which could not be tested in experiment due to limited solubility of the compounds. Altogether, the binding mechanism of TZD ligands was clearly dependent on the CAP group, but not so much on the linker structure, whether it was a bulky naphthyl, or a smaller phenyl or pyrimidyl group. This become particularly obvious, when comparing the association kinetics of TZD-ligands from cluster 1 and cluster 2, both with the same naphthyl linker, but different CAP groups. A similar observation could be made by comparing cluster 3 and cluster 4 members also with the same linkers, but different CAP groups. In conclusion, TZD ligands with branched dihydropyrazole CAP-group that were substituted by two aromatic rings showed a more complex 2-step binding mechanism involving induced conformational changes of the protein, while less bulky CAP groups correlated with one-step binding. Moreover, association kinetics was strikingly slow, which was associated with prolonged residence times in the order of 10–30 min. The four compounds with the slowest dissociation behavior and residence times > 20 min all contained a branched dihydropyrazole CAP group. A long residence time is potentially beneficial for a drug candidate because it can act longer on its target and is not so quickly washed out of cells. 

#### 2.2.4. Site Directed Mutagenesis Uncovers Hotspots of TZD Ligand-HDAC4 Interaction

**Figure 5:**
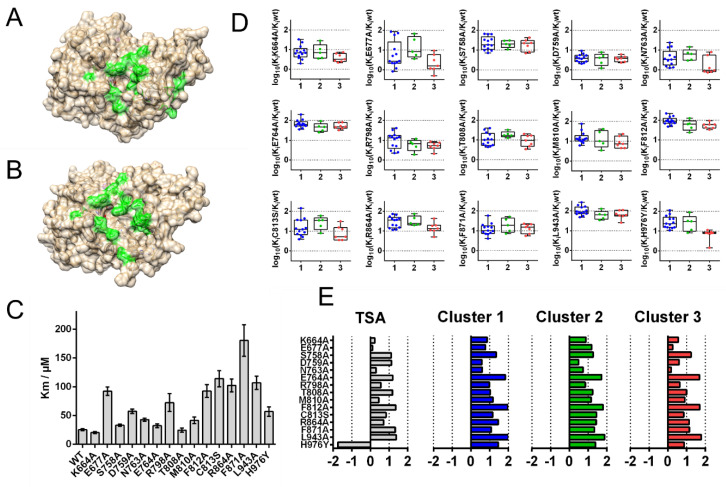
Overview of the mutational study. (**A**,**B**) are showing the substituted amino acid residues in green for the open (**A**) and closed (**B**) conformation. (**C**) K_m_ values for the artificial substrate Boc-Lys{TFA}-AMC. (**D**) Box-Whisker-Plot showing the impact of amino acid substitution on the binding of TZD-ligands on cdHDAC4_wt_. (**E**) Average impact of amino acid substitution for the three main TZD cluster in comparison to Trichostatin A (TSA).

To gain an thorough knowledge of the most involved amino acids necessary for molecular recognition of TZD ligands to cdHDAC4_wt_, a comprehensive mutational study was designed. Amino acids flanking the active site binding pocket in the *closed* (Figure 5A) and *open* (Figure 5B) conformation of the enzyme were systematically exchanged and IC_50_-values of a subset of 25 TZDs were determined against every mutant variant. Because IC_50_-values depends on the substrate concentration and the K_m_ of the substrate, the Michaelis Menten parameters were determined first using substrate tert-butyl *N*-[(2S)-1-[(4-methyl-2-oxochromen-7-yl)amino]-1-oxo-6-[(2,2,2-trifluoroacetyl)amino]hexan-2-yl]carbamate (Boc-Lys{TFA}-AMC) in a semi continuous enzyme activity assay (Figure 5C and Figure S1). IC_50_-values were transformed into binding constant K_i_ using the Cheng-Prusoff equation to enable a fair comparison of ligand affinities to the respective HDAC4 variant [28]:

(1) $${\mathrm{IC}}_{50}=\left(1+\frac{\left[S\right]}{K_{m}}\right)\times K_{i}$$

The application of the classic Cheng-Prusoff equation was justified, as TZD ligands were shown to be competitive inhibitors (see above). K_m_ values correlate with substrate affinity to the corresponding enzyme. Consequently, the exchange of amino acids that are important for substrate recognition should have an effect on the K_m_ value. The exchange of amino acids, which were far away from the active site (K644, S758, N763, E764, T808, M810) to alanine, had no notable effect on K_m_. 

In contrast, amino acids E677, D759, R798, F812, C813, R864, L943 and H976Y showed a high impact on substrate binding, which is sound because these amino acids flank the surface of the active site. The biggest impact was shown for F871, which is a highly conserved amino acid and essential component of the hydrophobic tunnel that accommodates and interacts with the alkyl chain of the acetylated lysine side chain of a substrate, when bound to the enzyme. Our results were in full agreement with previous knowledge about the molecular recognition of substrates by HDACs and, therefore, provided a good basis for the following analysis of the impact of amino acid exchanges on the binding affinity of TZD ligands to cdHDAC4_wt_. Figure 5D shows the log_10_ values for K_i_ of the mutant enzyme divided by the wildtype’s K_i_ for the main TZD ligand clusters 1, 2 and 3. This log_10_K_i_-ratio allowed for a precise examination of differences in binding upon amino acid substitution. A value of 0 implies that there is no change, a value between 0 and 1 implies a medium decrease of activity, whilst a value near 2 corresponds to a nearly complete loss of inhibition. Contrarily, a negative value corresponds to enhanced binding of the ligand to the mutant variant with respect to cdHDAC4_wt_. Most amino acid exchanges produced a medium to strong loss of affinity with similar effects for TZD ligands from different structural clusters (1–4) (Figure 5D). Comparing averaged log_10_K_i_ ratios of clusters 1–3 and the well-known pan-inhibitor Trichostatin A (TSA), revealed different patterns for the impact of mutations on the recognition of TZD ligands (Figure 5E). The main determinants for TSA binding were amino acids S758, D759, E764, T808, F812, F871 and L943 with a log_10_K_i_ ratio above 1. In contrast, the exchange of H976 to Y resulted in pronounced stronger binding of the inhibitor to the mutant variant cdHDAC4_H976Y_, which was in line with previous publications showing that hydroxamic acid inhibitors bind better to the gain-of-function mutant of HDAC4 [3]. In general, the TZD ligands showed more overall impact of amino acid substitution, which coincided with the generally higher isoenzyme selectivity of TZD ligands compared with pan-inhibitor TSA. Looking closer at the three main TZD ligand cluster revealed only minor differences between the three clusters. There were some subtle differences in the molecular recognition of clusters 2 and 3, particularly for E677A, N763A, C813S, R864A and H976Y, while no significant differences could be observed between clusters 1 and 2 (Figure 5D,E). Looking at the structural differences between cluster 1/2 and 3 compounds, TZD ligands in cluster 3 contained a phenyl linker, while TZD ligands in clusters 1 and 2 had a naphthyl linker. The greatest difference in binding affinity loss between cluster 1/2 and 3 was observed for the H976Y mutant variant of cdHDAC4 (Figure 5D,E) with highest impact on the recognition of cluster 1/2 compounds. Therefore, H976 was more important for the recognition of TZD ligands from clusters 1 and 2 than for members of cluster 3. Taken together, in contrast to TSA nearly each exchanged amino acid had a notable influence on the binding of TZD ligands to cdHDAC4. Most of the amino acid substitutions showed a similar effect on binding of all TZD ligands, but there were some interesting subtle differences between TZD ligand clusters, which suggested preferred molecular recognition of TZD ligands containing naphthyl linker over analog compounds with phenyl linker. The fact that so many amino acids had an impact on TZD ligand binding verified the high inhibitory activity and suggested a sound reason for the observed good isoenzyme selectivity of the investigated TZD ligands. All determined IC_50_-values of TZD ligands towards cdHDAC4_wt_ and mutants are summarized in the Supporting Information (Table S2).

#### 2.2.5. Docking and Mutational Analysis Predicts Binding to the Closed Conformation of HDAC4

**Figure 6:**
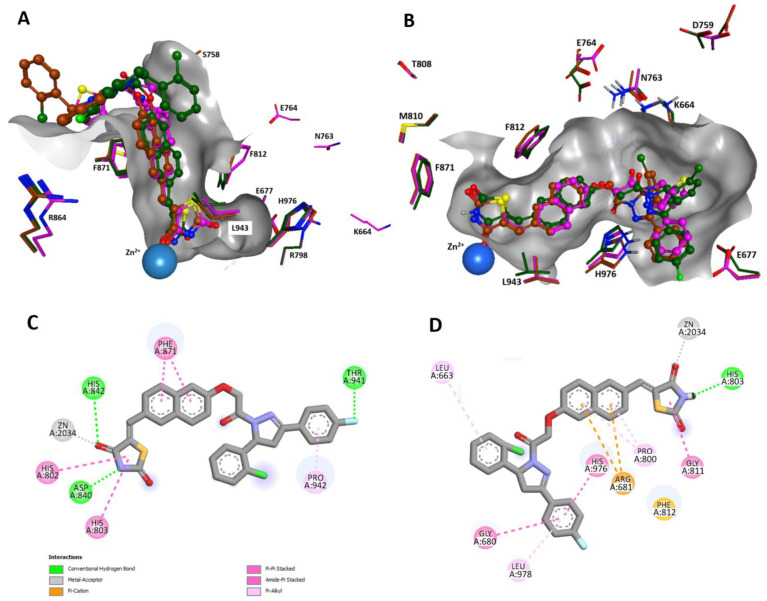
Docking poses of (S)-16b (dark green), (S)-8i (magenta) and (S)-8b (brown) within the binding pockets of (**A**) HDAC4_c_ (PDB-ID:4CBY) and (**B**) HDAC4_o_ (PDB-ID:2VQJ). The surface of the binding sites is colored in gray and clipped in the z-plane. The mutated amino acid residues are colored according to the corresponding docked ligand. The two lower panels show 2D ligand interactions of representative compound (S)-16b docked to (**C**) HDAC4_c_ and (**D**) HDAC4_o_.

The mutational analysis revealed characteristic influences of mutating selected amino acids surrounding the binding pockets of the *open* and *closed* conformation of cdHDAC4_wt_ on binding of TZD ligands. To rationalize these effects and gain more insight into key molecular determinants of molecular recognition, a set of TZD ligands was docked into the crystal structures of HDAC4_c_ (*closed*, PDB-ID:4CBY) and HDAC4_o_ (*open*, PDB-ID:2VQJ). The docking procedure was validated by redocking of the co-crystallized ligands into the respective crystal structure using AMBER 14 forcefield and London dG and GBVI/WSA dG scores implemented in MOE software, and allowing for induced fit around the binding pocket. The docked and crystallized poses of the ligand within the binding pocket of HDAC4_c_ (PDB-ID:4CBY) showed excellent overlap for the phenyl-cyclopropylhydroxamate moiety buried in the binding pocket with an RMSD-value of 0.267 Å and an acceptable overall RMSD-value 0.744 Å (Figure S3A). The trifluoromethyl warhead, which coordinates to the catalytic zinc ion, and thiophene linker of the redocked ligand in HDAC4_o_ (PDB ID:2VQJ) showed a very good RMSD value of 0.4 Å with respect to the ligand in the crystal structure (Figure S3B). Since the aromatic head group of the trifluoromethyl ketone ligand protrudes into free solution, this part of the molecules is intrinsically flexible and thus not considered for the calculation of RMSD. The largest structural movements during the transition from the *closed* to the *open* conformation of the catalytic domain of cdHDAC4_wt_ occur in two regions within the N-terminus of the catalytic domain. The first region between T660 and R681 forms the structural zinc binding domain (ZBD)-loop in the crystal structure of HDAC4_o_ (PDB-ID:2VQJ), and the second region between N726 and S767 defines the ZBD-helix of the zinc binding domain (Figure S4). The dramatic outward shift of ZBD-loop and ZBD-helix by about 15–20 Å, and additional minor conformational changes such as turn of the aromatic ring of F812 and some movement of H976 open the sidewall of the canonical binding pocket in the *closed* conformation to form the enlarged binding groove of HDAC4_o_ (Figure 6 and Figure S5). 

A key amino acid in the ZBD-helix is E764, which forms a salt bridge with R730. The transition from HDAC4_c_ to HDAC4_o_ involves disruption of this salt bridge and a 8.3 Å shift of the C_β_-atom of E764 (Figures S5 and S6). The effects of an exchange of this amino acid against alanine on the molecular recognition of the TZD ligands are discussed below. Clustering of most active 28 TZD analogs using compound similarity analysis revealed three clearly separated main cluster, a small cluster consisting of **5w** and **7w** carrying a characteristic benzothiazole moiety as head group, and the singleton **14d** with pyrimidine linker (Figure S7). Three representative TZD-analogs were docked for each of the three main clusters in order to identify contacts with surface amino acids and dissect differences between these clusters. Remarkably, docking results provided consistently better binding scores for the docking poses within HDAC4_o_ (PDB-ID: 2VQJ) compared with HDAC4_c_ (Table 2). Clusters 1 and 3 contained TZD-analogs have a dihydropyrazole CAP group with a stereo center at the heterocycle. Since the absolute configuration was not known, both enantiomers were docked into the respective crystal structures of HDAC4_c_ and HDAC4_o_. In all cases, docking scores for HDAC4_o_ were consistently more favorable than for HDAC4_c_. Therefore, TZD-analogs were suggested to bind tighter to the enlarged groove in the *open* conformation of HDAC4_o_, which offered more opportunities to interact with the surface than the *closed* conformation. Also, docking results suggested, that the TZD-enantiomers with the same absolute configuration as (S)-**16b** bind stronger or equal to both, HDAC4_c_ or HDAC4_o_. In general, the docking poses showed excellent overlap in the lower part of the binding pocket, where the TZD group coordinated to the catalytic zinc ion of both, *open* and *closed* conformation of cdHDAC4_wt_, through a carbonyl oxygen (Figure 6). There was also considerable structural overlap of the aromatic linker moieties among the compounds of a particular cluster docked to HDAC4_o_ or HDAC4_c_ (Figure 6 and Figure S8).

However, the location of the CAP group in HDAC4_c_ was less defined due to the widened upper region of the binding pocket and alternative interactions with the enzyme surface (Figure 6A and Figure S8A–C). In contrast, the binding poses of TZD analogs docked into HDAC4_o_ showed much better overlap for all ligand structures (Figure 6B and Figure S8D–F). The binding groove of the *open* conformation, HDAC4_o_, revealed an additional subpocket away from the catalytic zinc ion, which was occupied by an aromatic substituent of the dihydropyrazole CAP group of compounds in clusters 1 and 3 or the aromatic carboxamide CAP group of cluster 2 compounds (Figure 6B and Figure S10D–F). 

**Figure 7:**
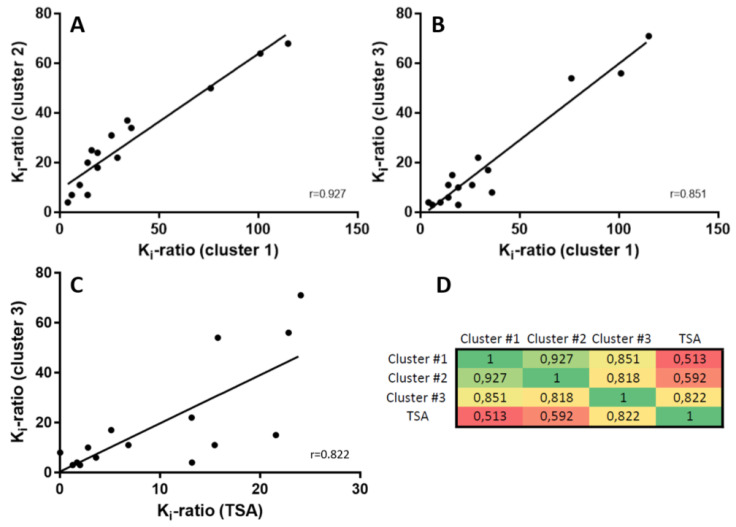
Binding profiles of TZD ligand clusters and TSA to HDAC4. Each data point represents a HDAC4-mutant, where one of the amino acids K664, E677, S758, D759, N763, E764, R798, T808, M810, F812, C813, R864, F871 or L943 is exchanged against alanine. The ratio of K_i_-values for binding to a mutant variant and cdHDAC4_wt_ indicates the impact of this specific mutation on the molecular recognition of a particular ligand. (**A**) Averaged K_i_-ratios for binding of TZD ligand cluster 2 and (**B**) TZD ligand cluster 3 are plotted versus the averaged K_i_-ratios of TZD ligand cluster 1. (**C**) The averaged K_i_-ratios of TZD ligand cluster 3 is plotted versus the K_i_-ratio of reference compound TSA. (**D**) Heat map of Spearman correlation coefficients for K_i_-ratios of indicated clusters and TSA.

To gain more insight into the binding mode of TZD ligands, affinity changes upon the mutation of selected amino acids and the contacts between ligands and HDAC4 in corresponding docking poses were analyzed and correlated. Looking at the effects of the exchange of single amino acids on binding affinity of TZD ligands in terms of K_i_-ratio revealed similarities, but also differences between the three TZD ligand clusters (Figure 7). The binding profiles of clusters 1 and 2, which contained a naphthalene linker, showed very similar impacts of amino acid exchanges on binding affinity with a Spearman correlation coefficient of 0.927 (Figure 7). The K_i_-ratios of TZD ligand clusters 2 and 3 were clearly less correlated (Spearman correlation coefficient = 0.818), which corresponded to a different linker and CAP group. Moreover, the binding profiles of cluster 3 and cluster 1 showed intermediate similarity, which corresponded to similar head groups but different linker (Spearman correlation coefficient = 0.851). Overall, these data suggested pronounced similarities in the molecular recognition of TZD ligands by cdHDAC4_wt_ with some differences between clusters. This finding was confirmed by docking, which predicted the TZD-group as zinc chelating warhead and additional hydrophobic interactions between the aromatic linker and surface amino acids of the canonical binding pocket in HDAC4_c_ or the wide open binding groove in HDAC4_o_ as common feature of TZD ligand binding. However, there were also distinct differences in the K_i_-ratio profiles between the TZD ligand clusters, which correlated mainly with the linker structure but also, to a minor extent, with the CAP group. 

The specific effect of exchanging amino acids at the expected interaction surfaces on the binding affinity of ligands was supposed to be a mixture of direct effects on specific interactions with the ligand and indirect effects on the conformation of the receptor protein, which vice versa affect binding in a more general way. The exchange of one of the three amino acids, E764, F812 and L943 produced drastic losses in affinity for all three TZD ligand clusters. E764 is a special case, because this amino acid is far away from the binding sites of HDAC4_c_, as well as HDAC4_o_, making a direct protein-ligand interaction unlikely.

**Figure 8:**
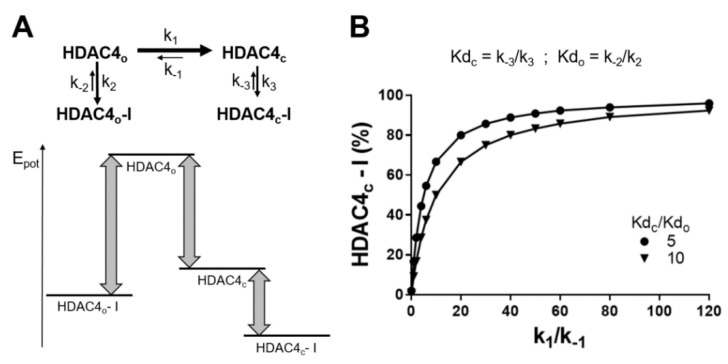
Energy diagram and simplified binding mechanism of *open* and *closed* form of cdHDAC4_wt_. (**A**) The upper panel shows a simplified binding mechanism of a ligand, I, to the *open* (HDAC4_o_) and *closed* (HDAC4_c_) conformation of HDAC4 yielding the corresponding reversible complexes HDAC4_o_-I and HDAC4_c_-I. The binding equilibria are coupled to the conformational equilibrium between HDAC4_o_ and HDAC4_c_. The k-parameters denote the respective reaction rate. The lower panel shows the corresponding energy diagram, which is consistent with the conformational equilibrium shifted toward HDAC4c and larger binding energy for the HDAC4_o_-I complex than for HDAC4_c_-I. (**B**) Simulation of percentage HDAC4_c_-I as a function of the ratio of HDAC4_c_ to HDAC4_o_ in terms of k_1_/k_−1_. Two cases are simulated: Ligand I has 5-times (filled circle) or 10-times (down-triangle) higher affinity for HDAC4_o_ than for HDAC4_c_.

The same observation occurred with binding of TSA to cdHDAC4_wt_. There is also a pronounced drop in affinity upon E764A exchange, although there is a very large distance (>20 Å) between TSA and the glutamate in the crystal structure of TSA in complex with HDAC7 that corresponds to E764. Therefore, the loss in affinity upon E764A exchange was essentially independent of the bound ligand and must be a secondary structural effect, which coincides with the disruption of a salt bridge to R730 and a considerable shift of this amino acid upon transition from the *closed* to the *open* conformation of HDAC4. This let us hypothesize, that the salt bridge between E764 and R730 stabilizes the *closed* conformation. If the salt bridge cannot be formed, the conformational equilibrium would be shifted from the *closed* to the *open* or other conformations and, as a general effect, weaken binding to the *closed* conformation. Exchanging amino acids K664, N763 and R798 by alanine produced only minor changes in the binding of TZD ligand in all three clusters. This was in agreement with docking poses in the binding pocket of the *closed* conformation HDAC4_c_, where these amino acids were more than 12 Å away from any docked TZD ligand. Moreover, the experimental binding results were in contrast to the observed close proximity between K664, N763 and R798 and TZD ligands docked into HDAC4_o_. TSA binding was also not impacted by the mutation of K664, N763 or R798. And a crystal structure of the complex between TSA and HDAC7 (PDB-ID:3C10) shows that TSA binds to the *closed* conformation of HDAC7. Since the binding pockets of HDAC4 and HDAC7 consist of identical amino acids and are structurally highly conserved, TSA was supposed to bind to HDAC4 in the same manner. These findings strongly suggested that the TZD ligands like TSA bind to the *closed* conformation of HDAC4. Docking poses of TZD ligands also indicated pi-pi-stacking between the aromatic naphtyl-linker and F812 for clusters 1 and 2 and aromatic/aliphatic interactions between the phenyl-linker of cluster 3 members and F812, when docked into HDAC4_c_ (Figure 6A and Figure S8A–C). Since there was no considerable shift of F812 upon transition from the *closed* to the *open* conformation of HDAC4, these interactions would also be possible in HDAC4_o_. L943 formed hydrophobic interactions with cluster 1 ligands that were docked into the *closed* conformation of HDAC4, but was not in proximity to members of cluster 1, when docked into HDAC4_o_. For cluster 2 and 3, docking results were consistent with both conformations of HDAC4. When F871 is exchanged against alanine, the binding affinity decreased only moderately by about 15–25 fold. Looking at the docking poses of TZD ligands revealed that the ligands form favorable pi-pi-stacking interactions between the aromatic linker and F871, when docked into HDAC4_c_, which was not the case for HDAC4_o_ (Figure 6 and Figure S8). This was also an argument for binding of TZD ligands to the *closed* rather than the *open* conformation of cdHDAC4_wt_. The gain-of -function mutant HDAC4_H976Y_ was a very interesting case, since Y976 is flipped-inward (PDB-ID:2VQW) and increased enzyme activity, because this residue is involved in the enzyme mechanism [3]. In HDAC4_wt_ the corresponding H976 is turned outward and opens a lower selectivity pocket, which was already exploited to develop selective inhibitors against class IIa HDACs [29]. Bottomley et al. showed, that the hydroxamic acid analog of a thiophen inhibitor is about 30-fold more active against HDAC4_H976Y_ compared to HDAC4_wt_, while the same inhibitor with a trifluoromethylketone warhead has similar activity against both variants of HDAC4. The crystal structure of the hydroxamate inhibitor with HDAC4_H976Y_ (PDB-ID:2VQV) reveals a hydrogen bond between Y976 and the carbonyl oxygen of the hydroxamate group as the most probable cause for the observed increased affinity. For TSA, an increase in affinity was observed upon H976Y exchange, in line with the results from Bottomley et al. In contrast, TZD ligands showed lower affinity to cdHDAC4_H976Y_ compared to cdHDAC4_wt_. A possible explanation for this experimental finding is that the flipped-in tyrosine residue may cause sterical hindrance and require conformational rearrangement of the binding pocket to recognize TZD ligands. Combining all binding data from the mutational analysis and molecular docking provided convincing evidence, that TZD ligands share common features of molecular recognition, but still can be grouped in three clusters with slightly different recognition patterns to specific amino acids of cdHDAC4_wt_. Docking suggested the TZD-group as alternative warhead to commonly used hydroxamate or trifluoromethylketone groups. Although docking scores for TZD ligand/HDAC4 complexes were consistently better for HDAC4_o_, the correlation of experimental affinity data and docking poses within HDAC4_c_ and HDAC4_o_ provided strong evidence that the TZD ligands bind preferentially to the *closed* form of HDAC4. How can the discrepancy between beneficial docking scores for HDAC4_o_, but good agreement between experimental binding data from the mutational study and docking poses within HDAC4_c_ be resolved? First of all, it must be considered that proteins in solution are highly flexible and usually exist in a chemical equilibrium between one, two or more major conformational states. Crystal structures reveal only snapshots of possible protein conformations, there is no guarantee that the crystallized conformation would be the dominant one in aqueous solution. Therefore, one has to consider both, the conformational equilibrium between the *open* and *closed* conformation of the catalytic domain of HDAC4, as well as the binding equilibrium of TZD ligands to both protein conformations (Figure 8A). If we assume that a ligand binds much tighter to HDAC4_o_ than to HDAC4_c_ as suggested by docking, this would predominantly lead to TZD ligand/HDAC4_o_ complexes, if similar concentrations of both conformations are present. However, if the equilibrium between HDAC4_o_ and HDAC4_c_ is shifted towards the *closed* conformation, this has also consequences for the ratio of TZD ligands bound to HDAC4_o_ or HDAC4_c_. The energy diagram in Figure 8A demonstrates, that a strong shift of unbound HDAC4_o_ towards HDAC4_c_ can produce a higher proportion of the complex of the ligand with HDAC4_c_, HDAC4_c_-I, although the ligand binds with much higher affinity to HDAC4_o_. This effect was simulated under the assumption of 5-fold or 10-fold lower K_d_-values for HDAC4_o_ than for HDAC4_c_ (Figure 8B). The percentage of HDAC4_c_-I in chemical equilibrium was calculated for different ratios of HDAC4_c_ and HDAC4_o_ in terms of the ratio of rate constants of the corresponding conformational equilibrium, k_1_/k_−1_. As expected, the complex of HDAC4_o_-I dominated, if the ratio of HDAC4_c_/HDAC4_o_ was 1 or less (Figure 8B). However, conformational equilibria with 50-fold or higher ratios of HDAC4_c_ to HDAC4_o_ produced more than 80% HDAC4c-I in chemical equilibrium. 

This implies that the dominant presence of a complex between TZD ligands and HDAC4_c_ in chemical equilibrium in solution requires a strong shift of the conformational equilibrium towards HDAC4_c_, e.g., >50:1 HDAC4_c_:HDAC4_o_. This conclusion is in agreement with the fact, that most crystal structures of the catalytic domain of HDAC4 and closely related HDAC7 in apo-form as well as in complex with ligands adopt the *closed* conformation. Only a few crystal structures, all of them protein-ligand complexes, show the *open* conformation. Moreover, the *closed* conformation of HDAC4 is thought to be physiologically relevant, because only the *closed* conformation is able to associate with the N-CoR-HDAC3 repressor complex [3].

## 3. Materials and Methods

### 3.1. General Procedures

Chemical reagents and solvents were procured from S D Fine or Sigma Aldrich suppliers in India. Thin layer chromatography (TLC) was used to monitor the reaction at each step and TLC was carried out on Merck pre-coated Silica Gel 60 F254 by using mixture of suitable mobile phase. Melting point of the intermediates and final compounds were obtained from VEEGO, MODEL: VMP-DS Melting Point apparatus by open capillary method and are uncorrected. Purity of all the final products were confirmed by Agilent 1200 high-performance liquid chromatography (HPLC) system; software—EZ chrome Elite; chromatographic column—HemochromIntsil A31 C18 5U 150 mm × 4.6 mm Sn-B180127; detection at 300 nm; detector—UV-visible; flow rate—1 mL/min; oven temperature—30 °C; gradient elution run time—10 min; mobile phase—methanol: formic Acid (1%) (formic acid: in 1000 mL double distilled water 1 mL formic acid was added) in 80:20/90:10 ratio. The structures of intermediates were confirmed by FTIR and ^1^H-NMR and that of the final compounds by FTIR, ^1^H-NMR, ^13^C-NMR and Mass spectrometry. FTIR was recorded on Schimadzu FT/IR-8400S by direct sampling technique. ^1^H-NMR spectra were recorded by Bruker Avance 400 MHz spectrometer with DMSO-d_6_. All shifts are reported in δ (ppm) units relative to the signals for solvent DMSO (δ- 2.50 ppm). All coupling constants (J values) are reported in hertz (Hz). NMR abbreviations are: bs, broad singlet; s, singlet; d, doublet; t, triplet; q, quartet; m, multiplet; and dd, doublet of doublets. ^13^C-NMR was recorded on Bruker Avance spectrometer at 100 MHz with DMSO-d_6_. Mass spectrum was determined on LC-MS Agilent Technologies 1260 Infinity instrument. 

**2-(2,4-dioxo-5-(pyridin-2-ylmethylene)thiazolidin-3-yl)-*N*-(6-nitrobenzo[d]thiazol-2-yl) acetamide (PB1).** Yield 0.6 g (53%). M.P. 250 °C (charred). White color solid. IR (cm^−1^) 3340 (NH str.), 1739 (C=O str. of cyclic amide), 1658, 1612, 1573, 1548 (C=C str^1^H NMR (400 MHz, DMSO-d_6_, δ ppm): 4.68 (s, 2H), 7.31–7.35 (m, 1H), 7.46–7.49 (m, 1H), 7.55–7.57 (m, 1H), 7.92–8.00 (m, 3H), 8.04 (s, 1H), 8.81 (d, *J* = 4.4 Hz, 1H), 13.27 (s, 1H). ^13^C NMR (400 MHz, DMSO-d_6_): 171.41, 166.40, 165.89, 162.76, 159.00, 151.56, 149.86, 145.84, 138.17, 133.56, 130.17, 128.57, 126.82, 125.70, 125.18, 125.05, 124.80, 121.48, 43.34, 36.25. Theoretical mass: 441.44; LCMS (*m*/*z*, I%): 440.0 [(M-H)^+^, 100%]. HPLC Purity: % Area 97.55, Retention Time 6.32 min.

**2-(2,4-dioxo-5-(pyridin-2-ylmethylene)thiazolidin-3-yl)-*N*-(4-methylbenzo[d]thiazol-2-yl)acetamide (PB2).** Yield 0.5 g (46%). M.P. 260 °C (charred). White color solid. IR (cm^−1^) 3323 (NH str.), 1737 (C=O str. of cyclic amide), 1656, 1614, 1595, 1554 (C=C str.). ^1^H NMR (400 MHz, DMSO-d_6_, δ ppm): 2.59 (s, 3H), 4.68 (s, 2H), 7.20–7.28 (m, 2H), 7.46–7.49 (m, 1H), 7.80 (d, *J* = 7.6 Hz, 1H), 7.92–8.00 (m, 2H), 8.04 (s, 1H), 8.80 (d, *J* = 4.4 Hz, 1H), 12.95 (s, 1H). ^13^C NMR (400 MHz, DMSO-d_6_): 171.41, 165.95, 156.96, 151.55, 149.86, 148.03, 138.16, 131.63, 130.55, 130.15, 128.57, 127.21, 125.72, 124.79, 124.26, 119.67, 43.41, 18.38. Theoretical mass: 410.47; LCMS (*m*/*z*, I%): 409.1 [(M-H)^+^, 100%]. HPLC Purity: % Area 97.11, Retention Time 5.96 min.

**2-(2,4-dioxo-5-(pyridin-2-ylmethylene)thiazolidin-3-yl)-*N*-(6-methylbenzo[d]thiazol-2-yl)acetamide (PB3).** Yield 0.7 g (67%). M.P. charred at 300 °C. White color solid. IR (cm^−1^) 3323 (NH str.), 1730 (C=O str. of cyclic amide), 1693, 1664, 1616, 1546 (C=C str.). ^1^H NMR (400 MHz, DMSO-d_6_, δ ppm): 2.40 (s, 3H), 4.68 (s, 2H), 7.24–7.27 (m, 1H), 7.44–7.47 (m, 1H), 7.65 (d, *J* = 8.4 Hz, 1H), 7.75 (s, 1H), 7.91 (d, *J* = 7.6 Hz, 1H), 7.94–8.02 (m, 1H), 8.03 (s, 1H), 8.79 (d, *J* = 4.4 Hz, 1H), 12.78 (s, 1H). ^13^C NMR (400 MHz, DMSO-d_6_): 171.42, 165.91, 156.96, 151.54, 149.84, 146.93, 138.12, 133.80, 132.09, 130.51, 130.13, 128.54, 128.04, 125.71, 124.76, 121.86, 120.86, 43.45, 21.44. Theoretical mass: 410.47; LCMS (*m*/*z*, I%): 409.0 [(M-H)^+^, 100%]. HPLC Purity: % Area 95.25, Retention Time 5.56 min.

***N*-(4-chlorobenzo[d]thiazol-2-yl)-2-(2,4-dioxo-5-(pyridin-2-ylmethylene)thiazolidin-3-yl)acetamide (PB4).** Yield 0.75 g (72%). M.P. 273 °C (charred). White color solid. IR (cm^−1^) 3342 (NH str.), 1745 (C=O str. of cyclic amide), 1680, 1631, 1604, 1573, 1548 (C=C str.) 705 (C-Cl str.). ^1^H NMR (400 MHz, DMSO-d_6_, δ ppm): 4.69 (s, 2H), 7.31–7.35 (m, 1H), 7.46–7.49 (m, 1H), 7.55–7.57 (m, 1H), 7.92–8.00 (m, 3H), 8.04 (s, 1H), 8.80 (d, *J* = 4.4 Hz, 1H), 13.26 (s, 1H). ^13^C NMR (400 MHz, DMSO-d_6_): 171.41, 166.40, 165.89, 162.77, 159.00, 151.54, 149.86, 145.84, 138.17, 133.56, 130.17, 128.57, 126.82, 125.70, 125.18, 125.05, 124.80, 121.48, 43.44, 36.25, 31.24. Theoretical mass: 430.89; LCMS (*m*/*z*, I%): 428.9 [(M-H)^+^, 100%]. HPLC Purity: % Area 97.60, Retention Time 4.99 min.

***N*-(4,6-difluorobenzo[d]thiazol-2-yl)-2-(2,4-dioxo-5-(pyridin-2-ylmethylene)thiazolidin-3-yl)acetamide (PB5).** Yield 0.8 g (85%). M.P. 294 °C (charred). White color solid. IR (cm^−1^) 3360 (NH str.), 1728 (C=O str. of cyclic amide), 1672, 1614, 1577, 1552 (C=C str.), 1383, 1145 (C-F str.). ^1^H NMR (400 MHz, DMSO-d_6_, δ ppm): 4.69 (s, 2H), 7.37–7.42 (m, 1H), 7.46–7.49 (m, 1H), 7.81–7.83 (m, 1H), 7.92–8.00 (m, 2H), 8.04 (s, 1H), 8.80 (d, *J* = 4.0 Hz, 1H), 13.14 (s, 1H). ^13^C NMR (400 MHz, DMSO-d_6_): 171.42, 166.43, 165.89, 158.36, 157.51, 157.41, 151.53, 149.86, 138.17, 135.23, 130.17, 128.57, 125.71, 124.81, 105.22, 104.99, 102.86, 102.64, 102.57, 102.35, 43.42. Theoretical mass: 432.42; LCMS (*m*/*z*, I%): 431.0 [(M-H)^+^, 100%]. HPLC Purity: % Area 96.23, Retention Time 5.96 min.

***N*-(5,6-dimethylbenzo[d]thiazol-2-yl)-2-(2,4-dioxo-5-(pyridin-2-ylmethylene)thiazolidin-3-yl)acetamide (PB6).** Yield 0.52 g (58%). M.P. 295 °C (charred). White color solid. IR (cm^−1^) 3259 (NH str.), 1741 (C=O str. of cyclic amide), 1668, 1614, 1577, 1552 (C=C str.). ^1^H NMR (400 MHz, DMSO-d_6_, δ ppm): 2.32 (d, *J* = 4.4 Hz, 6H), 4.66 (s, 2H), 7.48–7.57 (m, 2H), 7.72 (s, 1H), 7.94–7.98 (m, 2H), 8.04 (s, 1H), 8.80 (s, 1H), 12.75 (s, 1H). ^13^C NMR (400 MHz, DMSO-d_6_): 171.42, 165.92, 165.78, 156.88, 151.56, 149.87, 147.54, 138.17, 135.45, 133.22, 130.13, 129.32, 128.57, 125.72, 124.80, 122.02, 121.56, 43.44, 20.16, 20.02. Theoretical mass: 424.50; LCMS (*m*/*z*, I%): 422.8 [(M-2H)^+^, 100%]. HPLC Purity: % Area 98.58, Retention Time 6.92 min.

**2-(2,4-dioxo-5-(pyridin-2-ylmethylene)thiazolidin-3-yl)-*N*-(4-methoxybenzo[d]thiazol-2-yl)acetamide (PB7).** Yield 0.4 g (53%). M.P. 220–222 °C. White color solid. IR (cm^−1^) 3296 (NH str.), 1737 (C=O str. of cyclic amide), 1680, 1597, 1562 (C=C str.). ^1^H NMR (400 MHz, DMSO-d_6_, δ ppm): 2.91 (s, 3H), 4.66 (s, 2H), 7.02 (d, *J* = 8.0 Hz, 1H), 7.28 (t, *J* = 8.0 Hz, 1H), 7.48 (t, *J* = 6.0 Hz, 1H), 7.54 (d, *J* = 8.0 Hz, 1H), 7.93–8.01 (m, 2H), 8.04 (s, 1H), 8.80 (d, *J* = 4.8 Hz, 1H), 12.96 (s, 1H). ^13^C NMR (400 MHz, DMSO-d_6_): 171.45, 165.94, 165.88, 151.58, 149.88, 138.18, 130.09, 128.57, 125.80, 125.31, 124.80, 114.06, 108.28, 56.31, 43.45, 29.49. Theoretical mass: 426.47; LCMS (*m*/*z*, I%): 425.1 [(M-H)^+^, 100%]. HPLC Purity: % Area 98.67, Retention Time 5.52 min.

**2-(2,4-dioxo-5-(pyridin-2-ylmethylene)thiazolidin-3-yl)-*N*-(6-ethoxybenzo[d]thiazol-2-yl)acetamide (PB8).** Yield 0.57 g (55%). M.P. 230 °C (charred). White color solid. IR (cm^−1^) 3259 (NH str.), 1737 (C=O str. of cyclic amide), 1666, 1599, 1552, 1535 (C=C str.). ^1^H NMR (400 MHz, DMSO-d_6_, δ ppm): 1.32–1.36 (m, 3H), 4.03–4.08 (m, 2H), 4.67 (s, 2H), 7.01–7.04 (m, 1H), 7.45–7.48 (m, 1H), 7.55 (s, 1H), 7.65 (d, *J* = 8.8 Hz, 1H), 7.91–7.99 (m, 2H), 8.03 (s, 1H), 8.79 (d, *J* = 4.4 Hz, 1H), 12.71 (s, 1H). ^13^C NMR (400 MHz, DMSO-d_6_): 171.43, 165.92, 165.75, 156.02, 155.73, 151.54, 149.84, 142.92, 138.13, 133.26, 130.13, 128.55, 125.71, 124.77, 121.81, 115.94, 105.83, 64.08, 43.40, 15.15. Theoretical mass: 440.50; LCMS (*m*/*z*, I%): 438.8 [(M-2H)^+^, 100%]. HPLC Purity: % Area 98.57, Retention Time 4.37 min.

**2-(2,4-dioxo-5-(pyridin-2-ylmethylene)thiazolidin-3-yl)-*N*-(6-fluorobenzo[d]thiazol-2-yl)acetamide (PB9).** Yield 0.64 g (60%). M.P. 265 °C (charred). White color solid. IR (cm^−1^) 3246 (NH str.), 1732 (C=O str. of cyclic amide), 1689, 1651, 1612, 1566, 1554 (C=C str.), 1390 (C-F str.). ^1^H NMR (400 MHz, DMSO-d_6_, δ ppm): 4.70 (s, 2H), 7.26–7.31 (m, 1H), 7.43–7.46 (m, 1H), 7.75–7.79 (m, 1H), 7.87–7.90 (m, 2H), 7.93–7.97 (m, 1H), 8.01 (s, 1H), 8.78 (d, *J* = 4.4 Hz, 1H), 12.88 (s, 1H). ^13^C NMR (400 MHz, DMSO-d_6_): 171.43, 166.18, 165.89, 160.43, 158.05, 157.86, 151.51, 149.81, 145.65, 138.09, 133.26, 133.15, 130.16, 128.53, 125.68, 124.74, 122.37, 122.29, 114.96, 114.71, 108.86, 108.60, 43.41. Theoretical mass: 414.43; LCMS (*m*/*z*, I%): 413.0 [(M-H)^+^, 100%]. HPLC Purity: % Area 95.13, Retention Time 4.89 min.

**2-(5-benzylidene-2,4-dioxo thiazolidine-3-yl)-*N*-(4-chlorobenzo[d]thiazol-2-yl)acetamide (GB1)** Yield 0.53 g (55%); M.P. 279–295 °C. White color solid. IR (cm^−1^) 3340 (NH str.), 1745 (C=O str. of cyclic amide), 1691, 1666, 1595, 1556 (C=C str.), 761 (C-Cl str.); ^1^H NMR (400 MHz, DMSO-d_6_, δ ppm): 4.69 (s, 2H), 7.26–7.28 (m, 1H), 7.63–7.67 (m, 3H), 7.69–7.78 (m, 3H), 7.78 (s, 1H), 8.01 (s, 1H), 12.78 (s, 1H). ^13^C NMR (100 MHz, DMSO-d_6_): 40.13, 43.54, 120.89, 120.96, 124.54, 124.68, 126.31, 129.40, 130.17, 130.83, 132.78, 133.04, 133.79, 145.31, 158.45, 165.09, 165.74, 167.04. Theoretical mass: 429.90; LCMS (*m*/*z*, I%): 428.0 [(M-H)^+^, 100%]. HPLC Purity: % Area 96.63, RT 8.57 min.

***N*-(4-chlorobenzo[d]thiazol-2-yl)-2-(5-(4-methylbenzylidene)-2,4-dioxothiazolidin-3-yl)acetamide (GB2)** Yield 0.6 g (58%); M.P. 292–294 °C. White color solid. IR (cm^−1^) 3340 (NH str.), 1739 (C=O str. of cyclic amide), 1691, 1664, 1595, 1562 (C=C str.), 771 (C-Cl str.); ^1^H NMR (400 MHz, DMSO-d_6_, δ ppm): 2.39 (s, 3H), 4.70 (s, 2H), 7.32–7.36 (m, 1H), 7.39–7.41 (m, 1H), 7.56–7.59 (m, 2H), 7.96 (s, 2H), 7.98–7.99 (m, 1H), 8.31 (s, 1H), 13.27 (s, 1H). ^13^C NMR (100 MHz, DMSO-d_6_): 21.10, 30.75, 35.76, 79.12, 130.06, 130.29, 162.31; Theoretical mass: 443.93; LCMS (*m*/*z*, I%): 441.9 [(M-2H)^+^, 100%]. HPLC Purity: % Area 98.9, RT 11.41 min.

***N*-(4-chlorobenzo[d]thiazol-2-yl)-2-(5-(4-chlorobenzylidene)-2,4-dioxothiazolidin-3-yl) acetamide (GB3)** Yield 0.5 g (55%); M.P. charred above 300°C. White color solid. IR (cm^−1^) 3346 (NH str.), 1741 (C=O str. of cyclic amide), 1683, 1666, 1597, 1554 (C=C str.), 817, 771 (C-Cl str.); ^1^H NMR (400 MHz, DMSO-d_6_, δ ppm): 4.71 (s, 2H), 7.34 (t, *J* = 8.0 Hz, 1H), 7.57 (d, *J* = 8.0 Hz, 1H), 7.63 (d, *J* = 8.4 Hz, 2H), 7.78 (d, *J* = 8.4 Hz, 2H), 7.99–8.00 (m, 2H), 13.26 (s, 1H). ^13^C NMR (100 MHz, DMSO-d_6_): 145.17, 133.21, 132.60, 132.43, 131.96, 126.35, 124.75, 124.31, 121.76, 120.98. UV Spectrum (10 ppm, λ_max_—330 nm, absorbance—0.723). Theoretical mass: 464.34; LCMS (*m*/*z*, I%): 463.9 [(M-H)^+^, 100%]. HPLC Purity: % Area 98.7, RT 4.4 min.

**2-(5-(4-bromobenzylidene)-2,4-dioxothiazolidin-3-yl)-*N*-(4-chlorobenzo[d]thiazol-2-yl)acetamide (GB4)** Yield 0.6 g (62%); M.P. charred above 300 °C. White color solid. IR (cm^−1^) 3335 (NH str.), 1737 (C=O str. of cyclic amide), 1697, 1651, 1595, 1554 (C=C str.), 765 (C-Cl str.), 609 (C-Br str.); ^1^H NMR (400 MHz, DMSO-d_6_, δ ppm): 4.71 (s, 2H), 7.32–7.36 (m, 1H), 7.56–7.58 (m, 1H), 7.62–7.64 (m, 2H), 7.77–7.79 (m, 2H), 7.99–8.0 (m, 2H), 13.26 (s, 1H). ^13^C NMR (100 MHz, DMSO-d_6_): 120.98, 121.76, 124.31, 124.75, 126.35, 131.96, 132.43, 132.60, 133.21, 145.17; Theoretical mass: 508.80; LCMS (*m*/*z*, I%): 507.8 [(M-H)^+^, 100%]. HPLC Purity: % Area 98.14, RT 9.1 min.

***N*-(4-chlorobenzo[d]thiazol-2-yl)-2-(5-(2,4-difluorobenzylidene)-2,4-dioxothiazolidin-3-yl) acetamide (GB5)** Yield 0.45 g (51%). M.P. 288–289 °C. White color solid. IR (cm^−1^) 3392 (NH str.), 1745 (C=O str. of cyclic amide), 1691, 1681, 1612, 1604, 1589, 1562 (C=C str.), 817 (C-Cl str.), 1276, 1143 (C-F str.). ^1^H NMR (400 MHz, DMSO-d_6_, δ ppm): 4.70 (s, 2H), 7.31–7.33 (m, 1H), 7.40 (d, *J* = 8.0 Hz, 1H), 7.43 (s, 1H), 7.56 (d, *J* = 8.0 Hz, 1H), 7.94 (d, *J* = 12.4 Hz, 2H), 7.99 (d, *J* = 8.0 Hz, 1H), 13.27 (s, 1H). ^13^C NMR (100 MHz, DMSO-d_6_): 167.15, 165.19, 162.31, 145.32, 140.25, 137.57, 134.00, 133.04, 131.37, 130.51, 130.38, 127.68, 126.34, 124.72, 124.57, 120.98, 119.41, 79.12, 43.47, 19.46, 19.35. UV Spectrum (10 ppm, λ_max_—328 nm, absorbance—0.442). Theoretical mass: 465.88; LCMS (*m*/*z*, I%): 463.9 [(M-H)^+^, 100%]. HPLC Purity: % Area 97.02, RT 4.01 min.

***N*-(4-chlorobenzo[d]thiazol-2-yl)-2-(5-(3,4-dimethylbenzylidene)-2,4-dioxothiazolidin-3-yl)acetamide (GB6)** Yield 0.61 g (65%); M.P. 214–216 °C. White color solid. IR (cm^−1^) 3342 (NH str.), 1743 (C=O str. of cyclic amide), 1687, 1668, 1597, 1564 (C=C str.), 773 (C-Cl str.); ^1^H NMR (400 MHz, DMSO-d_6_, δ ppm): 2.30 (s, 6H), 4.70 (s, 2H), 7.39–7.41 (m, 1H), 7.43 (m, 1H), 7.5–7.57 (m, 1H), 7.92–7.96 (m, 3H), 7.98–8.0 (m, 1H), 13.27 (s, 1H). ^13^C NMR (100 MHz, DMSO-d_6_): 19.46, 30.75, 35.76, 43.47, 79.12, 119.41, 120.98, 124.57, 124.72, 126.34, 127.68, 130.38, 130.51, 131.37, 133.04, 134.0, 137.57, 140.25, 145.32, 162.31, 165.19, 167.15 Theoretical mass: 457.95; LCMS (*m*/*z*, I%): 456.1 [(M-H)^+^, 100%]. HPLC Purity: % Area 95.05, RT 5.77 min.

**2-(5-(4-bromobenzylidene)-2,4-dioxothiazolidin-3-yl)-*N*-(4,6-difluorobenzo[d]thiazol-2-yl) acetamide (GB7)** Yield 0.63 g (59%); M.P. charred above 300°C. White color solid. IR (cm^−1^) 3275 (NH str.), 1743 (C=O str. of cyclic amide), 1691, 1660, 1608, 1573 (C=C str.), 1288, 1153 (C-F str.), 596 (C-Br str.). ^1^H NMR (400 MHz, DMSO-d_6_, δ ppm): 4.71 (s, 2H), 7.37–7.42 (m, 1H), 7.62 (d, *J* = 8.4 Hz, 2H), 7.78 (d, *J* = 8.4 Hz, 2H), 7.80–7.83 (m, 1H), 7.99 (s, 1H), 13.15 (s, 1H). ^13^C NMR (100 MHz, DMSO-d_6_): 166.83, 165.68, 165.03, 132.6, 132.42, 132.0, 131.95, 124.42, 121.74, 104.43, 79.12, 43.56. UV Spectrum (10 ppm, λ_max_—326 nm, absorbance—1.817). Theoretical mass: 510.33; LCMS (*m*/*z*, I%): 509.9 [(M-H)^+^, 100%]. HPLC Purity: % Area 97.20, RT 4.78 min.

**2-(5-benzylidene-2,4-dioxothiazolidin-3-yl)-*N*-(4-methoxybenzo[d]thiazol-2-yl)acetamide (GB8)** Yield 0.54 g (60%); M.P. 157–159 °C. White color solid. IR (cm^−1^) 3342 (NH str.), 1741 (C=O str. of cyclic amide), 1681, 1602, 1568 (C=C str.); ^1^H NMR (400 MHz, DMSO-d_6_, δ ppm): 2.93 (s, 3H), 4.68 (s, 2H), 7.01–7.03 (m, 1H), 7.27–7.31 (m, 1H), 7.52–7.60 (m, 4H), 7.67–7.69 (m, 2H), 8.02 (s, 1H), 12.97 (s, 1H). ^13^C NMR (100 MHz, DMSO-d_6_): 55.81, 107.79, 113.56, 120.98, 124.87, 129.43, 130.19, 130.85, 132.81, 133.74, 165.17, 167.13; Theoretical mass: 425.48; LCMS (*m*/*z*, I%): 424.0 [(M-H)^+^, 100%]. HPLC Purity: % Area 97.59, RT 2.98 min.

**2-(5-benzylidene-2,4-dioxothiazolidin-3-yl)-*N*-(4-methylbenzo[d]thiazol-2-yl)acetamide (GB9)** Yield 0.59 g (55%). M.P. 272–273 °C. White color solid. IR (cm^−1^) 3323 (NH str.), 1743, 1712 (C=O str. of cyclic amide), 1672, 1608, 1537 (C=C str.). ^1^H NMR (400 MHz, DMSO-d_6_, δ ppm): 2.59 (s, 3H), 4.70 (s, 2H), 7.21–7.29 (m, 2H), 7.53–7.60 (m, 3H), 7.67 (d, *J* = 7.2 Hz, 2H), 7.80 (d, *J* = 7.6 Hz, 1H), 8.01 (s, 1H), 12.96 (s, 1H). ^13^C NMR (100 MHz, DMSO-d_6_): 167.09, 165.14, 156.28, 147.53, 133.79, 132.79, 131.12, 130.85, 130.19, 130.09, 129.42, 126.73, 123.80, 120.90, 119.17, 43.49, 17.87. UV Spectrum (10 ppm, λ_max_—326 nm, absorbance—0.929). Theoretical mass: 409.48; LCMS (*m*/*z*, I%): 408.0 [(M-H)^+^, 100%]. HPLC Purity: % Area 97.73, RT 3.67 min.

**2-(5-(4-fluorobenzylidene)-2,4-dioxothiazolidin-3-yl)-*N*-(4-methylbenzo[d]thiazol-2-yl)acetamide (GB10)** Yield 0.6 g (64%); M.P. 225–227 °C. White color solid. IR (cm^−1^) 3344 (NH str.), 1741 (C=O str. of cyclic amide), 1687, 1633, 1595, 1510 (C=C str.), 1149 (C-F str.), 771 (C-Cl str.); ^1^H NMR (400 MHz, DMSO-d_6_, δ ppm): 2.74 (s, 3H), 4.69 (s, 2H), 7.21–7.29 (m, 2H), 7.40–7.45 (m, 2H), 7.74–7.81 (m, 2H), 7.96 (s, 1H), 8.03 (s, 1H), 12.96 (s, 1H). ^13^C NMR (100 MHz, DMSO-d_6_): 17.86, 30.75, 35.76, 43.52, 116.50, 116.72, 119.17, 120.60, 123.81, 126.73, 129.50, 130.09, 131.12, 132.68, 132.73, 132.77, 147.52, 161.85, 162.312, 165.10, 165.20, 165.31, 166.98; Theoretical mass: 427.47; LCMS (*m*/*z*, I%): 426.0 [(M-H)^+^, 100%]. HPLC Purity: % Area 97.97, RT 3.34 min.

**2-(5-(4-chlorobenzylidene)-2,4-dioxothiazolidin-3-yl)-*N*-(4,6-difluorobenzo[d]thiazol-2-yl)acetamide (GB11)** Yield 0.52 g (55%); M.P. charred above 300 °C. White color solid. IR (cm^−1^) 3267 (NH str.), 1743 (C=O str. of cyclic amide), 1693, 1662, 1608, 1573 (C=C str.), 1153, 1101 (C-F str.), 729 (C-Cl str.); ^1^H NMR (400 MHz, DMSO-d_6_, δ ppm): 4.71 (s, 2H), 7.36–7.42 (m, 1H), 7.62–7.64 (m, 2H), 7.68–7.71 (m, 2H), 7.80–7.83 (s, 1H), 8.01 (s, 1H), 13.15 (s, 1H). ^13^C NMR (100 MHz, DMSO-d_6_): 43.55, 102.15, 104.47, 104.74, 121.64, 129.48, 131.68, 131.82, 132.50, 135.48, 152.11, 157.78, 165.02, 165.67, 166.84; Theoretical mass: 465.88; LCMS (*m*/*z*, I%): 463.9 [(M-2H)^+^, 100%]. HPLC Purity: % Area 97.72, RT 4.40 min.

**2-(5-benzylidene-2,4-dioxothiazolidin-3-yl)-*N*-(4,6-difluorobenzo[d]thiazol-2-yl)acetamide (GB12)** Yield 0.57 g (60%); M.P. 280–282 °C. White color solid. IR (cm^−1^) 3335 (NH str.), 1751 (C=O str. of cyclic amide), 1685, 1626, 1595, 1579 (C=C str.), 1149, 1107 (C-F str.); ^1^H NMR (400 MHz, DMSO-d_6_, δ ppm): 4.71 (s, 2H), 7.40–7.42 (m, 1H), 7.53–7.60 (m, 2H), 7.67–7.69 (m, 3H), 7.81–7.83 (s, 1H), 7.95 (s, 1H), 13.15 (s, 1H). ^13^C NMR (100 MHz, DMSO-d_6_): 30.75, 35.75, 43.52, 120.89, 129.42, 130.19, 130.87, 132.78, 133.83, 162.31, 165.13, 165.68, 167.09; Theoretical mass: 431.44; LCMS (*m*/*z*, I%): 429.9 [(M-2H)^+^, 100%]. HPLC Purity: % Area 95.82, RT 3.31 min.

**2-(5-(2,4-difluorobenzylidene)-2,4-dioxothiazolidin-3-yl)-*N*-(6-methylbenzo[d]thiazol-2-yl)acetamide (GB13)** Yield 0.65 g (68%); M.P. 266–268 °C. White color solid. IR (cm^−1^) 3342 (NH str.), 1745 (C=O str. of cyclic amide), 1685, 1604, 1585, 1548 (C=C str.), 1143, 1107 (C-F str.); ^1^H NMR (400 MHz, DMSO-d_6_, δ ppm): 2.42 (s, 3H), 4.70 (s, 2H), 7.27–7.29 (m, 1H), 7.31–7.36 (m, 1H), 7.49–7.55 (m, 1H), 7.66–7.74 (m, 2H), 7.78 (s, 1H), 7.93 (s, 1H), 12.78 (s, 1H). ^13^C NMR (100 MHz, DMSO-d_6_): 20.94, 79.12, 105.17, 112.99, 120.41, 121.38, 123.50, 124.24, 127.60, 130.77, 133.39, 164.84, 166.70; Theoretical mass: 445.46; LCMS (*m*/*z*, I%): 444.0 [(M-H)^+^, 100%]. HPLC Purity: % Area 97.33, RT 3.7 min.

***N*-(6-methylbenzo[d]thiazol-2-yl)-2-(5-(4-methylbenzylidene)-2,4-dioxothiazolidin-3-yl) acetamide (GB14)** Yield 0.49 g (56%). M.P. 268–269 °C. White color solid. IR (cm^−1^) 3342 (NH str.), 1739, 1701 (C=O str. of cyclic amide), 1660, 1593, 1546, 1512 (C=C str.). ^1^H NMR (400 MHz, DMSO-d_6_, δ ppm): 2.39 (s, 3H), 2.41 (s, 3H), 4.69 (s, 2H), 7.28 (d, *J* = 8.0 Hz, 2H), 7.39 (d, *J* = 8.0 Hz, 2H), 7.57 (d, *J* = 8.0 Hz, 2H), 7.67 (d, *J* = 8.0 Hz, 1H), 7.78 (s, 1H), 12.78 (s, 1H). ^13^C NMR (100 MHz, DMSO-d_6_): 167.11, 165.21, 146.41, 141.28, 133.87, 133.37, 131.59, 130.28, 130.05, 127.58, 121.37, 120.40, 119.64, 43.50, 21.10, 20.94. UV Spectrum (10 ppm, λ_max_—331 nm, absorbance—0.543). Theoretical mass: 423.51; LCMS (*m*/*z*, I%): 422.0 [(M-H)^+^, 100%]. HPLC Purity: % Area 95.87, RT 4.31 min.

**2-(5-(3,4-dimethylbenzylidene)-2,4-dioxothiazolidin-3-yl)-*N*-(6-methylbenzo[d]thiazol-2-yl) acetamide (GB15)** Yield 0.62 g (65%). M.P. 285–286 °C. White color solid. IR (cm^−1^) 3342 (NH str.), 1739, 1701 (C=O str. of cyclic amide), 1658, 1593, 1548, 1502 (C=C str.). ^1^H NMR (400 MHz, DMSO-d_6_, δ ppm): 2.30 (s, 6H), 2.41 (s, 3H), 4.68 (s, 2H), 7.28 (d, *J* = 8.0 Hz, 1H), 7.34 (d, *J* = 7.6 Hz, 1H), 7.40 (d, *J* = 8.0 Hz, 1H), 7.44 (s, 2H), 7.66 (d, *J* = 8.0 Hz, 1H), 7.78 (s, 1H), 12.78 (s, 1H). ^13^C NMR (100 MHz, DMSO-d_6_): 167.16, 165.22, 146.41, 140.25, 137.58, 133.98, 133.37, 131.59, 131.37, 130.52, 130.40, 127.68, 127.59, 121.38, 120.40, 119.42, 43.48, 20.94, 19.47, 19.35. UV Spectrum (10 ppm, λ_max_—334 nm, absorbance—1.079). Theoretical mass: 437.53; LCMS (*m*/*z*, I%): 436.0 [(M-H)^+^, 100%]. HPLC Purity: % Area 96.08, RT 5.36 min.

**2-(5-(4-chlorobenzylidene)-2,4-dioxothiazolidin-3-yl)-*N*-(6-methylbenzo[d]thiazol-2-yl) acetamide (GB16)** Yield: 0.39 g (48%). M.P. 283–284 °C. White color solid. IR (cm^−1^) 3304 (NH str.), 1739, 1703 (C=O str. of cyclic amide), 1662, 1602, 1585 (C=C str.), 858 (C-Cl str.). ^1^H NMR (400 MHz, DMSO-d_6_, δ ppm): 2.39 (s, 3H), 4.70 (s, 2H), 7.34 (t, *J* = 8.0 Hz, 1H), 7.40 (d, *J* = 8.0 Hz, 1H), 7.56–7.59 (m, 2H), 7.96 (s, 1H), 7.98–7.99 (m, 1H), 8.31 (s, 1H), 13.27 (s, 1H). ^13^C NMR (100 MHz, DMSO-d_6_): 162.31, 130.29, 130.06, 79.12, 35.76, 30.75, 21.10. UV Spectrum (10 ppm, λ_max_—330 nm, absorbance—1.196). Theoretical mass: 443.93; LCMS (*m*/*z*, I%): 441.9 [(M-H)^+^, 100%]. HPLC Purity: % Area 96.02, RT 5.45 min.

**2-(5-(4-chlorobenzylidene)-2,4-dioxothiazolidin-3-yl)-*N*-(6-ethoxybenzo[d]thiazol-2-yl) acetamide (GB17)** Yield 0.47 g (49%). M.P. 260.5–261.5 °C. White color solid. IR (cm^−1^) 3304 (NH str.), 1741 (C=O str. of cyclic amide), 1666, 1604, 1587, 1566, 1548 (C=C str.), 815 (C-Cl str.). ^1^H NMR (400 MHz, DMSO-d_6_, δ ppm): 1.33–1.37 (m, 3H), 4.05–4.08 (m, 2H), 4.69 (s, 2H), 7.04 (dd, *J* = 2.8, 8.8 Hz, 1H), 7.57 (d, *J* = 2.4 Hz, 1H), 7.63–7.72 (m, 5H), 8.02 (s, 1H), 12.71 (s, 1H). ^13^C NMR (100 MHz, DMSO-d_6_): 166.84, 165.06, 155.54, 135.47, 132.47, 131.83, 131.71, 129.50, 121.67, 121.37, 115.50, 105.37, 79.12, 63.60, 43.52, 14.65. UV Spectrum (10 ppm, λ_max_—329 nm, absorbance—0.622). Theoretical mass: 473.95; LCMS (*m*/*z*, I%): 472.0 [(M-H)^+^, 100%]. HPLC Purity: % Area 95.63, RT 4.30 min.

***N*-(4,6-difluorobenzo[d]thiazol-2-yl)-2-(5-(4-fluorobenzylidene)-2,4-dioxothiazolidin-3-yl)acetamide (GB18)** Yield 0.53 g (57%); M.P. charred above 300 °C. White color solid. IR (cm^−1^) 3398 (NH str.), 1741 (C=O str. of cyclic amide), 1695, 1662, 1622, 1599 (C=C str.), 1286, 1149, 1101 (C-F str.); ^1^H NMR (400 MHz, DMSO-d_6_, δ ppm): 4.71 (s, 2H), 7.37–7.45 (m, 3H), 7.74–7.77 (m, 2H), 7.80–7.83 (m, 1H), 8.31 (s, 1H), 13.15 (s, 1H). ^13^C NMR (100 MHz, DMSO-d_6_): 30.75, 35.75, 43.52, 79.12, 102.09, 102.37, 104.49, 104.73, 116.50, 116.72, 120.60, 129.47, 132.68, 132.77, 157.69, 161.86, 162.30, 164.36, 165.09, 166.98; Theoretical mass: 449.43; LCMS (*m*/*z*, I%): 447.9 [(M-2H)^+^, 100%]. HPLC Purity: % Area 95.31, RT 3.34 min.

***N*-(4,6-difluorobenzo[d]thiazol-2-yl)-2-(5-(4-methylbenzylidene)-2,4-dioxothiazolidin-3-yl)acetamide (GB19)** Yield 0.53 g (58%); M.P. 295–297 °C. White color solid. IR (cm^−1^) 3348 (NH str.), 1741 (C=O str. of cyclic amide), 1664, 1624, 1575 (C=C str.), 1147, 1103 (C-F str.); ^1^H NMR (400 MHz, DMSO-d_6_, δ ppm): 2.38 (s, 3H), 4.70 (s, 2H), 7.37–7.41 (m, 1H), 7.55–7.57 (m, 2H), 7.80–7.82 (m, 1H), 7.97 (m, 1H), 8.31 (s, 1H), 13.15 (s, 1H); ^13^C NMR (100 MHz, DMSO-d_6_): 21.09, 30.74, 35.75, 43.46, 79.12, 101.85, 102.08, 102.36, 104.43, 104.70, 133.89, 134.64, 134.72, 141.28, 152.11, 154.50, 154.64, 156.92, 157.03, 157.67, 162.30, 165.18, 167.11; Theoretical mass: 445.46; LCMS (*m*/*z*, I%): 444.0 [(M-H)^+^, 100%]. HPLC Purity: % Area 95.99, RT 4.19 min.

***N*-(4,6-difluorobenzo[d]thiazol-2-yl)-2-(5-(3,4-dimethylbenzylidene)-2,4-dioxothiazolidin-3-yl)acetamide (GB20)** Yield 0.63 g (65%); M.P. 271–273 °C. White color solid. IR (cm^−1^) 3398 (NH str.), 1741 (C=O str. of cyclic amide), 1697, 1664, 1641, 1622 (C=C str.), 1284, 1147 (C-F str.); ^1^H NMR (400 MHz, DMSO-d_6_, δ ppm): 2.30 (s, 6H), 4.70 (s, 2H), 7.34–7.36 (m, 1H), 7.37–7.44 (m, 2H), 7.81–7.83 (m, 1H), 7.93–7.96 (m, 2H), 13.14 (s, 1H); ^13^C NMR (100 MHz, DMSO-d_6_): 19.84, 31.25, 36.26, 43.97, 79.62, 119.93, 128.18, 130.89, 131.03, 131.87, 134.51, 138.09, 140.77, 162.82, 165.70, 167.67; Theoretical mass: 459.49; LCMS (*m*/*z*, I%): 458.0 [(M-H)^+^, 100%]. HPLC Purity: % Area 97.49, RT 5.21 min.

***N*-(5,6-dimethylbenzo[d]thiazol-2-yl)-2-(5-(4-methylbenzylidene)-2,4-dioxothiazolidin-3-yl) acetamide (GB21)** Yield 0.52 g (53%). M.P. 283–285 °C. White color solid. IR (cm^−1^) 3246 (NH str.), 1741 (C=O str. of cyclic amide), 1697, 1664, 1593, 1550 (C=C str.). ^1^H NMR (400 MHz, DMSO-d_6_, δ ppm): 2.32 (d, *J* = 6.4 Hz, 6H), 2.39 (s, 3H), 4.67 (s, 2H), 7.39 (d, *J* = 8.0 Hz, 2H), 7.57 (d, *J* = 8.0 Hz, 3H), 7.73 (s, 1H), 7.97 (s, 1H), 12.75 (s, 1H). ^13^C NMR (100 MHz, DMSO-d_6_): 167.12, 165.21, 146.95, 141.27, 133.85, 132.77, 130.28, 130.06, 121.51, 119.65, 21.10, 19.65, 19.51. UV Spectrum (10 ppm, λ_max_—332 nm, absorbance—0.388). Theoretical mass: 437.53; LCMS (*m*/*z*, I%): 435.8 [(M-H)^+^, 100%]. HPLC Purity: % Area 95.85, RT 5.47 min.

**2-(5-benzylidene-2,4-dioxothiazolidin-3-yl)-*N*-(5,6-dimethylbenzo[d]thiazol-2-yl)acetamide (GB22)** Yield 0.57 g (60%); M.P. 276–278 °C. White color solid. IR (cm^−1^) 3398 (NH str.), 1743 (C=O str. of cyclic amide), 1666, 1597, 1548 (C=C str.); ^1^H NMR (400 MHz, DMSO-d_6_, δ ppm): 2.32 (d, *J =* 6.4 Hz, 3H), 2.39 (s, 3H), 4.67 (s, 2H), 7.39 (d, *J =* 8.0 Hz, 2H), 7.57 (d, *J =* 8.0 Hz, 3H), 7.73 (s, 2H), 7.97 (s, 1H), 12.75 (s, 1H). ^13^C NMR (100 MHz, DMSO-d_6_): 167.12, 165.21, 146.95, 141.27, 133.85, 132.77, 130.28, 130.06, 121.51, 119.65, 21.10, 19.65, 19.51. Theoretical mass: 423.51; LCMS (*m*/*z*, I%): 421.9 [(M-2H)^+^, 100%]. HPLC Purity: % Area 97.41, RT 4.62 min.

**2-(5-(4-bromobenzylidene)-2,4-dioxothiazolidin-3-yl)-*N*-(5,6-dimethylbenzo[d]thiazol-2-yl)acetamide (GB23)** Yield 0.59 g (60%); M.P. charred above 300 °C. White color solid. IR (cm^−1^) 3398 (NH str.), 1747 (C=O str. of cyclic amide), 1697, 1655, 1604, 1546 (C=C str.), 650 (C-Br str.); ^1^H-NMR (400 MHz, DMSO-d_6_, δ ppm): 2.42 (s, 6H, CH_3_), 4.70 (s, 2H, CH_2_), 7.27–7.36 (m, 2H, ArH), 7.49–7.55 (m, 1H, ArH), 7.66–7.78 (m, 3H, ArH and benzylidene proton), 7.93 (s, 1H, ArH), 12.78 (bs, 1H, NH). ^13^C-NMR (100 MHz, DMSO-d_6_): 166.8, 165.0, 135.4, 133.3, 132.4, 131.8, 131.6, 129.4, 127.6, 121.6, 121.3, 120.3, 43.9, 19.9, 19.8. Theoretical mass: 502.40; LCMS (*m*/*z*, I%): 501.7 [(M-H)^+^, 100%]. HPLC Purity: % Area 96.22, RT 5.93 min.

**2-(5-(4-chlorobenzylidene)-2,4-dioxothiazolidin-3-yl)-*N*-(5,6-dimethylbenzo[d]thiazol-2-yl) acetamide (GB24)** Yield 0.63 g (68%). M.P. charred above 300 °C. White color solid. IR (cm^−1^) 3398 (NH str.), 1745, 1701 (C=O str. of cyclic amide), 1666, 1604, 1587, 1546 (C=C str.), 854 (C-Cl str.). ^1^H NMR (400 MHz, DMSO-d_6_, δ ppm): 2.32 (d, *J* = 6.0 Hz, 6H), 4.69 (s, 2H), 7.57 (s, 1H), 7.63–7.65 (m, 2H), 7.69–7.73 (m, 3H), 8.01 (s, 1H), 12.76 (s, 1H). ^13^C NMR (100 MHz, DMSO-d_6_): 166.84, 165.05, 135.46, 135.00, 132.78, 132.46, 131.83, 131.71, 129.50, 121.67, 121.52, 19.65, 19.52. UV Spectrum (10 ppm, λ_max_—329 nm, absorbance—0.219). Theoretical mass: 457.95; LCMS (*m*/*z*, I%): 455.7 [(M-H)^+^, 90%]. HPLC Purity: % Area 95.52, RT 5.04 min.

***N*-(5,6-dimethylbenzo[d]thiazol-2-yl)-2-(5-(4-fluorobenzylidene)-2,4-dioxothiazolidin-3-yl)acetamide (GB25)** Yield 0.54 g (59%); M.P. 298–299 °C. White color solid. IR (cm^−1^) 3398 (NH str.), 1745 (C=O str. of cyclic amide), 1699, 1662, 1597 (C=C str.), 1141 (C-F str.); ^1^H-NMR (400 MHz, DMSO-d_6_, δ ppm): 2.32–2.33 (d, J=2 Hz, 6H, CH_3_), 4.69 (s, 2H, CH_2_), 7.57 (s, 1H, benzylidene proton), 7.63–7.73 (m, 5H, ArH), 8.01 (s, 1H, ArH), 12.76 (bs, 1H, NH). ^13^C-NMR (100 MHz, DMSO-d_6_): 167.3, 167.2, 155.9, 138.4, 138.3, 130.9, 130.6, 126.8, 126.1, 125.1, 121.0, 120.7, 119.6, 119.5, 111.5, 67.3, 16.0, 13.4. Theoretical mass: 441.50; LCMS (*m*/*z*, I%): 440.0 [(M-H)^+^, 100%]. HPLC Purity: % Area 97.31, RT 4.0 min.

***N*-(5,6-dimethylbenzo[d]thiazol-2-yl)-2-(5-(3,4-dimethylbenzylidene)-2,4-dioxothiazolidin-3-yl)acetamide (GB26)** Yield 0.59 g (60%); M.P. 284–286 °C. White color solid. IR (cm^−1^) 3335 (NH str.), 1741 (C=O str. of cyclic amide), 1697, 1658, 1593, 1546 (C=C str.); ^1^H-NMR (400 MHz, DMSO-d_6_, δ ppm): 2.74 (m, 6H, CH_3_), 2.90 (m, 6H, CH_3_), 4.70 (s, 2H, CH_2_), 7.34–7.38 (m, 1H, ArH), 7.39–7.44 (m, 2H, ArH), 7.81–7.83 (m, 1H), 7.93–7.96 (d, J=10.8 Hz, 2H, ArH), 13.14 (bs, 1H, NH). ^13^C-NMR (100 MHz, DMSO-d_6_): 165.7, 165.6, 162.8, 140.7, 138.0, 134.5, 131.8, 131.0, 130.8, 128.1, 119.9, 79.6, 43.9, 19.9, 19.8. Theoretical mass: 451.56; LCMS (*m*/*z*, I%): 450.0 [(M-H)^+^, 100%]. HPLC Purity: % Area 98.14, RT 6.48 min.

***N*-(4-methoxybenzo[d]thiazol-2-yl)-2-(5-(4-methylbenzylidene)-2,4-dioxothiazolidin-3-yl) acetamide (GB27)** Yield 0.59 g (63%). M.P. 249–251 °C. White color solid. IR (cm^−1^) 3342 (NH str.), 1745, 1708 (C=O str. of cyclic amide), 1687, 1631, 1599, 1566, 1514 (C=C str.). ^1^H NMR (400 MHz, DMSO-d_6_, δ ppm): 2.59 (s, 3H), 3.92 (s, 3H), 4.68 (s, 2H), 7.01 (d, *J* = 7.6 Hz, 1H), 7.28 (t, *J* = 8.0 Hz, 1H), 7.42 (t, *J* = 8.8 Hz, 2H), 7.54 (d, *J* = 8.0 Hz, 1H), 7.73–7.77 (m, 2H), 8.02 (s, 1H), 12.97 (s, 1H). ^13^C NMR (100 MHz, DMSO-d_6_): 167.01, 165.12, 164.34, 161.85, 156.11, 151.93, 138.26, 132.82, 132.75, 132.67, 129.50, 129.46, 124.86, 120.68, 116.72, 116.50, 113.54, 107.79, 43.57. UV Spectrum (10 ppm, λ_max_—322 nm, absorbance—0.473). Theoretical mass: 439.51; LCMS (*m*/*z*, I%): 438.0 [(M-H)^+^, 100%]. HPLC Purity: % Area 95.65, RT 3.05 min.

**2-(5-benzylidene-2,4-dioxothiazolidin-3-yl)-*N*-(4-methylbenzo[d]thiazol-2-yl)acetamide (GB28)** Yield 0.60 g (62%); M.P. 278–280 °C. White color solid. IR (cm^−1^) 3321 (NH str.), 1741 (C=O str. of cyclic amide), 1693, 1666, 1599, 1548 (C=C str.); ^1^H NMR (400 MHz, DMSO-d_6_, δ ppm): 2.59 (s, 3H), 4.70 (s, 2H), 7.21–7.29 (m, 2H), 7.53–7.60 (m, 3H), 7.67 (d, *J =* 7.2 Hz, 2H), 7.80 (d, *J =* 7.6 Hz, 1H), 8.01 (s, 1H), 12.96 (s, 1H). ^13^C NMR (100 MHz, DMSO-d_6_): 167.09, 165.14, 156.28, 147.53, 133.79, 132.79, 131.12, 130.85, 130.19, 130.09, 129.42, 126.73, 123.80, 120.90, 119.17, 43.49, 17.87. Theoretical mass: 409.48; LCMS (*m*/*z*, I%): 408.0 [(M-H)^+^, 100%]. HPLC Purity: % Area 95.09, RT 3.46 min.

***N*-(4,6-difluorobenzo[d]thiazol-2-yl)-2-(5-(2,4-difluorobenzylidene)-2,4-dioxothiazolidin-3-yl)acetamide (GB29)** Yield 0.62 g (70%); M.P. 274–276 °C. White color solid. IR (cm^−1^) 3392 (NH str.), 1743 (C=O str. of cyclic amide), 1693, 1666, 1612, 1573 (C=C str.), 1244, 1192, 1145, 1199 (C-F str.); ^1^H-NMR (400 MHz, DMSO-d_6_, δ ppm): 4.69 (s, 2H, CH_2_), 7.02 (dd, J=2.8Hz, 2.4 Hz, 1H, ArH), 7.57–7.72 (m, 4H, ArH and benzylidene proton), 8.02 (s, 1H, ArH), 12.71 (bs, 1H, NH). ^13^C-NMR (100 MHz, DMSO-d_6_): 167.1, 165.2, 146.9, 141.2, 133.8, 132.7, 130.2, 130.0, 121.5, 119.6, 43.9. Theoretical mass: 467.42; LCMS (*m*/*z*, I%): 466.0 [(M-H)^+^, 100%]. HPLC Purity: % Area 97.63, RT 3.62 min.

**2-(5-benzylidene-2,4-dioxothiazolidin-3-yl)-*N*-(4-fluorobenzo[d]thiazol-2-yl)acetamide (GB30)** Yield 0.63 g (68%); M.P. 272–274 °C. White color solid. IR (cm^−1^) 3398 (NH str.), 1753 (C=O str. of cyclic amide), 1705, 1668, 1604, 1554 (C=C str.), 1147 (C-F str.); ^1^H-NMR (400 MHz, DMSO-d_6_, δ ppm): 4.69 (s, 2H, CH_2_), 7.21–7.29 (m, 2H, ArH), 7.40–7.45 (t, 2H, ArH), 7.74–7.81 (m, 3H, ArH and benzylidene proton), 7.96 (s, 1H, ArH), 12.96 (bs, 1H, NH). ^13^C-NMR (100 MHz, DMSO-d_6_): 166.9, 165.3, 165.2, 165.1, 162.3, 161.8, 147.5, 132.7, 132.6, 131.1, 130.0, 129.5, 126.7, 123.8, 120.6, 119.1, 116.7, 116.5, 43.5, 17.8. Theoretical mass: 413.45; LCMS (*m*/*z*, I%): 412.0 [(M-H)^+^, 100%]. HPLC Purity: % Area 95.28, RT 3.05 min.

**2-(5-(4-bromobenzylidene)-2,4-dioxothiazolidin-3-yl)-*N*-(6-methylbenzo[d]thiazol-2-yl)acetamide (GB31)** Yield 0.62 g (65%); M.P. 292–294 °C. White color solid. IR (cm^−1^) 3265 (NH str.), 1753 (C=O str. of cyclic amide), 1703, 1693, 1666, 1602 (C=C str.), 688 (C-Br str.); ^1^H-NMR (400 MHz, DMSO-d_6_, δ ppm): 2.41 (s, 3H, CH_3_), 4.69 (s, 2H, CH_2_), 7.26–7.28 (m, 1H, ArH), 7.63–7.78 (m, 6H, ArH and benzylidene proton), 8.01 (s, 1H, ArH), 12.78 (bs, 1H, NH). ^13^C-NMR (100 MHz, DMSO-d_6_): 166.8, 165.6, 165.0, 132.6, 132.4, 132.0, 131.9, 124.4, 121.7, 104.4, 79.1, 43.5, 21.7. Theoretical mass: 488.38; LCMS (*m*/*z*, I%): 487.9 [(M-H)^+^, 100%]. HPLC Purity: % Area 95.35, RT 4.85 min.

***N*-(6-fluorobenzo[d]thiazol-2-yl)-2-(5-(4-methylbenzylidene)-2,4-dioxothiazolidin-3-yl) acetamide (GB32)** Yield 0.52 g (58%). M.P. 274–275 °C. White color solid. IR (cm^−1^) 3259 (NH str.), 1737, 1703 (C=O str. of cyclic amide), 1658, 1591, 1552 (C=C str.), 1139 (C-F str.). ^1^H NMR (400 MHz, DMSO-d_6_, δ ppm): 2.74 (s, 3H), 4.69 (s, 2H), 7.21–7.29 (m, 2H), 7.43 (t, *J* = 8.8 Hz, 2H), 7.74–7.81 (m, 2H), 7.96 (s, 1H), 8.03 (s, 1H), 12.96 (s, 1H). ^13^C NMR (100 MHz, DMSO-d_6_): 166.98, 165.31, 165.20, 165.10, 162.31, 161.85, 147.52, 132.77, 132.73, 132.68, 131.12, 130.09, 129.50, 126.73, 123.81, 120.60, 119.17, 116.72, 116.50, 43.52, 35.76, 30.75, 17.86. UV Spectrum (10 ppm, λ_max_—329 nm, absorbance—0.843). Theoretical mass: 427.47; LCMS (*m*/*z*, I%): 425.9 [(M-H)^+^, 100%]. HPLC Purity: % Area 95.94, RT 3.98 min.

**2-(5-(4-chlorobenzylidene)-2,4-dioxothiazolidin-3-yl)-*N*-(6-fluorobenzo[d]thiazol-2-yl)acetamide (GB33)** Yield 0.63 g (65%); M.P. 290–292 °C. White color solid. IR (cm^−1^) 3387 (NH str.), 1739 (C=O str. of cyclic amide), 1704, 1664, 1600, 1585 (C=C str.), 1139 (C-F str.), 705 (C-Cl str.); ^1^H-NMR (400 MHz, DMSO-d_6_, δ ppm): 4.71 (s, 2H, CH_2_), 7.36–7.42 (m, 1H, ArH), 7.62–7.71 (dd, J=8.4 Hz, 8.8 Hz, 2H, ArH), 7.80–7.83 (m, 4H, ArH and benzylidene proton), 8.01 (s, 1H, ArH), 12.97 (bs, 1H, NH). ^13^C-NMR (100 MHz, DMSO-d_6_): 167.1, 165.2, 146.4, 141.2, 133.8, 133.3, 131.5, 130.2, 130.0, 127.5, 121.3, 120.4, 119.6, 43.5. Theoretical mass: 447.89; LCMS (*m*/*z*, I%): 445.9 [(M-2H)^+^, 100%]. HPLC Purity: % Area 95.97, RT 3.98 min.

***N*-(6-fluorobenzo[d]thiazol-2-yl)-2-(5-(4-fluorobenzylidene)-2,4-dioxothiazolidin-3-yl)acetamide (GB34)** Yield 0.64 g (68%); M.P. 260–262 °C. White color solid. IR (cm^−1^) 3398 (NH str.), 1741 (C=O str. of cyclic amide), 1712, 1674, 1589, 1545 (C=C str.), 1195, 1138 (C-F str.); ^1^H-NMR (400 MHz, DMSO-d_6_, δ ppm): 4.68 (s, 2H, CH_2_), 7.37–7.42 (m, 1H, ArH), 7.61–7.63 (d, J=8.4 Hz, 2H, ArH), 7.77–7.83 (m, 4H, ArH and benzylidene proton), 7.99 (s, 1H, ArH), 13.14 (bs, 1H, NH). ^13^C-NMR (100 MHz, DMSO-d_6_): 167.1, 165.2, 146.4, 140.2, 137.5, 133.9, 133.3, 131.5, 131.3, 130.5, 130.4, 127.6, 127.5, 121.3, 120.4, 119.4, 43.4. Theoretical mass: 431.44; LCMS (*m*/*z*, I%): 430.0 [(M-H)^+^, 100%]. HPLC Purity: % Area 97.87, RT 3.04 min.

**2-(5-(4-fluorobenzylidene)-2,4-dioxothiazolidin-3-yl)-*N*-(4-methoxybenzo[d]thiazol-2-yl)acetamide (GB35)** Yield 0.58 g (60%); M.P. 172–172 °C. White color solid. IR (cm^−1^) 3279 (NH str.), 1743 (C=O str. of cyclic amide), 1687, 1597, 1566, 1554 (C=C str.), 1147 (C-F str.); ^1^H NMR (400 MHz, DMSO-d_6_, δ ppm): 3.92 (s, 3H), 4.68 (s, 2H), 7.01–7.02 (m, 1H), 7.26–7.30 (m, 1H), 7.40–7.44 (m, 2H), 7.53–7.55 (m, 1H), 7.73–7.77 (m, 2H), 8.02 (s, 1H), 12.97 (s, 1H); ^13^C NMR (100 MHz, DMSO-d_6_): 43.57, 55.80, 79.12, 107.79, 113.54, 116.50, 116.72, 120.68, 124.86, 129.46, 129.50, 132.67, 132.75, 132.82, 138.26, 151.93, 156.11, 161.85, 164.34, 165.12, 167.01; Theoretical mass: 443.47; LCMS (*m*/*z*, I%): 441.9 [(M-2H)^+^, 100%]. HPLC Purity: % Area 96.52, RT 3.05 min.

**2-(5-benzylidene-2,4-dioxothiazolidin-3-yl)-*N*-(6-ethoxybenzo[d]thiazol-2-yl)acetamide (GB36)** Yield 0.55 g (59%); M.P. charred above 300 °C. White color solid. IR (cm^−1^) 3265 (NH str.), 1739 (C=O str. of cyclic amide), 1695, 1666, 1599, 1550 (C=C str.); ^1^H NMR (400 MHz, DMSO-d_6_, δ ppm): 1.33–1.37 (m, 3H), 4.05–4.08 (m, 2H), 4.69 (s, 2H), 7.04 (dd, *J =* 2.8, 8.8 Hz, 1H), 7.57 (d, *J =* 2.4 Hz, 2H), 7.63–7.72 (m, 5H), 8.02 (s, 1H), 12.71 (s, 1H). ^13^C NMR (100 MHz, DMSO-d_6_): 166.84, 165.06, 155.54, 135.47, 132.47, 131.83, 131.71, 129.50, 121.67, 121.37, 115.50, 105.37, 79.12, 63.60, 43.52, 14.65. Theoretical mass: 439.51; LCMS (*m*/*z*, I%): 438.0 [(M-H)^+^, 100%]. HPLC Purity: % Area 96.27, RT 2.94 min.

### 3.2. Mutagenesis, Recombinant Production and Purification of cdHDAC4

The catalytic domain of HDAC4 (cdHDAC4, T648–T1057; construct available in Supporting Information Table S3) was produced in *E. coli* (BL21) DE3 pLysS cells using a pET14b vector containing the cdHDAC4 gene fused with His6-SUMO tag. The respective mutants were generated by splicing by overlap extension polymerase chain reaction (SOE-PCR) [30] with the mutagenesis primers as listed in Table S4. An overnight culture of cells was incubated at 37 °C and 180 rpm in sterile Lennox LB media (20 g/L) containing 100 µg/mL ampicillin and 34 µg/mL chloramphenicol. Subsequently ca. 90 mL of overnight culture was equally transferred to three separate flasks containing a sterile solution of 26.6 g/L auto induction media (containing 3.08 g/L KH_2_PO_4_, 3.10 g/L Na_2_HPO_4_, 0.44 g/L MgSO_4_ and 20 g/L Lennox LB media), 4.6 g/L glycerol, 0.45 g/L glucose, 1.2 g/L lactose, 100 µg/mL ampicillin and 34 µg/mL chloramphenicol. The flasks were incubated at 30 °C and 225 rpm overnight. Cell harvest was performed at 8000 g for 10 min at 4 °C (6–16K centrifuge, Sigma, Osterode am Harz, Germany), the supernatant was discarded, cells were resuspended in IMAC-Buffer A (150 mM KCl, 50 mM TRIS-HCl, pH 8.0) and 3 µg/mL DNase I (AppliChem) as well as 5 mM dithiothreitol was added. Cell lysis was performed on ice via ultrasound (Digital Sonofier C25, Branson, MO, USA) with a maximum amplitude of 25% for three minutes and three subsequent repetitions. Cell debris were separated from cdHDAC4 containing lysate via centrifugation at 18,000× *g* for 30 min at 4 °C (6–16K centrifuge, Sigma). The lysate was filtrated with a 0.45 µm filter (Filtropur, Sarstedt, Nümbrecht, Germany), pooled, diluted to 100 mL with IMAC-Buffer A and 5 mM imidazole was added. First purification step was an IMAC chromatography. After equilibration of the IMAC column (5 mL cOmplete His-Tag Purification Resin, Roche, Basel, Switzerland) with IMAC-Buffer A containing 5 mM imidazole for a duration of 5 column volumes (CV) the lysate was loaded onto the IMAC column. Column wash was performed with IMAC-Buffer A containing 5 mM imidazole for a duration of 10 CV. The His6-tagged cdHDAC4 was eluted with IMAC-Buffer B containing 75 mM imidazole via step elution. The peak fractions were pooled, 6 µg/mL SUMO protease, as well as 5 mM dithiothreitol was added followed by an incubation overnight at 4 °C for cleaving of the SUMO-tag. The next day, the protein solution was diluted with an equally volume of 2× HIC buffer (4 M NaCl, 50 mM TRIS-HCl, pH 7.0). After equilibration of the HIC column (5 mL Toyopearl Butyl-650M, Tosoh Bioscience, Griesheim, Germany) with HIC Puffer containing 2 M NaCl, 50 mM TRIS-HCl, pH 7.0 for 5 CV the proteins solution was loaded onto the column. Column wash was performed with HIC Buffer for a duration of 10 CV. Elution was performed with HIC elution buffer containing 50 mM NaCl, 50 mM TRIS-HCl and pH 7.0 for a duration of 20 CV via step elution. The protein containing fractions were concentrated via ultrafiltration (Vivaspin 2, MWCO 3.5 kDa, Sartorius, Goettingen, Germany) at 8000 g and 4 °C (3–30KS centrifuge, Sigma, Osterode am Harz, Germany) to ca. 1.5 mL and 5 mM dithiothreitol was added. Last purification step was a size exclusion chromatography (SEC) using a (HiLoad^®®^ Superdex^®®^ 16/600 75 pg, Cytiva, Freiburg, Germany) with SEC-Buffer (150 mM KCl, 50 mM TRIS-HCl, pH 8.0. All chromatography steps were performed using a ÄKTA pure chromatography device (GE Healthcare Life Sciences, Freiburg, Germany). All flow rates were 5 mL/min, except for SEC which was 1 mL/min. To the purified cdHDAC4 25% glycerol and 1 mM TCEP tris(2-carboxyethyl)phosphine was added, flash frozen in liquid nitrogen and stored at −20 °C.

### 3.3. Determination of Michaelis Menten Parameters

For the determination of Michaelis Menten parameters a series of different substrate (Boc-Lys{TFA}-7-Amino-4-methylcoumarin) concentrations (200, 160, 120, 80, 60, 40, 20 and 10 µM) was mixed with 0.5 nM cdHDAC4_wt_ or 1 nM mutant variant protein in reaction Buffer (50 mM Tris, 137 mM NaCl, 2.7 mM KCl, 1 mM MgCl_2_, 1 mg/mL BSA, pH 8.0) at room temperature. For E677A, F812A, C813S, F871A and H976Y (GoF) 5 nM enzyme were used. Aliquots of 50 µL were removed at 0, 5, 10, 15, 20 and 30 min and transferred into 50 µL developer solution consisting of reaction buffer with the addition of 100 µM SATFMK and 20 mg/mL trypsin into the cavity of a black 96 well half area plate (Greiner, Kremsmuenster, Austria). Signal was developed for 30 min at room temperature and the fluorescence was measured at 450 nm (λ_Ex_ = 350 nm) in a PherStar Plus (BMG Labtech, Ortenberg, Germany) fluorescence plate reader. HDAC activity tracking throughout all applied enzyme activity assay was based on the work by Wegener et al. [31]. For the calculation of K_m_ the measured fluorescence at each time point and substrate concentration were corrected with the blank (0 min). Afterwards the resulting fluorescence units were calculated to the product concentration using an external AMC calibration with a slope of 5221 rfu/µM and corrected for the dilution by multiply the product concentration by two. Afterwards, the product concentration was plotted against the time and v_0_ (µM/min) was calculated from the resulting slopes. Now the initial velocity was plotted against the substrate concentration and K_m_ was calculated using a Michaelis Menten-Fit in GraphPad Prism. The value of v_max_ is neglected because enzyme concentration and purity are too inconsistent between the mutant variant to calculate this value appropriate.

### 3.4. Association Kinetics

A serial dilution of inhibitor beginning at 10-fold of respective IC_50_-value in assay buffer (25 mM Tris-HCl, pH 8.0, 75 mM KCl, 0.001% Pluronic F-127) was made in a black 96-well microtiter half-area plate (Greiner, Kremsmuenster, Austria). The dilutions were added to 3 nM cdHDAC4_wt_. Shortly after, the reaction was initiated by adding 20 µM Boc-Lys{TFA}-7-Amino-4-methylcoumarin (Bachem, Bubendorf, Switzerland) as substrate. The reaction was stopped after different incubation times: 2, 3, 4, 5, 7, 9, 12, 15 and 20 min with 500 µM SAHA. Finally 0.4 mg/mL trypsin was added to split the deacetylated substrate in the fluorescent product which was measured at 450 nm (λ_Ex_ = 350 nm) (PherStar Plus, BMG Labtech, Ortenberg, Germany). 15 compounds with highly structural diversity and IC_50_-values below 2 µM were tested. The standardized RFUs were plotted against the incubation time. The courses suggest the TZD ligands being slow binding inhibitors. Due to this knowledge the data were fitted via non linear regression with GraphPad Prism program to the following equation by Copeland [27]:

(2) $$Y=\nu_{s}\cdot t+\frac{\nu_{i}-\nu_{s}}{k_{\mathrm{obs}}}\cdot \left(1-e^{\left(-k_{\mathrm{obs}}\cdot t\right)}\right)+d$$

where *v_i_* is the initial slope, *v_s_* is the second lower slope, k_obs_ is the respective rate constant, *t* is time and *d* the correction for possible data offset. 

Afterwards k_obs_ values were plotted against their respective inhibitor concentration. Because all plots showed a saturation behavior as well as similar plateaus, the data was fitted via non linear regression with GraphPad Prism program to a two step model with the following equation by Copeland [27]:

(3) $$k_{\mathrm{obs}}=k_{\mathrm{off}}+\left(\frac{k_{\mathrm{on}}}{1+(K_{1}/I)}\right)$$

where *K*_1_ is the rapid equilibrium constant of an initial enzyme and inhibitor encounter complex k_on_ and k_off_ are the respective isomerization constants and *I* is the inhibitor concentration.

### 3.5. Reversibility Assay

The reversibility of all inhibitors was tested in 96-well microtiter half-area plate (Greiner) via rapid dilution using a modified enzyme activity assay according to Copeland [27]. 100 nM cdHDAC4_wt_ was incubated with respective TZD ligand (10-fold IC_50_-value) in assay buffer (25 mM Tris-HCl, pH 8.0, 75 mM KCl, 0.001% Pluronic F-127). Rapid dilution was performed by diluting the incubated mixture 100-fold with substrate (Boc-Lys{TFA}-7-Amino-4-methylcoumarin (Bachem, Bubendorf, Switzerland)) with the final substrate concentration being 20 µM. This caused the protein and the TZD ligand to be diluted down to 1 nM and 10% IC_50_-value, respectively. A positive control without TZD ligand and a blank without enzyme and TZD ligand were also carried out. A binding control was also determined: 10 nM cdHDAC4_wt_ and 100-fold IC_50_-value of respective TZD ligand was incubated and diluted 10 fold with substrate, resulting in a final enzyme and TZD ligand concentration of 1 nM and 10-fold IC_50_-value, respectively. Once again the final substrate concentration was 20 µM. The reactions were stopped after 15 min by the addition of 500 µM suberanilohydroxamic acid (Cayman Chemical Company, Ann Arbor, MI, USA). The deacetylated substrate was converted into a fluorescent product by the addition of 0.4 mg/mL trypsin (AppliChem, Darmstadt, Germany). The release of fluorogenic substrate was followed in a microplate reader (PherStar Plus, BMG Labtech, Ortenberg, Germany) at 450 nm (λ_Ex_ = 350 nm) and correlated the positive control. The data was analyzed with GraphPad Prism Version 6.01. All incubations steps were performed for 60 min at 30 °C and 450 rpm. The substrate was preheated to 30 °C before the rapid dilution step.

### 3.6. Calculations of Residence Time Based on Reversibility Data

Under the assumption of an exponential decay of the complex formed by cdHDAC4_wt_ and TZD ligand after rapid dilution the following equation for exponential decay was utilized [32]:

(4) $$A_{t}=A_{o}\cdot e^{\left(-k\cdot t\right)}$$

where *A_t_* in the concentration of the complex at a specific time, *A_o_* is the initial complex concentration, *k* is the decay constant and *t* is time. The reciprocal values for *k* equals the residence time of the respective TZD ligand (residence time = k_off_^−1^), which can be compared with the residence times gained from association data.

### 3.7. IC_50_ Determination

A serial dilution of TZD in assay buffer (25 mM TRIS-HCl, 75 mM KCl, 0.001% Pluronic F-127, pH 8.0) was incubated with 1 nM cdHDAC4wt in a black 96-well microtiter plate (Greiner). Afterwards the enzymatic reaction was initiated by the addition of 20 µM Boc-Lys{TFA}-7-Amino-4-methylcoumarin (Bachem, Bubendorf, Switzerland) as substrate. After incubation the enzymatic reaction was terminated by the addition of 1.7 µM SATFMK. The deacetylated substrate was converted into a fluorescent product by the addition of 0.4 mg/mL trypsin (AppliChem, Darmstadt, Germany). The release of fluorogenic substrate was followed in a microplate reader (PherStar Plus, BMG Labtech, Ortenberg, Germany) at 450 nm (λEx = 350 nm) and correlated to enzyme activity. GraphPad Prism program was used to generate dose response curves and were fitted to a four parameter logistic function to obtain IC_50_-values [33]:

(5) $$EA=E_{0}+\frac{\left(E_{max}-E_{0}\right)}{1+10^{\log\left({\mathrm{IC}}_{50}\right)-x\cdot h}}$$

in which *EA* is the enzyme activity for a given inhibitor concentration *x*, *E_max_* and *E*_0_ are the enzyme activities in the absence of inhibitor and at complete inhibition, respectively. *h* is the slope of the curve and IC_50_ is the inhibitor concentration at which half of the enzyme activity is inhibited. All incubations steps were performed for 60 min at 30 °C and 450 rpm.

### 3.8. Docking

Modeling, preparation and visualization of structural data as well as molecular docking was performed using MOE 2019 software (Chemical Computing Group ULC, Montreal, QC, Canada). Crystal structures of the closed and open conformation of the catalytic domain of HDAC4, PDB-ID’s 4CBY and 2VQJ, were obtained from RCSB Protein Data Bank and subjected to the Quickprep procedure of MOE 2019 including 3D-protonation for subsequent docking. The partial charges of all protein and ligand atoms were calculated using the implemented Amber14 force field. The docking site was defined by the ligand within the binding pocket of the respective crystal structure for HDAC4c (PDB-ID: 4CBY). This approach was not permissible for the open conformation of HDAC4 (PDB-ID:2VQJ), since the ligand covered only part of the significantly enlarged binding groove. In this special case, the binding site was analyzed using the Computed Atlas of Surface Topography of proteins (CASTp) (http://sts.bioe.uic.edu/castp/, accessed on 21 August 2021) [34]. The largest identified pocket with an estimated volume of 1019.7 Å^3^ was identical with the widely opened binding groove of HDAC4_o_. The flanking amino acids of this pocket (G36, R37, G331, G330, H198, H158, H159, F227, P156, P155, F168, S123, R154) were used to define the binding site of HDAC4_o_ for the subsequent docking procedure. Molecular docking was performed choosing the triangle matcher for placement of the ligand in the binding site and ranked with the London dG scoring function. The best 50 poses were passed to the refinement and energy minimization in the pocket using the induced fit method and the 10 best poses rescored using the GBVI/WSA dG scoring function. 

## 4. Conclusions

Very recently, we reported TZD ligands as inhibitors of HDAC4 lacking the problematic zinc binding group hydroxamic acid. However, the structural determinants of molecular recognition between TZD analogs and cdHDAC4_wt_ as well as the binding mechanism have not been elucidated until now. This study combines a large-scale SAR analysis of TZD ligands, extensive mutagenesis of HDAC4, Michaelis Menten, and binding kinetics as well as molecular docking to dissect the molecular interaction between TZD ligands and cdHDAC4_wt_ in molecular detail and advance the knowledge about HDAC inhibitors lacking the canonical hydroxamate zinc binding group. Potent TZD ligands are characterized by a terminal TZD moiety, a bulky hydrophobic linker such as naphthalene, and a hydrophobic CAP group. The mutational study and binding kinetics suggest that TZD compounds bind to the active site of cdHDAC4_wt,_ and are competitive and reversible inhibitors, which bind via a two-step or one-step binding mechanism depending on the CAP group. The residence time of **24g** is (34 ± 3) min and therefore 6 times larger than for the clinically approved pan-HDAC inhibitor SAHA (5 ± 2) min. Docking of TZD compounds into the catalytic domain of HDAC4 predicts the TZD group function as a warhead that coordinates to the catalytic zinc ion. Moreover, a comparison of binding constants from the mutational study with docking poses provides evidence that TZD inhibitors bind predominantly to the *closed* conformation of HDAC4 in solution. This is consistent with a conformational equilibrium of HDAC4, which is largely shifted to the *closed* form in the absence of an inhibitor. The predicted zinc binding property of the TZD group offers an alternative to the widely used hydroxamate group, which is found in by far the most known HDAC inhibitors and is suspected to have mutagenic effects. This is particularly relevant for drug application in chronic diseases. The slow two-step binding kinetics of TZD ligands to HDAC4 is consistent with an induced fit binding mechanism, which prolongs the residence time and is an important key parameter for the selection and development of safer active substances with long-lasting biological effects, particularly in indication areas such as cancer or anti-infective applications. 

In summary, TZD ligands with a suitable combination of linker and CAP group are selective inhibitors of HDAC4 and show slow two-step binding with prolonged residence time involving a conformational change. These beneficial features make the TZD ligands promising chemical starting points for the further development of drug candidates against cancer or Huntington’s Disease.

## Figures and Tables

**Table 2:** Docking scores of indicated compounds into HDAC4_c_ (PDB-ID 4CBY) and HDAC4_o_ (PDB-ID 2VQJ).

Title	?	GBVI/WSA dG	
Cluster #	Cpd	4CBY (Closed)	2VQJ (Open)
1	(S)-16b *	−9.2	−11.8
	(R)-16b	−8.8	−10.0
	(S)-8i *	−9.3	−10.2
	(R)-8i	−8.7	−10.0
	(S)-8b *	−9.1	−11.4
	(R)-8b	−8.6	−10.2
2	7l	−7.7	−9.4
	7n	−8.5	−9.5
	7s	−7.7	−8.3
3	(S)-12j	−8.8	−9.6
	(R)-12j *	−8.8	−9.9
	(S)-4d	−8.6	−9.6
	(R)-4d *	−9.1	−9.7
	(S)-4j	−8.5	−10.0
	(R)-4j *	−8.5	−10.0

The absolute configuration of compounds in cluster 1 and 3, which are highlighted by *, is identical.

## Data Availability

Data is contained within the article and Supplementary Material.

## Supplementary Materials

The following are available online at https://www.mdpi.com/article/10.3390/ph14101032/s1, Figure S1: Determination of Michaelis-Menten Paramters; Figure S2: Kinetic data plots of TZD ligands; Figure S3: Overlay of crystallized and redocked ligands; Figure S4: Regions of largest structural shifts for the transition from closed to open conformation of HDAC4; Figure S5: Overlay of HDAC4c and HDAC4o showing the amino acids that are mutated; Figure S6: The distance between Cβ-atoms is plotted versus the number of the mutated amino acids; Figure S7: Cluster analysis of most active TZD analogs; Figure S8: Overlap of docking poses of TZD analogs to (A–C) HDAC4c and (D–F) HDAC4o; Table S1: SMILES Strings and IC50-values of all tested TZD ligands; Table S2: IC50-values in µM for indicated TZD ligands towards cdHDAC4wt and corresponding mutants; Table S3: Open reading frame of cdHDAC4wt in pET14b for recombinant protein expression; Table S4: Mutant to cdHDAC4wt (WT) IC50 ratios.

## Abbreviations

AMC	7-amino-4-methylcoumarin
Boc	butoxycarbonyl
Cd	catalytic domain
HDAC4	human histone deacetylase 4
IC_50_	inhibitor concentration at 50% residual enzyme activity
Lys	lysine
NCoR	nuclear receptor co-repressor
RMSD	root mean square deviation
SAHA	suberoylanilide hydroxamic acid
SMRT	silencing mediator for retinoid or thyroid-hormone receptor
sZBD	structural zinc binding domain
TFA	trifluoroacetyl
TZD	1,3-thiazolidine-2,4-dione
Wt	wild type
ZBG	zinc binding group
