# Supplementary material for: Mechanistic Insights into Binding of Ligands with Thiazolidinedione Warhead to Human Histone Deacetylase 4
Source: Pharmaceuticals (Basel). 2021 Oct 11;14(10):1032. doi: 10.3390/ph14101032 (PMC8537711; doi:10.3390/ph14101032)
Supplement: Supplementary file 1 [file pharmaceuticals-14-01032-s001.zip › pharmaceuticals-1384956-supplementary.pdf]

Supplementary Materials:

Mechanistic Insights into Binding of Ligands with Thiazolidinedione warhead to human histone deacetylase 4

Markus Schweipert,[1‡] Niklas Jänsch,[1‡] Kalpana Tilekar, [2] Neha Upadhyay, [2*] Ewelina Wozny,[1] Sidra Basheer,[1] Eva Wurster,[1] Marlene Müller,[1] C. S. Ramaa, [2*] Franz-Josef Meyer-Almes[1*]*

[1]Department of Chemical Engineering and Biotechnology, University of Applied Sciences, 64295 Darmstadt, Germany

[2]Department of Pharmaceutical Chemistry, Bharati Vidyapeeth´s College of Pharmacy, Navi Mumbai, India

[‡]These Authors contributed equally

[*]Corresponding authors: franz-josef.meyer-almes@h-da.de, sinharamaa@yahoo.in

Table of contents:

- Tab. S1: SMILES Strings and IC_{50}-values of all tested TZD ligands, 2-9
- Fig. S1: Determination of Michaelis-Menten Paramters, 10
- Fig. S2: Kinetic data plots of TZD ligands, 11
- Tab. S2: IC_{50}-values in µM for indicated TZD ligands towards $cdHDAC4_{wt}$ and corresponding mutants 12
- Fig. S3: Overlay of crystallized and redocked ligands 13
- Fig. S4: Regions of largest structural shifts for the transition from *closed* to *open* conformation of HDAC4 14
- Fig. S5: Overlay of HDAC4c and HDAC4o showing the amino acids that are mutated 15
- Fig. S6: The distance between C_{β}-atoms is plotted versus the number of the mutated amino acids 16
- Fig. S7: Cluster analysis of most active TZD analogs 17
- Fig. S8: Overlap of docking poses of TZD analogs to A)-C) HDAC4c and D)-F) $HDAC4_o$ 18
- Tab. S3: Open reading frame of $cdHDAC4_{wt}$ in pET14b for recombinant protein expression 19
- Tab. S4: Mutant to $cdHDAC4_{wt}$ (WT) IC50 ratios 20-21
- Structural characterization of synthesized compounds (NMR, MS, FTIR, UV, HPLC) 22-268

Tab. S1: SMILES Strings and IC_{50}-values of all tested TZD ligands.

Ligand Name	IC50 / µM	Smiles Code
8b	0,33	O=C(COc1cc2ccc(/C=C(/C(N3)=O)\SC3=O)cc2cc1)N(C(C1)c(cccc2)c2Cl)N=C1c1ccccc1
8i	0,34	O=C(COc1ccc(cc(/C=C(/C(N2)=O)\SC2=O)cc2)c2c1)N(C(C1)c2cccs2)N=C1c1ccccc1
16b	0,42	O=C(COc1cc2ccc(/C=C(/C(N3)=O)\SC3=O)cc2cc1)N(C(C1)c(cccc2)c2Cl)N=C1c(cc1)ccc1F
16g	0,46	Cc1ccc(C(C2)N(C(COc3ccc(cc(/C=C(/C(N4)=O)\SC4=O)cc4)c4c3)=O)N=C2c(cc2)ccc2F)cc1
7l	0,54	Cc(cc(cc1)Br)c1NC(COc1cc2ccc(/C=C(/C(N3)=O)\SC3=O)cc2cc1)=O
4j	0,67	O=C(COc1ccc(/C=C(/C(N2)=O)\SC2=O)cc1)N(C(C1)c(cc2)cc(Cl)c2Cl)N=C1c1ccccc1
24g	0,71	Cc1ccc(C(C2)N(C(COc3ccc(cc(/C=C(/C(N4)=O)\SC4=O)cc4)c4c3)=O)N=C2c(ccc(F)c2)c2F)cc1
24e	0,73	O=C(COc1ccc(cc(/C=C(/C(N2)=O)\SC2=O)cc2)c2c1)N(C(C1)c(cc2)ccc2F)N=C1c(ccc(F)c1)c1F
8g	0,76	Cc1ccc(C(C2)N(C(COc3ccc(cc(/C=C(/C(N4)=O)\SC4=O)cc4)c4c3)=O)N=C2c2ccccc2)cc1
8a	0,77	O=C(COc1ccc(cc(/C=C(/C(N2)=O)\SC2=O)cc2)c2c1)N(C(C1)c2ccccc2)N=C1c1ccccc1
7s	0,78	O=C(COc1ccc(cc(/C=C(/C(N2)=O)\SC2=O)cc2)c2c1)Nc1ncccc1F
4d	0,79	O=C(COc1ccc(/C=C(/C(N2)=O)\SC2=O)cc1)N(C(C1)c2ccc(C(F)(F)F)cc2)N=C1c1ccccc1
12j	0,83	O=C(COc1ccc(/C=C(/C(N2)=O)\SC2=O)cc1)N(C(C1)c(cc2)cc(Cl)c2Cl)N=C1c(cc1)ccc1F
5w	0,90	O=C(COc1ncc(/C=C(/C(N2)=O)\SC2=O)cc1)Nc1nc(cccc2)c2s1

20e	1,0	O=C(COc1ccc(/C=C(/C(N2)=O)\SC2=O)cc1)N(C(C1)c(cc2)ccc2F)N=C1c(ccc(F)c1)c1F
8e	1,1	O=C(COc1ccc(cc(/C=C(/C(N2)=O)\SC2=O)cc2)c2c1)N(C(C1)c(cc2)ccc2F)N=C1c1ccccc1
16a	1,1	O=C(COc1ccc(cc(/C=C(/C(N2)=O)\SC2=O)cc2)c2c1)N(C(C1)c2ccccc2)N=C1c(cc1)ccc1F
8c	1,2	O=C(COc1ccc(cc(/C=C(/C(N2)=O)\SC2=O)cc2)c2c1)N(C(C1)c2ccco2)N=C1c1ccccc1
7x	1,2	Cc1cccc2c1nc(NC(COc1cc3ccc(/C=C(/C(N4)=O)\SC4=O)cc3cc1)=O)s2
16e	1,3	O=C(COc1ccc(cc(/C=C(/C(N2)=O)\SC2=O)cc2)c2c1)N(C(C1)c(cc2)ccc2F)N=C1c(cc1)ccc1F
7i	1,3	O=C(COc1cc2ccc(/C=C(/C(N3)=O)\SC3=O)cc2cc1)Nc(c(F)cc(F)c1)c1Br
4k	1,4	O=C(COc1ccc(/C=C(/C(N2)=O)\SC2=O)cc1)N(C(C1)c(cc2)ccc2Br)N=C1c1ccccc1
16c	1,4	O=C(COc1ccc(cc(/C=C(/C(N2)=O)\SC2=O)cc2)c2c1)N(C(C1)c2ccco2)N=C1c(cc1)ccc1F
22g	1,5	Cc1ccc(C(C2)N(C(COc3ccc(/C=C(/C(N4)=O)\SC4=O)cc3)=O)N=C2c(ccc(F)c2)c2F)cc1
12d	1,5	O=C(COc1ccc(/C=C(/C(N2)=O)\SC2=O)cc1)N(C(C1)c2ccc(C(F)(F)F)cc2)N=C1c(cc1)ccc1F
12a	1,6	O=C(COc1ccc(/C=C(/C(N2)=O)\SC2=O)cc1)N(C(C1)c2ccccc2)N=C1c(cc1)ccc1F
7w	1,6	O=C(COc1cc2ccc(/C=C(/C(N3)=O)\SC3=O)cc2cc1)Nc1nc(cccc2)c2s1
12f	1,6	O=C(COc1ccc(/C=C(/C(N2)=O)\SC2=O)cc1)N(C(C1)c(ccc(F)c2)c2F)N=C1c(cc1)ccc1F
4g	1,8	Cc1ccc(C(C2)N(C(COc3ccc(/C=C(/C(N4)=O)\SC4=O)cc3)=O)N=C2c2ccccc2)cc1
12g	1,8	Cc1ccc(C(C2)N(C(COc3ccc(/C=C(/C(N4)=O)\SC4=O)cc3)=O)N=C2c(cc2)ccc2F)cc1
7q	1,8	[O-][N+](c(cc1)ccc1NC(COc1ccc(cc(/C=C(/C(N2)=O)\SC2=O)cc2)c2c1)=O)=O
4b	1,9	O=C(COc1ccc(/C=C(/C(N2)=O)\SC2=O)cc1)N(C(C1)c(cccc2)c2Cl)N=C1c1ccccc1
22d	1,9	O=C(COc1ncc(/C=C(/C(N2)=O)\SC2=O)cc1)N(C(C1)c2ccc(C(F)(F)F)cc2)N=C1c(ccc(F)c1)c1F
5z	2,1	[O-][N+](c(cc1)cc2c1nc(NC(COc1ncc(/C=C(/C(N3)=O)\SC3=O)cc1)=O)s2)=O
14d	2,2	O=C(COc1ncc(/C=C(/C(N2)=O)\SC2=O)cc1)N(C(C1)c2ccc(C(F)(F)F)cc2)N=C1c(cc1)ccc1F
7g	2,2	COc1cccc(NC(COc2ccc(cc(/C=C(/C(N3)=O)\SC3=O)cc3)c3c2)=O)c1
12e	2,3	O=C(COc1ccc(/C=C(/C(N2)=O)\SC2=O)cc1)N(C(C1)c(cc2)ccc2F)N=C1c(cc1)ccc1F
14b	2,3	O=C(COc1ncc(/C=C(/C(N2)=O)\SC2=O)cc1)N(C(C1)c(cccc2)c2Cl)N=C1c(cc1)ccc1F
4f	2,5	O=C(COc1ccc(/C=C(/C(N2)=O)\SC2=O)cc1)N(C(C1)c(ccc(F)c2)c2F)N=C1c1ccccc1
7u	2,9	Cc(ccc(NC(COc1cc2ccc(/C=C(/C(N3)=O)\SC3=O)cc2cc1)=O)c1)c1Cl
4e	2,9	O=C(COc1ccc(/C=C(/C(N2)=O)\SC2=O)cc1)N(C(C1)c(cc2)ccc2F)N=C1c1ccccc1
7o	2,9	O=C(COc1cc2ccc(/C=C(/C(N3)=O)\SC3=O)cc2cc1)Nc(cc1)ccc1Br
7n	3,1	Cc1cccnc1NC(COc1ccc(cc(/C=C(/C(N2)=O)\SC2=O)cc2)c2c1)=O
22f	3,4	O=C(COc1ncc(/C=C(/C(N2)=O)\SC2=O)cc1)N(C(C1)c(ccc(F)c2)c2F)N=C1c(ccc(F)c1)c1F

5y	3,4	CCOc(cc1)cc2c1nc(NC(COc1ncc(/C=C(/C(N3)=O)\SC3=O)cc1)=O)s2
7p	3,5	O=C(COc1cc2ccc(/C=C(/C(N3)=O)\SC3=O)cc2cc1)Nc1cc(C(F)(F)F)cc(Cl)c1
7b	3,6	O=C(COc1ccc(cc(/C=C(/C(N2)=O)\SC2=O)cc2)c2c1)Nc(cc1)ccc1F
7y	3,6	CCOc(cc1)cc2c1nc(NC(COc1cc3ccc(/C=C(/C(N4)=O)\SC4=O)cc3cc1)=O)s2
14e	3,8	O=C(COc1ncc(/C=C(/C(N2)=O)\SC2=O)cc1)N(C(C1)c(cc2)ccc2F)N=C1c(cc1)ccc1F
7a	3,9	O=C(COc1ccc(cc(/C=C(/C(N2)=O)\SC2=O)cc2)c2c1)Nc1ccccc1
7j	4,0	O=C(COc1ccc(cc(/C=C(/C(N2)=O)\SC2=O)cc2)c2c1)Nc(cccc1)c1Oc1ccccc1
6e	4,2	O=C(COc1ncc(/C=C(/C(N2)=O)\SC2=O)cc1)N(C(C1)c(cc2)ccc2F)N=C1c1ccccc1
5x	4,3	Cc1cccc2c1nc(NC(COc1ncc(/C=C(/C(N3)=O)\SC3=O)cc1)=O)s2
6h	4,3	[O-][N+](c1cccc(C(C2)N(C(COc3ncc(/C=C(/C(N4)=O)\SC4=O)cc3)=O)N=C2c2ccccc2)c1)=O
7t	4,4	Cc1cc(NC(COc2ccc(cc(/C=C(/C(N3)=O)\SC3=O)cc3)c3c2)=O)no1
7c	4,5	Cc(cc1)ccc1NC(COc1ccc(cc(/C=C(/C(N2)=O)\SC2=O)cc2)c2c1)=O
7r	4,6	O=C(COc1ccc(cc(/C=C(/C(N2)=O)\SC2=O)cc2)c2c1)Nc(ccc(S)c1)c1F
14c	4,7	O=C(COc1ncc(/C=C(/C(N2)=O)\SC2=O)cc1)N(C(C1)c2ccco2)N=C1c(cc1)ccc1F
7e	4,8	O=C(COc1ccc(cc(/C=C(/C(N2)=O)\SC2=O)cc2)c2c1)Nc1cc(C(F)(F)F)ccc1
5o	5,0	O=C(COc1ncc(/C=C(/C(N2)=O)\SC2=O)cc1)Nc(cc1)ccc1Br
7v	5,2	O=C(COc1cc2ccc(/C=C(/C(N3)=O)\SC3=O)cc2cc1)Nc(cc1)cc(Br)c1Br
22e	5,2	O=C(COc1ncc(/C=C(/C(N2)=O)\SC2=O)cc1)N(C(C1)c(cc2)ccc2F)N=C1c(ccc(F)c1)c1F
6d	5,3	O=C(COc1ncc(/C=C(/C(N2)=O)\SC2=O)cc1)N(C(C1)c2ccc(C(F)(F)F)cc2)N=C1c1ccccc1
22b	5,6	O=C(COc1ncc(/C=C(/C(N2)=O)\SC2=O)cc1)N(C(C1)c(cccc2)c2Cl)N=C1c(ccc(F)c1)c1F
6a	5,7	O=C(COc1ncc(/C=C(/C(N2)=O)\SC2=O)cc1)N(C(C1)c2ccccc2)N=C1c1ccccc1
5h	5,7	O=C(COc1ncc(/C=C(/C(N2)=O)\SC2=O)cc1)Nc(cc1)cc(Cl)c1Cl
14f	5,8	O=C(COc1ncc(/C=C(/C(N2)=O)\SC2=O)cc1)N(C(C1)c(ccc(F)c2)c2F)N=C1c(cc1)ccc1F
6i	5,9	O=C(COc1ncc(/C=C(/C(N2)=O)\SC2=O)cc1)N(C(C1)c2cccs2)N=C1c1ccccc1
7h	6,2	O=C(COc1cc2ccc(/C=C(/C(N3)=O)\SC3=O)cc2cc1)Nc(cc1)cc(Cl)c1Cl
6b	6,3	O=C(COc1ncc(/C=C(/C(N2)=O)\SC2=O)cc1)N(C(C1)c(cccc2)c2Cl)N=C1c1ccccc1
5v	7,0	O=C(COc1ncc(/C=C(/C(N2)=O)\SC2=O)cc1)Nc(cc1)cc(Br)c1Br
12c	7,0	O=C(COc1ccc(/C=C(/C(N2)=O)\SC2=O)cc1)N(C(C1)c2ccco2)N=C1c(cc1)ccc1F
12b	8,0	O=C(COc1ccc(/C=C(/C(N2)=O)\SC2=O)cc1)N(C(C1)c(cccc2)c2Cl)N=C1c(cc1)ccc1F
22c	8,1	O=C(COc1ncc(/C=C(/C(N2)=O)\SC2=O)cc1)N(C(C1)c2ccco2)N=C1c(ccc(F)c1)c1F
5u	8,4	Cc1cc(Cl)cc(NC(COc2ncc(/C=C(/C(N3)=O)\SC3=O)cc2)=O)c1

7k	8,4	O=C(COc1ccc(cc(/C=C(\C(N2)=O)/SC2=O)cc2)c2c1)Nc(ccc(F)c1)c1F
6f	8,9	O=C(COc1ncc(/C=C(/C(N2)=O)\SC2=O)cc1)N(C(C1)c(ccc(F)c2)c2F)N=C1c1ccccc1
7f	9,4	O=C(COc1cc2ccc(/C=C(/C(N3)=O)\SC3=O)cc2cc1)Nc1cccc(Cl)c1
5f	11	O=C(COc1ncc(/C=C(/C(N2)=O)\SC2=O)cc1)Nc1cccc(Cl)c1
4a	12	O=C(COc1ccc(/C=C(/C(N2)=O)\SC2=O)cc1)N(C(C1)c2ccccc2)N=C1c1ccccc1
5c	12	Cc(cc1)ccc1NC(COc1ncc(/C=C(/C(N2)=O)\SC2=O)cc1)=O
5e	13	O=C(COc1ncc(/C=C(/C(N2)=O)\SC2=O)cc1)Nc1cc(C(F)(F)F)ccc1
3k	15	[O-][N+](c(cc1)ccc1NC(COc1c(/C=C(/C(N2)=O)\SC2=O)c2ccccc2cc1)=O)=O
20c	15	O=C(COc1ccc(/C=C(/C(N2)=O)\SC2=O)cc1)N(C(C1)c2ccco2)N=C1c(ccc(F)c1)c1F
5r	15	O=C(COc1ncc(/C=C(/C(N2)=O)\SC2=O)cc1)Nc(ccc(Br)c1)c1F
5k	19	O=C(COc1ncc(/C=C(/C(N2)=O)\SC2=O)cc1)Nc(ccc(F)c1)c1F
5s	19	O=C(COc1ncc(/C=C(/C(N2)=O)\SC2=O)cc1)Nc1ncccc1F
7m	20	O=C(COc1ccc(cc(/C=C(/C(N2)=O)\SC2=O)cc2)c2c1)Nc1ncccc1
14a	21	O=C(COc1ncc(/C=C(/C(N2)=O)\SC2=O)cc1)N(C(C1)c2ccccc2)N=C1c(cc1)ccc1F
5n	21	Cc1cccnc1NC(COc1ncc(/C=C(/C(N2)=O)\SC2=O)cc1)=O
5m	22	O=C(COc1ncc(/C=C(/C(N2)=O)\SC2=O)cc1)Nc1ncccc1
5d	25	COc(cc1)ccc1NC(COc1ncc(/C=C(/C(N2)=O)\SC2=O)cc1)=O
5p	34	O=C(COc1ncc(/C=C(/C(N2)=O)\SC2=O)cc1)Nc(c(C(F)(F)F)ccc1)c1Cl
GB24	35	Cc(c(C)c1)cc2c1sc(NC(CN(C(/C(/S1)=C/c(cc3)ccc3Cl)=O)C1=O)=O)n2
P10	43	CC(N(C)N(C1=O)c2ccccc2)=C1NC(CN(C(/C(/S1)=C/c2ncccc2)=O)C1=O)=O
3a	> 50	O=C(COc1c(/C=C(/C(N2)=O)\SC2=O)c2ccccc2cc1)Nc1ccccc1
3b	> 50	O=C(COc1c(/C=C(/C(N2)=O)\SC2=O)c2ccccc2cc1)Nc(cc1)ccc1F
3c	> 50	Cc(cc1)ccc1NC(COc1c(/C=C(/C(N2)=O)\SC2=O)c2ccccc2cc1)=O
3d	> 50	COc(cc1)ccc1NC(COc1c(/C=C(/C(N2)=O)\SC2=O)c2ccccc2cc1)=O
3e	> 50	O=C(COc1c(/C=C(/C(N2)=O)\SC2=O)c2ccccc2cc1)Nc(cc1)cc(Cl)c1Cl
3f	> 50	O=C(COc1c(/C=C(/C(N2)=O)\SC2=O)c2ccccc2cc1)Nc(c(F)cc(F)c1)c1Br
3g	> 50	O=C(COc1c(/C=C(/C(N2)=O)\SC2=O)c2ccccc2cc1)Nc(ccc(F)c1)c1F
3h	> 50	Cc(cc(cc1)Br)c1NC(COc1c(/C=C(/C(N2)=O)\SC2=O)c2ccccc2cc1)=O
3i	> 50	O=C(COc1c(/C=C(/C(N2)=O)\SC2=O)c2ccccc2cc1)Nc(cc1)ccc1Br
3j	> 50	O=C(COc1c(/C=C(/C(N2)=O)\SC2=O)c2ccccc2cc1)Nc1cc(C(F)(F)F)ccc1
5a	> 50	O=C(COc1ncc(/C=C(/C(N2)=O)\SC2=O)cc1)Nc1ccccc1

5b	> 50	O=C(COc1ncc(/C=C(/C(N2)=O)\SC2=O)cc1)Nc(cc1)ccc1F
5g	> 50	COc1cccc(NC(COc2ncc(/C=C(/C(N3)=O)\SC3=O)cc2)=O)c1
5i	> 50	O=C(COc1ncc(/C=C(/C(N2)=O)\SC2=O)cc1)Nc(c(F)cc(F)c1)c1Br
5j	> 50	Cc1ncc(NC(COc2ncc(/C=C(/C(N3)=O)\SC3=O)cc2)=O)s1
5l	> 50	Cc(cc(cc1)Br)c1NC(COc1ncc(/C=C(/C(N2)=O)\SC2=O)cc1)=O
5q	> 50	[O-][N+](c(cc1)ccc1NC(COc1ncc(/C=C(/C(N2)=O)\SC2=O)cc1)=O)=O
5t	> 50	Cc1cc(NC(COc2ncc(/C=C(/C(N3)=O)\SC3=O)cc2)=O)no1
7d	> 50	COc(cc1)ccc1NC(COc1ccc(cc(/C=C(/C(N2)=O)\SC2=O)cc2)c2c1)=O
F1	> 50	O=C(CN(C(/C(/S1)=C/c2ccco2)=O)C1=O)Nc1ccccc1
F10	> 50	O=C(CN(C(/C(/S1)=C/c2ccco2)=O)C1=O)Nc(cc(C(F)(F)F)cc1)c1Cl
F11	> 50	O=C(CN(C(/C(/S1)=C/c2ccco2)=O)C1=O)Nc(ccc(Br)c1)c1F
F12	> 50	COc(cc1)ccc1NC(CN(C(/C(/S1)=C/c2ccco2)=O)C1=O)=O
F13	> 50	O=C(CN(C(/C(/S1)=C/c2ccco2)=O)C1=O)Nc(cc1)ccc1F
F14	> 50	[O-][N+](c(cc1)ccc1NC(CN(C(/C(/S1)=C/c2ccco2)=O)C1=O)=O)=O
F15	> 50	Cc1cc(NC(CN(C(/C(/S2)=C/c3ccco3)=O)C2=O)=O)no1
F16	> 50	O=C(CN(C(/C(/S1)=C/c2ccco2)=O)C1=O)Nc1nccs1
F17	> 50	Cc(ccc(NC(CN(C(/C(/S1)=C/c2ccco2)=O)C1=O)=O)c1)c1Cl
F18	> 50	O=C(CN(C(/C(/S1)=C/c2ccco2)=O)C1=O)Nc1nc(cccc2)c2s1
F19	> 50	O=C(CN(C(/C(/S1)=C/c2ccco2)=O)C1=O)Nc(cc1)cc(Cl)c1Cl
F2	> 50	O=C(CN(C(/C(/S1)=C/c2ccco2)=O)C1=O)Nc(cccc1)c1F
F21	> 50	O=C(CN(C(/C(/S1)=C/c2ccco2)=O)C1=O)Nc(cc1)ccc1Br
F22	> 50	O=C(CN(C(/C(/S1)=C/c2ccco2)=O)C1=O)Nc1ncccc1
F23	> 50	Cc(cc1)ccc1NC(CN(C(/C(/S1)=C/c2ccco2)=O)C1=O)=O
F24	> 50	O=C(CN(C(/C(/S1)=C/c2ccco2)=O)C1=O)Nc(cc1)cc(Br)c1Br
F25	> 50	Cc1csc(NC(CN(C(/C(/S2)=C/c3ccco3)=O)C2=O)=O)n1
F3	> 50	O=C(CN(C(/C(/S1)=C/c2ccco2)=O)C1=O)Nc1cc(F)ccc1
F4	> 50	O=C(CN(C(/C(/S1)=C/c2ccco2)=O)C1=O)Nc(c(F)cc(Br)c1)c1F
F5	> 50	O=C(CN(C(/C(/S1)=C/c2ccco2)=O)C1=O)Nc(ccc(F)c1)c1F
F6	> 50	O=C(CN(C(/C(/S1)=C/c2ccco2)=O)C1=O)Nc1cc(C(F)(F)F)ccc1
F7	> 50	O=C(CN(C(/C(/S1)=C/c2ccco2)=O)C1=O)Nc(cc1)c(C(F)(F)F)cc1Cl
F8	> 50	O=C(CN(C(/C(/S1)=C/c2ccco2)=O)C1=O)Nc(cc1)c(C(F)(F)F)cc1Cl

F9	> 50	O=C(CN(C(/C(/S1)=C/c2ccco2)=O)C1=O)Nc(cc1)ccc1Oc1ccccc1
G1	> 50	O=C(CN(C(/C(/S1)=C/c(cc2)ccc2Br)=O)C1=O)Nc(cc1)c(C(F)(F)F)cc1Cl
G10	> 50	O=C(CN(C(/C(/S1)=C/c(cc2)ccc2F)=O)C1=O)Nc(ccc(F)c1)c1F
G11	> 50	Cc1ccc(/C=C(/C(N2CC(Nc(cc3)c(C(F)(F)F)cc3Cl)=O)=O)\SC2=O)cc1
G12	> 50	Cc1ccc(/C=C(/C(N2CC(Nc(ccc(F)c3)c3F)=O)=O)\SC2=O)cc1
G13	> 50	O=C(CN(C(/C(/S1)=C/c2ccccc2)=O)C1=O)Nc(cc1)c(C(F)(F)F)cc1Cl
G14	> 50	O=C(CN(C(/C(/S1)=C/c2ccccc2)=O)C1=O)Nc(ccc(F)c1)c1F
G15	> 50	O=C(CN(C(/C(/S1)=C/c(ccc(F)c2)c2F)=O)C1=O)Nc1cc(C(F)(F)F)ccc1
G16	> 50	O=C(CN(C(/C(/S1)=C/c(cc2)ccc2F)=O)C1=O)Nc1cc(C(F)(F)F)ccc1
G2	> 50	O=C(CN(C(/C(/S1)=C/c(cc2)ccc2Br)=O)C1=O)Nc(ccc(F)c1)c1F
G3	> 50	Cc1c(C)cc(/C=C(/C(N2CC(Nc(cc3)c(C(F)(F)F)cc3Cl)=O)=O)\SC2=O)cc1
G4	> 50	Cc1c(C)cc(/C=C(/C(N2CC(Nc(ccc(F)c3)c3F)=O)=O)\SC2=O)cc1
G5	> 50	O=C(CN(C(/C(/S1)=C/c(cc2)ccc2Cl)=O)C1=O)Nc(cc1)c(C(F)(F)F)cc1Cl
G6	> 50	O=C(CN(C(/C(/S1)=C/c(cc2)ccc2Cl)=O)C1=O)Nc(ccc(F)c1)c1F
G7	> 50	O=C(CN(C(/C(/S1)=C/c(ccc(F)c2)c2F)=O)C1=O)Nc(cc1)c(C(F)(F)F)cc1Cl
G8	> 50	O=C(CN(C(/C(/S1)=C/c(ccc(F)c2)c2F)=O)C1=O)Nc(ccc(F)c1)c1F
G9	> 50	O=C(CN(C(/C(/S1)=C/c(cc2)ccc2F)=O)C1=O)Nc(cc1)c(C(F)(F)F)cc1Cl
GB01	> 50	O=C(CN(C(/C(/S1)=C/c2ccccc2)=O)C1=O)Nc(sc1ccc2)nc1c2Cl
GB02	> 50	Cc1ccc(/C=C(/C(N2CC(Nc(sc3ccc4)nc3c4Cl)=O)=O)\SC2=O)cc1
GB03	> 50	O=C(CN(C(/C(/S1)=C/c(cc2)ccc2Cl)=O)C1=O)Nc(sc1ccc2)nc1c2Cl
GB04	> 50	O=C(CN(C(/C(/S1)=C/c(cc2)ccc2Br)=O)C1=O)Nc1nc(c(Cl)ccc2)c2s1
GB05	> 50	O=C(CN(C(/C(/S1)=C/c(ccc(F)c2)c2F)=O)C1=O)Nc(sc1ccc2)nc1c2Cl
GB06	> 50	Cc1c(C)cc(/C=C(/C(N2CC(Nc(sc3ccc4)nc3c4Cl)=O)=O)\SC2=O)cc1
GB07	> 50	O=C(CN(C(/C(/S1)=C/c(cc2)ccc2Br)=O)C1=O)Nc1nc(c(F)cc(F)c2)c2s1
GB08	> 50	COc1cccc2c1nc(NC(CN(C(/C(/S1)=C/c3ccccc3)=O)C1=O)=O)s2
GB09	> 50	Cc1cccc2c1nc(NC(CN(C(/C(/S1)=C/c3ccccc3)=O)C1=O)=O)s2
GB10	> 50	Cc1cccc2c1nc(NC(CN(C(/C(/S1)=C/c(cc3)ccc3F)=O)C1=O)=O)s2
GB11	> 50	O=C(CN(C(/C(/S1)=C/c(cc2)ccc2Cl)=O)C1=O)Nc1nc(c(F)cc(F)c2)c2s1
GB12	> 50	O=C(CN(C(/C(/S1)=C/c2ccccc2)=O)C1=O)Nc1nc(c(F)cc(F)c2)c2s1
GB13	> 50	Cc(cc1)cc2c1nc(NC(CN(C(/C(/S1)=C/c(ccc(F)c3)c3F)=O)C1=O)=O)s2
GB14	> 50	Cc1ccc(/C=C(/C(N2CC(Nc3nc(ccc(C)c4)c4s3)=O)=O)\SC2=O)cc1

GB15	> 50	Cc(cc1)cc2c1nc(NC(CN(C(/C(/S1)=C/c3cc(C)c(C)cc3)=O)C1=O)=O)s2
GB16	> 50	Cc(cc1)cc2c1nc(NC(CN(C(/C(/S1)=C/c(cc3)ccc3Cl)=O)C1=O)=O)s2
GB17	> 50	CCOc(cc1)cc2c1nc(NC(CN(C(/C(/S1)=C/c(cc3)ccc3Cl)=O)C1=O)=O)s2
GB18	> 50	O=C(CN(C(/C(/S1)=C/c(cc2)ccc2F)=O)C1=O)Nc1nc(c(F)cc(F)c2)c2s1
GB19	> 50	Cc1ccc(/C=C(/C(N2CC(Nc3nc(c(F)cc(F)c4)c4s3)=O)=O)\SC2=O)cc1
GB20	> 50	Cc1c(C)cc(/C=C(/C(N2CC(Nc3nc(c(F)cc(F)c4)c4s3)=O)=O)\SC2=O)cc1
GB21	> 50	Cc1ccc(/C=C(/C(N2CC(Nc3nc(cc(C)c(C)c4)c4s3)=O)=O)\SC2=O)cc1
GB23	> 50	Cc(c(C)c1)cc2c1sc(NC(CN(C(/C(/S1)=C/c(cc3)ccc3Br)=O)C1=O)=O)n2
GB25	> 50	Cc(c(C)c1)cc2c1sc(NC(CN(C(/C(/S1)=C/c(cc3)ccc3F)=O)C1=O)=O)n2
GB26	> 50	Cc1c(C)cc(/C=C(/C(N2CC(Nc3nc(cc(C)c(C)c4)c4s3)=O)=O)\SC2=O)cc1
GB27	> 50	Cc1ccc(/C=C(/C(N2CC(Nc3nc(c(OC)ccc4)c4s3)=O)=O)\SC2=O)cc1
GB28	> 50	Cc1cccc2c1nc(NC(CN(C(/C(/S1)=C/c3ccccc3)=O)C1=O)=O)s2
GB29	> 50	O=C(CN(C(/C(/S1)=C/c(ccc(F)c2)c2F)=O)C1=O)Nc1nc(c(F)cc(F)c2)c2s1
GB30	> 50	O=C(CN(C(/C(/S1)=C/c2ccccc2)=O)C1=O)Nc1nc(c(F)ccc2)c2s1
GB31	> 50	Cc(cc1)cc2c1nc(NC(CN(C(/C(/S1)=C/c(cc3)ccc3Br)=O)C1=O)=O)s2
GB32	> 50	Cc1ccc(/C=C(/C(N2CC(Nc3nc(ccc(F)c4)c4s3)=O)=O)\SC2=O)cc1
GB33	> 50	O=C(CN(C(/C(/S1)=C/c(cc2)ccc2Cl)=O)C1=O)Nc1nc(ccc(F)c2)c2s1
GB34	> 50	O=C(CN(C(/C(/S1)=C/c(cc2)ccc2F)=O)C1=O)Nc1nc(ccc(F)c2)c2s1
GB35	> 50	COc1cccc2c1nc(NC(CN(C(/C(/S1)=C/c(cc3)ccc3F)=O)C1=O)=O)s2
GB36	> 50	CCOc(cc1)cc2c1nc(NC(CN(C(/C(/S1)=C/c3ccccc3)=O)C1=O)=O)s2
P1	> 50	O=C(CN(C(/C(/S1)=C/c2ncccc2)=O)C1=O)Nc1ccccc1
P11	> 50	O=C(CN(C(/C(/S1)=C/c2ncccc2)=O)C1=O)Nc(cc1)ccc1F
P12	> 50	Cc(cc1)ccc1NC(CN(C(/C(/S1)=C/c2ncccc2)=O)C1=O)=O
P13	> 50	O=C(CN(C(/C(/S1)=C/c2ncccc2)=O)C1=O)Nc(cccc1)c1Oc1ccccc1
P14	> 50	COc(cc1)ccc1NC(CN(C(/C(/S1)=C/c2ncccc2)=O)C1=O)=O
P15	> 50	O=C(CN(C(/C(/S1)=C/c2ncccc2)=O)C1=O)Nc(c(F)cc(Br)c1)c1F
P16	> 50	[O-][N+](c(cc1)ccc1NC(CN(C(/C(/S1)=C/c2ncccc2)=O)C1=O)=O)=O
P17	> 50	Cc(ccc(NC(CN(C(/C(/S1)=C/c2ncccc2)=O)C1=O)=O)c1)c1Cl
P18	> 50	Cc1conc1NC(CN(C(/C(/S1)=C/c2ncccc2)=O)C1=O)=O
P19	> 50	O=C(CN(C(/C(/S1)=C/c2ncccc2)=O)C1=O)Nc1nc(cccc2)c2s1
P2	> 50	O=C(CN(C(/C(/S1)=C/c2ncccc2)=O)C1=O)Nc(cccc1)c1F

P20	> 50	O=C(CN(C(/C(/S1)=C/c2ncccc2)=O)C1=O)Nc1nccs1
P21	> 50	Cc1cnc(NC(CN(C(/C(/S2)=C/c3ncccc3)=O)C2=O)=O)s1
P22	> 50	O=C(CN(C(/C(/S1)=C/c2ncccc2)=O)C1=O)Nc(cc1)ccc1Br
P23	> 50	O=C(CN(C(/C(/S1)=C/c2ncccc2)=O)C1=O)Nc1ncccc1
P24	> 50	COc1cccc(NC(CN(C(/C(/S2)=C/c3ncccc3)=O)C2=O)=O)c1
P25	> 50	O=C(CN(C(/C(/S1)=C/c2ncccc2)=O)C1=O)Nc(cc1)cc(Br)c1Br
P3	> 50	O=C(CN(C(/C(/S1)=C/c2ncccc2)=O)C1=O)Nc1cc(F)ccc1
P4	> 50	O=C(CN(C(/C(/S1)=C/c2ncccc2)=O)C1=O)Nc(ccc(Br)c1)c1F
P5	> 50	O=C(CN(C(/C(/S1)=C/c2ncccc2)=O)C1=O)Nc(ccc(F)c1)c1F
P6	> 50	O=C(CN(C(/C(/S1)=C/c2ncccc2)=O)C1=O)Nc1cc(C(F)(F)F)ccc1
P7	> 50	O=C(CN(C(/C(/S1)=C/c2ncccc2)=O)C1=O)Nc(cc1)c(C(F)(F)F)cc1Cl
P8	> 50	O=C(CN(C(/C(/S1)=C/c2ncccc2)=O)C1=O)Nc(cc1)cc(Cl)c1Cl
P9	> 50	O=C(CN(C(/C(/S1)=C/c2ncccc2)=O)C1=O)Nc(cc(C(F)(F)F)cc1)c1Cl
PB01	> 50	[O-][N+](c(cc1)cc2c1nc(NC(CN(C(/C(/S1)=C/c3ncccc3)=O)C1=O)=O)s2)=O
PB02	> 50	Cc1cccc2c1nc(NC(CN(C(/C(/S1)=C/c3ncccc3)=O)C1=O)=O)s2
PB05	> 50	O=C(CN(C(/C(/S1)=C/c2ncccc2)=O)C1=O)Nc1nc(cc(c(F)c2)F)c2s1
PB07	> 50	COc1cccc2c1nc(NC(CN(C(/C(/S1)=C/c3ncccc3)=O)C1=O)=O)s2
PB08	> 50	CCOc(cc1)cc2c1nc(NC(CN(C(/C(/S1)=C/c3ncccc3)=O)C1=O)=O)s2
PB09	> 50	O=C(CN(C(/C(/S1)=C/c2ncccc2)=O)C1=O)Nc1nc(ccc(F)c2)c2s1
F20	> 50	CC(N(C)N(C1=O)c2ccccc2)=C1NC(CN(C(/C(/S1)=C/c2ccco2)=O)C1=O)=O
GB22	> 50	Cc(c(C)c1)cc2c1sc(NC(CN(C(/C(/S1)=C/c3ccccc3)=O)C1=O)=O)n2
PB03	> 50	Cc(cc1)cc2c1nc(NC(CN(C(/C(/S1)=C/c3ncccc3)=O)C1=O)=O)s2
PB04	> 50	O=C(CN(C(/C(/S1)=C/c2ncccc2)=O)C1=O)Nc(sc1ccc2)nc1c2Cl
PB06	> 50	Cc(c(C)c1)cc2c1sc(NC(CN(C(/C(/S1)=C/c3ncccc3)=O)C1=O)=O)n2
SAHA	40	ONC(CCCCCCC(Nc1ccccc1)=O)=O

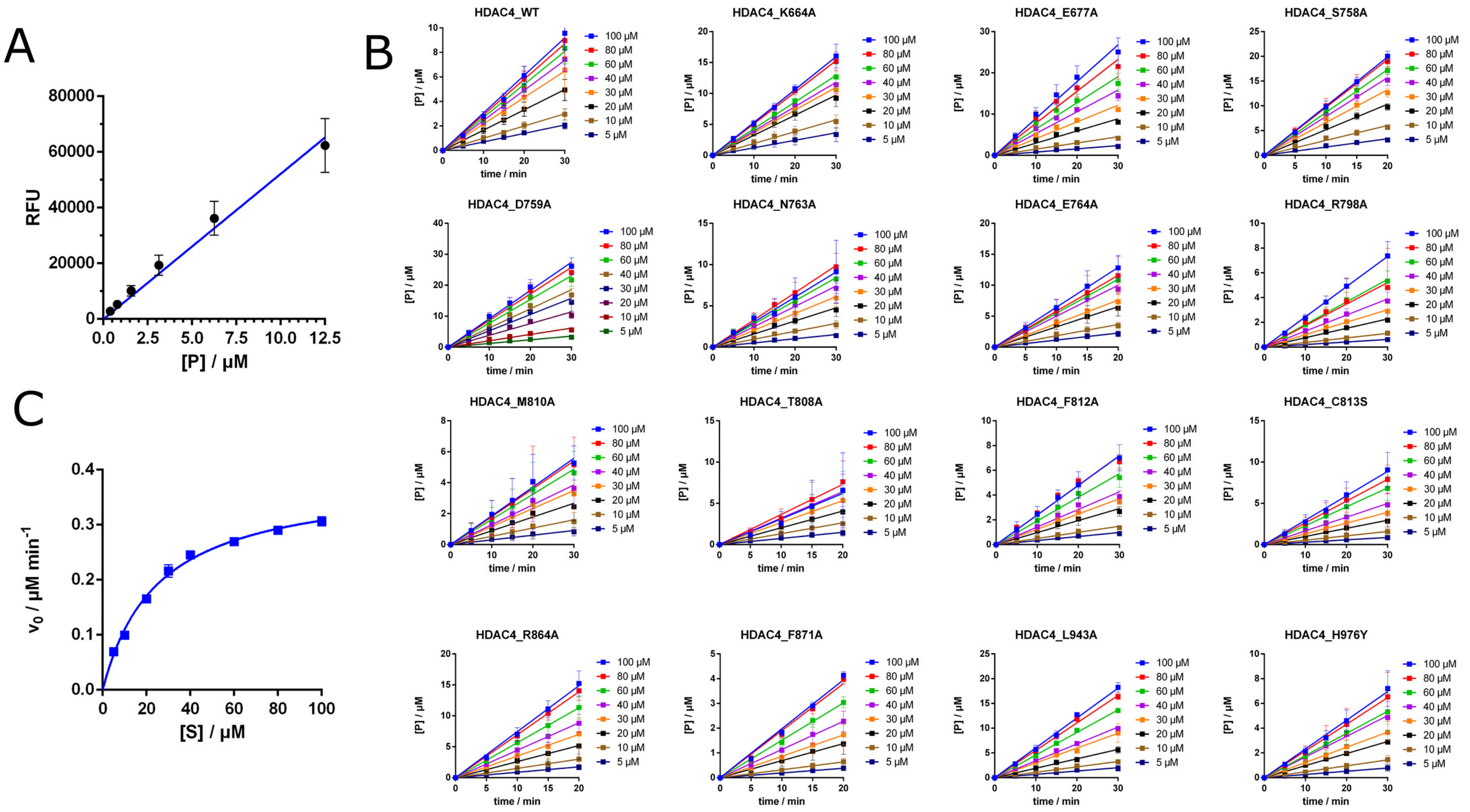

Fig. S2: Determination of Michaelis-Menten Paramters. (A) Calibration curve for the artificial substrate Boc-Lys{TFA}-AMC. A serial dilution of 7-Amino-4-methycoumarin was prepared and the slope of linear regression was calculated in RFU/µM**. (B)** Time progress curves for the HDAC4 wildtype and mutant variants with varying substrate concentrations. Y-axis was converted from RFU to product concentration using the slope of calibration from experiment **A**. Afterwards, the slope was calculated in µM/min which is the initial velocity of substrate conversion v_o. **(C)** Finally v_0 was plotted against substrate concentration for each HDAC4 variant. K_m was calculated using a Michaelis-Menten fit in GraphPad Prism.

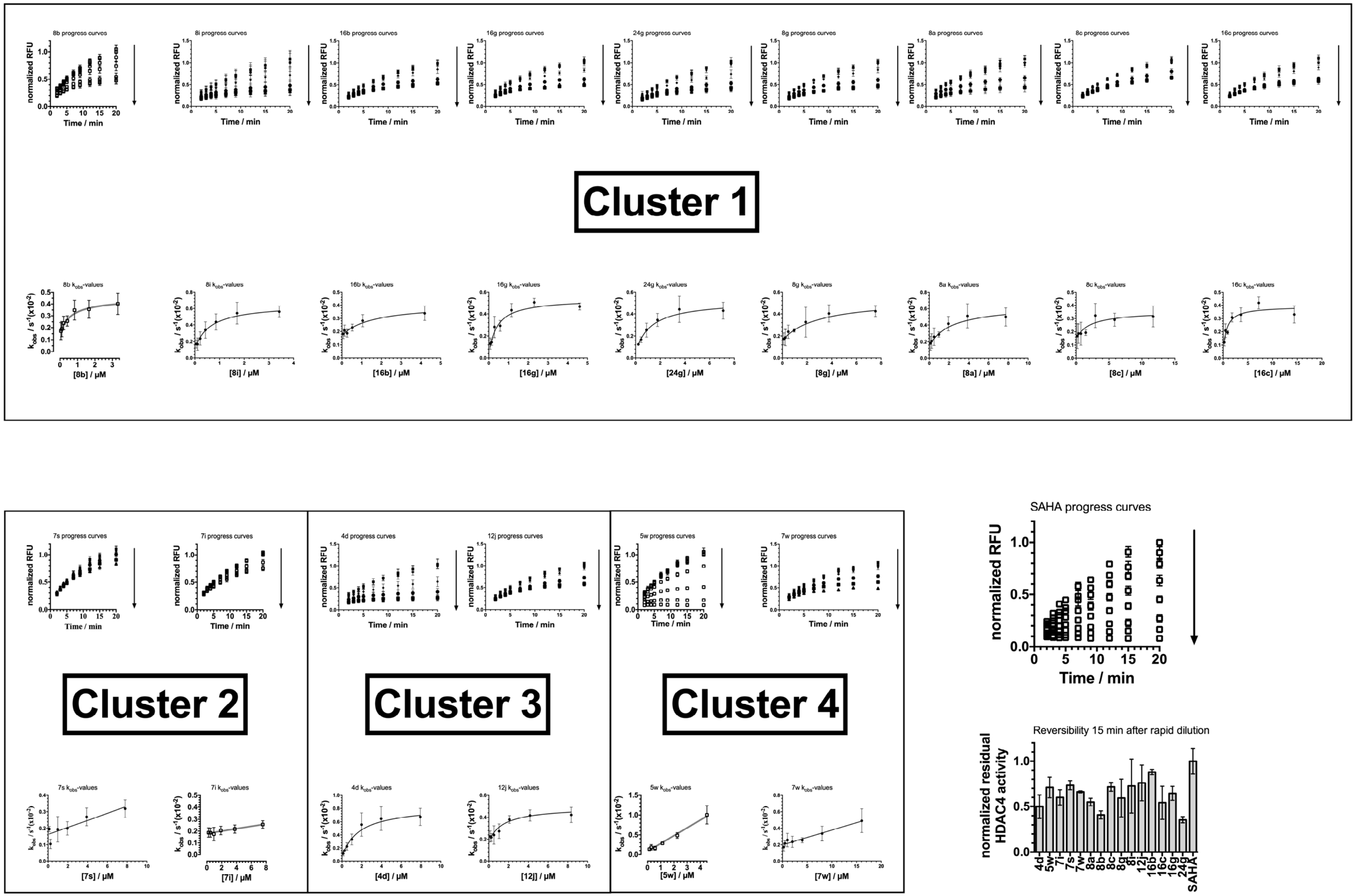

Fig. S3: Kinetic data plots of TZD ligands. Compound identifiers are displayed in the respective figures. Arrows indicate increasing TZD ligand concentration. Shown data represent means and standard deviations, N=3. Zoomable, high resolution pucture (1200 dpi).

Tab. S4: IC$_{50}$-values in µM for indicated TZD ligands towards cdHDAC4$_{wt}$ and corresponding mutants .

TZD ligand	WT	S758A	F871A	T808A	R864A	D759A	M810A	F812A	C813S	L943A	H976Y	K664A	E764A	N763A	R798A	E677A
**12d**	1,25	8,63	5,86	6,66	17,84	2,40	5,07	31,22	3,23	>50	1,64	3,30	>50	0,91	7,40	1,05
**12e**	1,13	35,01	38,64	29,43	12,38	5,81	6,28	>50	3,97	>50	4,79	12,41	>50	1,26	8,10	1,35
**12j**	1,42	28,85	14,61	16,76	8,01	4,84	15,99	28,85	8,83	44,92	6,21	2,91	>50	4,83	4,04	4,19
**14d**	1,66	20,54	26,93	34,53	11,16	10,17	7,58	49,09	3,69	>50	9,44	5,96	>50	1,87	1,95	0,85
**16a**	1,06	32,44	9,98	10,63	10,33	2,33	8,67	>50	25,31	>50	15,35	17,31	>50	2,84	14,31	2,01
**16b**	0,47	14,50	5,48	5,31	21,63	1,24	28,24	>50	4,01	28,74	29,17	2,39	>50	5,65	5,33	0,84
**16c**	1,66	26,25	7,61	10,42	11,61	3,14	7,78	>50	3,95	>50	26,42	6,53	>50	1,70	2,47	0,88
**16e**	0,78	16,16	6,19	7,59	29,73	4,27	20,11	>50	25,43	>50	7,46	27,85	>50	20,02	29,69	>50
**16g**	2,04	11,91	6,34	7,70	11,09	2,09	9,29	>50	4,61	>50	15,10	2,96	>50	1,41	2,91	2,24
**20e**	1,23	43,60	11,10	10,97	32,78	3,33	5,95	46,73	3,94	>50	3,47	4,19	>50	0,77	1,73	2,42
**24e**	0,69	41,36	3,85	13,85	20,07	2,38	7,45	>50	4,11	>50	28,45	7,15	>50	2,12	5,85	0,58
**24g**	1,18	6,19	4,04	4,30	9,82	1,61	8,47	>50	3,87	>50	6,49	3,27	>50	2,62	3,84	1,43
**4d**	0,49	5,02	2,43	4,44	8,61	1,75	4,23	43,61	6,95	>50	4,03	3,99	>50	0,68	2,26	0,76
**4j**	0,41	10,48	9,12	14,32	6,12	2,08	13,27	28,15	14,11	>50	2,79	5,50	>50	4,39	4,15	4,27
**4k**	1,17	23,60	9,29	4,20	4,15	2,26	8,81	48,59	3,97	>50	4,12	3,67	>50	0,72	4,14	0,41
**5w**	0,78	3,04	8,66	2,02	7,36	1,93	4,43	25,47	4,04	>50	3,60	4,92	33,24	1,81	7,20	11,00
**7b**	0,73	21,37	>50	28,98	29,25	6,73	13,77	34,75	26,32	>50	8,53	8,56	>50	5,31	7,38	>50
**7i**	1,04	31,05	4,90	38,94	28,93	1,99	19,85	>50	23,95	>50	26,72	37,15	>50	6,10	2,77	4,55
**7l**	0,47	16,16	2,55	11,70	28,93	2,17	20,07	>50	23,07	>50	26,72	6,89	>50	3,54	2,77	3,26
**7n**	0,45	26,53	10,53	29,76	29,31	1,61	4,95	>50	9,24	>50	25,27	7,42	>50	4,67	2,40	4,29
**7s**	1,41	20,26	34,79	18,55	16,47	6,42	9,25	>50	26,44	>50	27,38	8,49	49,16	12,83	6,29	>50
**7w**	0,47	22,36	13,69	34,02	6,41	0,63	21,39	>50	>50	>50	0,44	4,68	>50	2,99	3,58	8,00
**8a**	0,90	>50	32,23	30,02	39,30	6,00	26,58	>50	25,33	>50	15,61	32,61	>50	9,80	21,24	>50
**8b**	0,44	3,21	3,55	1,93	5,16	1,09	4,27	25,85	2,50	>50	9,00	2,51	33,51	1,74	3,91	2,96
**8c**	0,51	>50	5,40	33,57	11,29	4,22	9,22	>50	4,03	37,56	26,77	6,36	>50	1,41	1,46	2,89
**8e**	0,54	11,03	1,99	4,21	29,49	1,50	7,39	>50	6,55	>50	11,52	5,72	>50	1,26	7,55	1,17
**8g**	0,22	13,07	4,99	11,89	11,60	1,97	6,37	>50	4,69	>50	2,84	5,03	>50	1,18	5,23	2,11
**8i**	0,17	3,15	3,50	2,90	6,93	0,91	2,88	40,56	25,73	>50	1,93	2,34	>50	1,03	1,04	11,81

DOCKING

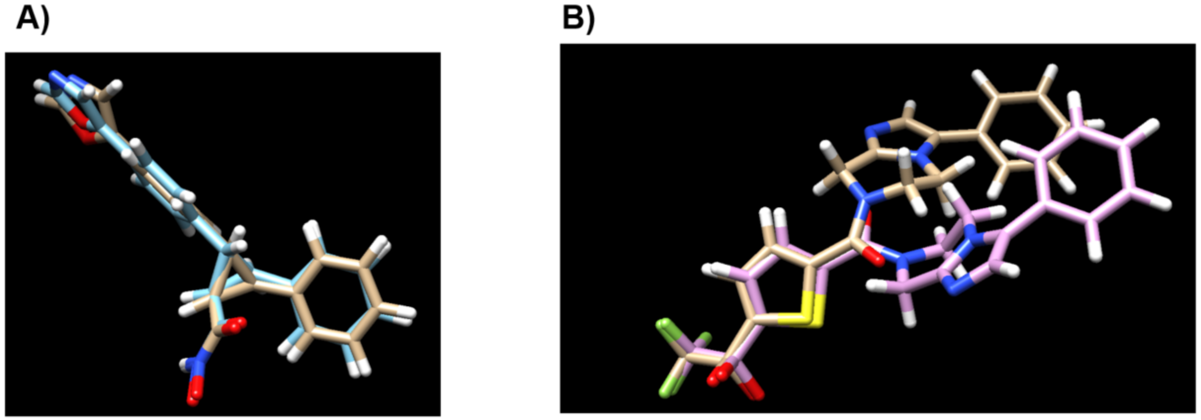

Fig. S5: Overlay of crystallized and redocked ligands in complex with A) HDAC4$_c$ (PDB-ID: 4CBY) and B) HDAC4$_o$ (PDB-ID: 2VQJ).

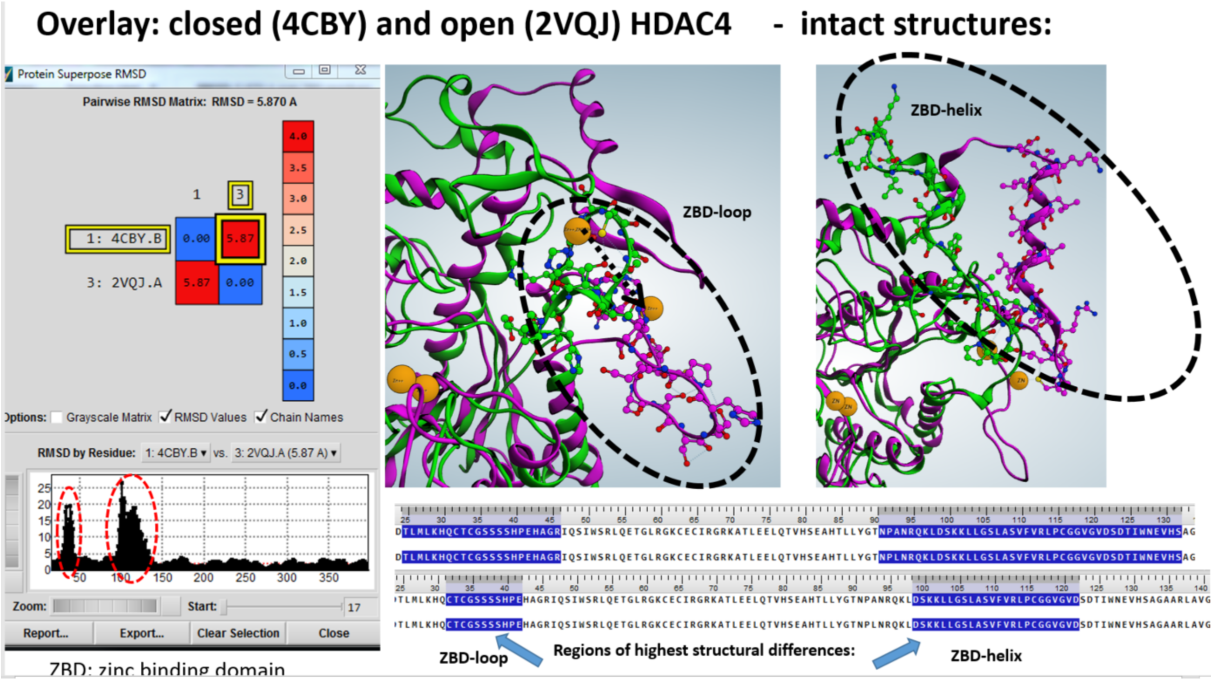

Fig. S6: Regions of largest structural shifts for the transition from *closed* to *open* conformation of HDAC4.

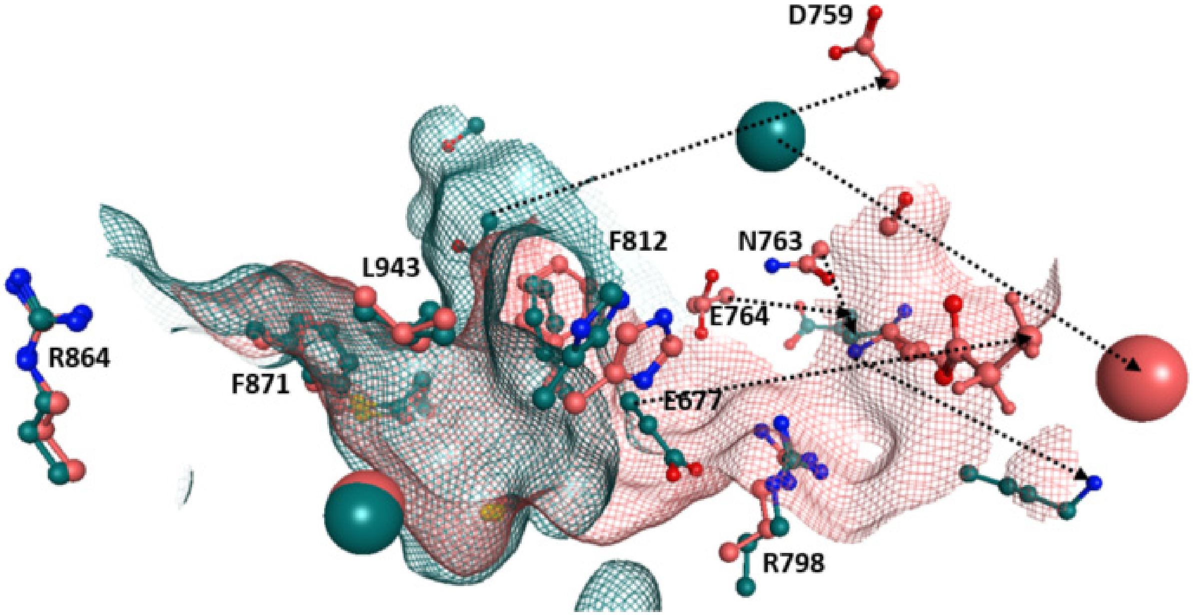

Fig. S7: Overlay of HDAC4c (4CBY, dark cyan) and HDAC4o (2VQJ, dark pink) showing the amino acids that are mutated in this study. Larger shifts upon the transition from open to closed conformation are indicated by dotted black arrows. The bigger beads represent the catalytic and structural zinc ions.

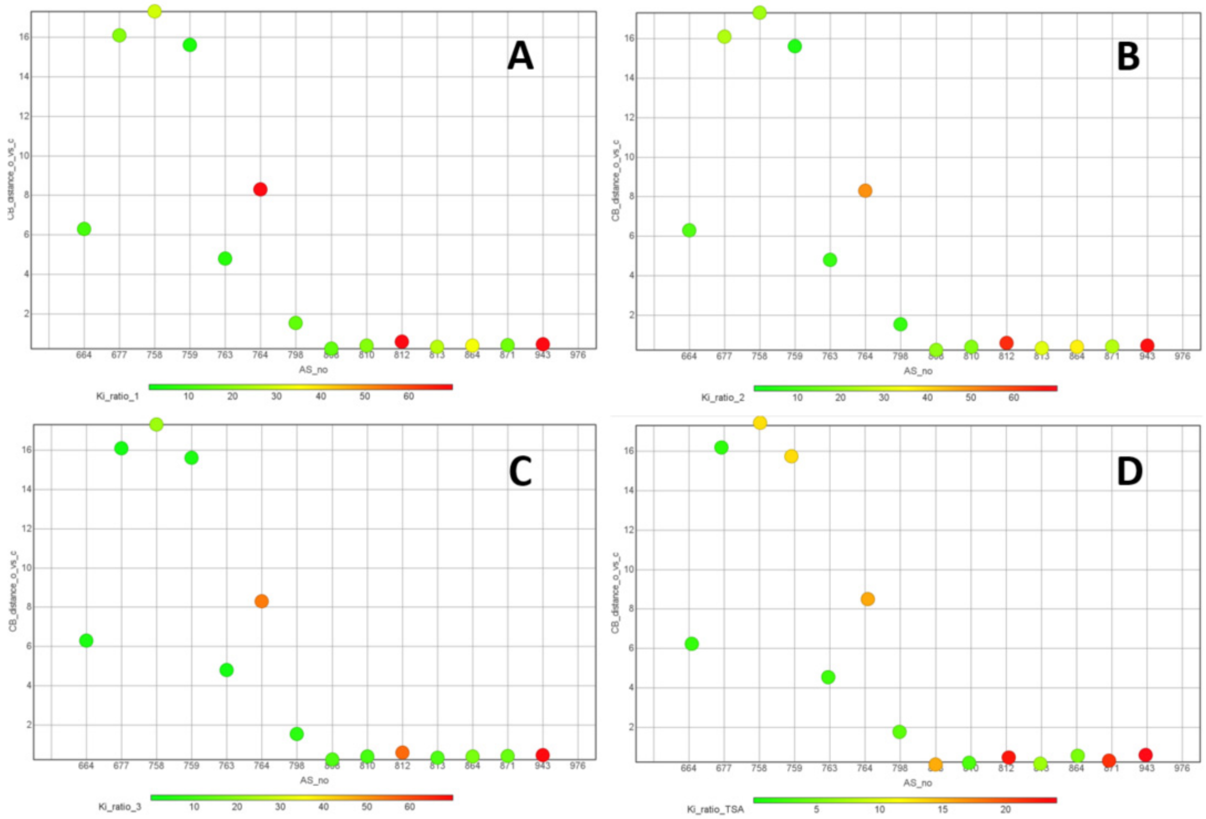

Fig. S8: The distance between C_{β}-atoms is plotted versus the number of the mutated amino acids. Coloring is according to the Ki-rato for docking poses in A) cluster 1, B) cluster 2, C) cluster 3 and D) TSA.

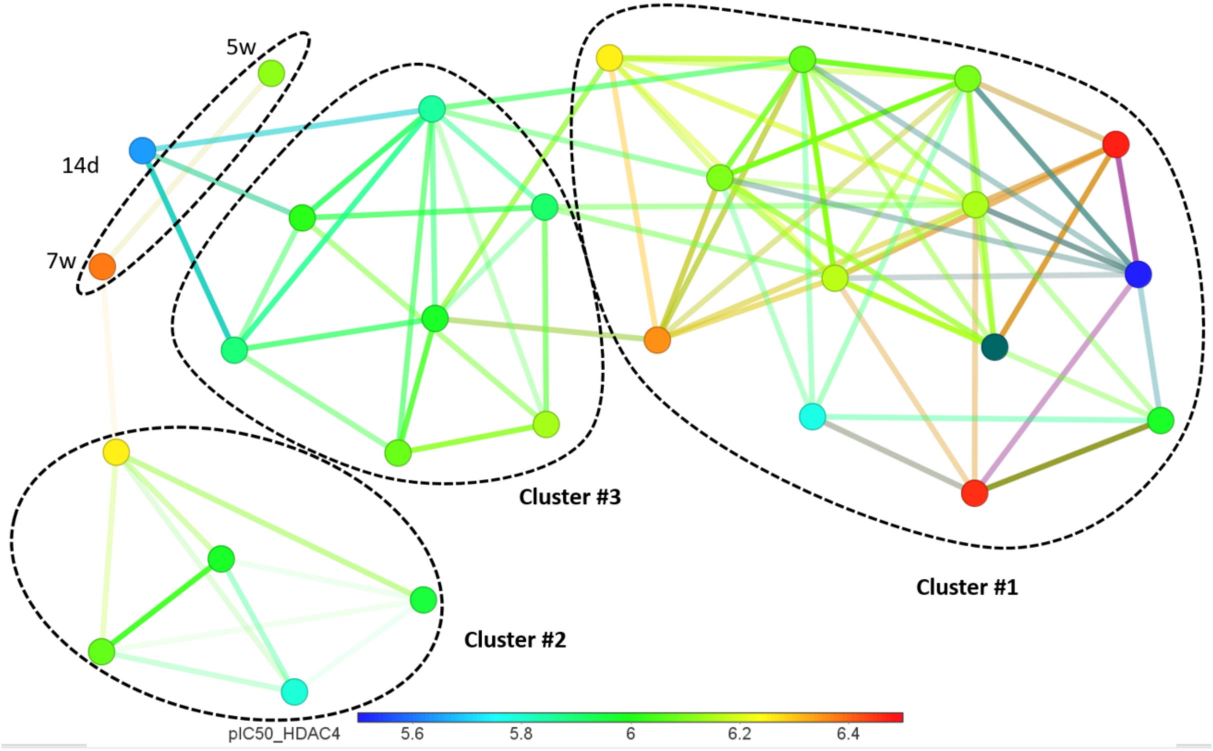

Fig. S9: Cluster analysis of most active TZD analogs. Each dot represents one compound. The dots are colored according to experimental pIC50 against cdHDAC4$_{wt}$. Structurally nearest neighbours are connecte by lines.

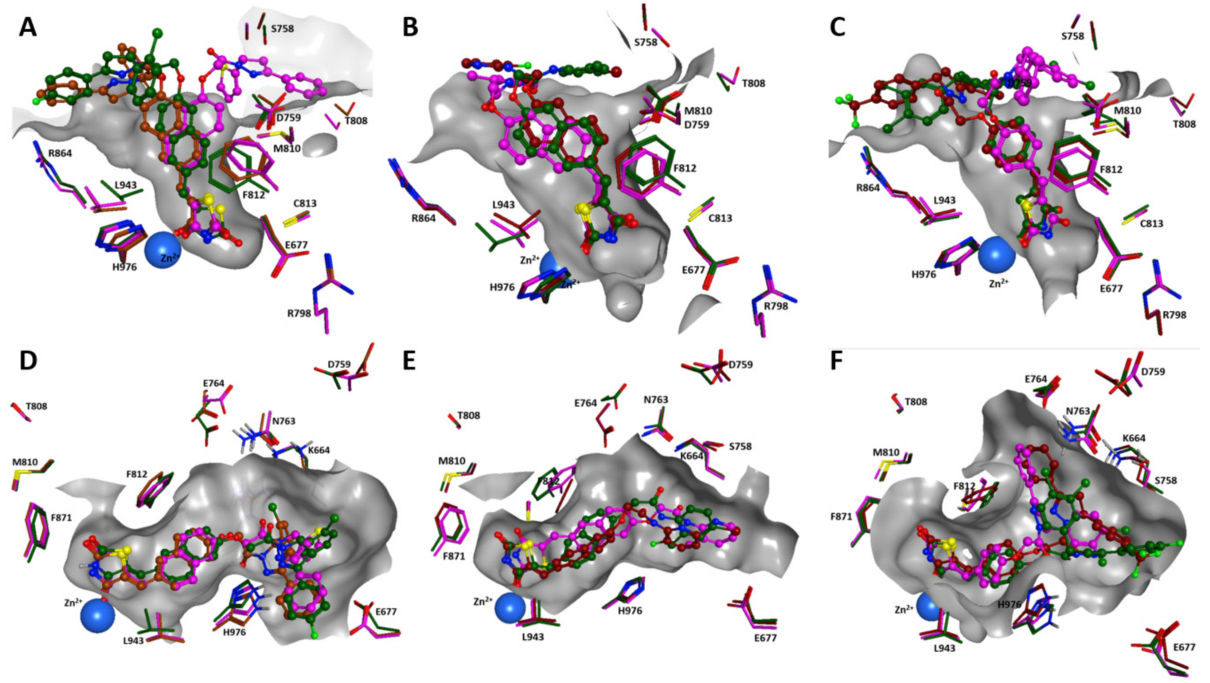

Fig. S10: Overlap of docking poses of TZD analogs to A)-C) HDAC4$_c$ (PDB-ID: 4CBY) and D)-F) HDAC4$_o$ (PDB-ID: 2VQJ). Docking poses of cluster 1 containing **(S)-16b** [green], **(S)-8i** [magenta] and **(S)-8b** [brown] are shown in A and D, cluster 2 containing **7l** [green], **7n** [magenta] and **7s** [brown] in B and E, and cluster 3 containing **(R)-12j** [green], **(R)-4d** [brown] and **(R)-4j** [magenta] are shown in C and F. The catalytic zinc ion at the bottom of the binding pocket is colored blue and the surface of the pocket light gray.

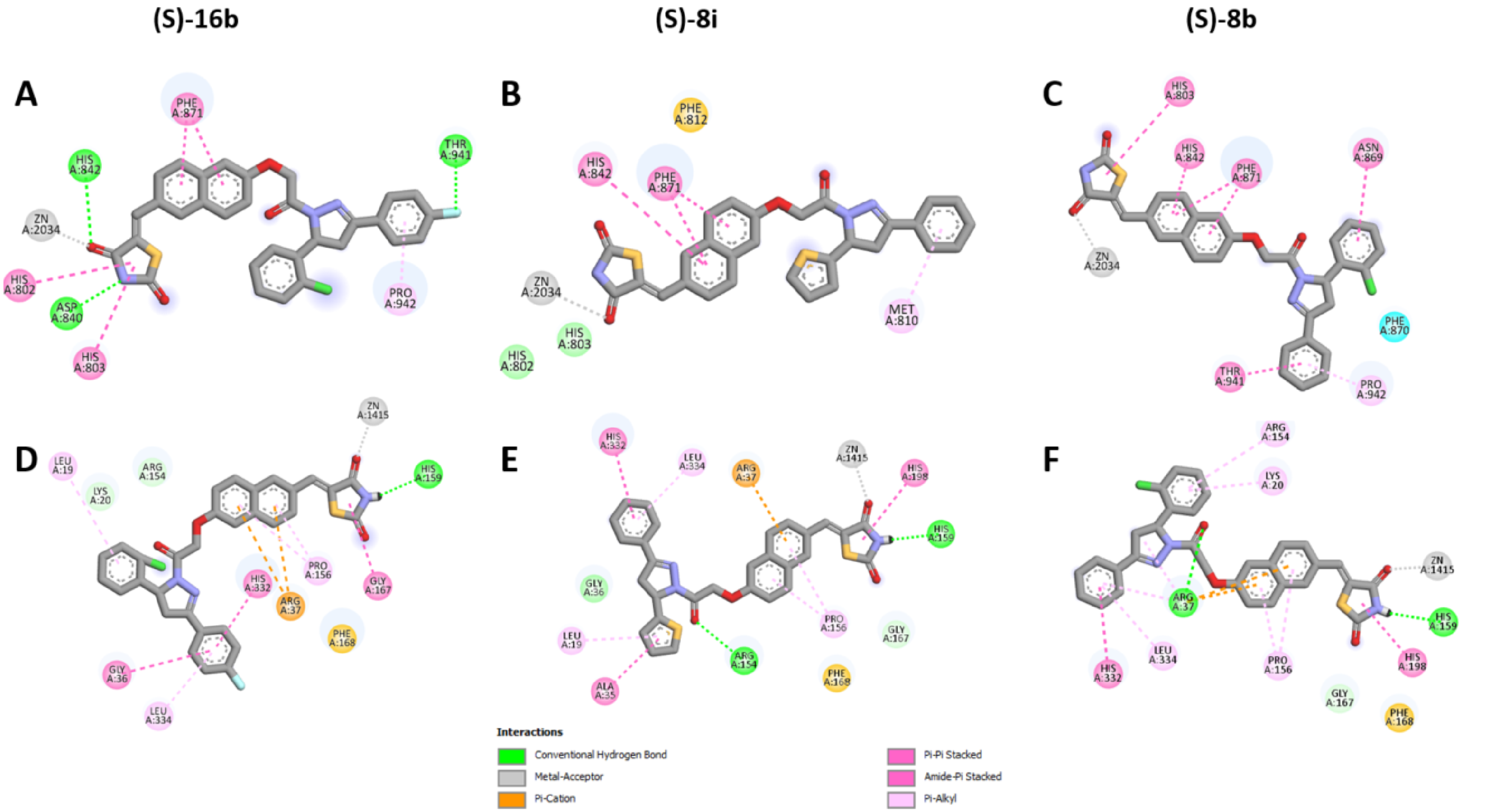

Fig. S11: 2D ligand interactions of (S)-16b, (S)-8i and (S)-8b in the binding pocket of A)-C) HDAC4$_c$ (PDB-ID: 4CBY) and D)-F) HDAC4$_o$ (PDB-ID: 2VQJ).

Tab. S11: Open reading frame of cdHDAC4$_{wt}$ in pET14b for recombinant protein expression.

ATGGGCAGCAGCCATCATCATCATCATCACGGTACCATGTCGGACTCAGAAGTCAATCAAGAAGCTAAGCCAGAGGTCAAGCCAGAAGTCAAGCCTGAGACTCACATCAATTTAA
AGGTGTCCGATGGATCTTCAGAGATCTTCTTCAAGATCAAAAAGACCACTCCTTTAAGAAGGCTGATGGAAGCGTTCGCTAAAAGACAGGGTAAGGAAATGGACTCCTTAAGATT
CTTGTACGACGGTATTAGAATTCAAGCTGATCAGACCCCTGAAGATTTGGACATGGAGGATAACGATATTATTGAGGCTCACAGAGAACAGATTGGTGGTCATATGACCAAACCG
CGCTTTACGACGGGTCTGGTCTACGACACGCTGATGCTGAAACACCAATGCACCTGTGGCTCAAGCTCCTCACATCCGGAACATGCAGGCCGCATTCAGTCGATCTGGAGCCGTCT
GCAAGAAACCGGCCTGCGCGGTAAATGCGAATGTATTCGTGGCCGCAAAGCCACCCTGGAAGAACTGCAGACGGTGCATTCCGAAGCACACACCCTGCTGTATGGTACGAACCC
GCTGAATCGTCAAAAACTGGATAGTAAAAAACTGCTGGGCTCTCTGGCGAGTGTCTTTGTGCGTCTGCCGTGCGGTGGTGTTGGTGTCGATTCTGACACCATCTGGAACGAAGTG
CACAGTGCAGGTGCAGCACGTCTGGCAGTTGGTTGTGTGGTTGAACTGGTCTTTAAAGTGGCGACCGGCGAACTGAAAAACGGTTTCGCAGTCGTGCGTCCGCCGGGTCATCACG
CAGAAGAATCGACGCCGATGGGTTTTTGCTATTTCAATAGCGTGGCCGTTGCAGCTAAACTGCTGCAGCAACGTCTGAGTGTTTCCAAAATTCTGATCGTTGATTGGGACGTCCAT
CACGGCAACGGTACCCAGCAAGCGTTCTACTCCGATCCGTCAGTGCTGTATATGTCTCTGCATCGCTACGATGACGGCAACTTTTTCCCGGGCAGTGGTGCACCGGATGAAGTTGG
TACCGGTCCGGGTGTGGGTTTTAACGTTAATATGGCTTTCACGGGCGGTCTGGACCCGCCGATGGGTGACGCGGAATATCTGGCGGCCTTTCGTACCGTTGTCATGCCGATTGCAA
GCGAATTTGCTCCGGATGTGGTTCTGGTTAGCTCTGGCTTTGACGCAGTCGAAGGTCATCCGACCCCGCTGGGCGGTTATAACCTGAGCGCACGTTGCTTTGGCTACCTGACGAAA
CAGCTGATGGGTCTGGCAGGCGGTCGCATTGTGCTGGCTCTGGAAGGCGGTCACGATCTGACCGCTATCTGCGACGCATCGGAAGCATGTGTCAGCGCACTGCTGGGTAATGAA
CTGGACCCGCTGCCGGAAAAAGTTCTGCAGCAACGTCCGAACGCAAATGCTGTCCGCTCCATGGAAAAAGTGATGGAAATCCACTCAAAATACTGGCGTTGTCTGCAGCGCACCA
CGTCTACGGCAGGTCGCAGTCTGATTGAAGCGCAAACGTGTGAAAACGAAGAAGCCGAAACCGTGACGGAGCTCAGCGCATGGAGCCATCCGCAGTTTGAAAAATGA

START

His_6-SUMO

cHDAC4$_{WT}$

Strep(II)-tag

STOP

Tab. S12: Mutant to cdHDAC4$_{wt}$ (WT) IC50 ratios. Mutant HDAC4 variants were generated using splicing by overlap extension PCR (SOE-PCR) with the following primers and cloned into a pET14b vector (Novagen, EMD Millipore) consists of a n-terminal His6-SUMO-tag and c-terminal Strep(II)-tag with NdeI and BamHI restriction enzymes.

Primer	5'-sequence-3'
K664A_for	ACGCTGATGCTGGCTCACCAATGCACC
K664A_rev	GGTGCATTGGTGAGCCAGCATCAGCGT
E677A_for	TCCTCACATCCGGCTCATGCAGGCCGC
E677A_rev	GCGGCCTGCATGAGCCGGATGTGAGGA
S758A_for	GTTGGTGTCGATGCTGACACCATCTGG
S758A_rev	CCAGATGGTGTCAGCATCGACACCAAC
D759A_for	GGTGTCGATTCTGCTACCATCTGGAAC
D759A_rev	GTTCCAGATGGTAGCAGAATCGACACC
N763A_for	GACACCATCTGGGCTGAAGTGCACAGT
N763A_rev	ACTGTGCACTTCAGCCCAGATGGTGTC
E764A_for	ACCATCTGGAACGCTGTGCACAGTGCA
E764A_rev	TGCACTGTGCACAGCGTTCCAGATGGT
R798A_for	TTCGCAGTCGTGGCTCCGCCGGGTCAT
R798A_rev	ATGACCCGGCGGAGCCACGACTGCGAA
T808A_for	GCAGAAGAATCGGCTCCGATGGGTTTT
T808A_rev	AAAACCCATCGGAGCCGATTCTTCTGC
M810A_for	GAATCGACGCCGGCTGGTTTTTGCTAT
M810A_rev	ATAGCAAAAACCAGCCGGCGTCGATTC
F812A_for	ACGCCGATGGGTGCTTGCTATTTCAAT
F812A_rev	ATTGAAATAGCAAGCACCCATCGGCGT
C813S_for	ACGCCGATGGGTTTTTCTTATTTCAATAGCGTG
C813S_rev	CACGCTATTGAAATAAGAAAAACCCATCGGCGT
R864A_for	ATGTCTCTGCATGCTTACGATGACGGC
R864A_rev	GCCGTCATCGTAAGCATGCAGAGACAT
F871A_for	GACGGCAACTTTGCTCCGGGCAGTGGT

F871A_rev	ACCACTGCCCGGAGCAAAGTTGCCGTC
L943A_for	CATCCGACCCCGGCTGGCGGTTATAAC
L943A_rev	GTTATAACCGCCAGCCGGGGTCGGATG
H976Y_for	GCTCTGGAAGGCGGTTATGATCTGACCGCTATC
H976Y_rev	GATAGCGGTCAGATCATAACCGCCTTCCAGAGC

Structural characterization

2-(2,4-dioxo-5-(pyridin-2-ylmethylene)thiazolidin-3-yl)-N-(6-nitrobenzo[d]thiazol-2-yl)acetamide (PB1)

1. FTIR

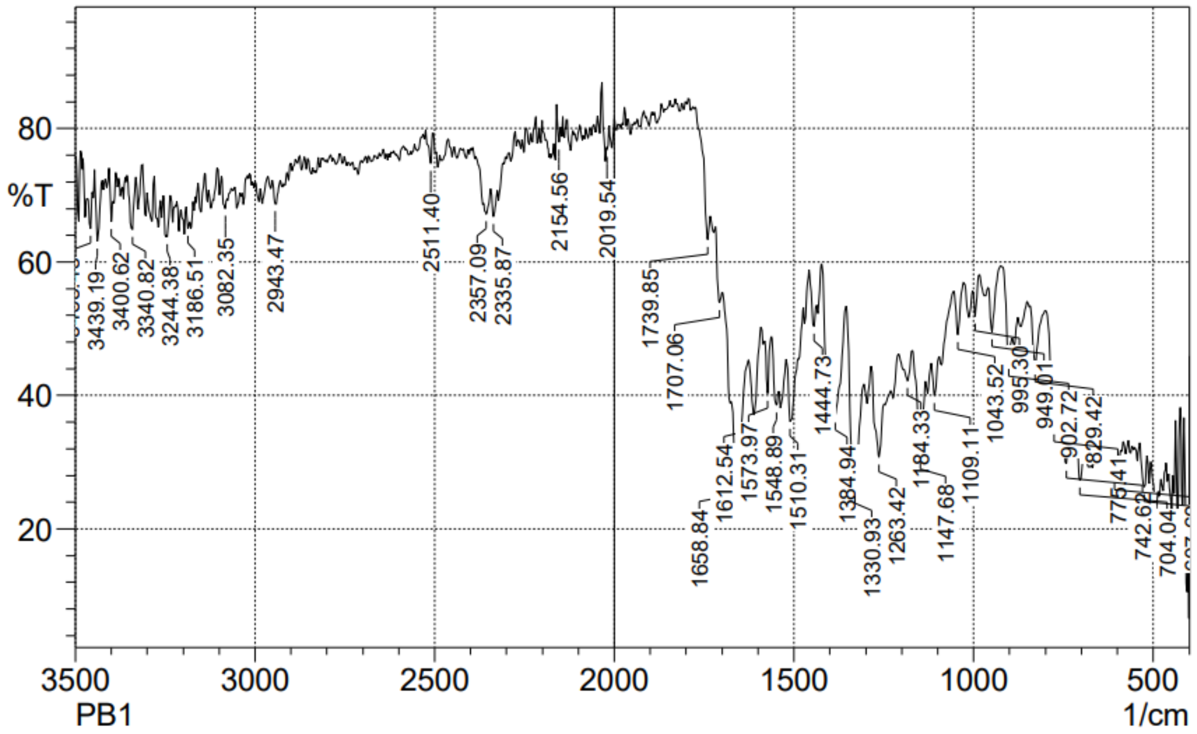

2. 1H-NMR

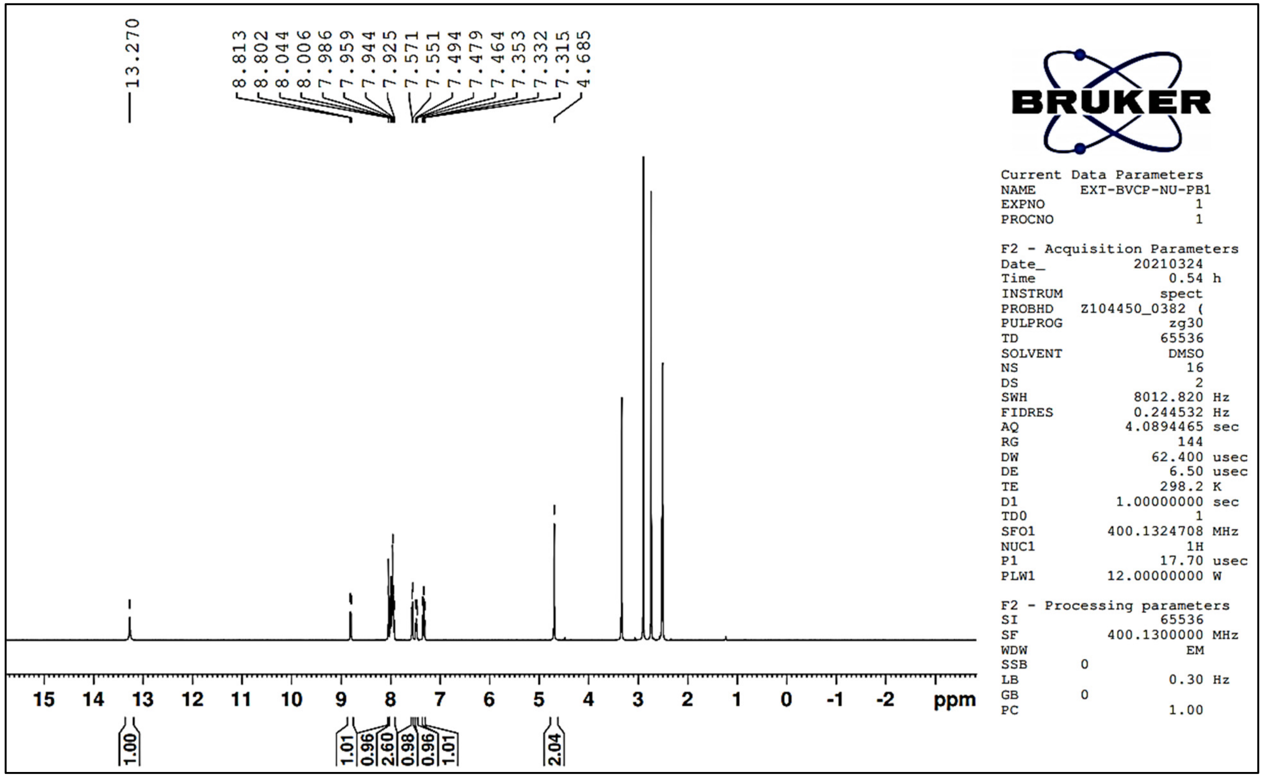

13.270
8.813
8.802
8.044
8.006
7.986
7.959
7.944
7.925
7.571
7.551
7.494
7.479
7.464
7.353
7.332
7.315
4.685
BRUKER
Current Data Parameters
NAME EXT-BVCP-NU-PB1
EXPNO 1
PROCNO 1
F2 - Acquisition Parameters
Date_ 20210324
Time 0.54 h
INSTRUM spect
PROBHD Z104450_0382 (
PULPROG zg30
TD 65536
SOLVENT DMSO
NS 16
DS 2
SWH 8012.820 Hz
FIDRES 0.244532 Hz
AQ 4.0894465 sec
RG 144
DW 62.400 usec
DE 6.50 usec
TE 298.2 K
D1 1.00000000 sec
TD0 1
SFO1 400.1324708 MHz
NUC1 1H
P1 17.70 usec
PLW1 12.00000000 W
F2 - Processing parameters
SI 65536
SF 400.1300000 MHz
WDW EM
SSB 0
LB 0.30 Hz
GB 0
PC 1.00
15 14 13 12 11 10 9 8 7 6 5 4 3 2 1 0 -1 -2 ppm
1.00
1.01
0.96
2.60
0.98
0.96
1.01
2.04

3. 13C-NMR

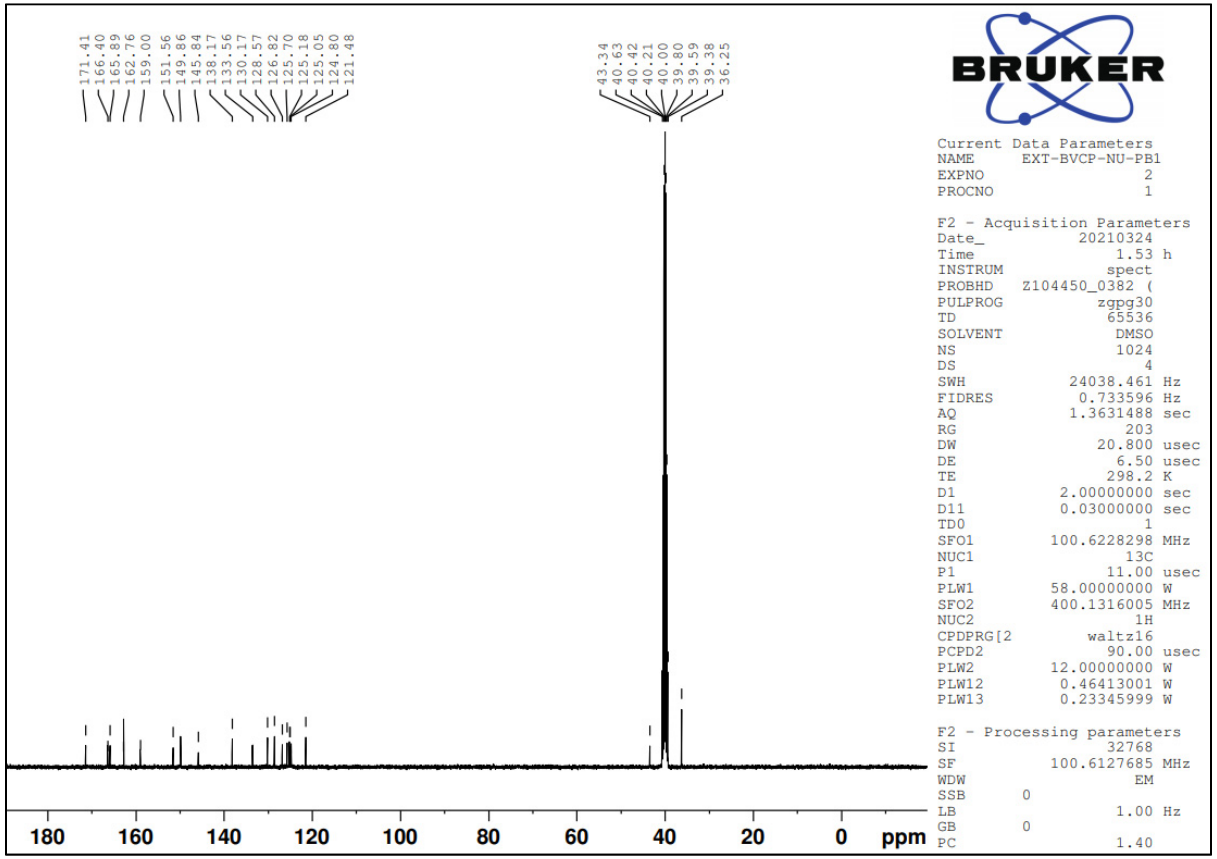
171.41
166.40
165.89
162.76
159.00
151.56
149.86
145.84
138.17
133.56
130.17
128.57
126.82
125.70
125.18
125.05
124.80
121.48
43.34
40.63
40.42
40.21
40.00
39.80
39.59
39.38
36.25
BRUKER
Current Data Parameters
NAME EXT-BVCP-NU-PB1
EXPNO 2
PROCNO 1
F2 - Acquisition Parameters
Date_ 20210324
Time 1.53 h
INSTRUM spect
PROBHD Z104450_0382 (
PULPROG zgpg30
TD 65536
SOLVENT DMSO
NS 1024
DS 4
SWH 24038.461 Hz
FIDRES 0.733596 Hz
AQ 1.3631488 sec
RG 203
DW 20.800 usec
DE 6.50 usec
TE 298.2 K
D1 2.00000000 sec
D11 0.03000000 sec
TD0 1
SFO1 100.6228298 MHz
NUC1 13C
P1 11.00 usec
PLW1 58.00000000 W
SFO2 400.1316005 MHz
NUC2 1H
CPDPRG[2 waltz16
PCPD2 90.00 usec
PLW2 12.00000000 W
PLW12 0.46413001 W
PLW13 0.23345999 W
F2 - Processing parameters
SI 32768
SF 100.6127685 MHz
WDW EM
SSB 0
LB 1.00 Hz
GB 0
PC 1.40
180
160
140
120
100
80
60
40
20
0
ppm

4. HPLC

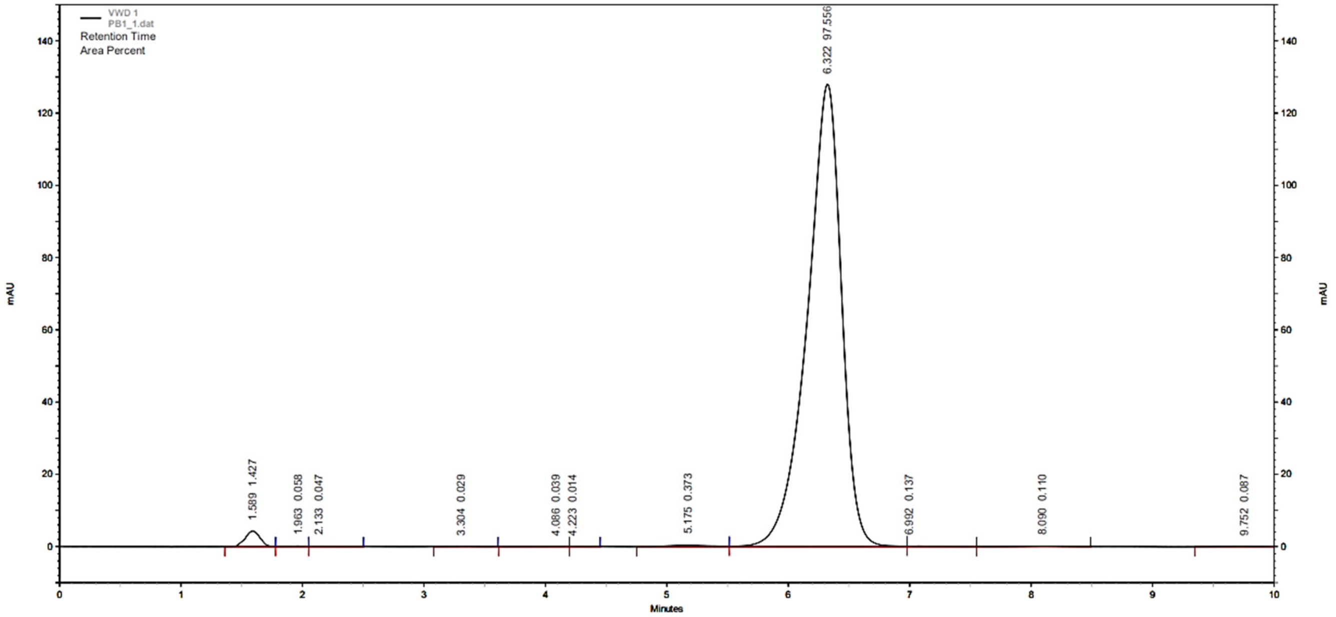
VWD 1
PB1_1.dat
Retention Time
Area Percent
mAU
Minutes
1.589 1.427
1.963 0.058
2.133 0.047
3.304 0.029
4.086 0.039
4.223 0.014
5.175 0.373
6.322 97.556
6.992 0.137
8.090 0.110
9.752 0.087

5. Mass

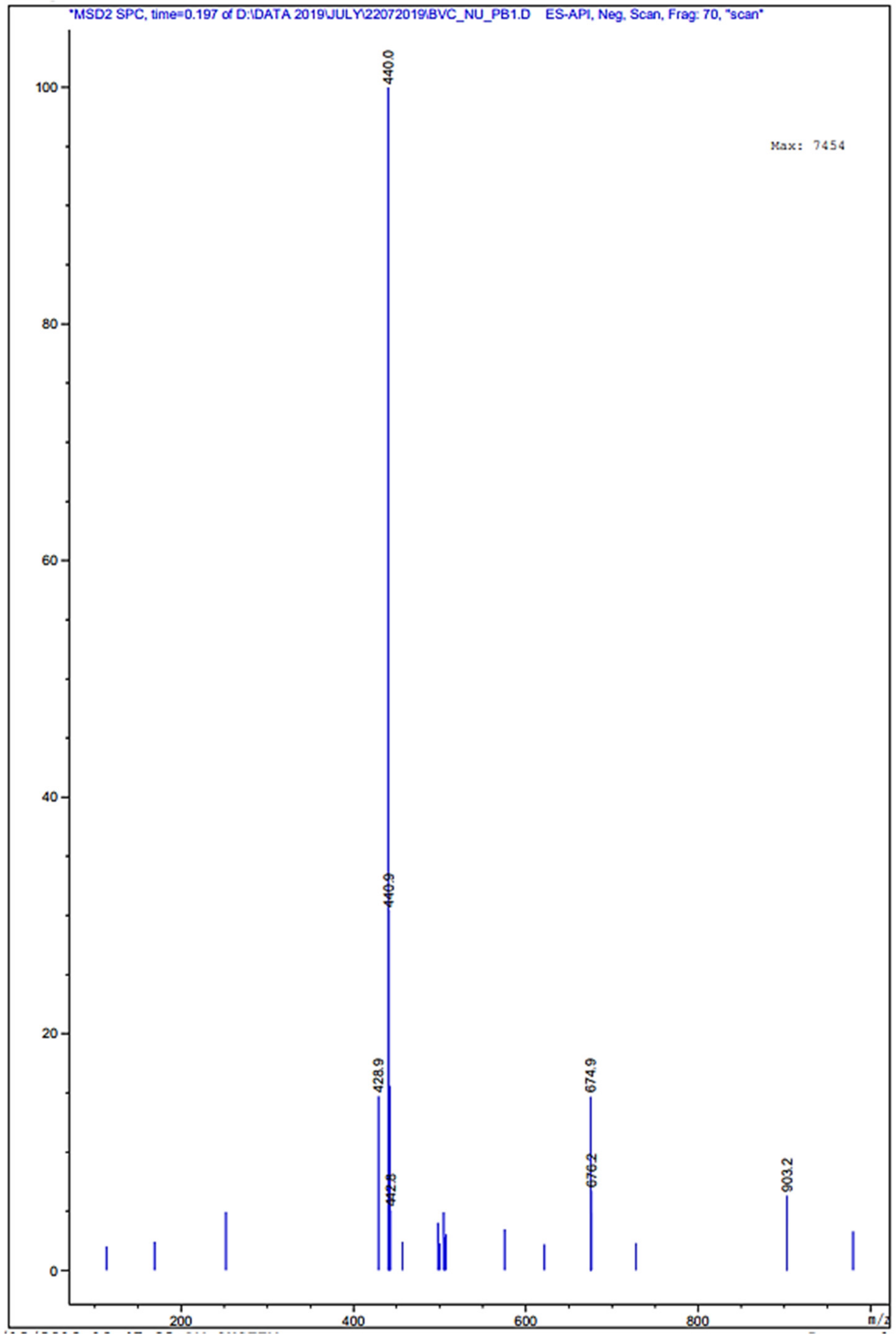
*MSD2 SPC, time=0.197 of D:\DATA 2019\JULY\22072019\BVC_NU_PB1.D ES-API, Neg, Scan, Frag: 70, "scan"
Max: 7454
440.0
440.9
428.9
442.0
674.9
676.2
903.2
100
80
60
40
20
0
200
400
600
800
m/z

2-(2,4-dioxo-5-(pyridin-2-ylmethylene)thiazolidin-3-yl)-N-(4-methylbenzo[d]thiazol-2-yl)acetamide (PB2)

1. FTIR

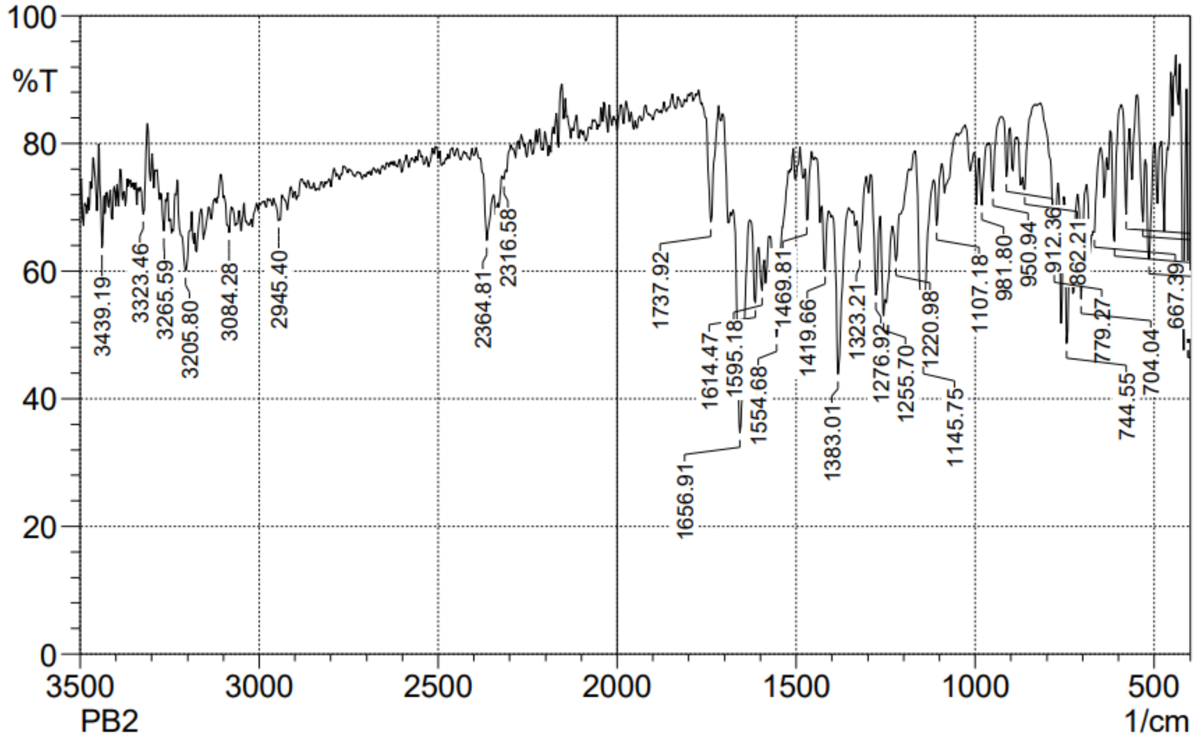

2. 1H-NMR

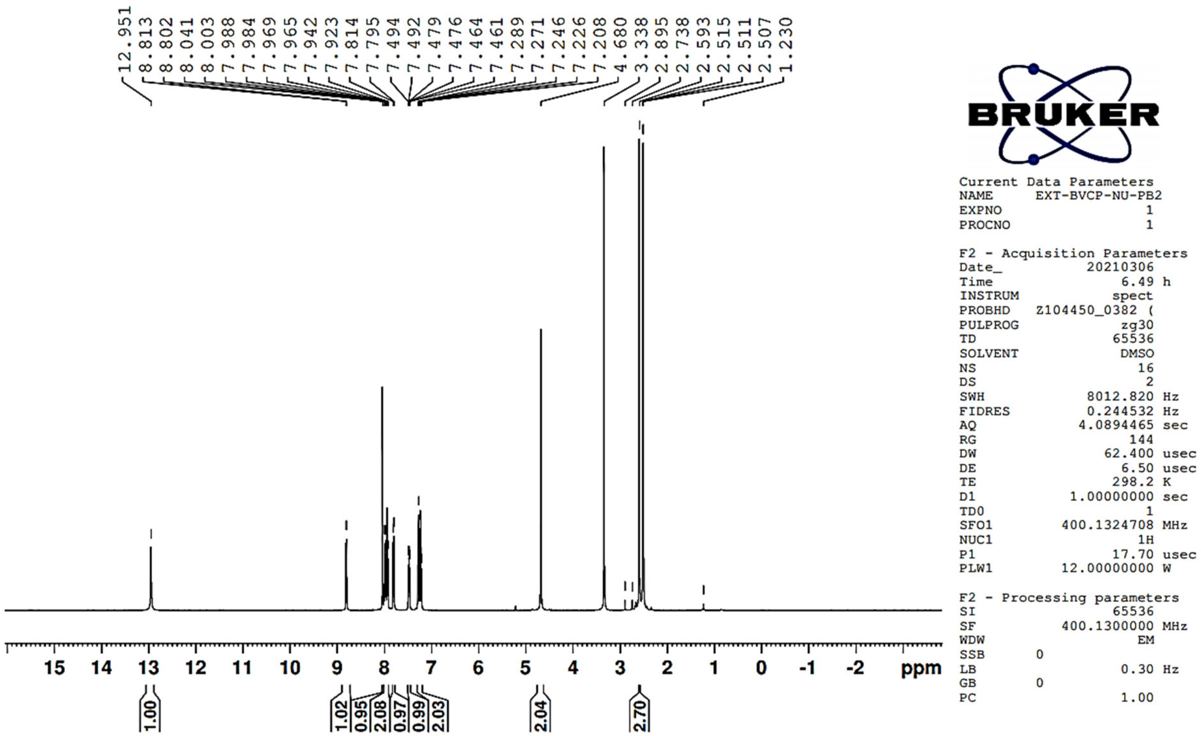
12.951
8.813
8.802
8.041
8.003
7.988
7.984
7.969
7.965
7.942
7.923
7.814
7.795
7.494
7.492
7.479
7.476
7.464
7.461
7.289
7.271
7.246
7.226
7.208
4.680
3.338
2.895
2.738
2.593
2.515
2.511
2.507
1.230
15 14 13 12 11 10 9 8 7 6 5 4 3 2 1 0 -1 -2 ppm
1.00
1.02
0.95
2.08
0.97
0.99
2.03
2.04
2.70
BRUKER
Current Data Parameters
NAME EXT-BVCP-NU-PB2
EXPNO 1
PROCNO 1
F2 - Acquisition Parameters
Date_ 20210306
Time 6.49 h
INSTRUM spect
PROBHD Z104450_0382 (
PULPROG zg30
TD 65536
SOLVENT DMSO
NS 16
DS 2
SWH 8012.820 Hz
FIDRES 0.244532 Hz
AQ 4.0894465 sec
RG 144
DW 62.400 usec
DE 6.50 usec
TE 298.2 K
D1 1.00000000 sec
TD0 1
SFO1 400.1324708 MHz
NUC1 1H
P1 17.70 usec
PLW1 12.00000000 W
F2 - Processing parameters
SI 65536
SF 400.1300000 MHz
WDW EM
SSB 0
LB 0.30 Hz
GB 0
PC 1.00

3. 13C-NMR

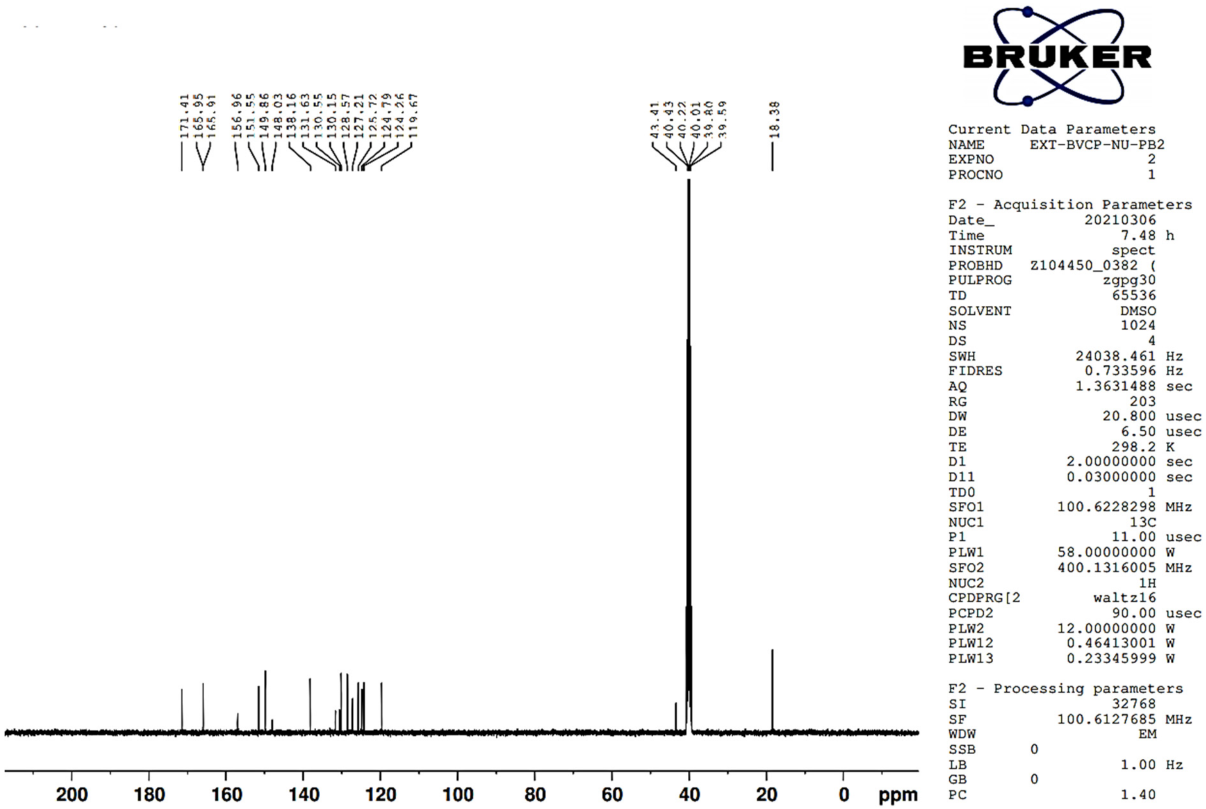
171.41
165.95
165.91
156.96
151.55
149.86
148.03
138.16
131.63
130.55
130.15
128.57
127.21
125.72
124.79
124.26
119.67
43.41
40.43
40.22
40.01
39.80
39.59
18.38
200 180 160 140 120 100 80 60 40 20 0 ppm
BRUKER
Current Data Parameters
NAME EXT-BVCP-NU-PB2
EXPNO 2
PROCNO 1
F2 - Acquisition Parameters
Date_ 20210306
Time 7.48 h
INSTRUM spect
PROBHD Z104450_0382 (
PULPROG zgpg30
TD 65536
SOLVENT DMSO
NS 1024
DS 4
SWH 24038.461 Hz
FIDRES 0.733596 Hz
AQ 1.3631488 sec
RG 203
DW 20.800 usec
DE 6.50 usec
TE 298.2 K
D1 2.00000000 sec
D11 0.03000000 sec
TD0 1
SFO1 100.6228298 MHz
NUC1 13C
P1 11.00 usec
PLW1 58.00000000 W
SFO2 400.1316005 MHz
NUC2 1H
CPDPRG[2 waltz16
PCPD2 90.00 usec
PLW2 12.00000000 W
PLW12 0.46413001 W
PLW13 0.23345999 W
F2 - Processing parameters
SI 32768
SF 100.6127685 MHz
WDW EM
SSB 0
LB 1.00 Hz
GB 0
PC 1.40

4. HPLC

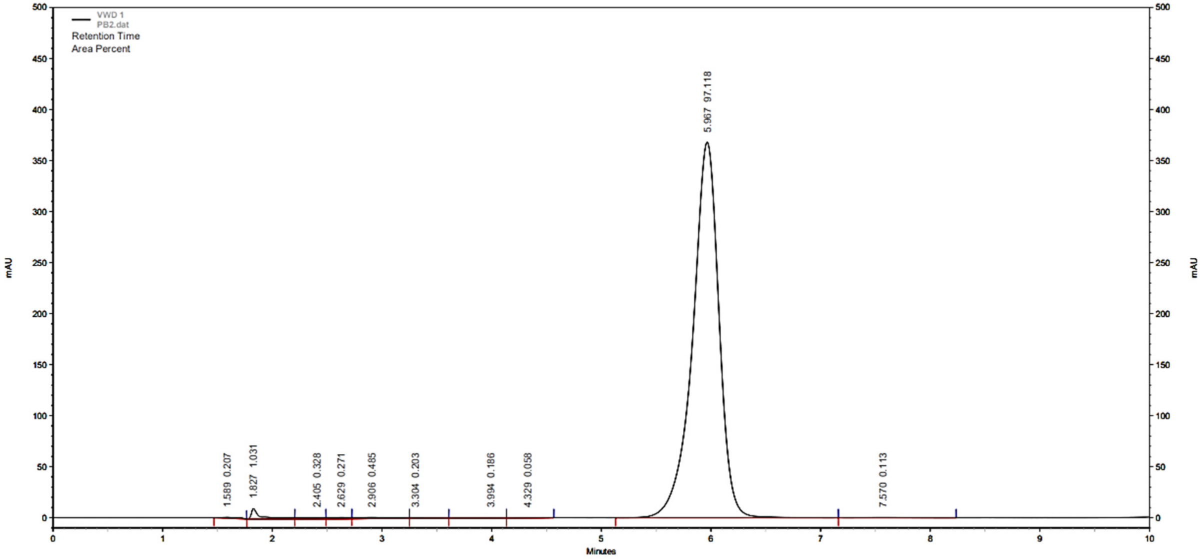
VWD 1
PB2.dat
Retention Time
Area Percent
5.967 97.118
1.589 0.207
1.827 1.031
2.405 0.328
2.629 0.271
2.906 0.485
3.304 0.203
3.994 0.186
4.329 0.058
7.570 0.113
mAU
Minutes

5. Mass

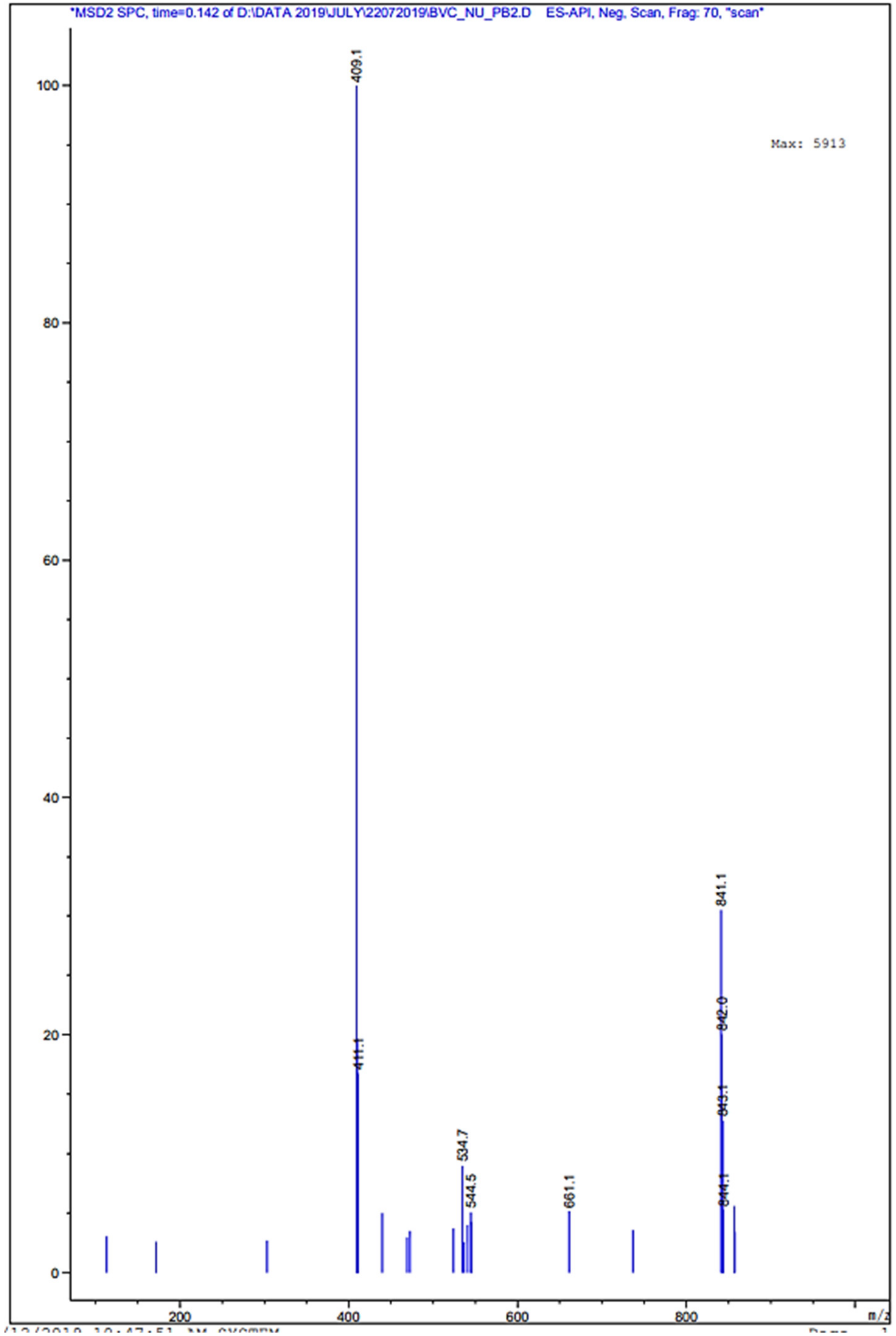
*MSD2 SPC, time=0.142 of D:\DATA 2019\JULY\22072019\BVC_NU_PB2.D ES-API, Neg, Scan, Frag: 70, "scan"
Max: 5913
409.1
411.1
534.7
544.5
661.1
841.1
842.0
843.1
844.1
100
80
60
40
20
0
200
400
600
800
m/z

2-(2,4-dioxo-5-(pyridin-2-ylmethylene)thiazolidin-3-yl)-N-(6-methylbenzo[d]thiazol-2-yl)acetamide (PB3)

1. FTIR

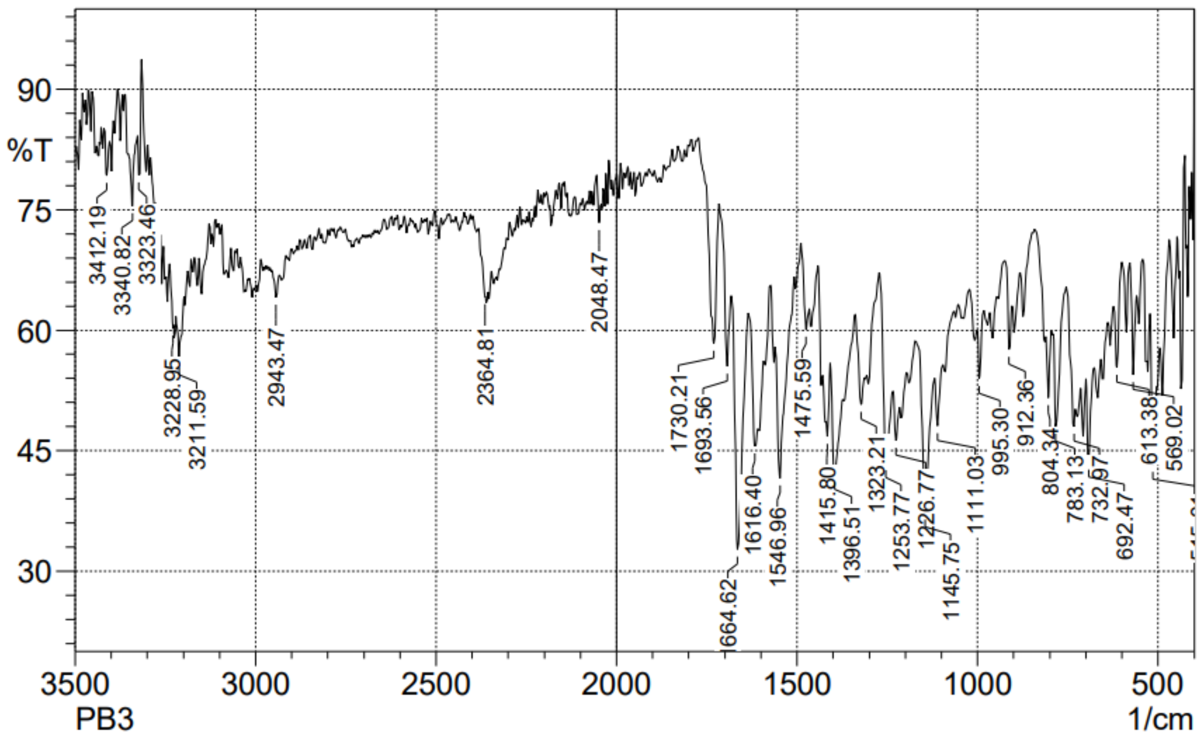

2. 1H-NMR

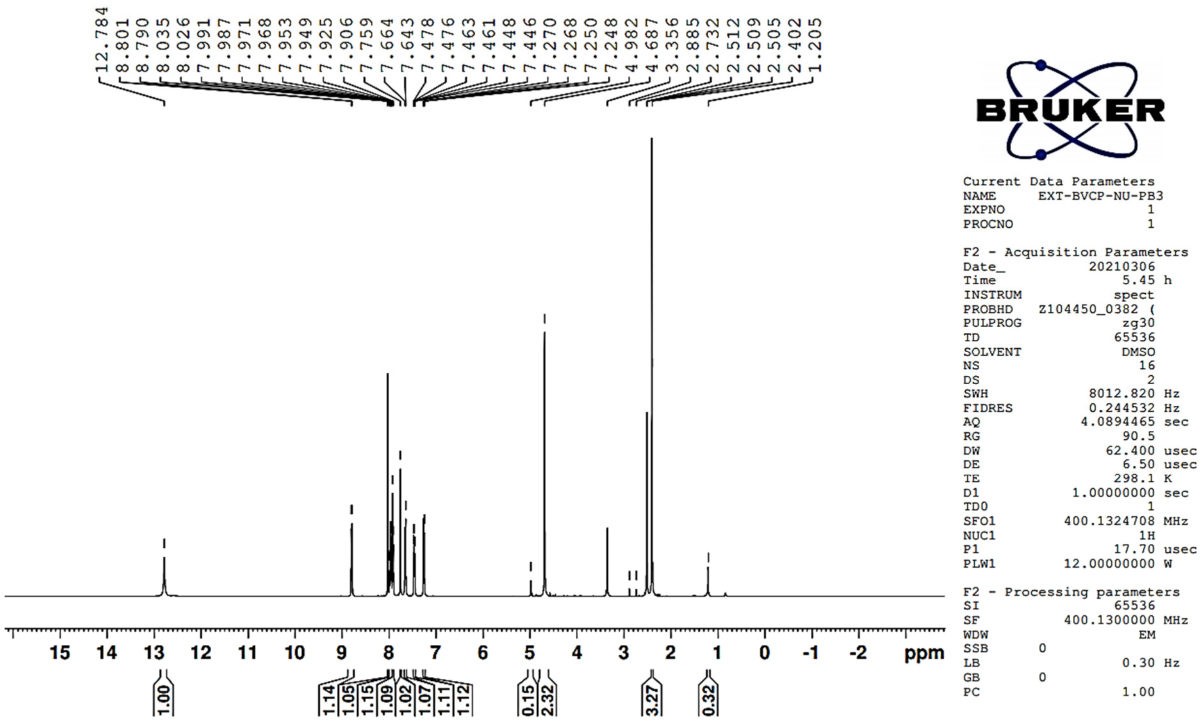
12.784
8.801
8.790
8.035
8.026
7.991
7.987
7.971
7.968
7.953
7.949
7.925
7.906
7.759
7.664
7.643
7.478
7.476
7.463
7.461
7.448
7.446
7.270
7.268
7.250
7.248
4.982
4.687
3.356
2.885
2.732
2.512
2.509
2.505
2.402
1.205
15 14 13 12 11 10 9 8 7 6 5 4 3 2 1 0 -1 -2 ppm
1.00
1.14
1.05
1.15
1.09
1.02
1.07
1.11
1.12
0.15
2.32
3.27
0.32
BRUKER
Current Data Parameters
NAME EXT-BVCP-NU-PB3
EXPNO 1
PROCNO 1
F2 - Acquisition Parameters
Date_ 20210306
Time 5.45 h
INSTRUM spect
PROBHD Z104450_0382 (
PULPROG zg30
TD 65536
SOLVENT DMSO
NS 16
DS 2
SWH 8012.820 Hz
FIDRES 0.244532 Hz
AQ 4.0894465 sec
RG 90.5
DW 62.400 usec
DE 6.50 usec
TE 298.1 K
D1 1.00000000 sec
TD0 1
SFO1 400.1324708 MHz
NUC1 1H
P1 17.70 usec
PLW1 12.00000000 W
F2 - Processing parameters
SI 65536
SF 400.1300000 MHz
WDW EM
SSB 0
LB 0.30 Hz
GB 0
PC 1.00

3. 13C-NMR

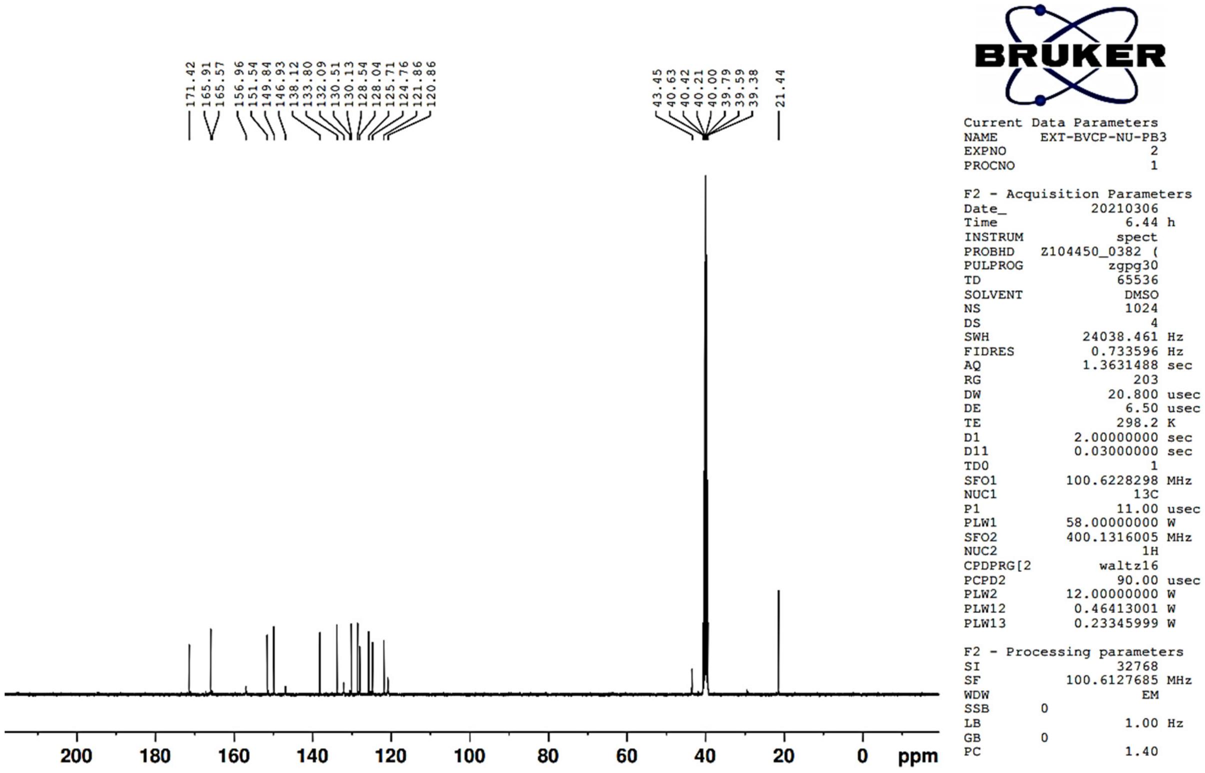
171.42
165.91
165.57
156.96
151.54
149.84
146.93
138.12
133.80
132.09
130.51
130.13
128.54
128.04
125.71
124.76
121.86
120.86
43.45
40.63
40.42
40.21
40.00
39.79
39.59
39.38
21.44
200
180
160
140
120
100
80
60
40
20
0
ppm
BRUKER
Current Data Parameters
NAME EXT-BVCP-NU-PB3
EXPNO 2
PROCNO 1
F2 - Acquisition Parameters
Date_ 20210306
Time 6.44 h
INSTRUM spect
PROBHD Z104450_0382 (
PULPROG zgpg30
TD 65536
SOLVENT DMSO
NS 1024
DS 4
SWH 24038.461 Hz
FIDRES 0.733596 Hz
AQ 1.3631488 sec
RG 203
DW 20.800 usec
DE 6.50 usec
TE 298.2 K
D1 2.00000000 sec
D11 0.03000000 sec
TD0 1
SFO1 100.6228298 MHz
NUC1 13C
P1 11.00 usec
PLW1 58.00000000 W
SFO2 400.1316005 MHz
NUC2 1H
CPDPRG[2 waltz16
PCPD2 90.00 usec
PLW2 12.00000000 W
PLW12 0.46413001 W
PLW13 0.23345999 W
F2 - Processing parameters
SI 32768
SF 100.6127685 MHz
WDW EM
SSB 0
LB 1.00 Hz
GB 0
PC 1.40

4. HPLC

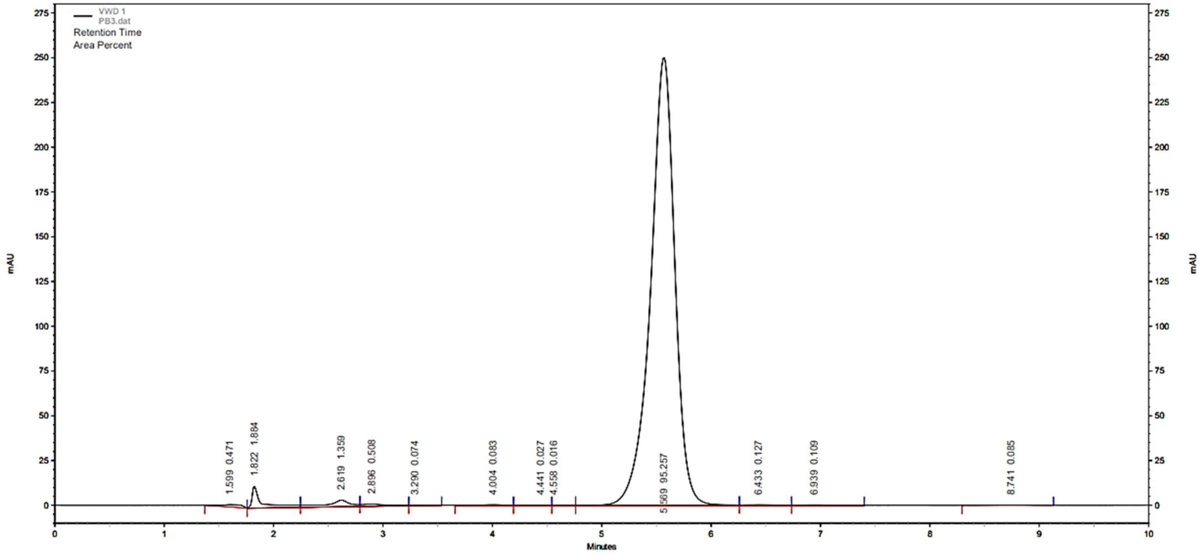
VWD 1
PB3.dat
Retention Time
Area Percent
mAU
Minutes
1.599 0.471
1.822 1.884
2.619 1.359
2.896 0.508
3.290 0.074
4.004 0.083
4.441 0.027
4.558 0.016
5.569 95.257
6.433 0.127
6.939 0.109
8.741 0.085

5. Mass

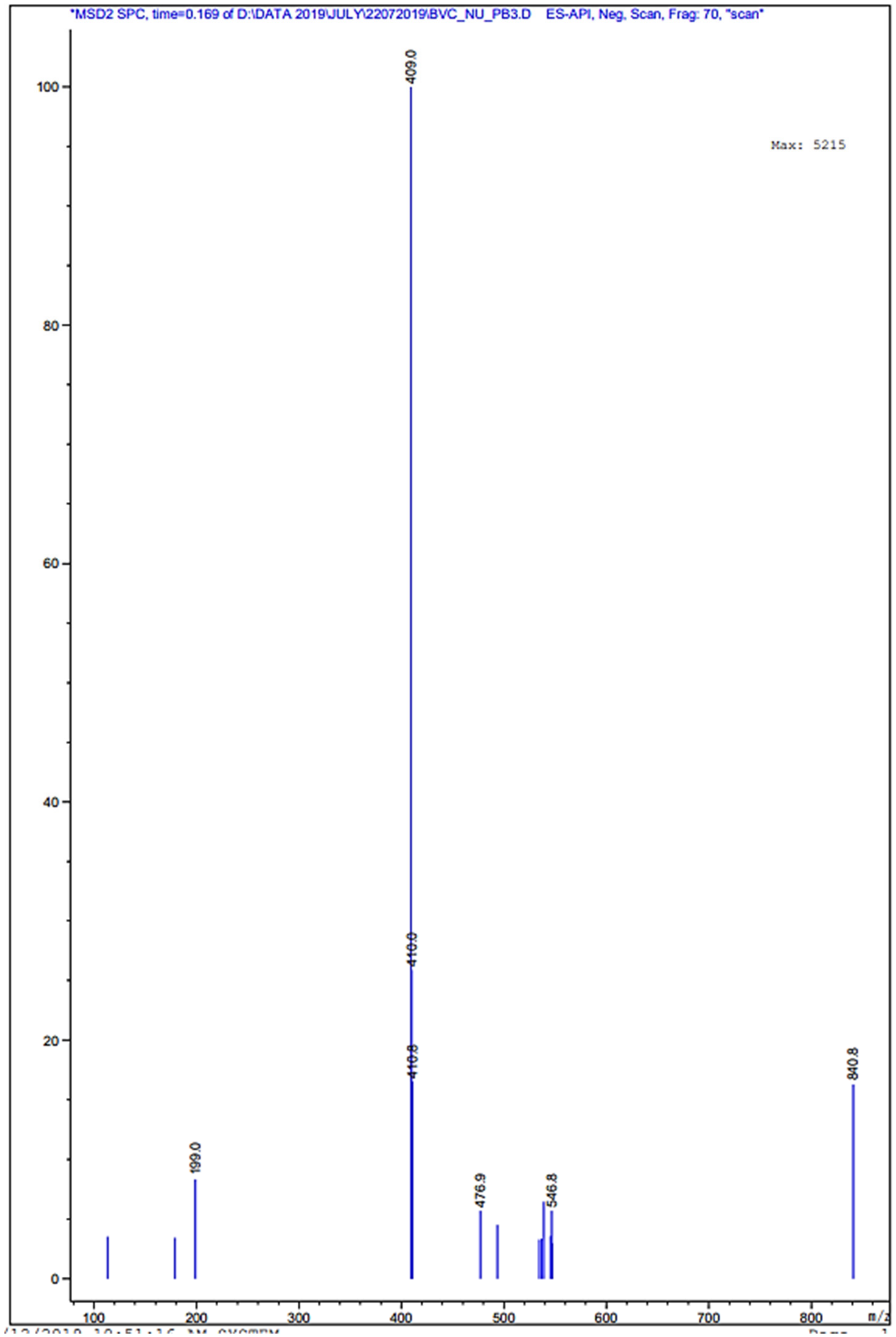
*MSD2 SPC, time=0.169 of D:\DATA 2019\JULY\22072019\BVC_NU_PB3.D ES-API, Neg, Scan, Frag: 70, "scan"
Max: 5215
409.0
410.0
410.8
199.0
476.9
546.8
840.8
100
80
60
40
20
0
100
200
300
400
500
600
700
800
m/z

N-(4-chlorobenzo[d]thiazol-2-yl)-2-(2,4-dioxo-5-(pyridin-2-ylmethylene)thiazolidin-3-yl)acetamide (PB4)

1. FTIR

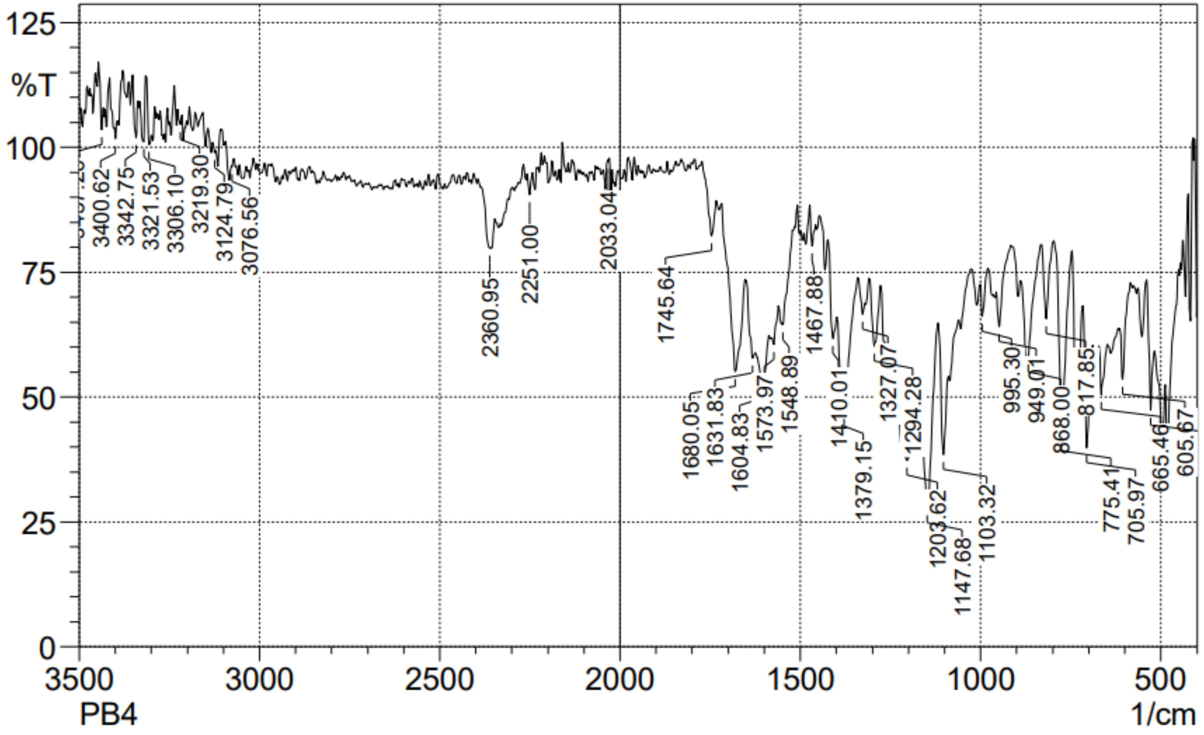

2. 1H-NMR

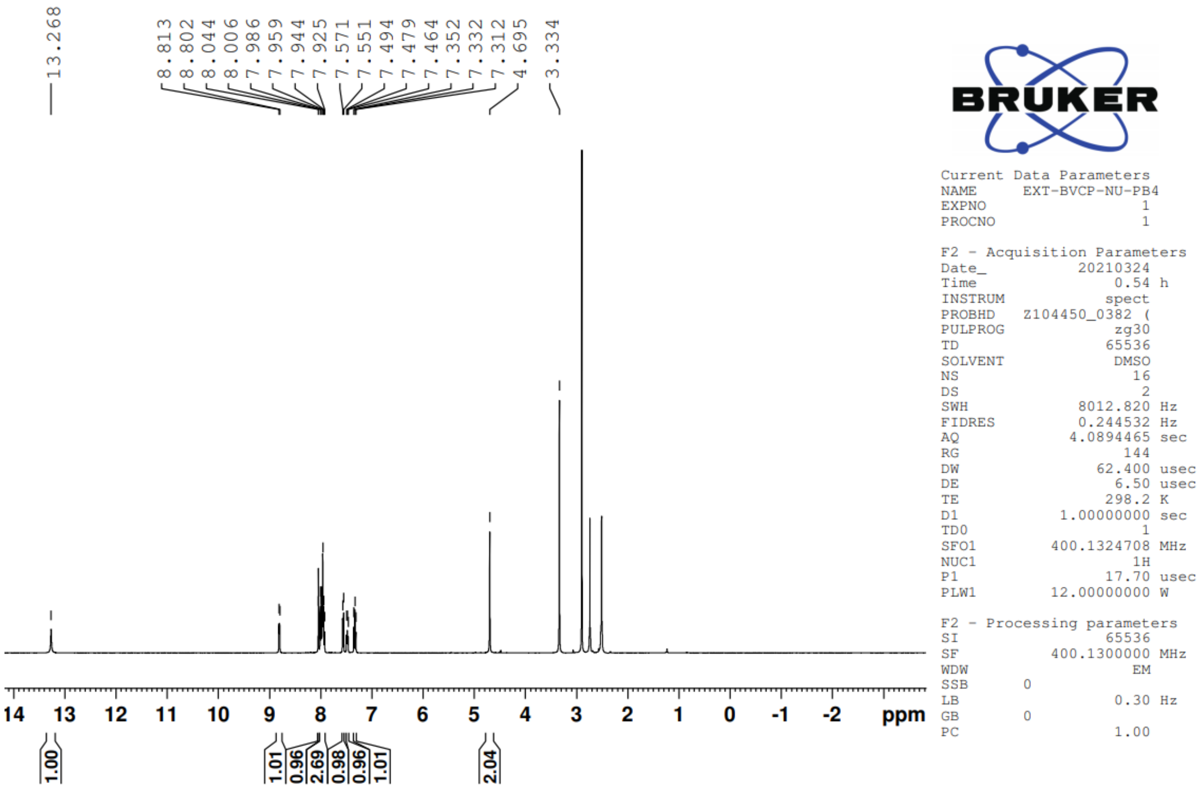
13.268
8.813
8.802
8.044
8.006
7.986
7.959
7.944
7.925
7.571
7.551
7.494
7.479
7.464
7.352
7.332
7.312
4.695
3.334
14 13 12 11 10 9 8 7 6 5 4 3 2 1 0 -1 -2 ppm
1.00
1.01
0.96
2.69
0.98
0.96
1.01
2.04
Current Data Parameters
NAME EXT-BVCP-NU-PB4
EXPNO 1
PROCNO 1
F2 - Acquisition Parameters
Date_ 20210324
Time 0.54 h
INSTRUM spect
PROBHD Z104450_0382 (
PULPROG zg30
TD 65536
SOLVENT DMSO
NS 16
DS 2
SWH 8012.820 Hz
FIDRES 0.244532 Hz
AQ 4.0894465 sec
RG 144
DW 62.400 usec
DE 6.50 usec
TE 298.2 K
D1 1.00000000 sec
TD0 1
SFO1 400.1324708 MHz
NUC1 1H
P1 17.70 usec
PLW1 12.00000000 W
F2 - Processing parameters
SI 65536
SF 400.1300000 MHz
WDW EM
SSB 0
LB 0.30 Hz
GB 0
PC 1.00

3. 13C-NMR

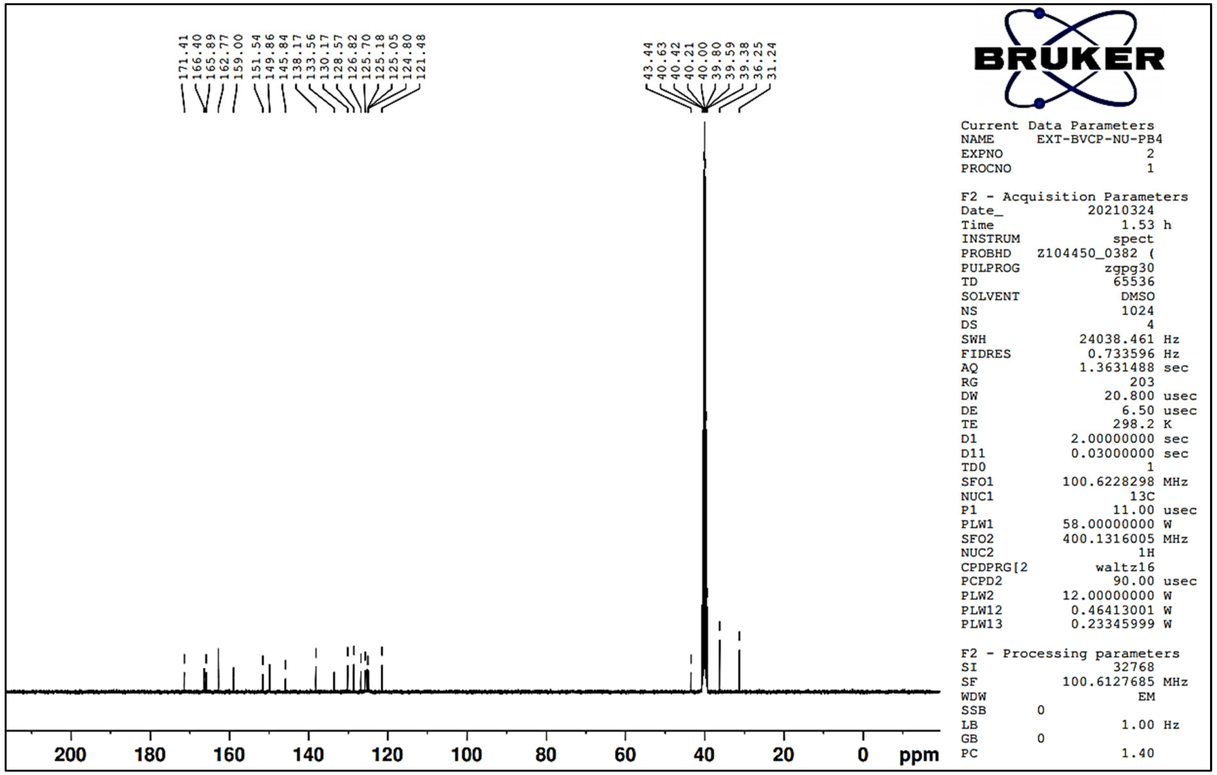
171.41
166.40
165.89
162.77
159.00
151.54
149.86
145.84
138.17
133.56
130.17
128.57
126.82
125.70
125.18
125.05
124.80
121.48
43.44
40.63
40.42
40.21
40.00
39.80
39.59
39.38
36.25
31.24
200 180 160 140 120 100 80 60 40 20 0 ppm
BRUKER
Current Data Parameters
NAME EXT-BVCP-NU-PB4
EXPNO 2
PROCNO 1
F2 - Acquisition Parameters
Date_ 20210324
Time 1.53 h
INSTRUM spect
PROBHD Z104450_0382 (
PULPROG zgpg30
TD 65536
SOLVENT DMSO
NS 1024
DS 4
SWH 24038.461 Hz
FIDRES 0.733596 Hz
AQ 1.3631488 sec
RG 203
DW 20.800 usec
DE 6.50 usec
TE 298.2 K
D1 2.00000000 sec
D11 0.03000000 sec
TD0 1
SFO1 100.6228298 MHz
NUC1 13C
P1 11.00 usec
PLW1 58.00000000 W
SFO2 400.1316005 MHz
NUC2 1H
CPDPRG[2 waltz16
PCPD2 90.00 usec
PLW2 12.00000000 W
PLW12 0.46413001 W
PLW13 0.23345999 W
F2 - Processing parameters
SI 32768
SF 100.6127685 MHz
WDW EM
SSB 0
LB 1.00 Hz
GB 0
PC 1.40

4. HPLC

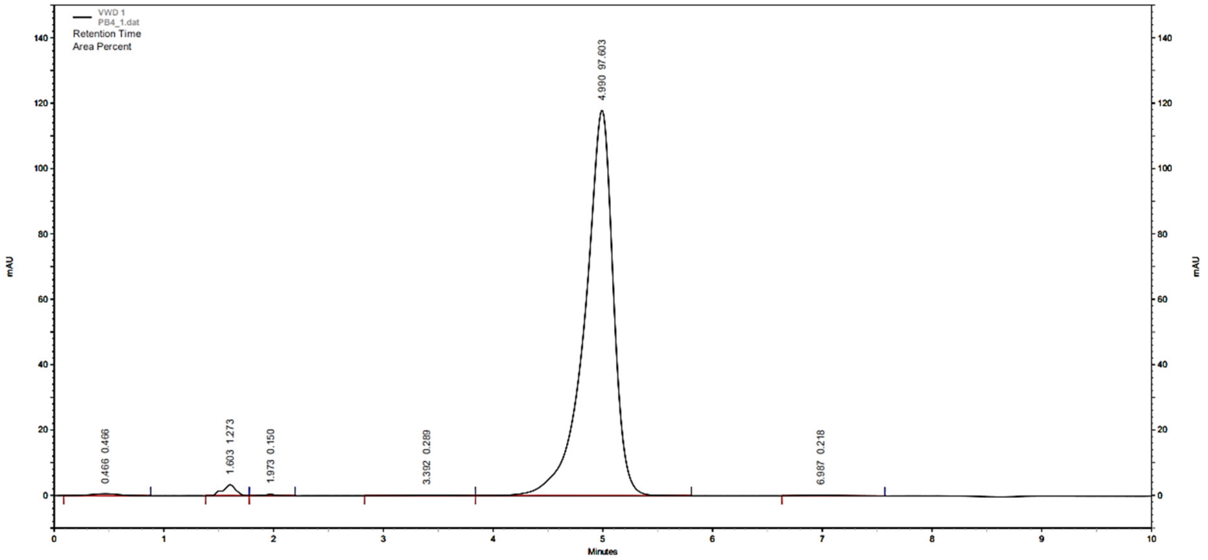
VWD 1
PB4_1.dat
Retention Time
Area Percent
0.466 0.466
1.603 1.273
1.973 0.150
3.392 0.289
4.990 97.603
6.987 0.218
mAU
Minutes

5. Mass

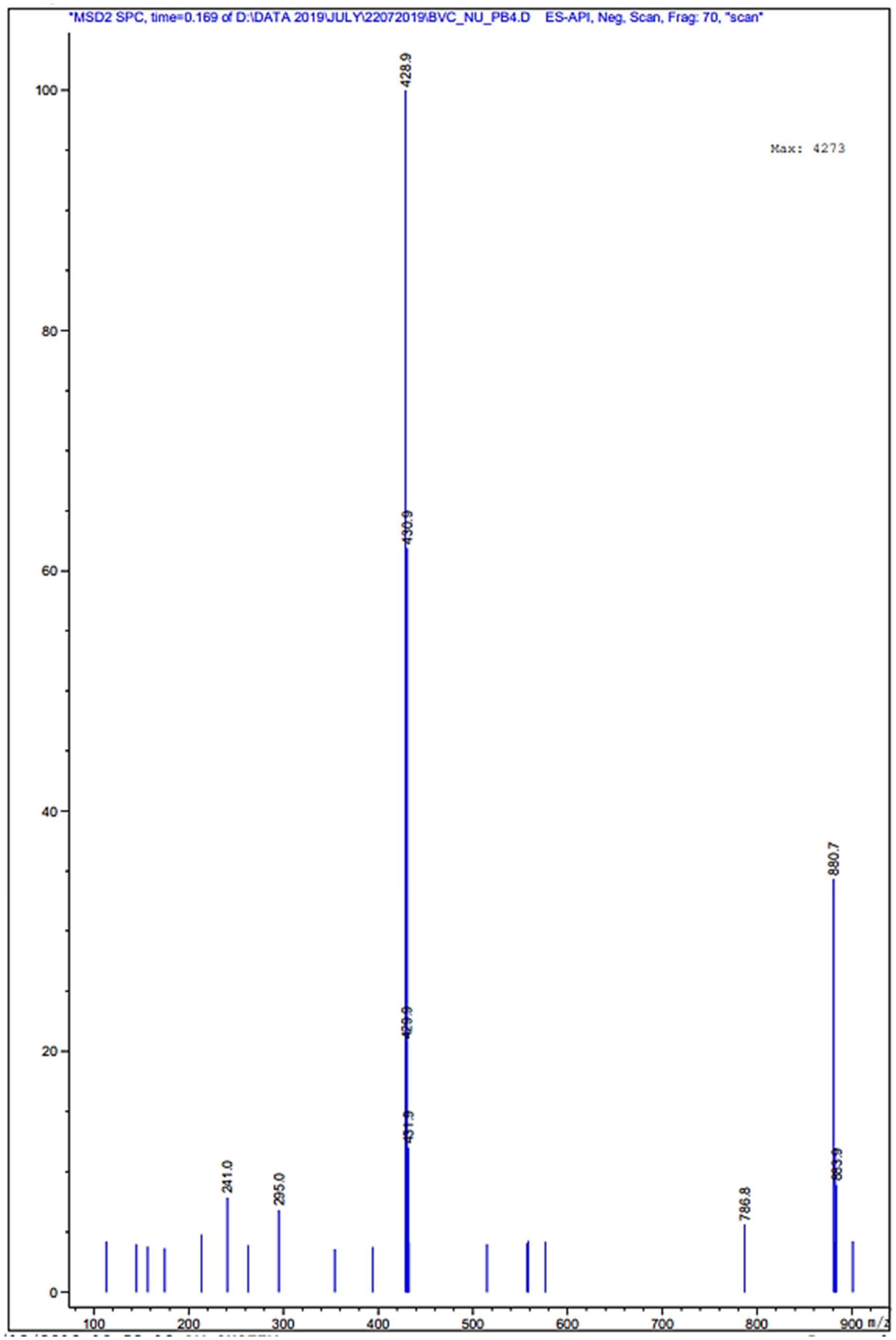
*MSD2 SPC, time=0.169 of D:\DATA 2019\JULY\22072019\BVC_NU_PB4.D ES-API, Neg, Scan, Frag: 70, "scan"
Max: 4273
428.9
430.9
241.0
295.0
786.8
880.7
m/z

N-(4,6-difluorobenzo[d]thiazol-2-yl)-2-(2,4-dioxo-5-(pyridin-2-ylmethylene)thiazolidin-3-yl)acetamide (PB5)

1. FTIR

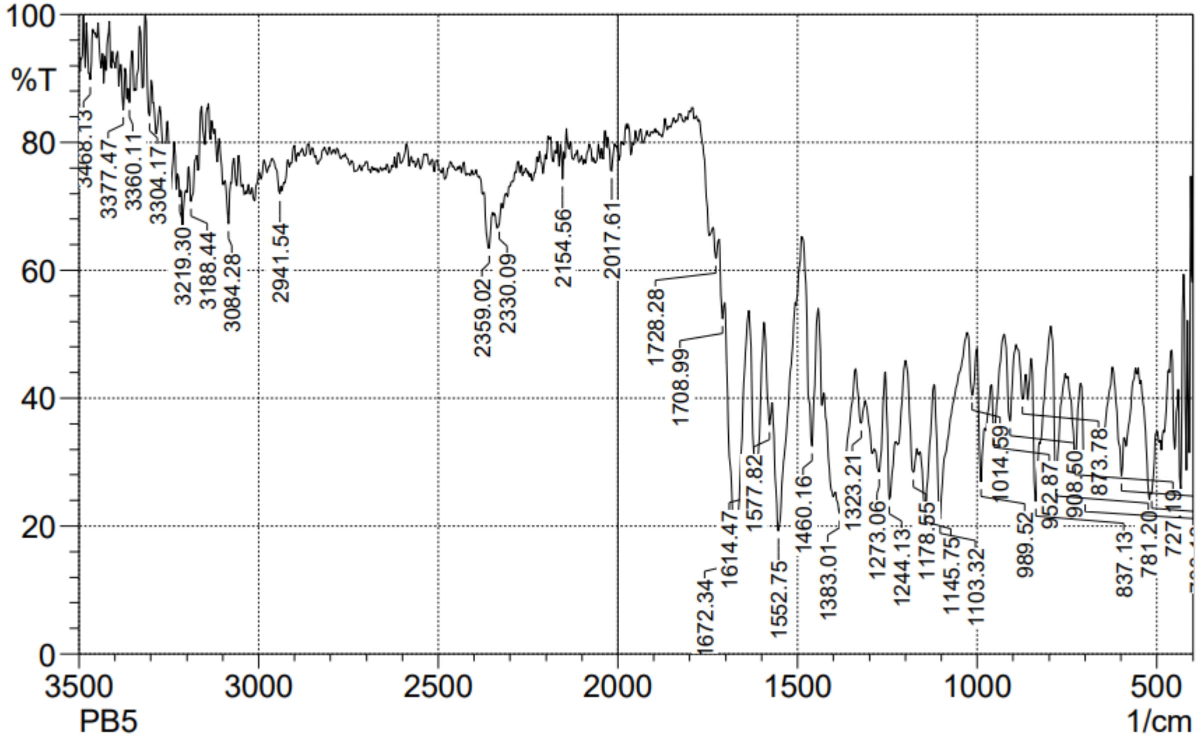

2. 1H-NMR

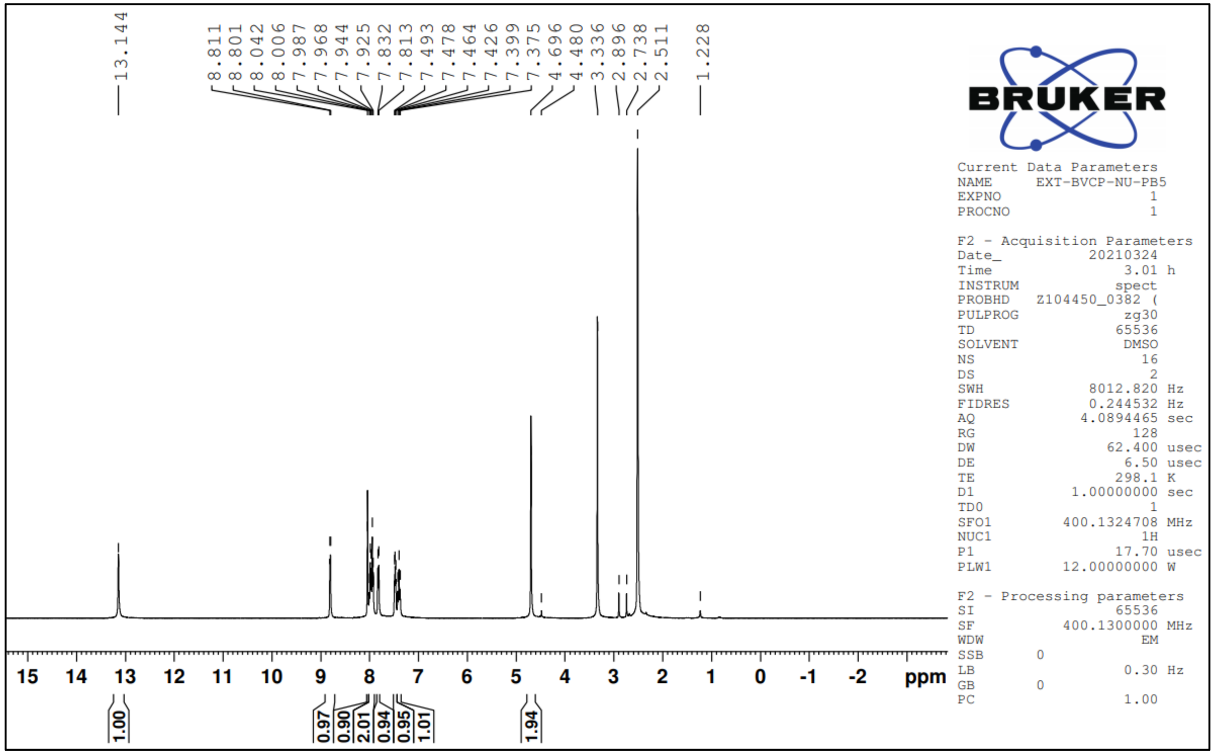
13.144
8.811
8.801
8.042
8.006
7.987
7.968
7.944
7.925
7.832
7.813
7.493
7.478
7.464
7.426
7.399
7.375
4.696
4.480
3.336
2.896
2.738
2.511
1.228
BRUKER
Current Data Parameters
NAME EXT-BVCP-NU-PB5
EXPNO 1
PROCNO 1
F2 - Acquisition Parameters
Date_ 20210324
Time 3.01 h
INSTRUM spect
PROBHD Z104450_0382 (
PULPROG zg30
TD 65536
SOLVENT DMSO
NS 16
DS 2
SWH 8012.820 Hz
FIDRES 0.244532 Hz
AQ 4.0894465 sec
RG 128
DW 62.400 usec
DE 6.50 usec
TE 298.1 K
D1 1.00000000 sec
TD0 1
SFO1 400.1324708 MHz
NUC1 1H
P1 17.70 usec
PLW1 12.00000000 W
F2 - Processing parameters
SI 65536
SF 400.1300000 MHz
WDW EM
SSB 0
LB 0.30 Hz
GB 0
PC 1.00
15 14 13 12 11 10 9 8 7 6 5 4 3 2 1 0 -1 -2 ppm
1.00
0.97
0.90
2.01
0.94
0.95
1.01
1.94

3. 13C-NMR

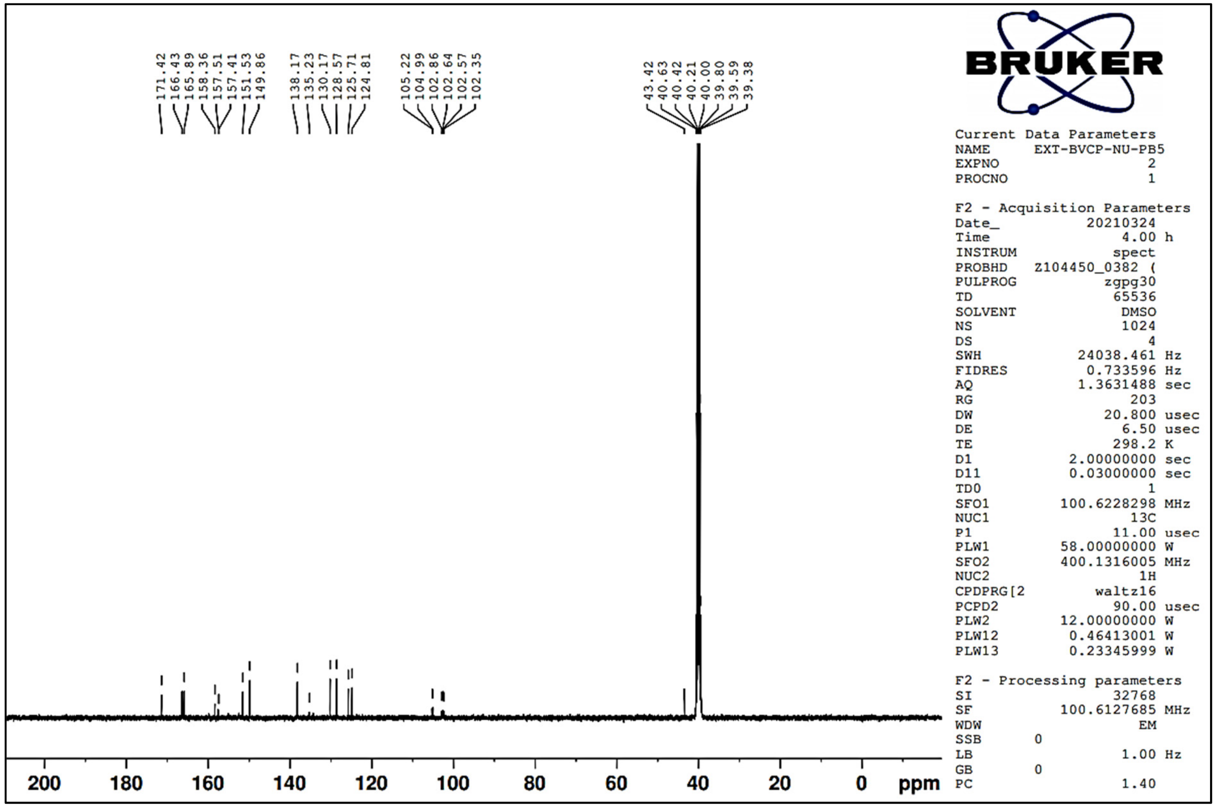
171.42
166.43
165.89
158.36
157.51
157.41
151.53
149.86
138.17
135.23
130.17
128.57
125.71
124.81
105.22
104.99
102.86
102.64
102.57
102.35
43.42
40.63
40.42
40.21
40.00
39.80
39.59
39.38
BRUKER
Current Data Parameters
NAME EXT-BVCP-NU-PB5
EXPNO 2
PROCNO 1
F2 - Acquisition Parameters
Date_ 20210324
Time 4.00 h
INSTRUM spect
PROBHD Z104450_0382 (
PULPROG zgpg30
TD 65536
SOLVENT DMSO
NS 1024
DS 4
SWH 24038.461 Hz
FIDRES 0.733596 Hz
AQ 1.3631488 sec
RG 203
DW 20.800 usec
DE 6.50 usec
TE 298.2 K
D1 2.00000000 sec
D11 0.03000000 sec
TD0 1
SFO1 100.6228298 MHz
NUC1 13C
P1 11.00 usec
PLW1 58.00000000 W
SFO2 400.1316005 MHz
NUC2 1H
CPDPRG[2 waltz16
PCPD2 90.00 usec
PLW2 12.00000000 W
PLW12 0.46413001 W
PLW13 0.23345999 W
F2 - Processing parameters
SI 32768
SF 100.6127685 MHz
WDW EM
SSB 0
LB 1.00 Hz
GB 0
PC 1.40
200 180 160 140 120 100 80 60 40 20 0 ppm

4. HPLC

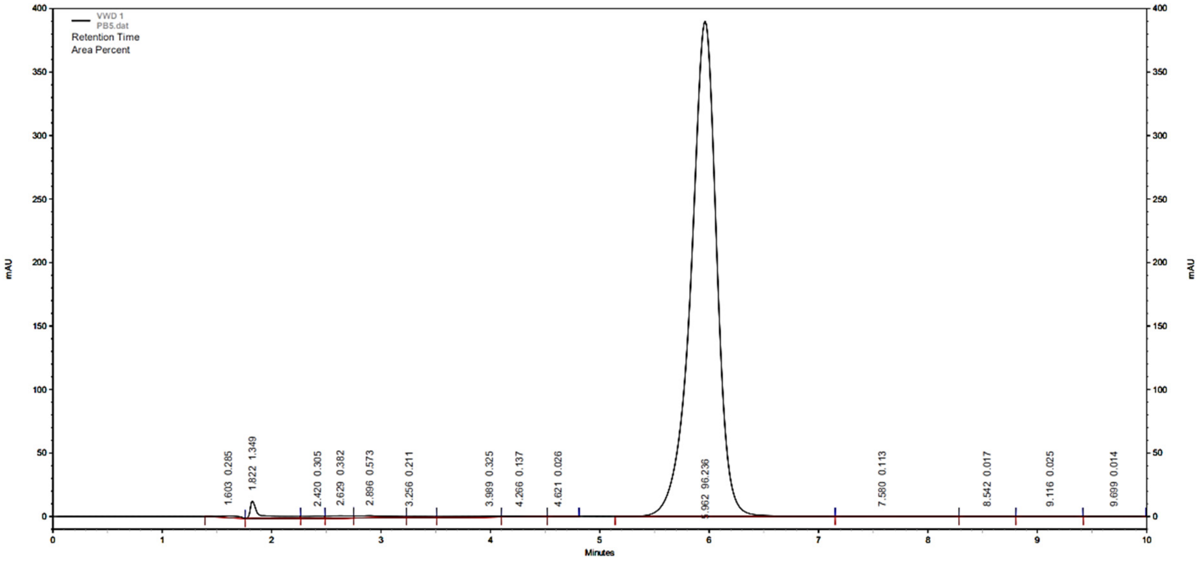
VWD 1
PB5.dat
Retention Time
Area Percent
mAU
Minutes
1.603 0.285
1.822 1.349
2.420 0.305
2.629 0.382
2.896 0.573
3.256 0.211
3.989 0.325
4.266 0.137
4.621 0.026
5.962 96.236
7.580 0.113
8.542 0.017
9.116 0.025
9.699 0.014

5. Mass

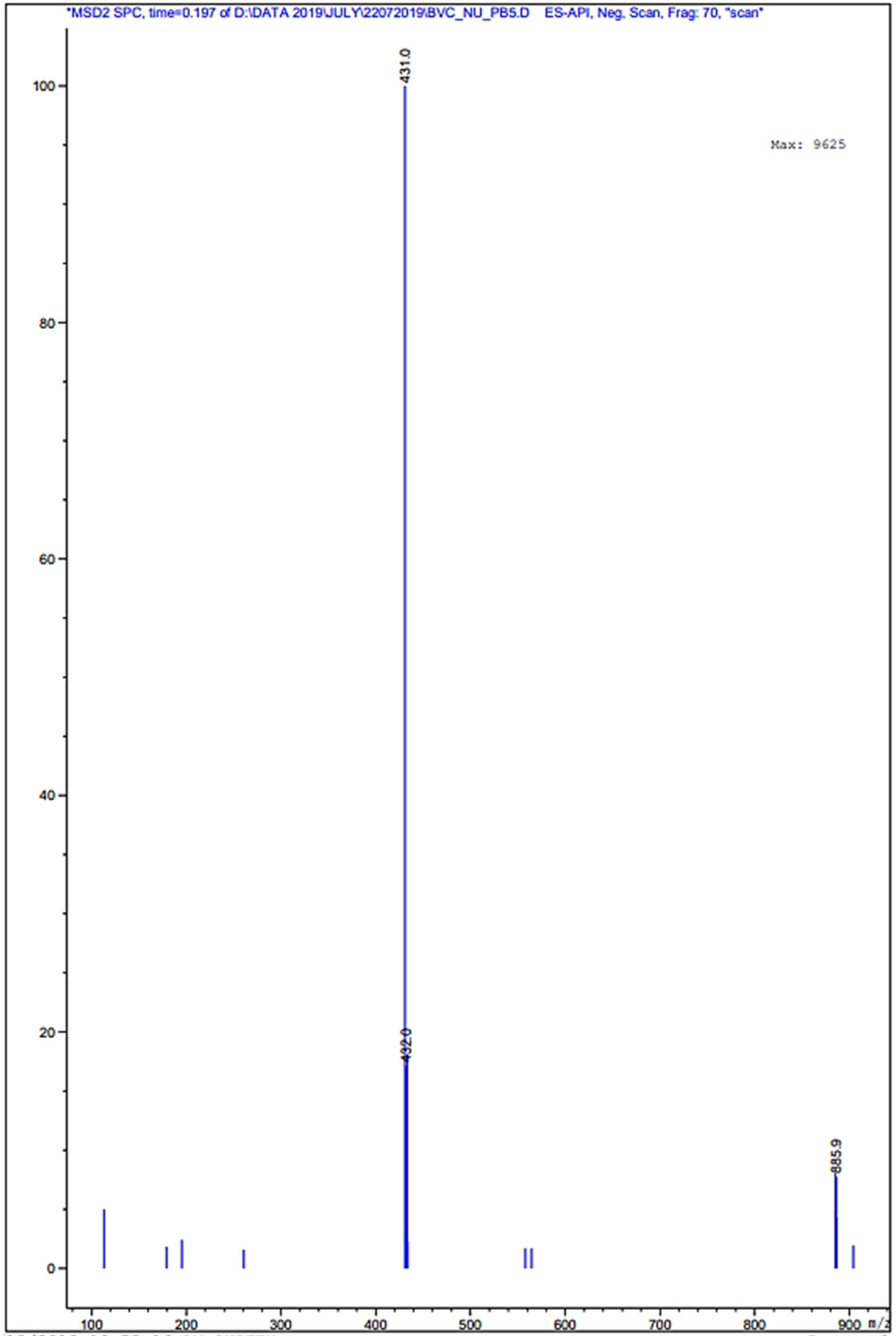
*MSD2 SPC, time=0.197 of D:\DATA 2019\JULY\22072019\BVC_NU_PB5.D ES-API, Neg, Scan, Frag: 70, "scan"
Max: 9625
431.0
432.0
885.9
100
80
60
40
20
0
100
200
300
400
500
600
700
800
900
m/z

N-(5,6-dimethylbenzo[d]thiazol-2-yl)-2-(2,4-dioxo-5-(pyridin-2-ylmethylene)thiazolidin-3-yl)acetamide (PB6)

1. FTIR

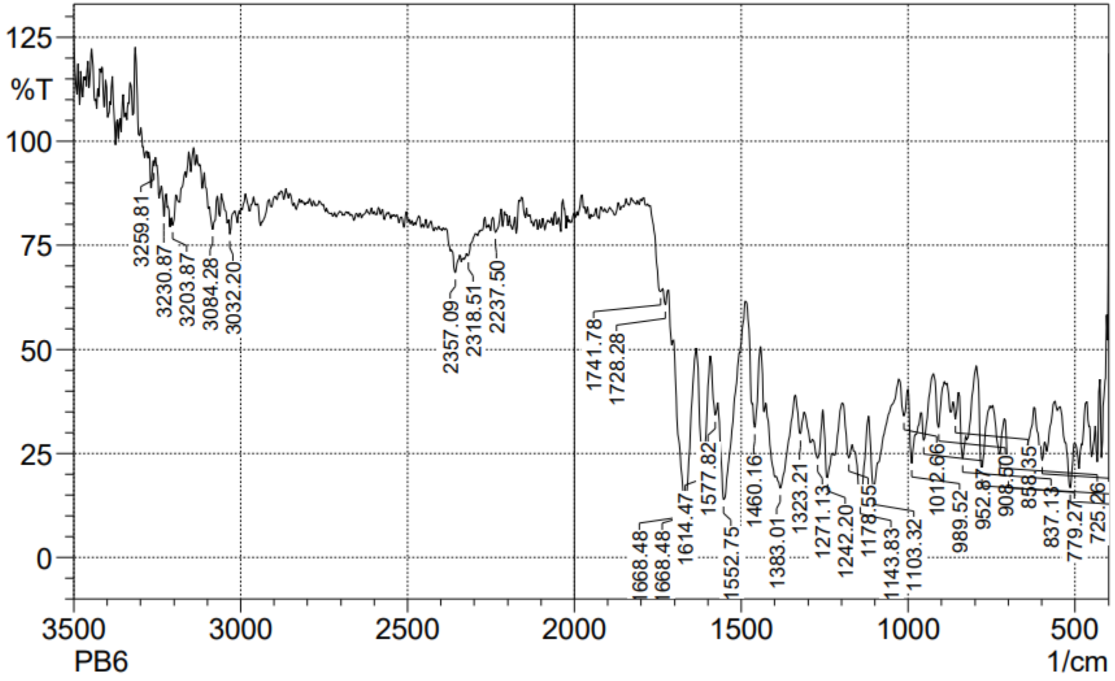

2. 1H-NMR

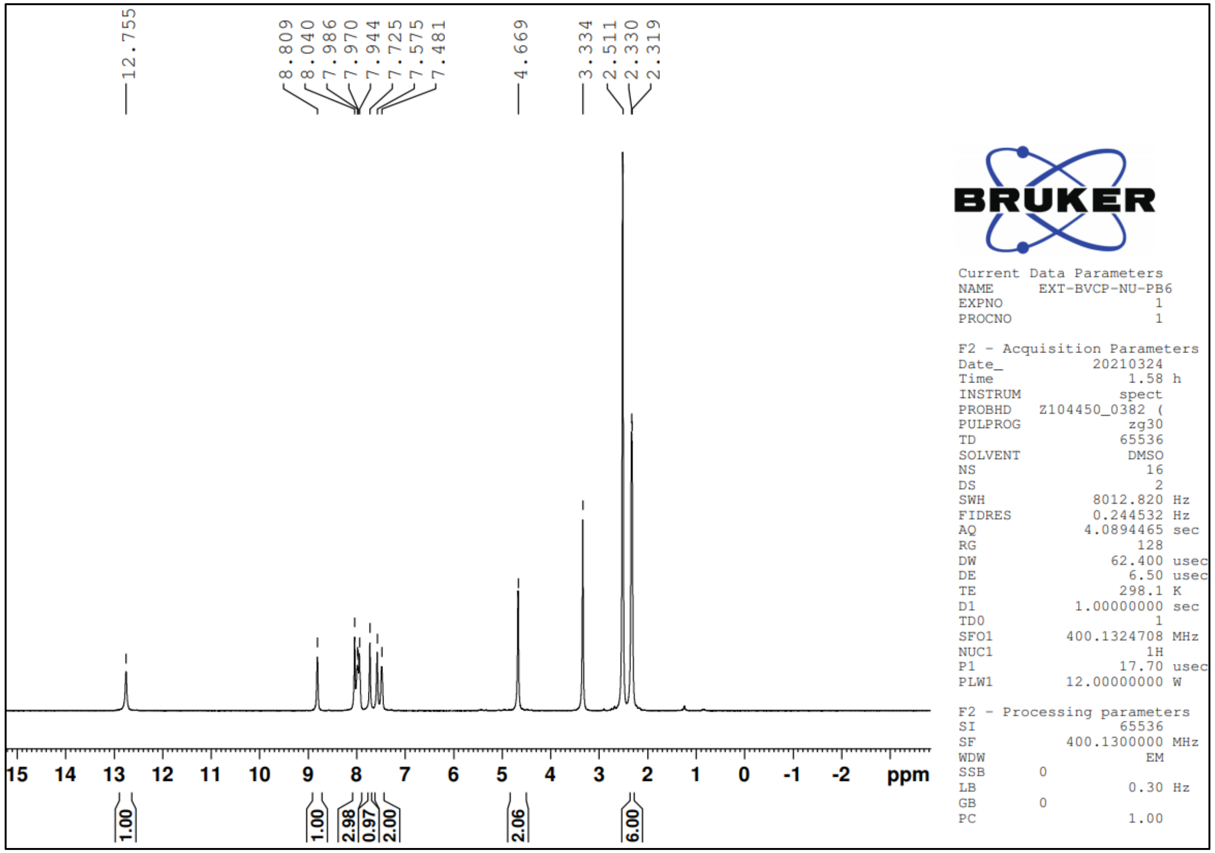
12.755
8.809
8.040
7.986
7.970
7.944
7.725
7.575
7.481
4.669
3.334
2.511
2.330
2.319
BRUKER
Current Data Parameters
NAME EXT-BVCP-NU-PB6
EXPNO 1
PROCNO 1
F2 - Acquisition Parameters
Date_ 20210324
Time 1.58 h
INSTRUM spect
PROBHD Z104450_0382 (
PULPROG zg30
TD 65536
SOLVENT DMSO
NS 16
DS 2
SWH 8012.820 Hz
FIDRES 0.244532 Hz
AQ 4.0894465 sec
RG 128
DW 62.400 usec
DE 6.50 usec
TE 298.1 K
D1 1.00000000 sec
TD0 1
SFO1 400.1324708 MHz
NUC1 1H
P1 17.70 usec
PLW1 12.00000000 W
F2 - Processing parameters
SI 65536
SF 400.1300000 MHz
WDW EM
SSB 0
LB 0.30 Hz
GB 0
PC 1.00
15 14 13 12 11 10 9 8 7 6 5 4 3 2 1 0 -1 -2 ppm
1.00
1.00
2.98
0.97
2.00
2.06
6.00

3. 13C-NMR

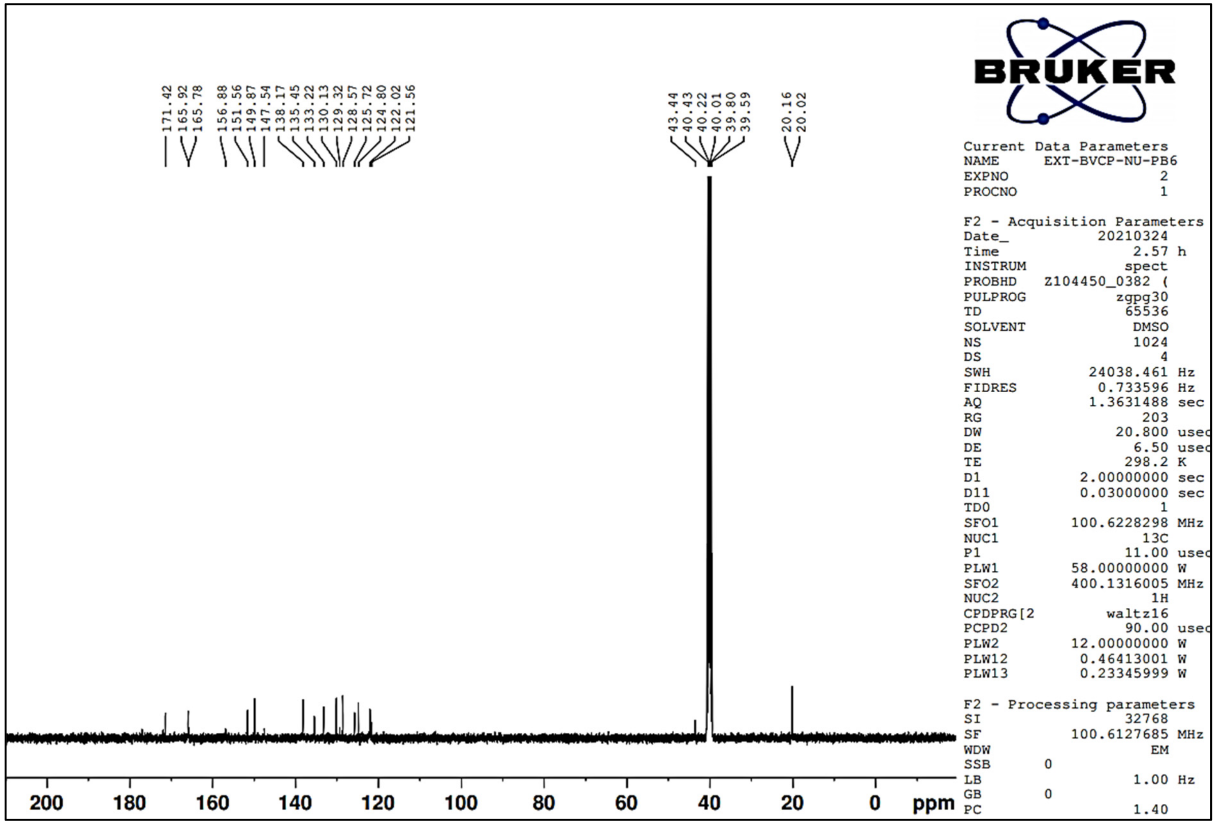
171.42
165.92
165.78
156.88
151.56
149.87
147.54
138.17
135.45
133.22
130.13
129.32
128.57
125.72
124.80
122.02
121.56
43.44
40.43
40.22
40.01
39.80
39.59
20.16
20.02
200 180 160 140 120 100 80 60 40 20 0 ppm
BRUKER
Current Data Parameters
NAME EXT-BVCP-NU-PB6
EXPNO 2
PROCNO 1
F2 - Acquisition Parameters
Date_ 20210324
Time 2.57 h
INSTRUM spect
PROBHD Z104450_0382 (
PULPROG zgpg30
TD 65536
SOLVENT DMSO
NS 1024
DS 4
SWH 24038.461 Hz
FIDRES 0.733596 Hz
AQ 1.3631488 sec
RG 203
DW 20.800 usec
DE 6.50 usec
TE 298.2 K
D1 2.00000000 sec
D11 0.03000000 sec
TD0 1
SFO1 100.6228298 MHz
NUC1 13C
P1 11.00 usec
PLW1 58.00000000 W
SFO2 400.1316005 MHz
NUC2 1H
CPDPRG[2 waltz16
PCPD2 90.00 usec
PLW2 12.00000000 W
PLW12 0.46413001 W
PLW13 0.23345999 W
F2 - Processing parameters
SI 32768
SF 100.6127685 MHz
WDW EM
SSB 0
LB 1.00 Hz
GB 0
PC 1.40

4. HPLC

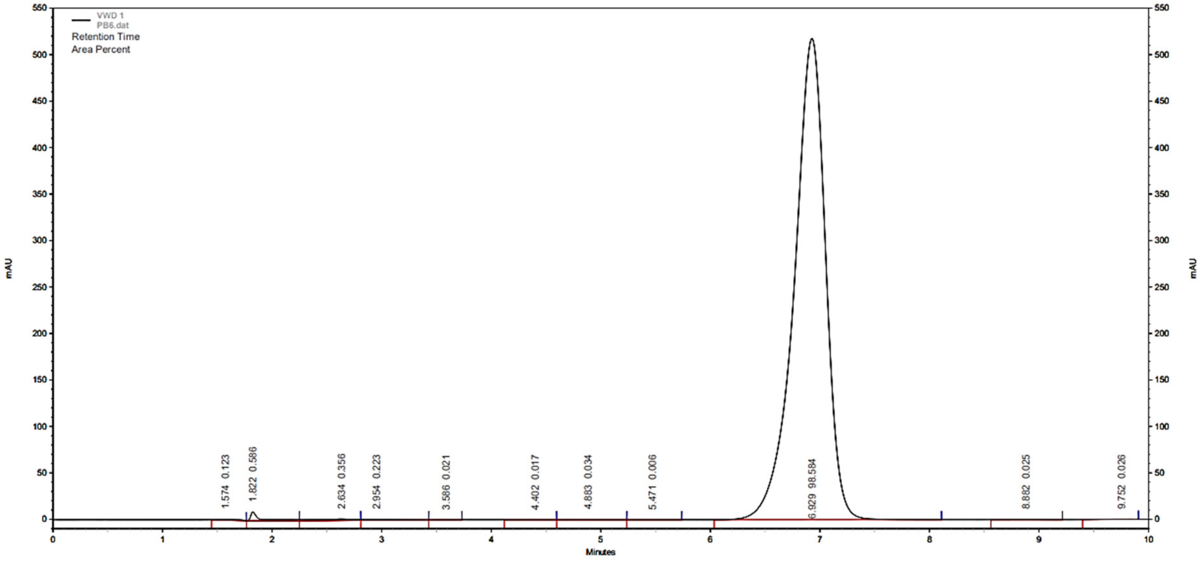
VWD 1
PB6.dat
Retention Time
Area Percent
mAU
Minutes
1.574 0.123
1.822 0.586
2.634 0.356
2.954 0.223
3.586 0.021
4.402 0.017
4.883 0.034
5.471 0.006
6.929 98.584
8.882 0.025
9.752 0.026

5. Mass

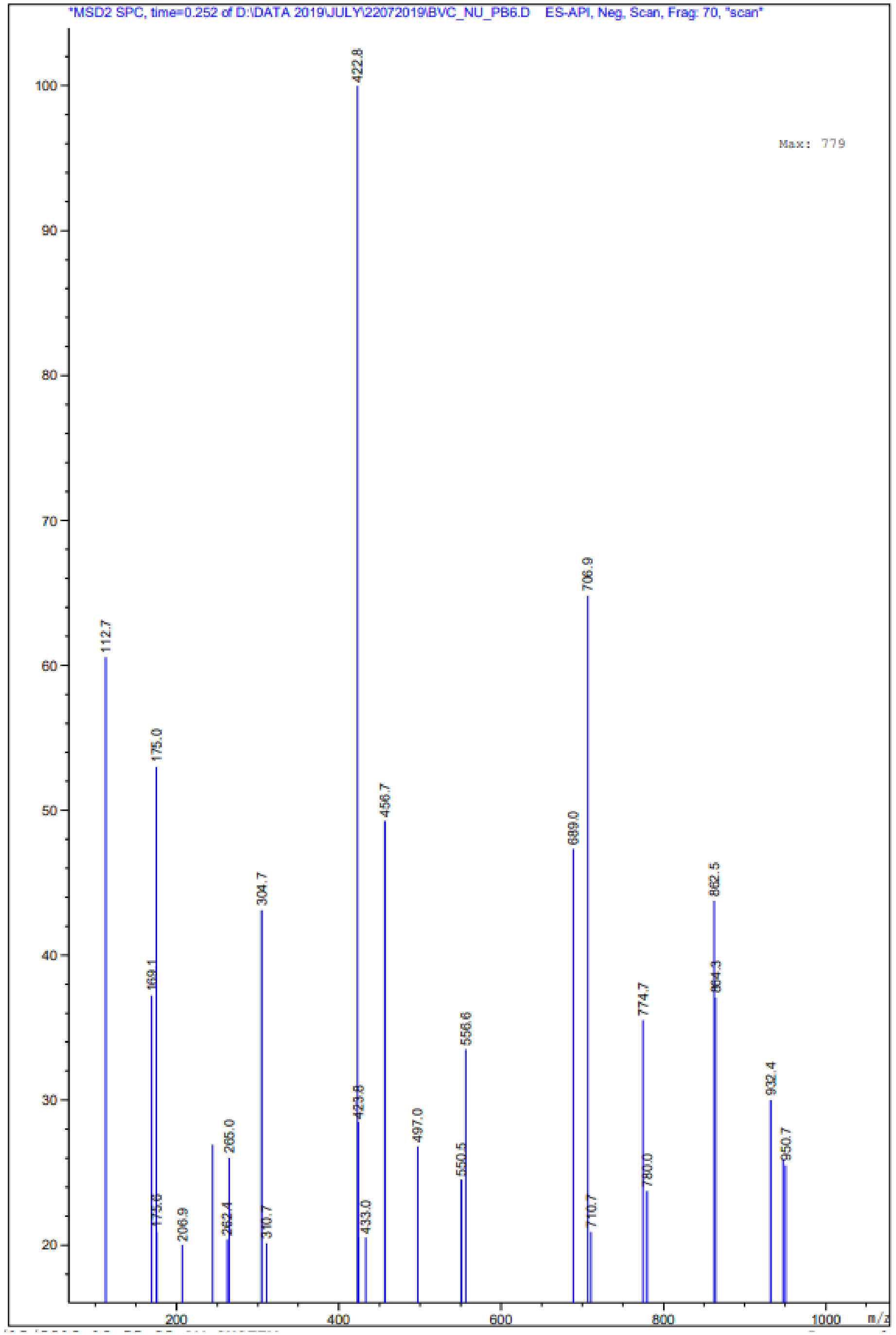
*MSD2 SPC, time=0.252 of D:\DATA 2019\JULY\22072019\BVC_NU_PB6.D ES-API, Neg, Scan, Frag: 70, "scan"
Max: 779
422.8
706.9
112.7
175.0
456.7
689.0
304.7
862.5
169.1
864.3
774.7
556.6
932.4
423.8
497.0
265.0
950.7
550.5
780.0
173.6
262.4
433.0
310.7
206.9
710.7
100
90
80
70
60
50
40
30
20
200
400
600
800
1000
m/z

2-(2,4-dioxo-5-(pyridin-2-ylmethylene)thiazolidin-3-yl)-N-(4-methoxybenzo[d]thiazol-2-yl)acetamide (PB7)

1. FTIR

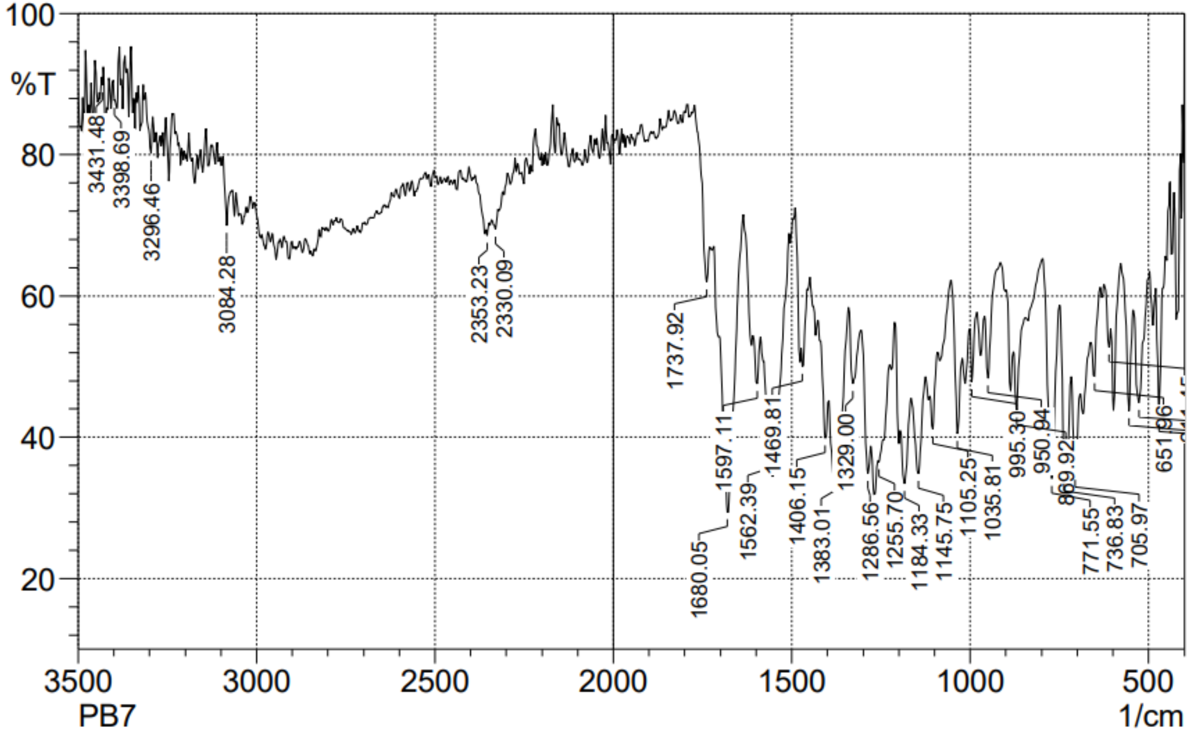

2. 1H-NMR

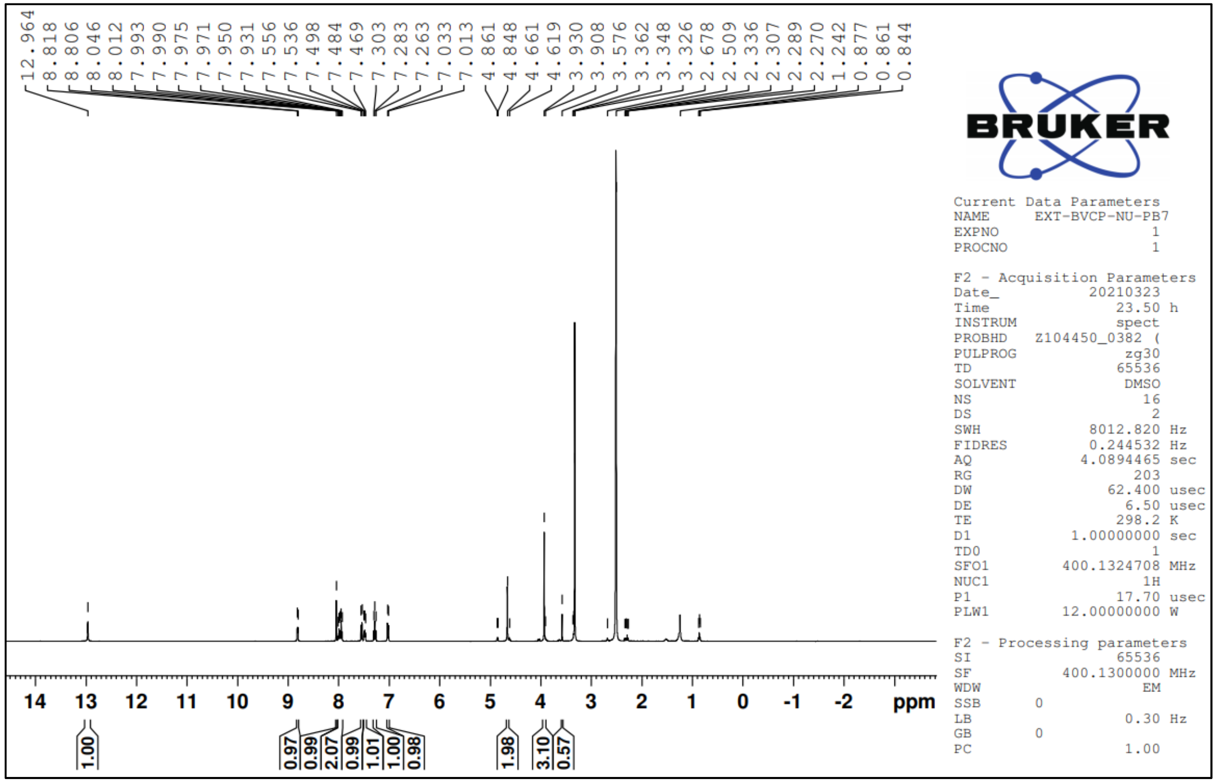

12.964
8.818
8.806
8.046
8.012
7.993
7.990
7.975
7.971
7.950
7.931
7.556
7.536
7.498
7.484
7.469
7.303
7.283
7.263
7.033
7.013
4.861
4.848
4.661
4.619
3.930
3.908
3.576
3.362
3.348
3.326
2.678
2.509
2.336
2.307
2.289
2.270
1.242
0.877
0.861
0.844
BRUKER
Current Data Parameters
NAME EXT-BVCP-NU-PB7
EXPNO 1
PROCNO 1
F2 - Acquisition Parameters
Date_ 20210323
Time 23.50 h
INSTRUM spect
PROBHD Z104450_0382 (
PULPROG zg30
TD 65536
SOLVENT DMSO
NS 16
DS 2
SWH 8012.820 Hz
FIDRES 0.244532 Hz
AQ 4.0894465 sec
RG 203
DW 62.400 usec
DE 6.50 usec
TE 298.2 K
D1 1.00000000 sec
TD0 1
SFO1 400.1324708 MHz
NUC1 1H
P1 17.70 usec
PLW1 12.00000000 W
F2 - Processing parameters
SI 65536
SF 400.1300000 MHz
WDW EM
SSB 0
LB 0.30 Hz
GB 0
PC 1.00
14 13 12 11 10 9 8 7 6 5 4 3 2 1 0 -1 -2 ppm
1.00
0.97
0.99
2.07
0.99
1.01
1.00
0.98
1.98
3.10
0.57

3. 13C-NMR

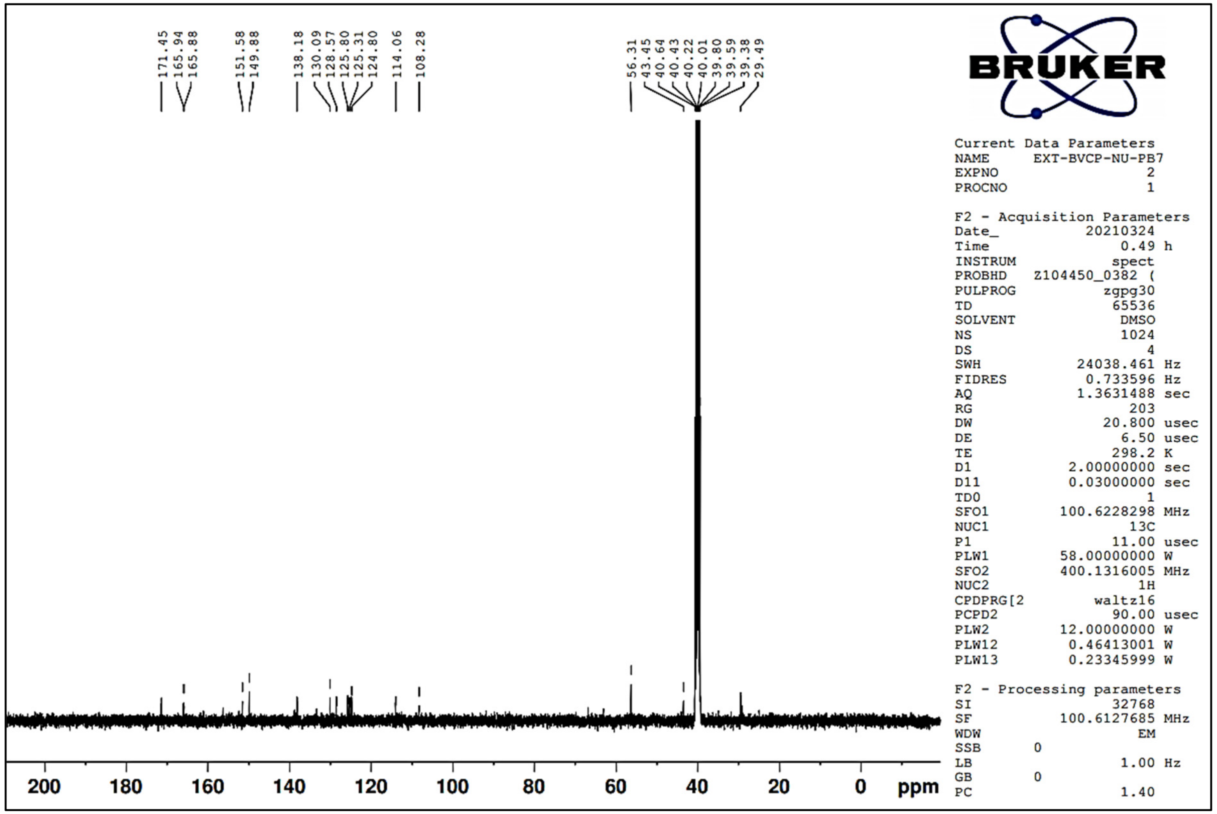
171.45
165.94
165.88
151.58
149.88
138.18
130.09
128.57
125.80
125.31
124.80
114.06
108.28
56.31
43.45
40.64
40.43
40.22
40.01
39.80
39.59
39.38
29.49
200 180 160 140 120 100 80 60 40 20 0 ppm
Current Data Parameters
NAME EXT-BVCP-NU-PB7
EXPNO 2
PROCNO 1
F2 - Acquisition Parameters
Date_ 20210324
Time 0.49 h
INSTRUM spect
PROBHD Z104450_0382 (
PULPROG zgpg30
TD 65536
SOLVENT DMSO
NS 1024
DS 4
SWH 24038.461 Hz
FIDRES 0.733596 Hz
AQ 1.3631488 sec
RG 203
DW 20.800 usec
DE 6.50 usec
TE 298.2 K
D1 2.00000000 sec
D11 0.03000000 sec
TD0 1
SFO1 100.6228298 MHz
NUC1 13C
P1 11.00 usec
PLW1 58.00000000 W
SFO2 400.1316005 MHz
NUC2 1H
CPDPRG[2 waltz16
PCPD2 90.00 usec
PLW2 12.00000000 W
PLW12 0.46413001 W
PLW13 0.23345999 W
F2 - Processing parameters
SI 32768
SF 100.6127685 MHz
WDW EM
SSB 0
LB 1.00 Hz
GB 0
PC 1.40

4. HPLC

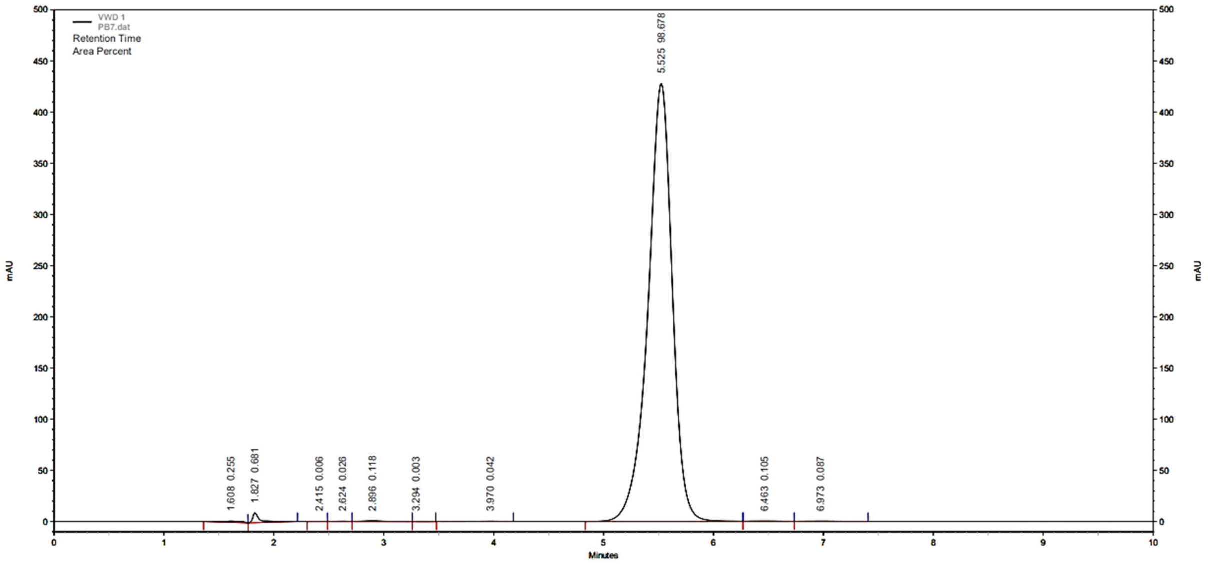

VWD 1
PB7.dat
Retention Time
Area Percent
mAU
Minutes
5.525 98.678
1.608 0.255
1.827 0.681
2.415 0.006
2.624 0.026
2.896 0.118
3.294 0.003
3.970 0.042
6.463 0.105
6.973 0.087

5. Mass

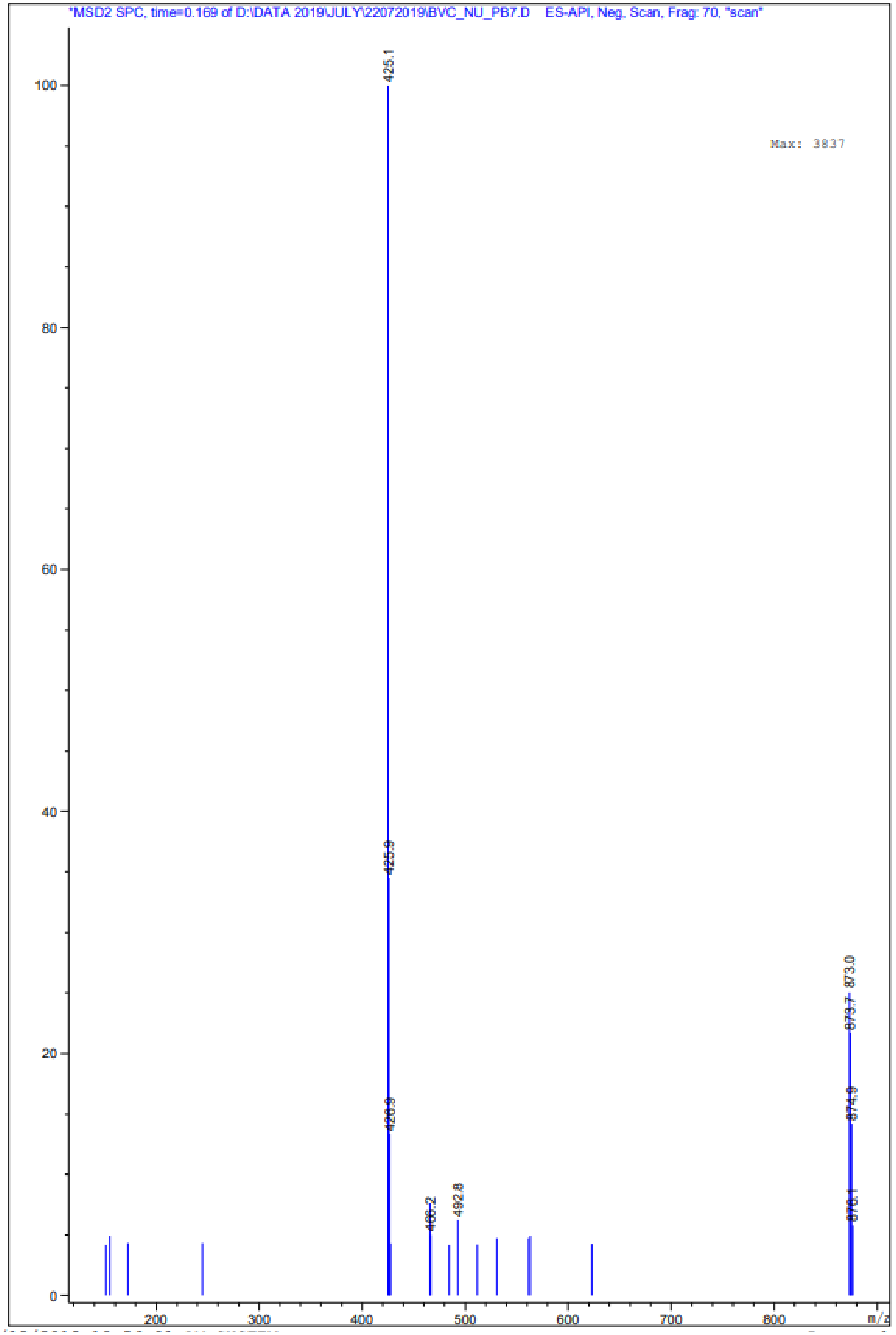

*MSD2 SPC, time=0.169 of D:\DATA 2019\JULY\22072019\BVC_NU_PB7.D ES-API, Neg, Scan, Frag: 70, "scan"
Max: 3837
425.1
425.9
426.9
466.2
492.8
873.0
873.7
874.8
876.1
100
80
60
40
20
0
200
300
400
500
600
700
800
m/z

2-(2,4-dioxo-5-(pyridin-2-ylmethylene)thiazolidin-3-yl)-N-(6-ethoxybenzo[d]thiazol-2-yl)acetamide (PB8)

1. FTIR

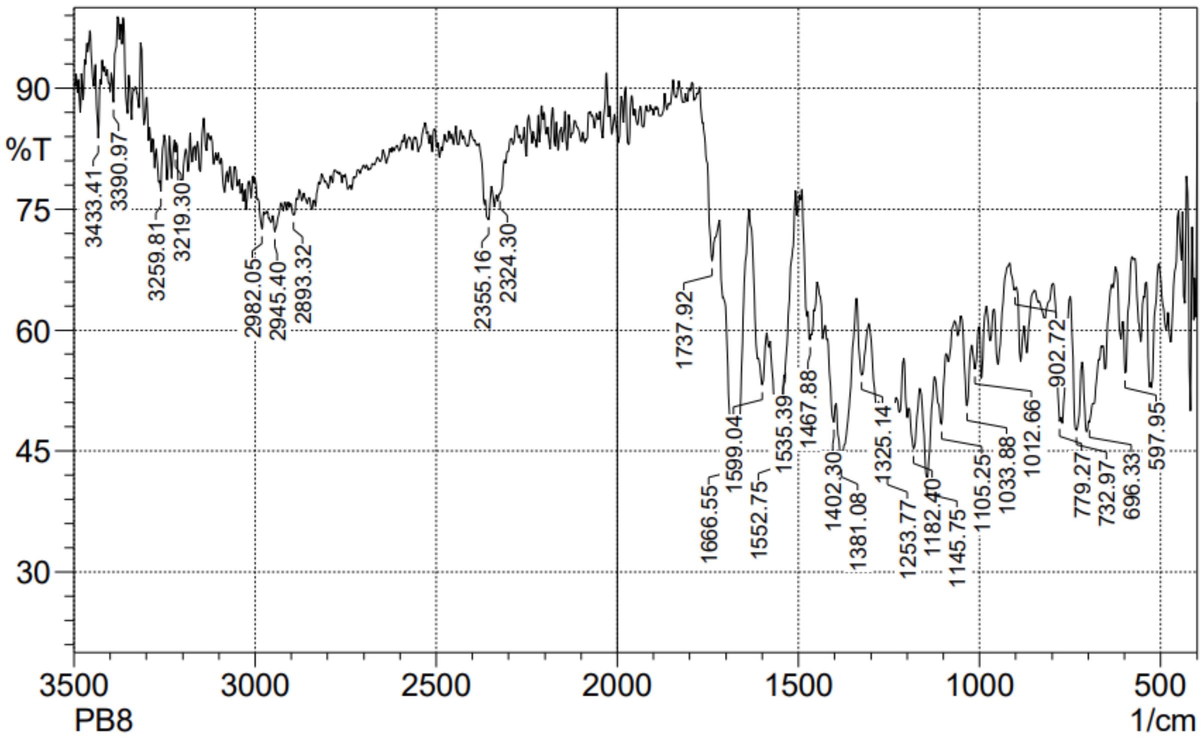

2. 1H-NMR

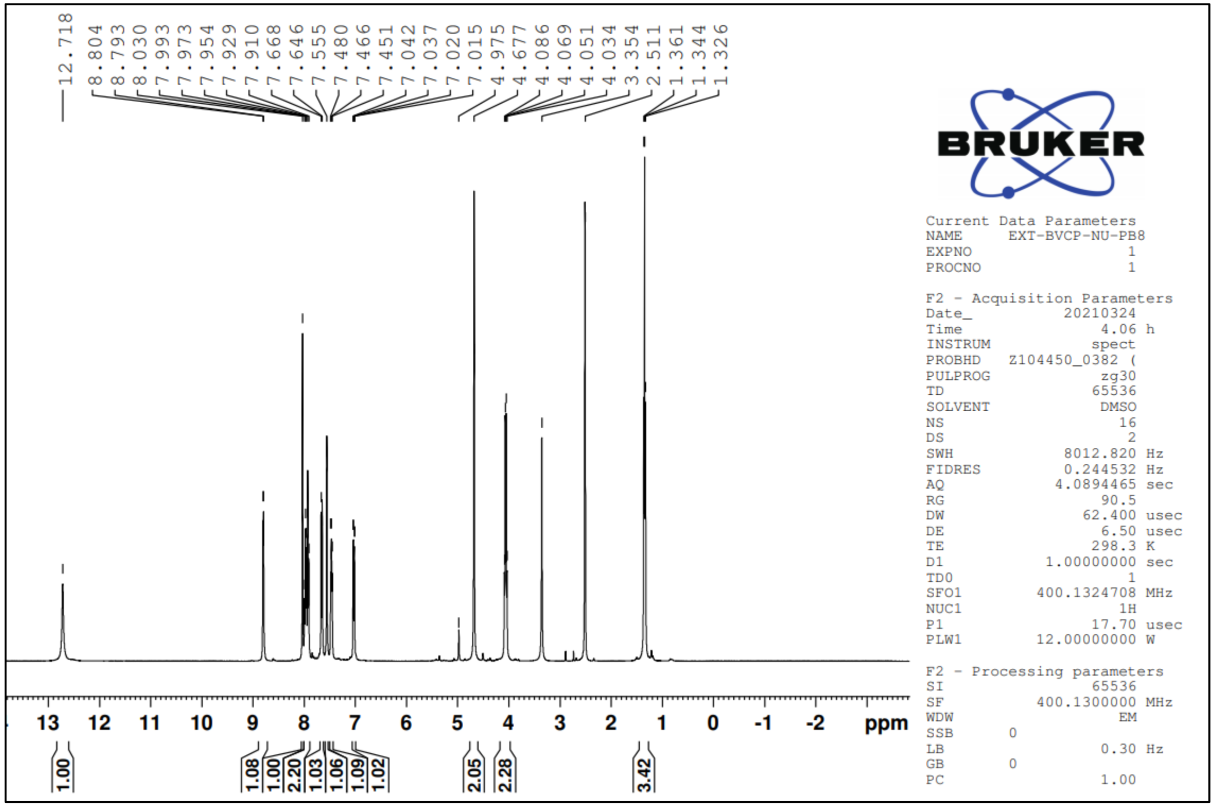

12.718
8.804
8.793
8.030
7.993
7.973
7.954
7.929
7.910
7.668
7.646
7.555
7.480
7.466
7.451
7.042
7.037
7.020
7.015
4.975
4.677
4.086
4.069
4.051
4.034
3.354
2.511
1.361
1.344
1.326
BRUKER
Current Data Parameters
NAME EXT-BVCP-NU-PB8
EXPNO 1
PROCNO 1
F2 - Acquisition Parameters
Date_ 20210324
Time 4.06 h
INSTRUM spect
PROBHD Z104450_0382 (
PULPROG zg30
TD 65536
SOLVENT DMSO
NS 16
DS 2
SWH 8012.820 Hz
FIDRES 0.244532 Hz
AQ 4.0894465 sec
RG 90.5
DW 62.400 usec
DE 6.50 usec
TE 298.3 K
D1 1.00000000 sec
TD0 1
SFO1 400.1324708 MHz
NUC1 1H
P1 17.70 usec
PLW1 12.00000000 W
F2 - Processing parameters
SI 65536
SF 400.1300000 MHz
WDW EM
SSB 0
LB 0.30 Hz
GB 0
PC 1.00
13
12
11
10
9
8
7
6
5
4
3
2
1
0
-1
-2
ppm
1.00
1.08
1.00
2.20
1.03
1.06
1.09
1.02
2.05
2.28
3.42

3. 13C-NMR

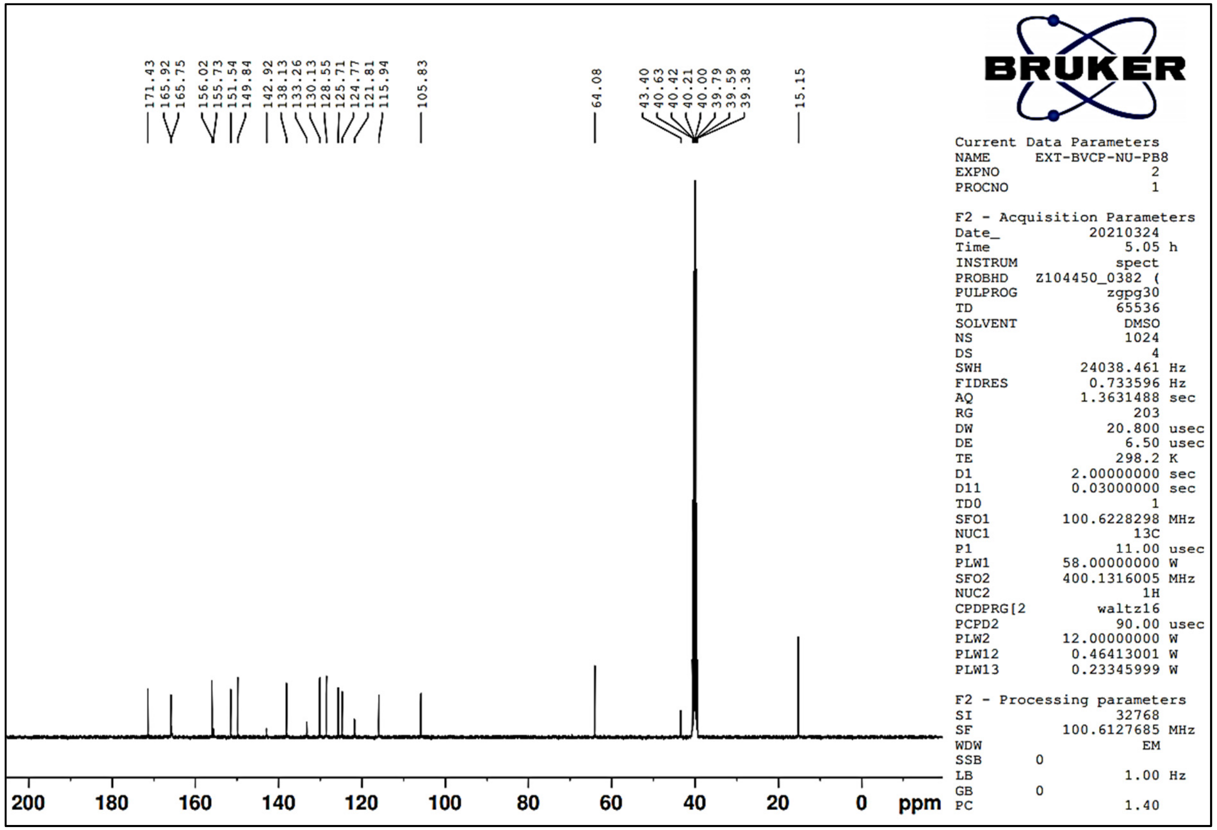
171.43
165.92
165.75
156.02
155.73
151.54
149.84
142.92
138.13
133.26
130.13
128.55
125.71
124.77
121.81
115.94
105.83
64.08
43.40
40.63
40.42
40.21
40.00
39.79
39.59
39.38
15.15
BRUKER
Current Data Parameters
NAME EXT-BVCP-NU-PB8
EXPNO 2
PROCNO 1
F2 - Acquisition Parameters
Date_ 20210324
Time 5.05 h
INSTRUM spect
PROBHD Z104450_0382 (
PULPROG zgpg30
TD 65536
SOLVENT DMSO
NS 1024
DS 4
SWH 24038.461 Hz
FIDRES 0.733596 Hz
AQ 1.3631488 sec
RG 203
DW 20.800 usec
DE 6.50 usec
TE 298.2 K
D1 2.00000000 sec
D11 0.03000000 sec
TD0 1
SFO1 100.6228298 MHz
NUC1 13C
P1 11.00 usec
PLW1 58.00000000 W
SFO2 400.1316005 MHz
NUC2 1H
CPDPRG[2 waltz16
PCPD2 90.00 usec
PLW2 12.00000000 W
PLW12 0.46413001 W
PLW13 0.23345999 W
F2 - Processing parameters
SI 32768
SF 100.6127685 MHz
WDW EM
SSB 0
LB 1.00 Hz
GB 0
PC 1.40
200
180
160
140
120
100
80
60
40
20
0
ppm

4. HPLC

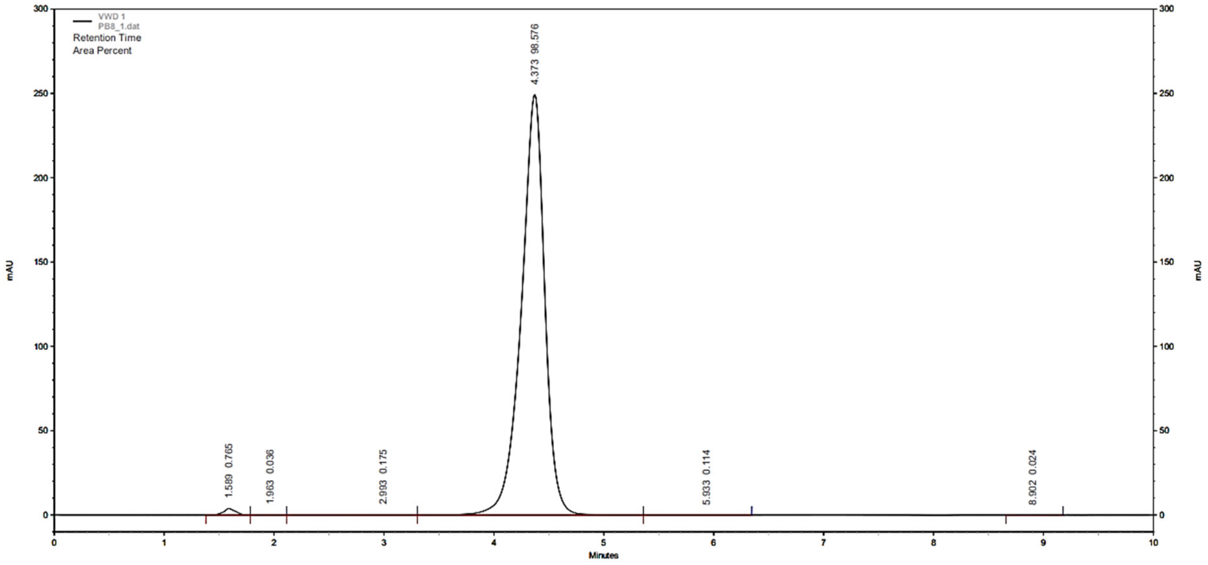
VWD 1
PB8_1.dat
Retention Time
Area Percent
4.373 98.576
1.589 0.765
1.963 0.036
2.993 0.175
5.933 0.114
8.902 0.024
mAU
Minutes

5. Mass

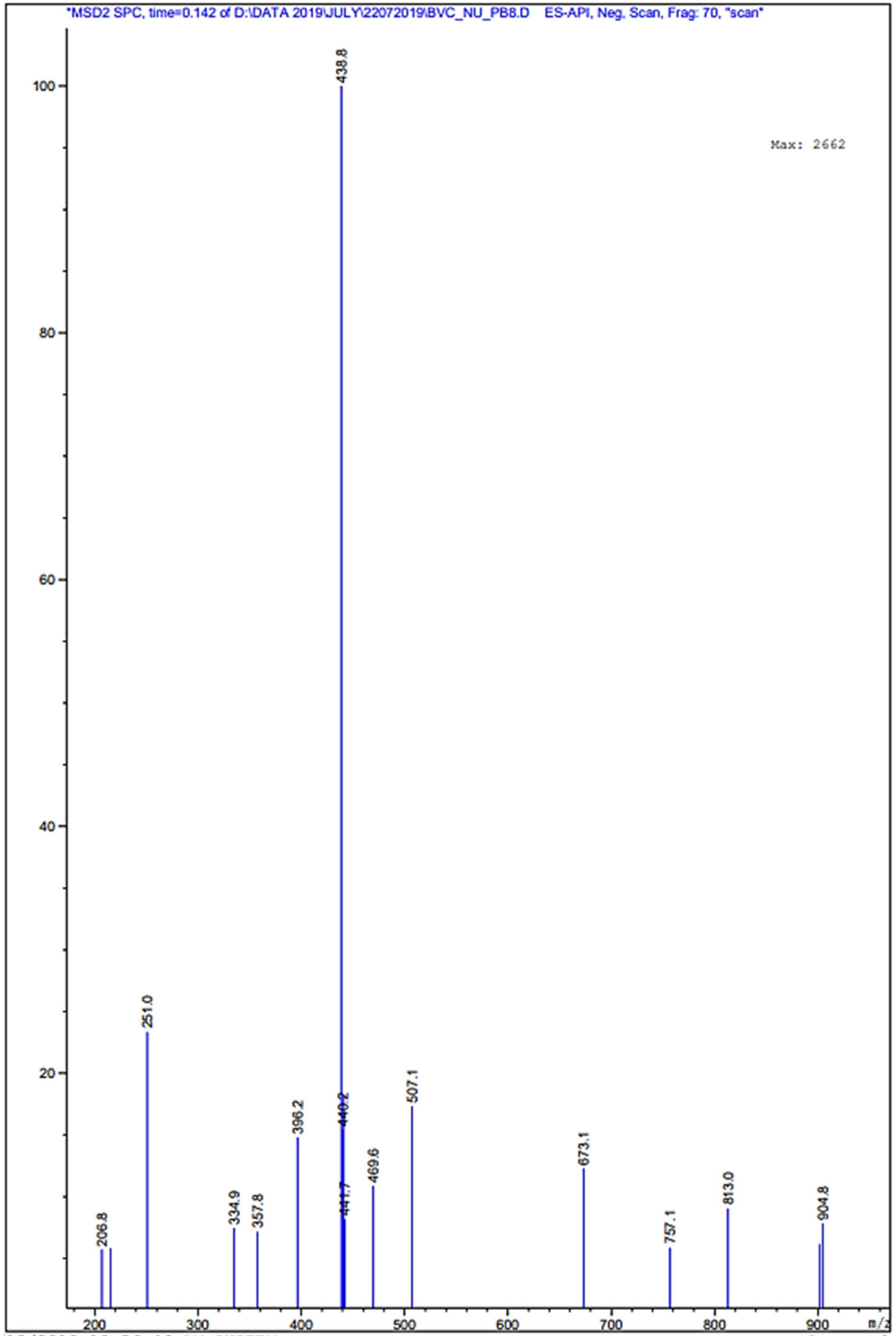

*MSD2 SPC, time=0.142 of D:\DATA 2019\JULY\22072019\BVC_NU_PB8.D ES-API, Neg, Scan, Frag: 70, "scan"
Max: 2662
438.8
251.0
507.1
396.2
440.2
469.6
673.1
441.7
813.0
904.8
334.9
357.8
206.8
757.1
m/z

2-(2,4-dioxo-5-(pyridin-2-ylmethylene)thiazolidin-3-yl)-N-(6-fluorobenzo[d]thiazol-2-yl)acetamide (PB9)

1. FTIR

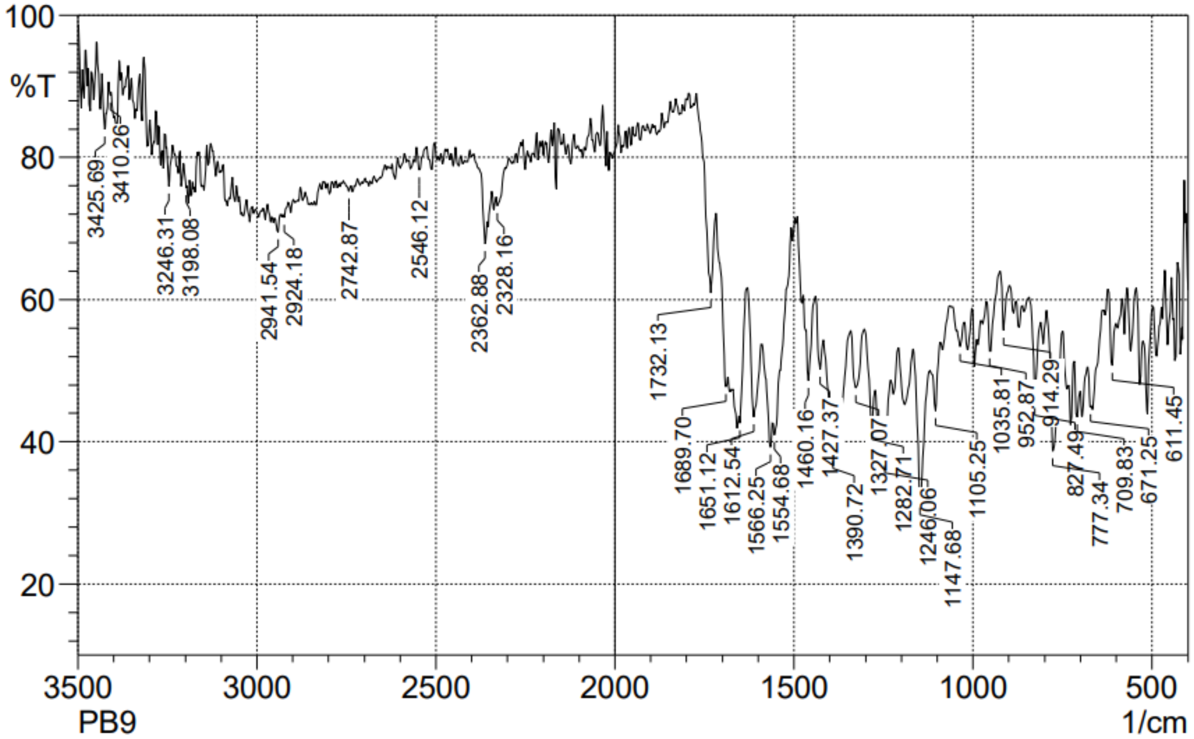

2. 1H-NMR

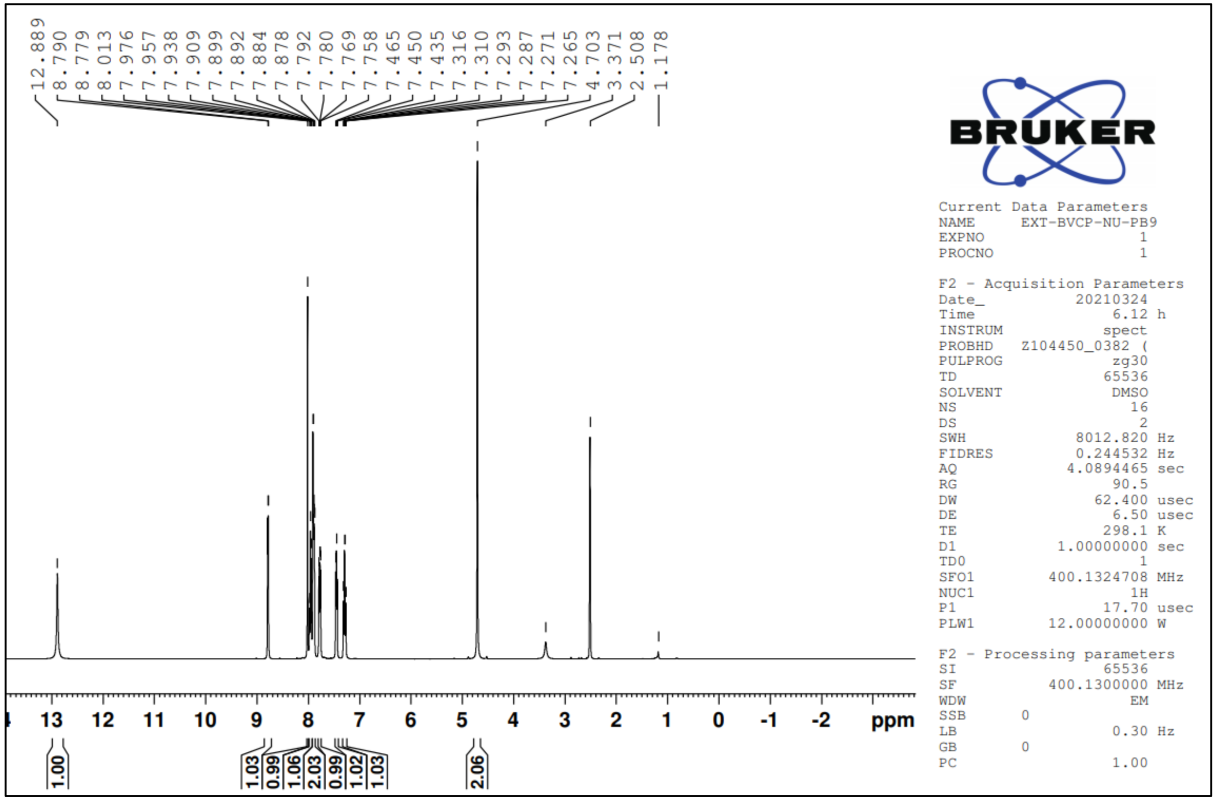
12.889
8.790
8.779
8.013
7.976
7.957
7.938
7.909
7.899
7.892
7.884
7.878
7.792
7.780
7.769
7.758
7.465
7.450
7.435
7.316
7.310
7.293
7.287
7.271
7.265
4.703
3.371
2.508
1.178
BRUKER
Current Data Parameters
NAME EXT-BVCP-NU-PB9
EXPNO 1
PROCNO 1
F2 - Acquisition Parameters
Date_ 20210324
Time 6.12 h
INSTRUM spect
PROBHD Z104450_0382 (
PULPROG zg30
TD 65536
SOLVENT DMSO
NS 16
DS 2
SWH 8012.820 Hz
FIDRES 0.244532 Hz
AQ 4.0894465 sec
RG 90.5
DW 62.400 usec
DE 6.50 usec
TE 298.1 K
D1 1.00000000 sec
TD0 1
SFO1 400.1324708 MHz
NUC1 1H
P1 17.70 usec
PLW1 12.00000000 W
F2 - Processing parameters
SI 65536
SF 400.1300000 MHz
WDW EM
SSB 0
LB 0.30 Hz
GB 0
PC 1.00
13 12 11 10 9 8 7 6 5 4 3 2 1 0 -1 -2 ppm
1.00
1.03
0.99
1.06
2.03
0.99
1.02
1.03
2.06

3. 13C-NMR

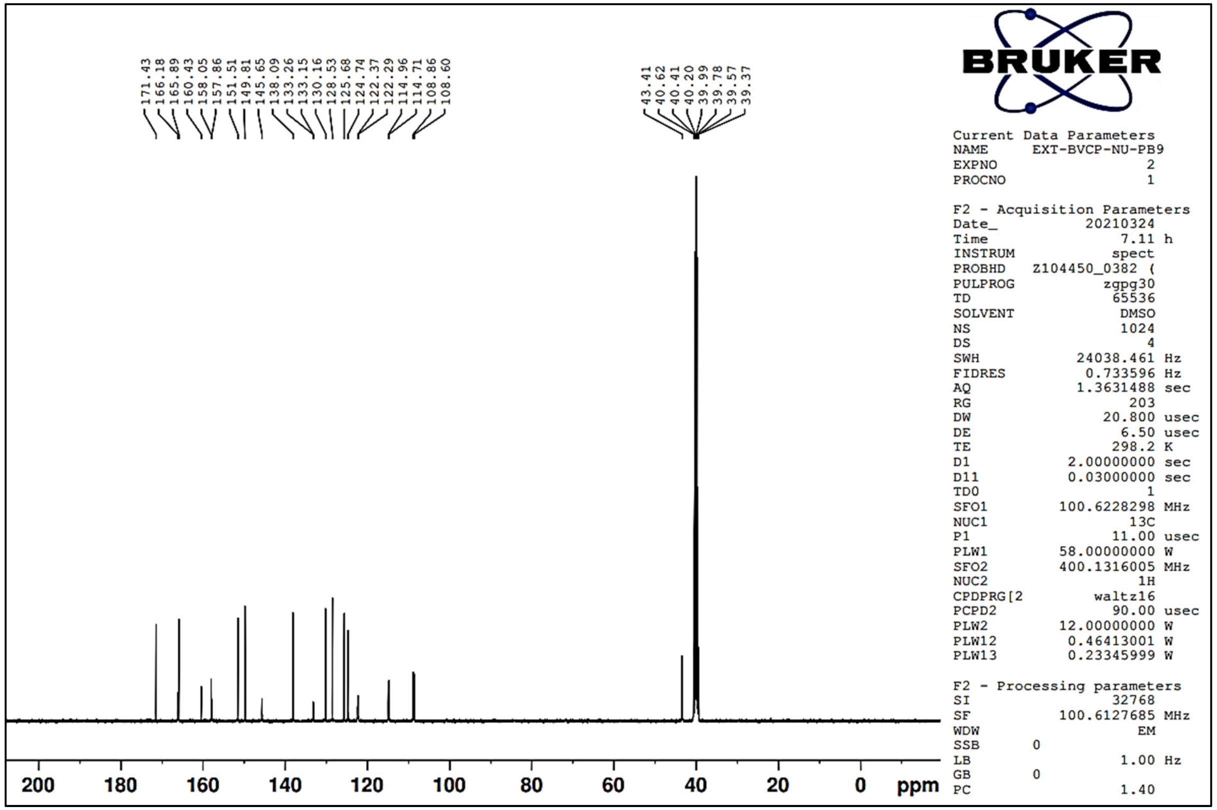
171.43
166.18
165.89
160.43
158.05
157.86
151.51
149.81
145.65
138.09
133.26
133.15
130.16
128.53
125.68
124.74
122.37
122.29
114.96
114.71
108.86
108.60
43.41
40.62
40.41
40.20
39.99
39.78
39.57
39.37
BRUKER
Current Data Parameters
NAME EXT-BVCP-NU-PB9
EXPNO 2
PROCNO 1
F2 - Acquisition Parameters
Date_ 20210324
Time 7.11 h
INSTRUM spect
PROBHD Z104450_0382 (
PULPROG zgpg30
TD 65536
SOLVENT DMSO
NS 1024
DS 4
SWH 24038.461 Hz
FIDRES 0.733596 Hz
AQ 1.3631488 sec
RG 203
DW 20.800 usec
DE 6.50 usec
TE 298.2 K
D1 2.00000000 sec
D11 0.03000000 sec
TD0 1
SFO1 100.6228298 MHz
NUC1 13C
P1 11.00 usec
PLW1 58.00000000 W
SFO2 400.1316005 MHz
NUC2 1H
CPDPRG[2 waltz16
PCPD2 90.00 usec
PLW2 12.00000000 W
PLW12 0.46413001 W
PLW13 0.23345999 W
F2 - Processing parameters
SI 32768
SF 100.6127685 MHz
WDW EM
SSB 0
LB 1.00 Hz
GB 0
PC 1.40
200
180
160
140
120
100
80
60
40
20
0
ppm

4. HPLC

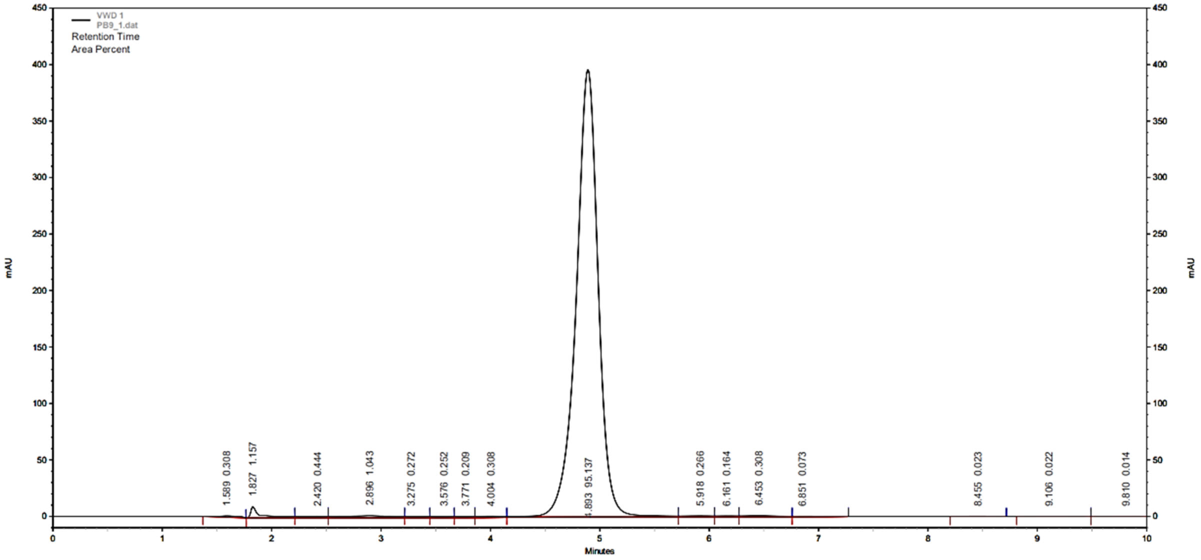
VWD 1
PB9_1.dat
Retention Time
Area Percent
mAU
Minutes
1.589 0.308
1.827 1.157
2.420 0.444
2.896 1.043
3.275 0.272
3.576 0.252
3.771 0.209
4.004 0.308
4.893 95.137
5.918 0.266
6.161 0.164
6.453 0.308
6.851 0.073
8.455 0.023
9.106 0.022
9.810 0.014

5. Mass

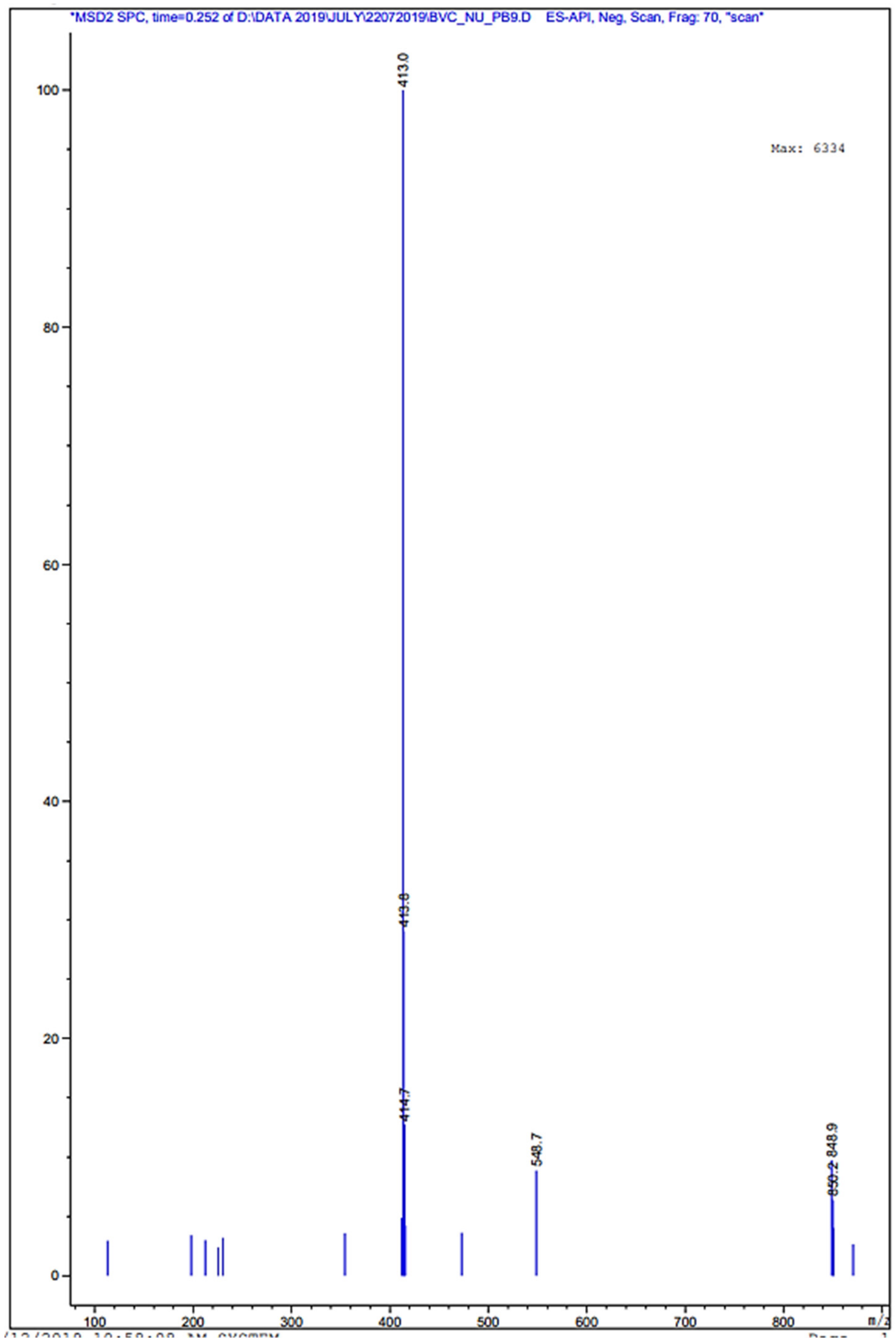
"MSD2 SPC, time=0.252 of D:\DATA 2019\JULY\22072019\BVC_NU_PB9.D ES-API, Neg, Scan, Frag: 70, "scan"
Max: 6334
413.0
413.8
414.7
548.7
850.2
848.9
m/z

2-(5-benzylidene-2,4-dioxo thiazolidine-3-yl)-N-(4-chloro benzo[d]thiazol-2-yl)acetamide (GB1)

1. FTIR

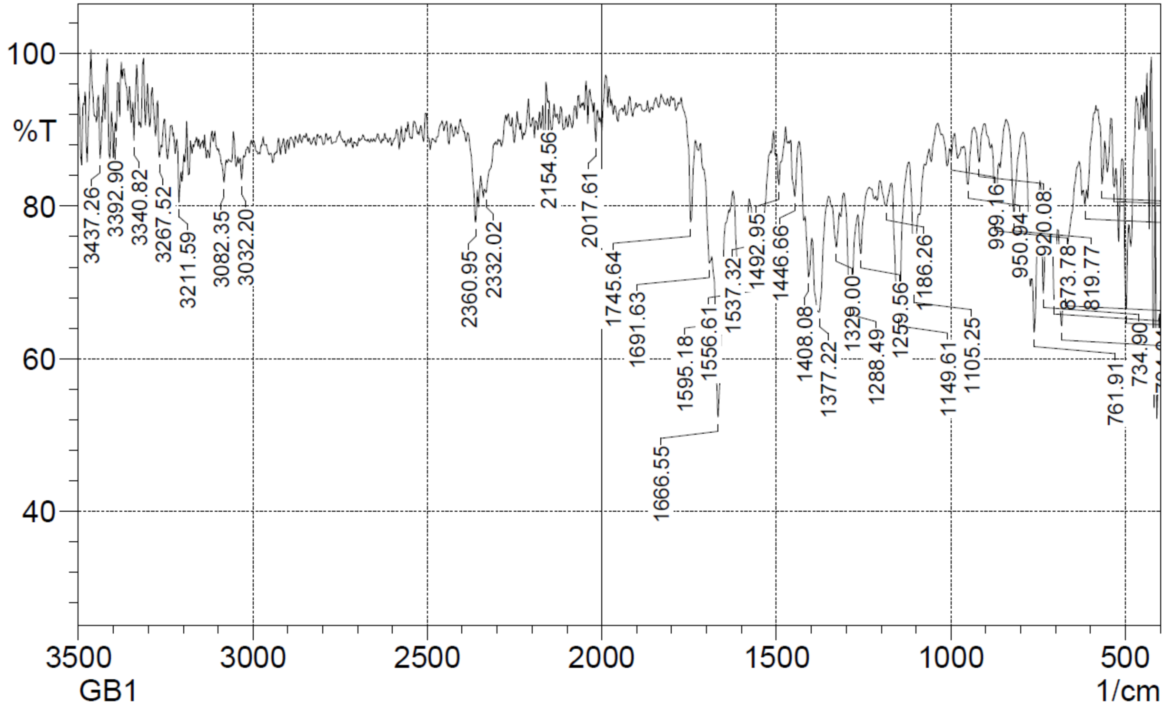

2. 1H-NMR

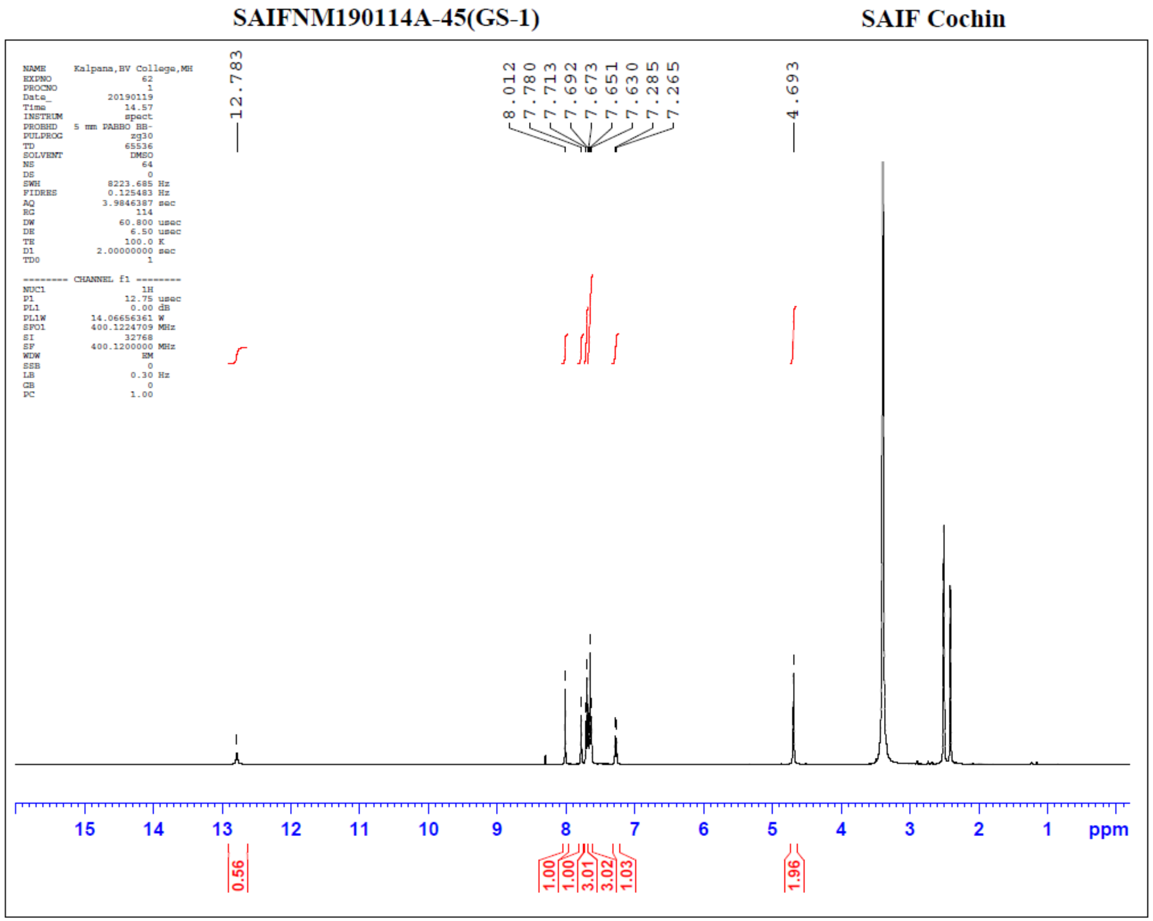
SAIFNM190114A-45(GS-1)
SAIF Cochin
NAME Kalpana,BV College,MH
EXPNO 62
PROCNO 1
Date_ 20190119
Time 14.57
INSTRUM spect
PROBHD 5 mm PABBO BB-
PULPROG zg30
TD 65536
SOLVENT DMSO
NS 64
DS 0
SWH 8223.685 Hz
FIDRES 0.125483 Hz
AQ 3.9846387 sec
RG 114
DW 60.800 usec
DE 6.50 usec
TE 100.0 K
D1 2.00000000 sec
TD0 1
======== CHANNEL f1 ========
NUC1 1H
P1 12.75 usec
PL1 0.00 dB
PL1W 14.06656361 W
SFO1 400.1224709 MHz
SI 32768
SF 400.1200000 MHz
WDW EM
SSB 0
LB 0.30 Hz
GB 0
PC 1.00
12.783
8.012
7.780
7.713
7.692
7.673
7.651
7.630
7.285
7.265
4.693
15
14
13
12
11
10
9
8
7
6
5
4
3
2
1
ppm
0.56
1.00
1.00
3.01
3.02
1.03
1.96

3. 13C-NMR

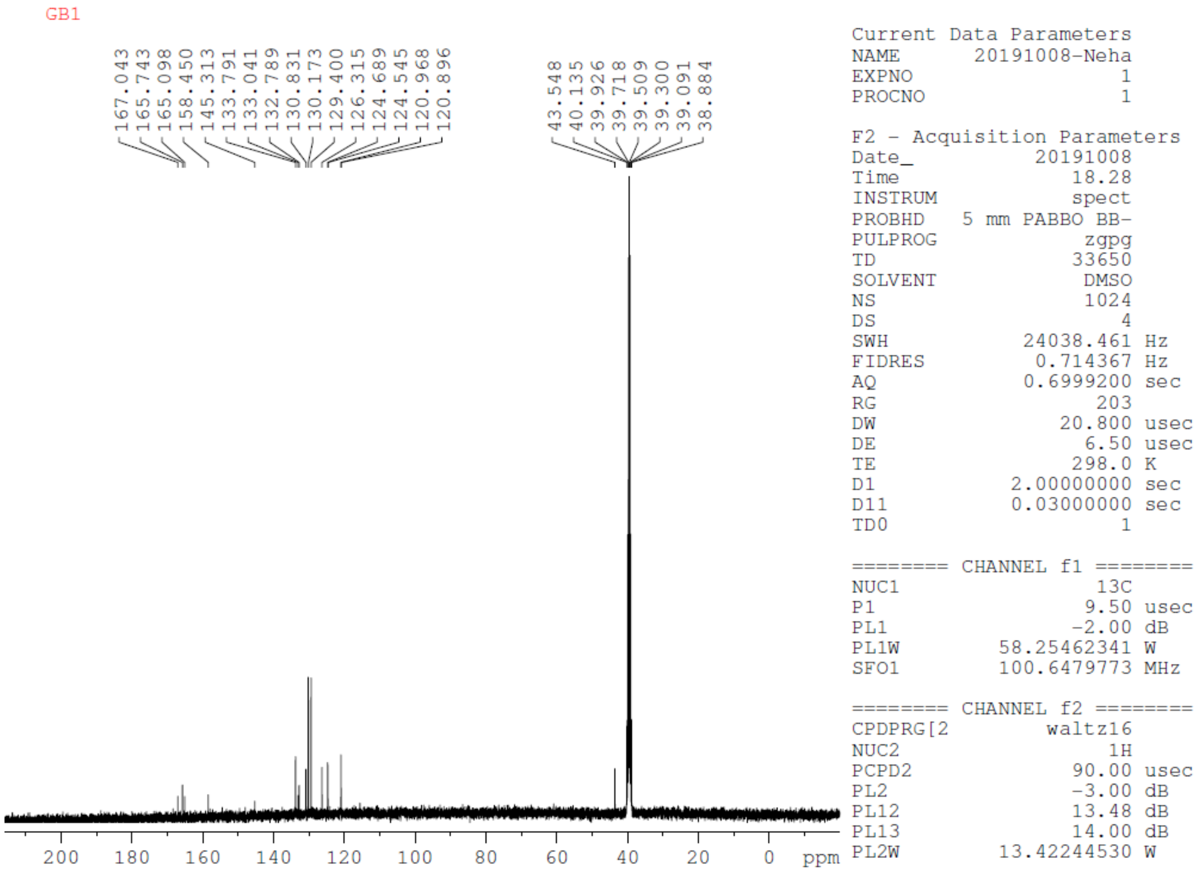
GB1
167.043
165.743
165.098
158.450
145.313
133.791
133.041
132.789
130.831
130.173
129.400
126.315
124.689
124.545
120.968
120.896
43.548
40.135
39.926
39.718
39.509
39.300
39.091
38.884
200 180 160 140 120 100 80 60 40 20 0 ppm
Current Data Parameters
NAME 20191008-Neha
EXPNO 1
PROCNO 1
F2 - Acquisition Parameters
Date_ 20191008
Time 18.28
INSTRUM spect
PROBHD 5 mm PABBO BB-
PULPROG zgpg
TD 33650
SOLVENT DMSO
NS 1024
DS 4
SWH 24038.461 Hz
FIDRES 0.714367 Hz
AQ 0.6999200 sec
RG 203
DW 20.800 usec
DE 6.50 usec
TE 298.0 K
D1 2.00000000 sec
D11 0.03000000 sec
TD0 1
======== CHANNEL f1 ========
NUC1 13C
P1 9.50 usec
PL1 -2.00 dB
PL1W 58.25462341 W
SFO1 100.6479773 MHz
======== CHANNEL f2 ========
CPDPRG[2 waltz16
NUC2 1H
PCPD2 90.00 usec
PL2 -3.00 dB
PL12 13.48 dB
PL13 14.00 dB
PL2W 13.42244530 W

4. HPLC

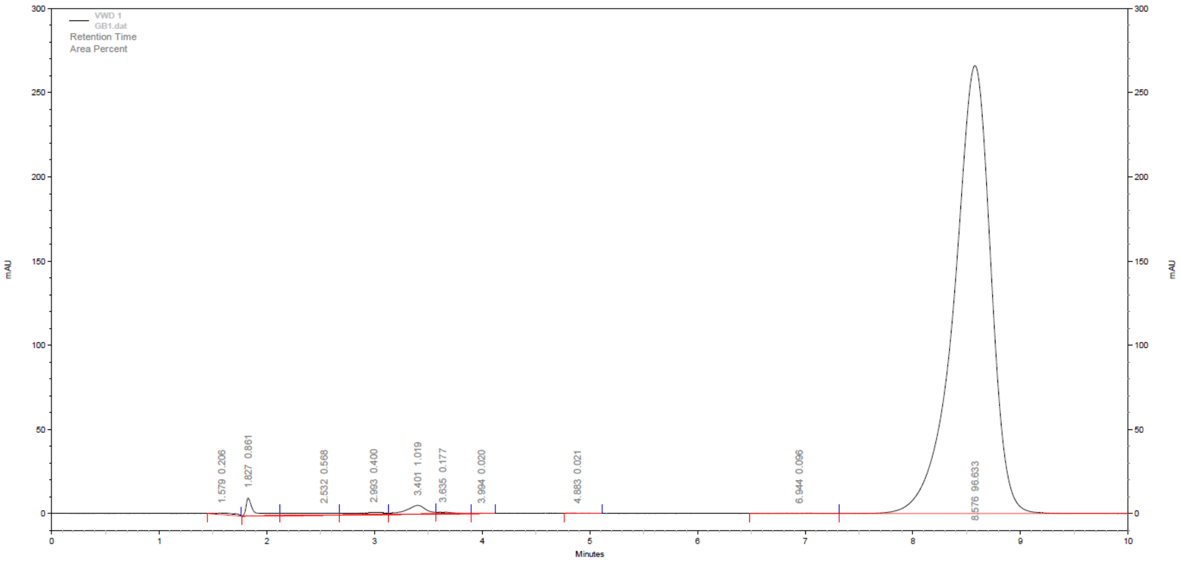
VWD 1
GB1.dat
Retention Time
Area Percent
mAU
Minutes
1.579 0.206
1.827 0.861
2.532 0.568
2.993 0.400
3.401 1.019
3.635 0.177
3.994 0.020
4.883 0.021
6.944 0.096
8.576 96.633

5. Mass

MS Spectrum

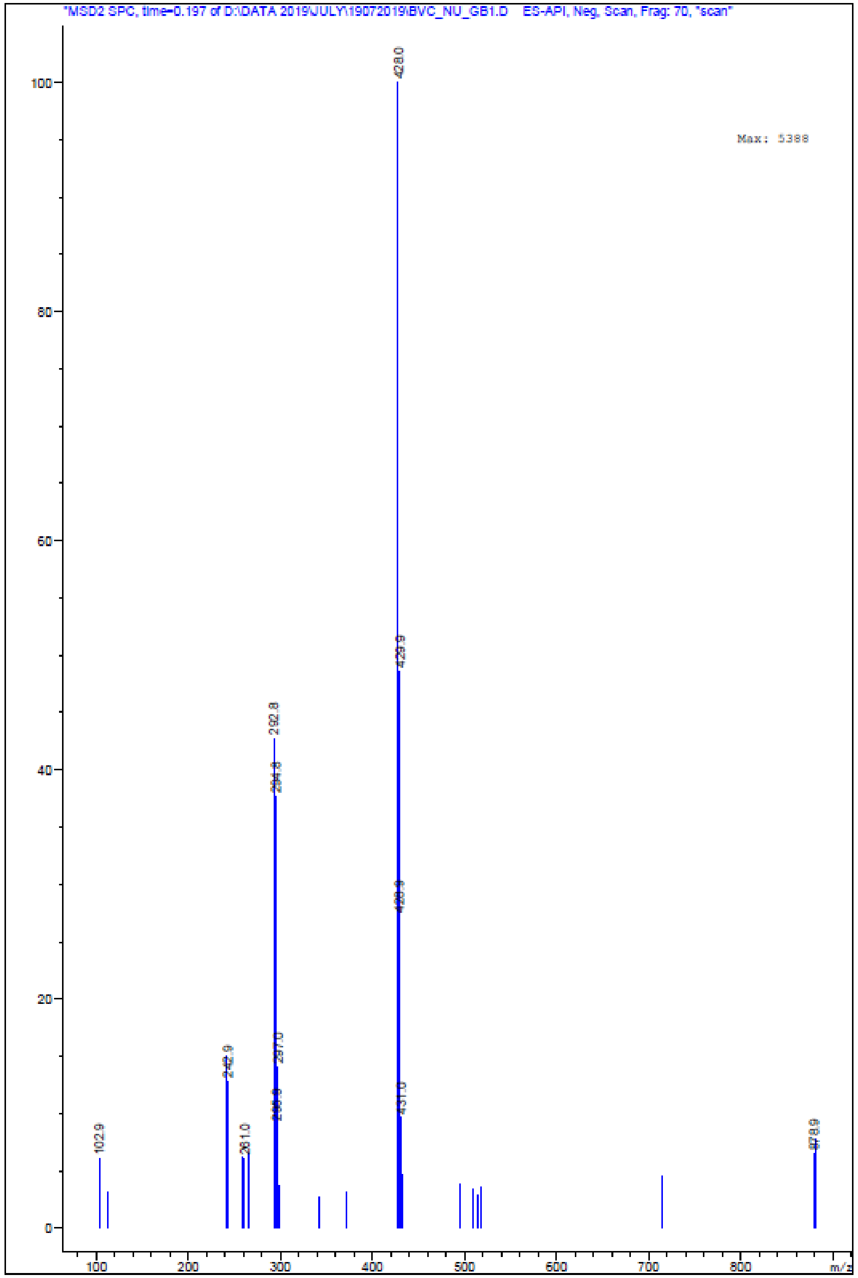
"MSD2 SPC, time=0.197 of D:\DATA 2019\JULY\19072019\BVC_NU_GB1.D ES-API, Neg, Scan, Frag: 70, "scan"
Max: 5388
428.0
429.9
292.8
294.8
242.9
297.0
431.0
102.9
261.0
878.9
m/z

N-(4-chlorobenzo[d]thiazol-2-yl)-2-(5-(4-methylbenzylidene)-2,4-dioxothiazolidin-3-yl)acetamide (GB2)

1. FTIR

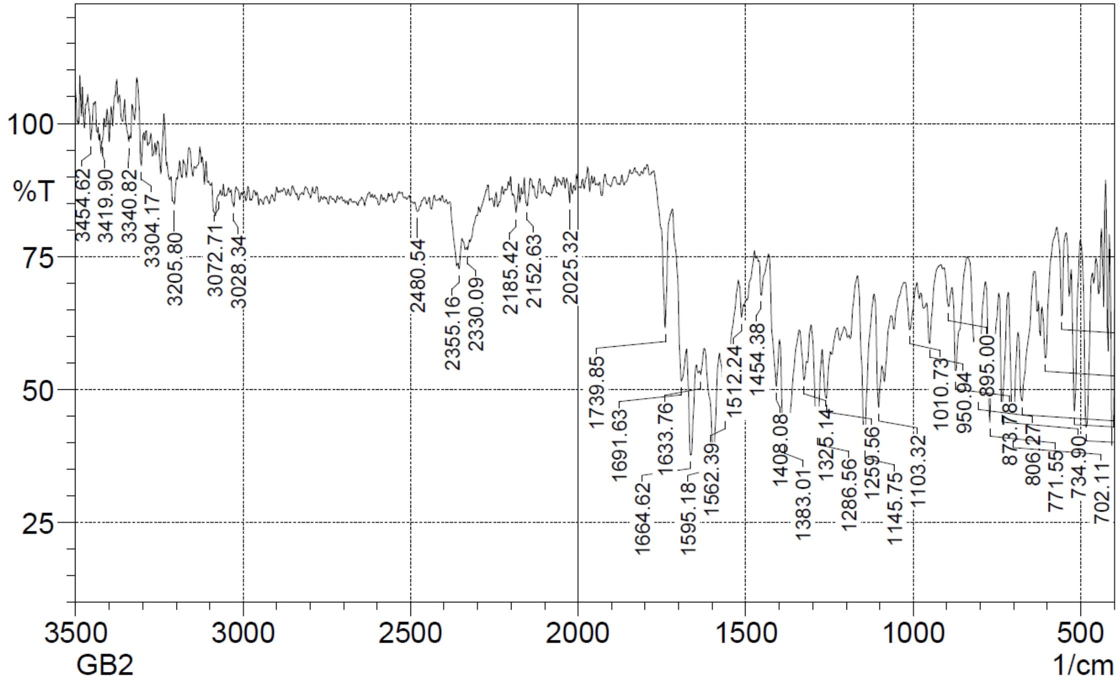

2. 1H-NMR

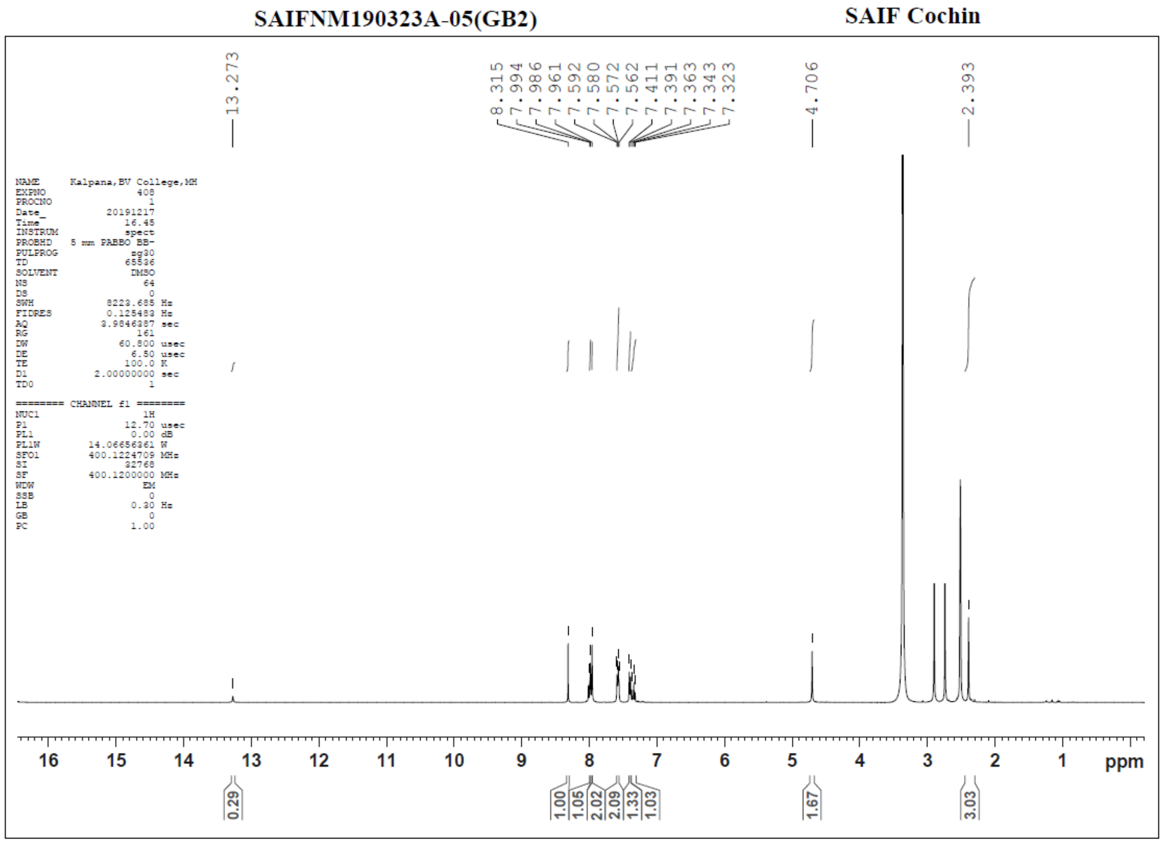
SAIFNM190323A-05(GB2)
SAIF Cochin
13.273
8.315
7.994
7.986
7.961
7.592
7.580
7.572
7.562
7.411
7.391
7.363
7.343
7.323
4.706
2.393
NAME Kalpana,BV College,MH
EXPNO 408
PROCNO 1
Date_ 20191217
Time 16.45
INSTRUM spect
PROBHD 5 mm PABBO BB-
PULPROG zg30
TD 65536
SOLVENT DMSO
NS 64
DS 0
SWH 8223.685 Hz
FIDRES 0.125483 Hz
AQ 3.9846387 sec
RG 161
DW 60.800 usec
DE 6.50 usec
TE 100.0 K
D1 2.00000000 sec
TD0 1
======== CHANNEL f1 ========
NUC1 1H
P1 12.70 usec
PL1 0.00 dB
PL1W 14.06656361 W
SFO1 400.1224709 MHz
SI 32768
SF 400.1200000 MHz
WDW EM
SSB 0
LB 0.30 Hz
GB 0
PC 1.00
16 15 14 13 12 11 10 9 8 7 6 5 4 3 2 1 ppm
0.29
1.00
1.05
2.02
2.09
1.33
1.03
1.67
3.03

3. 13C-NMR

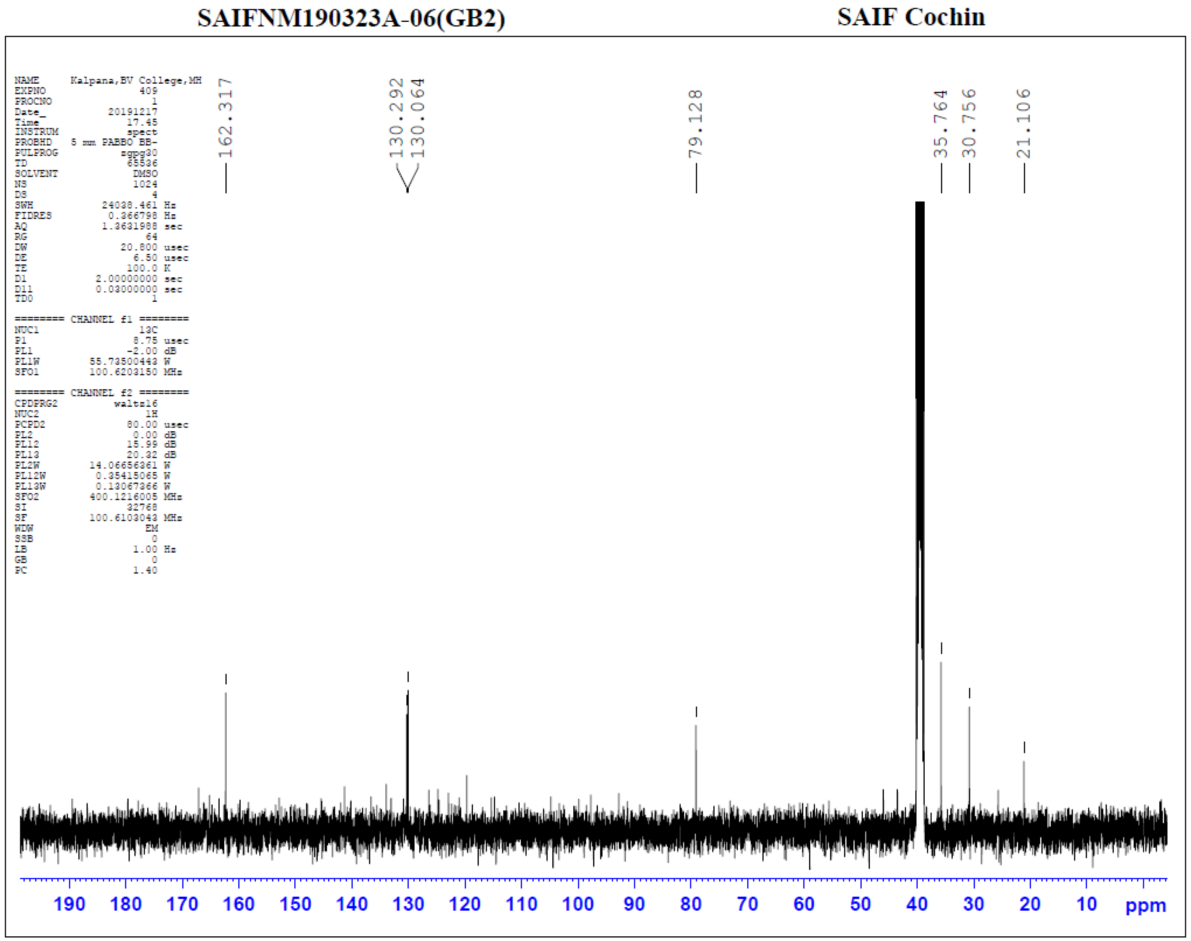
SAIFNM190323A-06(GB2)
SAIF Cochin
NAME Kalpana,BV College,MH
EXPNO 409
PROCNO 1
Date_ 20191217
Time 17.45
INSTRUM spect
PROBHD 5 mm PABBO BB-
PULPROG zgpg30
TD 65536
SOLVENT DMSO
NS 1024
DS 4
SWH 24038.461 Hz
FIDRES 0.366798 Hz
AQ 1.3631988 sec
RG 64
DW 20.800 usec
DE 6.50 usec
TE 100.0 K
D1 2.00000000 sec
D11 0.03000000 sec
TD0 1
======== CHANNEL f1 ========
NUC1 13C
P1 8.75 usec
PL1 -2.00 dB
PL1W 55.73500443 W
SFO1 100.6203150 MHz
======== CHANNEL f2 ========
CPDPRG2 waltz16
NUC2 1H
PCPD2 80.00 usec
PL2 0.00 dB
PL12 15.99 dB
PL13 20.32 dB
PL2W 14.06656361 W
PL12W 0.35415065 W
PL13W 0.13067366 W
SFO2 400.1216005 MHz
SI 32768
SF 100.6103043 MHz
WDW EM
SSB 0
LB 1.00 Hz
GB 0
PC 1.40
162.317
130.292
130.064
79.128
35.764
30.756
21.106
190 180 170 160 150 140 130 120 110 100 90 80 70 60 50 40 30 20 10 ppm

4. HPLC

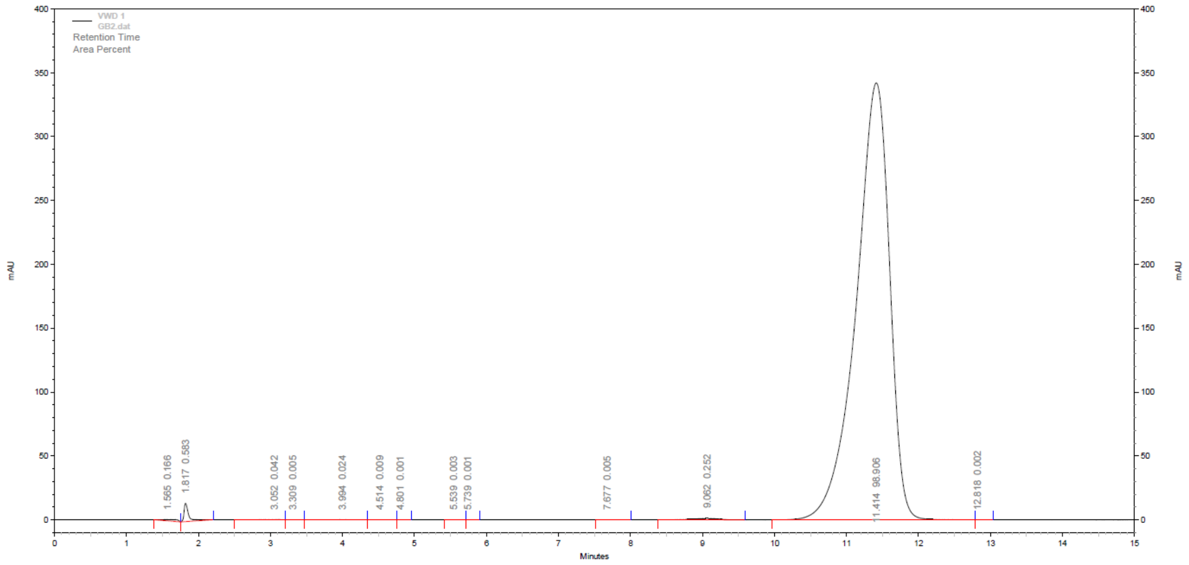
VWD 1
GB2.dat
Retention Time
Area Percent
mAU
Minutes
1.565 0.166
1.817 0.583
3.052 0.042
3.309 0.005
3.994 0.024
4.514 0.009
4.801 0.001
5.539 0.003
5.739 0.001
7.677 0.005
9.062 0.252
11.414 98.906
12.818 0.002

5. Mass

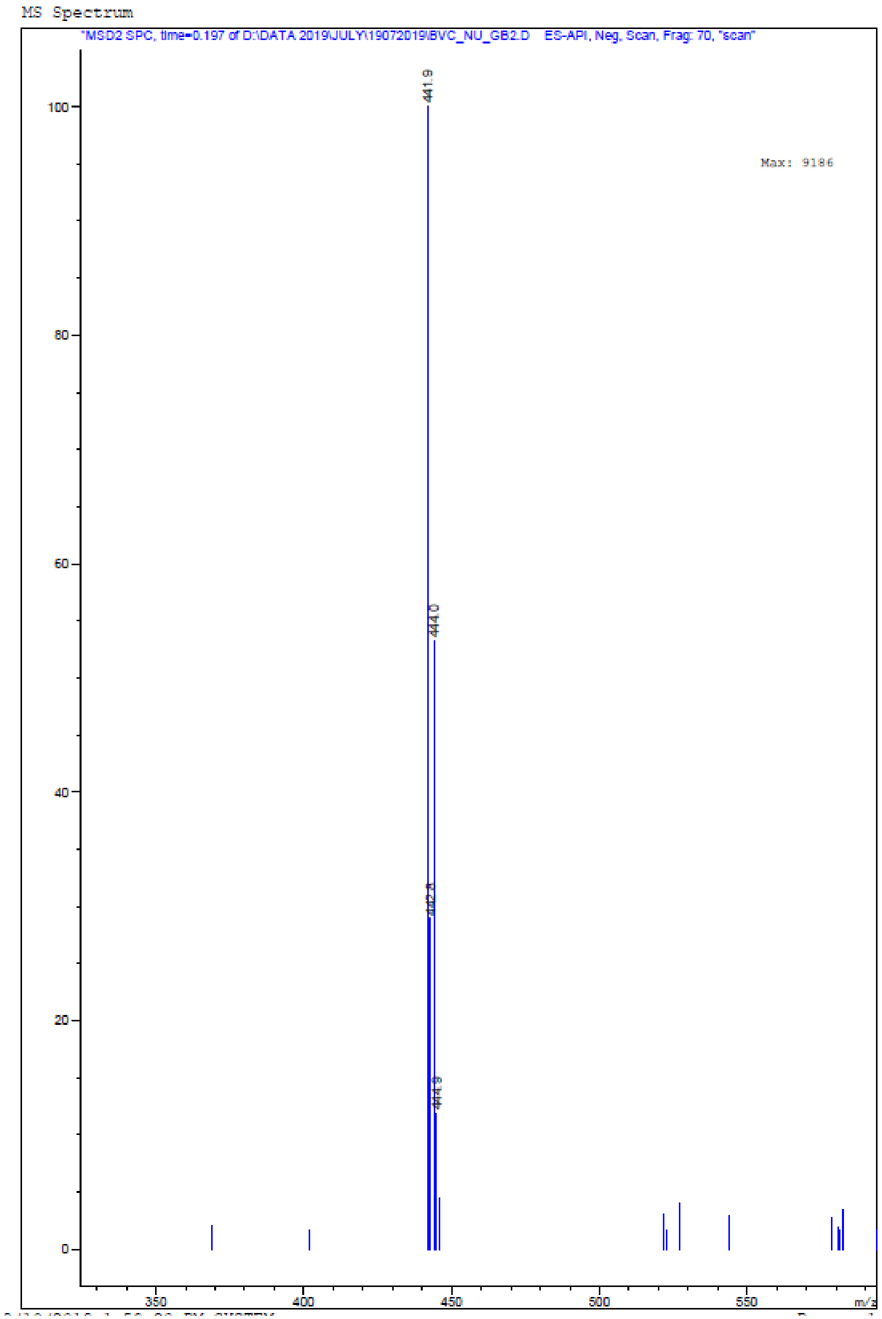

MS Spectrum
"MSD2 SPC, time=0.197 of D:\DATA 2019\JULY\19072019\BVC_NU_GB2.D ES-API, Neg, Scan, Frag: 70, "scan"
Max: 9186
441.9
444.0
m/z

N-(4-chlorobenzo[d]thiazol-2-yl)-2-(5-(4-chlorobenzylidene)-2,4-dioxothiazolidin-3-yl)acetamide (GB3)

1. FTIR -

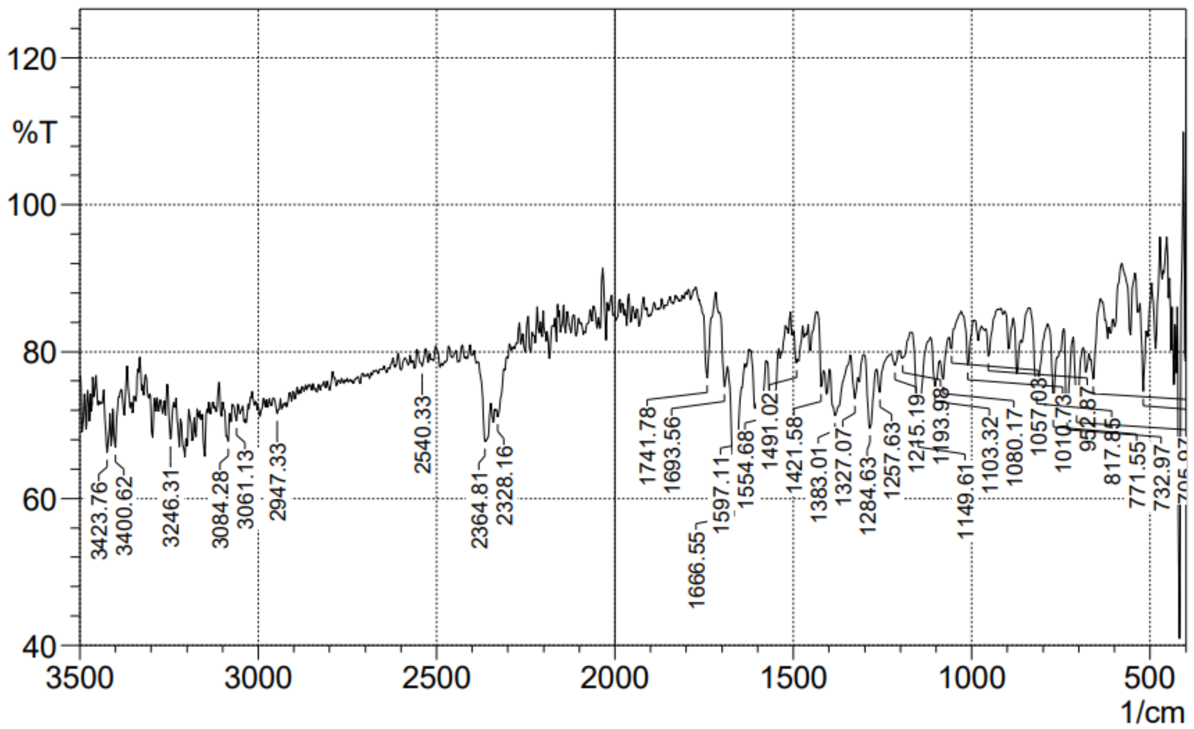

2. ¹H-NMR

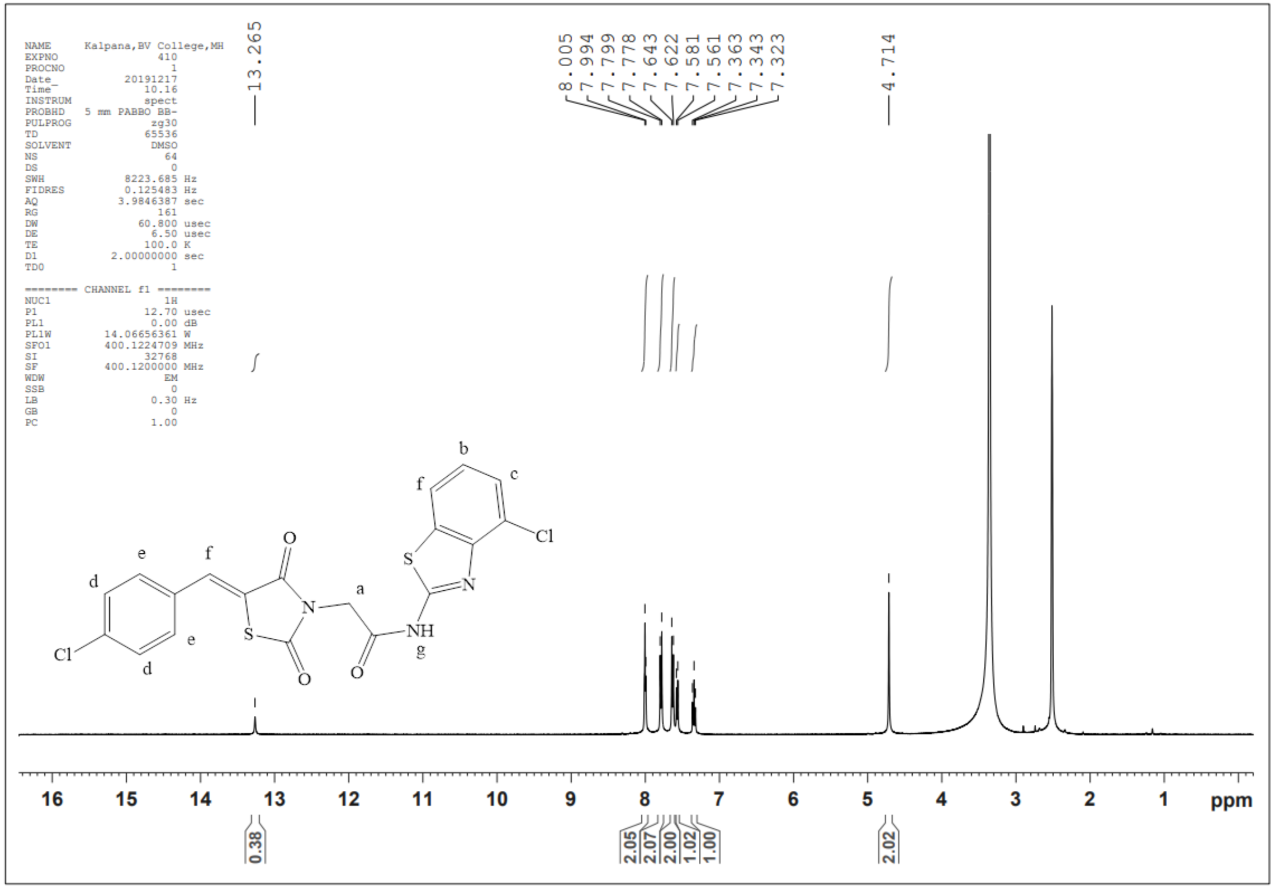
NAME Kalpana,BV College,MH
EXPNO 410
PROCNO 1
Date_ 20191217
Time 10.16
INSTRUM spect
PROBHD 5 mm PABBO BB-
PULPROG zg30
TD 65536
SOLVENT DMSO
NS 64
DS 0
SWH 8223.685 Hz
FIDRES 0.125483 Hz
AQ 3.9846387 sec
RG 161
DW 60.800 usec
DE 6.50 usec
TE 100.0 K
D1 2.00000000 sec
TD0 1
======== CHANNEL f1 ========
NUC1 1H
P1 12.70 usec
PL1 0.00 dB
PL1W 14.06656361 W
SFO1 400.1224709 MHz
SI 32768
SF 400.1200000 MHz
WDW EM
SSB 0
LB 0.30 Hz
GB 0
PC 1.00
13.265
8.005
7.994
7.799
7.778
7.643
7.622
7.581
7.561
7.363
7.343
7.323
4.714
ppm
0.38
2.05
2.07
2.00
2.00
1.02
1.00
2.02

3. ^{13}C-NMR

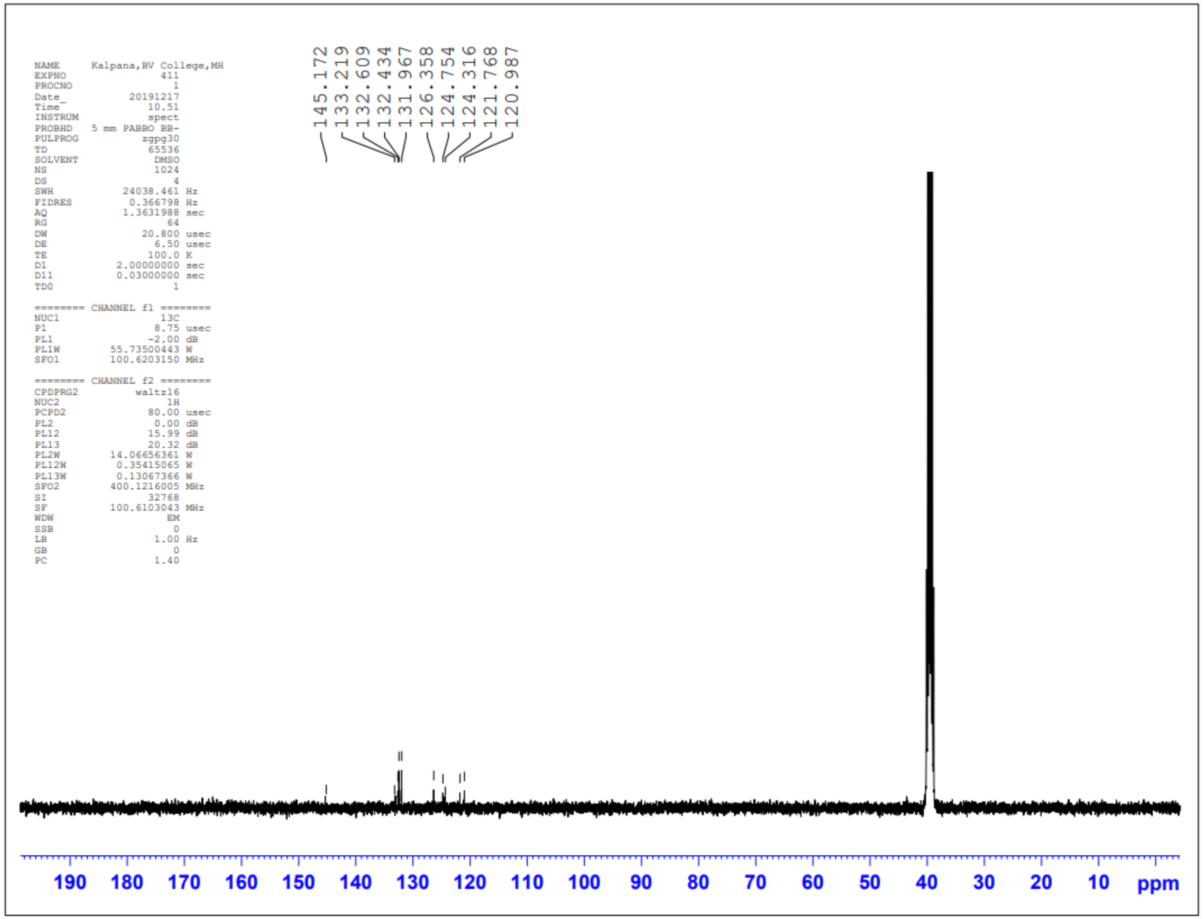

4. HPLC Analysis

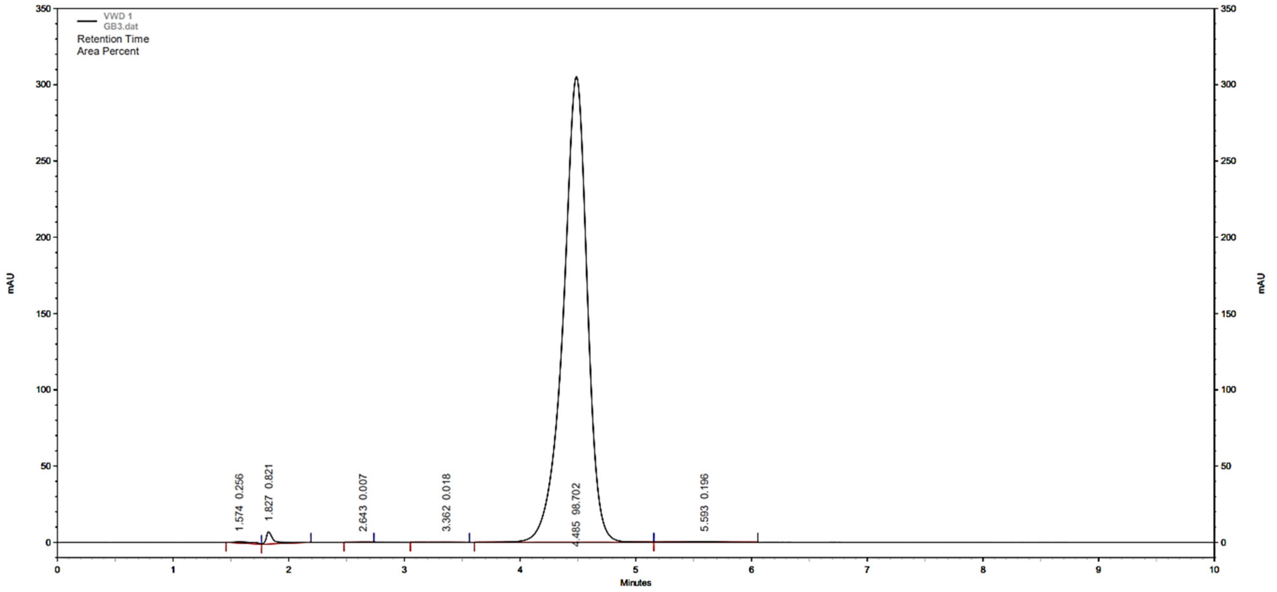

5. Mass Spectrometry

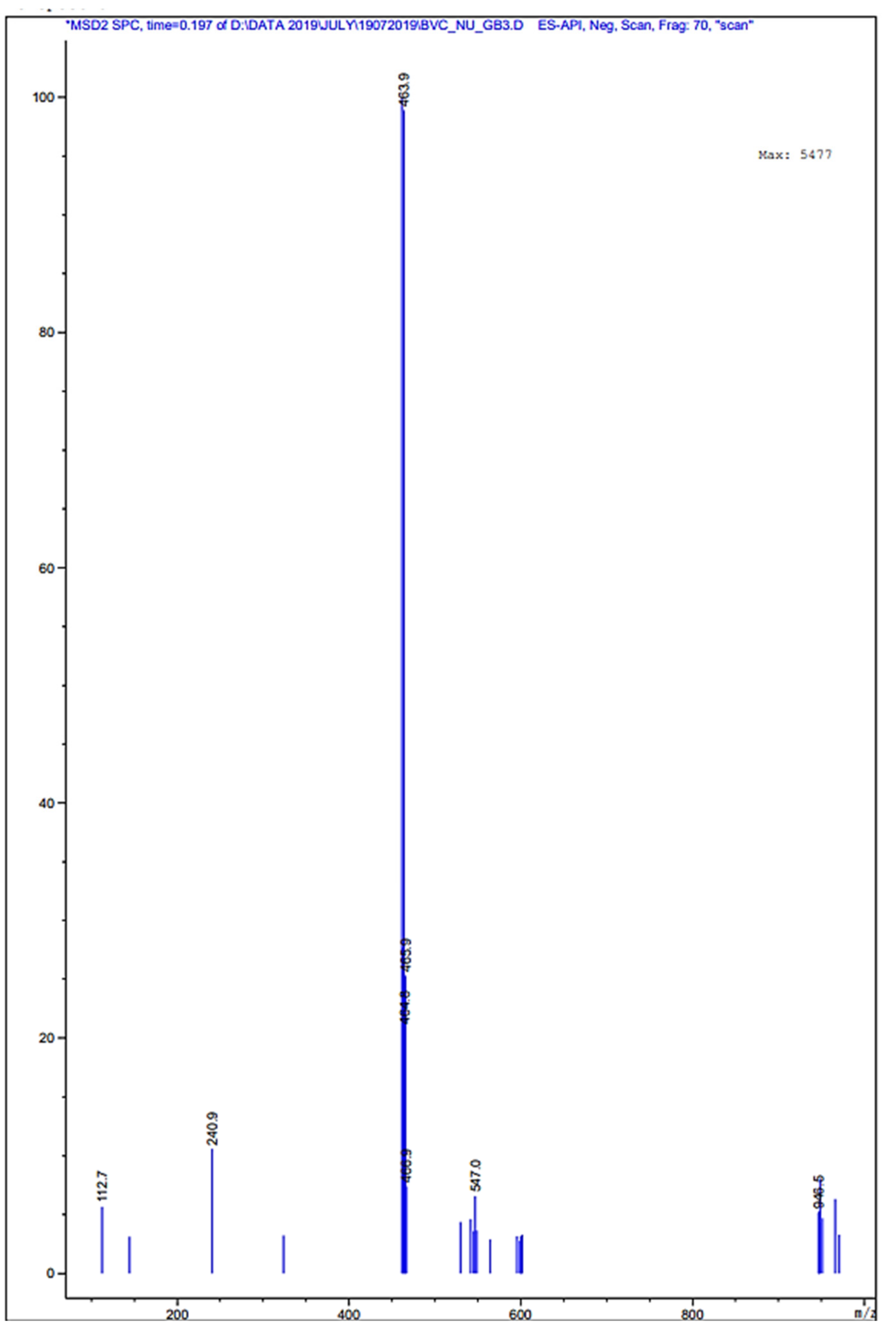
*MSD2 SPC, time=0.197 of D:\DATA 2019\JULY\19072019\BVC_NU_GB3.D ES-API, Neg, Scan, Frag: 70, "scan"
Max: 5477
463.9
240.9
112.7
547.0
100
80
60
40
20
0
200
400
600
800
m/z

6. UV

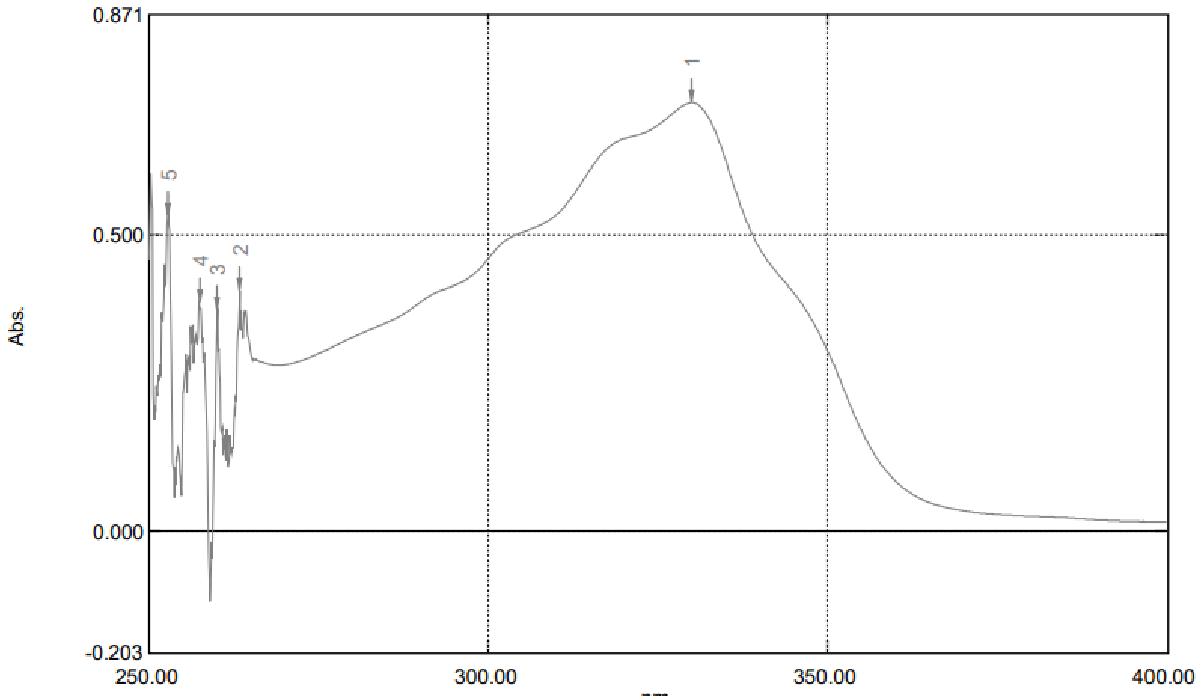

2-(5-(4-bromobenzylidene)-2,4-dioxothiazolidin-3-yl)-N-(4-chlorobenzo[d]thiazol-2-yl)acetamide (GB4)

1. FTIR

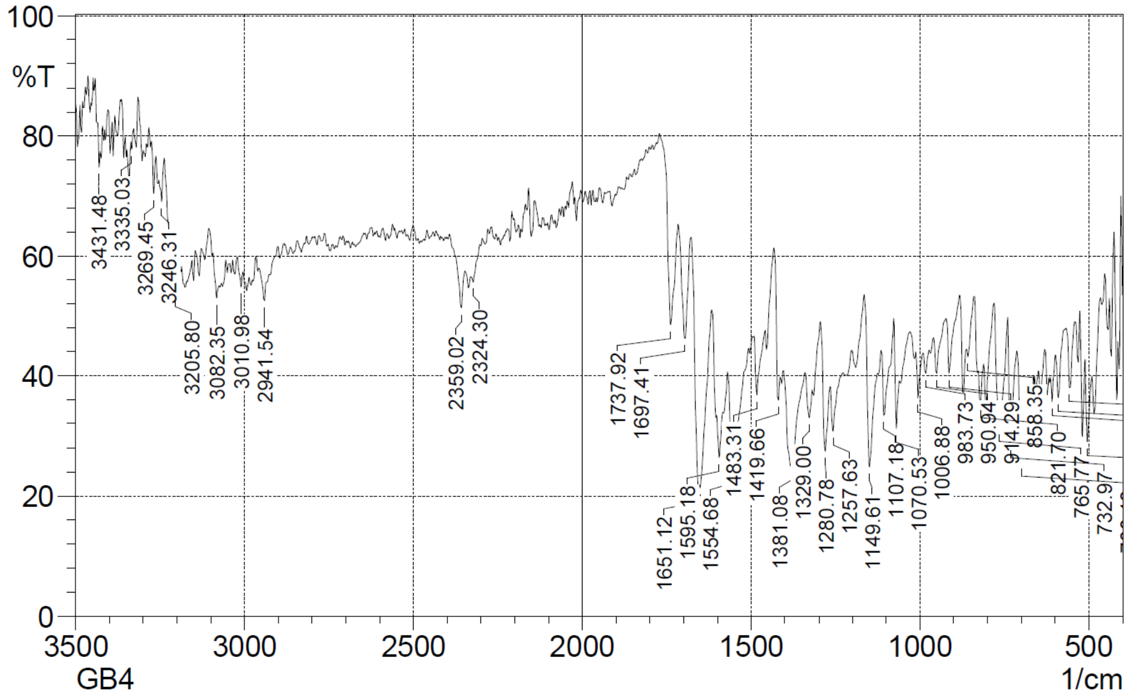

2. 1H-NMR

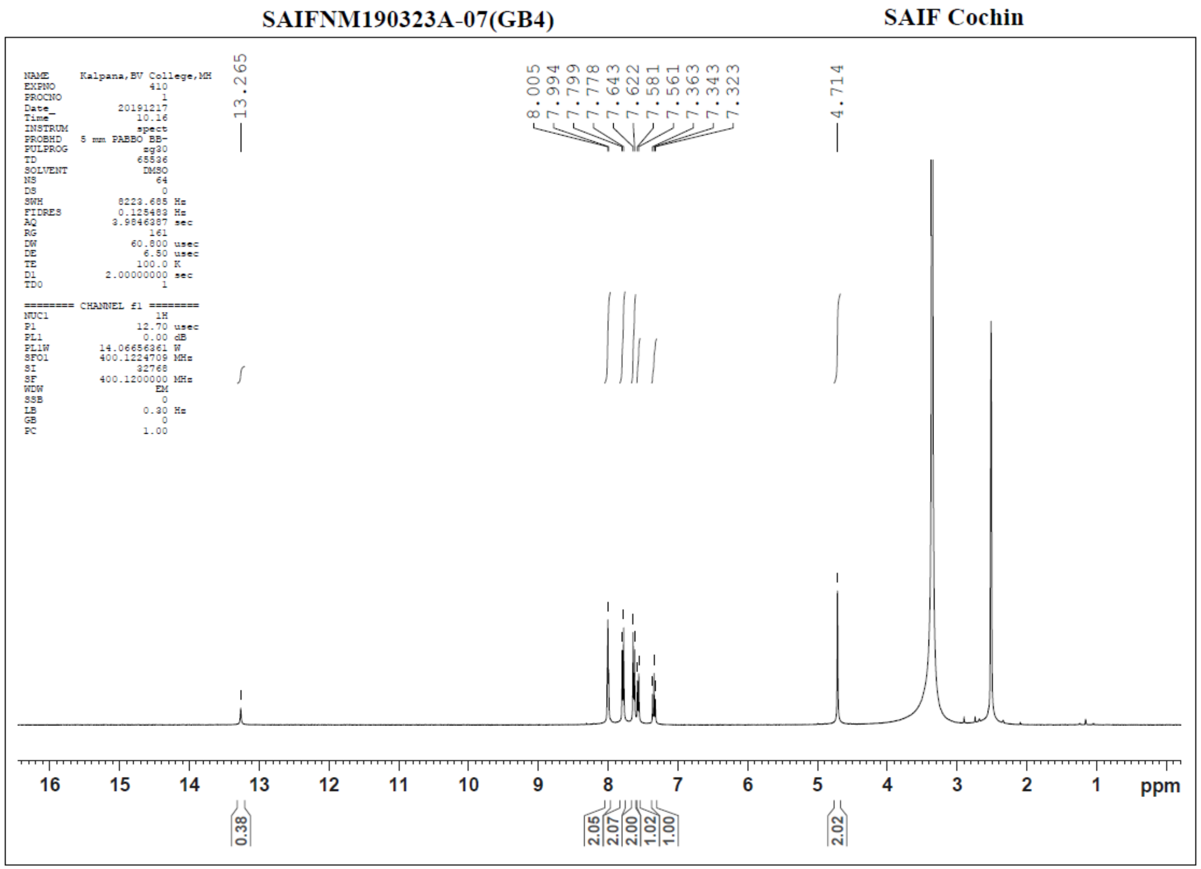
SAIFNM190323A-07(GB4)
SAIF Cochin
13.265
8.005
7.994
7.799
7.778
7.643
7.622
7.581
7.561
7.363
7.343
7.323
4.714
16 15 14 13 12 11 10 9 8 7 6 5 4 3 2 1 ppm
0.38
2.05
2.07
2.00
1.02
1.00
2.02

3. 13C-NMR

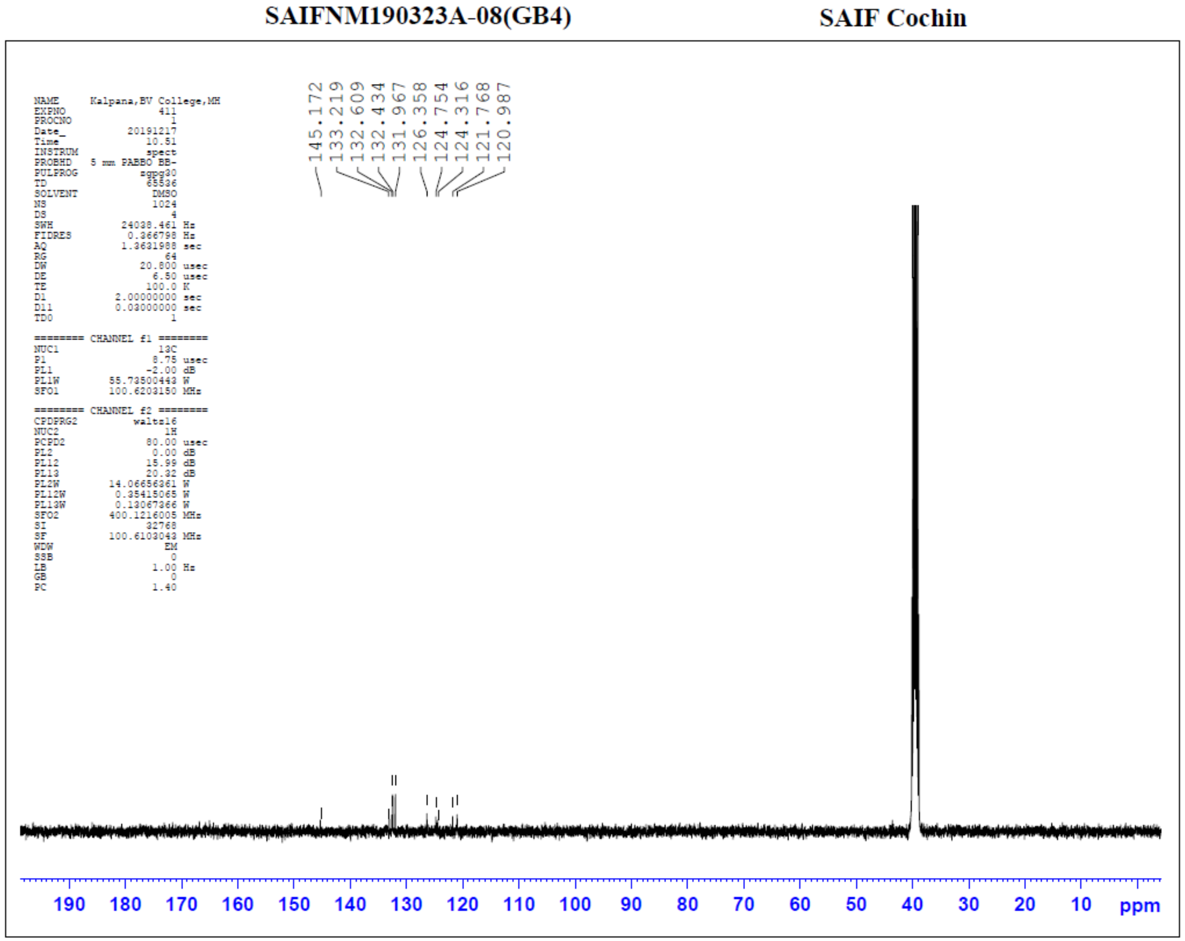
SAIFNM190323A-08(GB4)
SAIF Cochin
NAME Kalpana,BV College,MH
EXPNO 411
PROCNO 1
Date_ 20191217
Time 10.51
INSTRUM spect
PROBHD 5 mm PABBO BB-
PULPROG zgpg30
TD 65536
SOLVENT DMSO
NS 1024
DS 4
SWH 24038.461 Hz
FIDRES 0.366798 Hz
AQ 1.3631988 sec
RG 64
DW 20.800 usec
DE 6.50 usec
TE 100.0 K
D1 2.00000000 sec
D11 0.03000000 sec
TD0 1
======== CHANNEL f1 ========
NUC1 13C
P1 8.75 usec
PL1 -2.00 dB
PL1W 55.73500443 W
SFO1 100.6203150 MHz
======== CHANNEL f2 ========
CPDPRG2 waltz16
NUC2 1H
PCPD2 80.00 usec
PL2 0.00 dB
PL12 15.99 dB
PL13 20.32 dB
PL2W 14.06656361 W
PL12W 0.35415065 W
PL13W 0.13067366 W
SFO2 400.1216005 MHz
SI 32768
SF 100.6103043 MHz
WDW EM
SSB 0
LB 1.00 Hz
GB 0
PC 1.40
145.172
133.219
132.609
132.434
131.967
126.358
124.754
124.316
121.768
120.987
190 180 170 160 150 140 130 120 110 100 90 80 70 60 50 40 30 20 10 ppm

5. HPLC

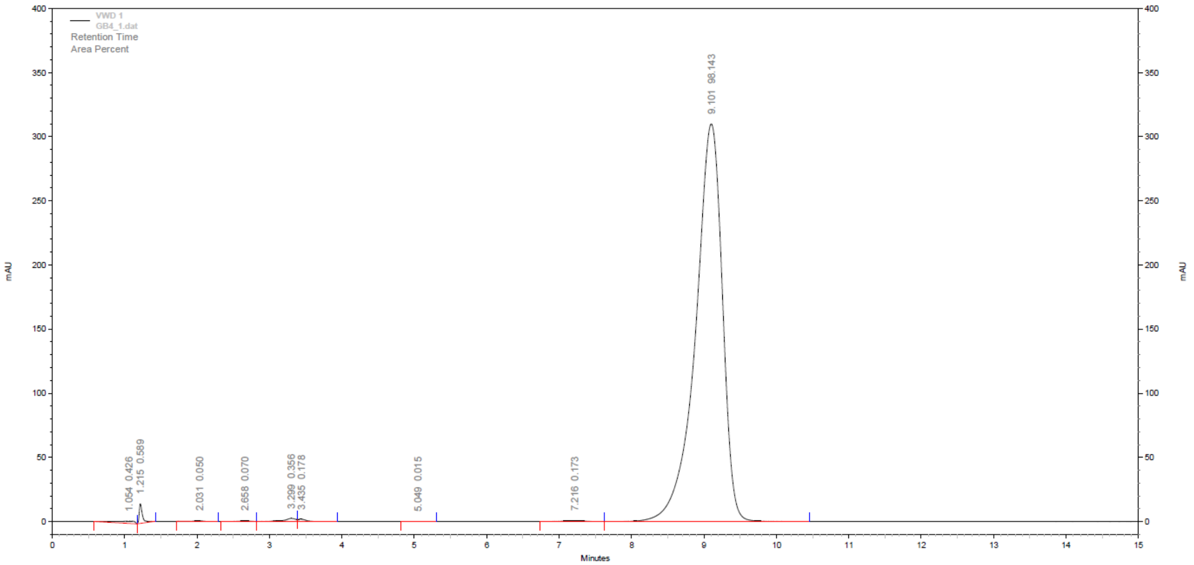
VWD 1
GB4_1.dat
Retention Time
Area Percent
9.101 98.143
1.054 0.426
1.215 0.589
2.031 0.050
2.658 0.070
3.299 0.356
3.435 0.178
5.049 0.015
7.216 0.173
mAU
Minutes

5. Mass

MS Spectrum

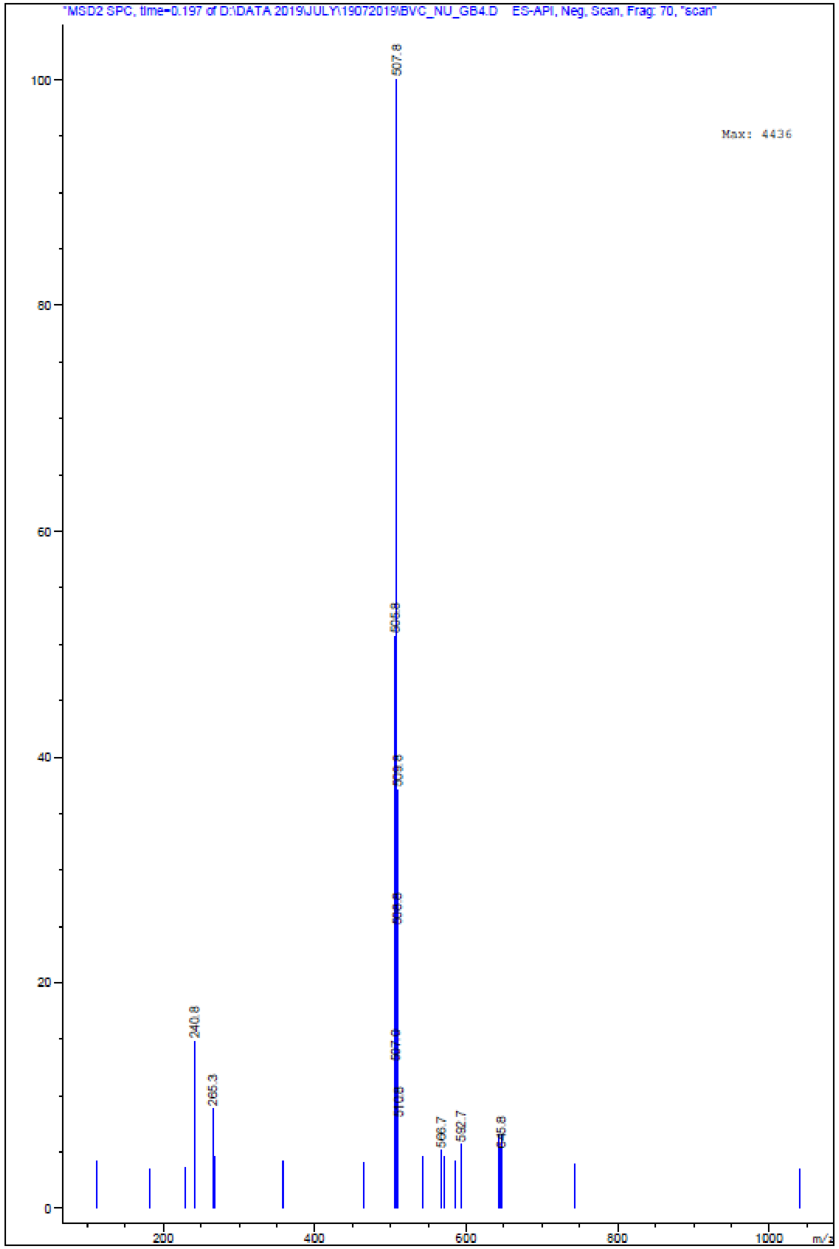

*MSD2 SPC, time=0.197 of D:\DATA 2019\JULY\19072019\BVC_NU_GB4.D ES-API, Neg, Scan, Frag: 70, "scan"
Max: 4436
507.8
240.8
265.3
592.7
645.8
m/z

N-(4-chlorobenzo[d]thiazol-2-yl)-2-(5-(2,4-difluorobenzylidene)-2,4-dioxothiazolidin-3-yl)acetamide (GB5)

1. FTIR -

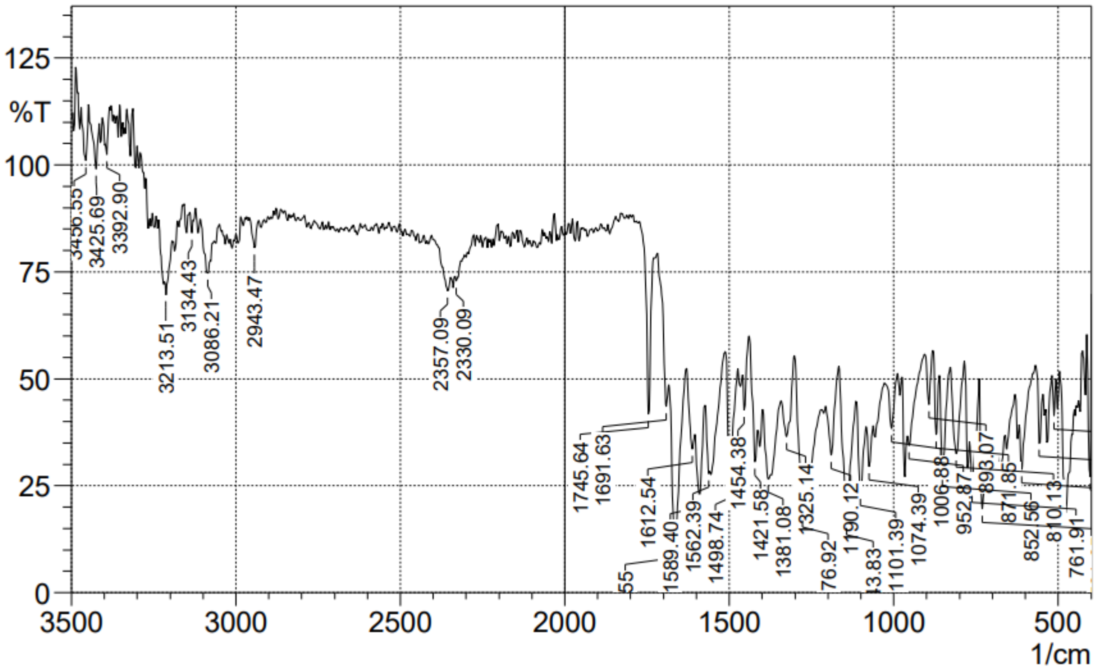

2. ^{I}H-NMR

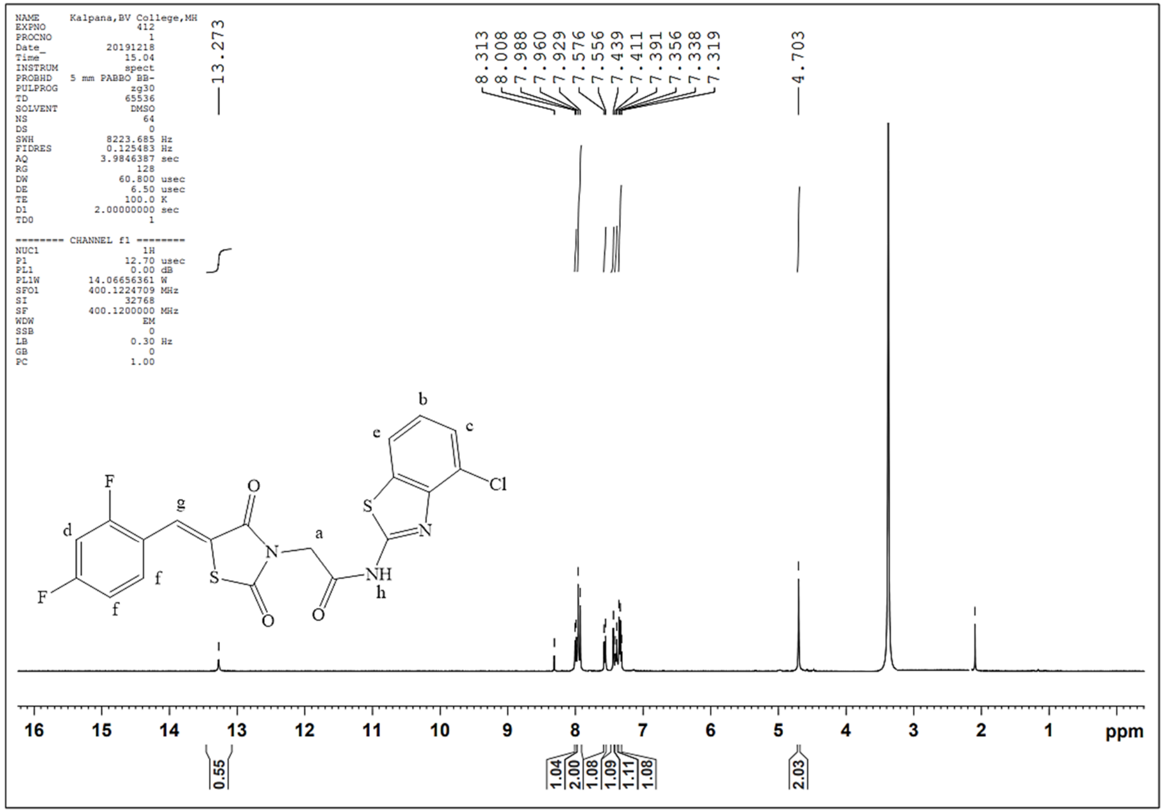
NAME Kalpana,BV College,MH
EXPNO 412
PROCNO 1
Date_ 20191218
Time 15.04
INSTRUM spect
PROBHD 5 mm PABBO BB-
PULPROG zg30
TD 65536
SOLVENT DMSO
NS 64
DS 0
SWH 8223.685 Hz
FIDRES 0.125483 Hz
AQ 3.9846387 sec
RG 128
DW 60.800 usec
DE 6.50 usec
TE 100.0 K
D1 2.00000000 sec
TD0 1
======== CHANNEL f1 ========
NUC1 1H
P1 12.70 usec
PL1 0.00 dB
PL1W 14.06656361 W
SFO1 400.1224709 MHz
SI 32768
SF 400.1200000 MHz
WDW EM
SSB 0
LB 0.30 Hz
GB 0
PC 1.00
13.273
8.313
8.008
7.988
7.960
7.929
7.576
7.556
7.439
7.411
7.391
7.356
7.338
7.319
4.703
b
c
e
Cl
F
O
S
N
g
d
a
NH
h
F
S
f
f
O
O
16 15 14 13 12 11 10 9 8 7 6 5 4 3 2 1 ppm
0.55
1.04
2.00
1.08
1.09
1.11
1.08
2.03

3. ^{13}C-NMR

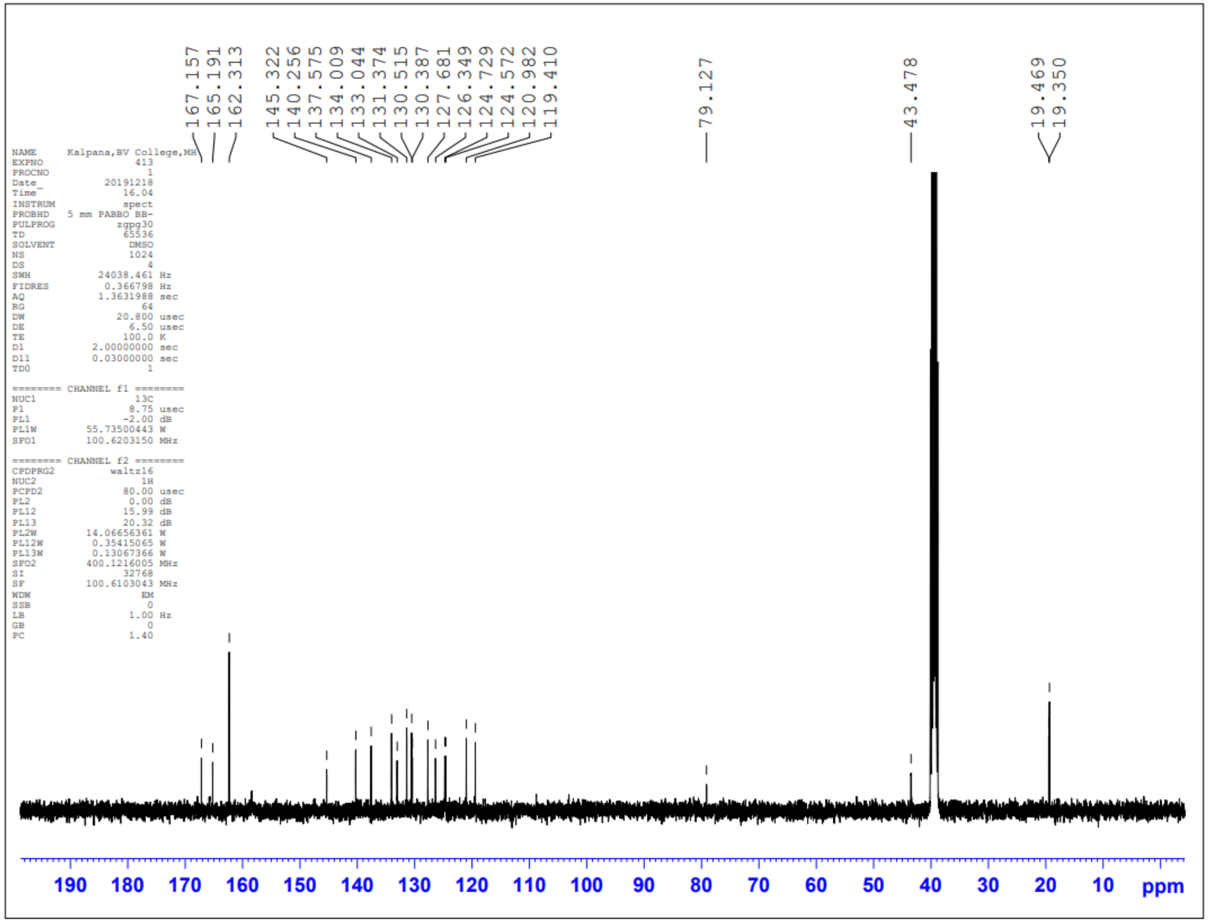
167.157
165.191
162.313
145.322
140.256
137.575
134.009
133.044
131.374
130.515
130.387
127.681
126.349
124.729
124.572
120.982
119.410
79.127
43.478
19.469
19.350
NAME Kalpana,BV College,MH
EXPNO 413
PROCNO 1
Date_ 20191218
Time 16.04
INSTRUM spect
PROBHD 5 mm PABBO BB-
PULPROG zgpg30
TD 65536
SOLVENT DMSO
NS 1024
DS 4
SWH 24038.461 Hz
FIDRES 0.366798 Hz
AQ 1.3631988 sec
RG 64
DW 20.800 usec
DE 6.50 usec
TE 100.0 K
D1 2.00000000 sec
D11 0.03000000 sec
TD0 1
======== CHANNEL f1 ========
NUC1 13C
P1 8.75 usec
PL1 -2.00 dB
PL1W 55.73500443 W
SFO1 100.6203150 MHz
======== CHANNEL f2 ========
CPDPRG2 waltz16
NUC2 1H
PCPD2 80.00 usec
PL2 0.00 dB
PL12 15.99 dB
PL13 20.32 dB
PL2W 14.06656361 W
PL12W 0.35415065 W
PL13W 0.13067366 W
SFO2 400.1216005 MHz
SI 32768
SF 100.6103043 MHz
WDW EM
SSB 0
LB 1.00 Hz
GB 0
PC 1.40
190 180 170 160 150 140 130 120 110 100 90 80 70 60 50 40 30 20 10 ppm

4. HPLC

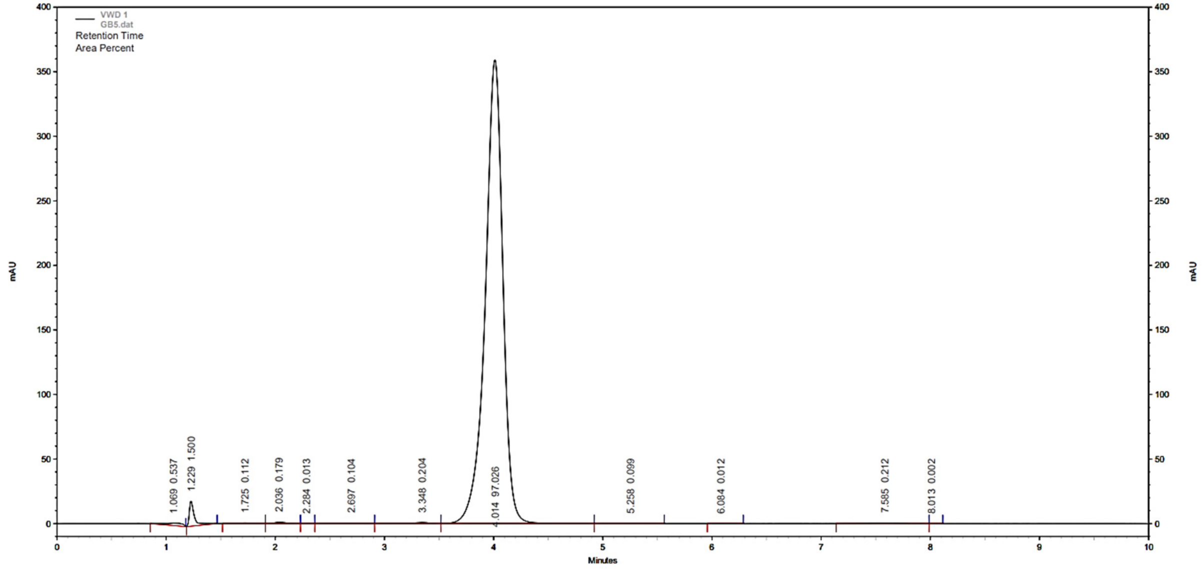

5. Mass Spectrometry

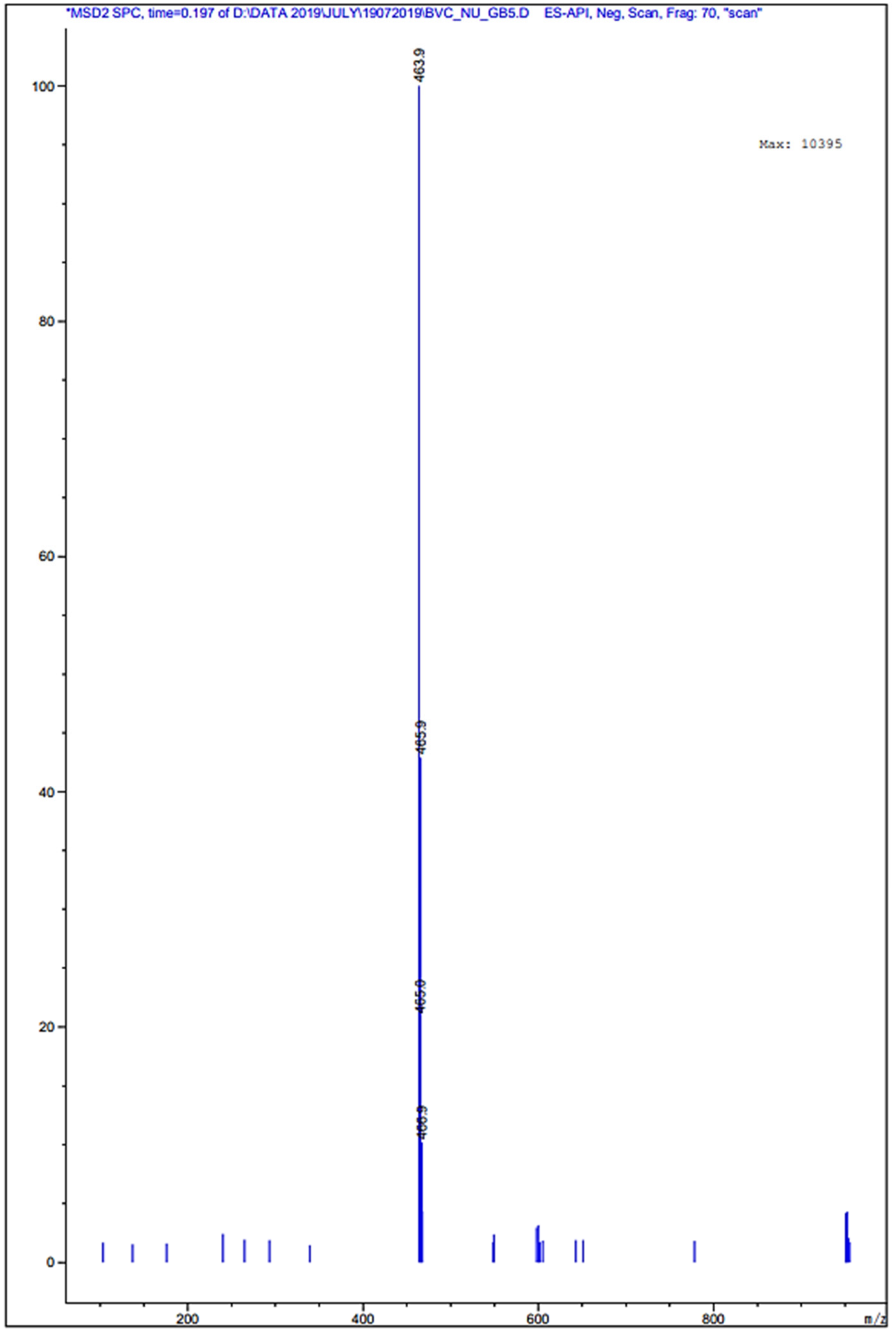
*MSD2 SPC, time=0.197 of D:\DATA 2019\JULY\19072019\BVC_NU_GB5.D ES-API, Neg, Scan, Frag: 70, "scan"
Max: 10395
463.9
465.9
465.0
466.9
100
80
60
40
20
0
200
400
600
800
m/z

6. UV

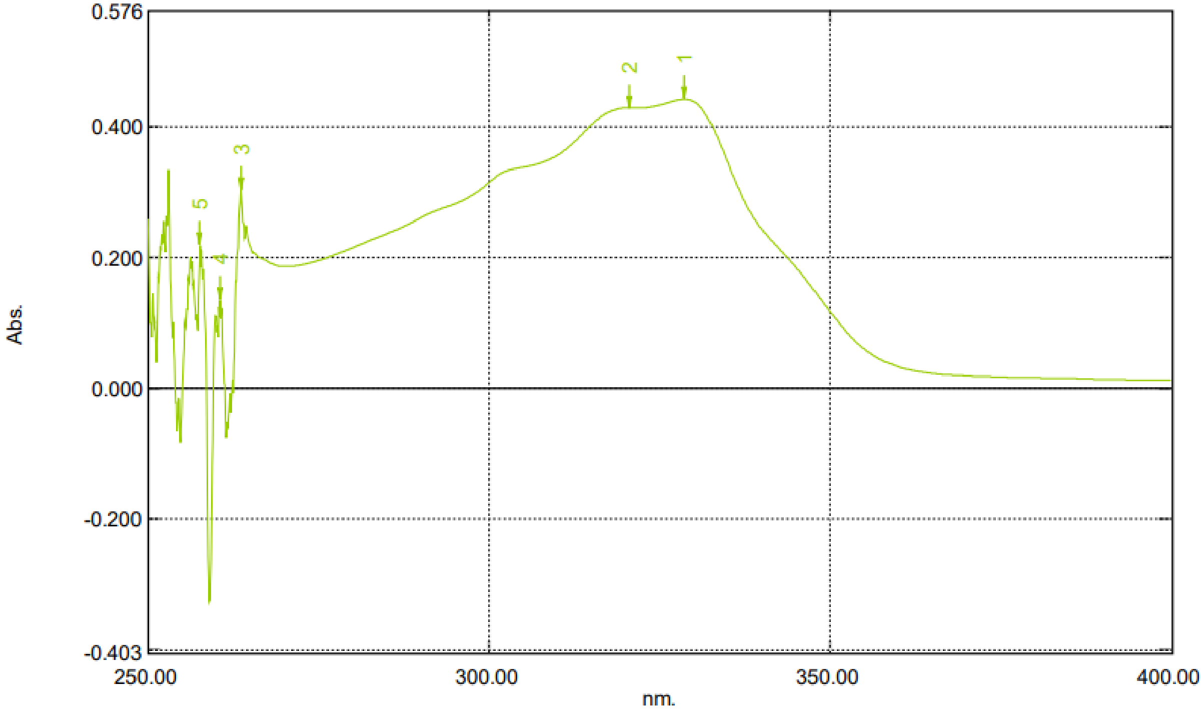

0.576
0.400
0.200
0.000
-0.200
-0.403
Abs.
250.00
300.00
350.00
400.00
nm.
1
2
3
4
5

N-(4-chlorobenzo[d]thiazol-2-yl)-2-(5-(3,4-dimethylbenzylidene)-2,4-dioxothiazolidin-3-yl)acetamide (GB6)

1. FTIR

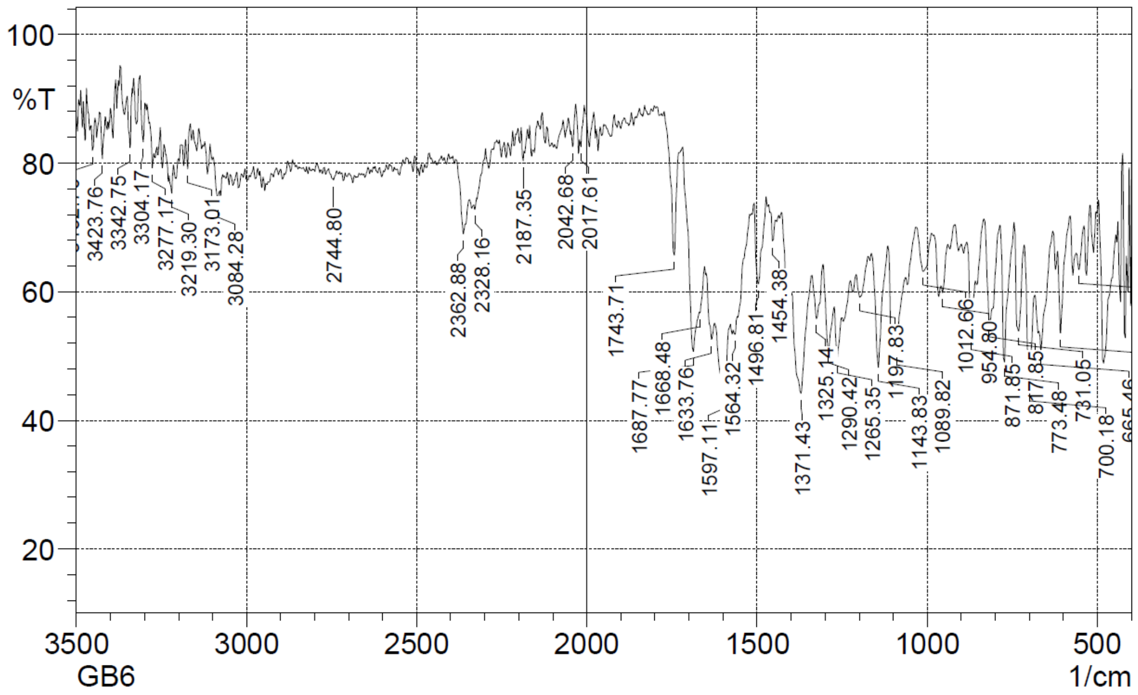

2. 1H-NMR

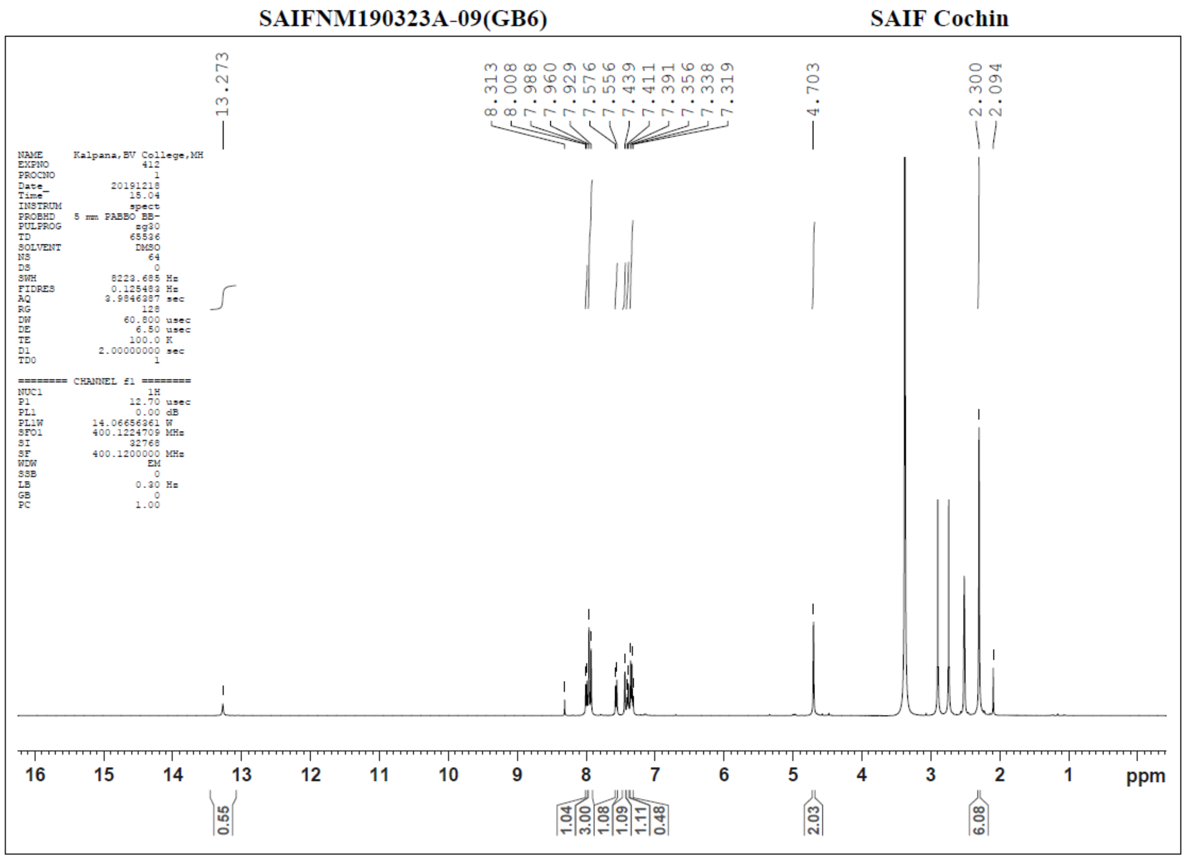
SAIFNM190323A-09(GB6)
SAIF Cochin
13.273
8.313
8.008
7.988
7.960
7.929
7.576
7.556
7.439
7.411
7.391
7.356
7.338
7.319
4.703
2.300
2.094
NAME Kalpana,BV College,MH
EXPNO 412
PROCNO 1
Date_ 20191218
Time 15.04
INSTRUM spect
PROBHD 5 mm PABBO BB-
PULPROG zg30
TD 65536
SOLVENT DMSO
NS 64
DS 0
SWH 8223.685 Hz
FIDRES 0.125483 Hz
AQ 3.9846387 sec
RG 128
DW 60.800 usec
DE 6.50 usec
TE 100.0 K
D1 2.00000000 sec
TD0 1
======== CHANNEL f1 ========
NUC1 1H
P1 12.70 usec
PL1 0.00 dB
PL1W 14.06656361 W
SFO1 400.1224709 MHz
SI 32768
SF 400.1200000 MHz
WDW EM
SSB 0
LB 0.30 Hz
GB 0
PC 1.00
16 15 14 13 12 11 10 9 8 7 6 5 4 3 2 1 ppm
0.55
1.04
3.00
1.08
1.09
1.11
0.48
2.03
6.08

3. 13C-NMR

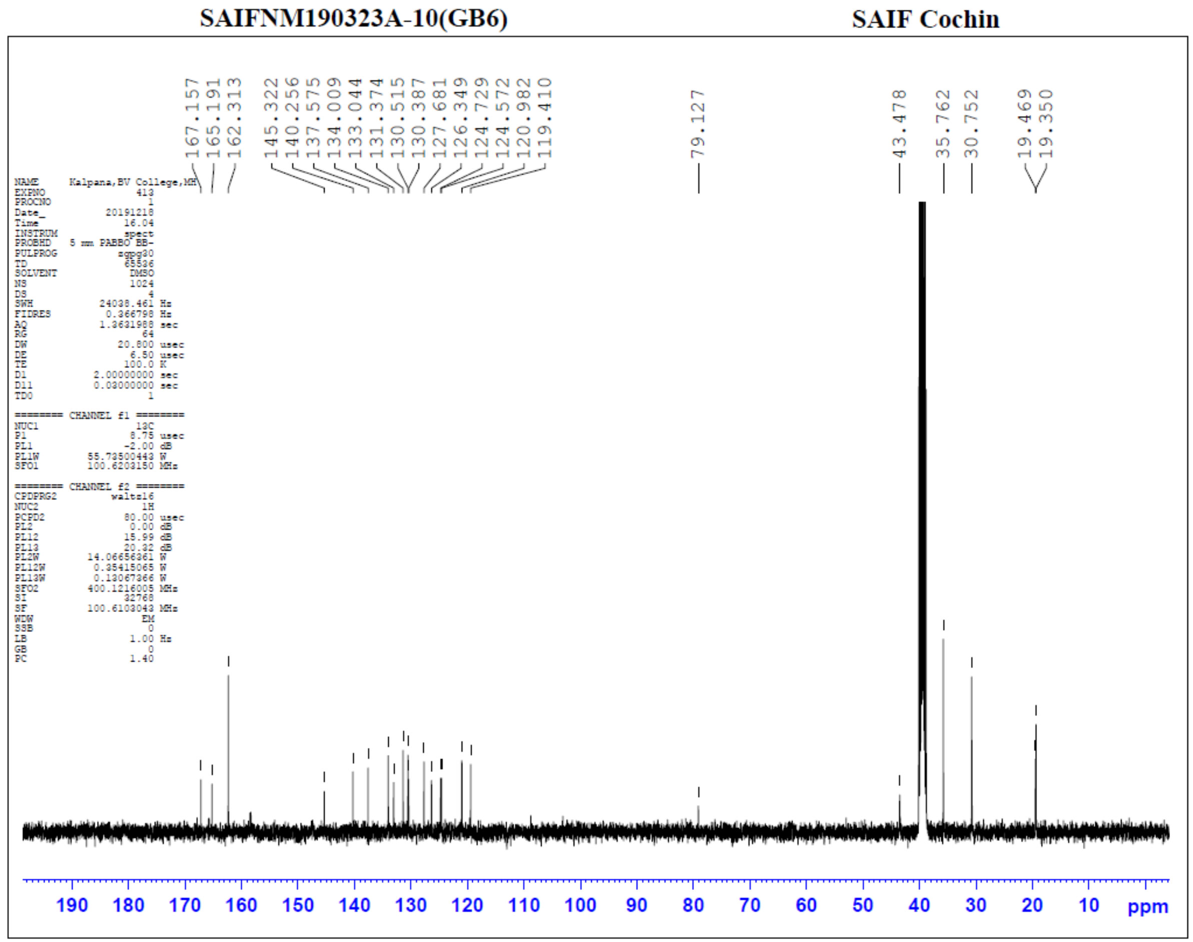
SAIFNM190323A-10(GB6)
SAIF Cochin
167.157
165.191
162.313
145.322
140.256
137.575
134.009
133.044
131.374
130.515
130.387
127.681
126.349
124.729
124.572
120.982
119.410
79.127
43.478
35.762
30.752
19.469
19.350
190 180 170 160 150 140 130 120 110 100 90 80 70 60 50 40 30 20 10 ppm

4. HPLC

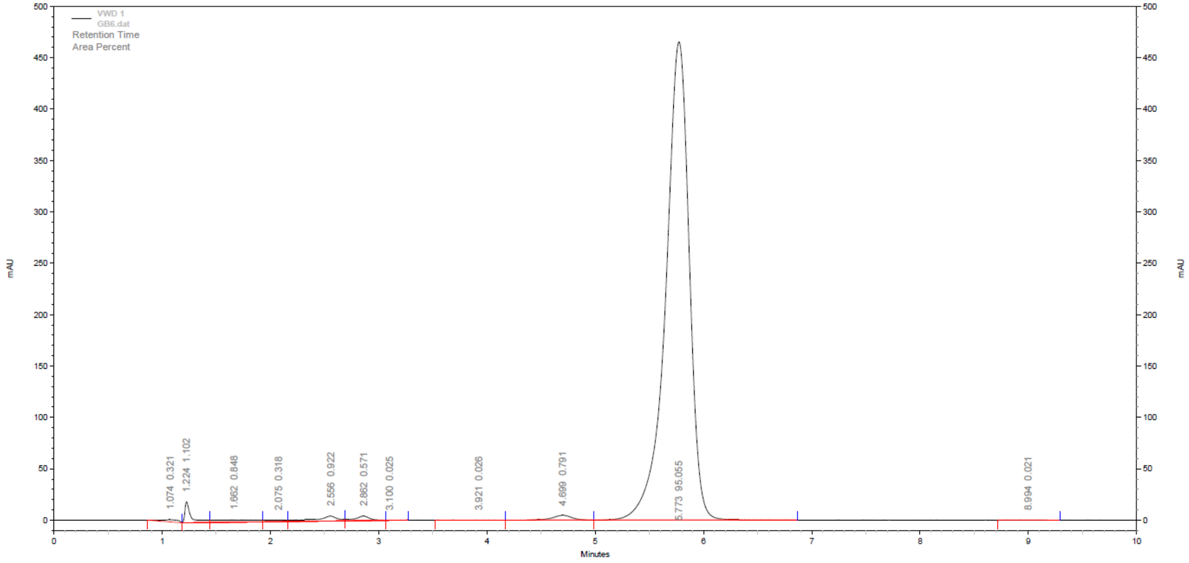

VWD 1
GB6.dat
Retention Time
Area Percent
mAU
Minutes
1.074 0.321
1.224 1.102
1.662 0.848
2.075 0.318
2.556 0.922
2.862 0.571
3.100 0.025
3.921 0.026
4.699 0.791
5.773 95.055
8.994 0.021

5. Mass

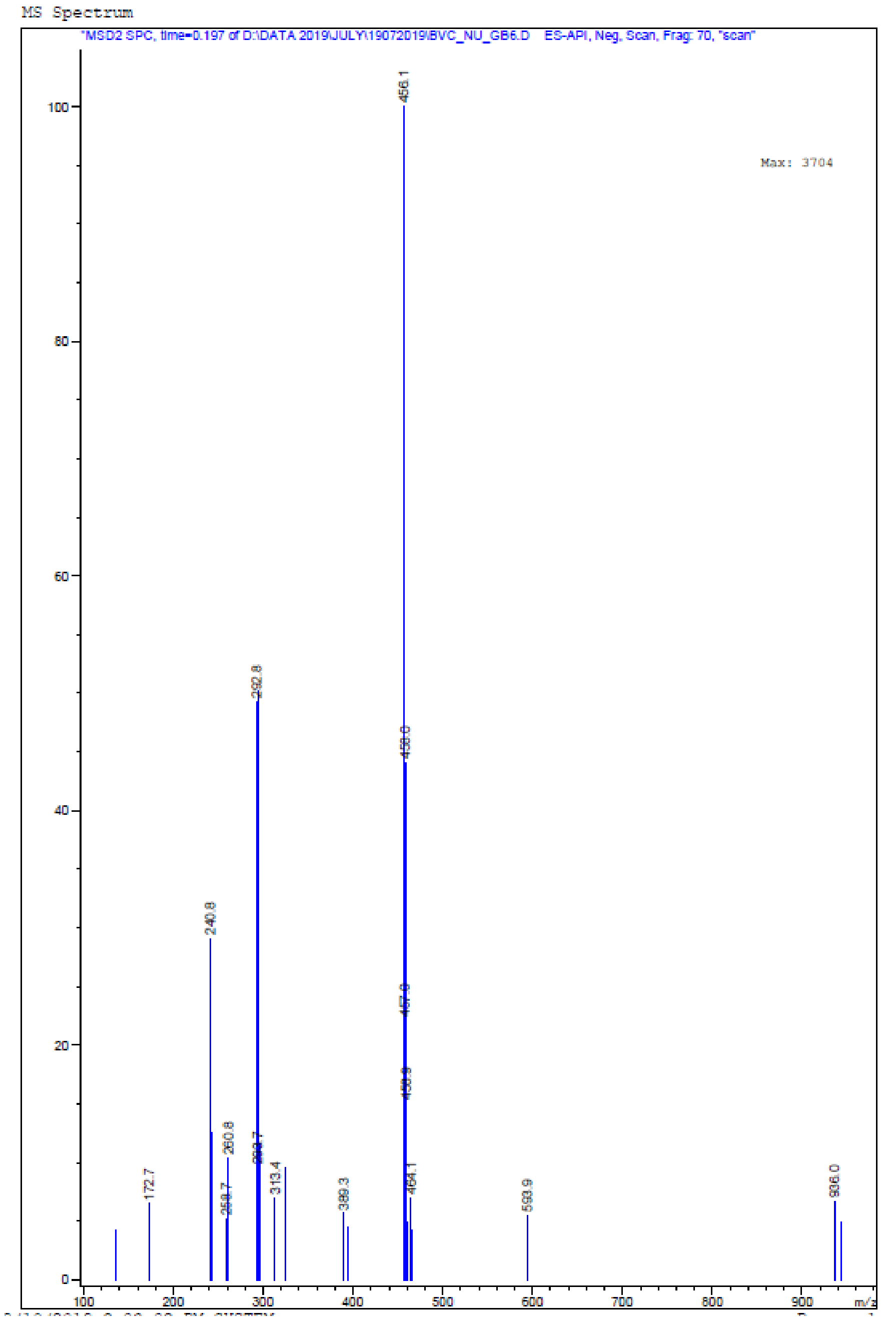
MS Spectrum
"MSD2 SPC, time=0.197 of D:\DATA 2019\JULY\19072019\BVC_NU_GB6.D   ES-API, Neg, Scan, Frag: 70, "scan"
Max: 3704
456.1
292.8
240.8
260.8
258.7
313.4
172.7
389.3
464.1
593.9
936.0
100
80
60
40
20
0
100
200
300
400
500
600
700
800
900
m/z

2-(5-(4-bromobenzylidene)-2,4-dioxothiazolidin-3-yl)-N-(4,6-difluorobenzo[d]thiazol-2-yl)acetamide (GB7)

1. FTIR –

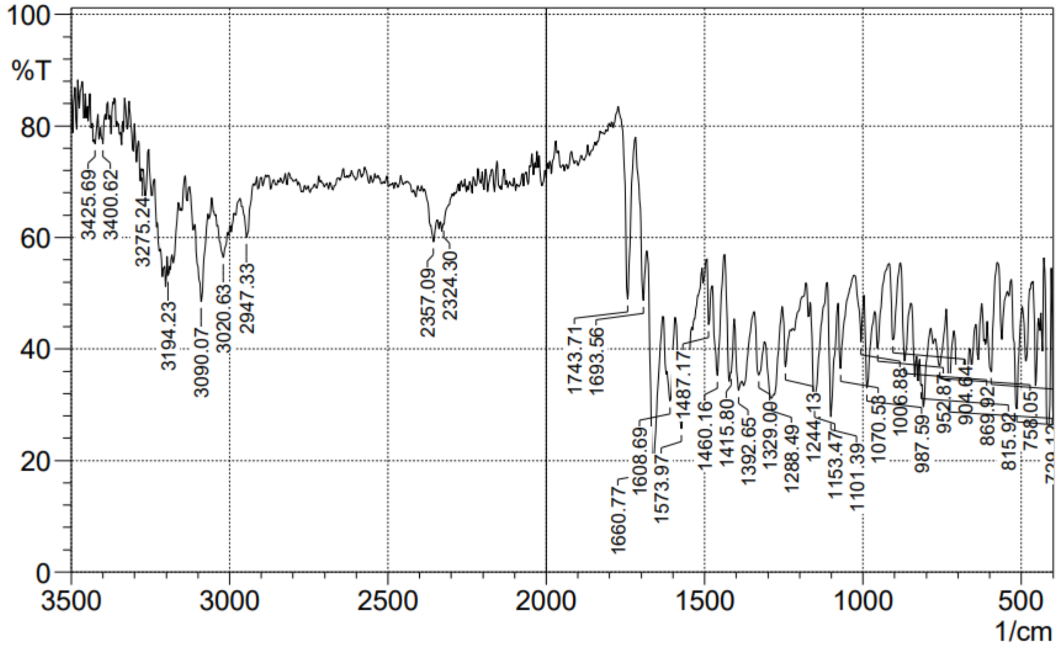

2. ^{1}H-NMR

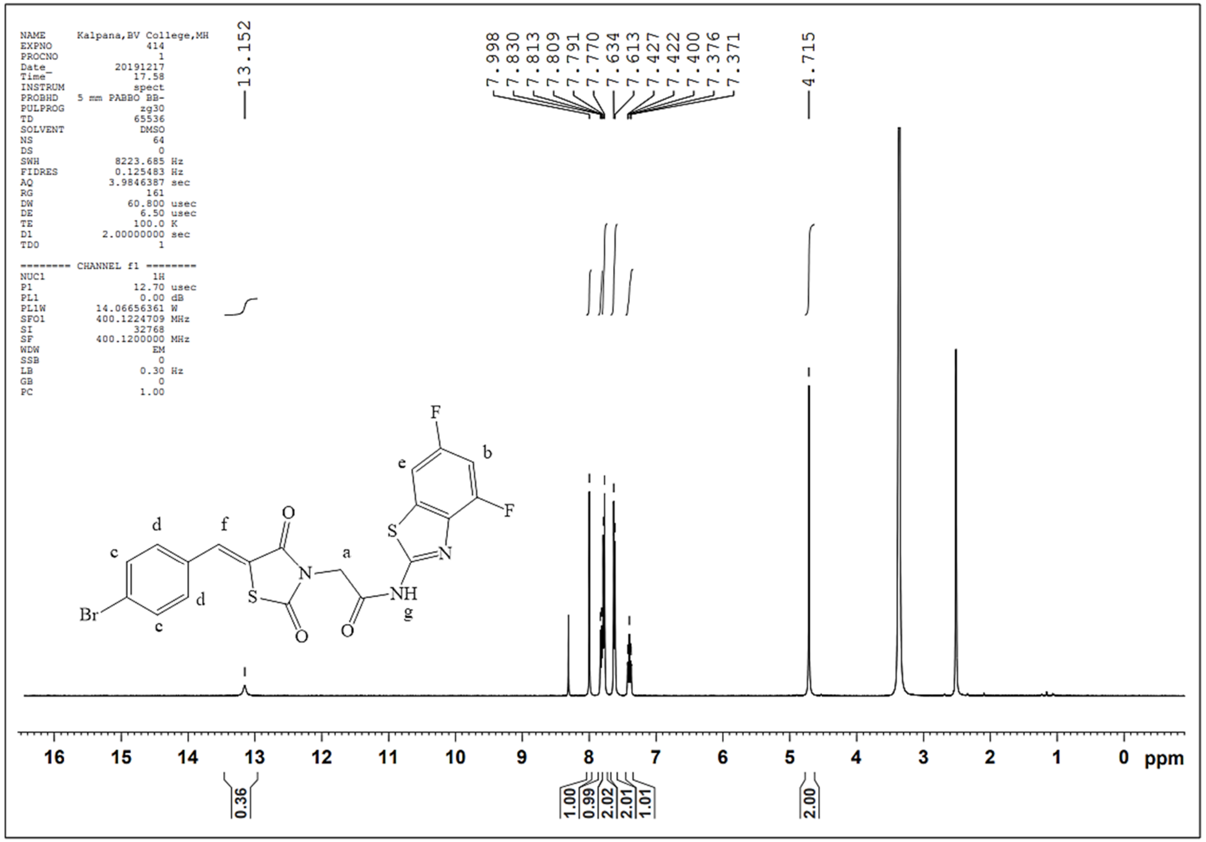
NAME Kalpana,BV College,MH
EXPNO 414
PROCNO 1
Date_ 20191217
Time 17.58
INSTRUM spect
PROBHD 5 mm PABBO BB-
PULPROG zg30
TD 65536
SOLVENT DMSO
NS 64
DS 0
SWH 8223.685 Hz
FIDRES 0.125483 Hz
AQ 3.9846387 sec
RG 161
DW 60.800 usec
DE 6.50 usec
TE 100.0 K
D1 2.00000000 sec
TD0 1
======== CHANNEL f1 ========
NUC1 1H
P1 12.70 usec
PL1 0.00 dB
PL1W 14.06656361 W
SFO1 400.1224709 MHz
SI 32768
SF 400.1200000 MHz
WDW EM
SSB 0
LB 0.30 Hz
GB 0
PC 1.00
13.152
7.998
7.830
7.813
7.809
7.791
7.770
7.634
7.613
7.427
7.422
7.400
7.376
7.371
4.715
16
15
14
13
12
11
10
9
8
7
6
5
4
3
2
1
0 ppm
0.36
1.00
0.99
2.02
2.01
1.01
2.00

3. ^{13}C-NMR

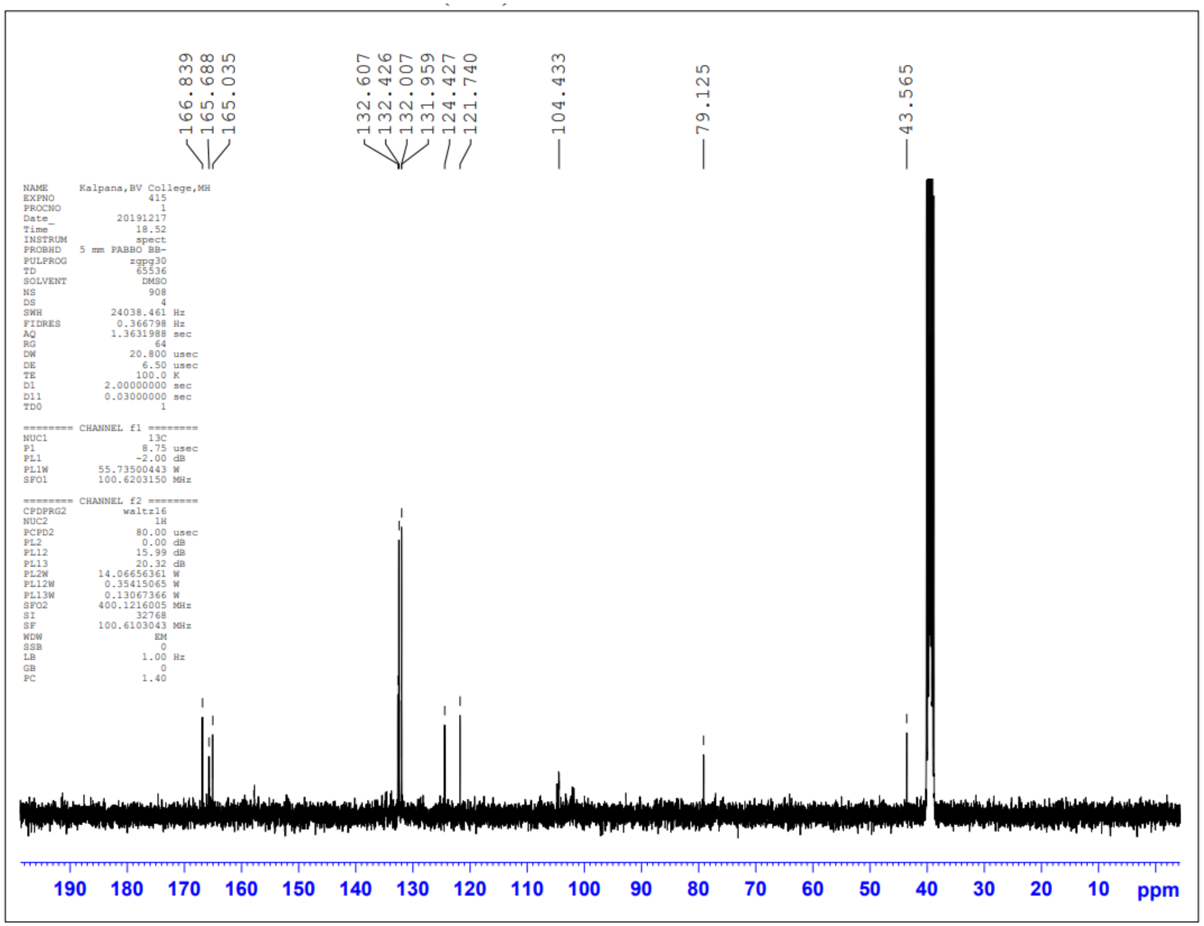

4. HPLC Analysis

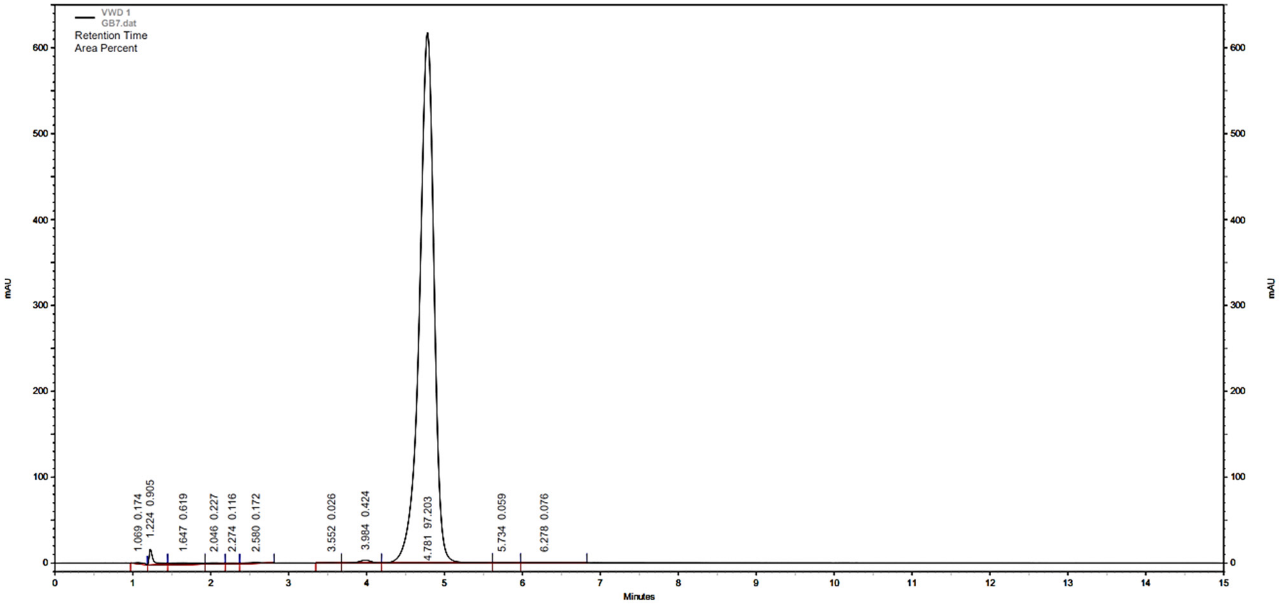

5. Mass Spectrometry

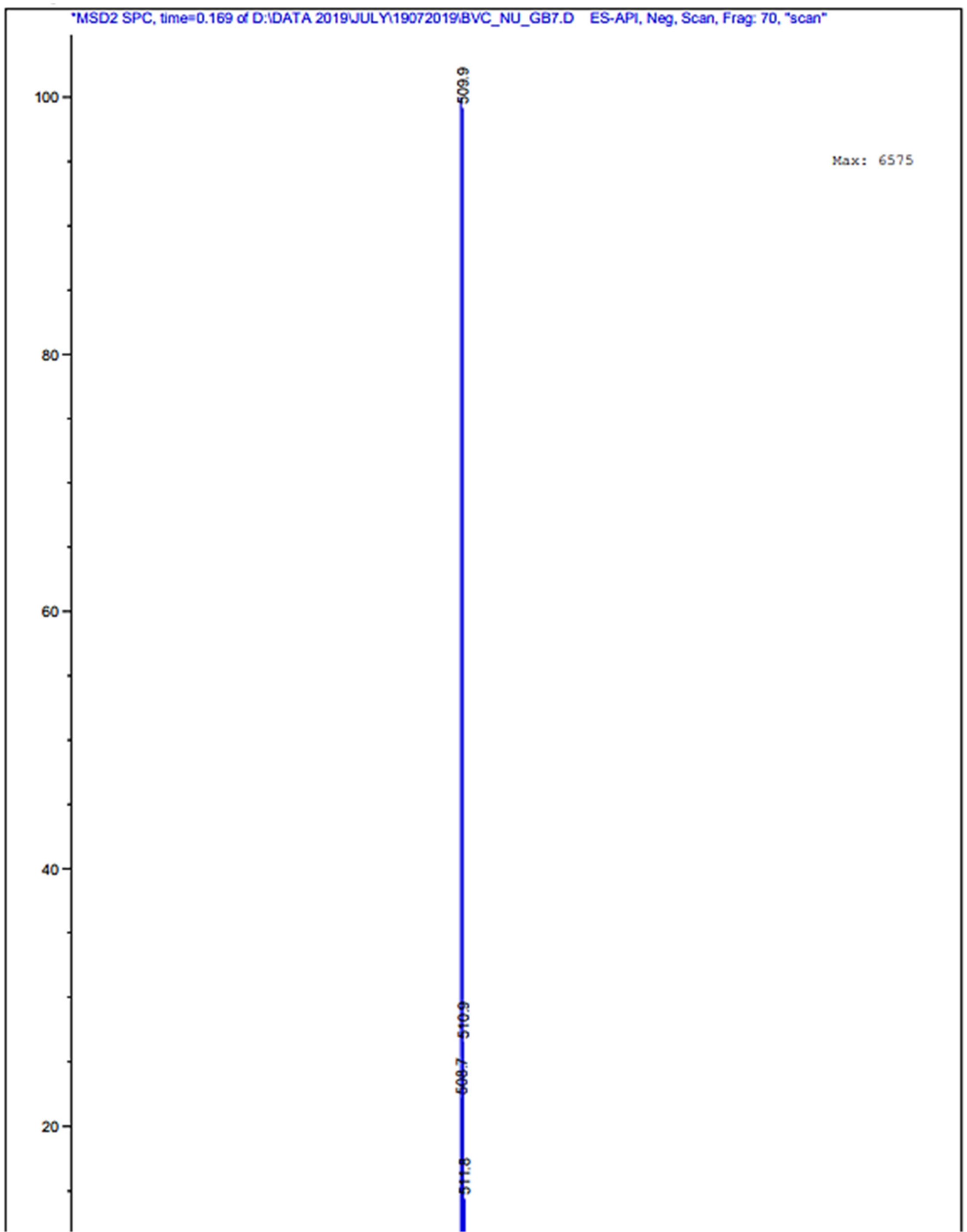

*MSD2 SPC, time=0.169 of D:\DATA 2019\JULY\19072019\BVC_NU_GB7.D ES-API, Neg, Scan, Frag: 70, "scan"
100
80
60
40
20
509.9
Max: 6575
510.9
508.7
511.8

6. UV

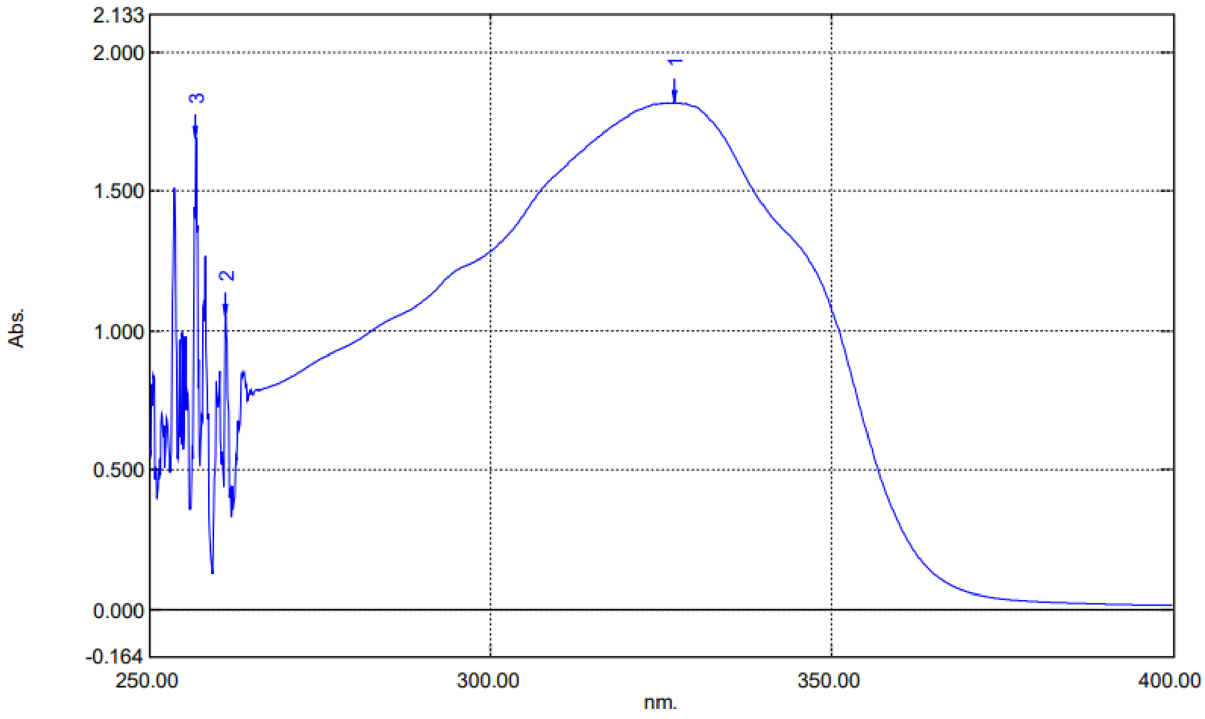

2.133
2.000
1.500
1.000
0.500
0.000
-0.164
Abs.
250.00
300.00
350.00
400.00
nm.
1
2
3

2-(5-benzylidene-2,4-dioxothiazolidin-3-yl)-N-(4-methoxybenzo[d]thiazol-2-yl)acetamide (GB8)

1. FTIR

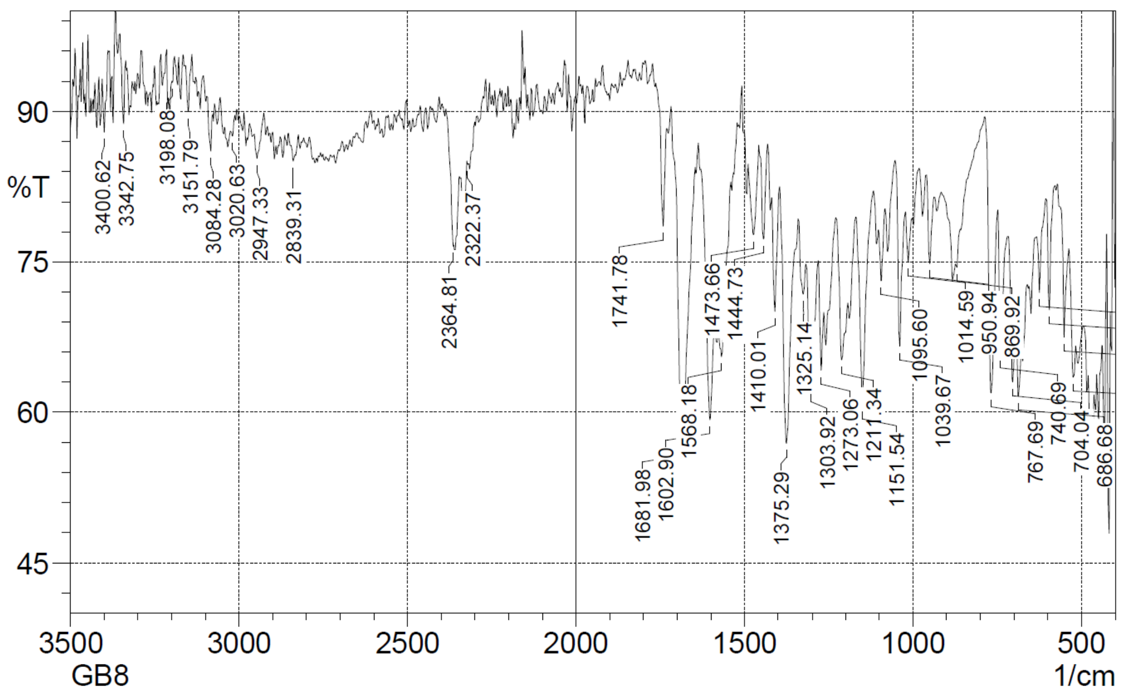

2. 1H-NMR

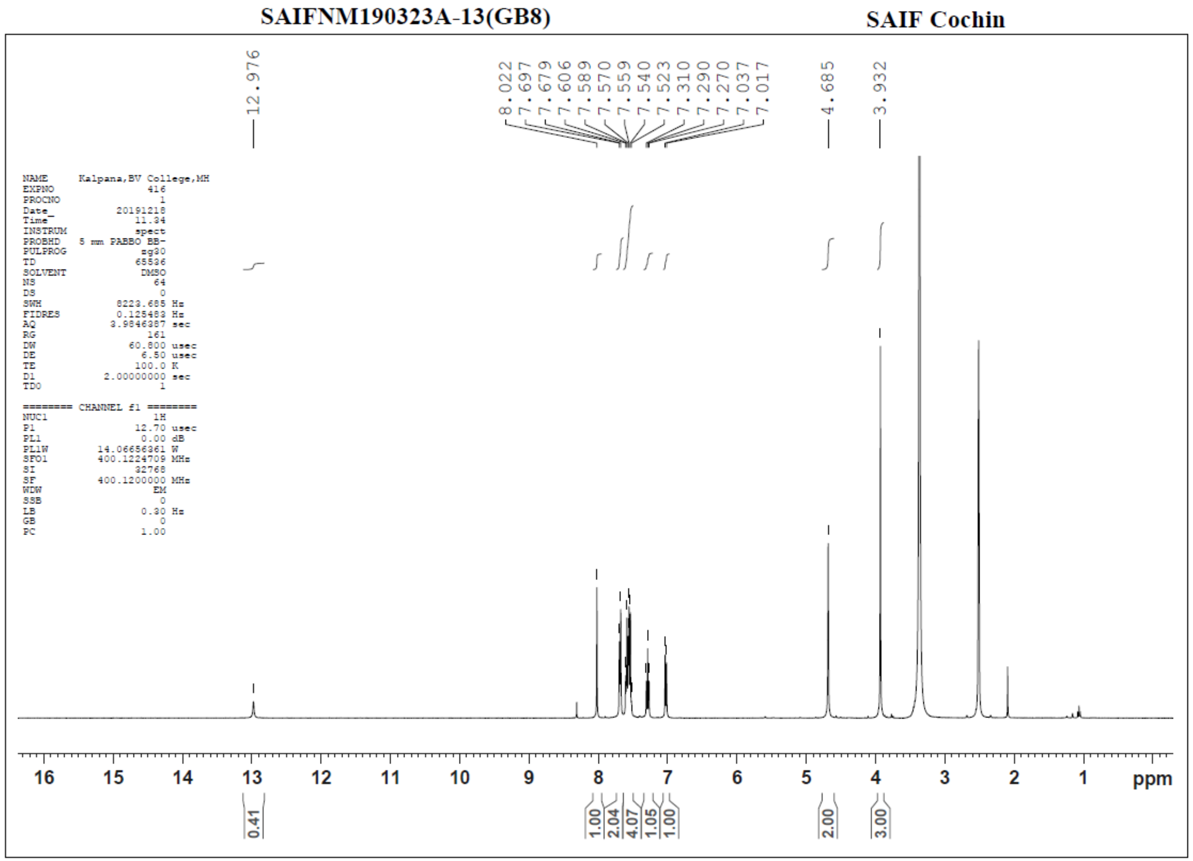
SAIFNM190323A-13(GB8)
SAIF Cochin
12.976
8.022
7.697
7.679
7.606
7.589
7.570
7.559
7.540
7.523
7.310
7.290
7.270
7.037
7.017
4.685
3.932
NAME Kalpana,BV College,MH
EXPNO 416
PROCNO 1
Date_ 20191218
Time 11.34
INSTRUM spect
PROBHD 5 mm PABBO BB-
PULPROG zg30
TD 65536
SOLVENT DMSO
NS 64
DS 0
SWH 8223.685 Hz
FIDRES 0.125483 Hz
AQ 3.9846387 sec
RG 161
DW 60.800 usec
DE 6.50 usec
TE 100.0 K
D1 2.00000000 sec
TD0 1
======== CHANNEL f1 ========
NUC1 1H
P1 12.70 usec
PL1 0.00 dB
PL1W 14.06656361 W
SFO1 400.1224709 MHz
SI 32768
SF 400.1200000 MHz
WDW EM
SSB 0
LB 0.30 Hz
GB 0
PC 1.00
16 15 14 13 12 11 10 9 8 7 6 5 4 3 2 1 ppm
0.41
1.00
2.04
4.07
1.05
1.00
2.00
3.00

3. 13C-NMR

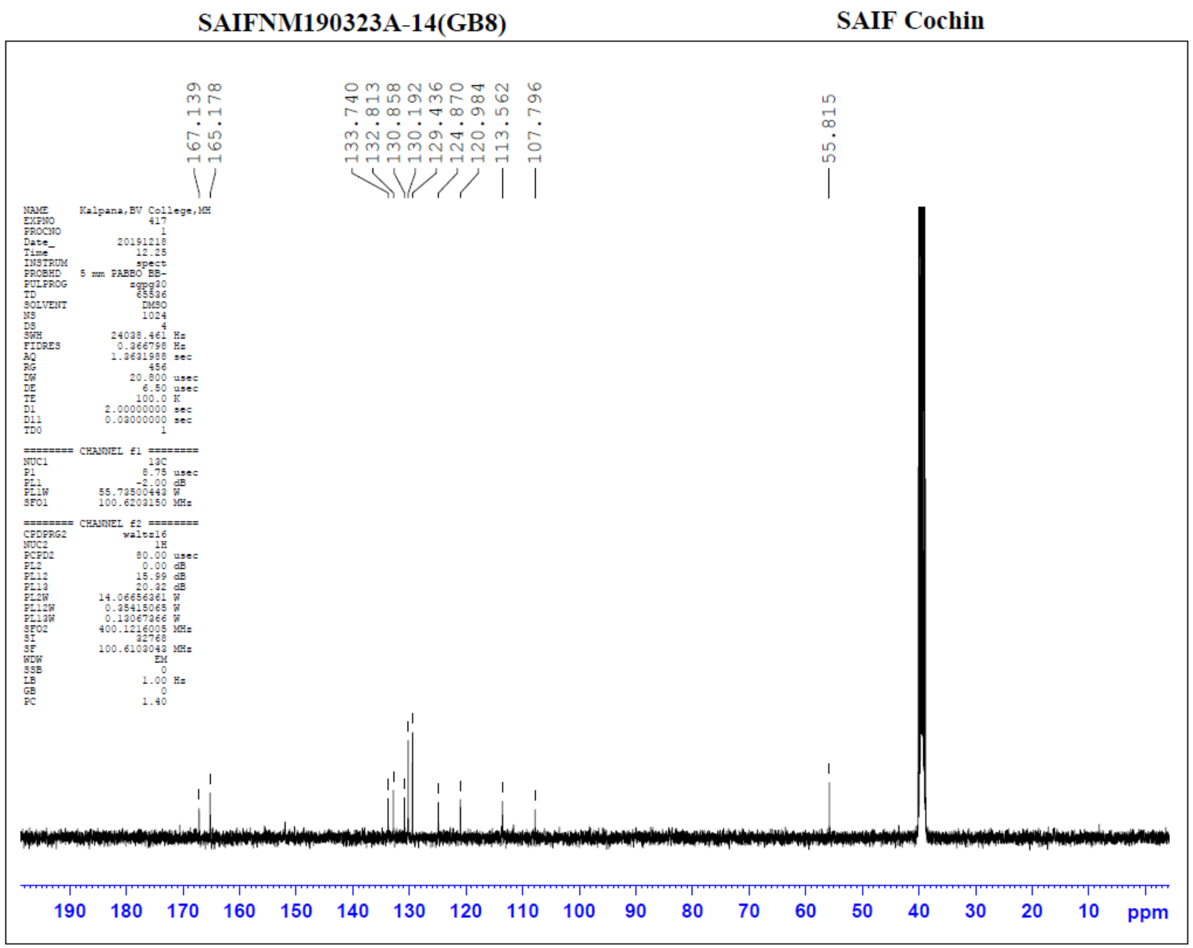
SAIFNM190323A-14(GB8)
SAIF Cochin
167.139
165.178
133.740
132.813
130.858
130.192
129.436
124.870
120.984
113.562
107.796
55.815
NAME Kalpana,BV College,MH
EXPNO 417
PROCNO 1
Date_ 20191218
Time 12.25
INSTRUM spect
PROBHD 5 mm PABBO BB-
PULPROG zgpg30
TD 65536
SOLVENT DMSO
NS 1024
DS 4
SWH 24038.461 Hz
FIDRES 0.366798 Hz
AQ 1.3631988 sec
RG 456
DW 20.800 usec
DE 6.50 usec
TE 100.0 K
D1 2.00000000 sec
D11 0.03000000 sec
TD0 1
======== CHANNEL f1 ========
NUC1 13C
P1 8.75 usec
PL1 -2.00 dB
PL1W 55.73500443 W
SFO1 100.6203150 MHz
======== CHANNEL f2 ========
CPDPRG2 waltz16
NUC2 1H
PCPD2 80.00 usec
PL2 0.00 dB
PL12 15.99 dB
PL13 20.32 dB
PL2W 14.06656361 W
PL12W 0.35415065 W
PL13W 0.13067366 W
SFO2 400.1216005 MHz
SI 32768
SF 100.6103043 MHz
WDW EM
SSB 0
LB 1.00 Hz
GB 0
PC 1.40
190
180
170
160
150
140
130
120
110
100
90
80
70
60
50
40
30
20
10
ppm

4. HPLC

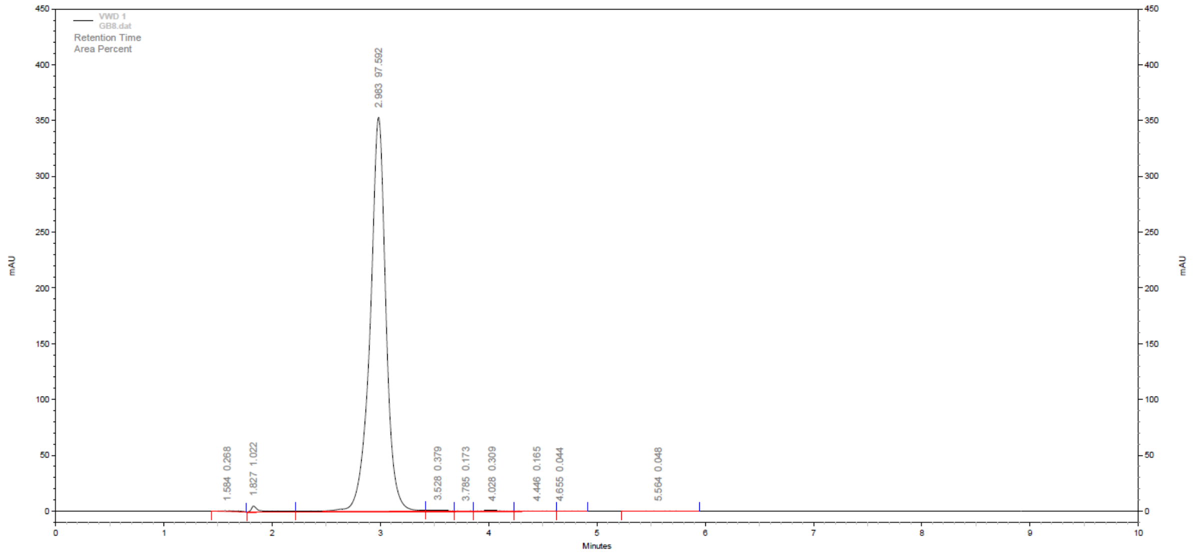

VWD 1
GB8.dat
Retention Time
Area Percent
2.983 97.592
1.584 0.268
1.827 1.022
3.528 0.379
3.785 0.173
4.028 0.309
4.446 0.165
4.655 0.044
5.564 0.048
mAU
Minutes

4. Mass

MS Spectrum

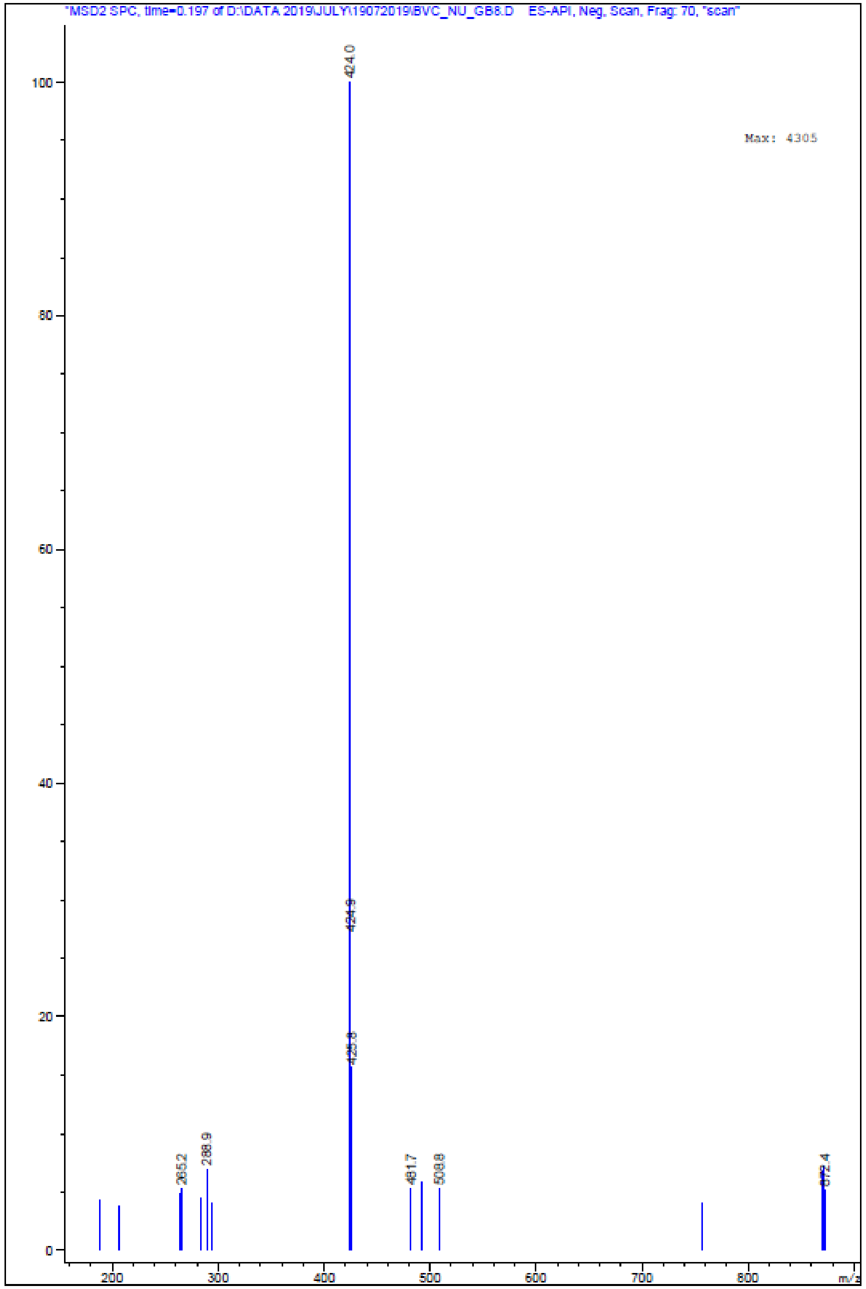

"MSD2 SPC, time=0.197 of D:\DATA 2019\JULY\19072019\BVC_NU_GB8.D ES-API, Neg, Scan, Frag: 70, "scan"
Max: 4305
424.0
424.9
425.8
265.2
288.9
481.7
508.8
872.4
m/z

2-(5-benzylidene-2,4-dioxothiazolidin-3-yl)-N-(4-methylbenzo[d]thiazol-2-yl)acetamide (GB9)

1. FTIR –

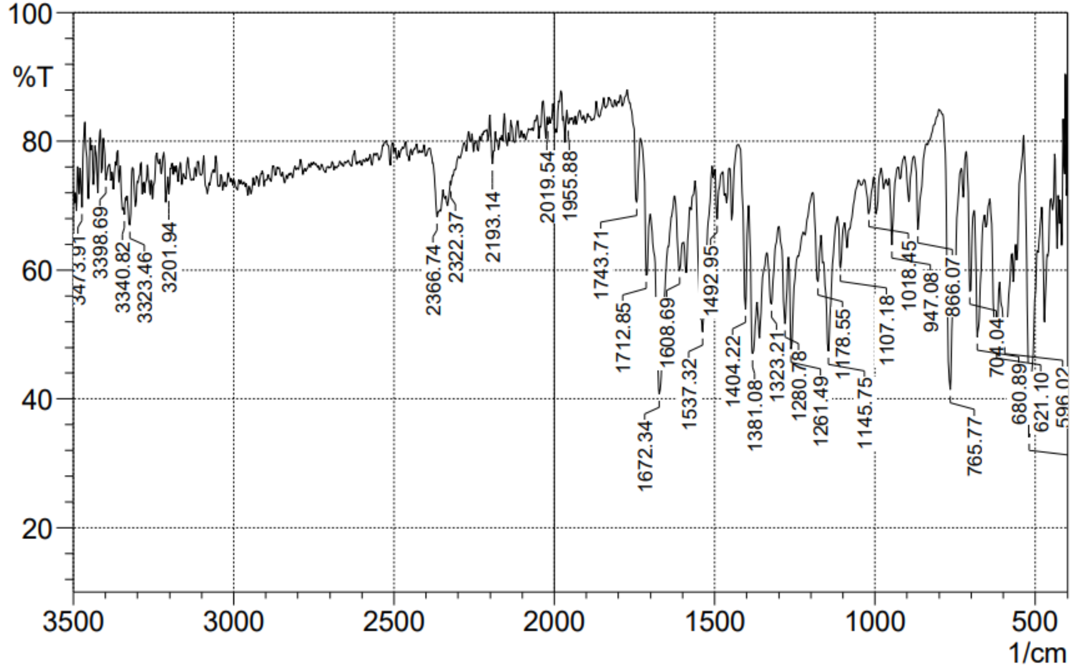

2. IH-NMR

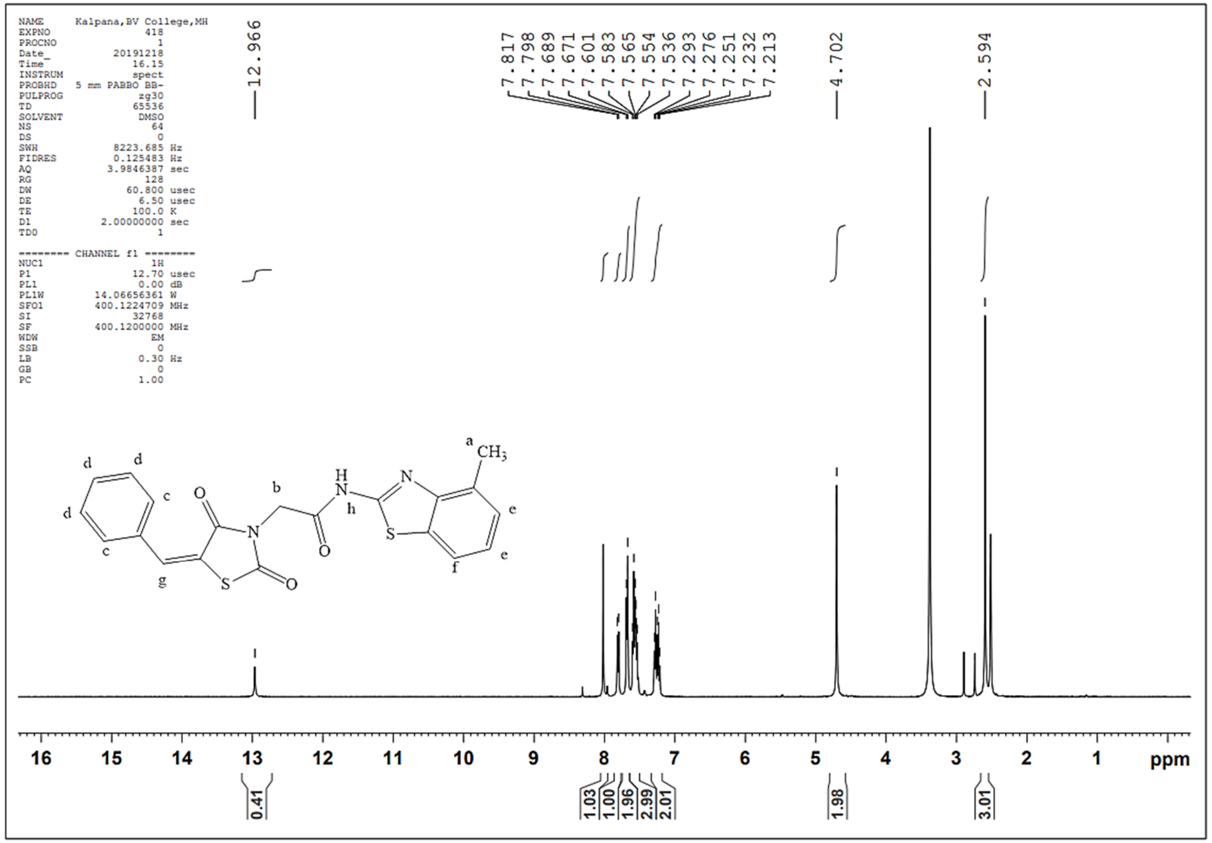
NAME Kalpana,BV College,MH
EXPNO 418
PROCNO 1
Date_ 20191218
Time 16.15
INSTRUM spect
PROBHD 5 mm PABBO BB-
PULPROG zg30
TD 65536
SOLVENT DMSO
NS 64
DS 0
SWH 8223.685 Hz
FIDRES 0.125483 Hz
AQ 3.9846387 sec
RG 128
DW 60.800 usec
DE 6.50 usec
TE 100.0 K
D1 2.00000000 sec
TD0 1
======== CHANNEL f1 ========
NUC1 1H
P1 12.70 usec
PL1 0.00 dB
PL1W 14.06656361 W
SFO1 400.1224709 MHz
SI 32768
SF 400.1200000 MHz
WDW EM
SSB 0
LB 0.30 Hz
GB 0
PC 1.00
12.966
7.817
7.798
7.689
7.671
7.601
7.583
7.565
7.554
7.536
7.293
7.276
7.251
7.232
7.213
4.702
2.594
16 15 14 13 12 11 10 9 8 7 6 5 4 3 2 1 ppm
0.41
1.03
1.00
1.96
2.99
2.01
1.98
3.01
a
CH3
b
c
d
e
f
g
h
H
N
O
S

3. ^{13}C-NMR

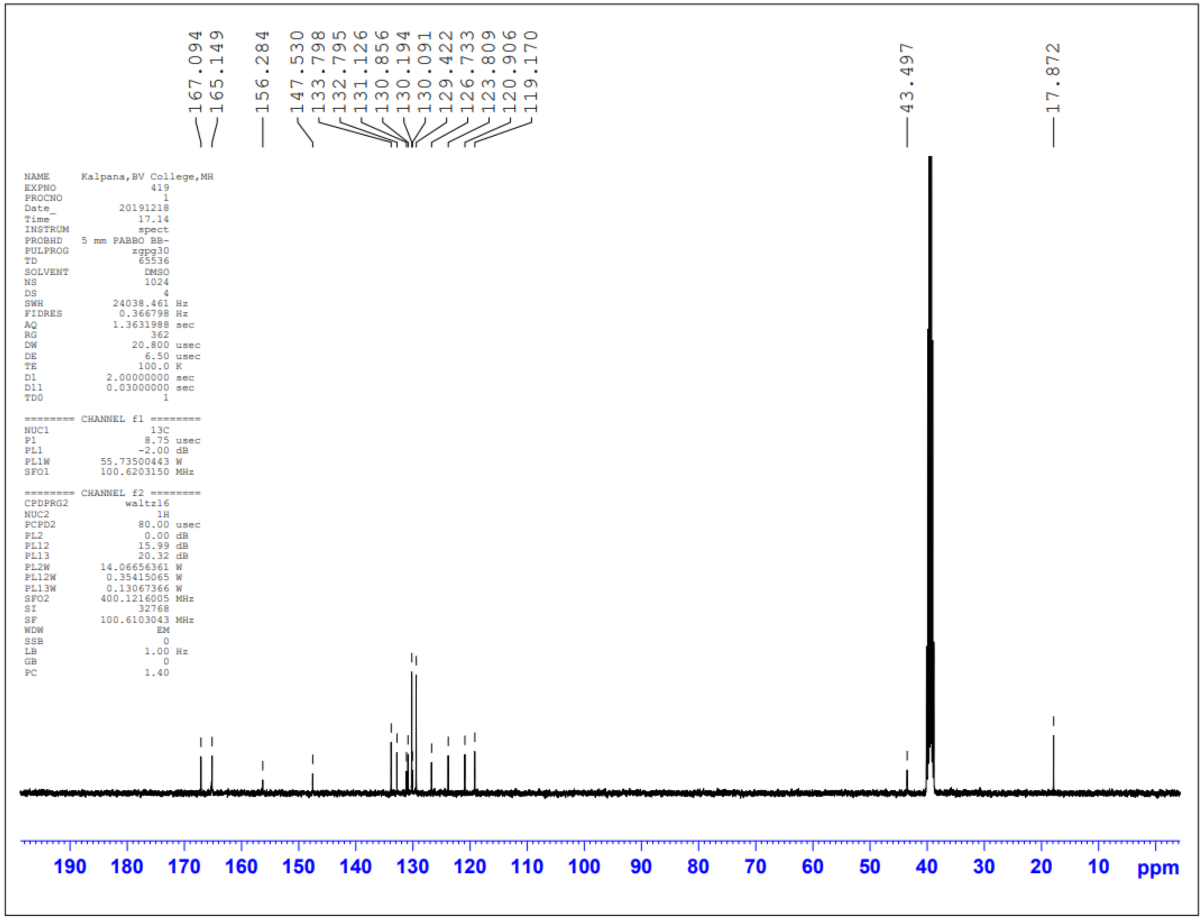

4. HPLC Analysis

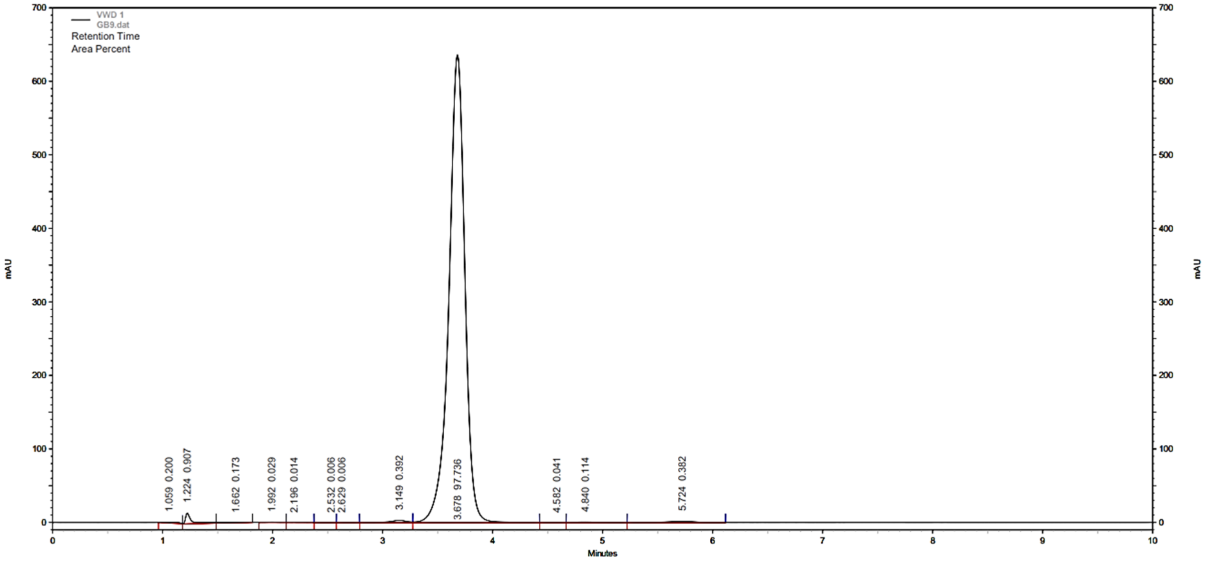

5. Mass Spectrometry

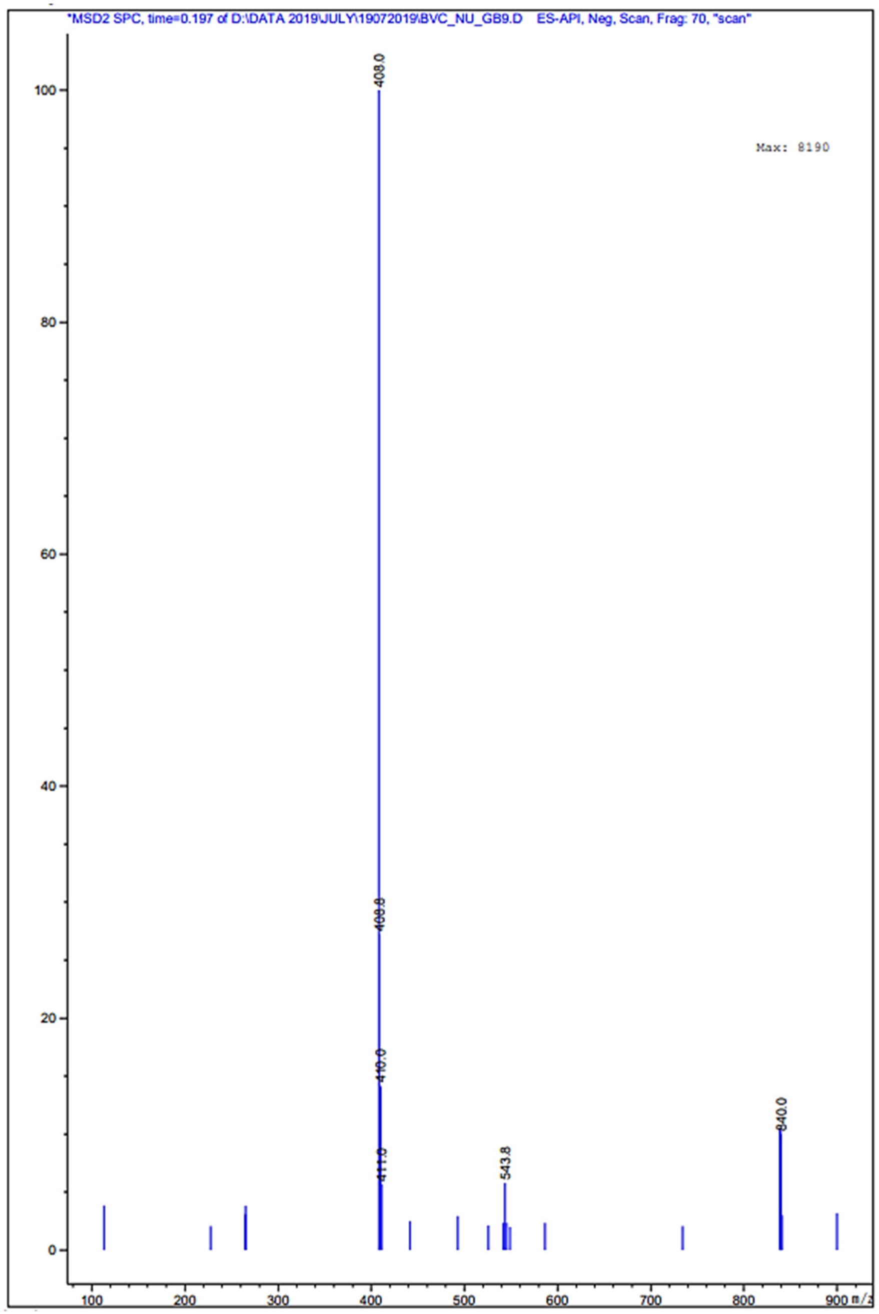
*MSD2 SPC, time=0.197 of D:\DATA 2019\JULY\19072019\BVC_NU_GB9.D ES-API, Neg, Scan, Frag: 70, "scan"
Max: 8190
408.0
408.8
410.0
411.0
543.8
840.0
m/z

6. UV

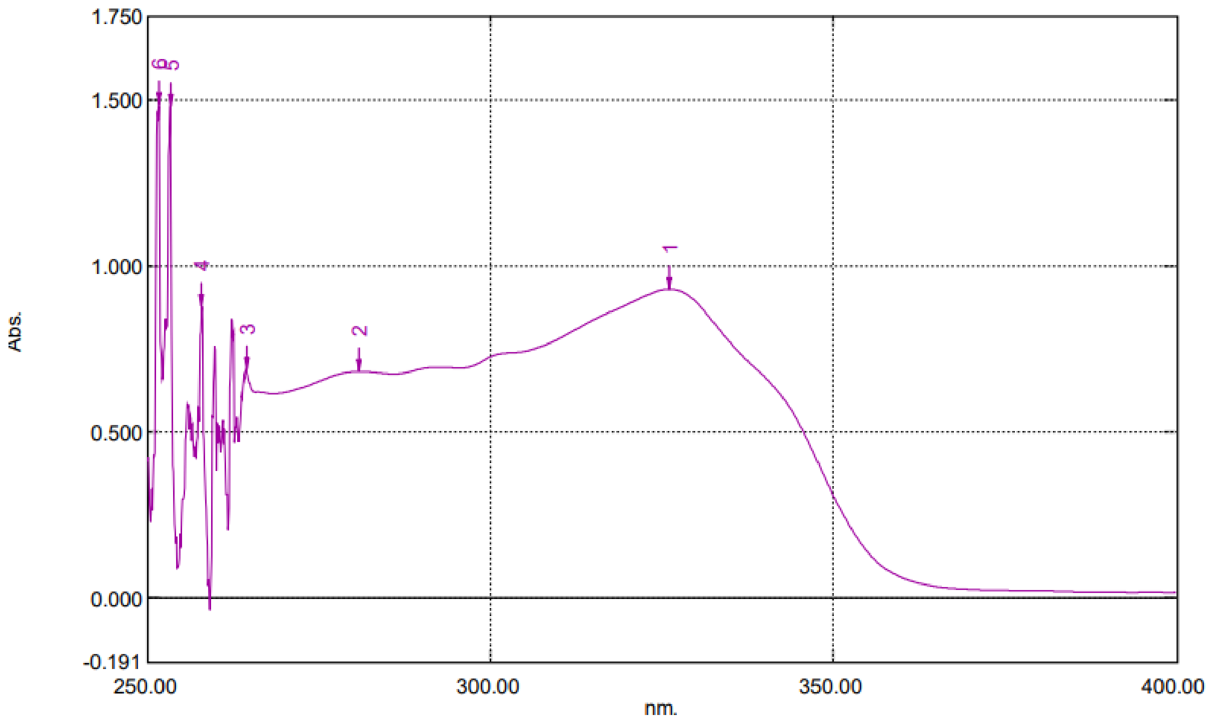

1.750
1.500
1.000
0.500
0.000
-0.191
Abs.
250.00
300.00
350.00
400.00
nm.
1
2
3
4
5
6

2-(5-(4-fluorobenzylidene)-2,4-dioxothiazolidin-3-yl)-N-(4-methylbenzo[d]thiazol-2-yl)acetamide (GB10)

1. FTIR

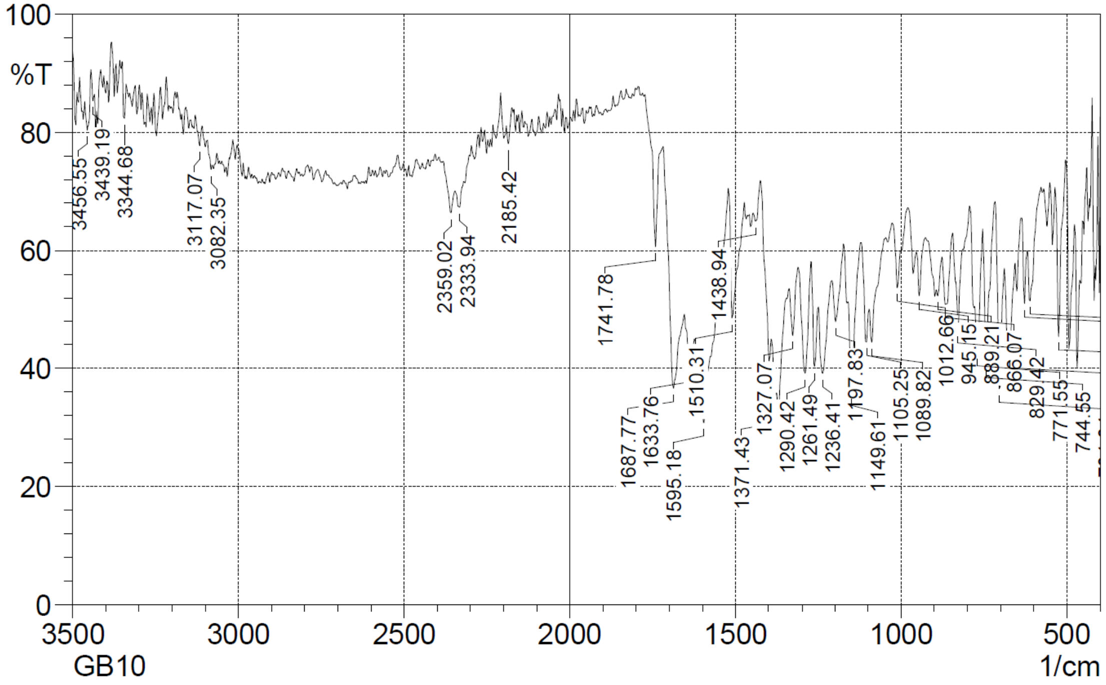

2. 1H-NMR

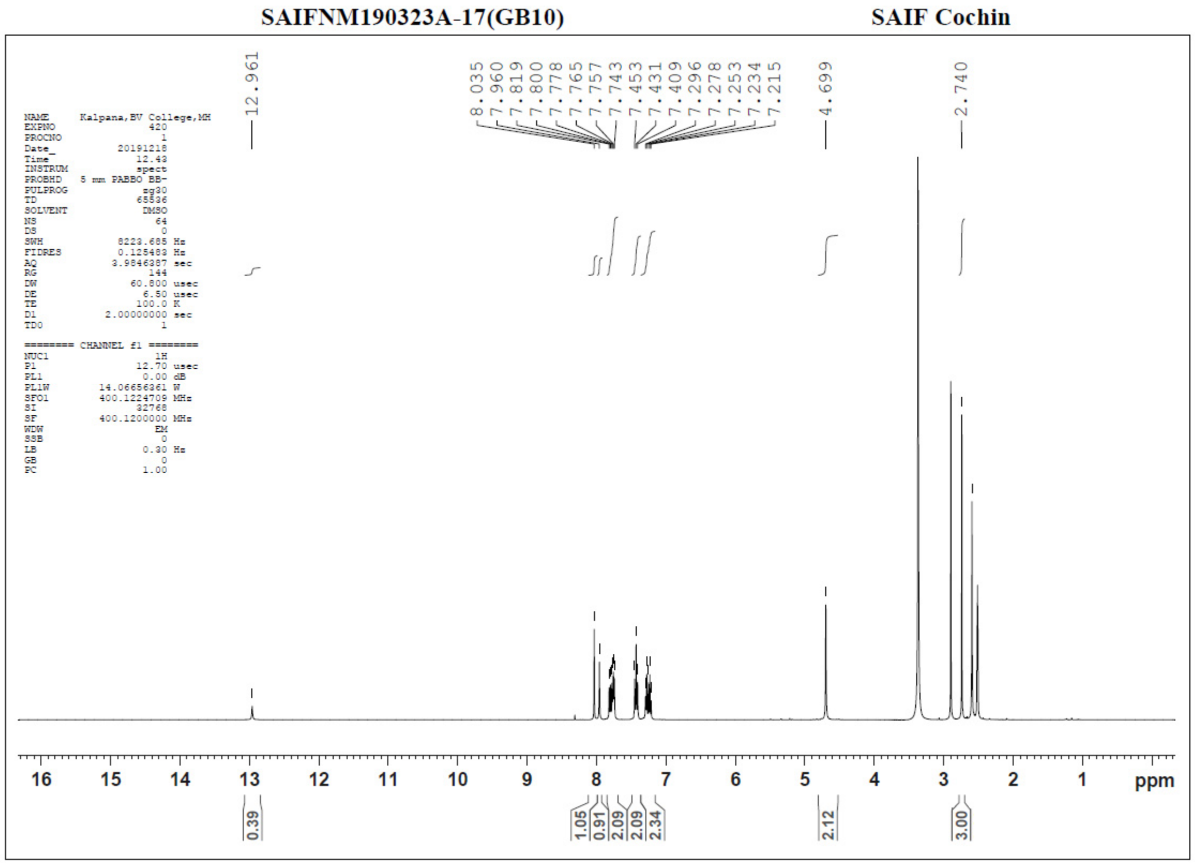
SAIFNM190323A-17(GB10)
SAIF Cochin
NAME Kalpana,BV College,MH
EXPNO 420
PROCNO 1
Date_ 20191218
Time 12.43
INSTRUM spect
PROBHD 5 mm PABBO BB-
PULPROG zg30
TD 65536
SOLVENT DMSO
NS 64
DS 0
SWH 8223.685 Hz
FIDRES 0.125483 Hz
AQ 3.9846387 sec
RG 144
DW 60.800 usec
DE 6.50 usec
TE 100.0 K
D1 2.00000000 sec
TD0 1
======== CHANNEL f1 ========
NUC1 1H
P1 12.70 usec
PL1 0.00 dB
PL1W 14.06656361 W
SFO1 400.1224709 MHz
SI 32768
SF 400.1200000 MHz
WDW EM
SSB 0
LB 0.30 Hz
GB 0
PC 1.00
12.961
8.035
7.960
7.819
7.800
7.778
7.765
7.757
7.743
7.453
7.431
7.409
7.296
7.278
7.253
7.234
7.215
4.699
2.740
16 15 14 13 12 11 10 9 8 7 6 5 4 3 2 1 ppm
0.39
1.05
0.91
2.09
2.09
2.34
2.12
3.00

3. 13C-NMR

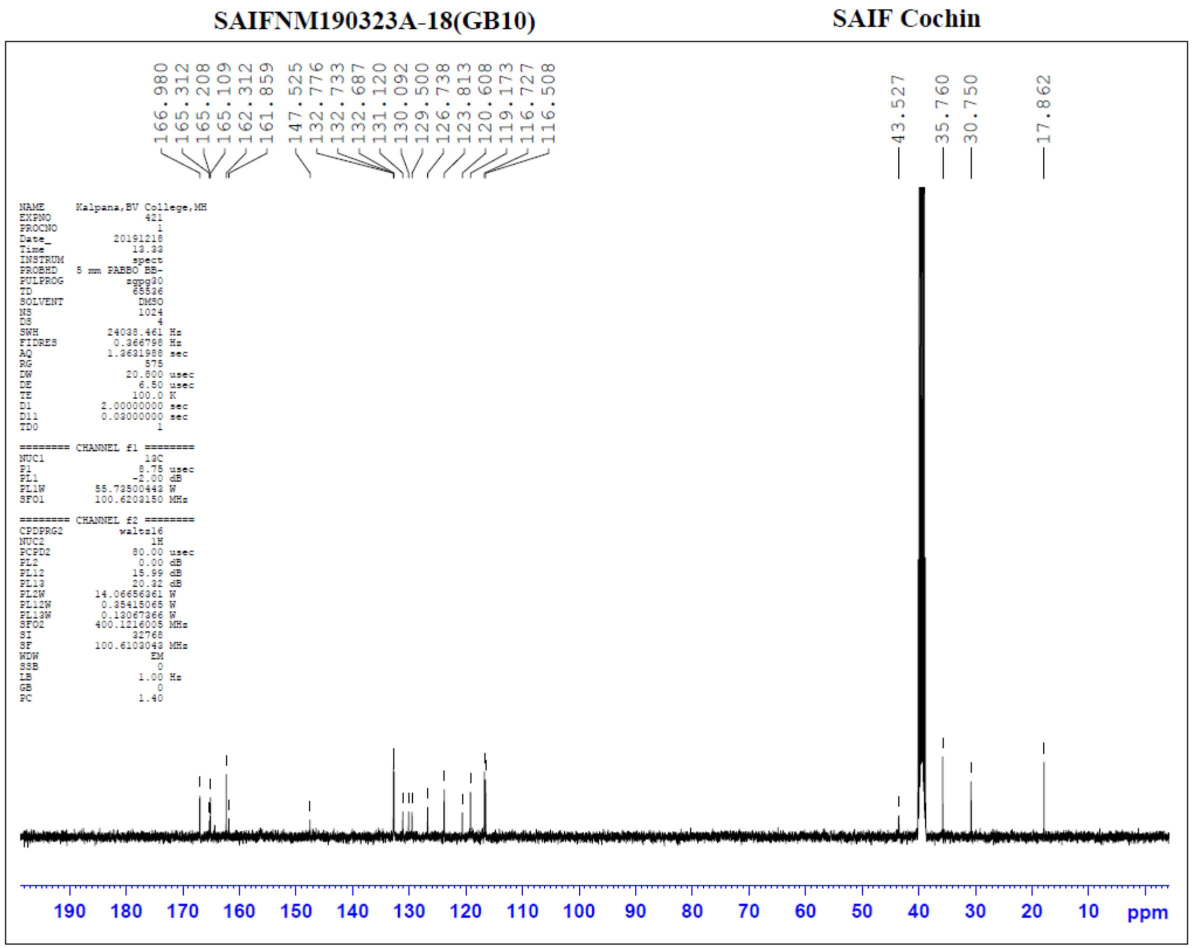
SAIFNM190323A-18(GB10)
SAIF Cochin
166.980
165.312
165.208
165.109
162.312
161.859
147.525
132.776
132.733
132.687
131.120
130.092
129.500
126.738
123.813
120.608
119.173
116.727
116.508
43.527
35.760
30.750
17.862
NAME Kalpana,BV College,MH
EXPNO 421
PROCNO 1
Date_ 20191218
Time 13.33
INSTRUM spect
PROBHD 5 mm PABBO BB-
PULPROG zgpg30
TD 65536
SOLVENT DMSO
NS 1024
DS 4
SWH 24038.461 Hz
FIDRES 0.366798 Hz
AQ 1.3631988 sec
RG 575
DW 20.800 usec
DE 6.50 usec
TE 100.0 K
D1 2.00000000 sec
D11 0.03000000 sec
TD0 1
======== CHANNEL f1 ========
NUC1 13C
P1 8.75 usec
PL1 -2.00 dB
PL1W 55.73500443 W
SFO1 100.6203150 MHz
======== CHANNEL f2 ========
CPDPRG2 waltz16
NUC2 1H
PCPD2 80.00 usec
PL2 0.00 dB
PL12 15.99 dB
PL13 20.32 dB
PL2W 14.06656361 W
PL12W 0.35415065 W
PL13W 0.13067366 W
SFO2 400.1216005 MHz
SI 32768
SF 100.6103043 MHz
WDW EM
SSB 0
LB 1.00 Hz
GB 0
PC 1.40
190 180 170 160 150 140 130 120 110 100 90 80 70 60 50 40 30 20 10 ppm

4. HPLC

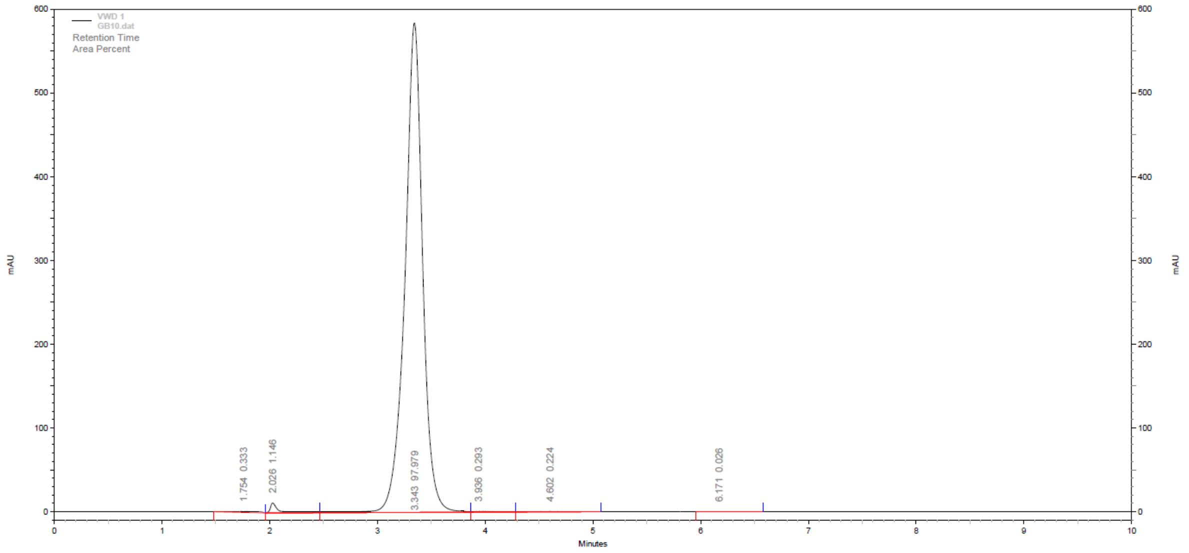
VWD 1
GB10.dat
Retention Time
Area Percent
mAU
Minutes
1.754 0.333
2.026 1.146
3.343 97.979
3.936 0.293
4.602 0.224
6.171 0.026

4. Mass

MS Spectrum

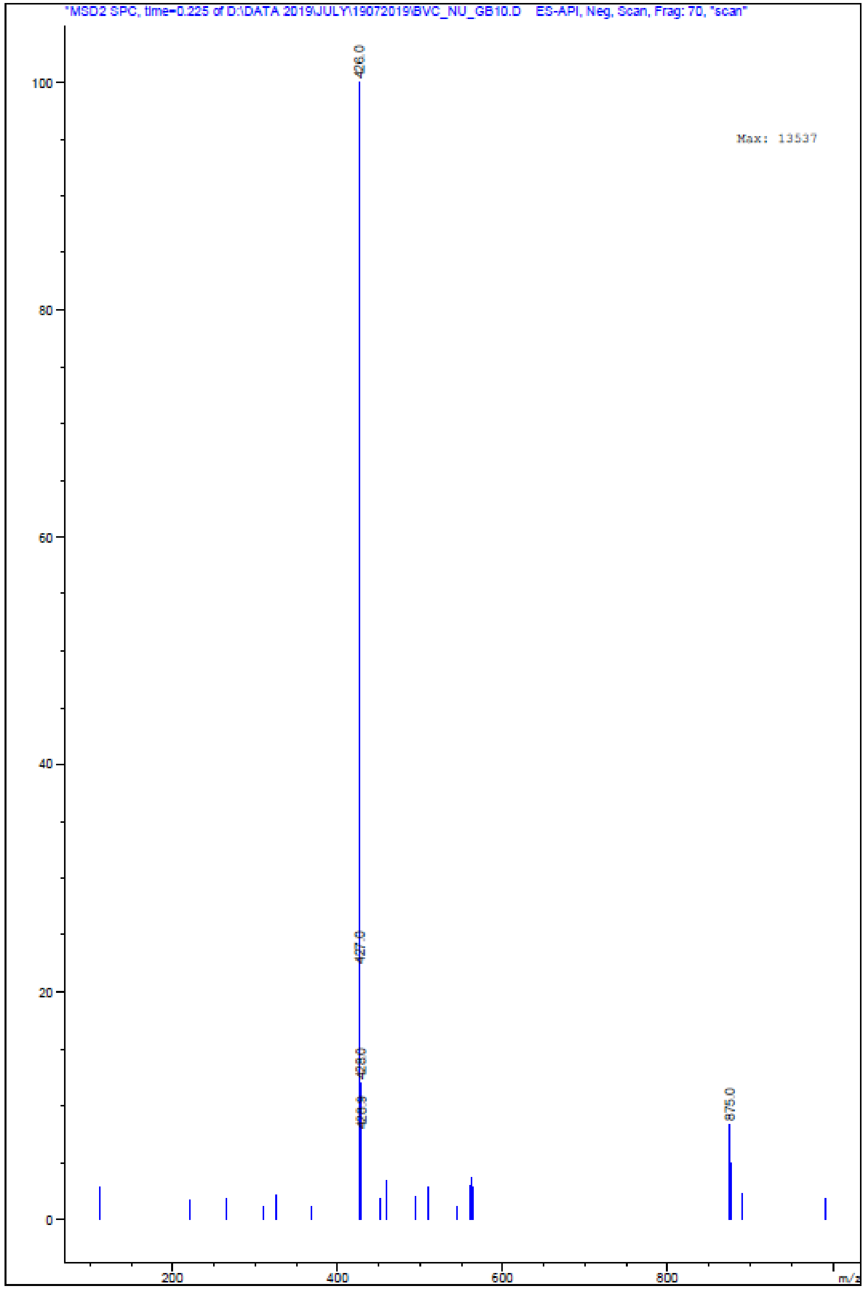

*MSD2 SPC, time=0.225 of D:\DATA 2019\JULY\19072019\BVC_NU_GB10.D ES-API, Neg, Scan, Frag: 70, "scan"
Max: 13537
426.0
427.0
428.0
875.0
100
80
60
40
20
0
200
400
600
800
m/z

2-(5-(4-chlorobenzylidene)-2,4-dioxothiazolidin-3-yl)-N-(4,6-difluorobenzo[d]thiazol-2-yl)acetamide (GB11)

1. FTIR

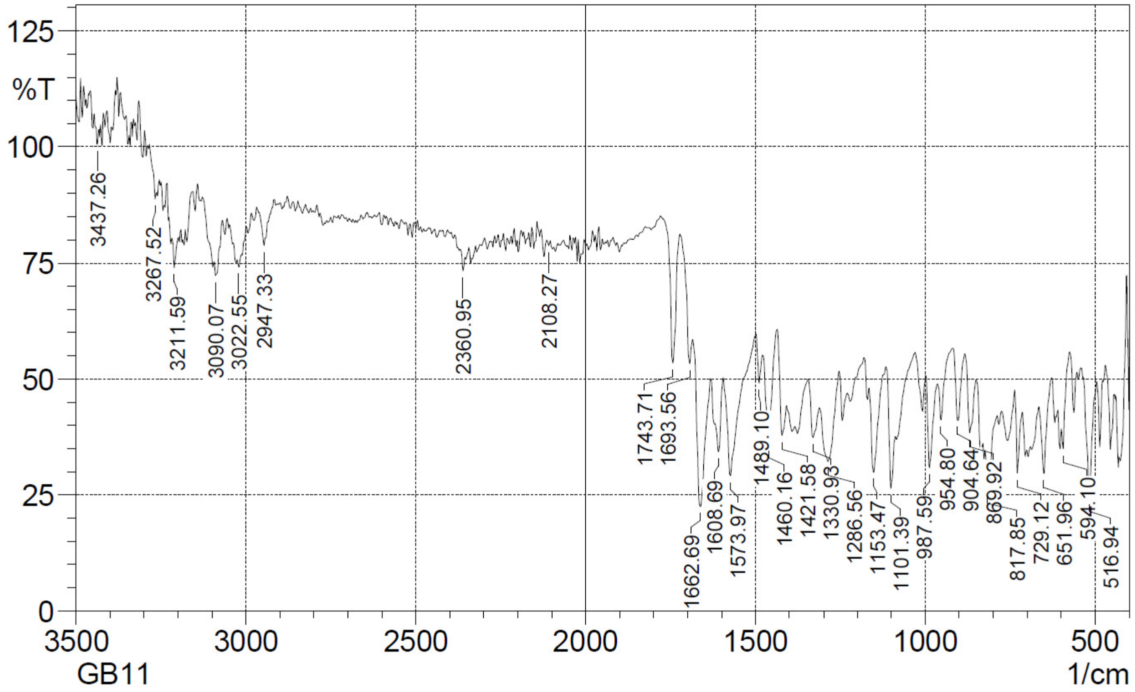

2. 1H-NMR

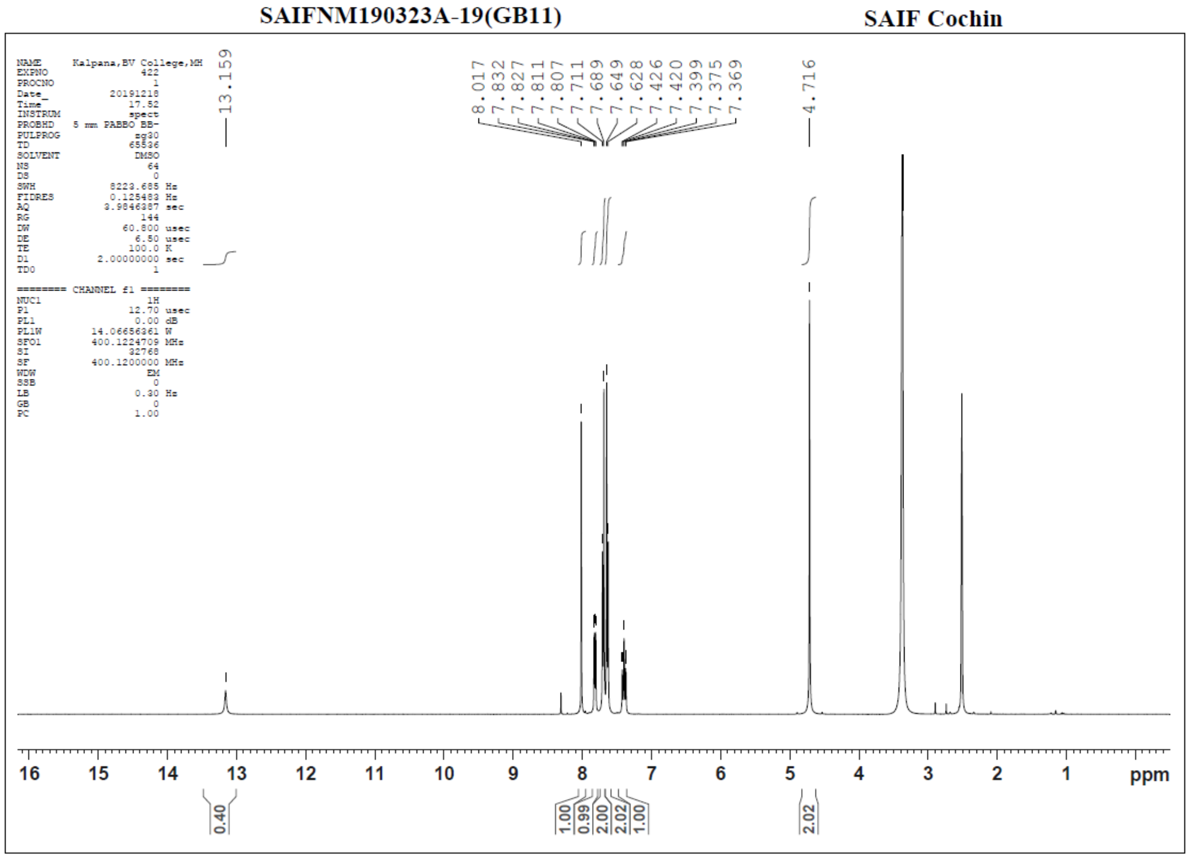
SAIFNM190323A-19(GB11)
SAIF Cochin
NAME Kalpana,BV College,MH
EXPNO 422
PROCNO 1
Date_ 20191218
Time 17.52
INSTRUM spect
PROBHD 5 mm PABBO BB-
PULPROG zg30
TD 65536
SOLVENT DMSO
NS 64
DS 0
SWH 8223.685 Hz
FIDRES 0.125483 Hz
AQ 3.9846387 sec
RG 144
DW 60.800 usec
DE 6.50 usec
TE 100.0 K
D1 2.00000000 sec
TD0 1
======== CHANNEL f1 ========
NUC1 1H
P1 12.70 usec
PL1 0.00 dB
PL1W 14.06656361 W
SFO1 400.1224709 MHz
SI 32768
SF 400.1200000 MHz
WDW EM
SSB 0
LB 0.30 Hz
GB 0
PC 1.00
13.159
8.017
7.832
7.827
7.811
7.807
7.711
7.689
7.649
7.628
7.426
7.420
7.399
7.375
7.369
4.716
16 15 14 13 12 11 10 9 8 7 6 5 4 3 2 1 ppm
0.40
1.00
0.99
2.00
2.02
1.00
2.02

3. 13C-NMR

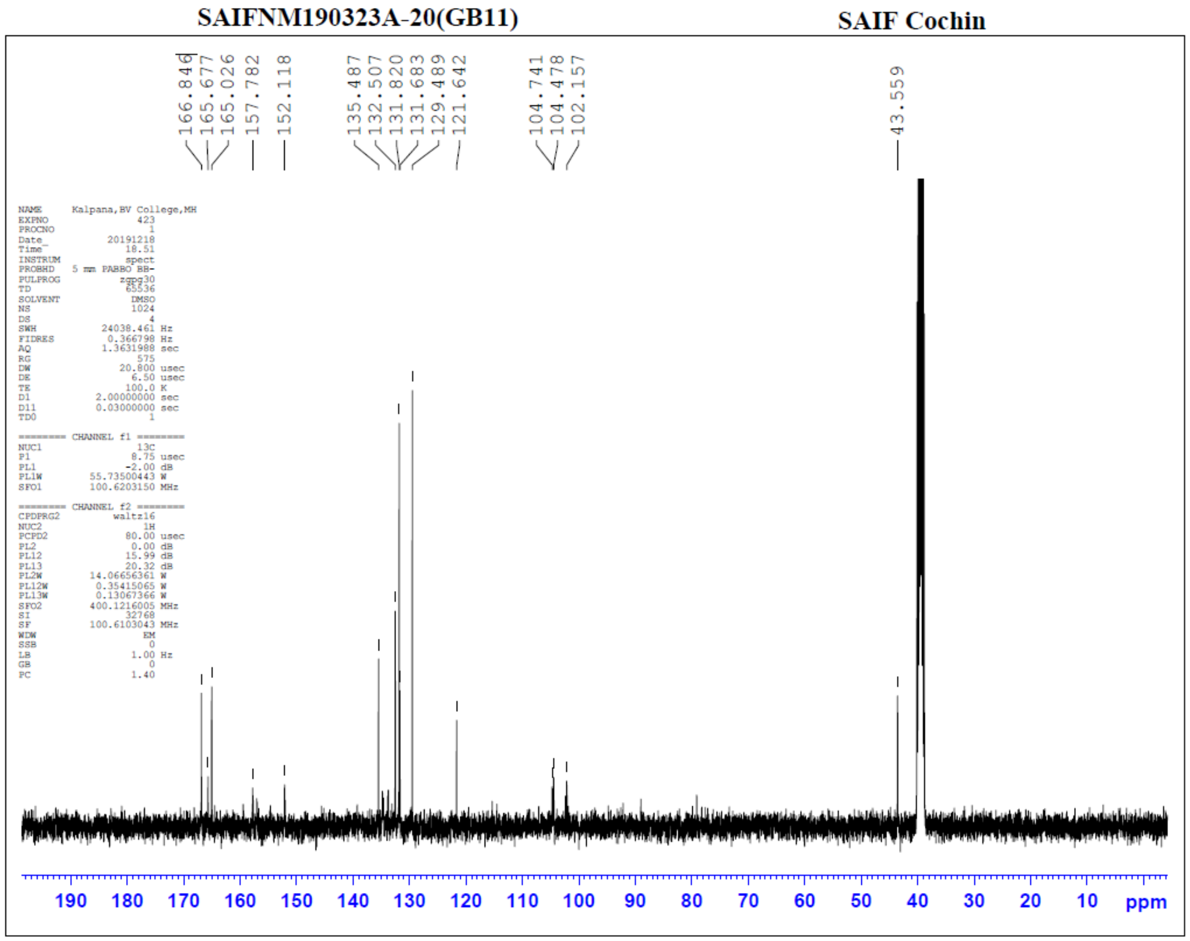
SAIFNM190323A-20(GB11)
SAIF Cochin
166.846
165.677
165.026
157.782
152.118
135.487
132.507
131.820
131.683
129.489
121.642
104.741
104.478
102.157
43.559
NAME Kalpana,BV College,MH
EXPNO 423
PROCNO 1
Date_ 20191218
Time 18.51
INSTRUM spect
PROBHD 5 mm PABBO BB-
PULPROG zgpg30
TD 65536
SOLVENT DMSO
NS 1024
DS 4
SWH 24038.461 Hz
FIDRES 0.366798 Hz
AQ 1.3631988 sec
RG 575
DW 20.800 usec
DE 6.50 usec
TE 100.0 K
D1 2.00000000 sec
D11 0.03000000 sec
TD0 1
======== CHANNEL f1 ========
NUC1 13C
P1 8.75 usec
PL1 -2.00 dB
PL1W 55.73500443 W
SFO1 100.6203150 MHz
======== CHANNEL f2 ========
CPDPRG2 waltz16
NUC2 1H
PCPD2 80.00 usec
PL2 0.00 dB
PL12 15.99 dB
PL13 20.32 dB
PL2W 14.06656361 W
PL12W 0.35415065 W
PL13W 0.13067366 W
SFO2 400.1216005 MHz
SI 32768
SF 100.6103043 MHz
WDW EM
SSB 0
LB 1.00 Hz
GB 0
PC 1.40
190 180 170 160 150 140 130 120 110 100 90 80 70 60 50 40 30 20 10 ppm

4. HPLC

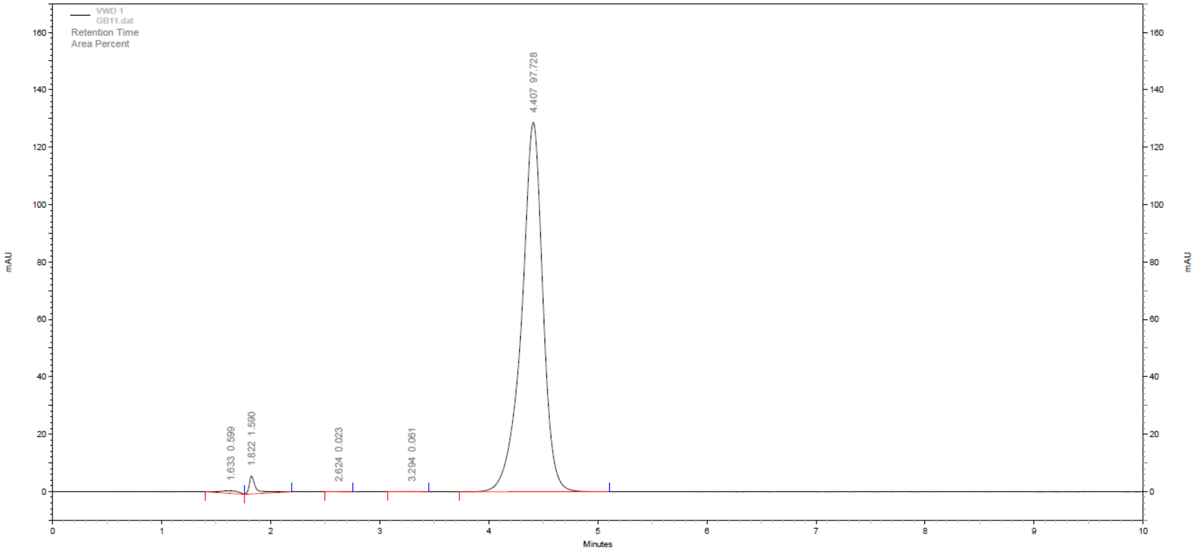
VWD 1
GB11.dat
Retention Time
Area Percent
4.407 97.728
1.633 0.599
1.822 1.590
2.624 0.023
3.294 0.061
mAU
Minutes

5. Mass

MS Spectrum

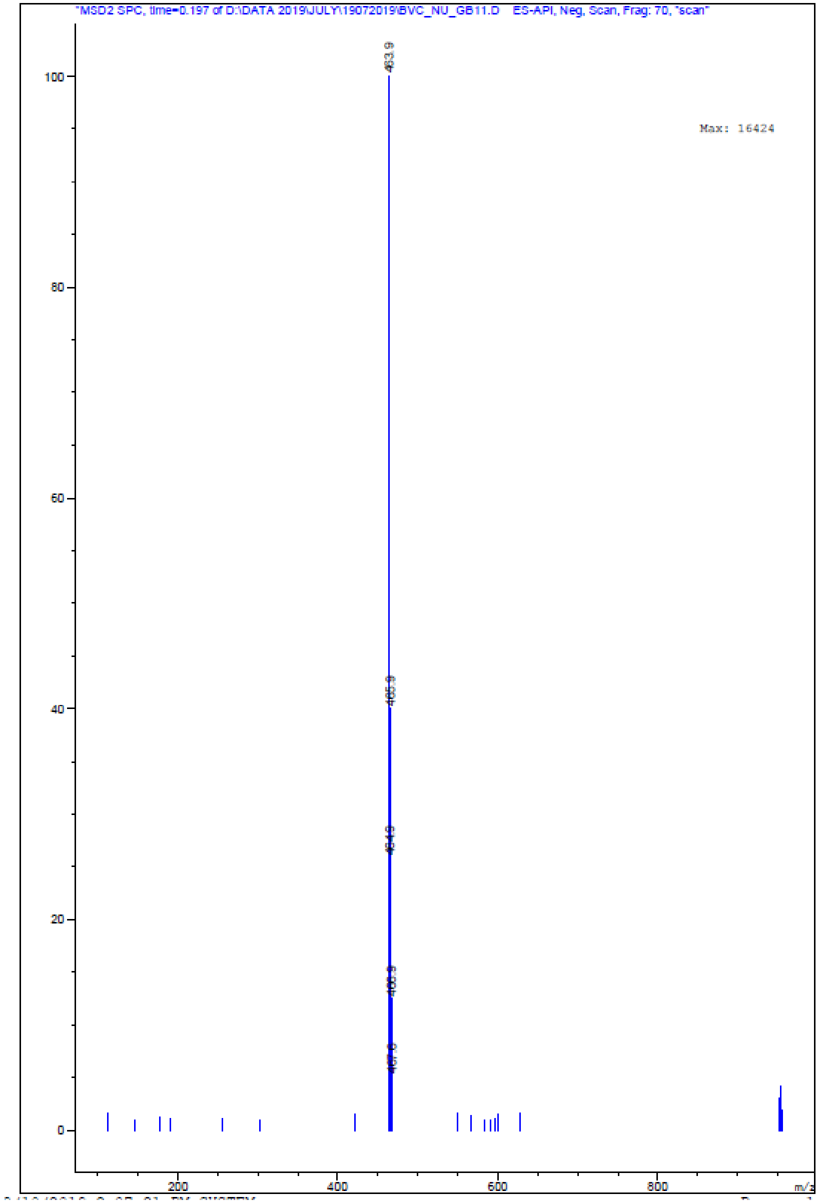

"MSD2 SPC, time=0.197 of D:\DATA 2019\JULY\19072019\BVC_NU_GB11.D ES-API, Neg, Scan, Frag: 70, "scan"
463.9
Max: 16424
100
80
60
40
20
0
200
400
600
800
m/z

2-(5-benzylidene-2,4-dioxothiazolidin-3-yl)-N-(4,6-difluorobenzo[d]thiazol-2-yl)acetamide (GB12)

1. FTIR

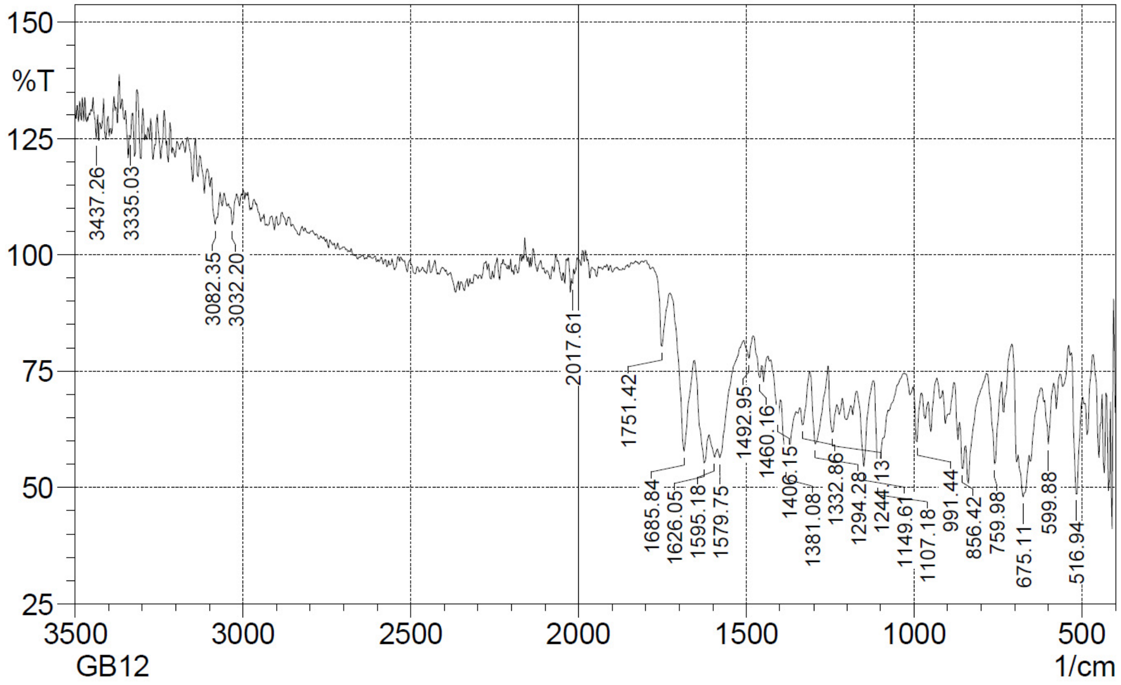

2. 1H-NMR

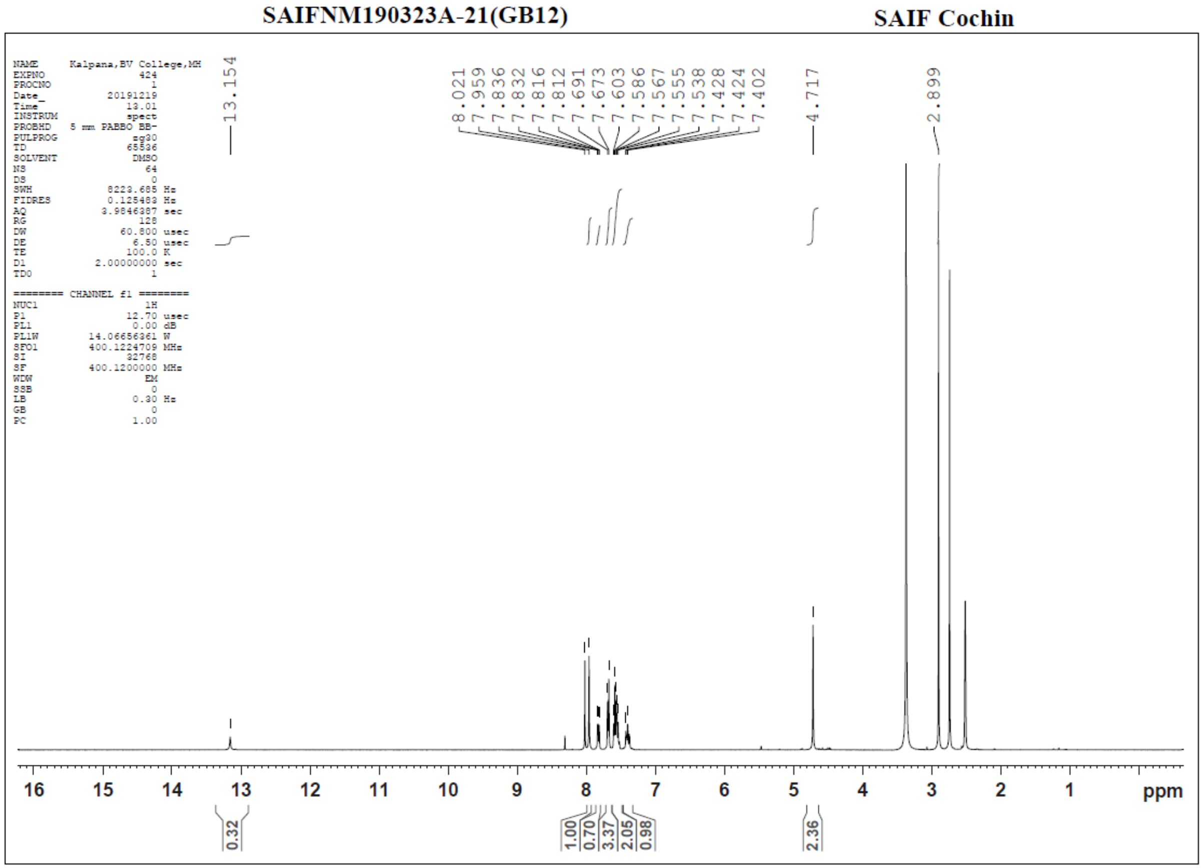
SAIFNM190323A-21(GB12)
SAIF Cochin
NAME Kalpana,BV College,MH
EXPNO 424
PROCNO 1
Date_ 20191219
Time 13.01
INSTRUM spect
PROBHD 5 mm PABBO BB-
PULPROG zg30
TD 65536
SOLVENT DMSO
NS 64
DS 0
SWH 8223.685 Hz
FIDRES 0.125483 Hz
AQ 3.9846387 sec
RG 128
DW 60.800 usec
DE 6.50 usec
TE 100.0 K
D1 2.00000000 sec
TD0 1
======== CHANNEL f1 ========
NUC1 1H
P1 12.70 usec
PL1 0.00 dB
PL1W 14.06656361 W
SFO1 400.1224709 MHz
SI 32768
SF 400.1200000 MHz
WDW EM
SSB 0
LB 0.30 Hz
GB 0
PC 1.00
13.154
8.021
7.959
7.836
7.832
7.816
7.812
7.691
7.673
7.603
7.586
7.567
7.555
7.538
7.428
7.424
7.402
4.717
2.899
16 15 14 13 12 11 10 9 8 7 6 5 4 3 2 1 ppm
0.32
1.00
0.70
3.37
2.05
0.98
2.36

3. 13C-NMR

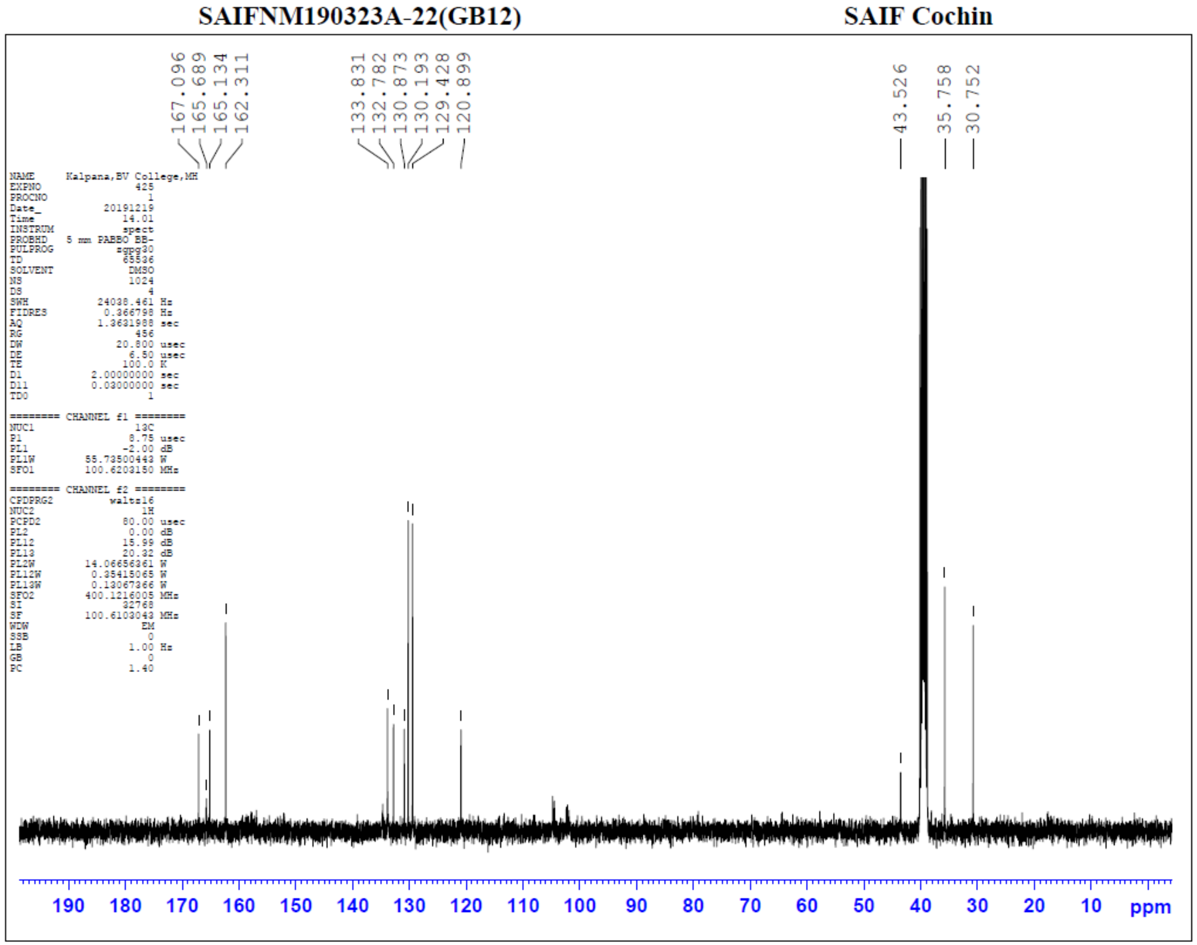
SAIFNM190323A-22(GB12)
SAIF Cochin
167.096
165.689
165.134
162.311
133.831
132.782
130.873
130.193
129.428
120.899
43.526
35.758
30.752
NAME Kalpana,BV College,MH
EXPNO 425
PROCNO 1
Date_ 20191219
Time 14.01
INSTRUM spect
PROBHD 5 mm PABBO BB-
PULPROG zgpg30
TD 65536
SOLVENT DMSO
NS 1024
DS 4
SWH 24038.461 Hz
FIDRES 0.366798 Hz
AQ 1.3631988 sec
RG 456
DW 20.800 usec
DE 6.50 usec
TE 100.0 K
D1 2.00000000 sec
D11 0.03000000 sec
TD0 1
======== CHANNEL f1 ========
NUC1 13C
P1 8.75 usec
PL1 -2.00 dB
PL1W 55.73500443 W
SFO1 100.6203150 MHz
======== CHANNEL f2 ========
CPDPRG2 waltz16
NUC2 1H
PCPD2 80.00 usec
PL2 0.00 dB
PL12 15.99 dB
PL13 20.32 dB
PL2W 14.06656361 W
PL12W 0.35415065 W
PL13W 0.13067366 W
SFO2 400.1216005 MHz
SI 32768
SF 100.6103043 MHz
WDW EM
SSB 0
LB 1.00 Hz
GB 0
PC 1.40
190 180 170 160 150 140 130 120 110 100 90 80 70 60 50 40 30 20 10 ppm

4. HPLC

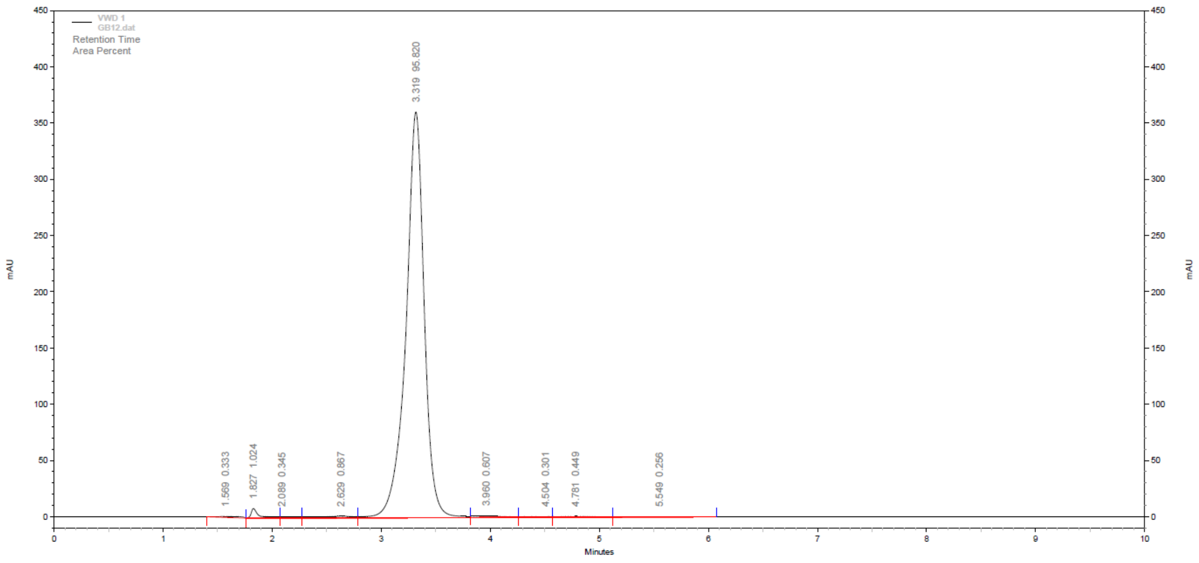

VWD 1
GB12.dat
Retention Time
Area Percent
3.319 95.820
1.569 0.333
1.827 1.024
2.089 0.345
2.629 0.867
3.960 0.607
4.504 0.301
4.781 0.449
5.549 0.256
mAU
Minutes

5. Mass

MS Spectrum

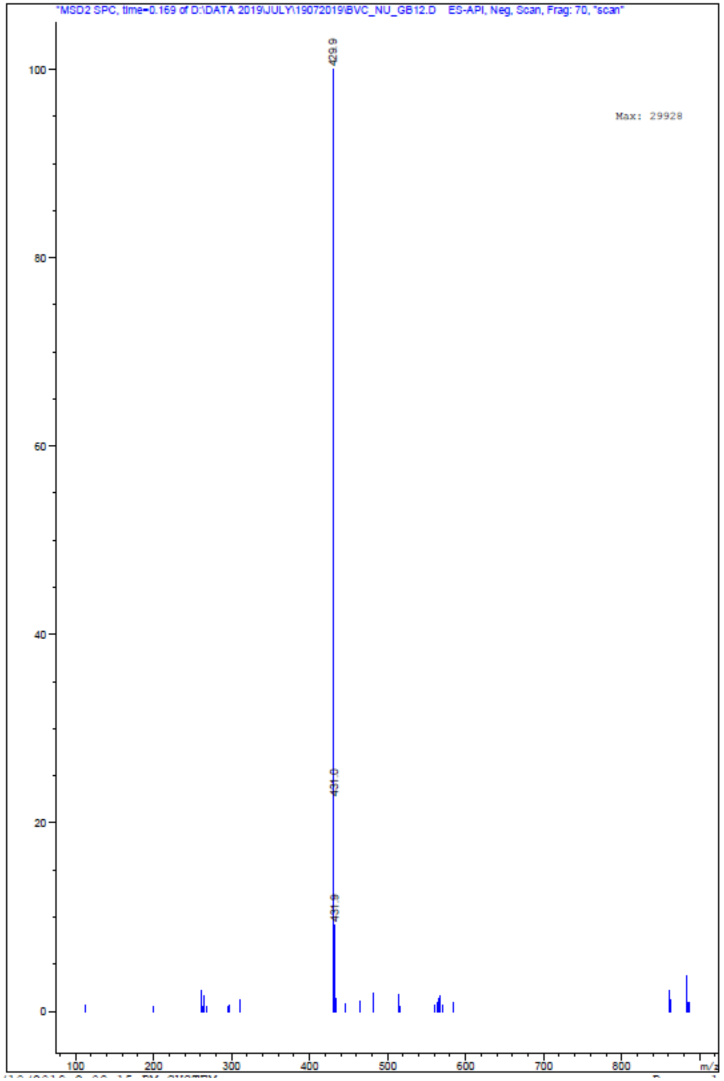

*MSD2 SPC, time=0.169 of D:\DATA 2019\JULY\19072019\BVC_NU_GB12.D ES-API, Neg, Scan, Frag: 70, "scan"
429.9
Max: 29928
431.0
431.9
100
80
60
40
20
0
100
200
300
400
500
600
700
800
m/z

2-(5-(2,4-difluorobenzylidene)-2,4-dioxothiazolidin-3-yl)-N-(6-methylbenzo[d]thiazol-2-yl)acetamide (GB13)

1. FTIR

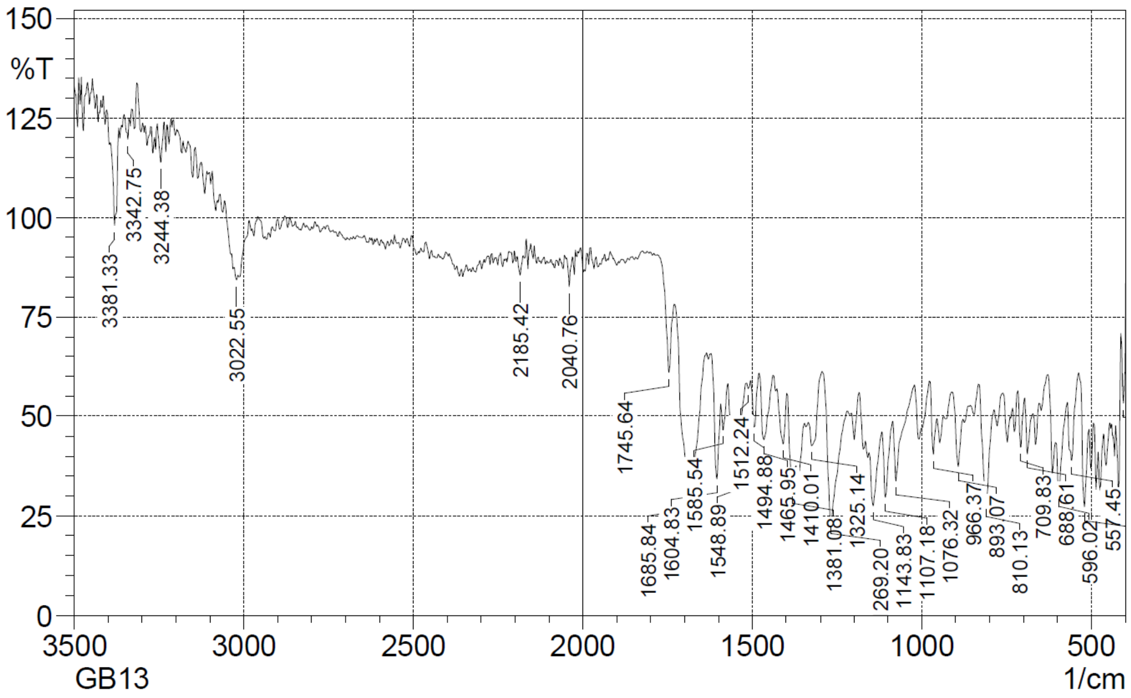

2. 1H-NMR

SAIFNM190323A-23(GB13) SAIF Cochin

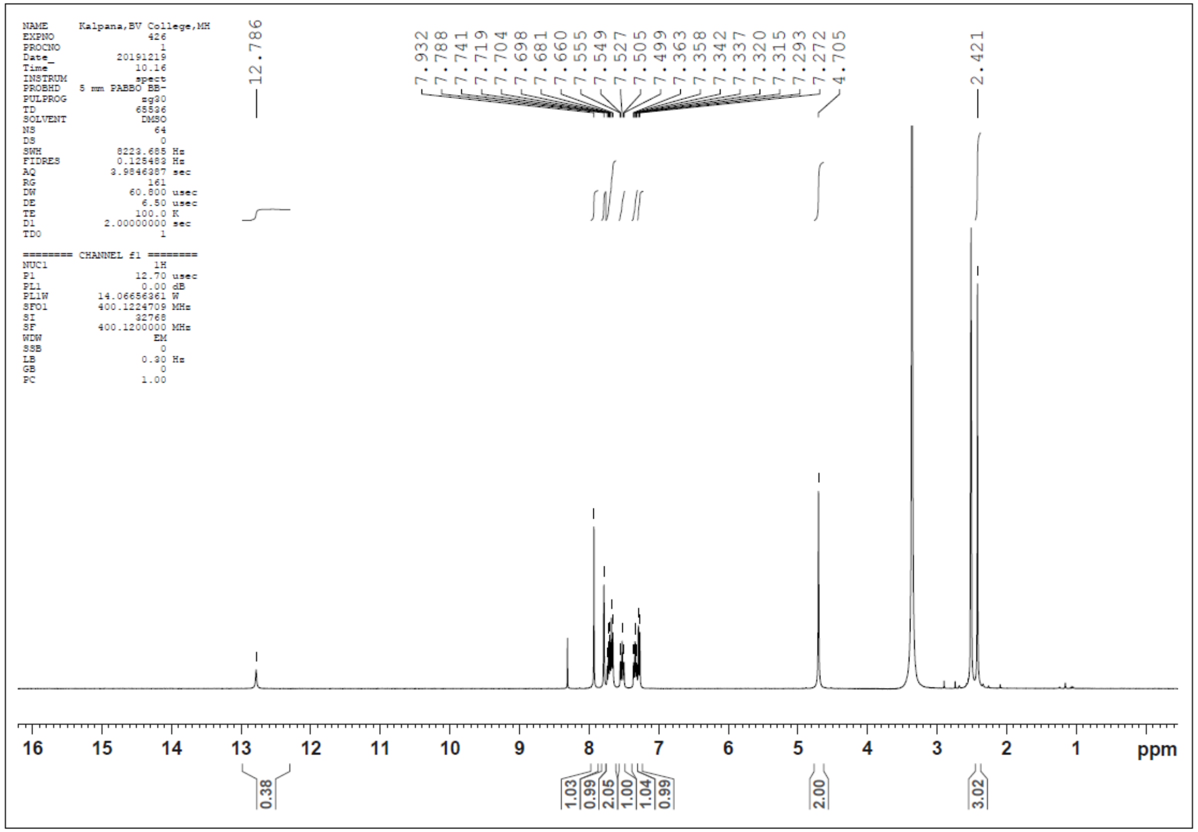

3. 13C-NMR

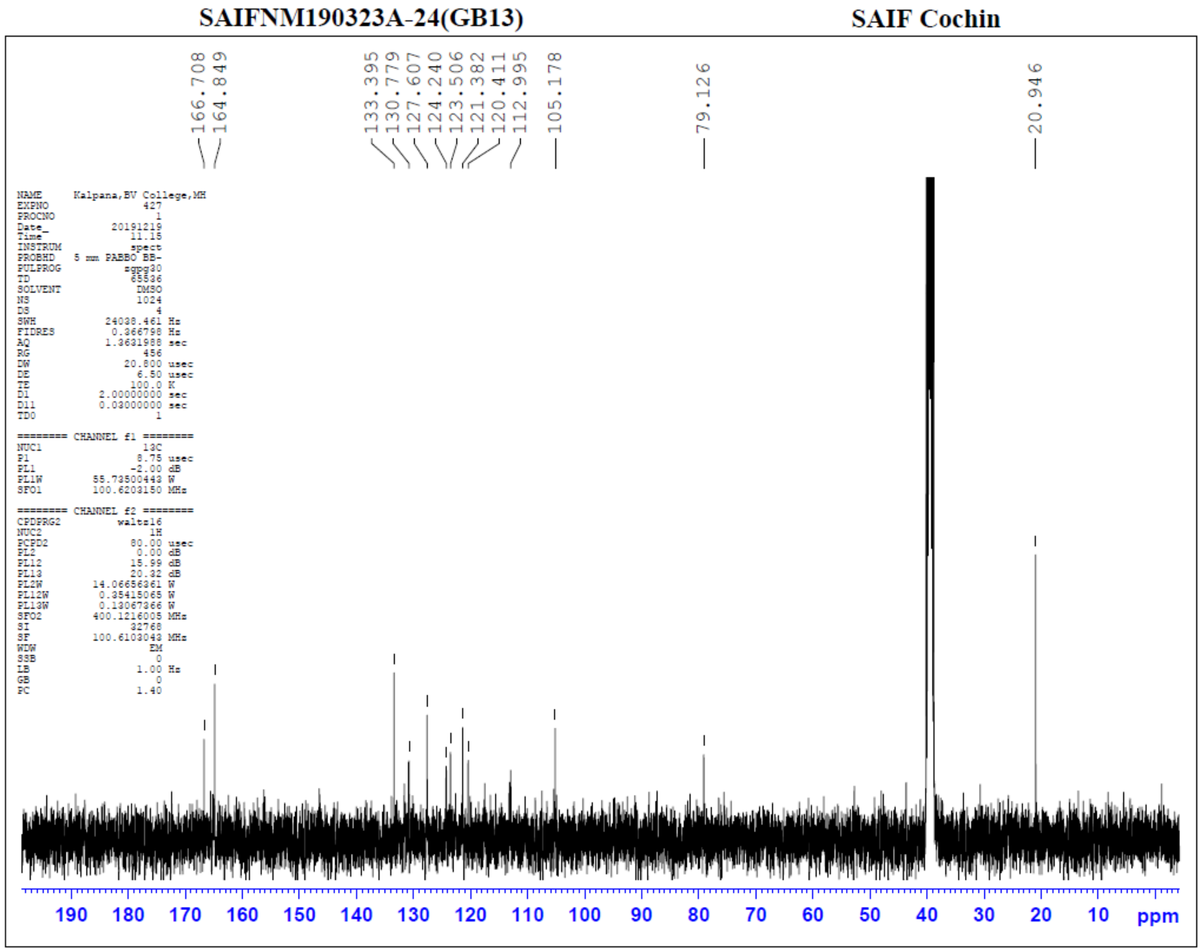
SAIFNM190323A-24(GB13)
SAIF Cochin
166.708
164.849
133.395
130.779
127.607
124.240
123.506
121.382
120.411
112.995
105.178
79.126
20.946
NAME Kalpana,BV College,MH
EXPNO 427
PROCNO 1
Date_ 20191219
Time 11.15
INSTRUM spect
PROBHD 5 mm PABBO BB-
PULPROG zgpg30
TD 65536
SOLVENT DMSO
NS 1024
DS 4
SWH 24038.461 Hz
FIDRES 0.366798 Hz
AQ 1.3631988 sec
RG 456
DW 20.800 usec
DE 6.50 usec
TE 100.0 K
D1 2.00000000 sec
D11 0.03000000 sec
TD0 1
======== CHANNEL f1 ========
NUC1 13C
P1 8.75 usec
PL1 -2.00 dB
PL1W 55.73500443 W
SFO1 100.6203150 MHz
======== CHANNEL f2 ========
CPDPRG2 waltz16
NUC2 1H
PCPD2 80.00 usec
PL2 0.00 dB
PL12 15.99 dB
PL13 20.32 dB
PL2W 14.06656361 W
PL12W 0.35415065 W
PL13W 0.13067366 W
SFO2 400.1216005 MHz
SI 32768
SF 100.6103043 MHz
WDW EM
SSB 0
LB 1.00 Hz
GB 0
PC 1.40
190 180 170 160 150 140 130 120 110 100 90 80 70 60 50 40 30 20 10 ppm

4. HPLC

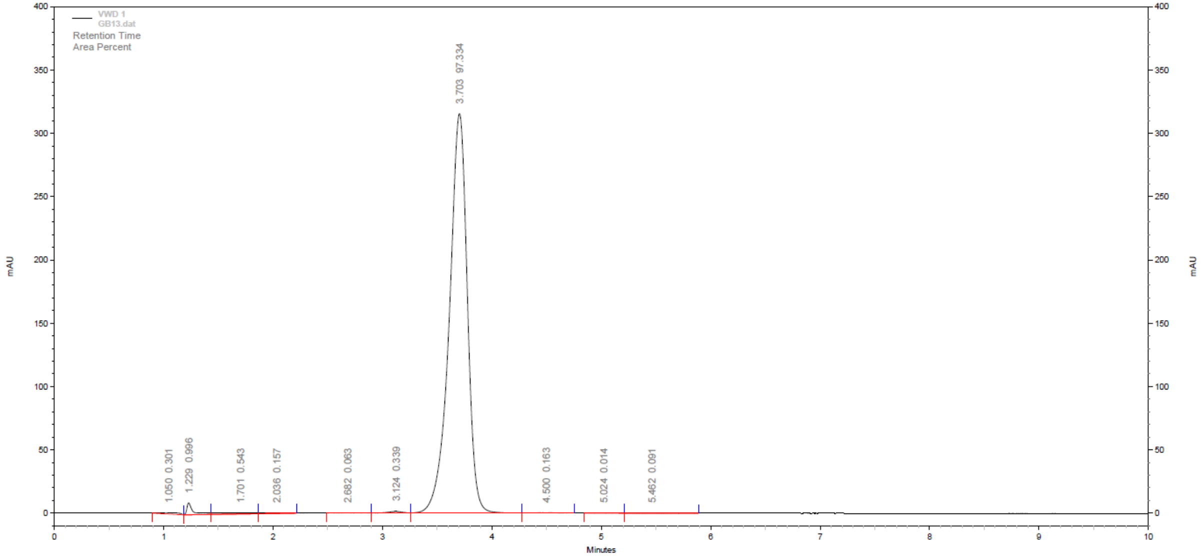
VWD 1
GB13.dat
Retention Time
Area Percent
3.703 97.334
1.050 0.301
1.229 0.996
1.701 0.543
2.036 0.157
2.682 0.063
3.124 0.339
4.500 0.163
5.024 0.014
5.462 0.091
mAU
Minutes

5. Mass

MS Spectrum

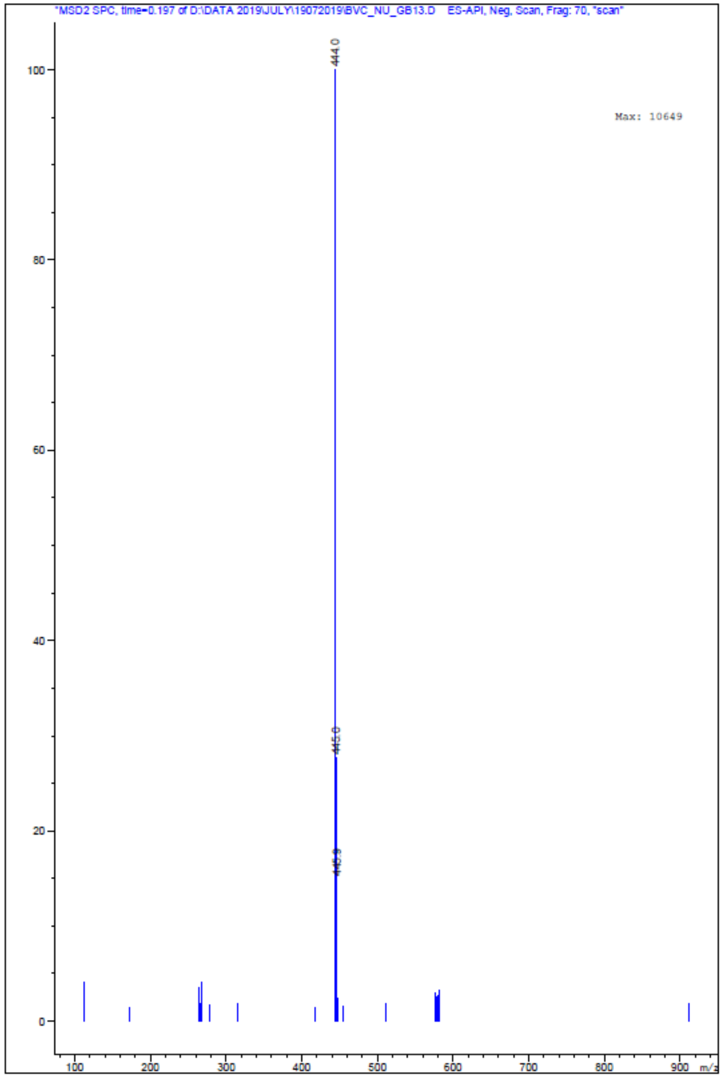
"MSD2 SPC, time=0.197 of D:\DATA 2019\JULY\19072019\BVC_NU_GB13.D ES-API, Neg, Scan, Frag: 70, "scan"
444.0
445.0
Max: 10649
100
80
60
40
20
0
100
200
300
400
500
600
700
800
900
m/z

N-(6-methylbenzo[d]thiazol-2-yl)-2-(5-(4-methylbenzylidene)-2,4-dioxothiazolidin-3-yl)acetamide (GB14)

1. FTIR

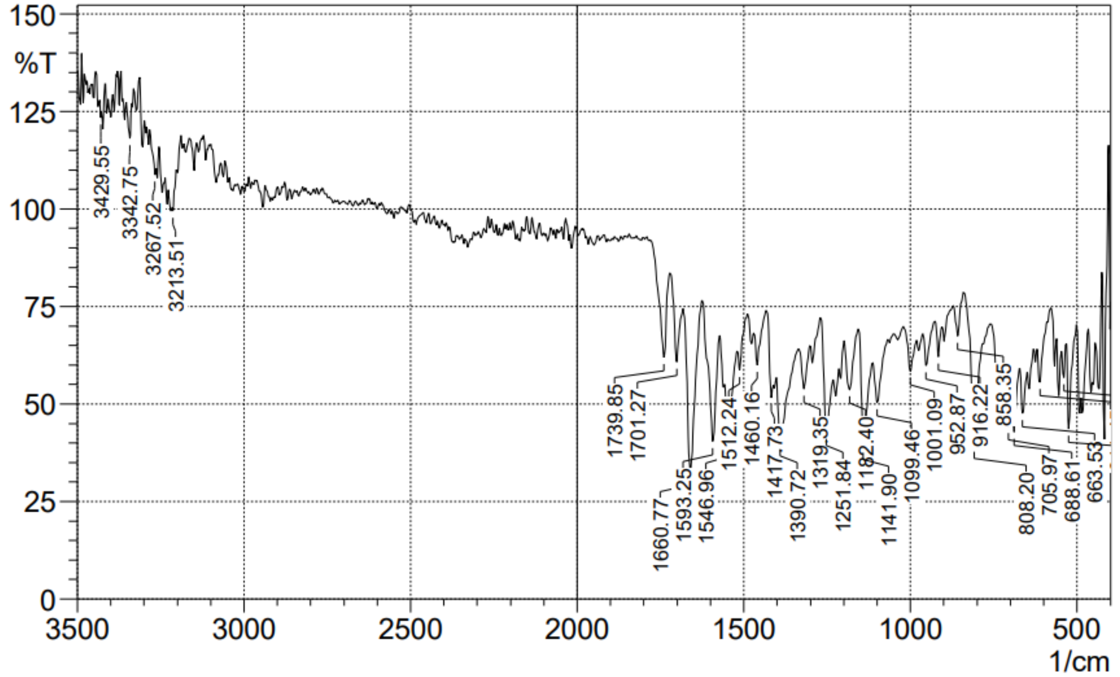

2. ^{1}H-NMR

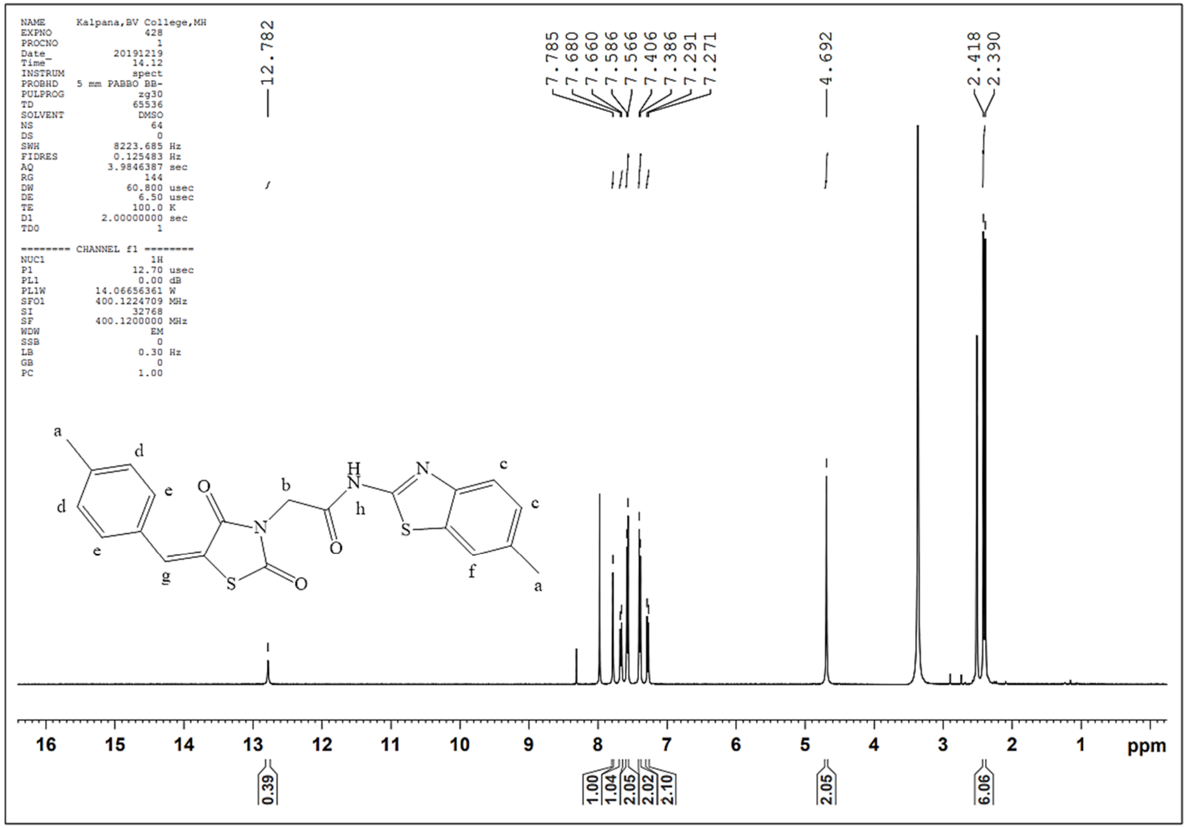
NAME Kalpana,BV College,MH
EXPNO 428
PROCNO 1
Date_ 20191219
Time 14.12
INSTRUM spect
PROBHD 5 mm PABBO BB-
PULPROG zg30
TD 65536
SOLVENT DMSO
NS 64
DS 0
SWH 8223.685 Hz
FIDRES 0.125483 Hz
AQ 3.9846387 sec
RG 144
DW 60.800 usec
DE 6.50 usec
TE 100.0 K
D1 2.00000000 sec
TD0 1
======== CHANNEL f1 ========
NUC1 1H
P1 12.70 usec
PL1 0.00 dB
PL1W 14.06656361 W
SFO1 400.1224709 MHz
SI 32768
SF 400.1200000 MHz
WDW EM
SSB 0
LB 0.30 Hz
GB 0
PC 1.00
12.782
7.785
7.680
7.660
7.586
7.566
7.406
7.386
7.291
7.271
4.692
2.418
2.390
ppm
0.39
1.00
1.04
2.05
2.02
2.10
2.05
6.06

3. ^{13}C-NMR

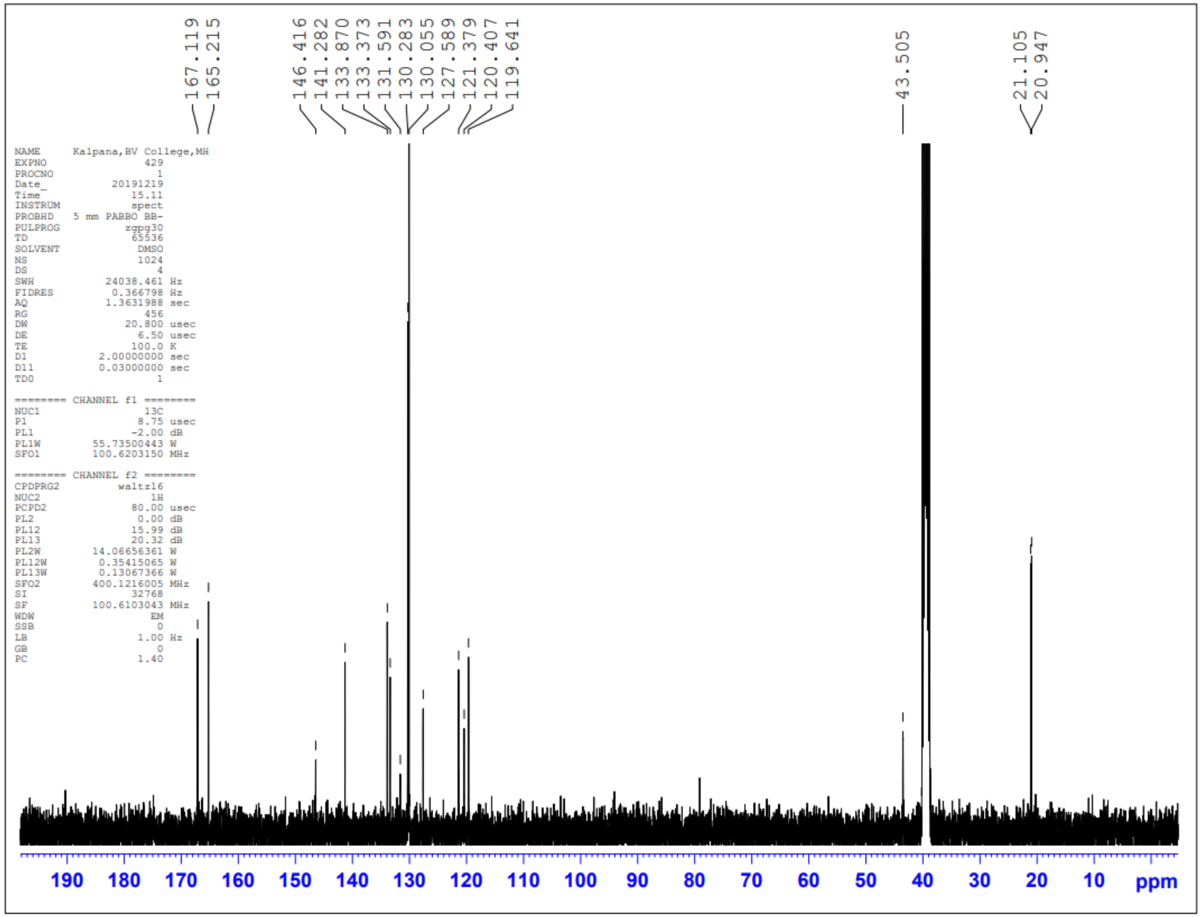

4. HPLC Analysis

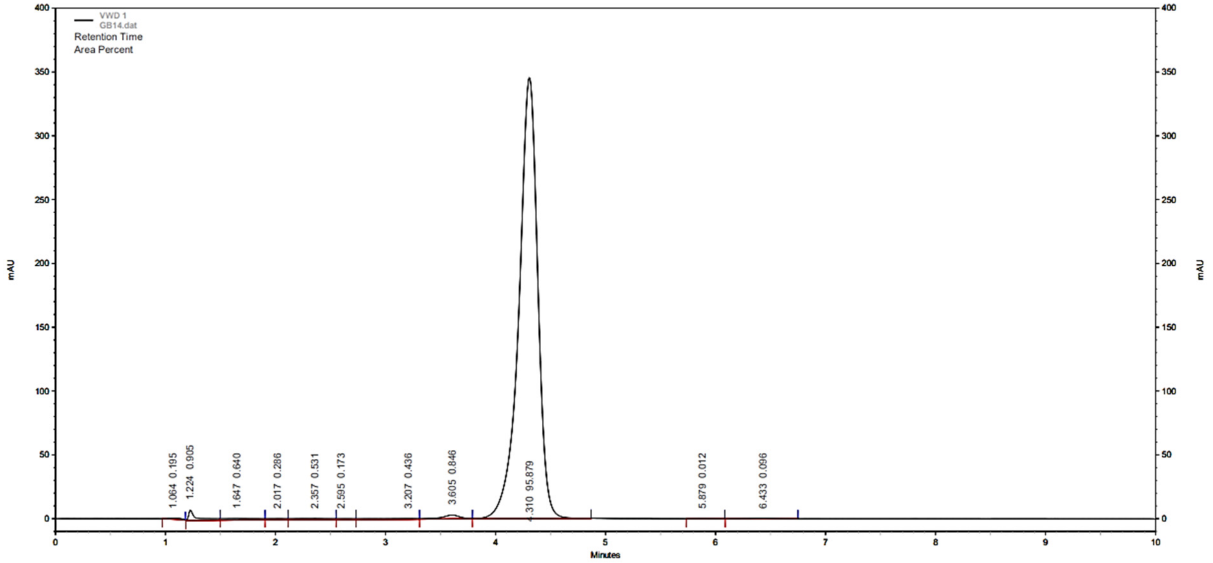

5. Mass Spectrometry

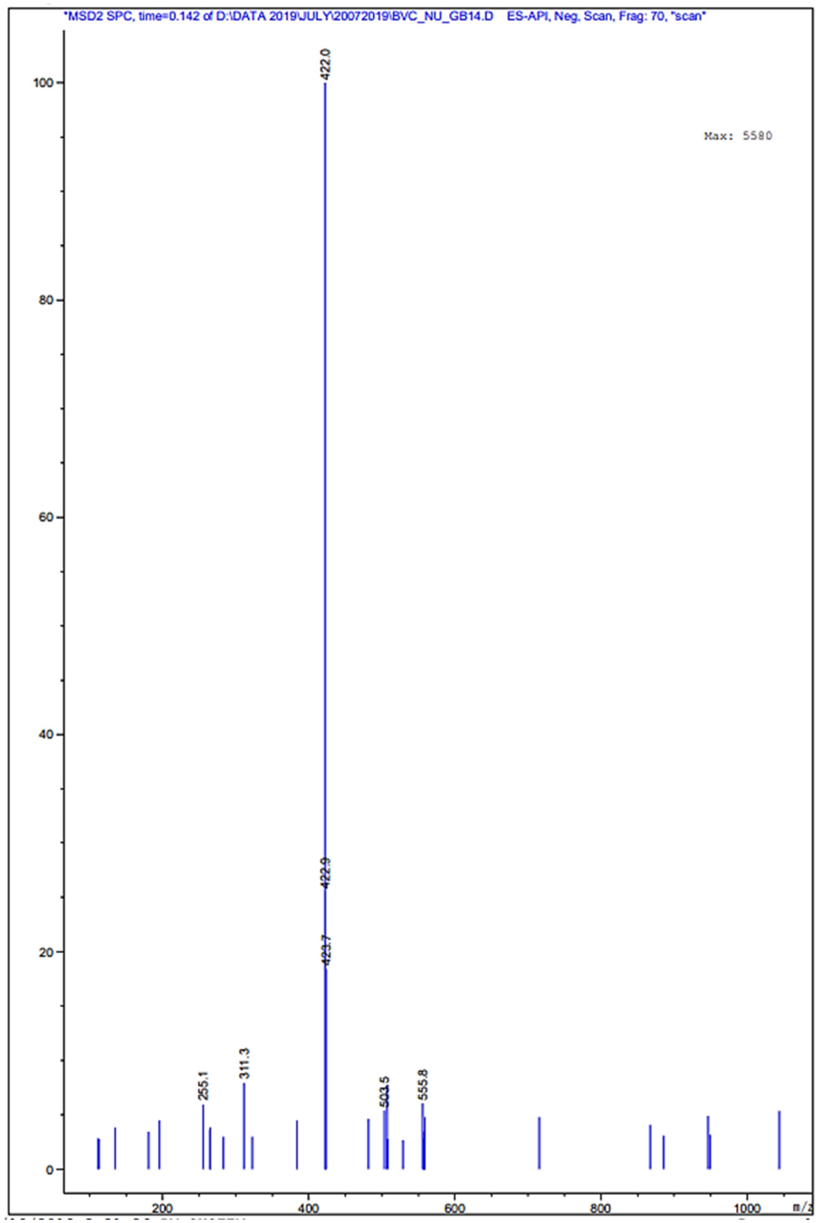
*MSD2 SPC, time=0.142 of D:\DATA 2019\JULY\20072019\BVC_NU_GB14.D ES-API, Neg, Scan, Frag: 70, "scan"
Max: 5580
422.0
422.9
423.7
255.1
311.3
503.5
555.8
100
80
60
40
20
0
200
400
600
800
1000
m/z

6. UV

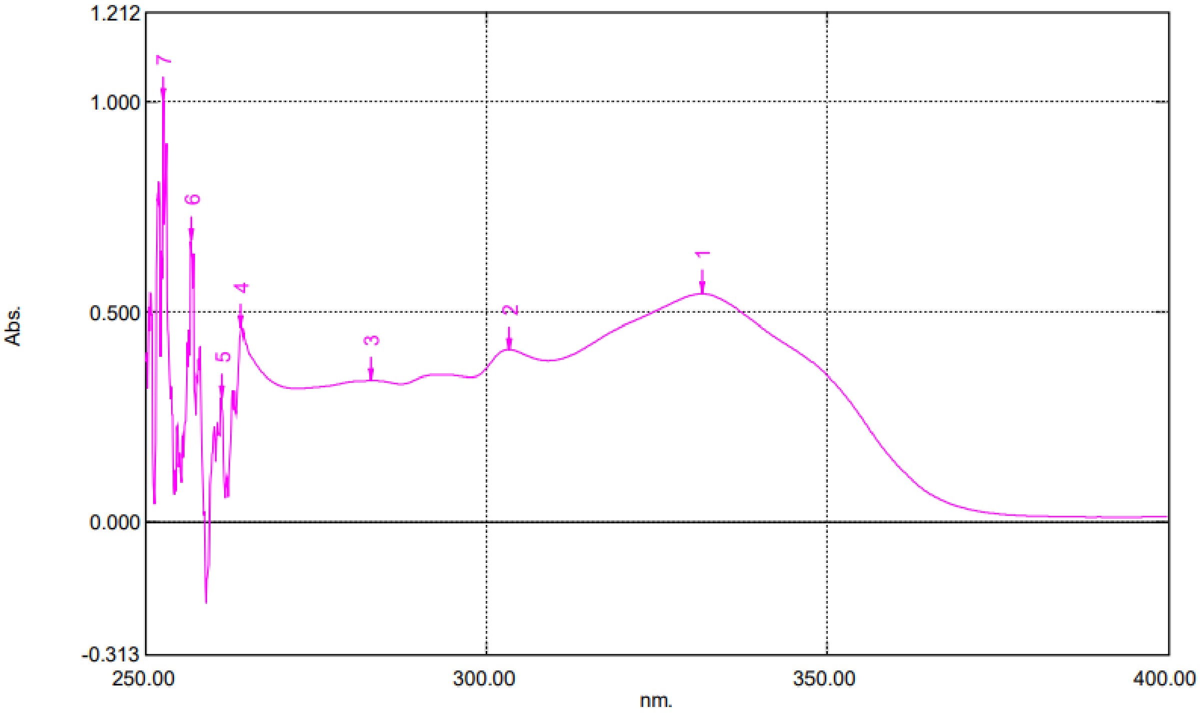

1.212
1.000
0.500
0.000
-0.313
Abs.
250.00
300.00
350.00
400.00
nm.
1
2
3
4
5
6
7

2-(5-(3,4-dimethylbenzylidene)-2,4-dioxothiazolidin-3-yl)-N-(6-methylbenzo[d]thiazol-2-yl)acetamide (GB15)

1. FTIR –

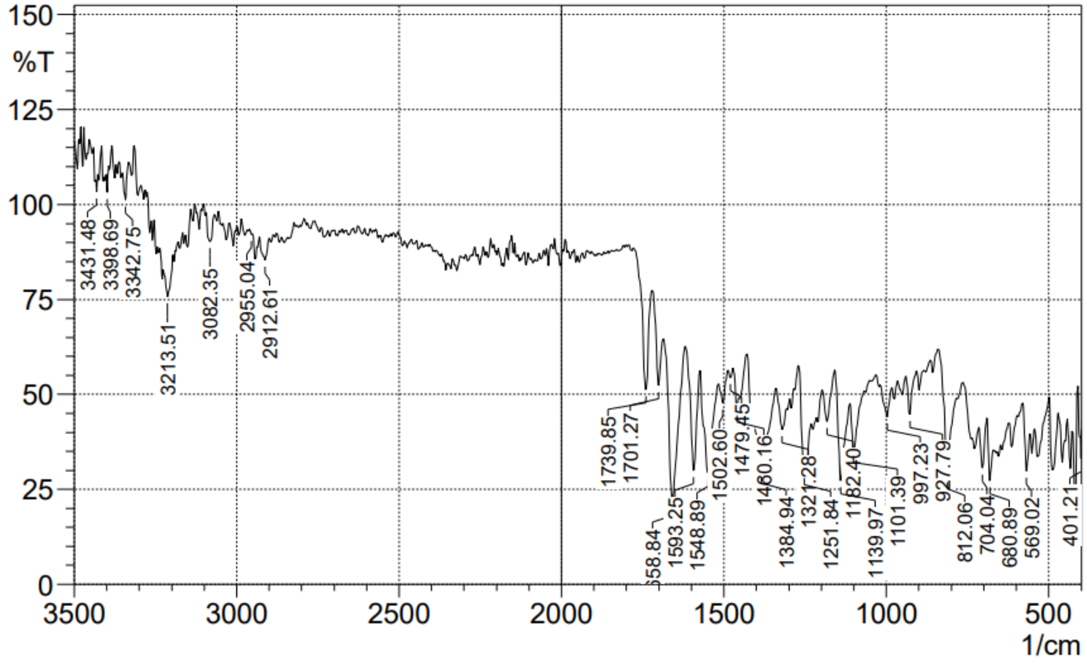

2. ^{1}H-NMR

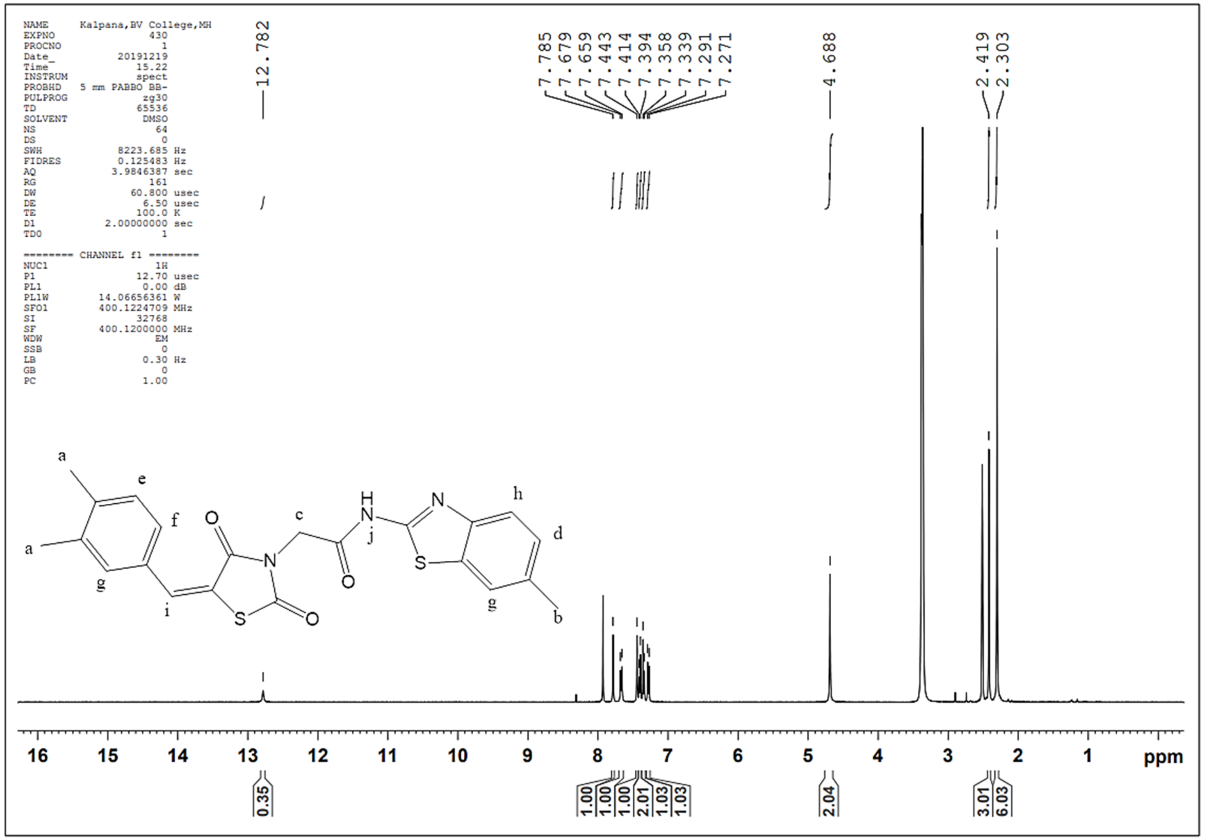
NAME Kalpana,BV College,MH
EXPNO 430
PROCNO 1
Date_ 20191219
Time 15.22
INSTRUM spect
PROBHD 5 mm PABBO BB-
PULPROG zg30
TD 65536
SOLVENT DMSO
NS 64
DS 0
SWH 8223.685 Hz
FIDRES 0.125483 Hz
AQ 3.9846387 sec
RG 161
DW 60.800 usec
DE 6.50 usec
TE 100.0 K
D1 2.00000000 sec
TD0 1
======== CHANNEL f1 ========
NUC1 1H
P1 12.70 usec
PL1 0.00 dB
PL1W 14.06656361 W
SFO1 400.1224709 MHz
SI 32768
SF 400.1200000 MHz
WDW EM
SSB 0
LB 0.30 Hz
GB 0
PC 1.00
12.782
7.785
7.679
7.659
7.443
7.414
7.394
7.358
7.339
7.291
7.271
4.688
2.419
2.303
ppm
0.35
1.00
1.00
1.00
2.01
1.03
1.03
2.04
3.01
6.03

3. ^{13}C-NMR

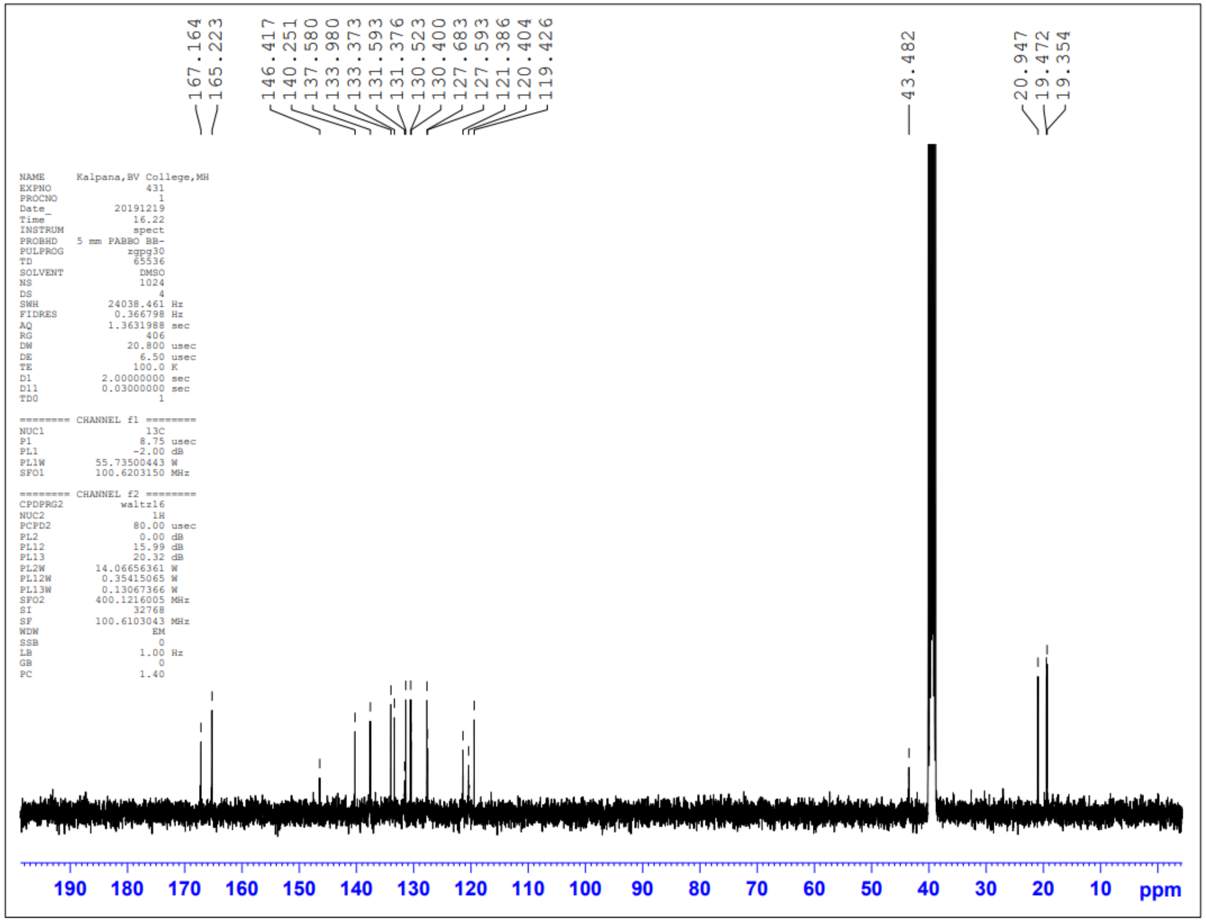

4. HPLC Analysis

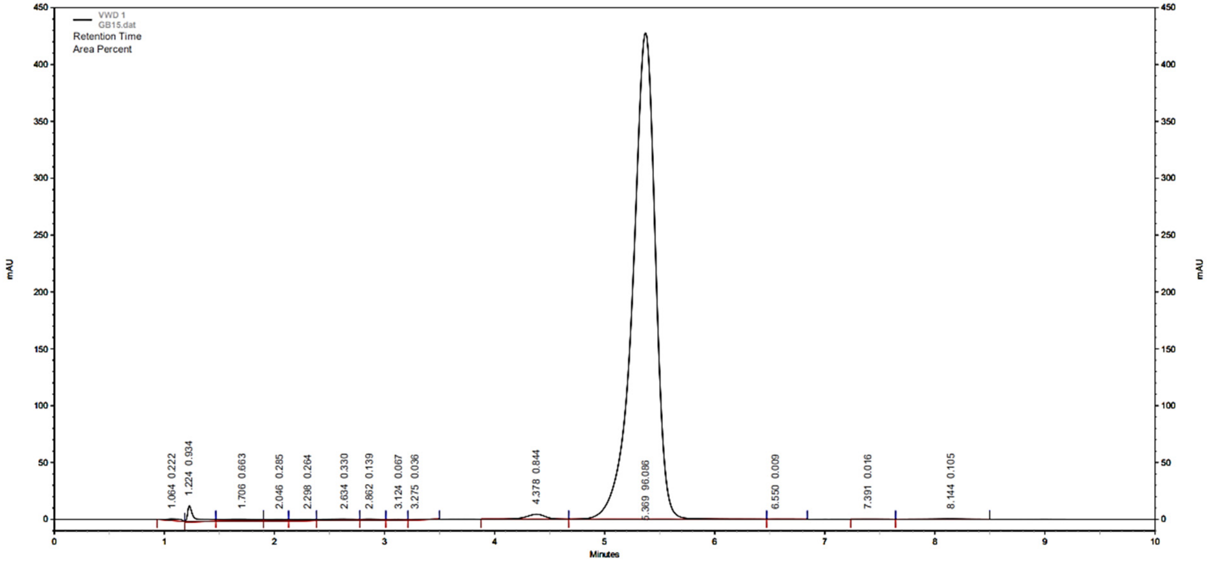

5. Mass Spectrometry

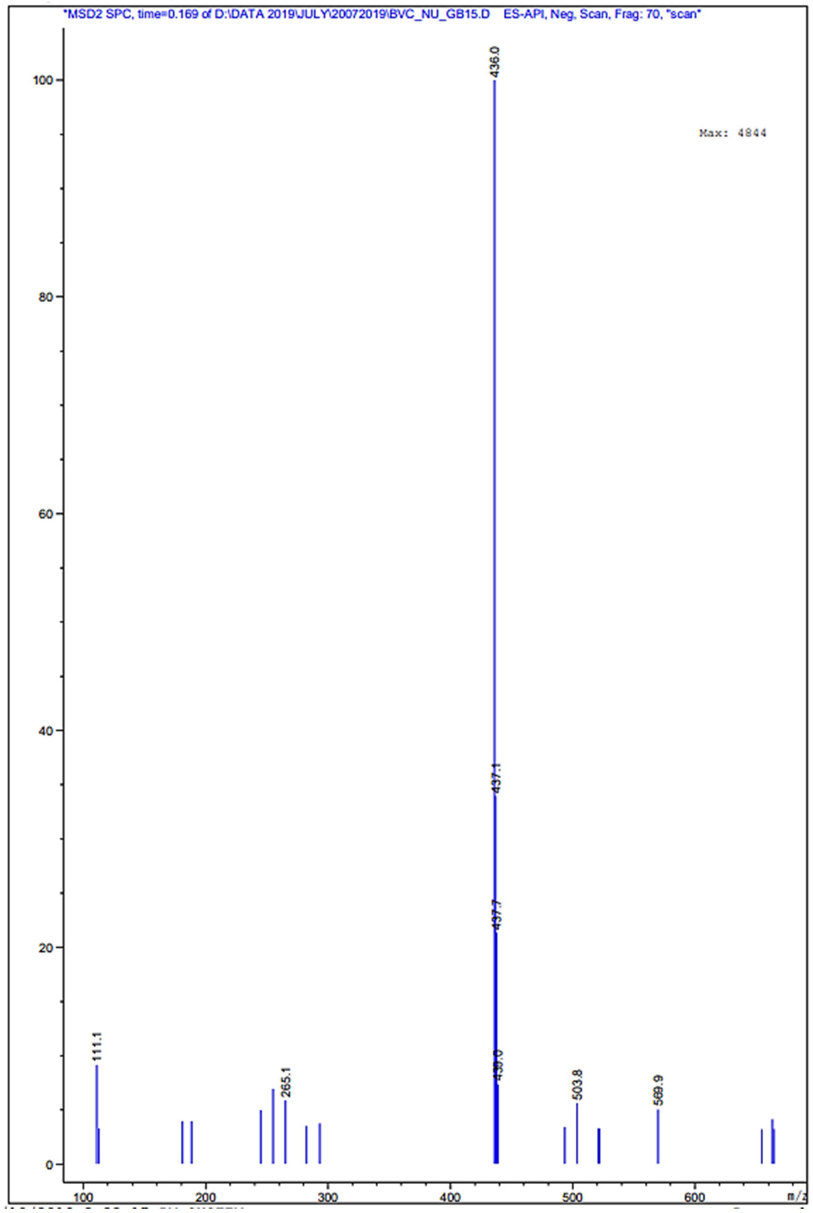
*MSD2 SPC, time=0.169 of D:\DATA 2019\JULY\20072019\BVC_NU_GB15.D   ES-API, Neg, Scan, Frag: 70, "scan"
Max: 4844
436.0
437.1
437.7
111.1
265.1
503.8
569.9
m/z

6. UV

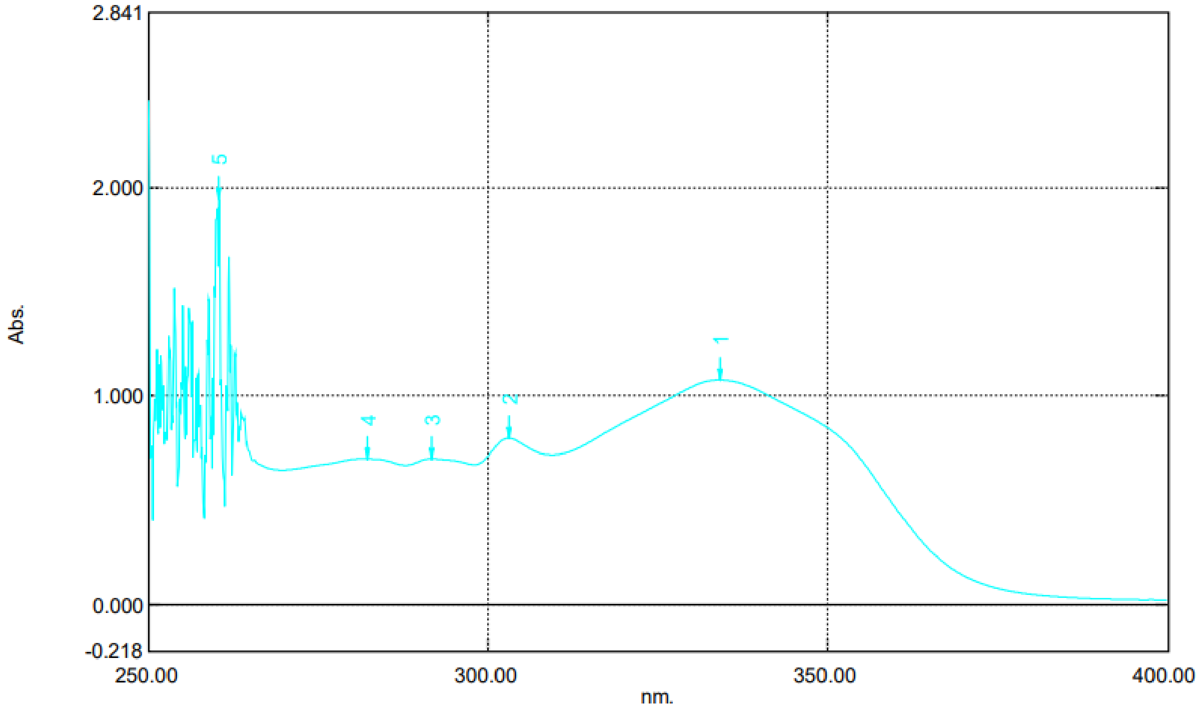
2.841
2.000
1.000
0.000
-0.218
Abs.
250.00
300.00
350.00
400.00
nm.
1
2
3
4
5

2-(5-(4-chlorobenzylidene)-2,4-dioxothiazolidin-3-yl)-N-(6-methylbenzo[d]thiazol-2-yl)acetamide (GB16)

1. FTIR –

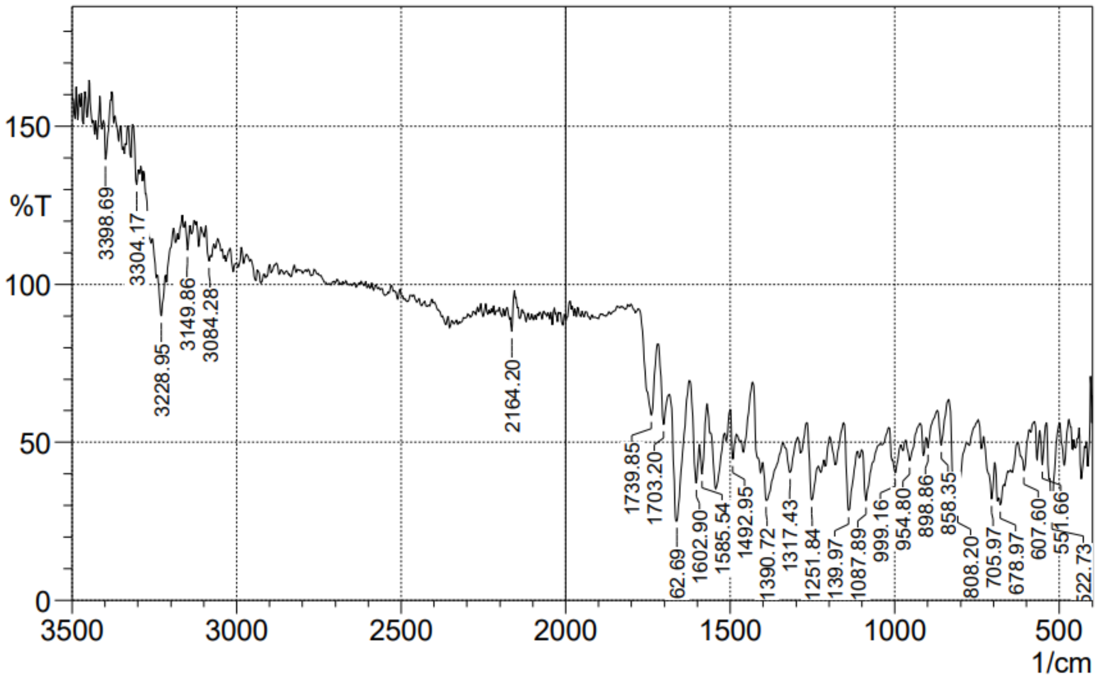

2. ¹H-NMR

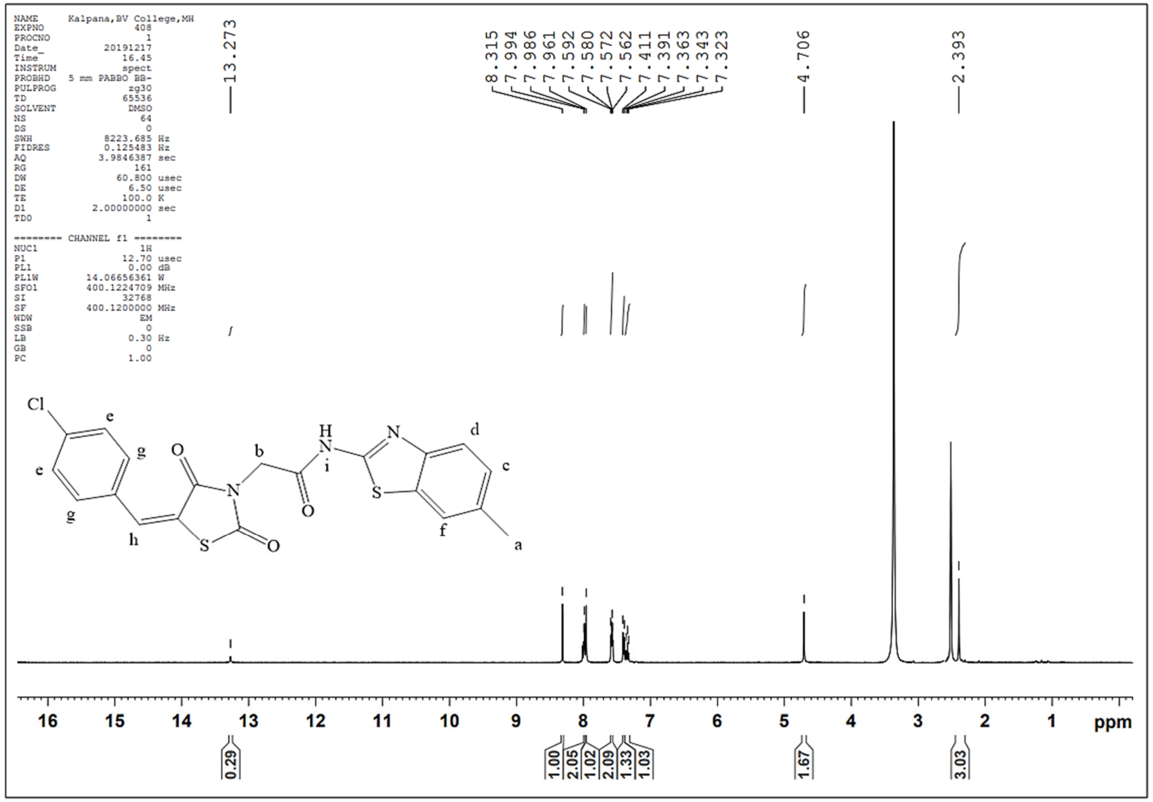
NAME Kalpana,BV College,MH
EXPNO 408
PROCNO 1
Date_ 20191217
Time 16.45
INSTRUM spect
PROBHD 5 mm PABBO BB-
PULPROG zg30
TD 65536
SOLVENT DMSO
NS 64
DS 0
SWH 8223.685 Hz
FIDRES 0.125483 Hz
AQ 3.9846387 sec
RG 161
DW 60.800 usec
DE 6.50 usec
TE 100.0 K
D1 2.00000000 sec
TD0 1
======== CHANNEL f1 ========
NUC1 1H
P1 12.70 usec
PL1 0.00 dB
PL1W 14.06656361 W
SFO1 400.1224709 MHz
SI 32768
SF 400.1200000 MHz
WDW EM
SSB 0
LB 0.30 Hz
GB 0
PC 1.00
13.273
8.315
7.994
7.986
7.961
7.592
7.580
7.572
7.562
7.411
7.391
7.363
7.343
7.323
4.706
2.393
Cl
e
g
O
b
H
N
i
N
d
c
e
g
h
S
O
N
O
S
f
a
16
15
14
13
12
11
10
9
8
7
6
5
4
3
2
1
ppm
0.29
1.00
2.05
1.02
2.09
1.33
1.03
1.67
3.03

3. ^{13}C-NMR

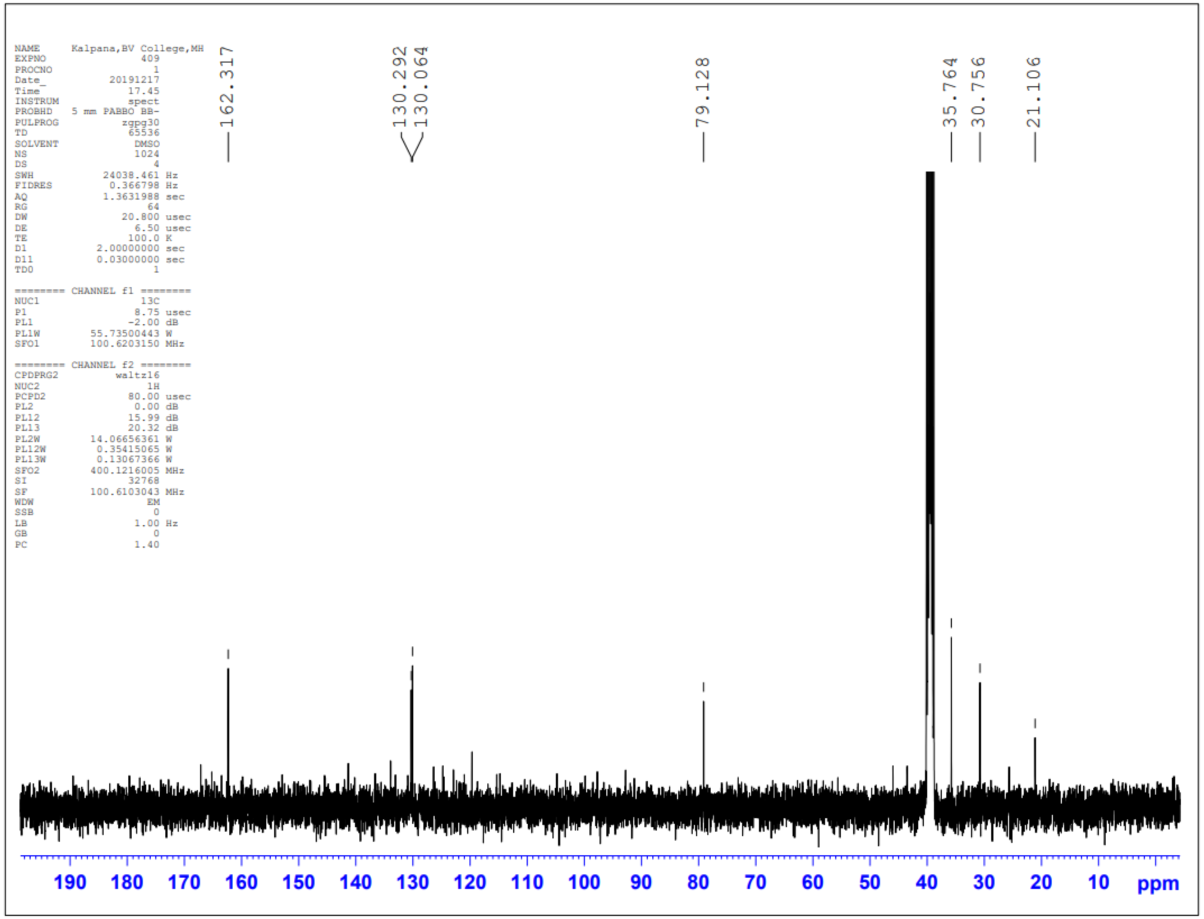

4. HPLC Analysis

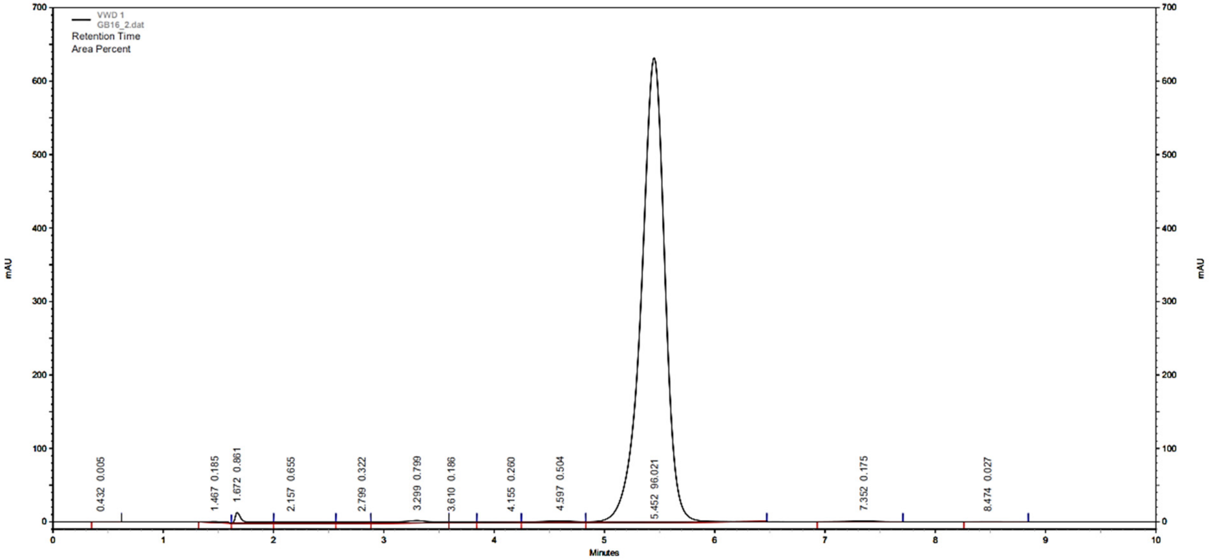

5. Mass Spectrometry

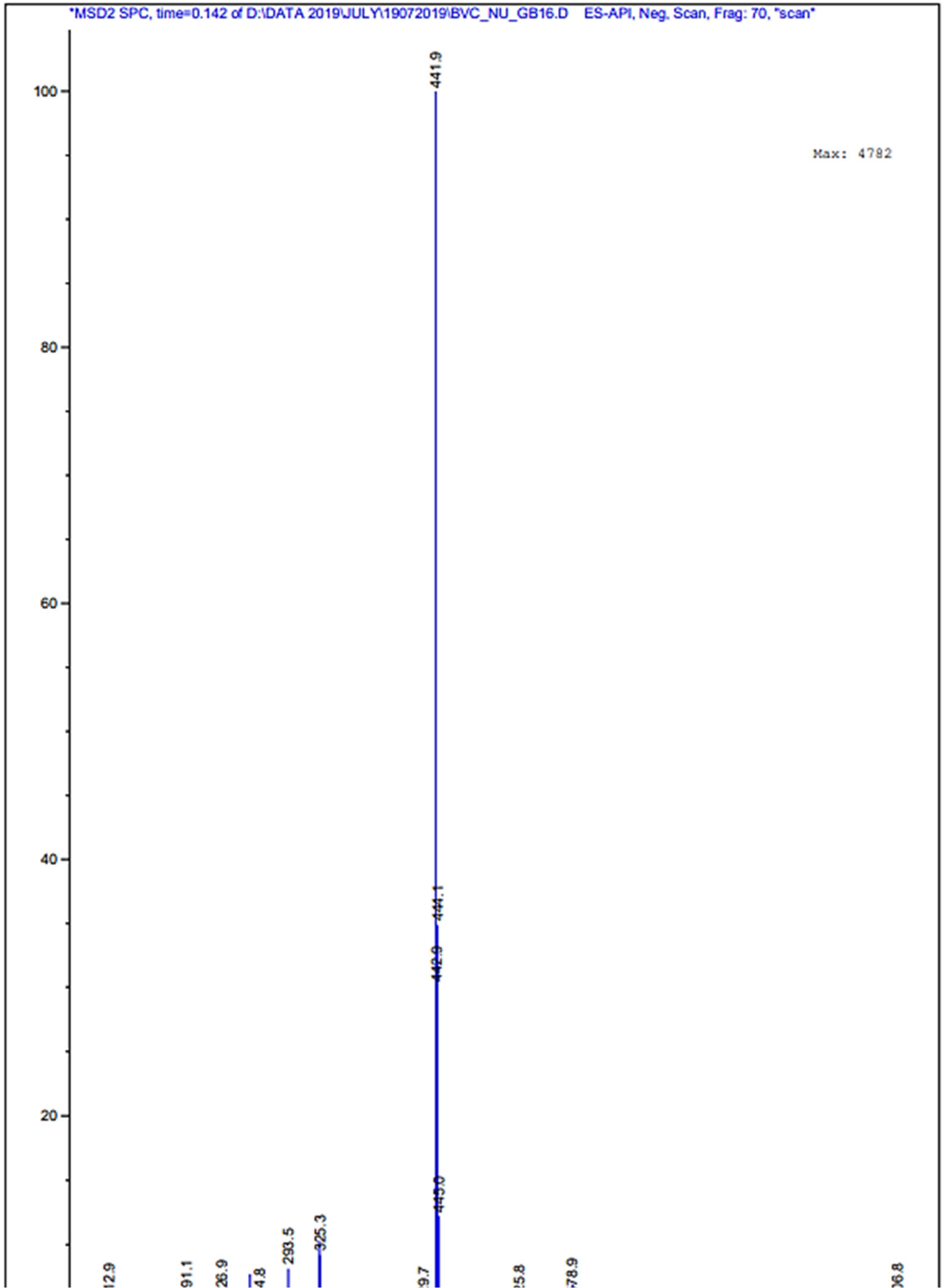
*MSD2 SPC, time=0.142 of D:\DATA 2019\JULY\19072019\BVC_NU_GB16.D ES-API, Neg, Scan, Frag: 70, "scan"
Max: 4782
100
80
60
40
20
441.9
293.5
325.3

6. UV

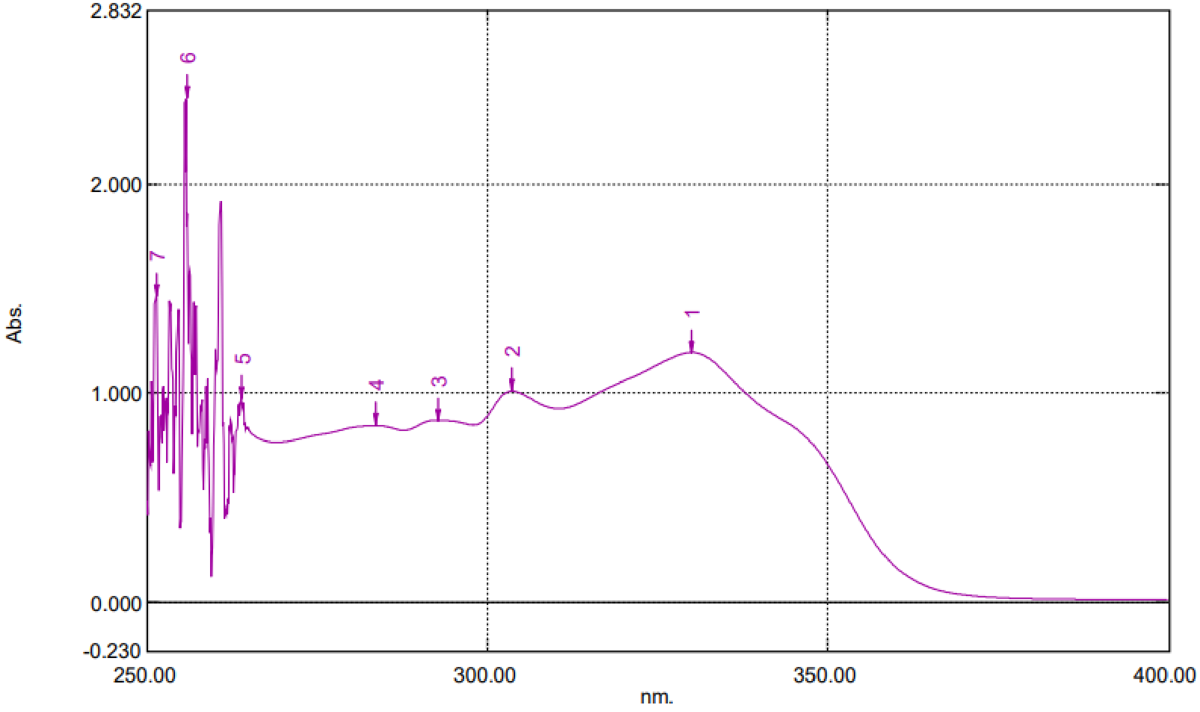

2.832
2.000
1.000
0.000
-0.230
Abs.
250.00
300.00
350.00
400.00
nm.
1
2
3
4
5
6
7

2-(5-(4-chlorobenzylidene)-2,4-dioxothiazolidin-3-yl)-N-(6-ethoxybenzo[d]thiazol-2-yl)acetamide (GB17)

1. FTIR –

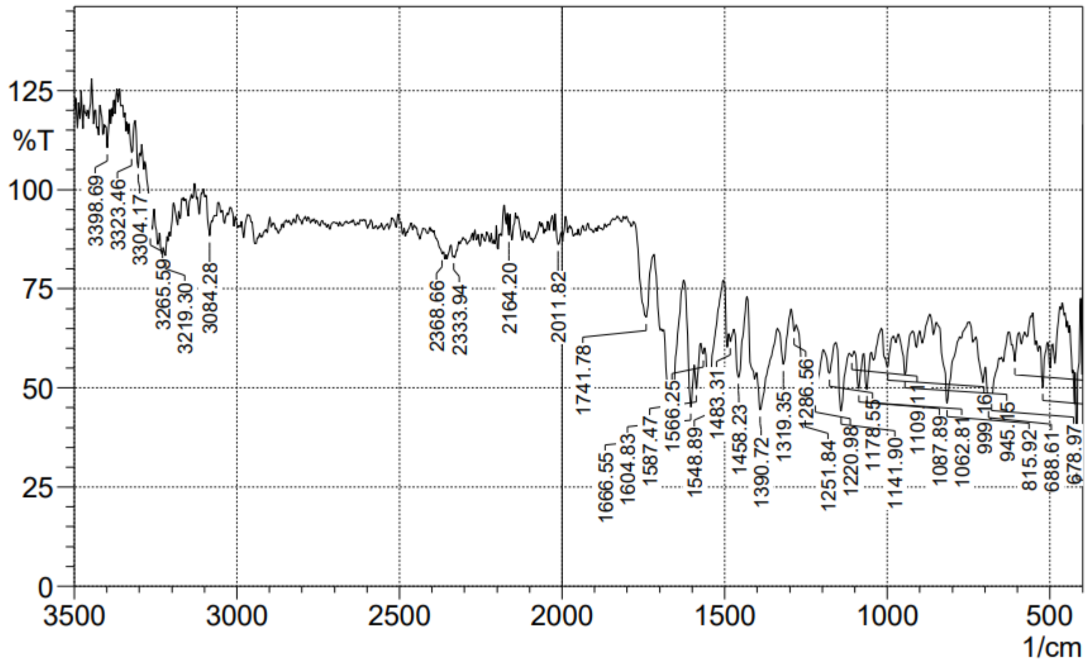

2. ^{1}H-NMR

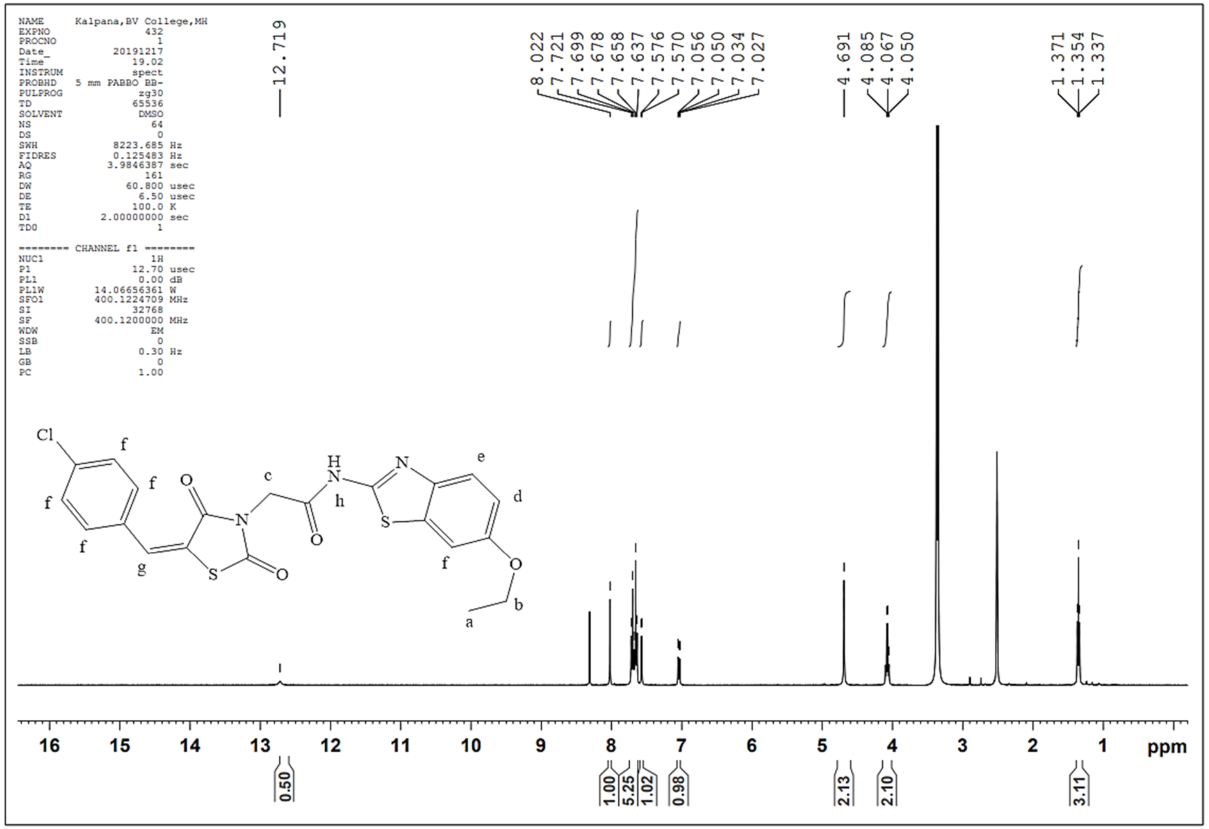
NAME Kalpana,BV College,MH
EXPNO 432
PROCNO 1
Date_ 20191217
Time 19.02
INSTRUM spect
PROBHD 5 mm PABBO BB-
PULPROG zg30
TD 65536
SOLVENT DMSO
NS 64
DS 0
SWH 8223.685 Hz
FIDRES 0.125483 Hz
AQ 3.9846387 sec
RG 161
DW 60.800 usec
DE 6.50 usec
TE 100.0 K
D1 2.00000000 sec
TD0 1
======== CHANNEL f1 ========
NUC1 1H
P1 12.70 usec
PL1 0.00 dB
PL1W 14.06656361 W
SFO1 400.1224709 MHz
SI 32768
SF 400.1200000 MHz
WDW EM
SSB 0
LB 0.30 Hz
GB 0
PC 1.00
12.719
8.022
7.721
7.699
7.678
7.658
7.637
7.576
7.570
7.056
7.050
7.034
7.027
4.691
4.085
4.067
4.050
1.371
1.354
1.337
Cl
f
f
f
f
g
S
O
O
N
c
O
H
N
h
S
N
e
d
f
O
b
a
16 15 14 13 12 11 10 9 8 7 6 5 4 3 2 1 ppm
0.50
1.00
5.25
1.02
0.98
2.13
2.10
3.11

3. ^{13}C-NMR

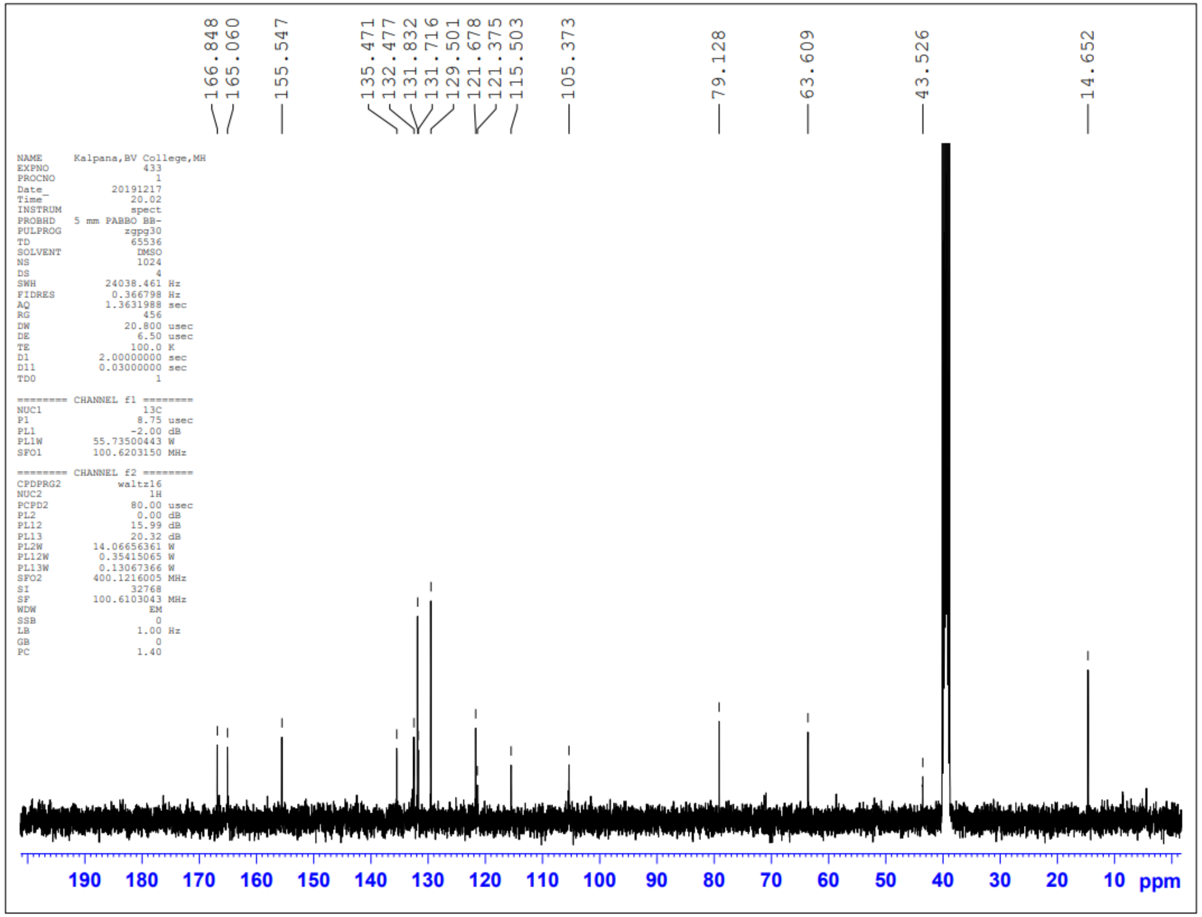

4. HPLC Analysis

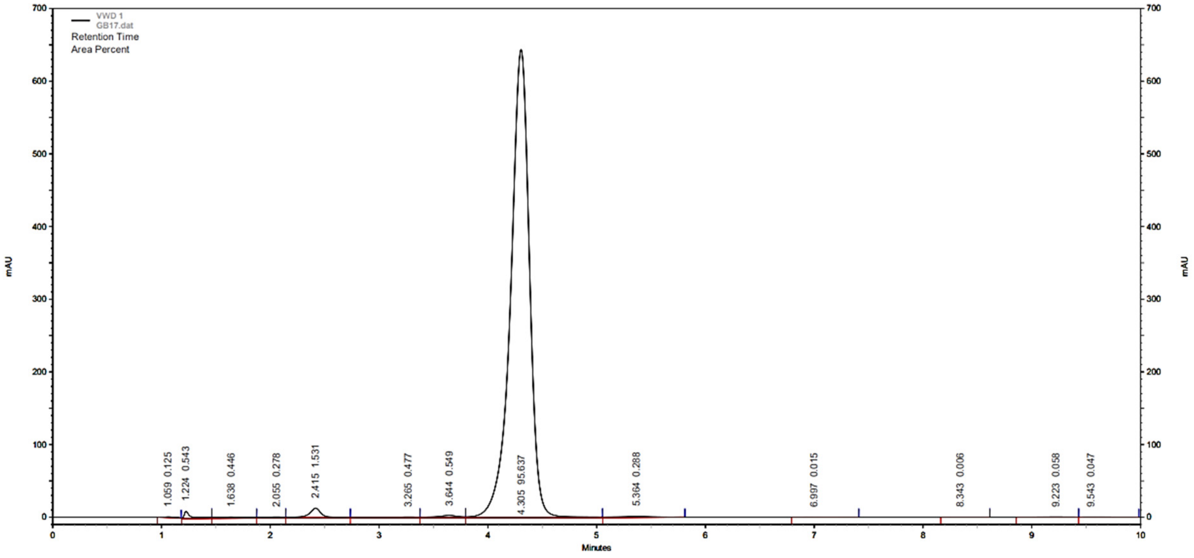

5. Mass Spectrometry

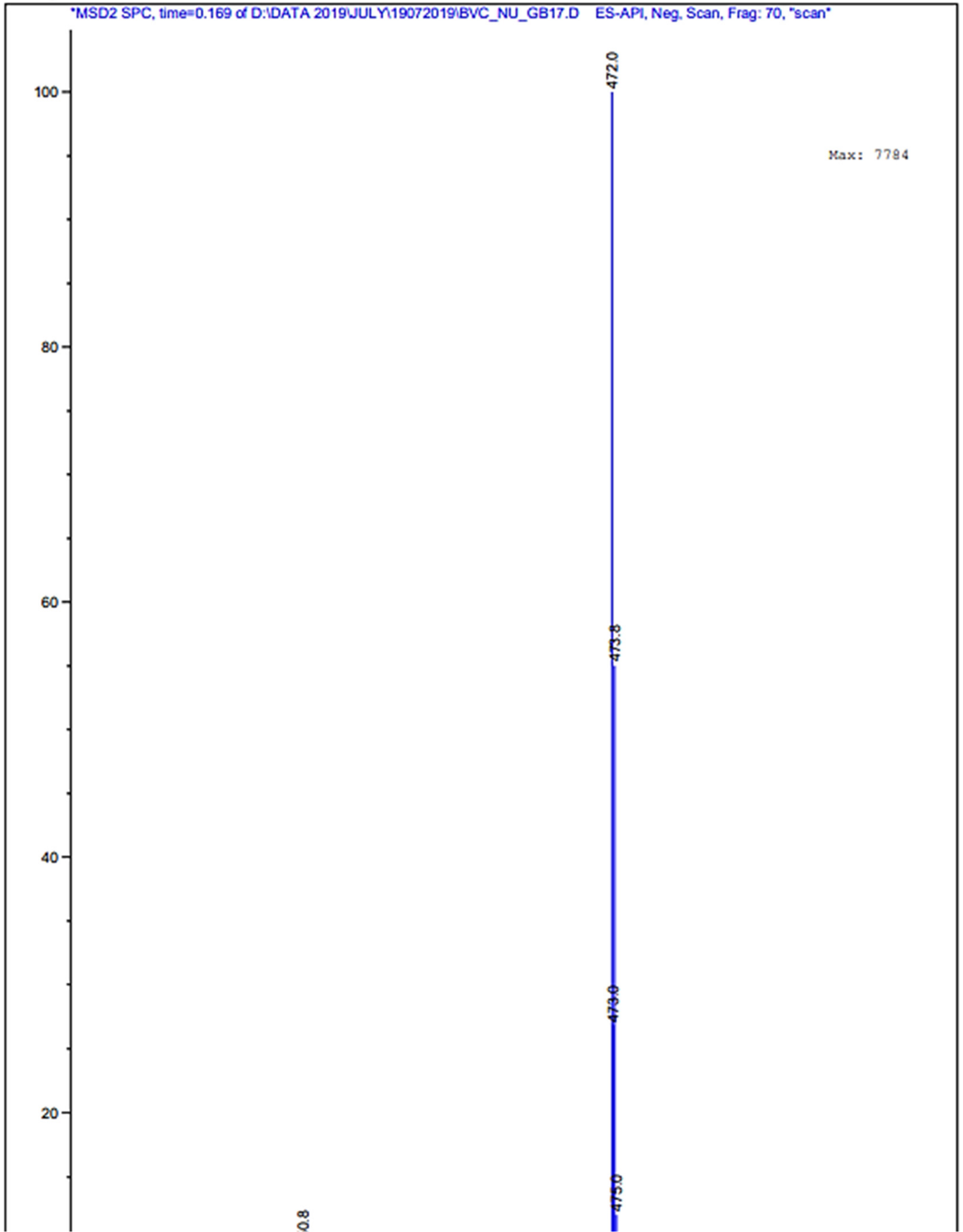
*MSD2 SPC, time=0.169 of D:\DATA 2019\JULY\19072019\BVC_NU_GB17.D ES-API, Neg, Scan, Frag: 70, "scan"
Max: 7784
100
80
60
40
20
472.0
473.8
473.0
475.0

6. UV

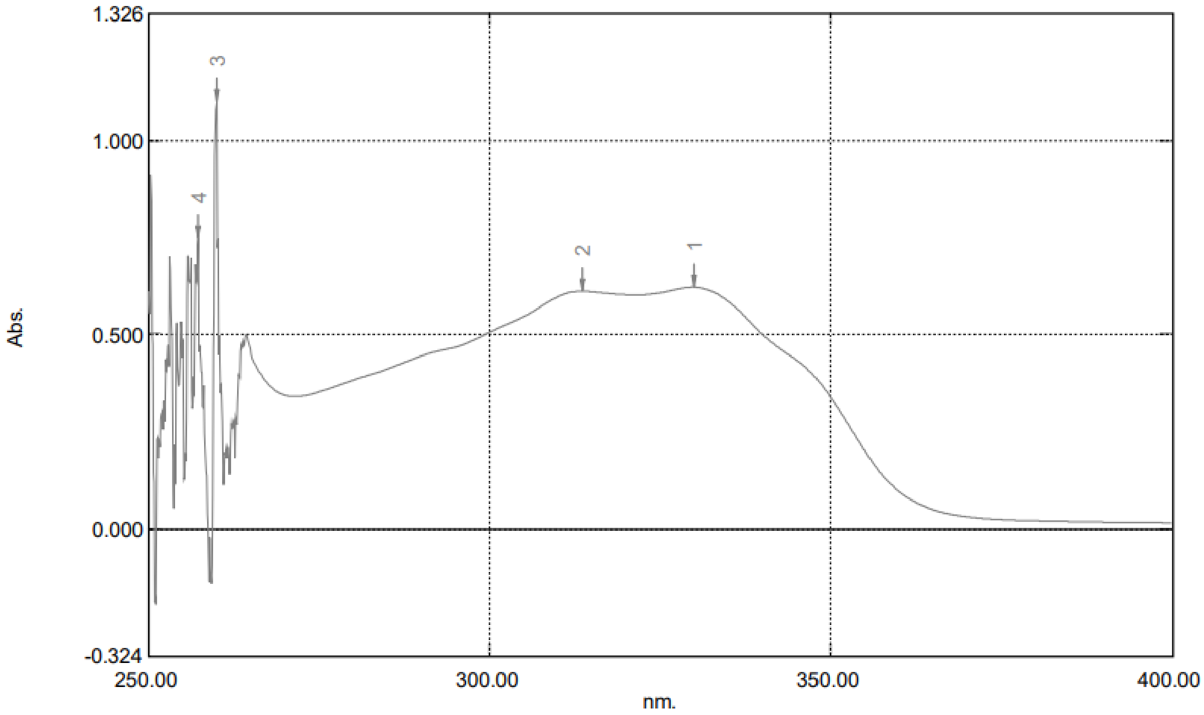

N-(4,6-difluorobenzo[d]thiazol-2-yl)-2-(5-(4-fluorobenzylidene)-2,4-dioxothiazolidin-3-yl)acetamide (GB18)

1. FTIR

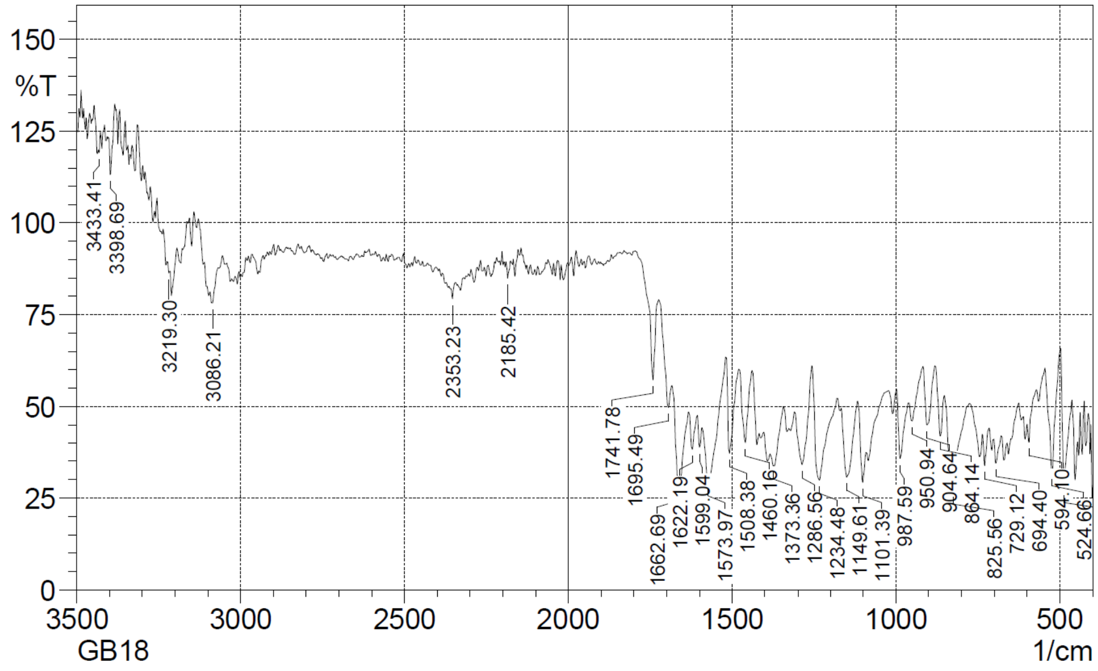

2. 1H-NMR

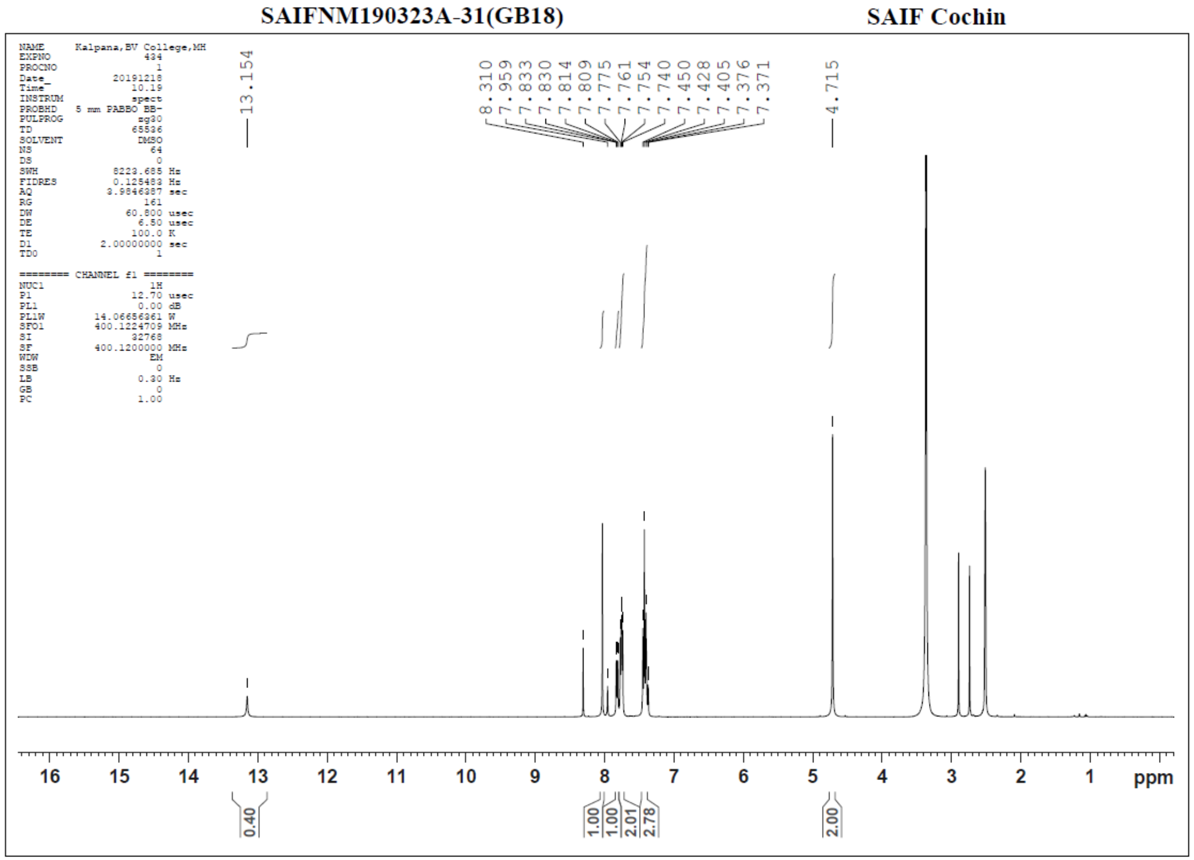
SAIFNM190323A-31(GB18)
SAIF Cochin
NAME Kalpana,BV College,MH
EXPNO 434
PROCNO 1
Date_ 20191218
Time 10.19
INSTRUM spect
PROBHD 5 mm PABBO BB-
PULPROG zg30
TD 65536
SOLVENT DMSO
NS 64
DS 0
SWH 8223.685 Hz
FIDRES 0.125483 Hz
AQ 3.9846387 sec
RG 161
DW 60.800 usec
DE 6.50 usec
TE 100.0 K
D1 2.00000000 sec
TD0 1
======== CHANNEL f1 ========
NUC1 1H
P1 12.70 usec
PL1 0.00 dB
PL1W 14.06656361 W
SFO1 400.1224709 MHz
SI 32768
SF 400.1200000 MHz
WDW EM
SSB 0
LB 0.30 Hz
GB 0
PC 1.00
13.154
8.310
7.959
7.833
7.830
7.814
7.809
7.775
7.761
7.754
7.740
7.450
7.428
7.405
7.376
7.371
4.715
16 15 14 13 12 11 10 9 8 7 6 5 4 3 2 1 ppm
0.40
1.00
1.00
2.01
2.78
2.00

3. 13C-NMR

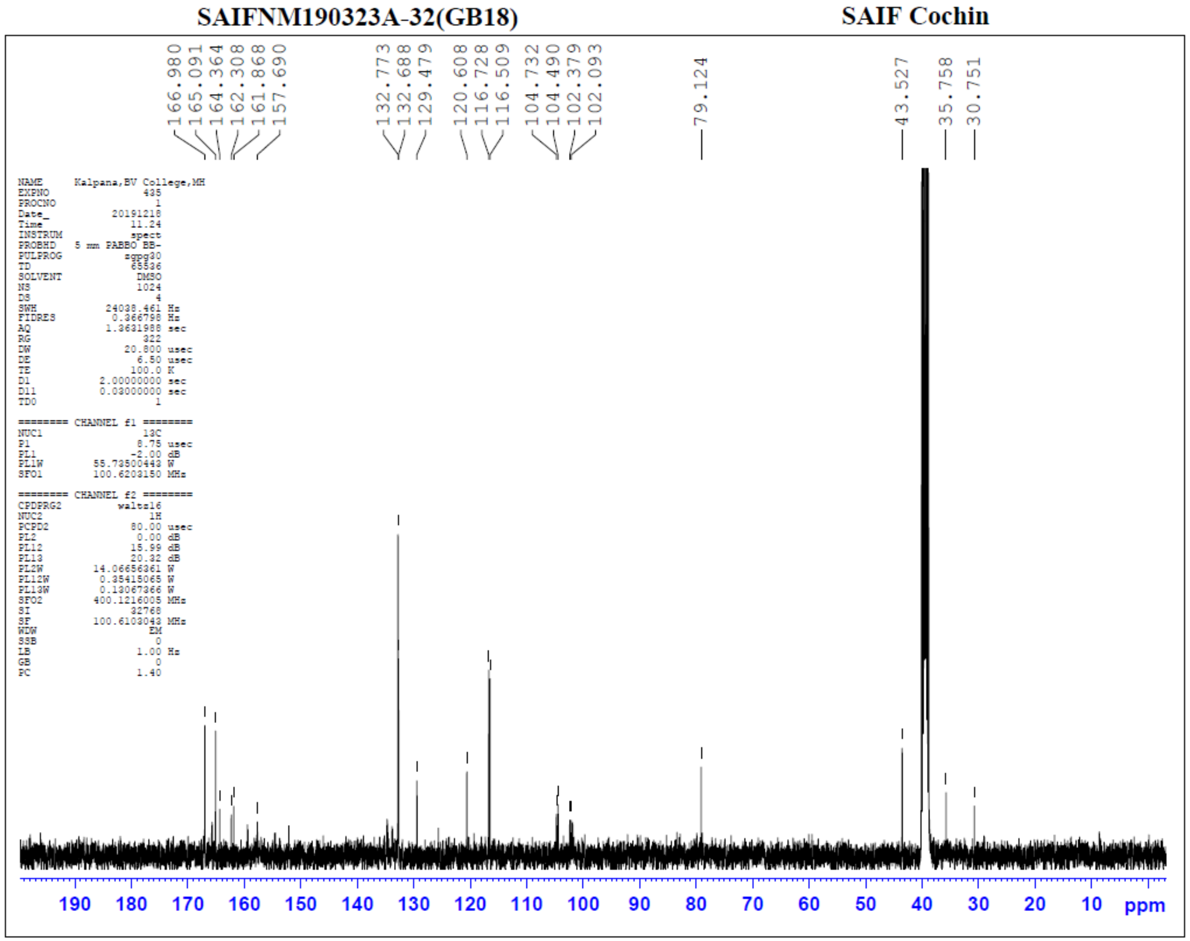
SAIFNM190323A-32(GB18)
SAIF Cochin
166.980
165.091
164.364
162.308
161.868
157.690
132.773
132.688
129.479
120.608
116.728
116.509
104.732
104.490
102.379
102.093
79.124
43.527
35.758
30.751
NAME Kalpana,BV College,MH
EXPNO 435
PROCNO 1
Date_ 20191218
Time 11.24
INSTRUM spect
PROBHD 5 mm PABBO BB-
PULPROG zgpg30
TD 65536
SOLVENT DMSO
NS 1024
DS 4
SWH 24038.461 Hz
FIDRES 0.366798 Hz
AQ 1.3631988 sec
RG 322
DW 20.800 usec
DE 6.50 usec
TE 100.0 K
D1 2.00000000 sec
D11 0.03000000 sec
TD0 1
======== CHANNEL f1 ========
NUC1 13C
P1 8.75 usec
PL1 -2.00 dB
PL1W 55.73500443 W
SFO1 100.6203150 MHz
======== CHANNEL f2 ========
CPDPRG2 waltz16
NUC2 1H
PCPD2 80.00 usec
PL2 0.00 dB
PL12 15.99 dB
PL13 20.32 dB
PL2W 14.06656361 W
PL12W 0.35415065 W
PL13W 0.13067366 W
SFO2 400.1216005 MHz
SI 32768
SF 100.6103043 MHz
WDW EM
SSB 0
LB 1.00 Hz
GB 0
PC 1.40
190 180 170 160 150 140 130 120 110 100 90 80 70 60 50 40 30 20 10 ppm

4. HPLC

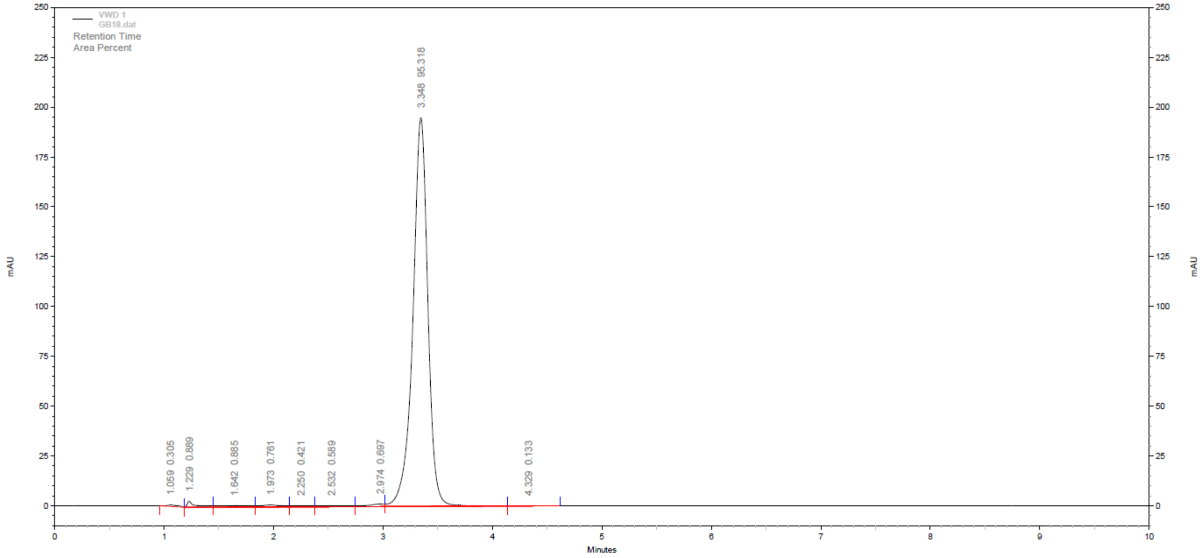
VWD 1
GB18.dat
Retention Time
Area Percent
1.059 0.305
1.229 0.889
1.642 0.885
1.973 0.761
2.250 0.421
2.532 0.589
2.974 0.697
3.348 95.318
4.329 0.133
mAU
Minutes

\

5. Mass

MS Spectrum

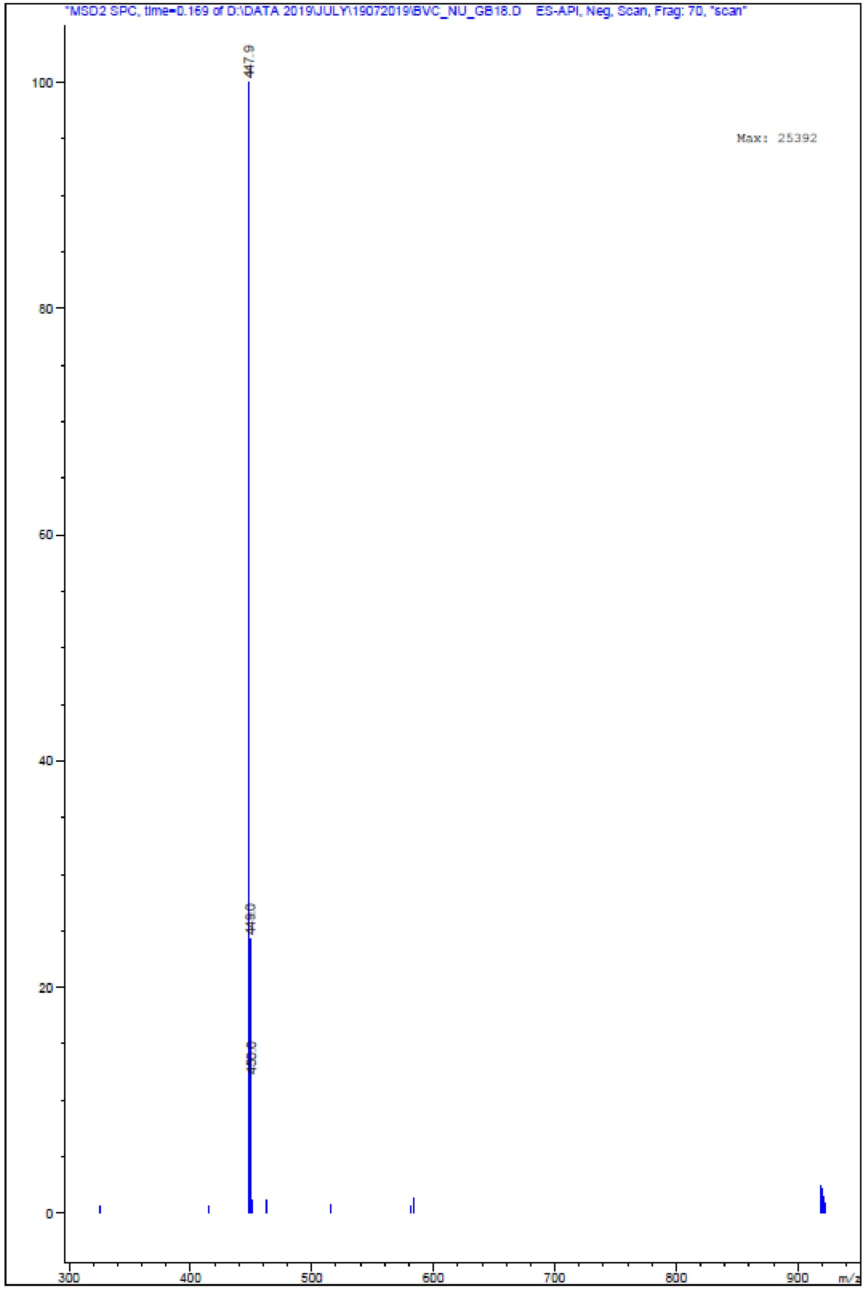
"MSD2 SPC, time=0.169 of D:\DATA 2019\JULY\19072019\BVC_NU_GB18.D   ES-API, Neg, Scan, Frag: 70, "scan"
447.9
Max: 25392
100
80
60
40
20
0
300
400
500
600
700
800
900
m/z

N-(4,6-difluorobenzo[d]thiazol-2-yl)-2-(5-(4-methylbenzylidene)-2,4-dioxothiazolidin-3-yl)acetamide (GB19)

1. FTIR

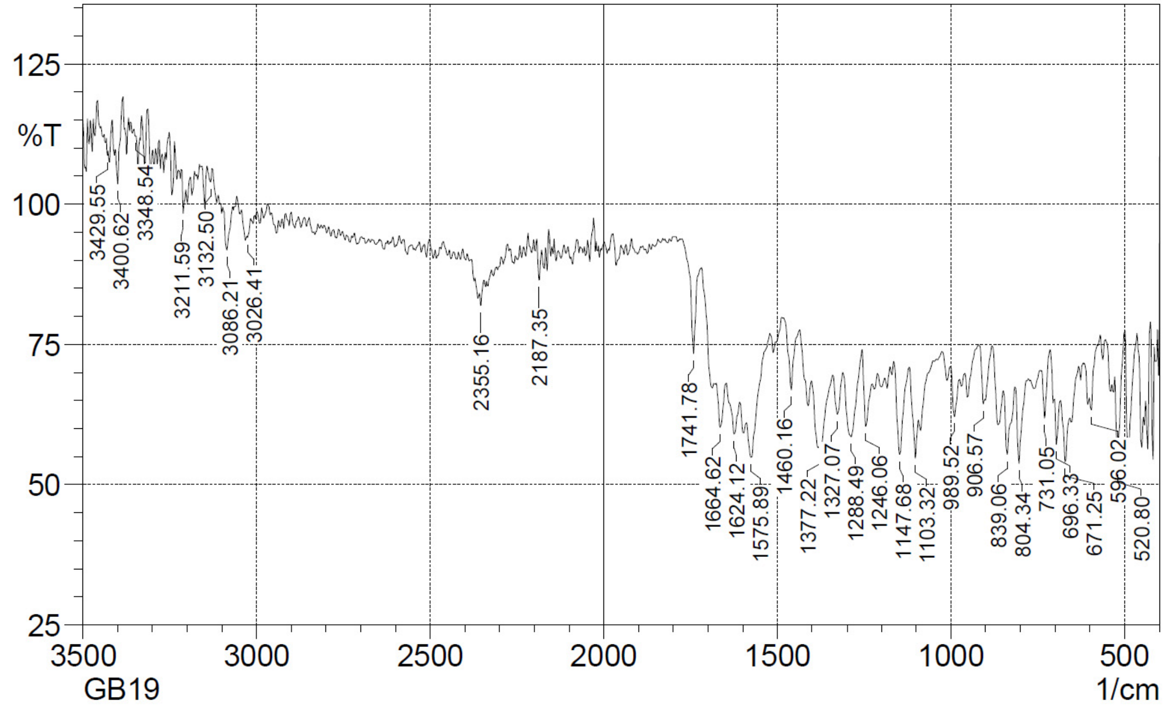

2. 1H-NMR

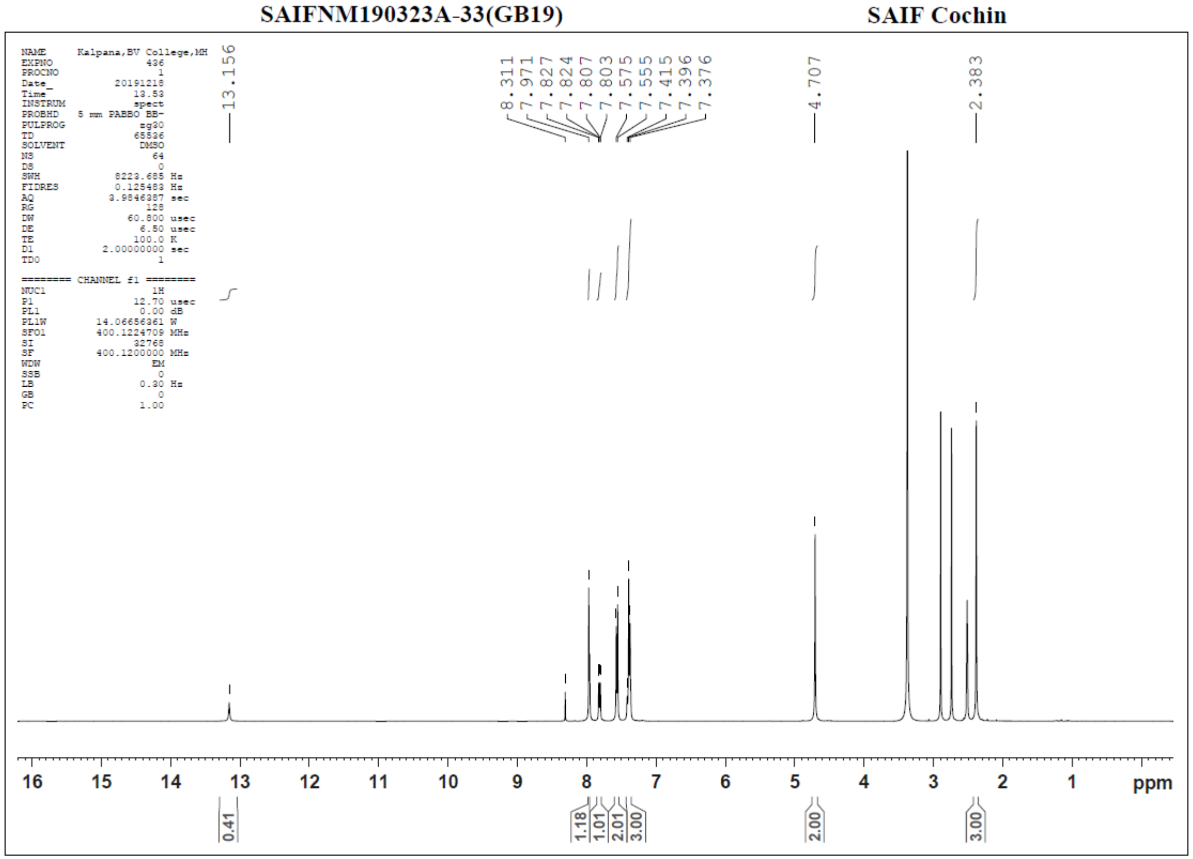
SAIFNM190323A-33(GB19)
SAIF Cochin
13.156
8.311
7.971
7.827
7.824
7.807
7.803
7.575
7.555
7.415
7.396
7.376
4.707
2.383
ppm
0.41
1.18
1.01
2.01
3.00
2.00
3.00

3. 13C-NMR

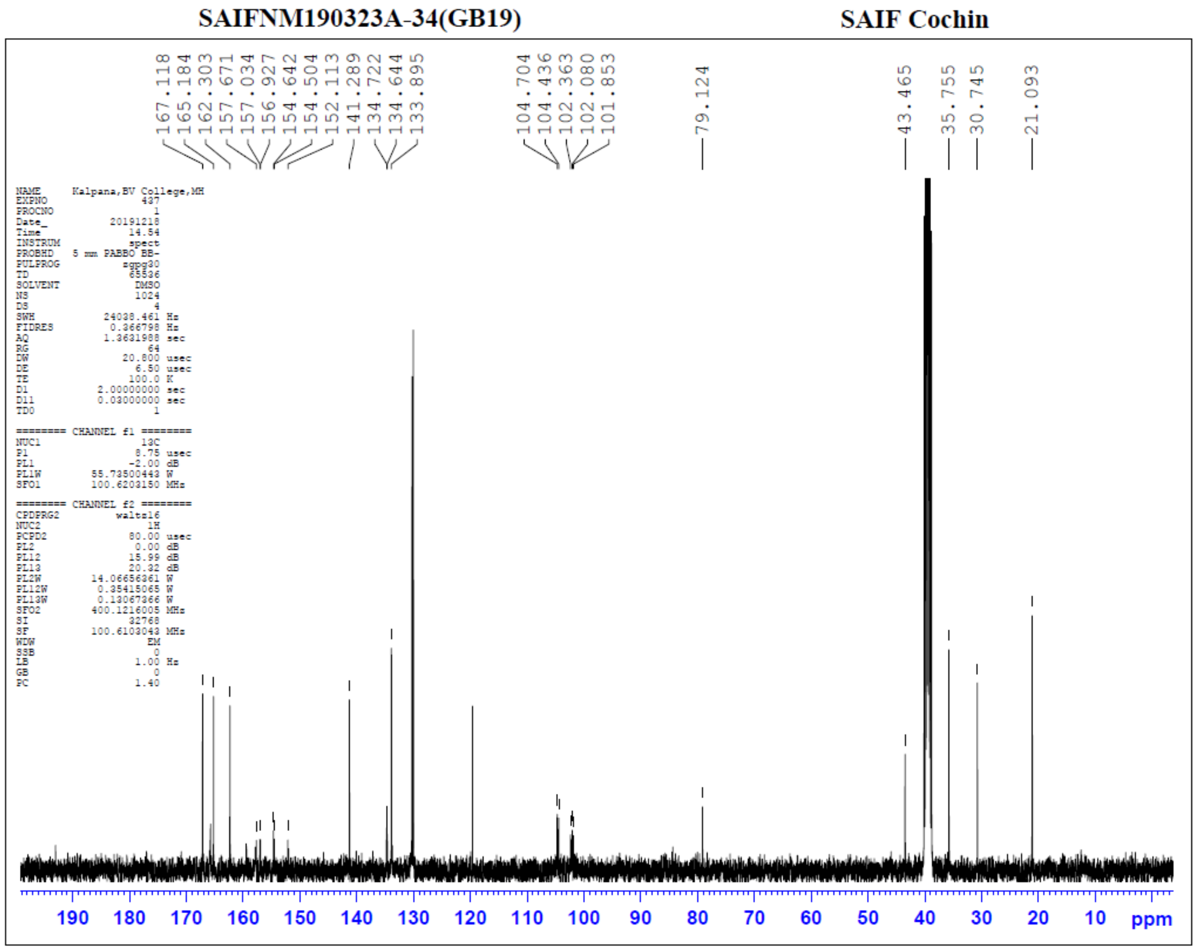
SAIFNM190323A-34(GB19)
SAIF Cochin
167.118
165.184
162.303
157.671
157.034
156.927
154.642
154.504
152.113
141.289
134.722
134.644
133.895
104.704
104.436
102.363
102.080
101.853
79.124
43.465
35.755
30.745
21.093
NAME Kalpana,BV College,MH
EXPNO 437
PROCNO 1
Date_ 20191218
Time 14.54
INSTRUM spect
PROBHD 5 mm PABBO BB-
PULPROG zgpg30
TD 65536
SOLVENT DMSO
NS 1024
DS 4
SWH 24038.461 Hz
FIDRES 0.366798 Hz
AQ 1.3631988 sec
RG 64
DW 20.800 usec
DE 6.50 usec
TE 100.0 K
D1 2.00000000 sec
D11 0.03000000 sec
TD0 1
======== CHANNEL f1 ========
NUC1 13C
P1 8.75 usec
PL1 -2.00 dB
PL1W 55.73500443 W
SFO1 100.6203150 MHz
======== CHANNEL f2 ========
CPDPRG2 waltz16
NUC2 1H
PCPD2 80.00 usec
PL2 0.00 dB
PL12 15.99 dB
PL13 20.32 dB
PL2W 14.06656361 W
PL12W 0.35415065 W
PL13W 0.13067366 W
SFO2 400.1216005 MHz
SI 32768
SF 100.6103043 MHz
WDW EM
SSB 0
LB 1.00 Hz
GB 0
PC 1.40
190 180 170 160 150 140 130 120 110 100 90 80 70 60 50 40 30 20 10 ppm

4. HPLC

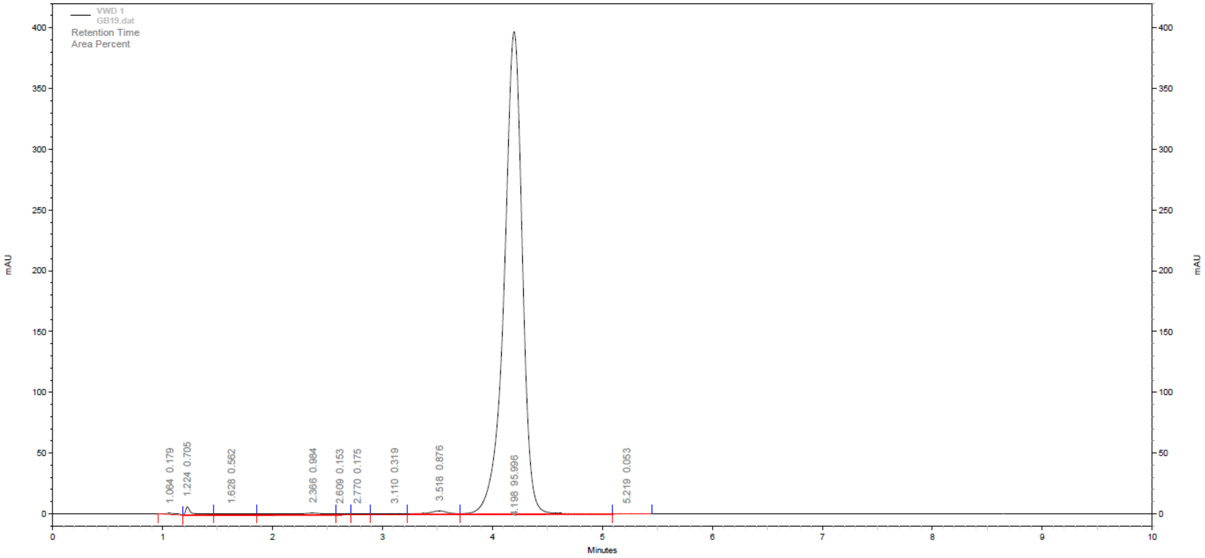
VWD 1
GB19.dat
Retention Time
Area Percent
mAU
Minutes
1.064 0.179
1.224 0.705
1.628 0.562
2.366 0.984
2.609 0.153
2.770 0.175
3.110 0.319
3.518 0.876
4.198 95.996
5.219 0.053

5. Mass

MS Spectrum

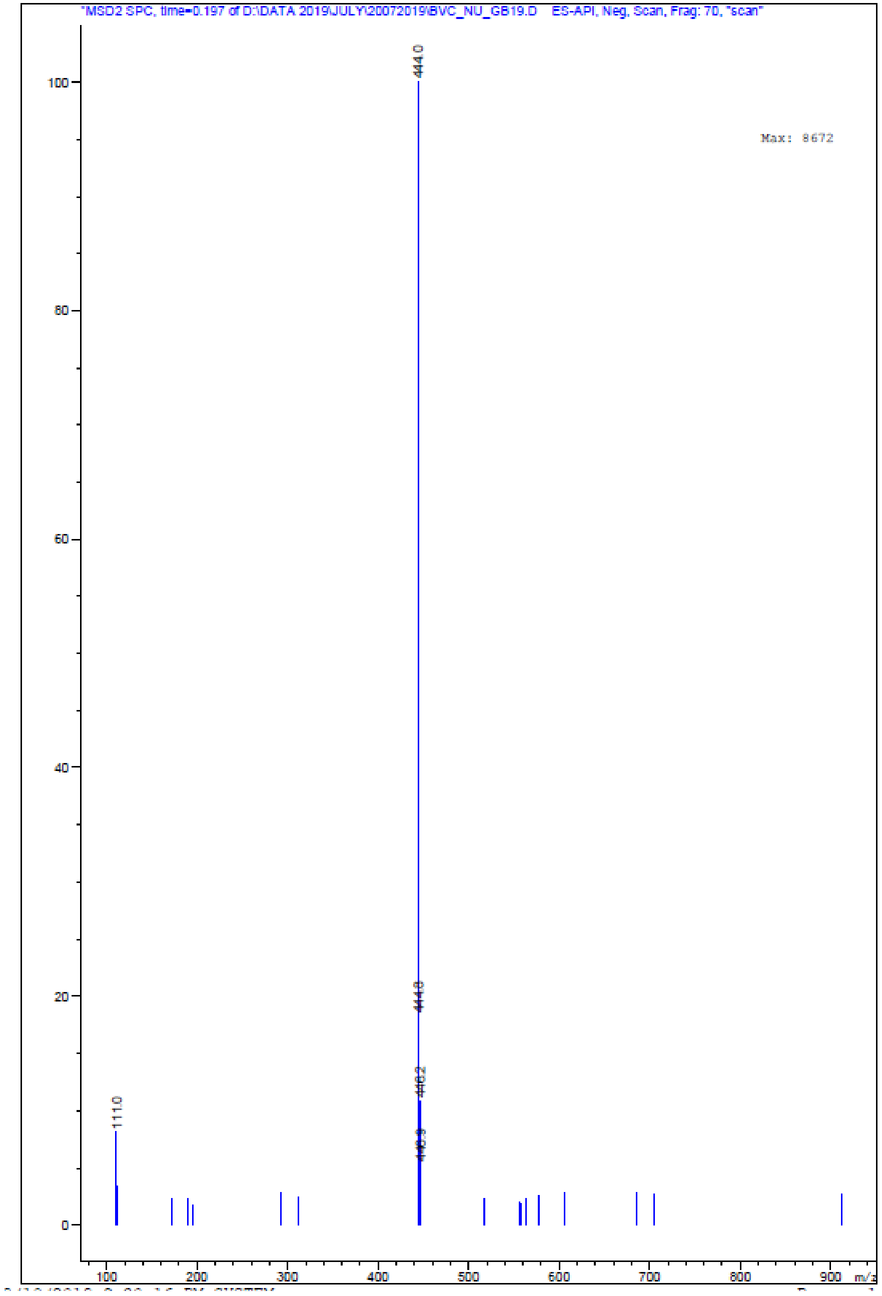

*MSD2 SPC, time=0.197 of D:\DATA 2019\JULY\20072019\BVC_NU_GB19.D   ES-API, Neg, Scan, Frag: 70, "scan"
Max: 8672
444.0
111.0
100
80
60
40
20
0
100
200
300
400
500
600
700
800
900
m/z

N-(4,6-difluorobenzo[d]thiazol-2-yl)-2-(5-(3,4-dimethylbenzylidene)-2,4-dioxothiazolidin-3-yl)acetamide (GB20)

1. FTIR

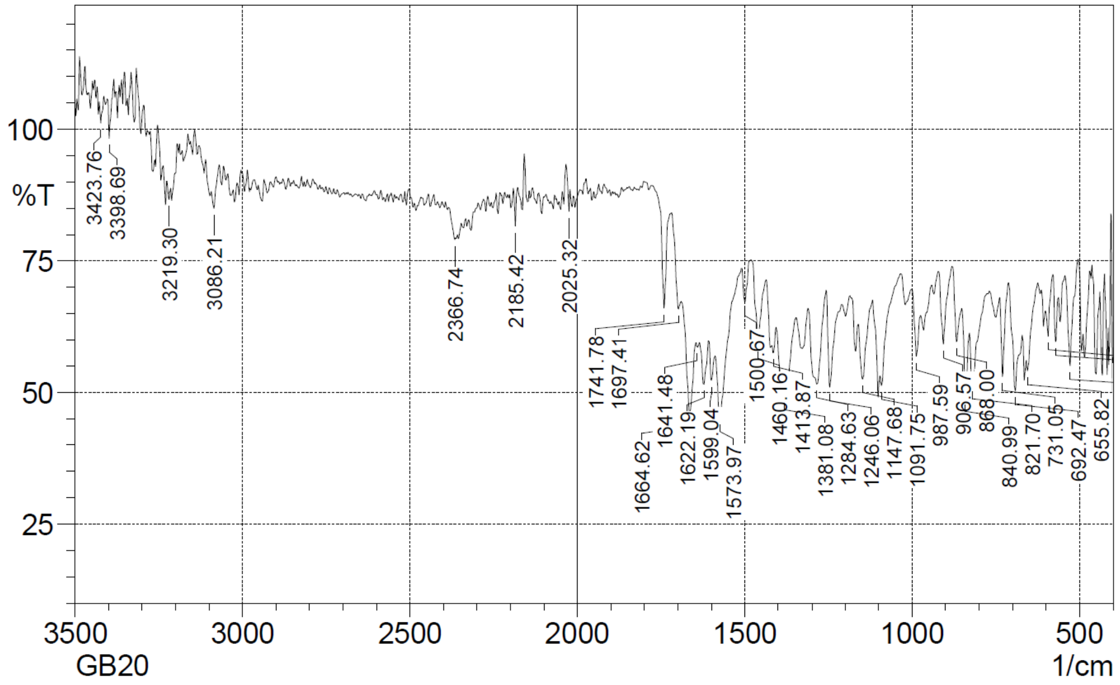

2. 1H-NMR

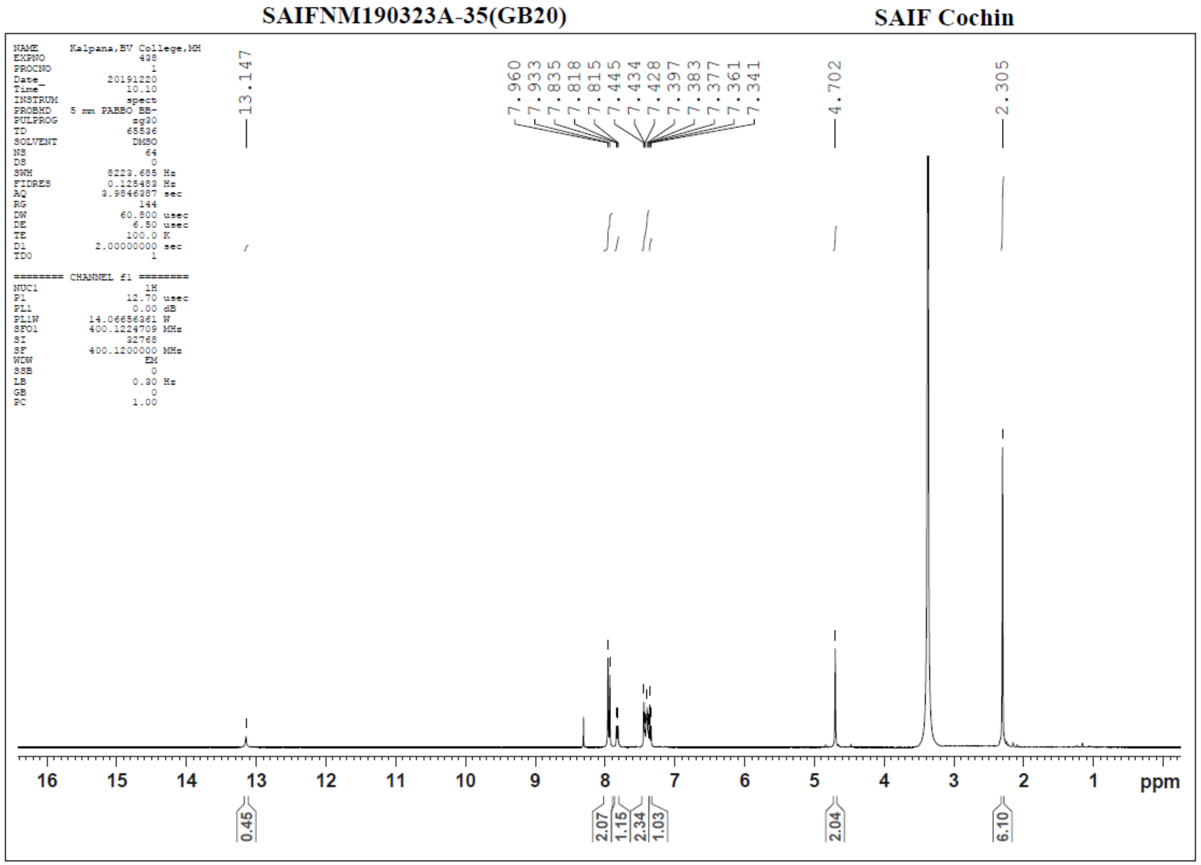
SAIFNM190323A-35(GB20)
SAIF Cochin
NAME Kalpana,BV College,MH
EXPNO 438
PROCNO 1
Date_ 20191220
Time 10.10
INSTRUM spect
PROBHD 5 mm PABBO BB-
PULPROG zg30
TD 65536
SOLVENT DMSO
NS 64
DS 0
SWH 8223.685 Hz
FIDRES 0.125483 Hz
AQ 3.9846387 sec
RG 144
DW 60.800 usec
DE 6.50 usec
TE 100.0 K
D1 2.00000000 sec
TD0 1
======== CHANNEL f1 ========
NUC1 1H
P1 12.70 usec
PL1 0.00 dB
PL1W 14.06656361 W
SFO1 400.1224709 MHz
SI 32768
SF 400.1200000 MHz
WDW EM
SSB 0
LB 0.30 Hz
GB 0
PC 1.00
13.147
7.960
7.933
7.835
7.818
7.815
7.445
7.434
7.428
7.397
7.383
7.377
7.361
7.341
4.702
2.305
16 15 14 13 12 11 10 9 8 7 6 5 4 3 2 1 ppm
0.45
2.07
1.15
2.34
1.03
2.04
6.10

3. 13C-NMR

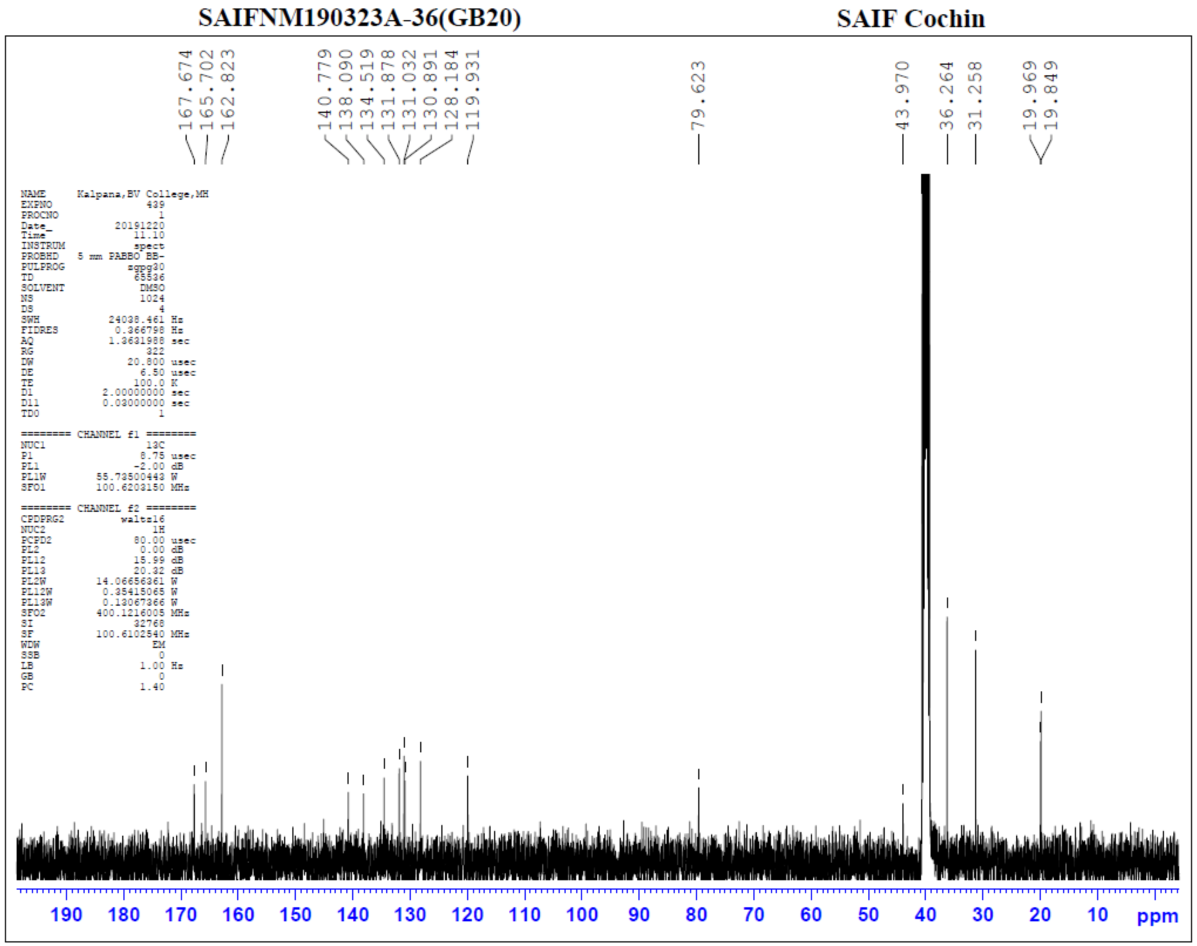
SAIFNM190323A-36(GB20)
SAIF Cochin
167.674
165.702
162.823
140.779
138.090
134.519
131.878
131.032
130.891
128.184
119.931
79.623
43.970
36.264
31.258
19.969
19.849
NAME Kalpana,BV College,MH
EXPNO 439
PROCNO 1
Date_ 20191220
Time 11.10
INSTRUM spect
PROBHD 5 mm PABBO BB-
PULPROG zgpg30
TD 65536
SOLVENT DMSO
NS 1024
DS 4
SWH 24038.461 Hz
FIDRES 0.366798 Hz
AQ 1.3631988 sec
RG 322
DW 20.800 usec
DE 6.50 usec
TE 100.0 K
D1 2.00000000 sec
D11 0.03000000 sec
TD0 1
======== CHANNEL f1 ========
NUC1 13C
P1 8.75 usec
PL1 -2.00 dB
PL1W 55.73500443 W
SFO1 100.6203150 MHz
======== CHANNEL f2 ========
CPDPRG2 waltz16
NUC2 1H
PCPD2 80.00 usec
PL2 0.00 dB
PL12 15.99 dB
PL13 20.32 dB
PL2W 14.06656361 W
PL12W 0.35415065 W
PL13W 0.13067366 W
SFO2 400.1216005 MHz
SI 32768
SF 100.6102540 MHz
WDW EM
SSB 0
LB 1.00 Hz
GB 0
PC 1.40
190 180 170 160 150 140 130 120 110 100 90 80 70 60 50 40 30 20 10 ppm

4. HPLC

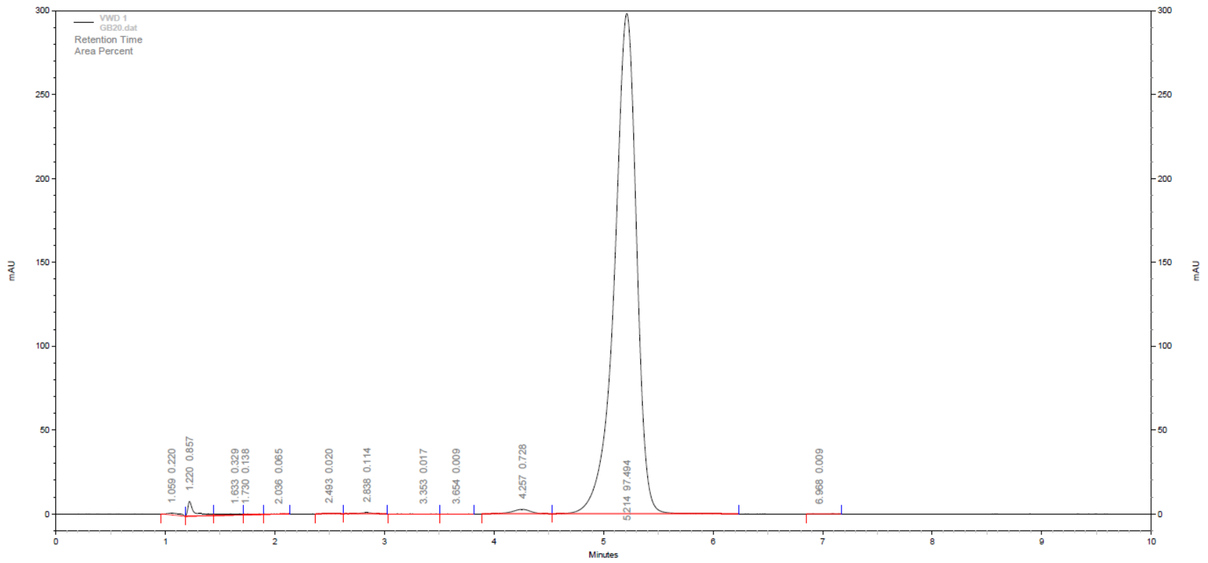
VWD 1
GB20.dat
Retention Time
Area Percent
mAU
Minutes
1.059 0.220
1.220 0.857
1.633 0.329
1.730 0.138
2.036 0.065
2.493 0.020
2.838 0.114
3.353 0.017
3.654 0.009
4.257 0.728
5.214 97.494
6.968 0.009

5. Mass

MS Spectrum

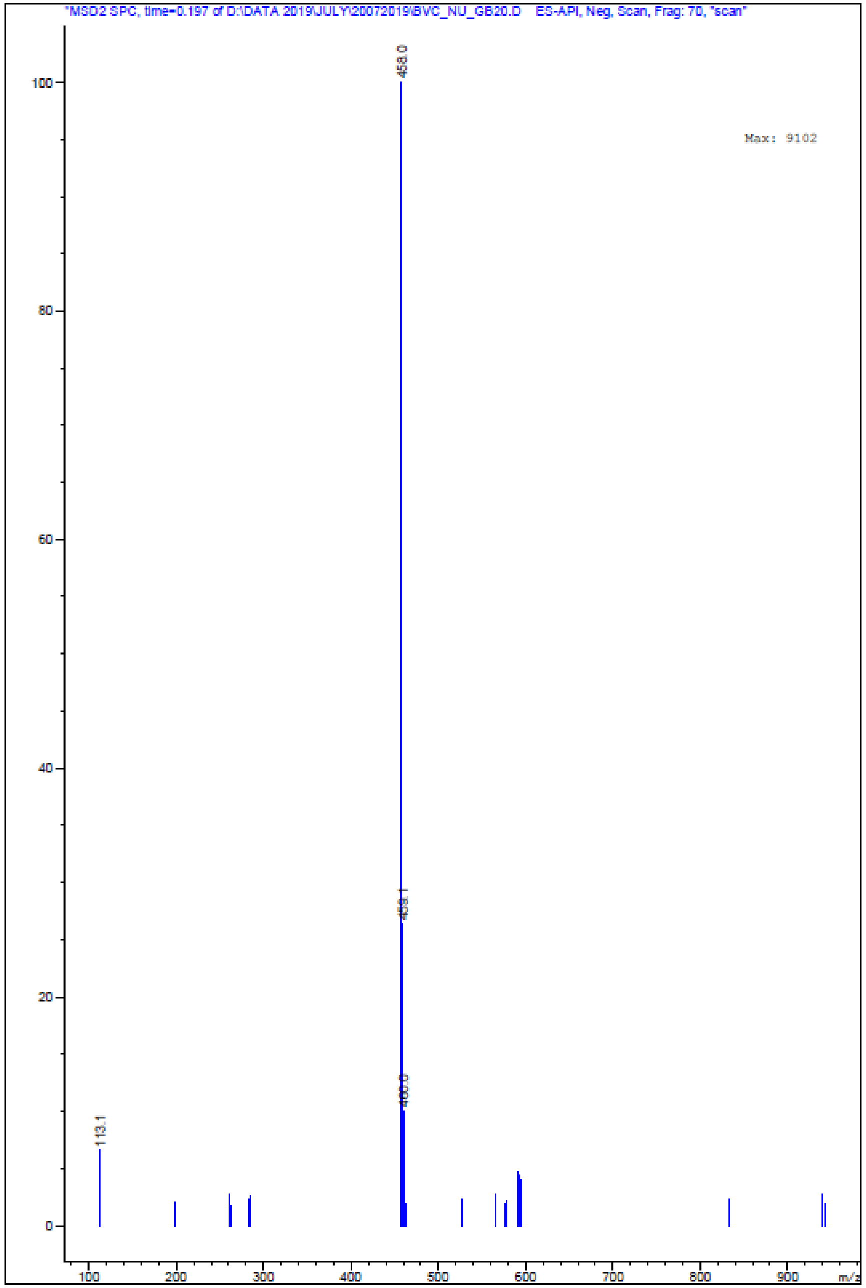

*MSD2 SPC, time=0.197 of D:\DATA 2019\JULY\20072019\BVC_NU_GB20.D ES-API, Neg, Scan, Frag: 70, "scan"
Max: 9102
458.0
459.1
113.1
100
80
60
40
20
0
100
200
300
400
500
600
700
800
900
m/z

N-(5,6-dimethylbenzo[d]thiazol-2-yl)-2-(5-(4-methylbenzylidene)-2,4-dioxothiazolidin-3-yl)acetamide (GB21)

1. FTIR

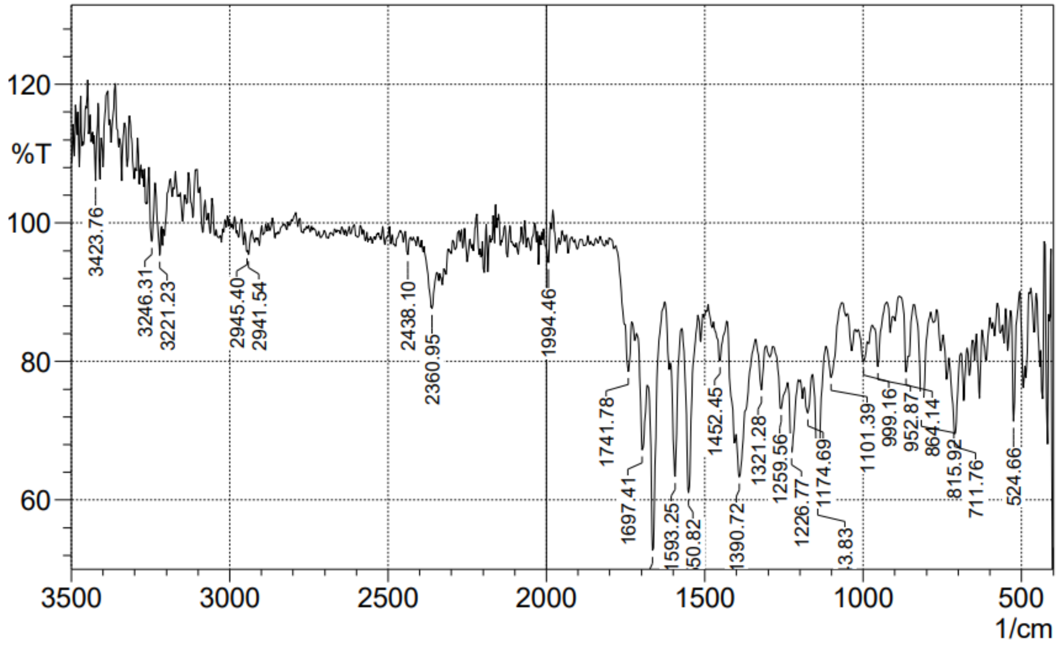

2. ^{1}H-NMR

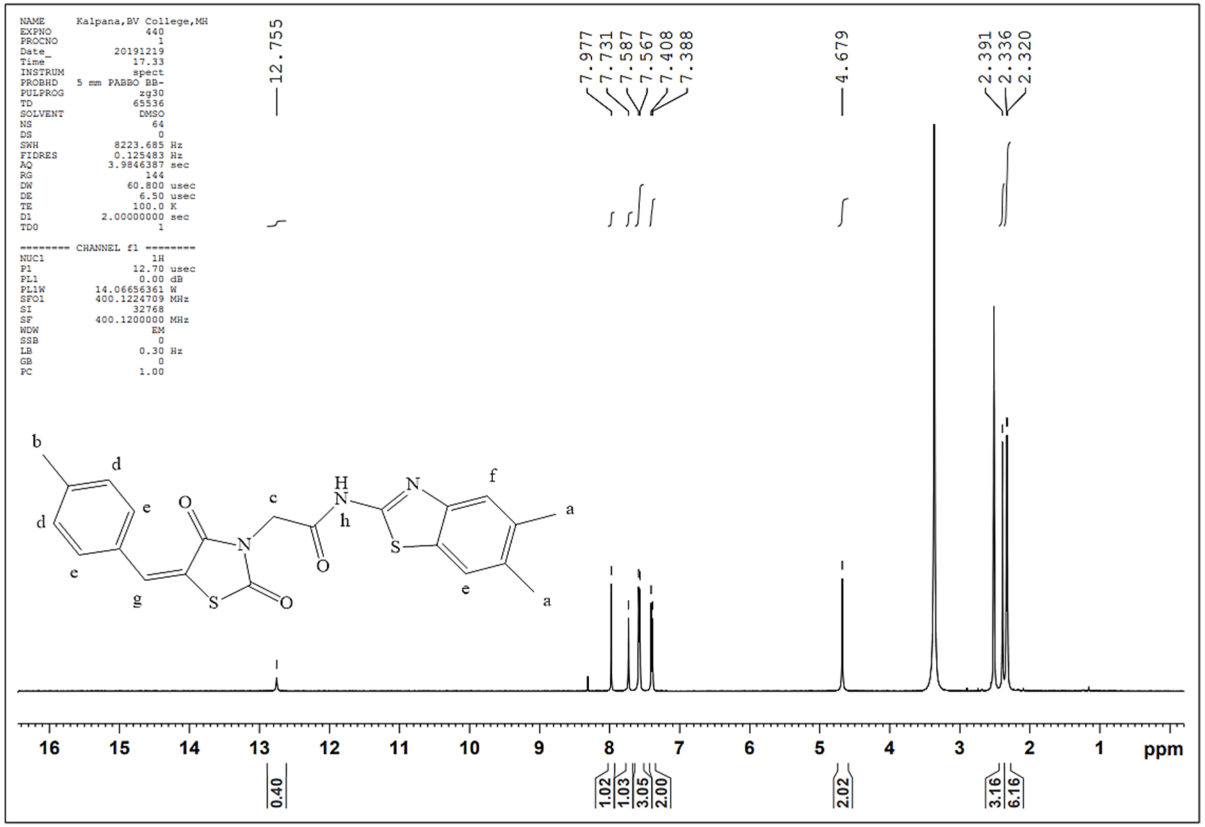
NAME Kalpana,BV College,MH
EXPNO 440
PROCNO 1
Date_ 20191219
Time 17.33
INSTRUM spect
PROBHD 5 mm PABBO BB-
PULPROG zg30
TD 65536
SOLVENT DMSO
NS 64
DS 0
SWH 8223.685 Hz
FIDRES 0.125483 Hz
AQ 3.9846387 sec
RG 144
DW 60.800 usec
DE 6.50 usec
TE 100.0 K
D1 2.00000000 sec
TD0 1
======== CHANNEL f1 ========
NUC1 1H
P1 12.70 usec
PL1 0.00 dB
PL1W 14.06656361 W
SFO1 400.1224709 MHz
SI 32768
SF 400.1200000 MHz
WDW EM
SSB 0
LB 0.30 Hz
GB 0
PC 1.00
12.755
7.977
7.731
7.587
7.567
7.408
7.388
4.679
2.391
2.336
2.320
ppm
0.40
1.02
1.03
3.05
2.00
2.02
3.16
6.16

3. ^{13}C-NMR

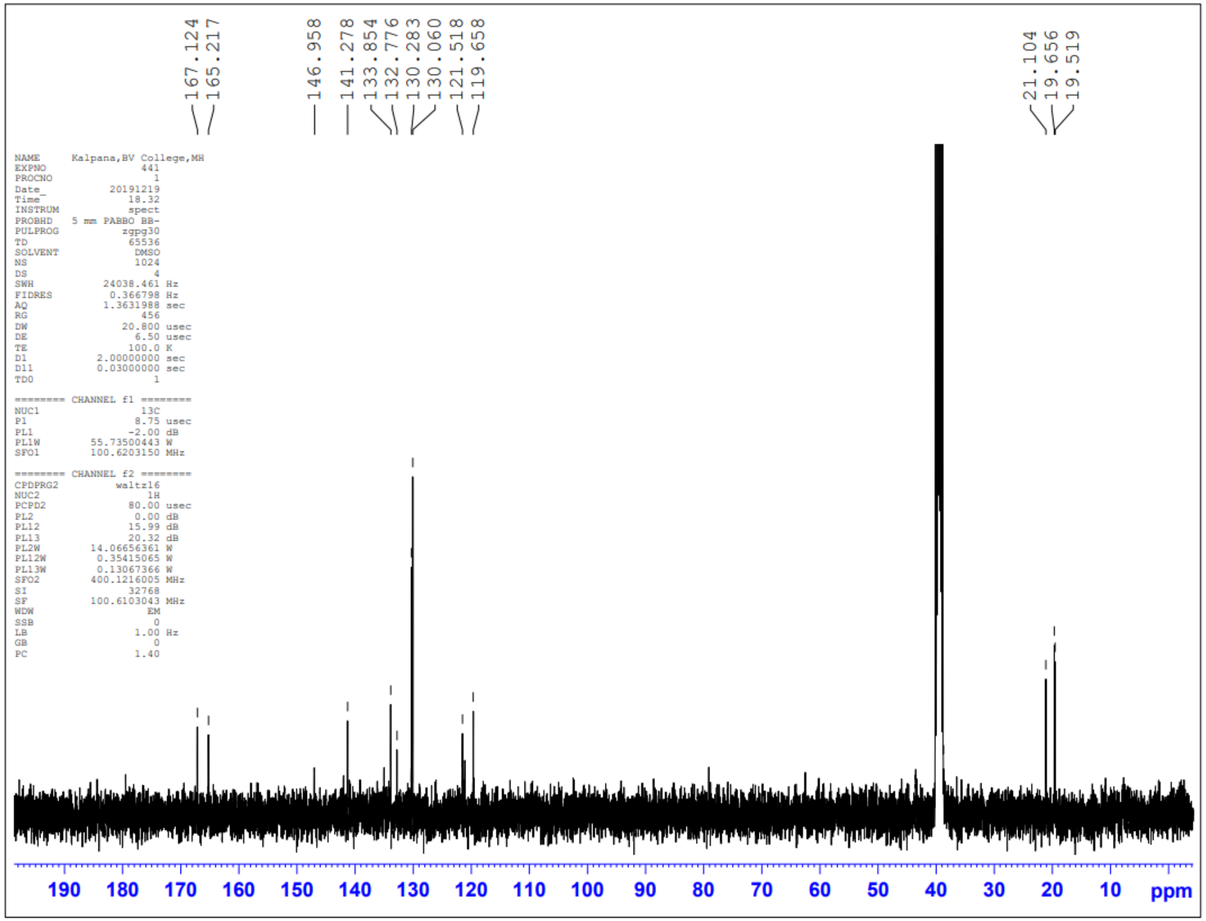

4. HPLC Analysis

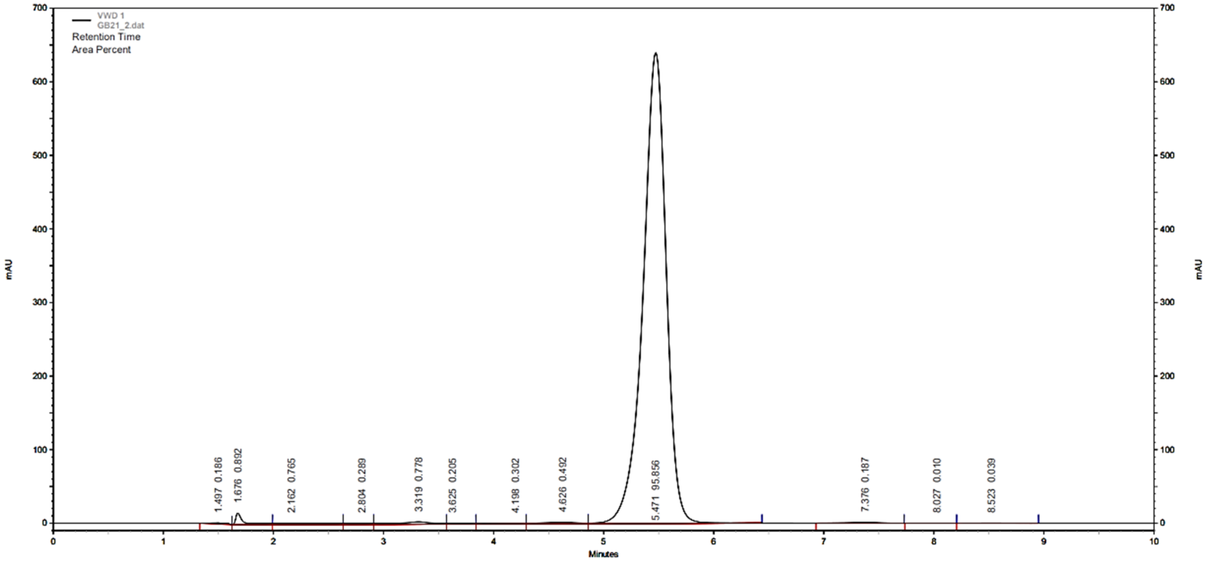

5. Mass Spectrometry

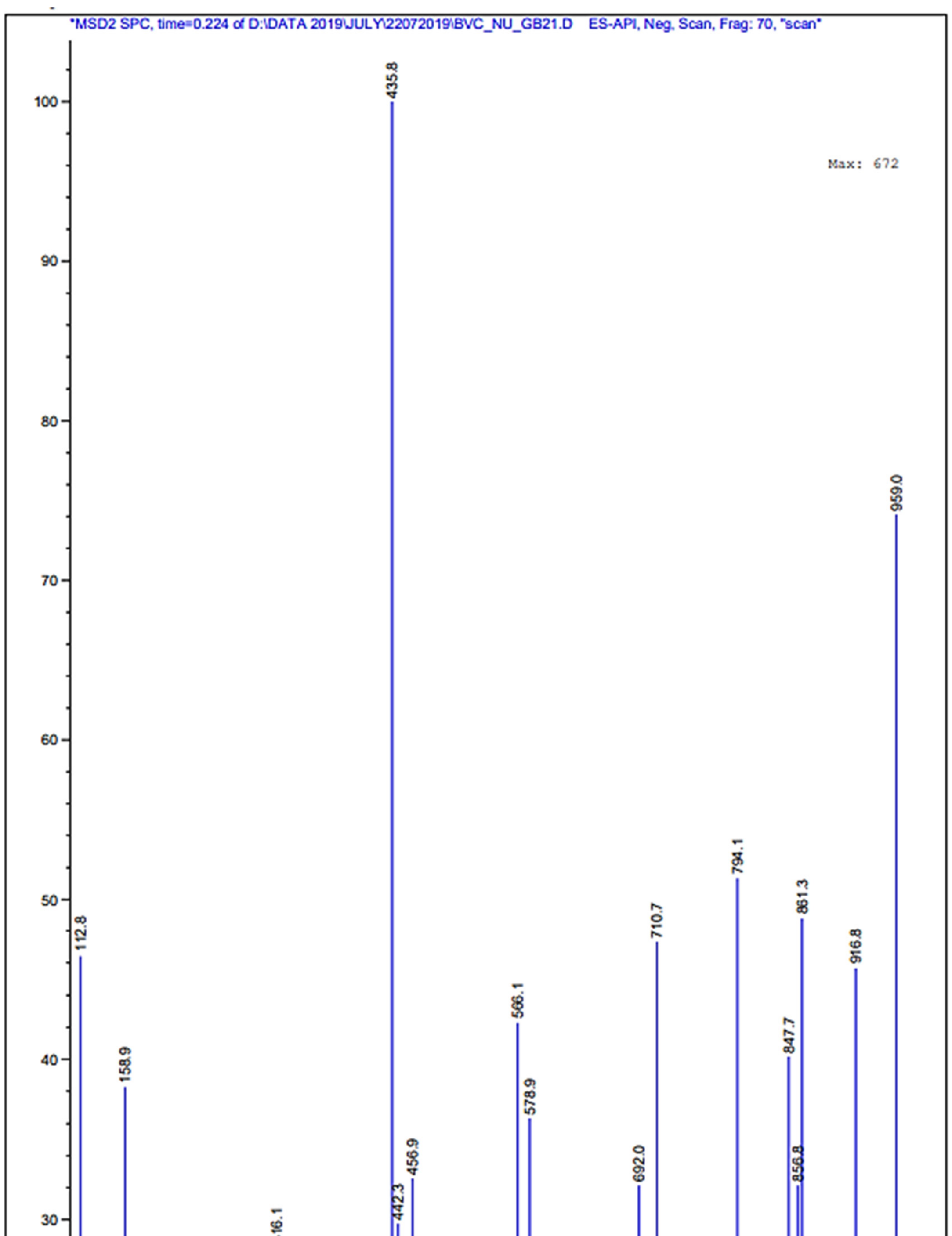
*MSD2 SPC, time=0.224 of D:\DATA 2019\JULY\22072019\BVC_NU_GB21.D ES-API, Neg, Scan, Frag: 70, "scan"
Max: 672
100
90
80
70
60
50
40
30
435.8
959.0
794.1
861.3
710.7
916.8
112.8
566.1
847.7
158.9
578.9
456.9
692.0
856.8
442.3
16.1

6. UV

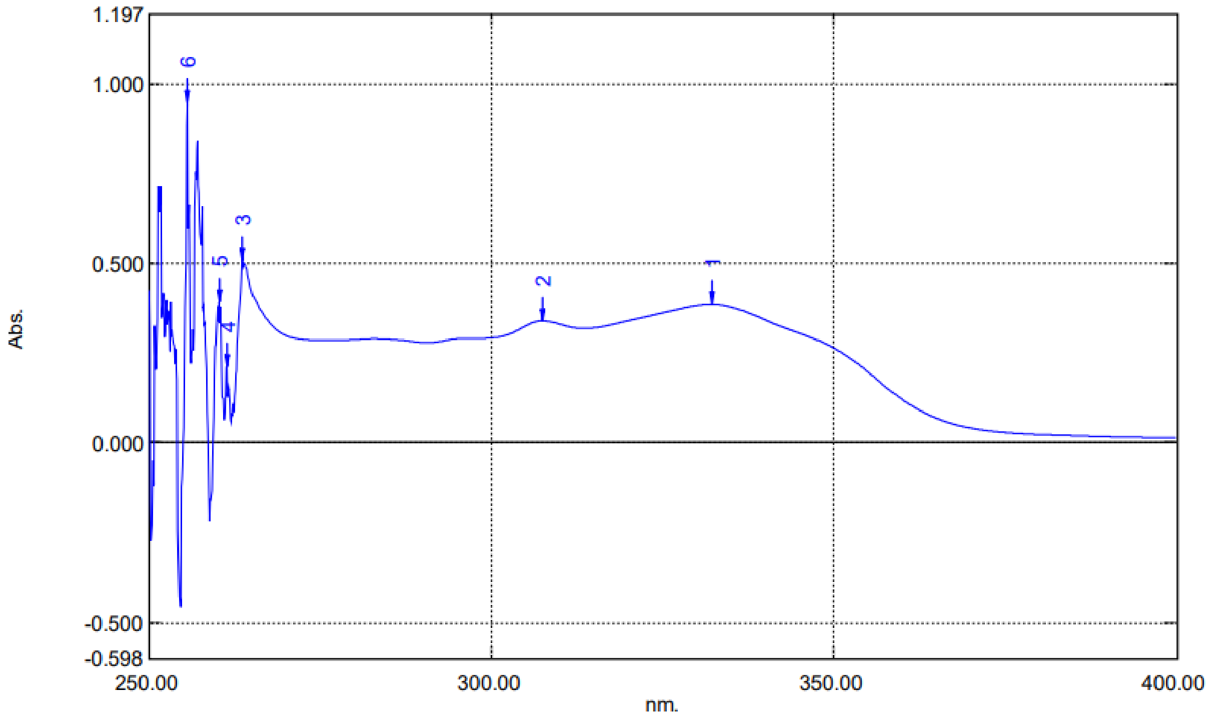

1.197
1.000
0.500
0.000
-0.500
-0.598
Abs.
250.00
300.00
350.00
400.00
nm.
6
5
4
3
2
1

2-(5-benzylidene-2,4-dioxothiazolidin-3-yl)-N-(5,6-dimethylbenzo[d]thiazol-2-yl)acetamide (GB22)

1. FTIR

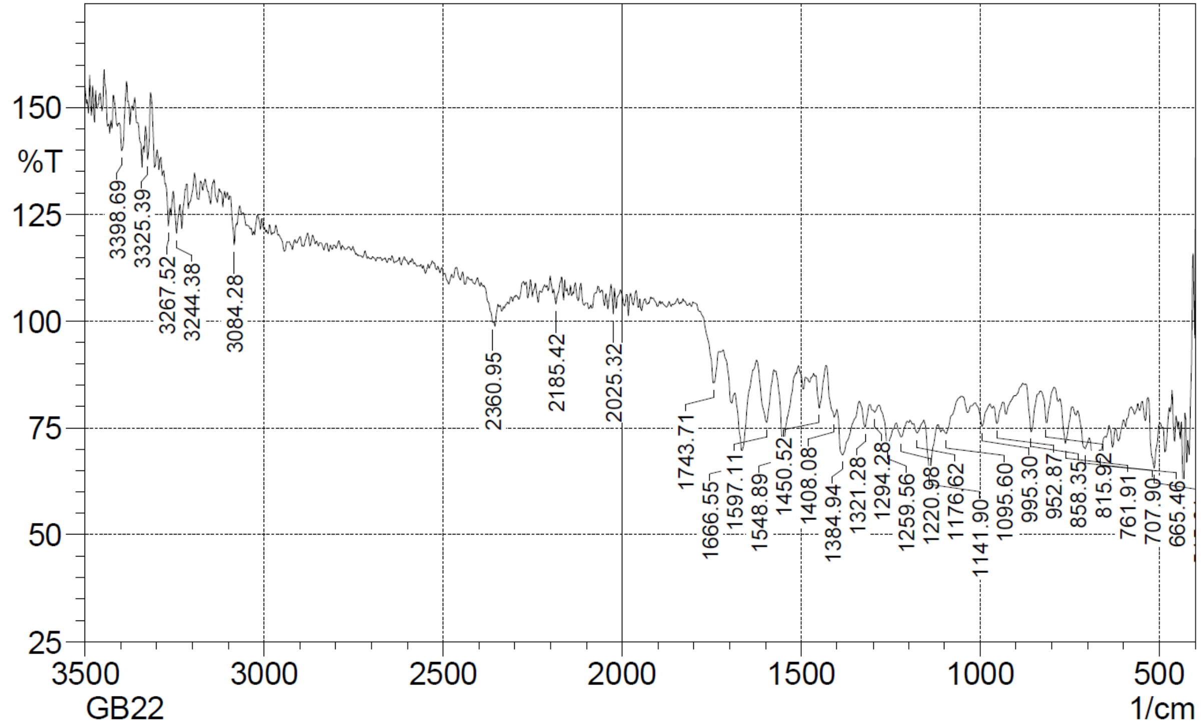

2. ^{1}H-NMR

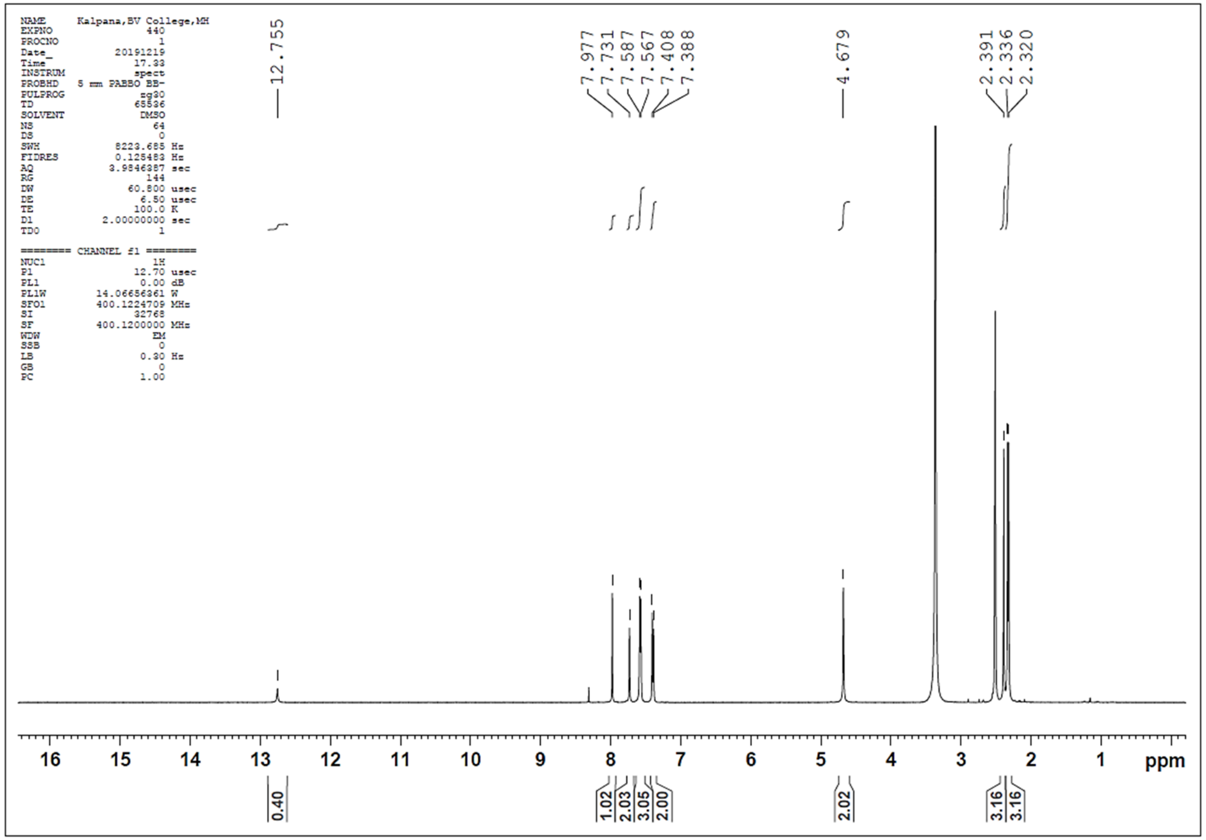
NAME Kalpana,BV College,MH
EXPNO 440
PROCNO 1
Date_ 20191219
Time 17.33
INSTRUM spect
PROBHD 5 mm PABBO BB-
PULPROG zg30
TD 65536
SOLVENT DMSO
NS 64
DS 0
SWH 8223.685 Hz
FIDRES 0.125483 Hz
AQ 3.9846387 sec
RG 144
DW 60.800 usec
DE 6.50 usec
TE 100.0 K
D1 2.00000000 sec
TD0 1
======== CHANNEL f1 ========
NUC1 1H
P1 12.70 usec
PL1 0.00 dB
PL1W 14.06656361 W
SFO1 400.1224709 MHz
SI 32768
SF 400.1200000 MHz
WDW EM
SSB 0
LB 0.30 Hz
GB 0
PC 1.00
12.755
7.977
7.731
7.587
7.567
7.408
7.388
4.679
2.391
2.336
2.320
16 15 14 13 12 11 10 9 8 7 6 5 4 3 2 1 ppm
0.40
1.02
2.03
3.05
2.00
2.02
3.16
3.16

3. ^{13}C-NMR

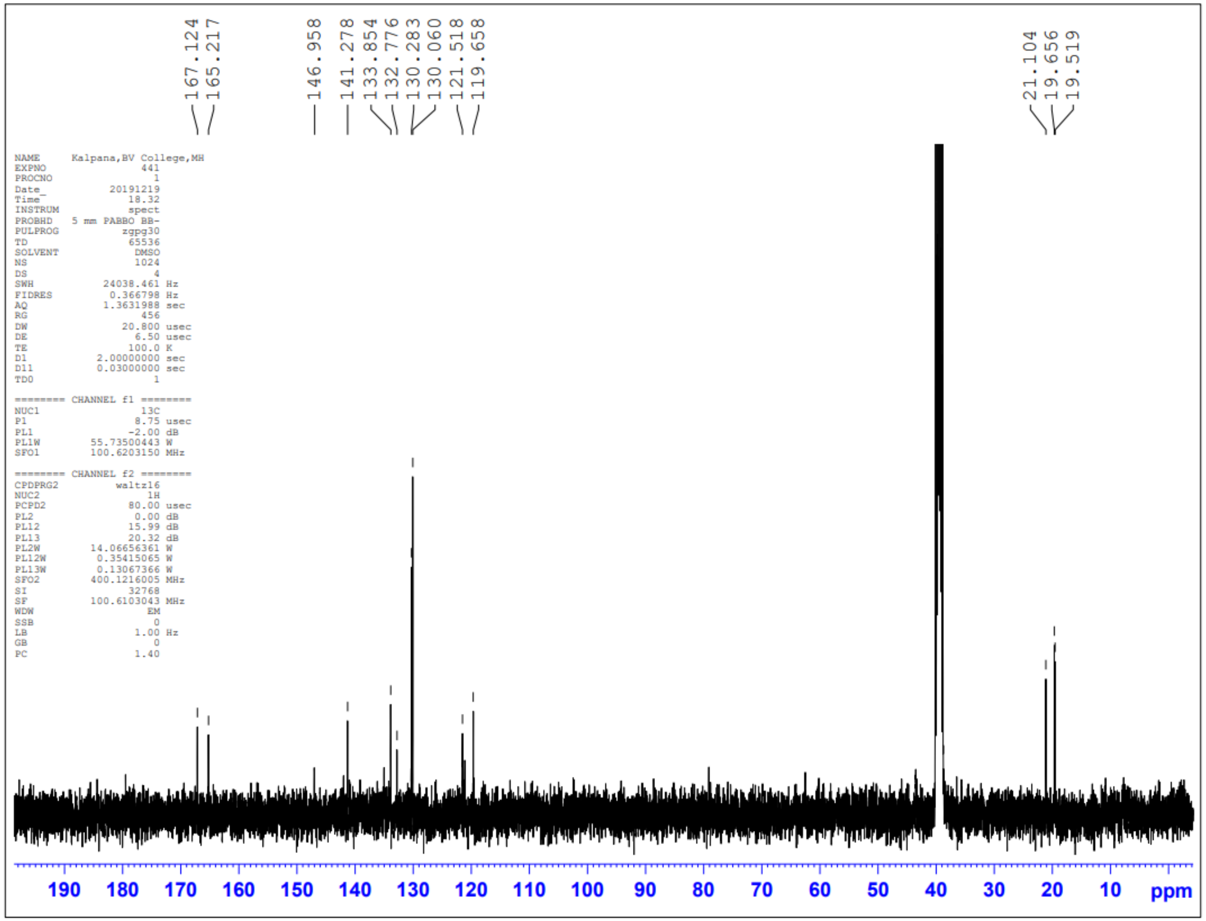

167.124
165.217
146.958
141.278
133.854
132.776
130.283
130.060
121.518
119.658
21.104
19.656
19.519
NAME Kalpana,BV College,MH
EXPNO 441
PROCNO 1
Date_ 20191219
Time 18.32
INSTRUM spect
PROBHD 5 mm PABBO BB-
PULPROG zgpg30
TD 65536
SOLVENT DMSO
NS 1024
DS 4
SWH 24038.461 Hz
FIDRES 0.366798 Hz
AQ 1.3631988 sec
RG 456
DW 20.800 usec
DE 6.50 usec
TE 100.0 K
D1 2.00000000 sec
D11 0.03000000 sec
TD0 1
======== CHANNEL f1 ========
NUC1 13C
P1 8.75 usec
PL1 -2.00 dB
PL1W 55.73500443 W
SFO1 100.6203150 MHz
======== CHANNEL f2 ========
CPDPRG2 waltz16
NUC2 1H
PCPD2 80.00 usec
PL2 0.00 dB
PL12 15.99 dB
PL13 20.32 dB
PL2W 14.06656361 W
PL12W 0.35415065 W
PL13W 0.13067366 W
SFO2 400.1216005 MHz
SI 32768
SF 100.6103043 MHz
WDW EM
SSB 0
LB 1.00 Hz
GB 0
PC 1.40
190 180 170 160 150 140 130 120 110 100 90 80 70 60 50 40 30 20 10 ppm

4. HPLC

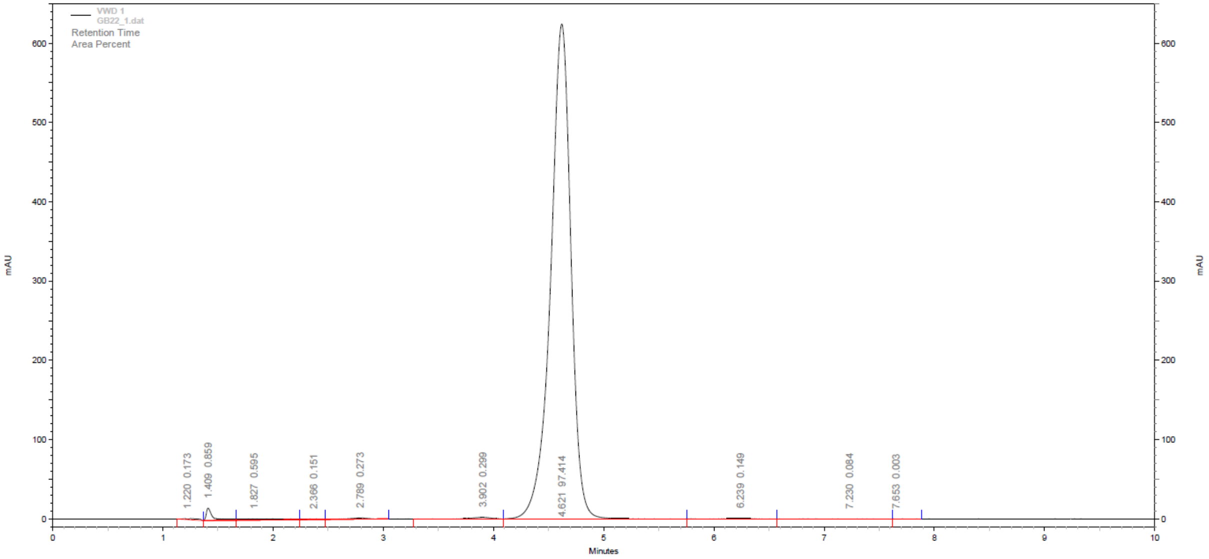
VWD 1
GB22_1.dat
Retention Time
Area Percent
mAU
Minutes
1.220 0.173
1.409 0.859
1.827 0.595
2.366 0.151
2.789 0.273
3.902 0.299
4.621 97.414
6.239 0.149
7.230 0.084
7.653 0.003

5. Mass

MS Spectrum

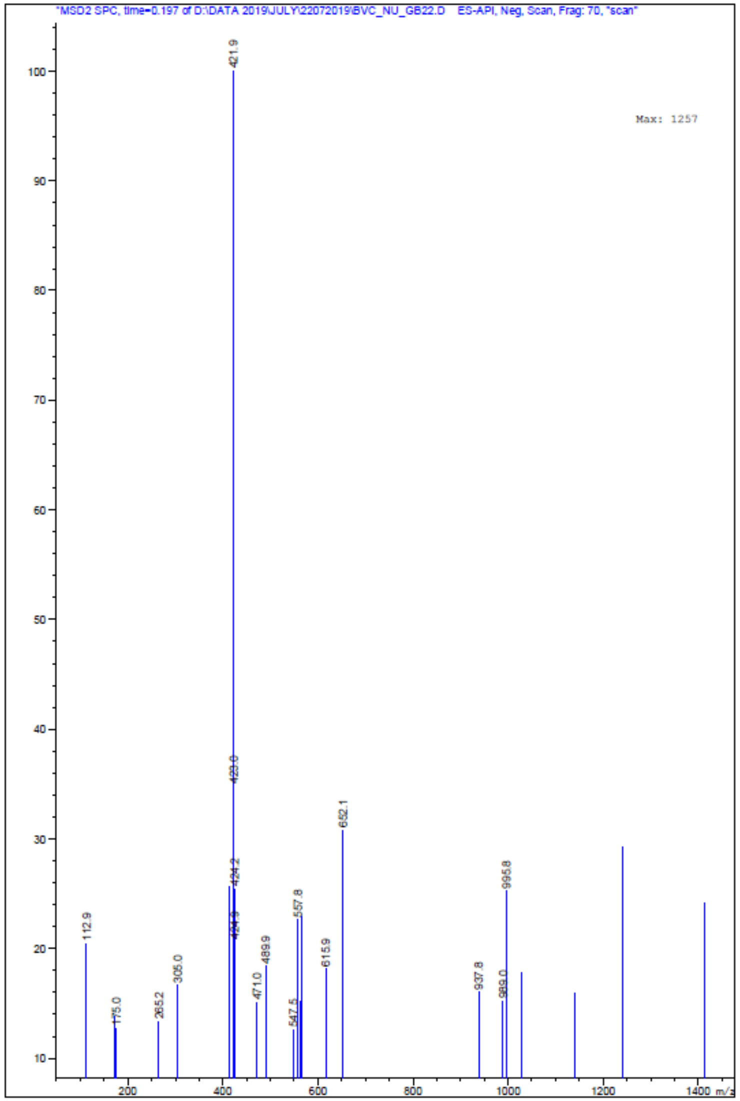
*MSD2 SPC, time=0.197 of D:\DATA 2019\JULY\22072019\BVC_NU_GB22.D ES-API, Neg, Scan, Frag: 70, "scan"
Max: 1257
421.9
423.0
424.2
652.1
995.8
557.8
112.9
489.9
615.9
305.0
937.8
471.0
265.2
989.0
175.0
547.5
m/z

2-(5-(4-bromobenzylidene)-2,4-dioxothiazolidin-3-yl)-N-(5,6-dimethylbenzo[d]thiazol-2-yl)acetamide (GB23)

1. FTIR

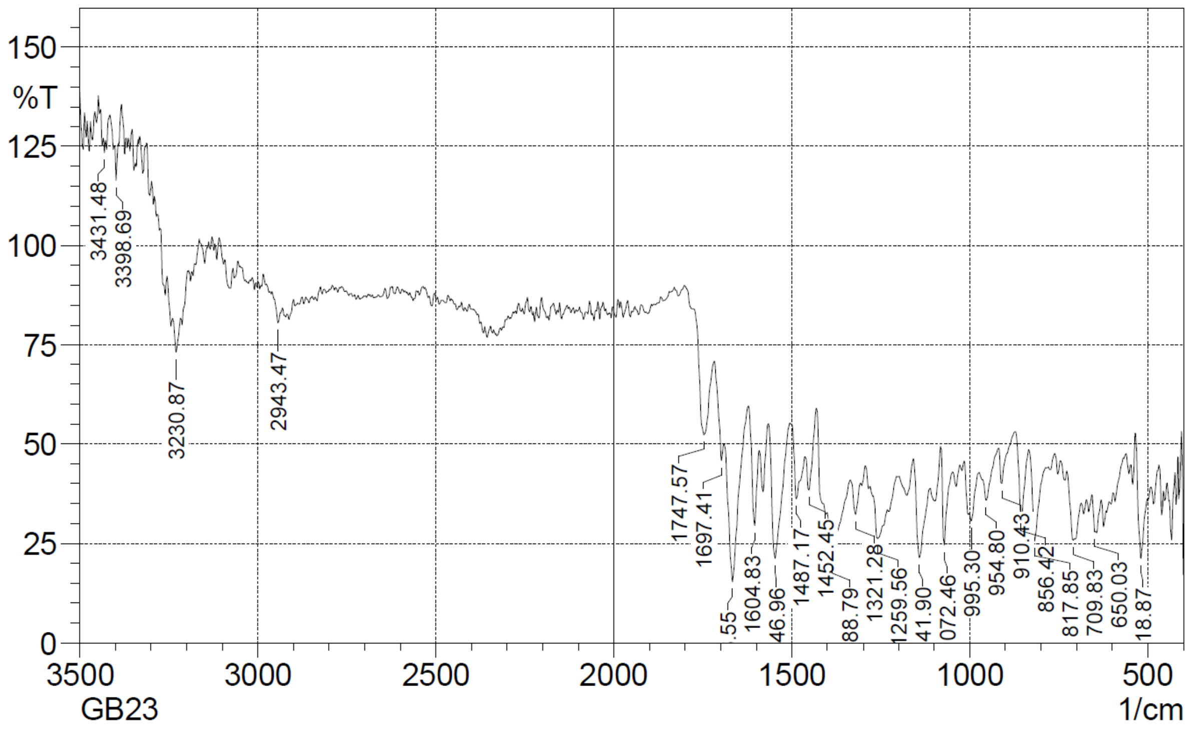

2. ^{1}HNMR

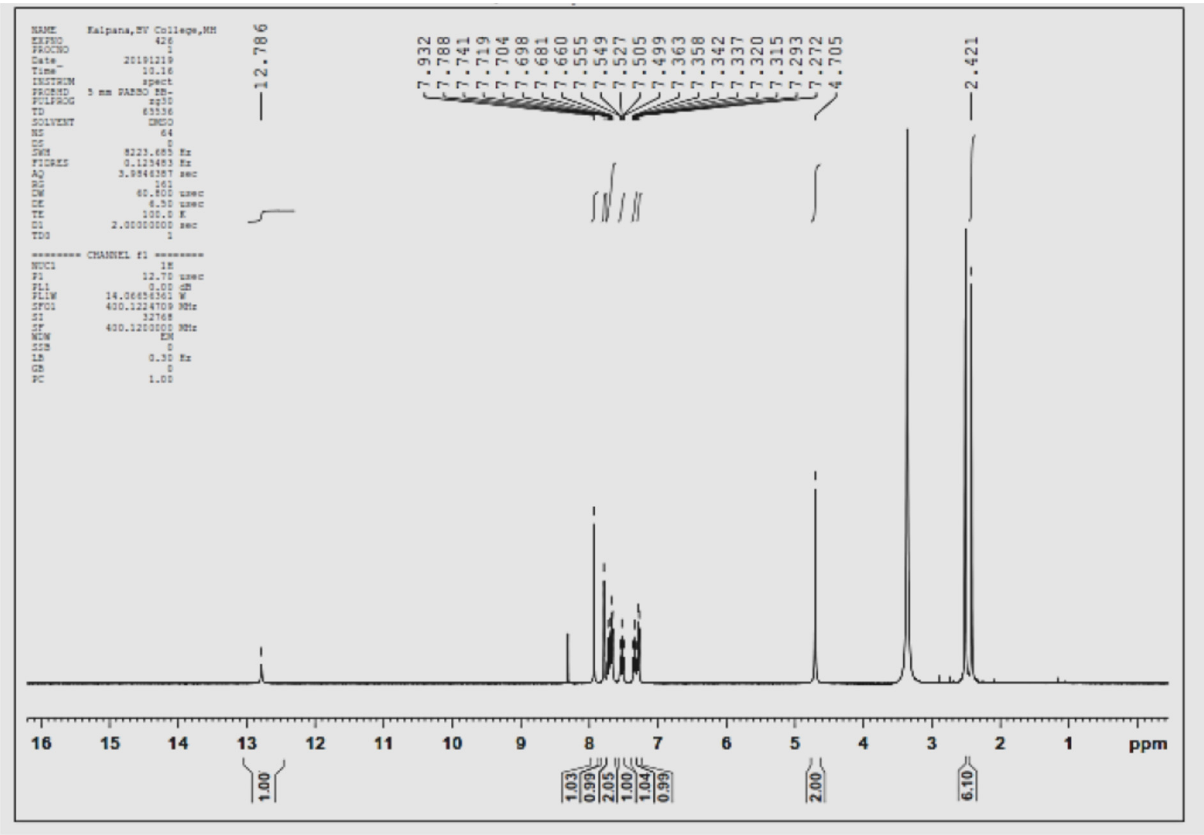
12.786
7.932
7.788
7.741
7.719
7.704
7.698
7.681
7.660
7.555
7.549
7.527
7.505
7.499
7.363
7.358
7.342
7.337
7.320
7.315
7.293
7.272
4.705
2.421
16 15 14 13 12 11 10 9 8 7 6 5 4 3 2 1 ppm
1.00
1.03
0.99
2.05
1.00
1.04
0.99
2.00
6.10

3. ^{13}CNMR

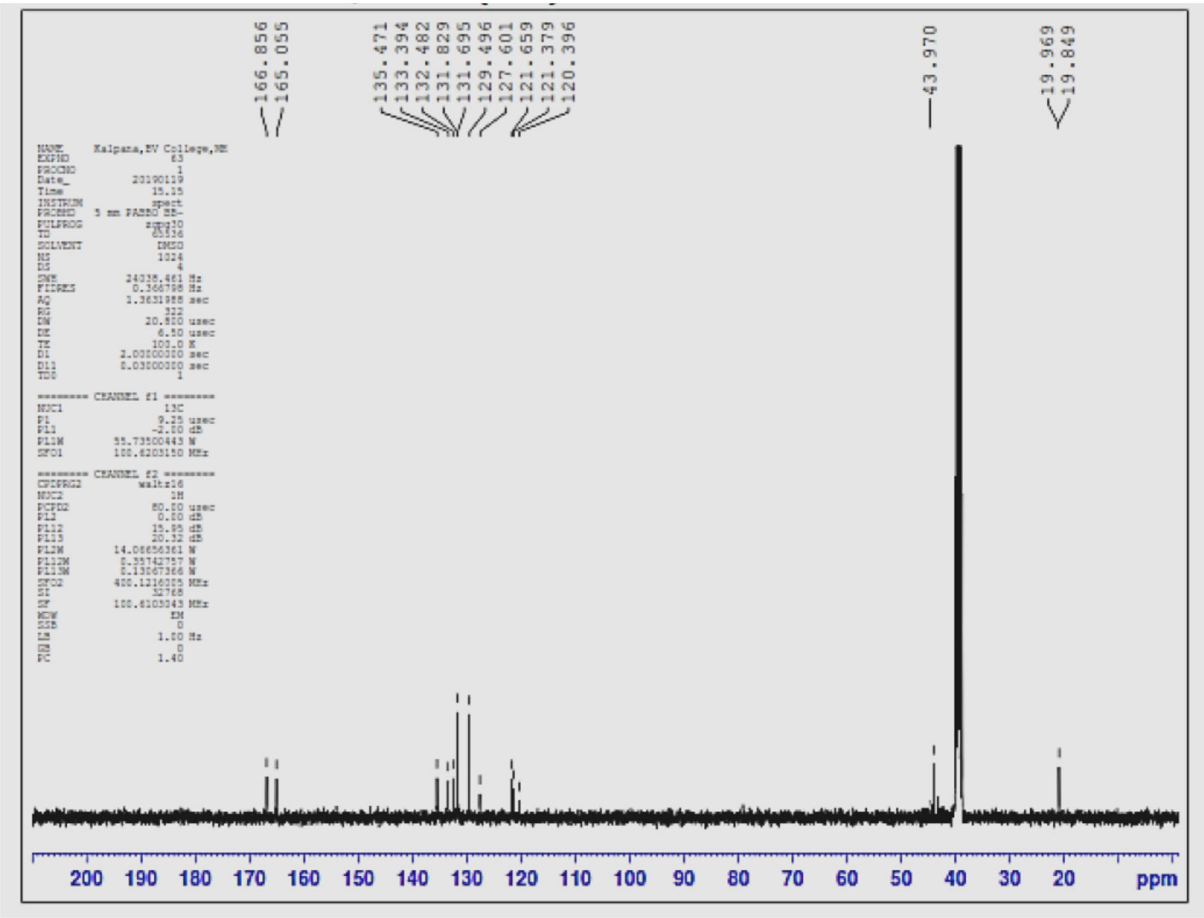
166.856
165.055
135.471
133.394
132.482
131.829
131.695
129.496
127.601
121.659
121.379
120.396
43.970
19.969
19.849
ppm

4. HPLC

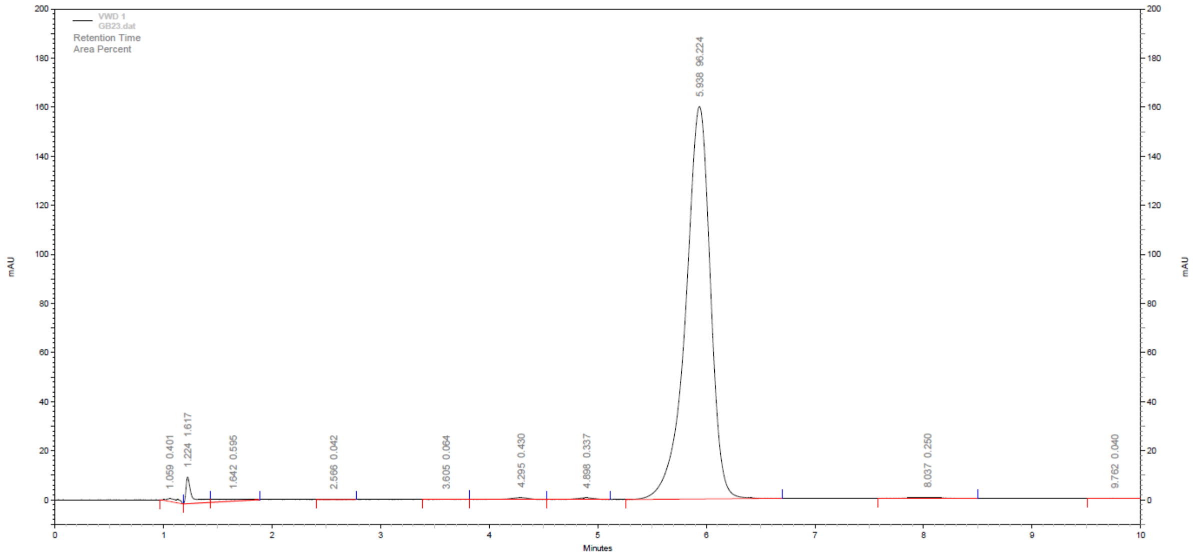
VWD 1
GB23.dat
Retention Time
Area Percent
1.059 0.401
1.224 1.617
1.642 0.595
2.566 0.042
3.605 0.064
4.295 0.430
4.898 0.337
5.938 96.224
8.037 0.250
9.762 0.040
mAU
Minutes

5. Mass

MS Spectrum

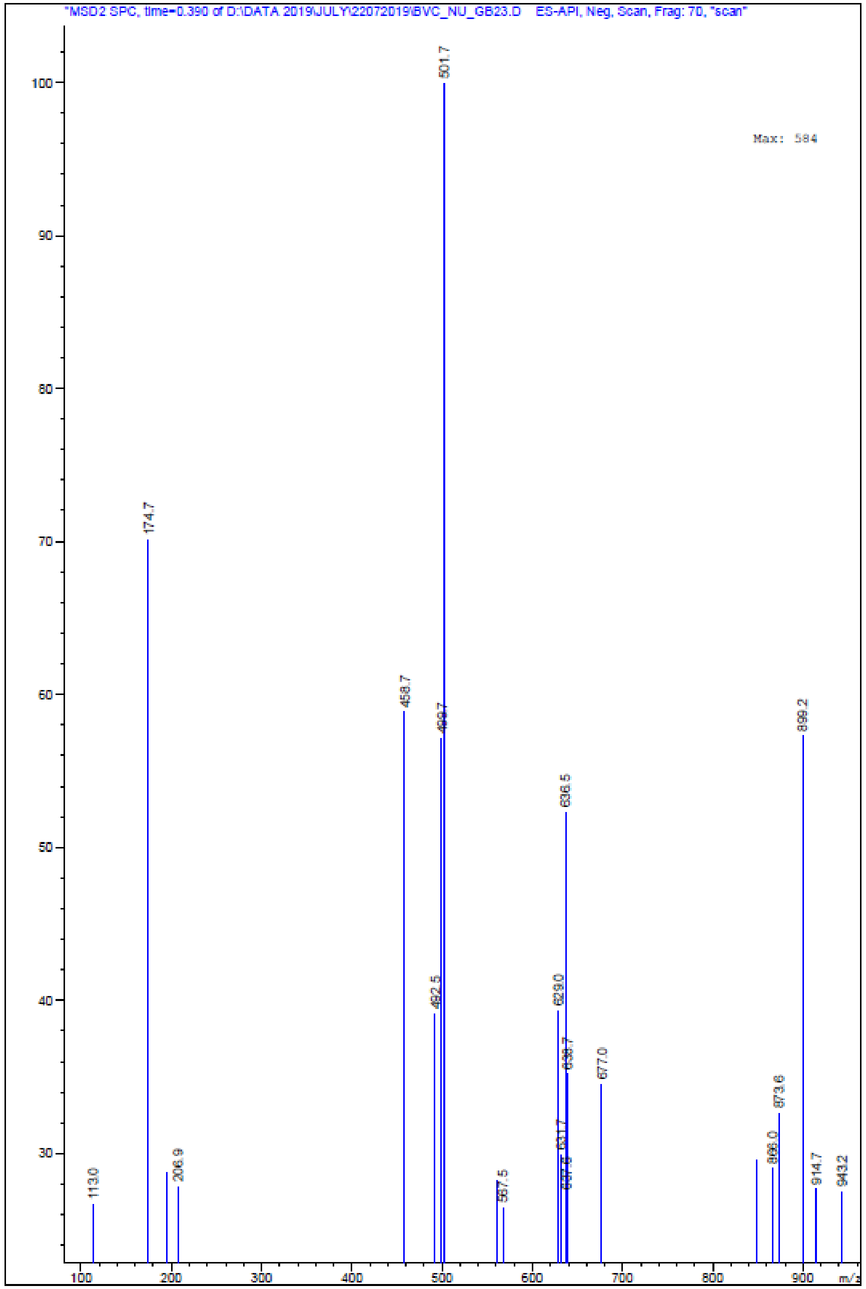
"MSD2 SPC, time=0.390 of D:\DATA 2019\JULY\22072019\BVC_NU_GB23.D   ES-API, Neg, Scan, Frag: 70, "scan"
Max: 584
501.7
174.7
458.7
899.2
636.5
492.5
629.0
677.0
873.6
113.0
206.9
567.5
914.7
943.2
100
90
80
70
60
50
40
30
100
200
300
400
500
600
700
800
900
m/z

2-(5-(4-chlorobenzylidene)-2,4-dioxothiazolidin-3-yl)-N-(5,6-dimethylbenzo[d]thiazol-2-yl)acetamide (GB24)

1. FTIR –

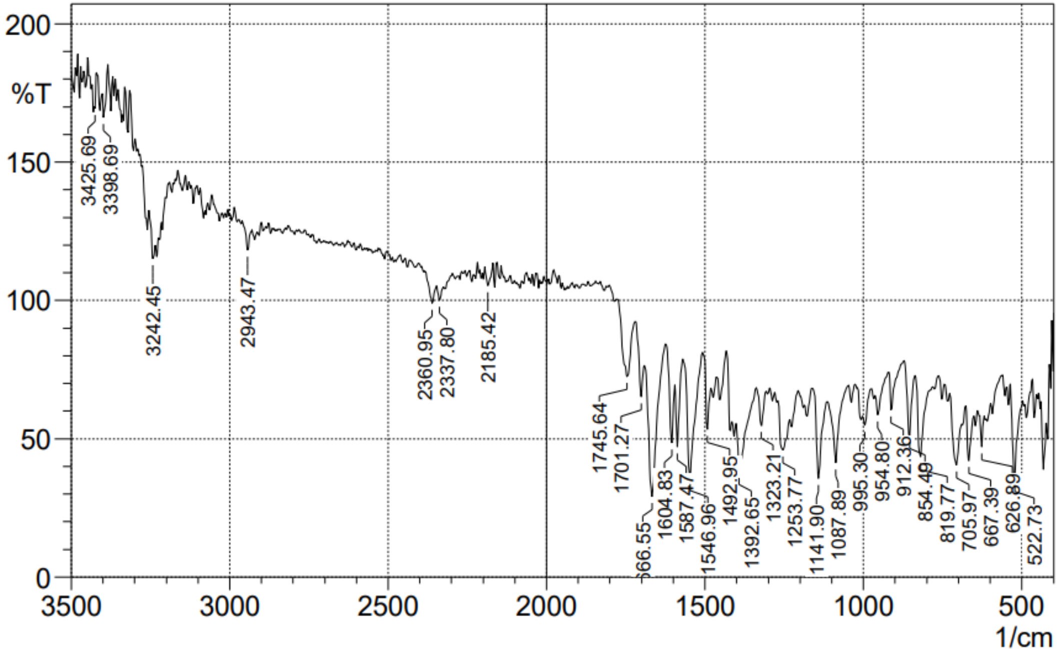

2. ^{1}H-NMR

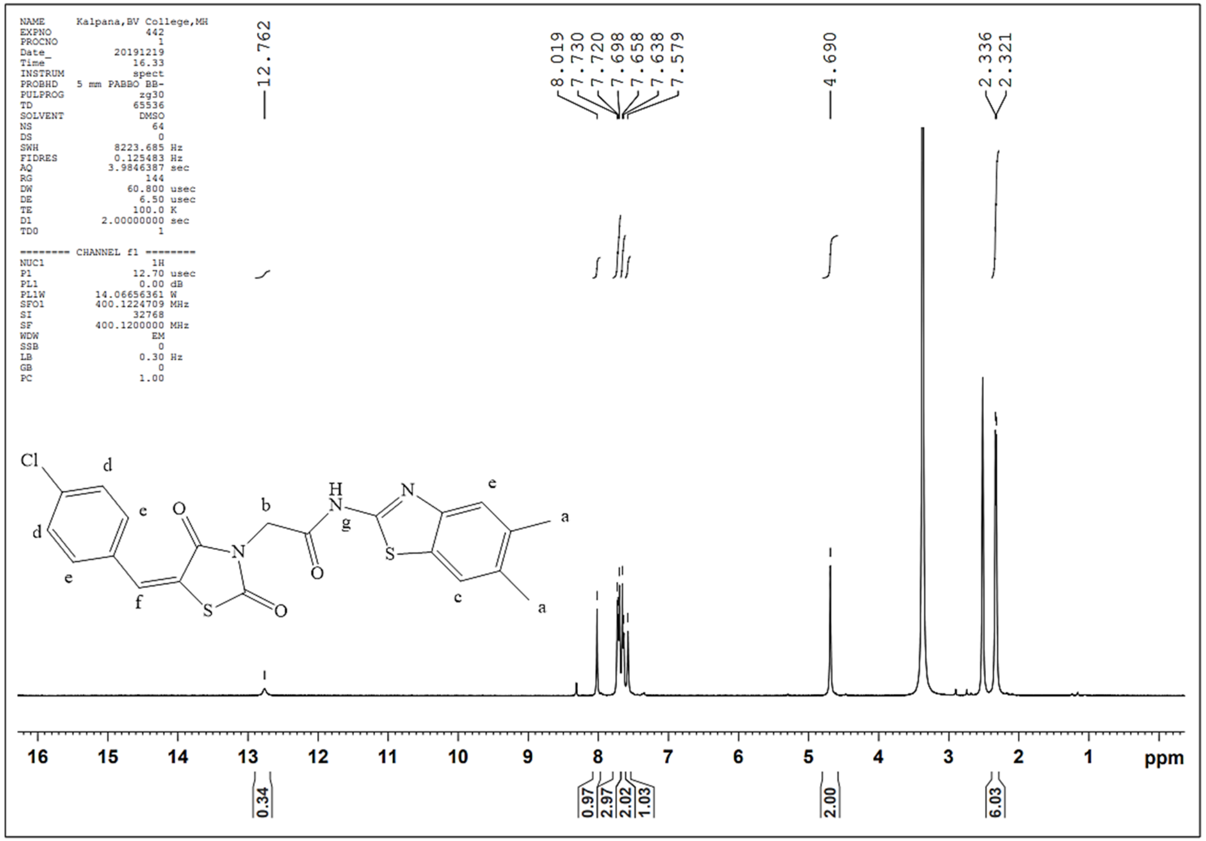

3. ^{13}C-NMR

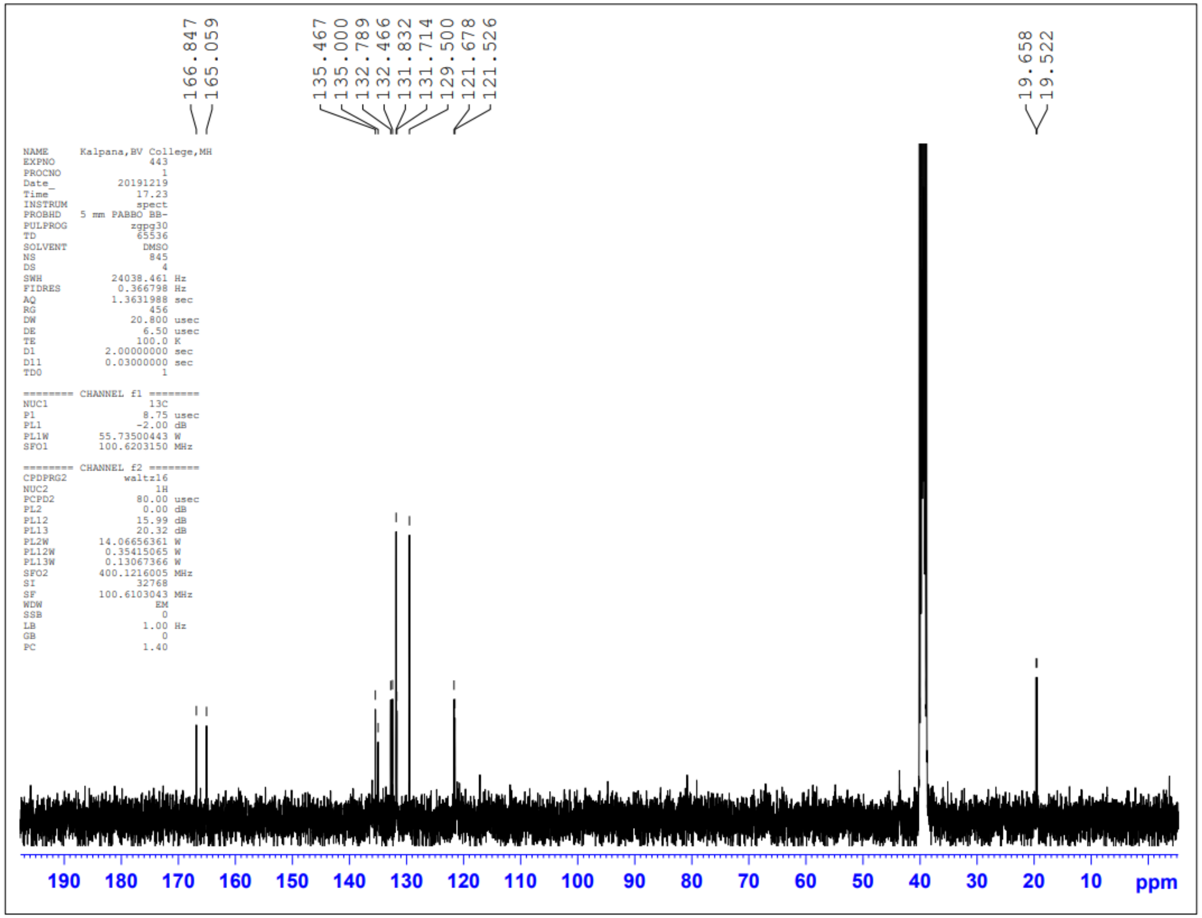

4. HPLC Analysis

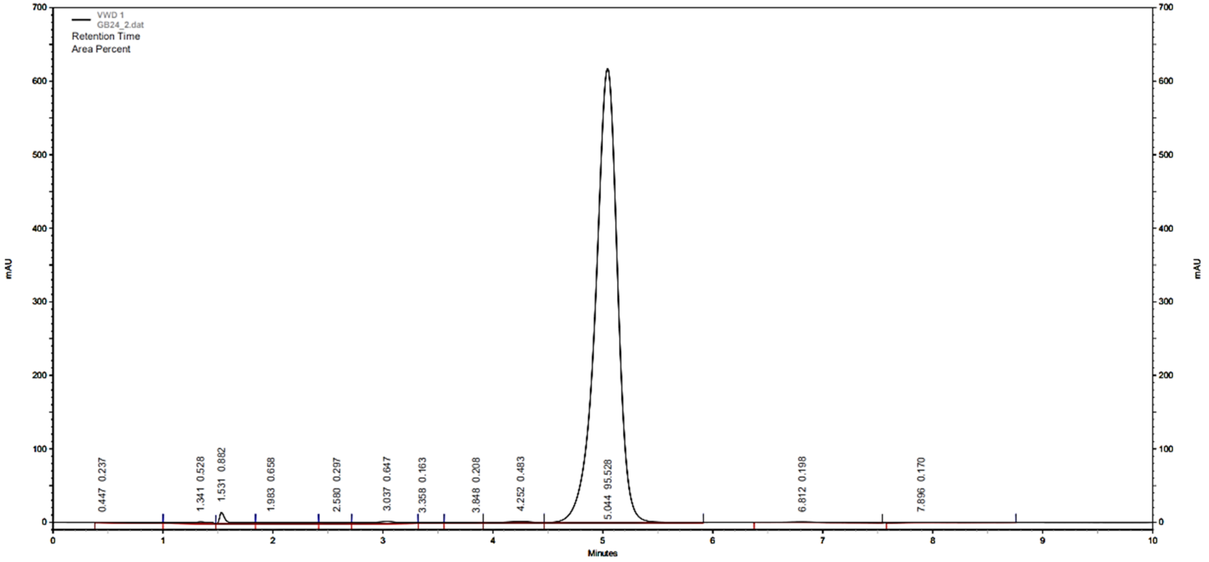

5. Mass Spectrometry

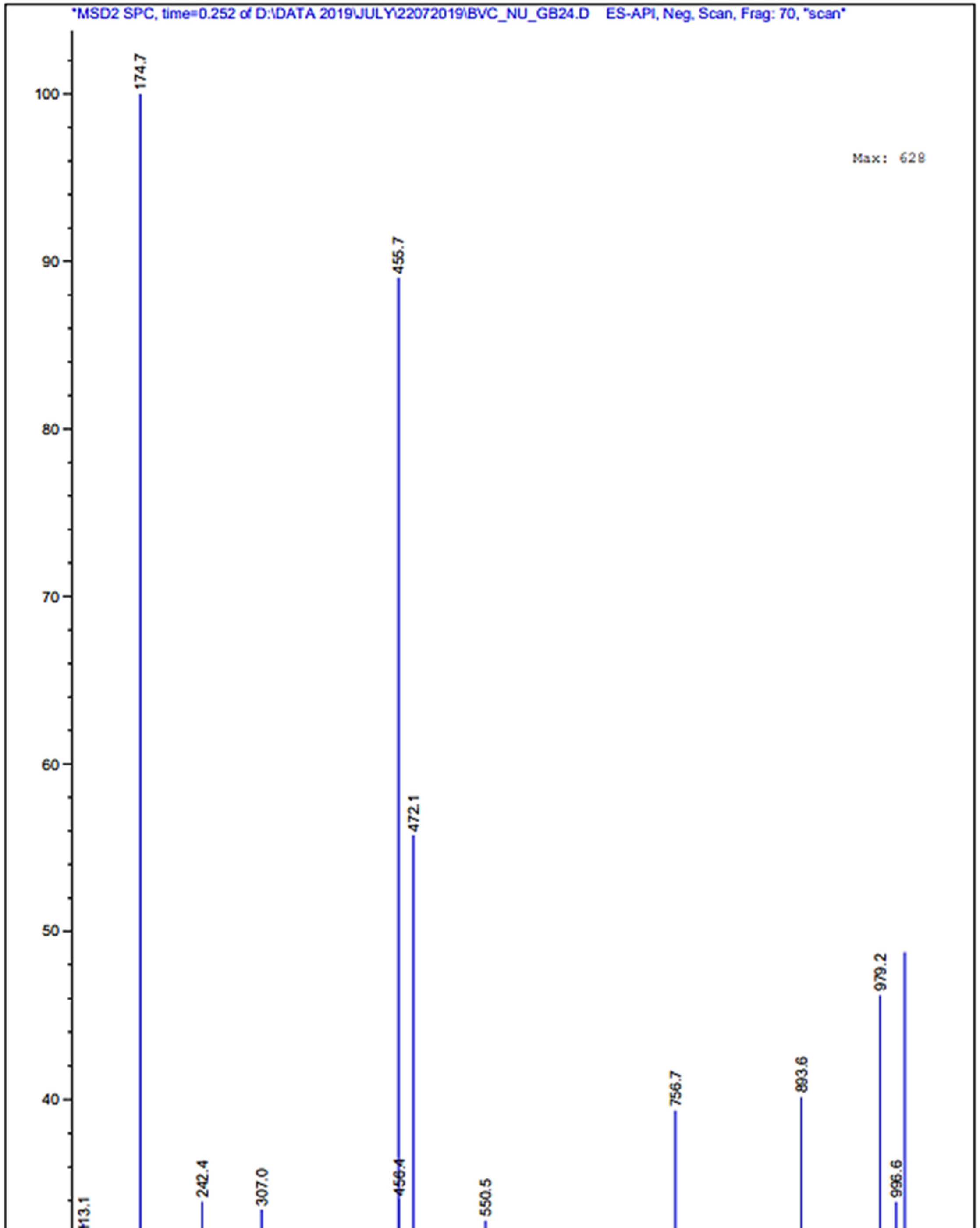
*MSD2 SPC, time=0.252 of D:\DATA 2019\JULY\22072019\BVC_NU_GB24.D ES-API, Neg, Scan, Frag: 70, "scan"
Max: 628
100
90
80
70
60
50
40
174.7
455.7
472.1
979.2
756.7
893.6
242.4
307.0
456.4
550.5
996.6

6. UV

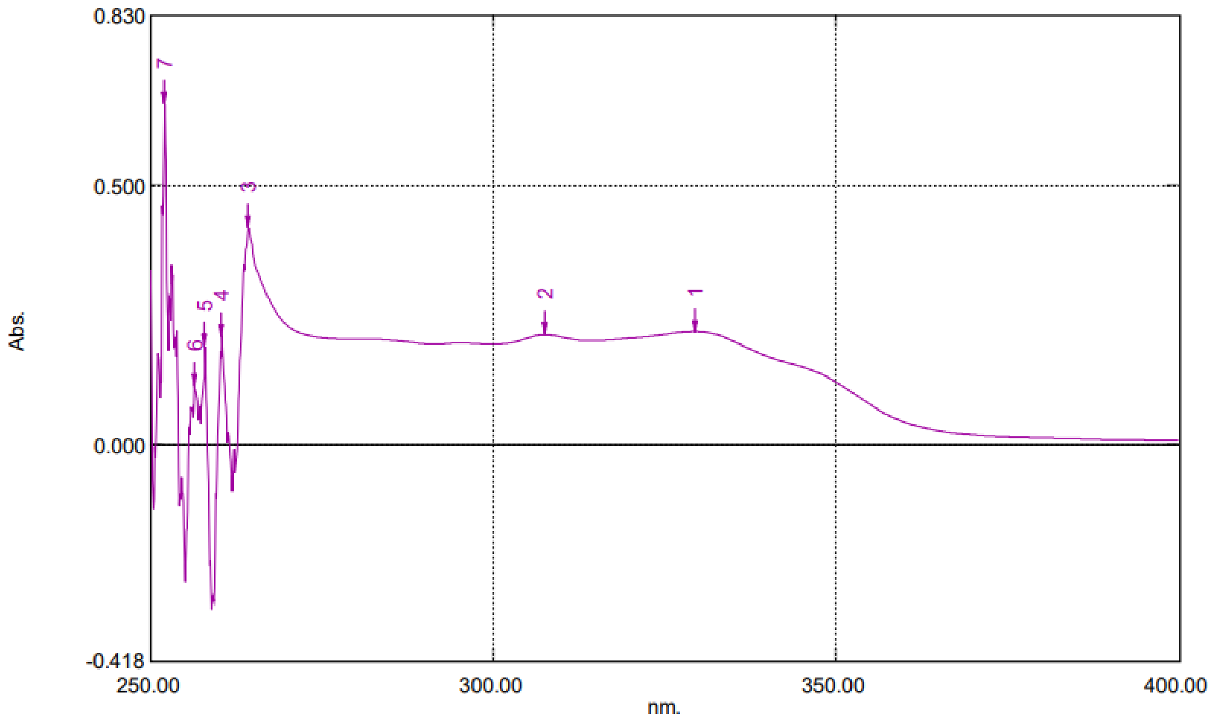

N-(5,6-dimethylbenzo[d]thiazol-2-yl)-2-(5-(4-fluorobenzylidene)-2,4-dioxothiazolidin-3-yl)acetamide (GB25)

1. FTIR

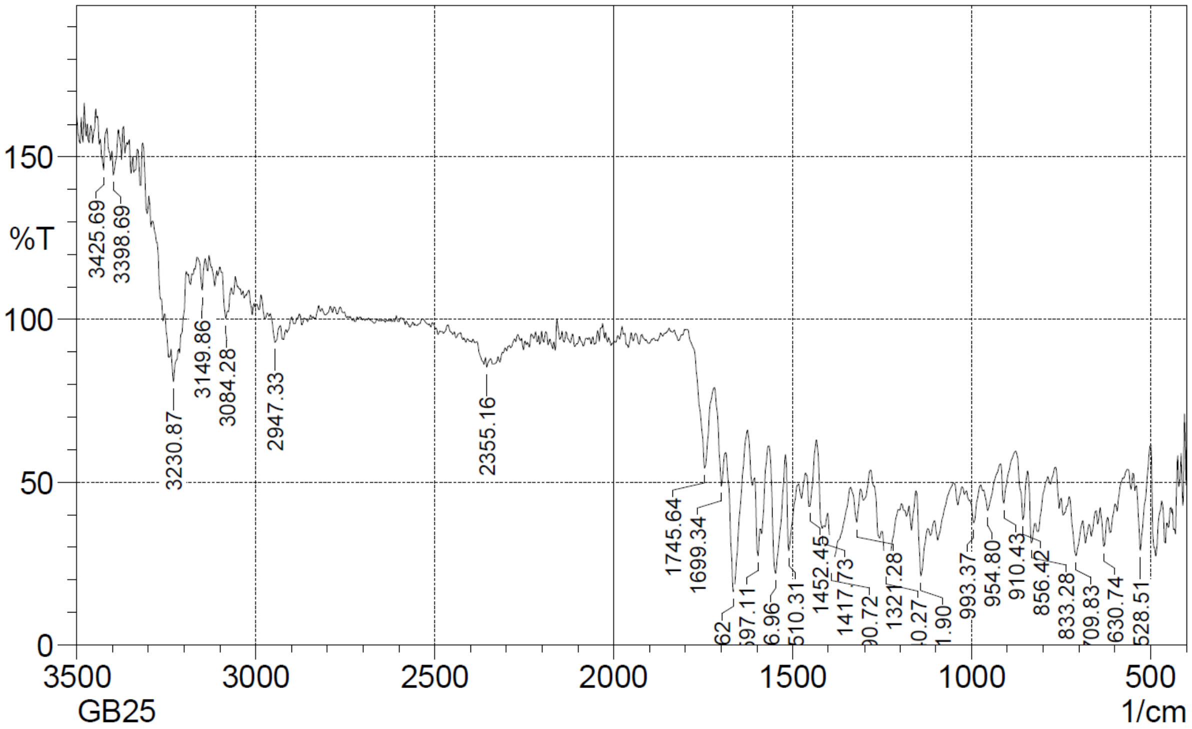

2. ^{1}HNMR

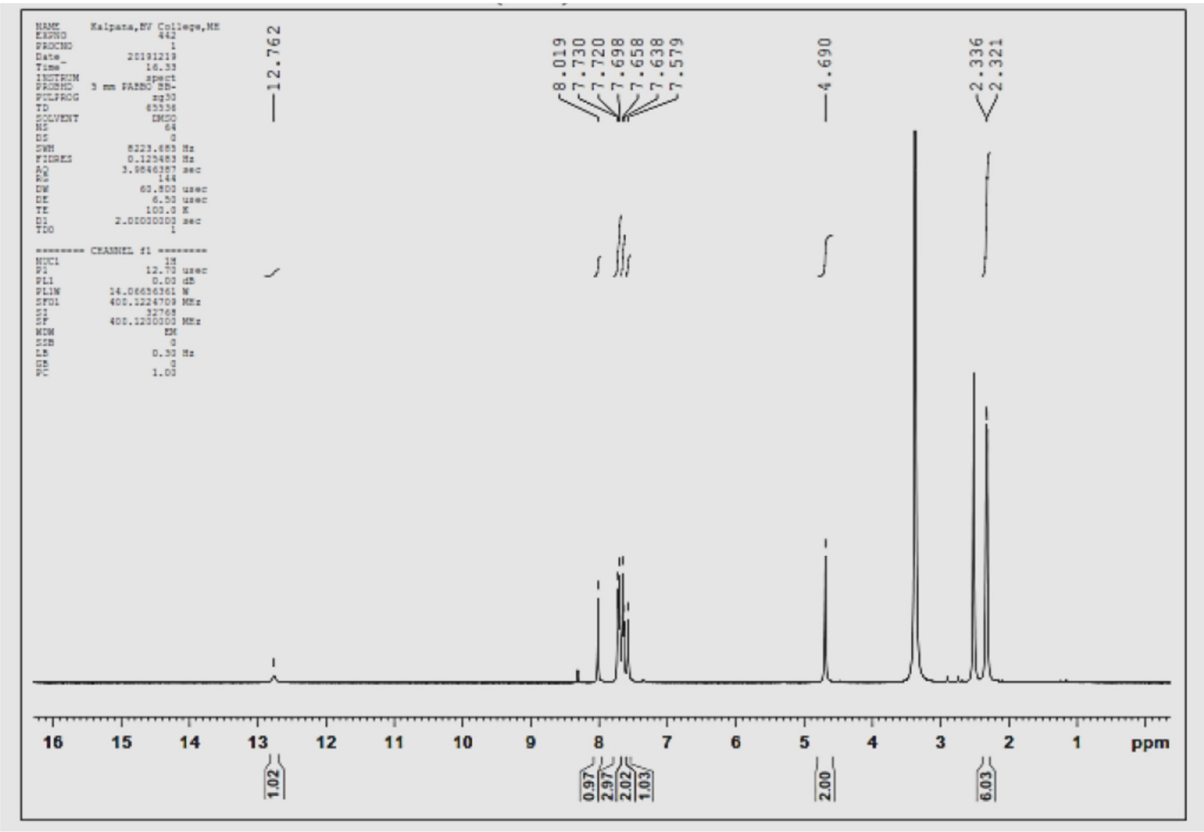
NAME Kalpana,BV College,MH
EXPNO 442
PROCNO 1
Date_ 20191219
Time 16.33
INSTRUM spect
PROBHD 5 mm PABBO BB-
PULPROG zg30
TD 65536
SOLVENT DMSO
NS 64
DS 0
SWH 8223.685 Hz
FIDRES 0.125483 Hz
AQ 3.9846387 sec
RG 144
DW 60.800 usec
DE 6.50 usec
TE 100.0 K
D1 2.00000000 sec
TD0 1
======== CHANNEL f1 ========
NUC1 1H
P1 12.70 usec
PL1 0.00 dB
PL1W 14.06636361 W
SFO1 400.1224709 MHz
SI 32768
SF 400.1200000 MHz
WDW EM
SSB 0
LB 0.30 Hz
GB 0
PC 1.00
12.762
8.019
7.730
7.720
7.698
7.658
7.638
7.579
4.690
2.336
2.321
16 15 14 13 12 11 10 9 8 7 6 5 4 3 2 1 ppm
1.02
0.97
2.97
2.02
1.03
2.00
6.03

3. ^{13}CNMR

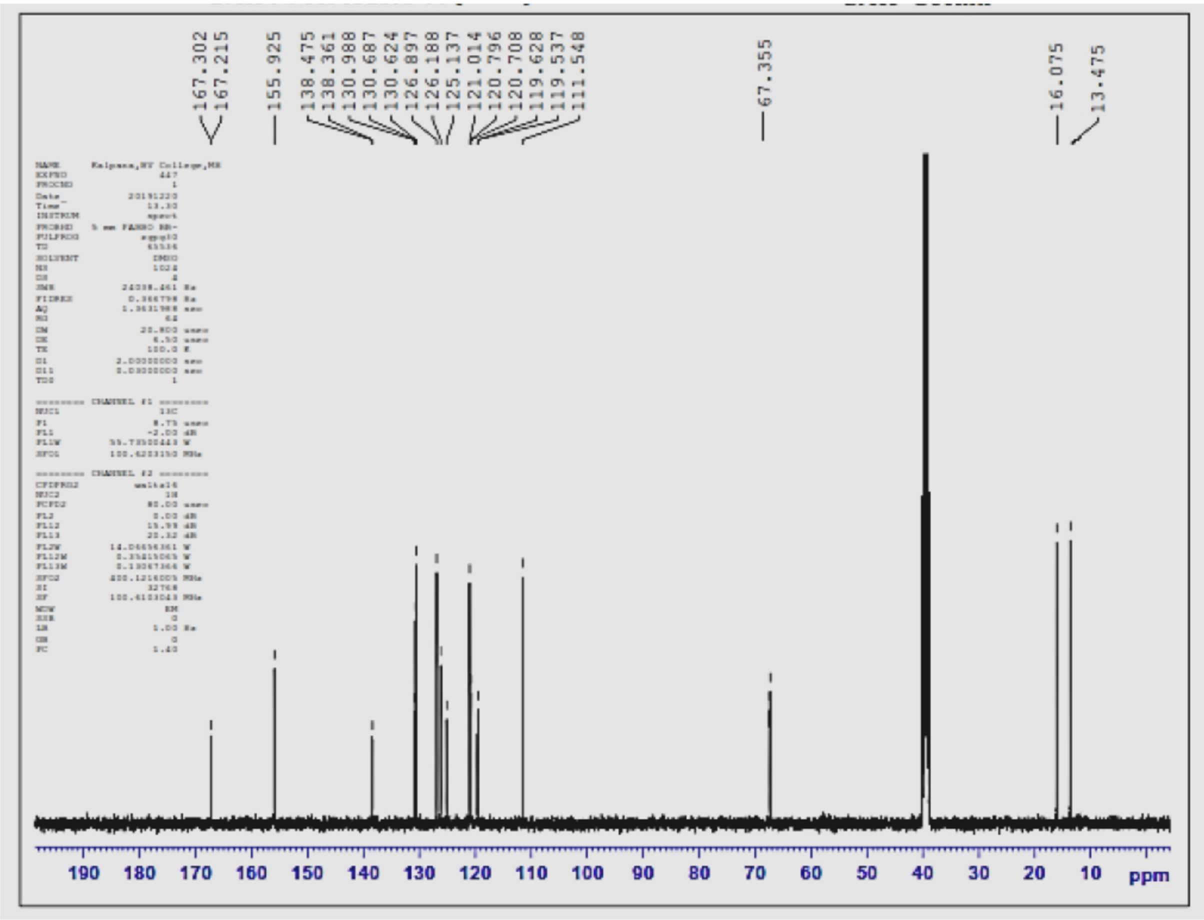
167.302
167.215
155.925
138.475
138.361
130.988
130.687
130.624
126.897
126.188
125.137
121.014
120.796
120.708
119.628
119.537
111.548
67.355
16.075
13.475
190 180 170 160 150 140 130 120 110 100 90 80 70 60 50 40 30 20 10 ppm

4. HPLC

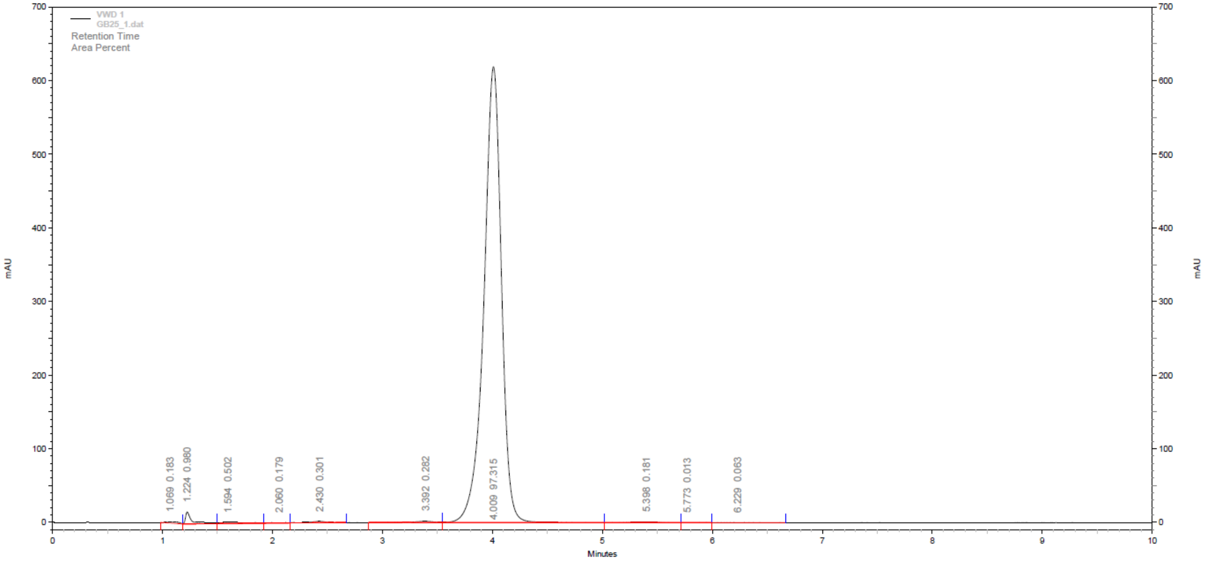
VWD 1
GB25_1.dat
Retention Time
Area Percent
1.069 0.183
1.224 0.980
1.594 0.502
2.060 0.179
2.430 0.301
3.392 0.282
4.009 97.315
5.398 0.181
5.773 0.013
6.229 0.063
mAU
Minutes

5. Mass

MS Spectrum

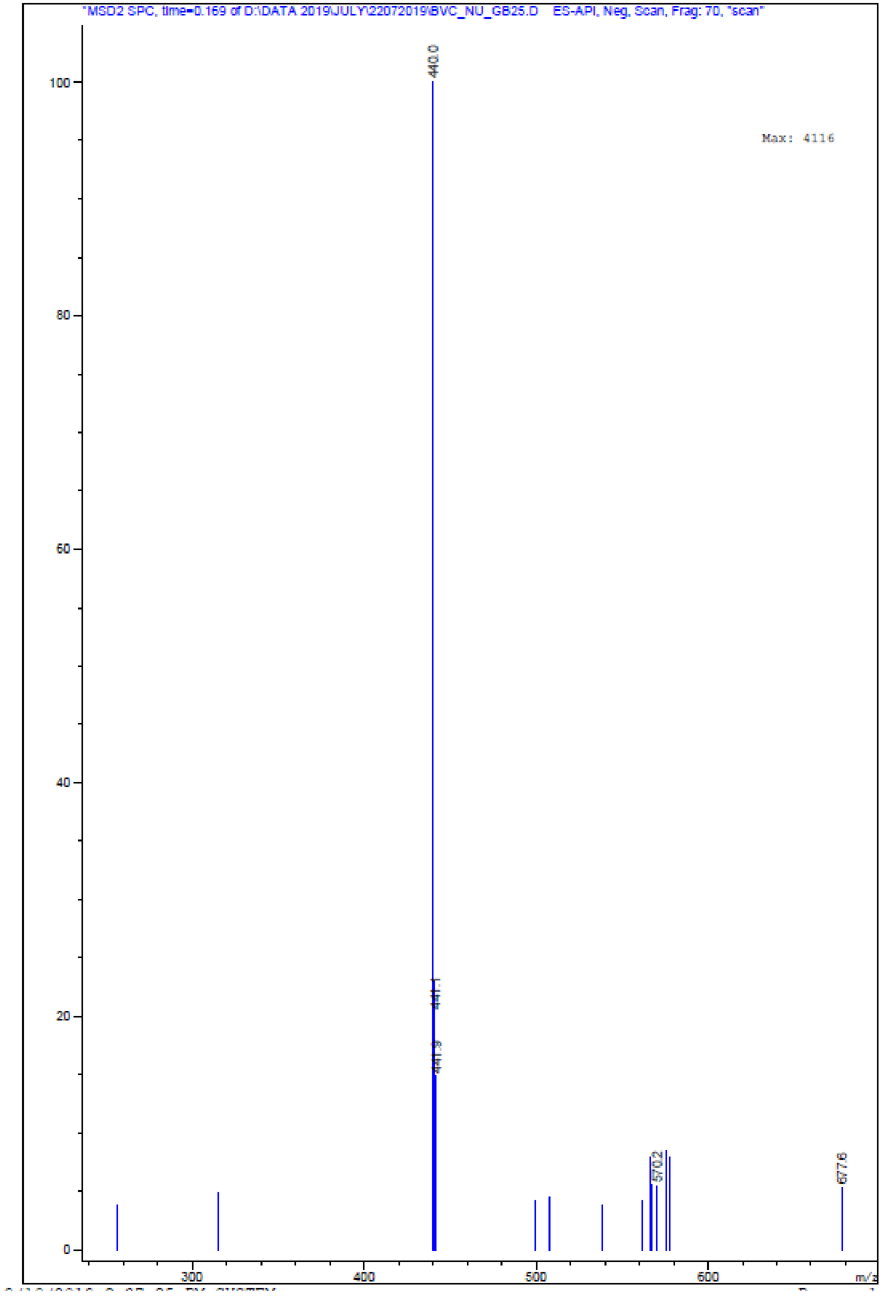
*MSD2 SPC, time=0.169 of D:\DATA 2019\JULY\22072019\BVC_NU_GB25.D ES-API, Neg, Scan, Frag: 70, "scan"
Max: 4116
440.0
441.1
441.9
570.2
677.6
100
80
60
40
20
0
300
400
500
600
m/z

N-(5,6-dimethylbenzo[d]thiazol-2-yl)-2-(5-(3,4-dimethylbenzylidene)-2,4-dioxothiazolidin-3-yl)acetamide (GB26)

1. FTIR

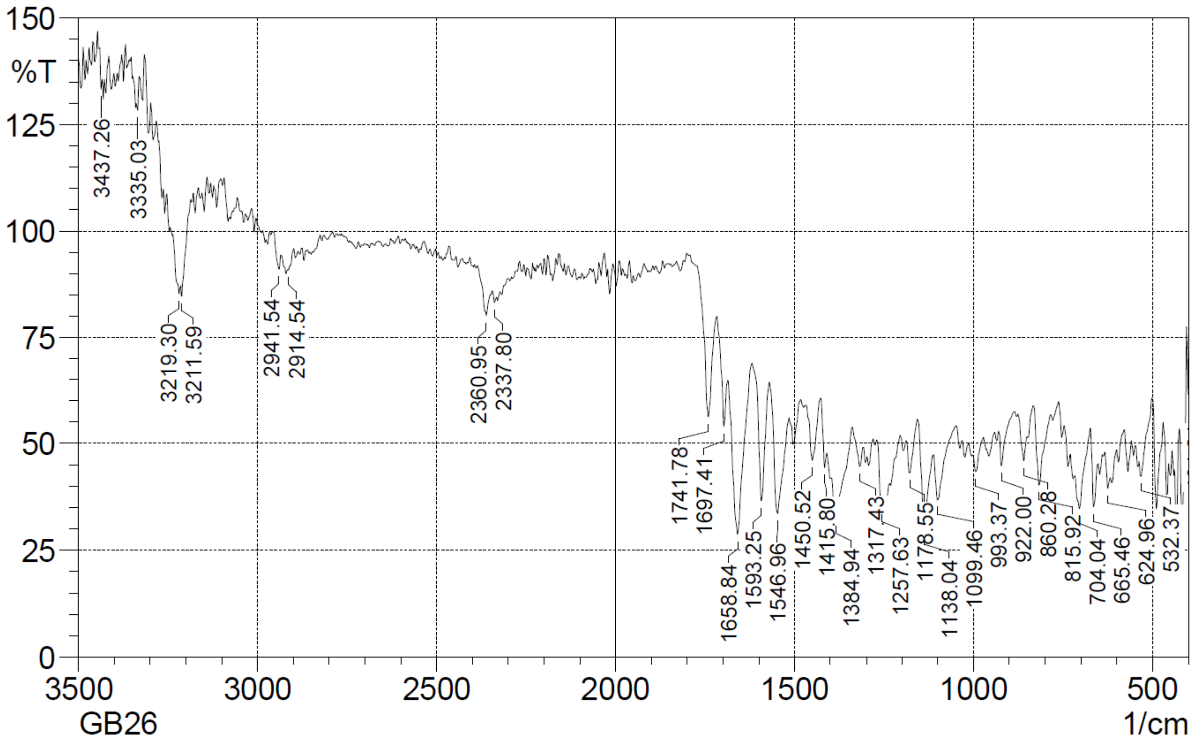

2. 1H-NMR

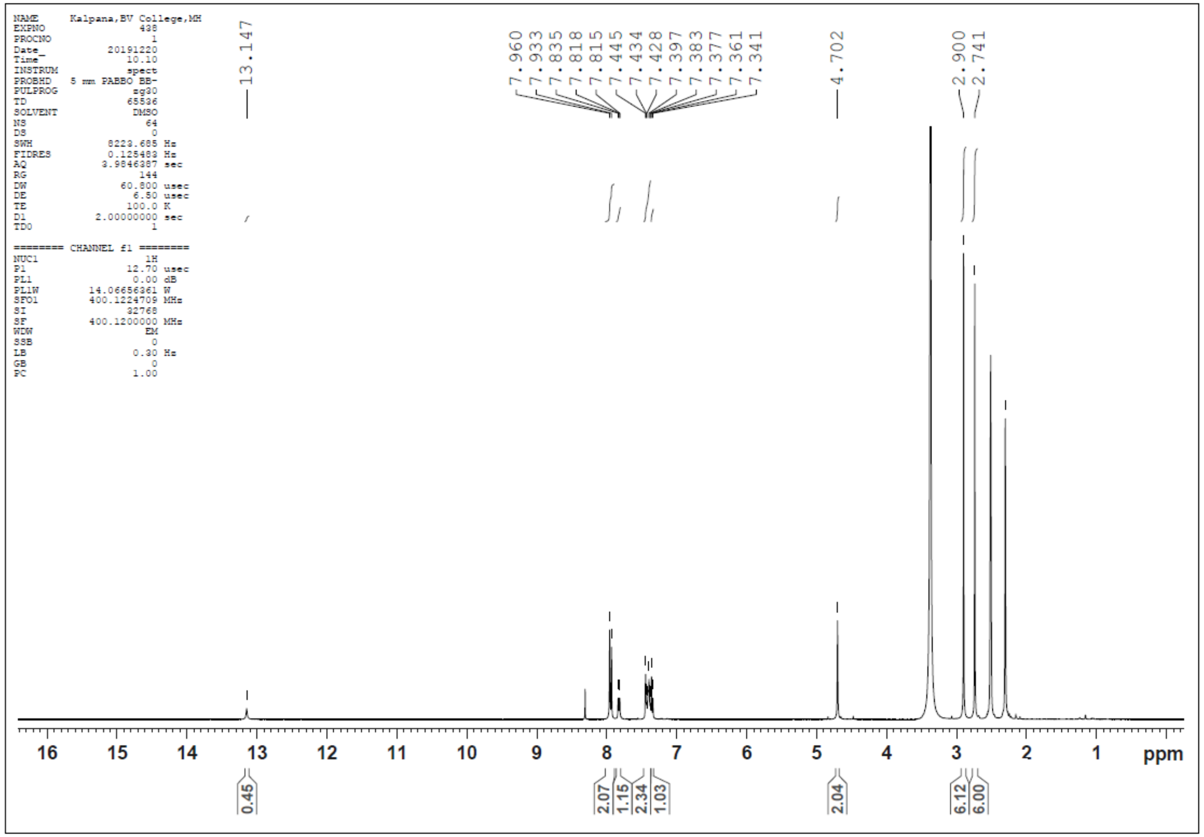

NAME Kalpana,BV College,MH
EXPNO 438
PROCNO 1
Date_ 20191220
Time 10.10
INSTRUM spect
PROBHD 5 mm PABBO BB-
PULPROG zg30
TD 65536
SOLVENT DMSO
NS 64
DS 0
SWH 8223.685 Hz
FIDRES 0.125483 Hz
AQ 3.9846387 sec
RG 144
DW 60.800 usec
DE 6.50 usec
TE 100.0 K
D1 2.00000000 sec
TD0 1
======== CHANNEL f1 ========
NUC1 1H
P1 12.70 usec
PL1 0.00 dB
PL1W 14.06656361 W
SFO1 400.1224709 MHz
SI 32768
SF 400.1200000 MHz
WDW EM
SSB 0
LB 0.30 Hz
GB 0
PC 1.00
13.147
7.960
7.933
7.835
7.818
7.815
7.445
7.434
7.428
7.397
7.383
7.377
7.361
7.341
4.702
2.900
2.741
ppm
0.45
2.07
1.15
2.34
1.03
2.04
6.12
6.00

3. 13C-NMR

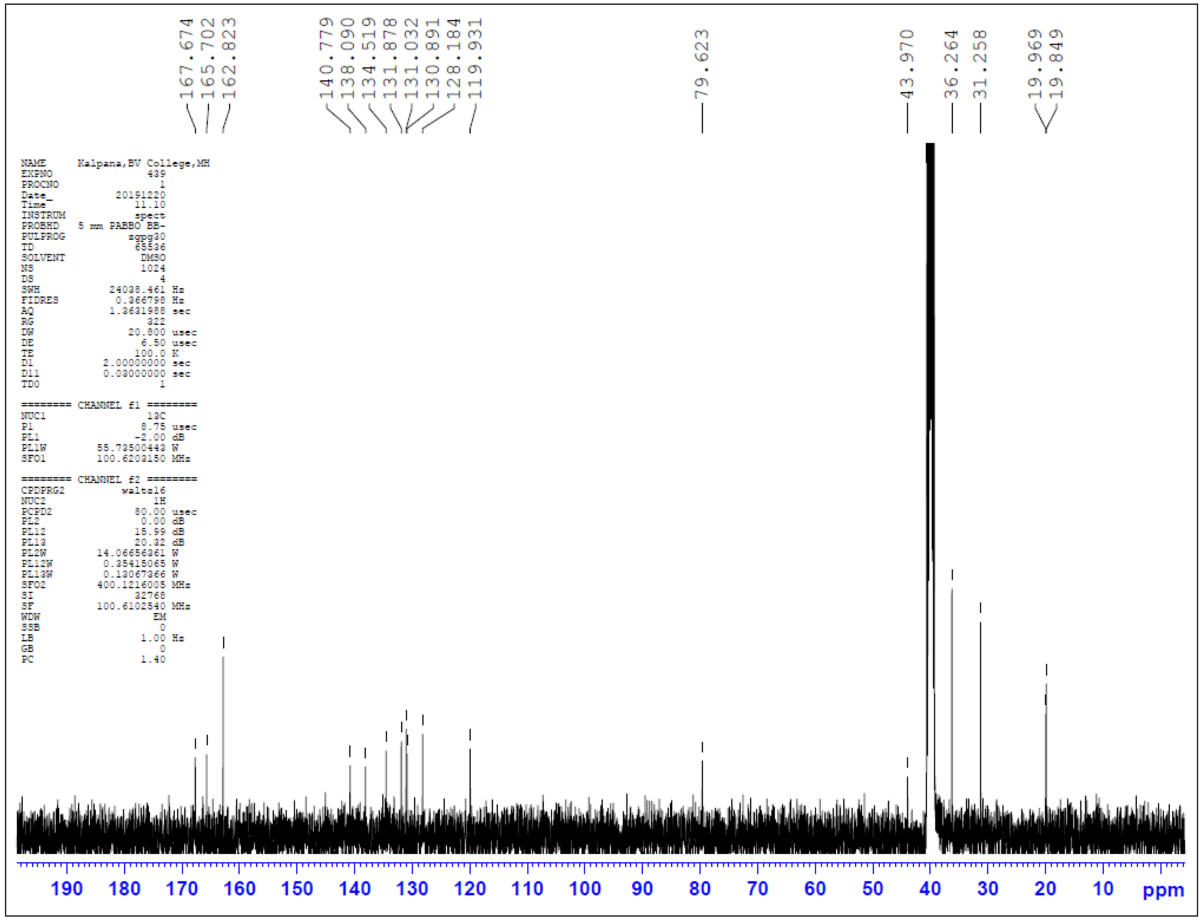
167.674
165.702
162.823
140.779
138.090
134.519
131.878
131.032
130.891
128.184
119.931
79.623
43.970
36.264
31.258
19.969
19.849
NAME Kalpana,BV College,MH
EXPNO 439
PROCNO 1
Date_ 20191220
Time 11.10
INSTRUM spect
PROBHD 5 mm PABBO BB-
PULPROG zgpg30
TD 65536
SOLVENT DMSO
NS 1024
DS 4
SWH 24038.461 Hz
FIDRES 0.366798 Hz
AQ 1.3631988 sec
RG 322
DW 20.800 usec
DE 6.50 usec
TE 100.0 K
D1 2.00000000 sec
D11 0.03000000 sec
TD0 1
======== CHANNEL f1 ========
NUC1 13C
P1 8.75 usec
PL1 -2.00 dB
PL1W 55.73500443 W
SFO1 100.6203150 MHz
======== CHANNEL f2 ========
CPDPRG2 waltz16
NUC2 1H
PCPD2 80.00 usec
PL2 0.00 dB
PL12 15.99 dB
PL13 20.32 dB
PL2W 14.06656361 W
PL12W 0.35415065 W
PL13W 0.13067366 W
SFO2 400.1216005 MHz
SI 32768
SF 100.6102540 MHz
WDW EM
SSB 0
LB 1.00 Hz
GB 0
PC 1.40
190 180 170 160 150 140 130 120 110 100 90 80 70 60 50 40 30 20 10 ppm

4. HPLC

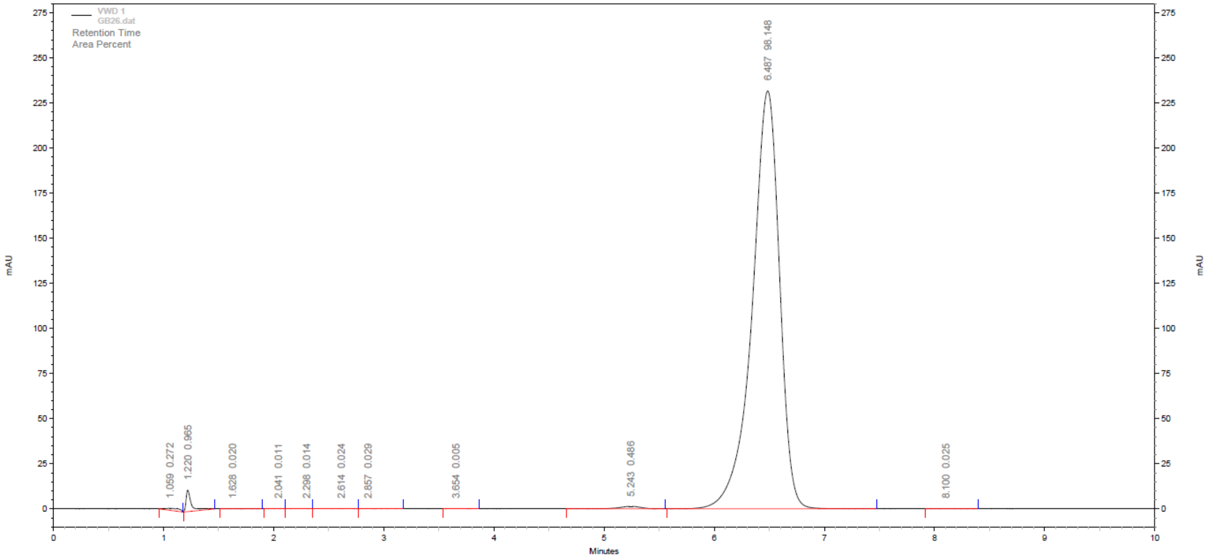
VWD 1
GB26.dat
Retention Time
Area Percent
1.059 0.272
1.220 0.965
1.628 0.020
2.041 0.011
2.298 0.014
2.614 0.024
2.857 0.029
3.654 0.005
5.243 0.486
6.487 98.148
8.100 0.025
mAU
Minutes

5. Mass

MS Spectrum

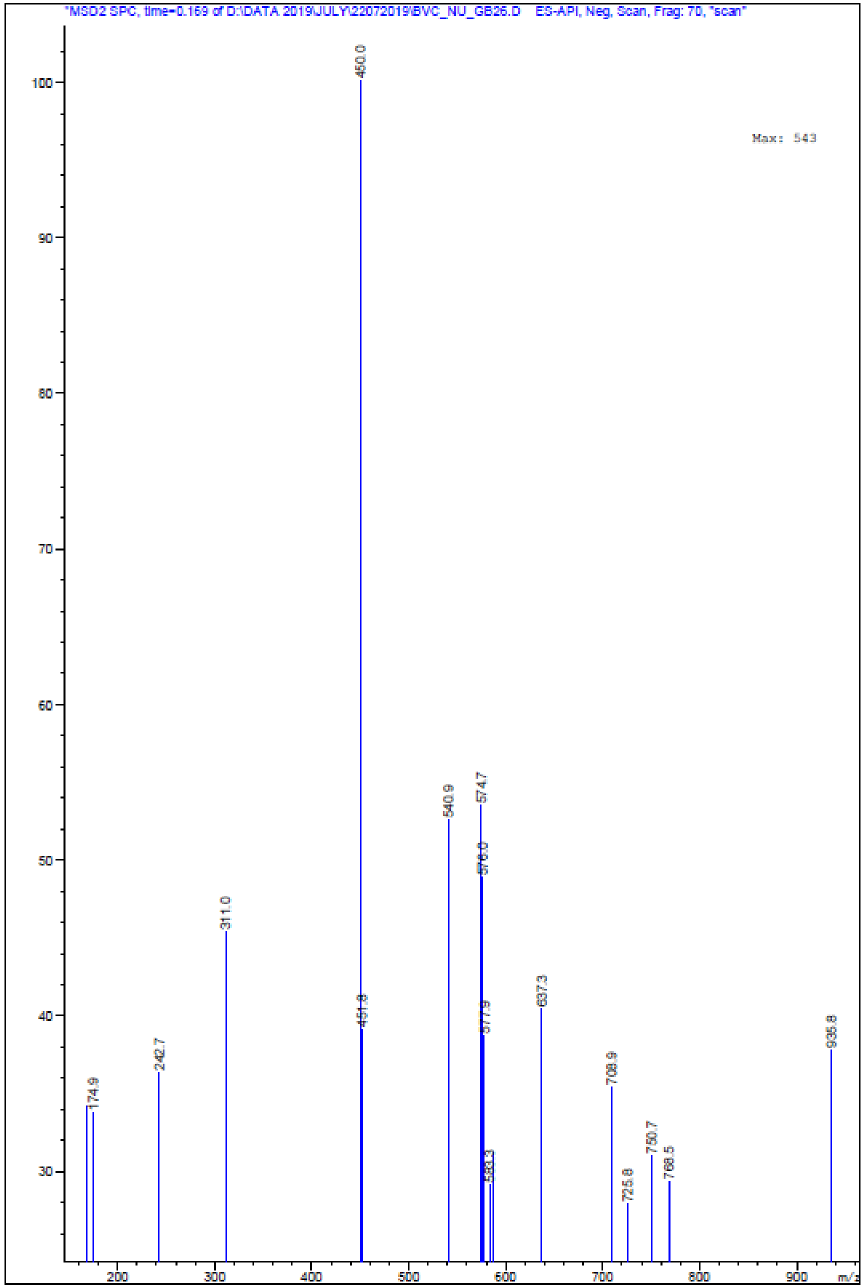
*MSD2 SPC, time=0.169 of D:\DATA 2019\JULY\22072019\BVC_NU_GB26.D ES-API, Neg, Scan, Frag: 70, "scan"
Max: 543
450.0
540.9
574.7
576.0
311.0
451.8
577.9
637.3
242.7
174.9
708.9
935.8
750.7
768.5
725.8
583.3
m/z

N-(4-methoxybenzo[d]thiazol-2-yl)-2-(5-(4-methylbenzylidene)-2,4-dioxothiazolidin-3-yl)acetamide (GB27)

1. FTIR

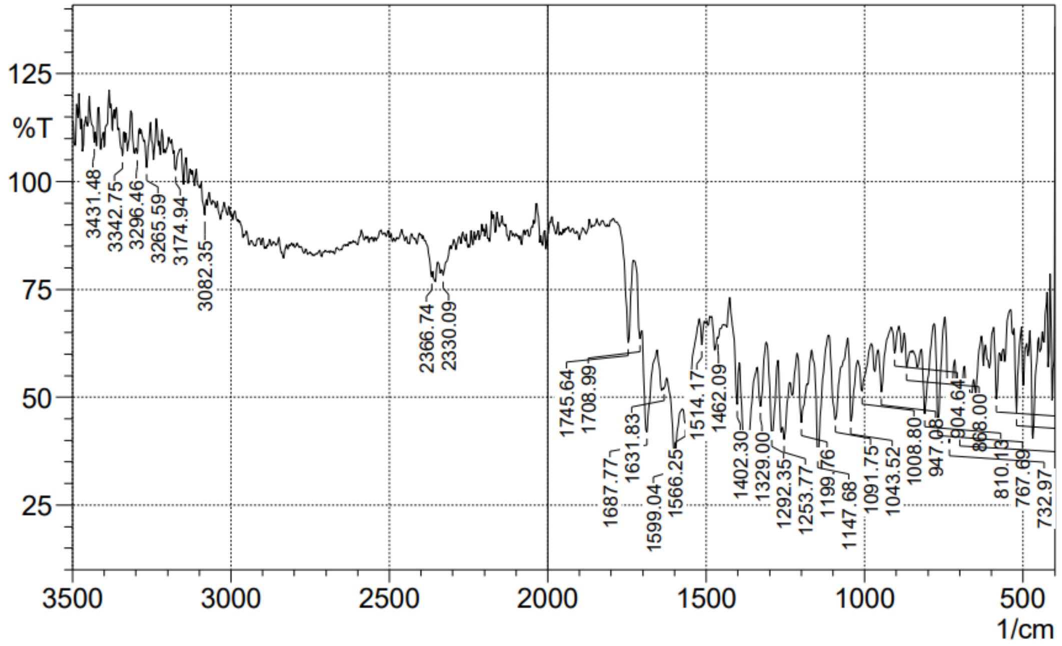

2. ^{I}H-NMR

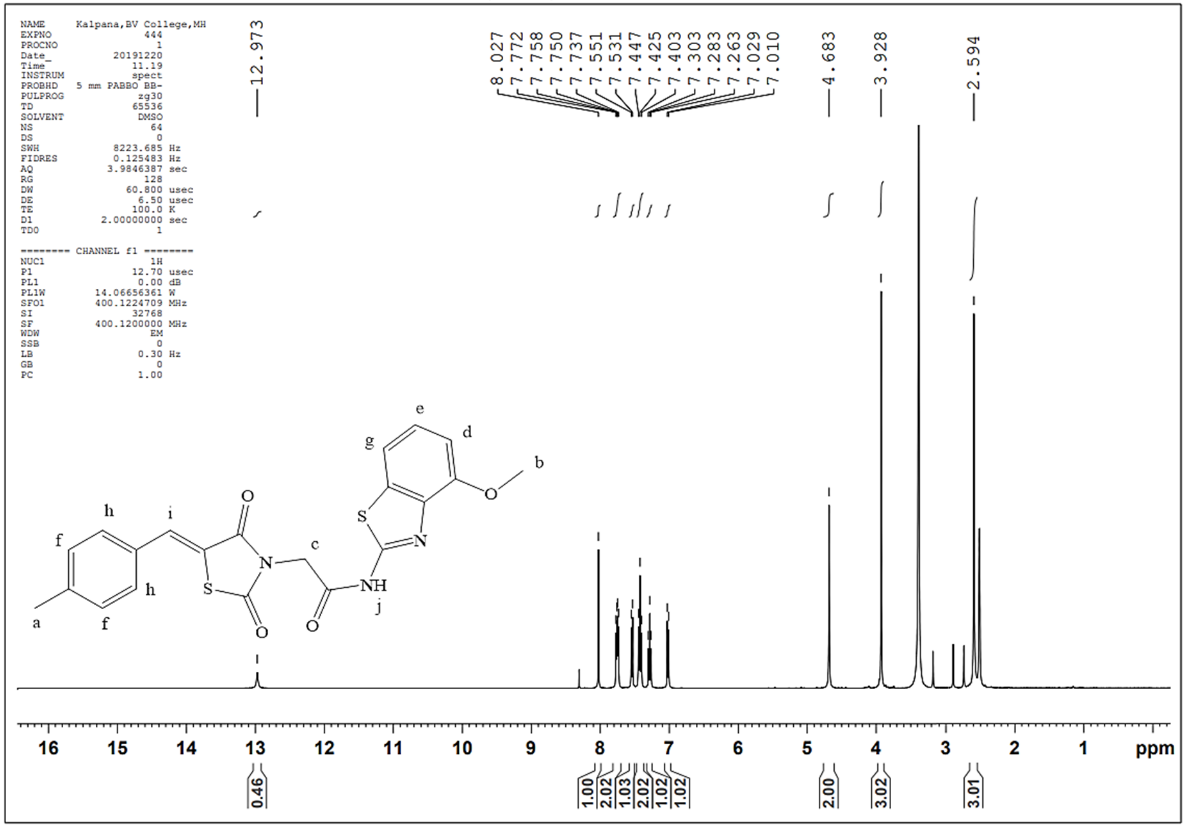
NAME Kalpana,BV College,MH
EXPNO 444
PROCNO 1
Date_ 20191220
Time 11.19
INSTRUM spect
PROBHD 5 mm PABBO BB-
PULPROG zg30
TD 65536
SOLVENT DMSO
NS 64
DS 0
SWH 8223.685 Hz
FIDRES 0.125483 Hz
AQ 3.9846387 sec
RG 128
DW 60.800 usec
DE 6.50 usec
TE 100.0 K
D1 2.00000000 sec
TD0 1
======== CHANNEL f1 ========
NUC1 1H
P1 12.70 usec
PL1 0.00 dB
PL1W 14.06656361 W
SFO1 400.1224709 MHz
SI 32768
SF 400.1200000 MHz
WDW EM
SSB 0
LB 0.30 Hz
GB 0
PC 1.00
12.973
8.027
7.772
7.758
7.750
7.737
7.551
7.531
7.447
7.425
7.403
7.303
7.283
7.263
7.029
7.010
4.683
3.928
2.594
ppm
0.46
1.00
2.02
1.03
2.02
1.02
1.02
2.00
3.02
3.01

3. ^{13}C-NMR

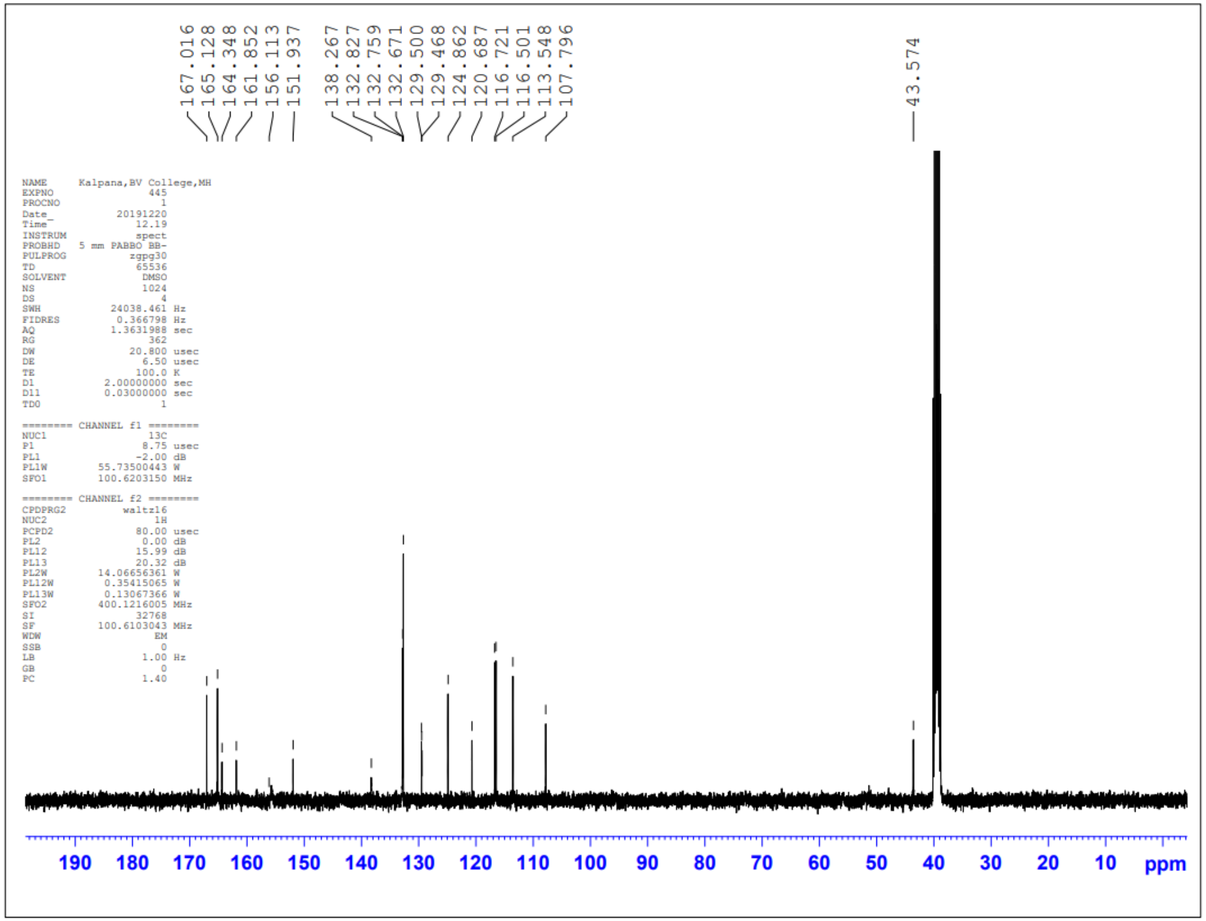

4. HPLC Analysis

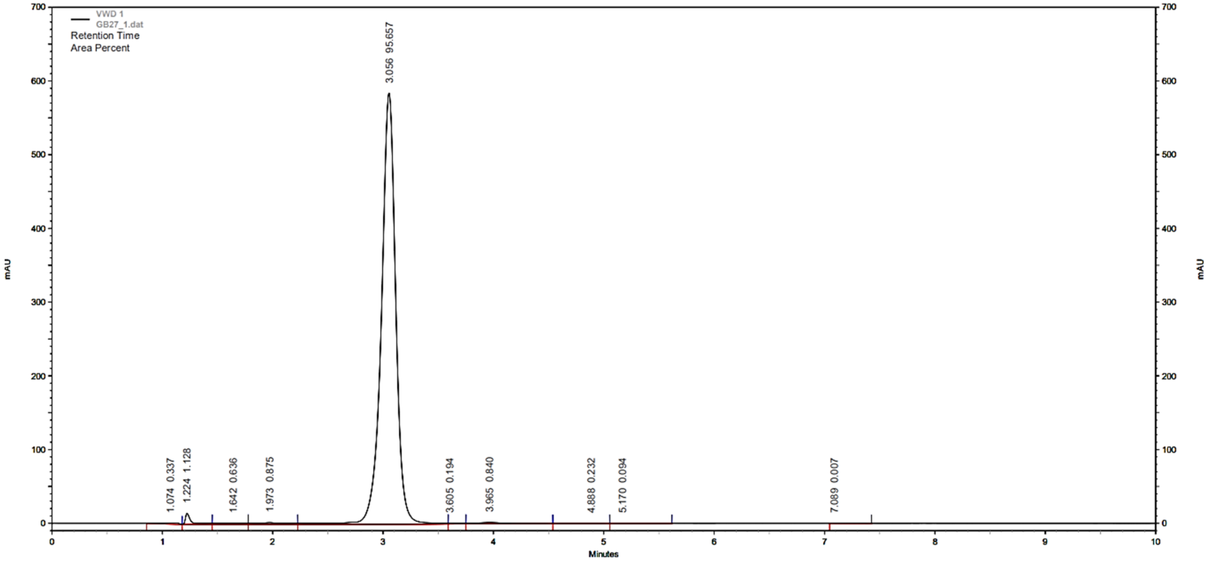

5. Mass Spectrometry

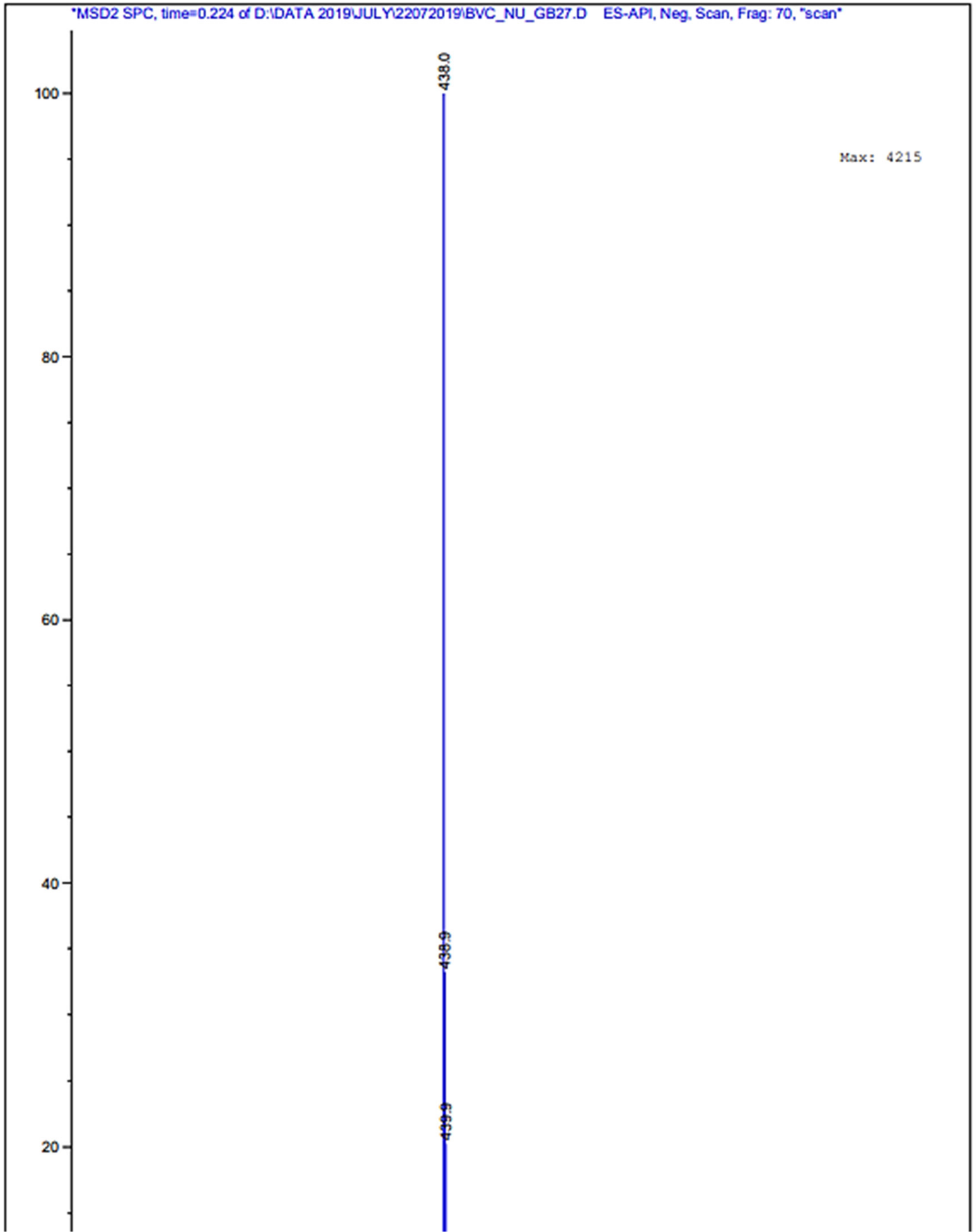
*MSD2 SPC, time=0.224 of D:\DATA 2019\JULY\22072019\BVC_NU_GB27.D ES-API, Neg, Scan, Frag: 70, "scan"
Max: 4215
438.0
438.9
439.9
100
80
60
40
20

6. UV

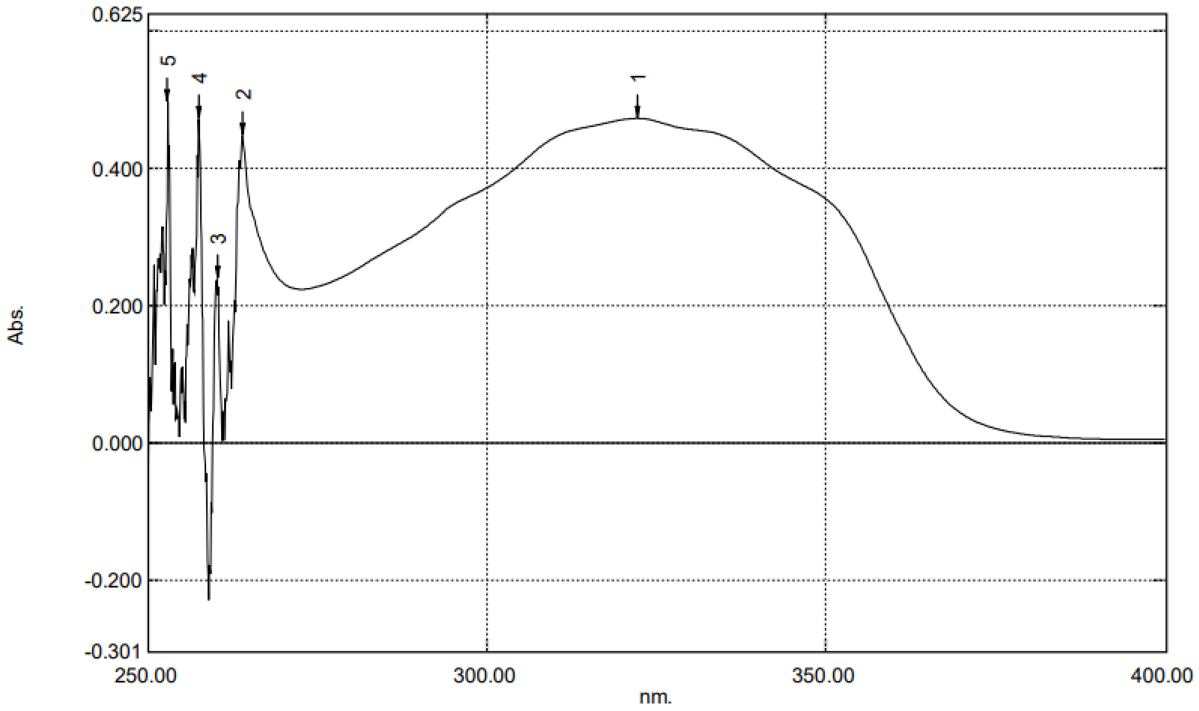

0.625
0.400
0.200
0.000
-0.200
-0.301
Abs.
250.00
300.00
350.00
400.00
nm.
1
2
3
4
5

2-(5-benzylidene-2,4-dioxothiazolidin-3-yl)-N-(4-methylbenzo[d]thiazol-2-yl)acetamide (GB28)

1. FTIR

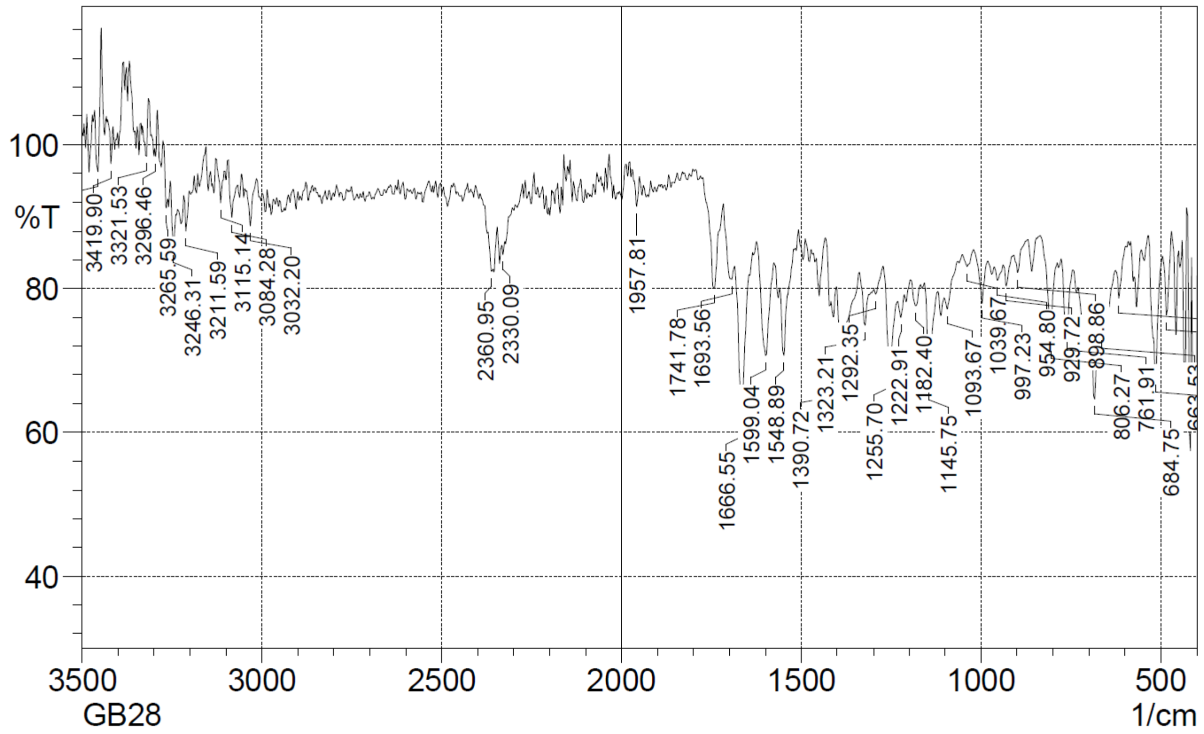

2. ¹H-NMR

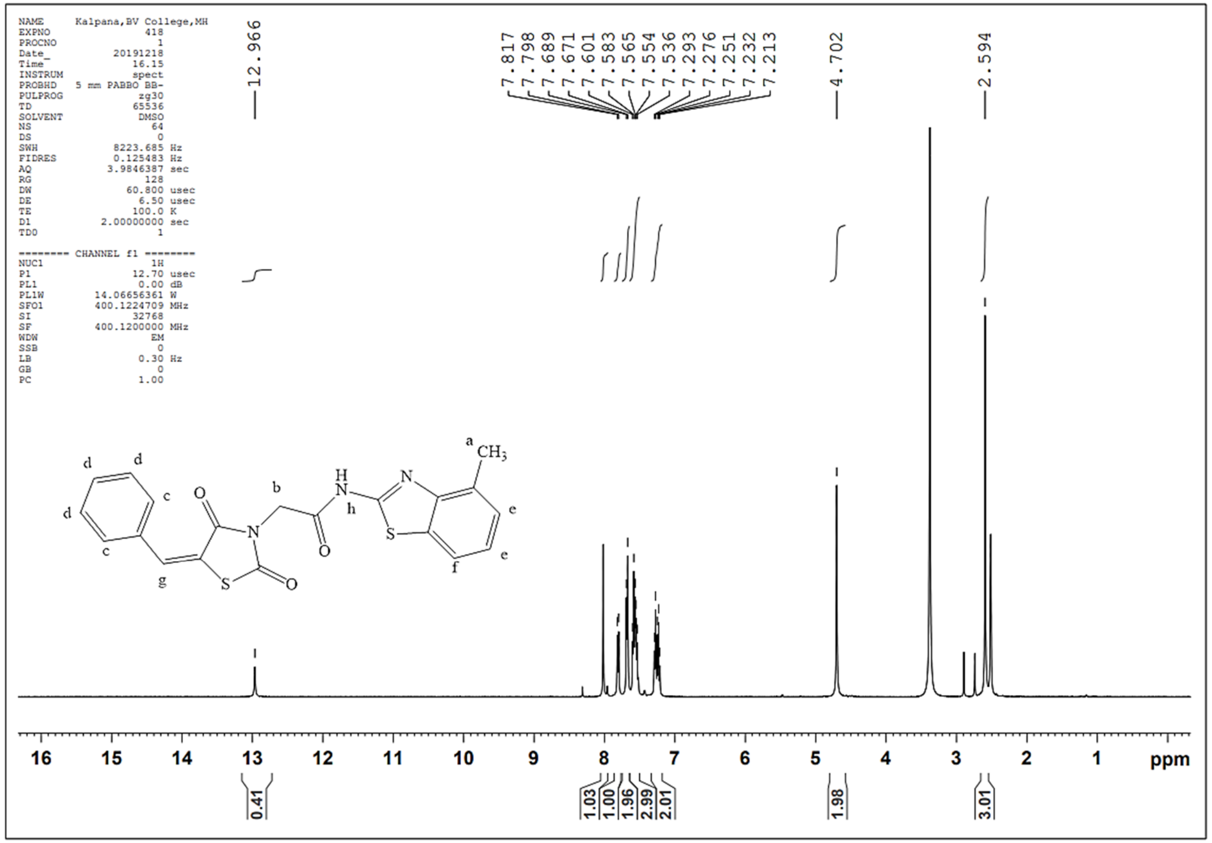

NAME Kalpana,BV College,MH
EXPNO 418
PROCNO 1
Date_ 20191218
Time 16.15
INSTRUM spect
PROBHD 5 mm PABBO BB-
PULPROG zg30
TD 65536
SOLVENT DMSO
NS 64
DS 0
SWH 8223.685 Hz
FIDRES 0.125483 Hz
AQ 3.9846387 sec
RG 128
DW 60.800 usec
DE 6.50 usec
TE 100.0 K
D1 2.00000000 sec
TD0 1
======== CHANNEL f1 ========
NUC1 1H
P1 12.70 usec
PL1 0.00 dB
PL1W 14.06656361 W
SFO1 400.1224709 MHz
SI 32768
SF 400.1200000 MHz
WDW EM
SSB 0
LB 0.30 Hz
GB 0
PC 1.00
12.966
7.817
7.798
7.689
7.671
7.601
7.583
7.565
7.554
7.536
7.293
7.276
7.251
7.232
7.213
4.702
2.594
0.41
1.03
1.00
1.96
2.99
2.01
1.98
3.01
ppm

3. ^{13}C-NMR

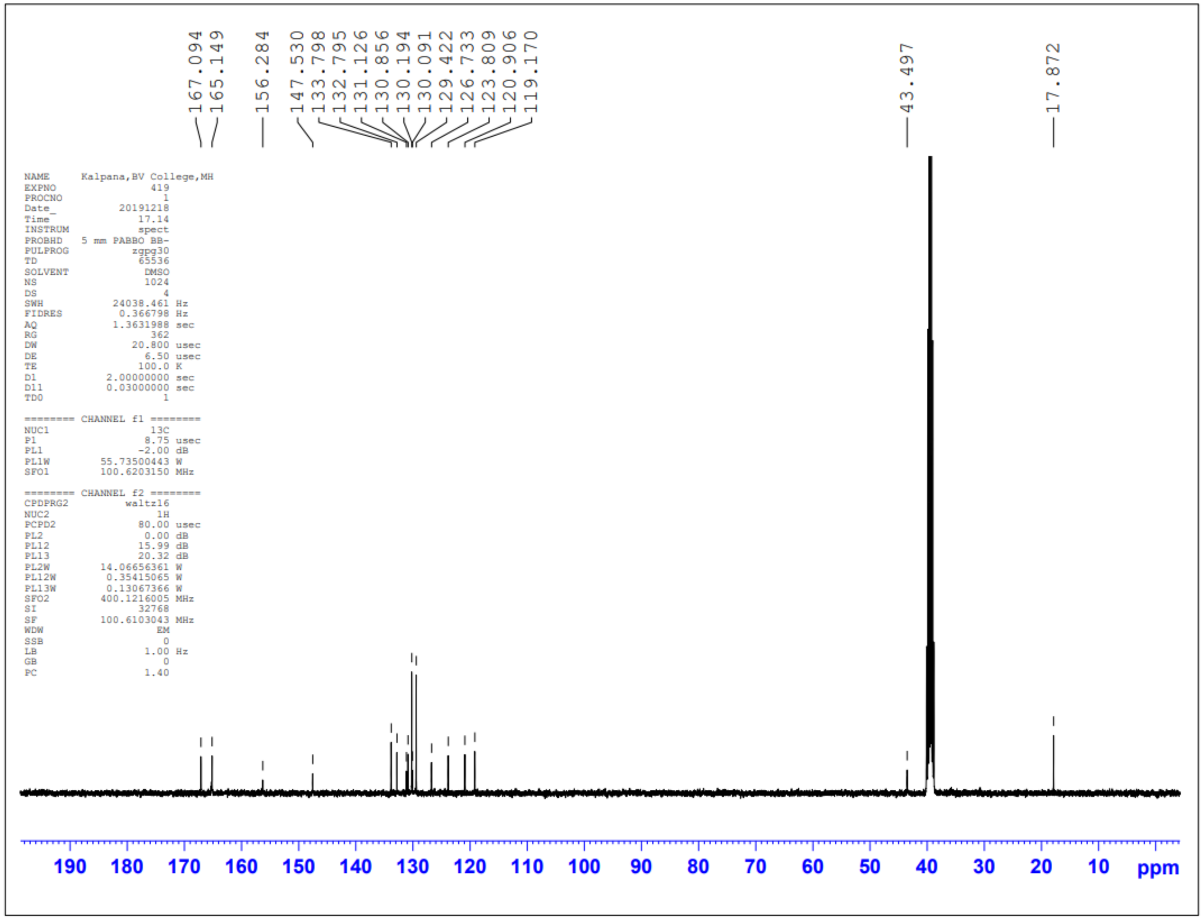
167.094
165.149
156.284
147.530
133.798
132.795
131.126
130.856
130.194
130.091
129.422
126.733
123.809
120.906
119.170
43.497
17.872
NAME Kalpana,BV College,MH
EXPNO 419
PROCNO 1
Date_ 20191218
Time 17.14
INSTRUM spect
PROBHD 5 mm PABBO BB-
PULPROG zgpg30
TD 65536
SOLVENT DMSO
NS 1024
DS 4
SWH 24038.461 Hz
FIDRES 0.366798 Hz
AQ 1.3631988 sec
RG 362
DW 20.800 usec
DE 6.50 usec
TE 100.0 K
D1 2.00000000 sec
D11 0.03000000 sec
TD0 1
======== CHANNEL f1 ========
NUC1 13C
P1 8.75 usec
PL1 -2.00 dB
PL1W 55.73500443 W
SFO1 100.6203150 MHz
======== CHANNEL f2 ========
CPDPRG2 waltz16
NUC2 1H
PCPD2 80.00 usec
PL2 0.00 dB
PL12 15.99 dB
PL13 20.32 dB
PL2W 14.06656361 W
PL12W 0.35415065 W
PL13W 0.13067366 W
SFO2 400.1216005 MHz
SI 32768
SF 100.6103043 MHz
WDW EM
SSB 0
LB 1.00 Hz
GB 0
PC 1.40
190 180 170 160 150 140 130 120 110 100 90 80 70 60 50 40 30 20 10 ppm

4. HPLC

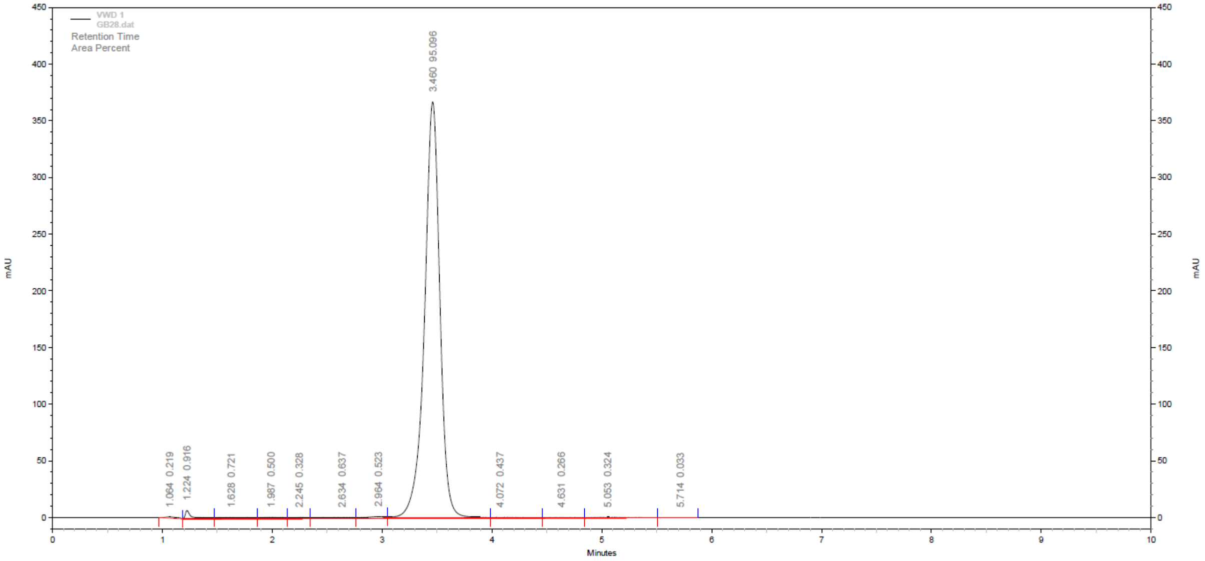
VWD 1
GB28.dat
Retention Time
Area Percent
3.460 95.096
1.064 0.219
1.224 0.916
1.628 0.721
1.987 0.500
2.245 0.328
2.634 0.637
2.964 0.523
4.072 0.437
4.631 0.266
5.053 0.324
5.714 0.033
mAU
Minutes

5. Mass

MS Spectrum

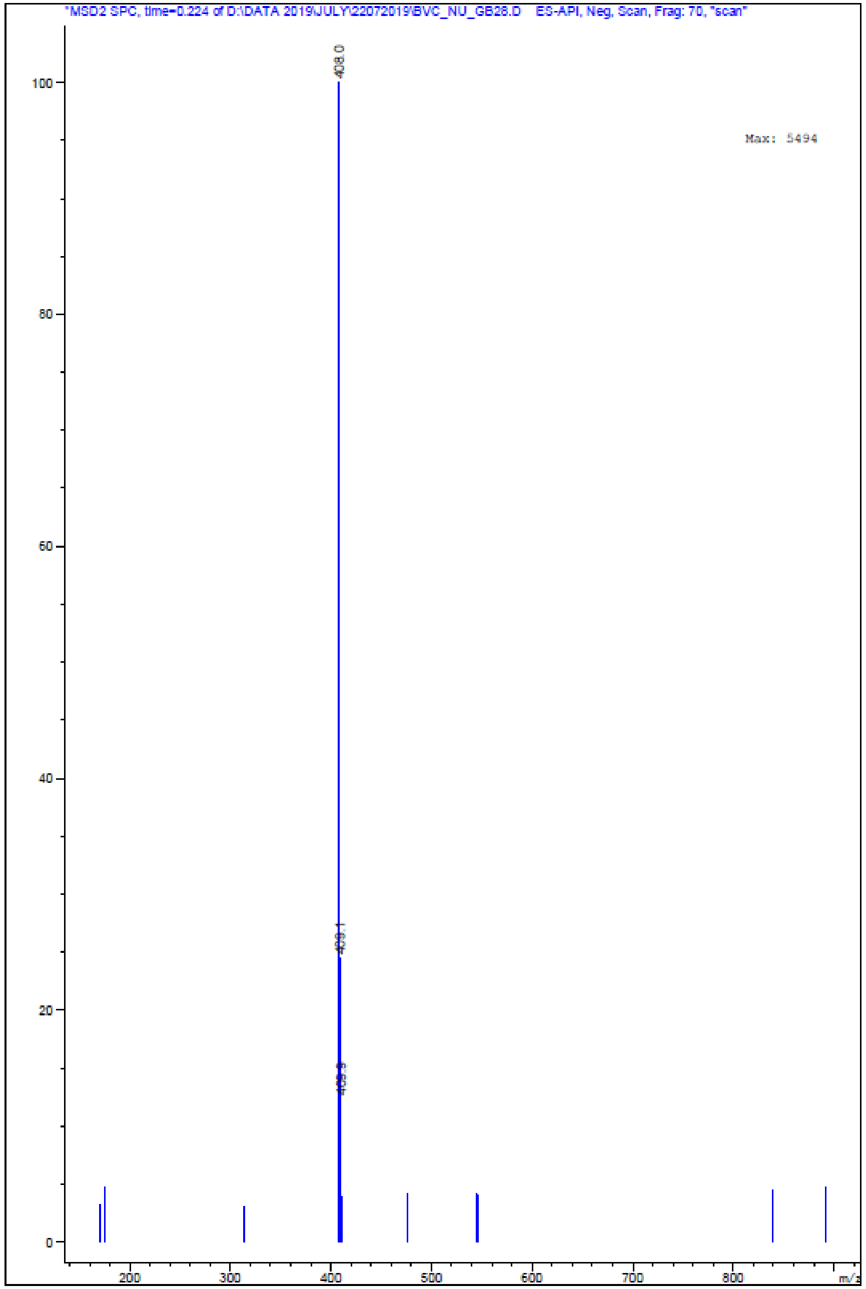

"MSD2 SPC, time=0.224 of D:\DATA 2019\JULY\22072019\BVC_NU_GB28.D ES-API, Neg, Scan, Frag: 70, "scan"
408.0
Max: 5494
100
80
60
40
20
0
200
300
400
500
600
700
800

N-(4,6-difluorobenzo[d]thiazol-2-yl)-2-(5-(2,4-difluorobenzylidene)-2,4-dioxothiazolidin-3-yl)acetamide (GB29)

1. FTIR

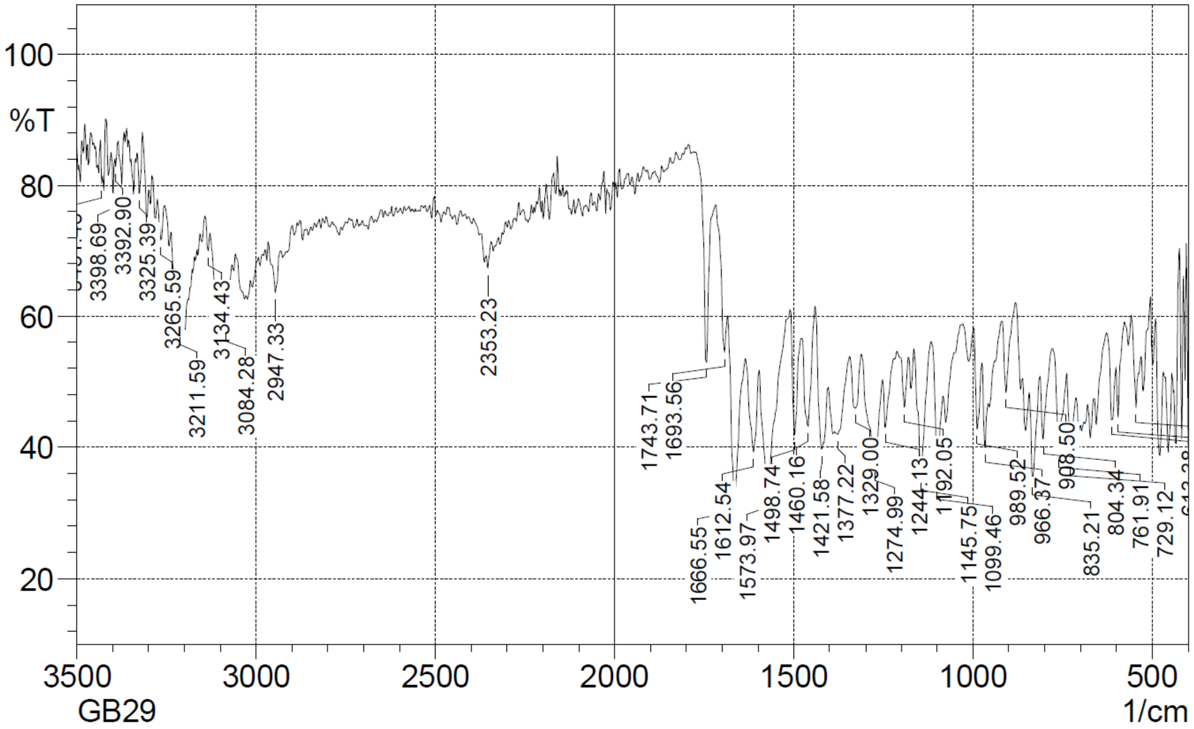

2. ^{1}HNMR

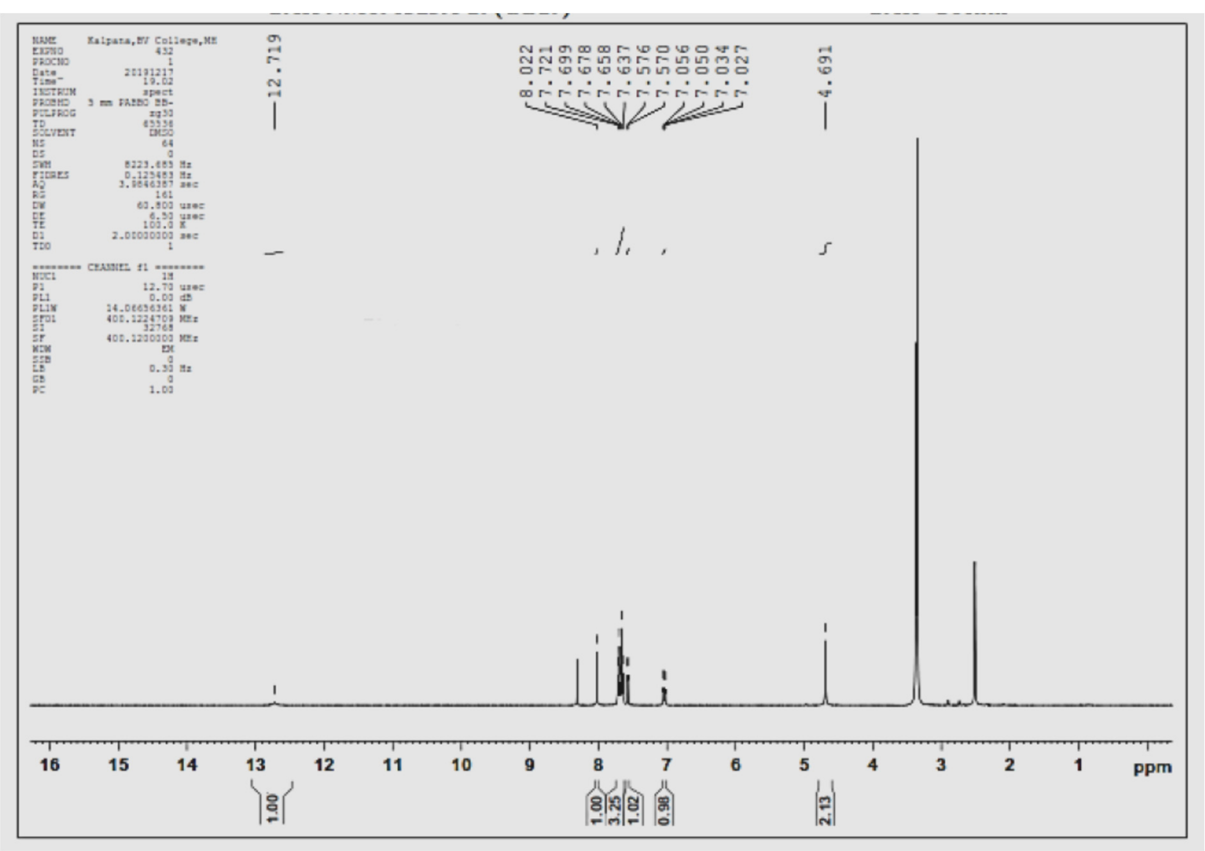
12.719
8.022
7.721
7.699
7.678
7.658
7.637
7.576
7.570
7.056
7.050
7.034
7.027
4.691
ppm
1.00
1.00
3.25
1.02
0.98
2.13

3. ^{13}CNMR

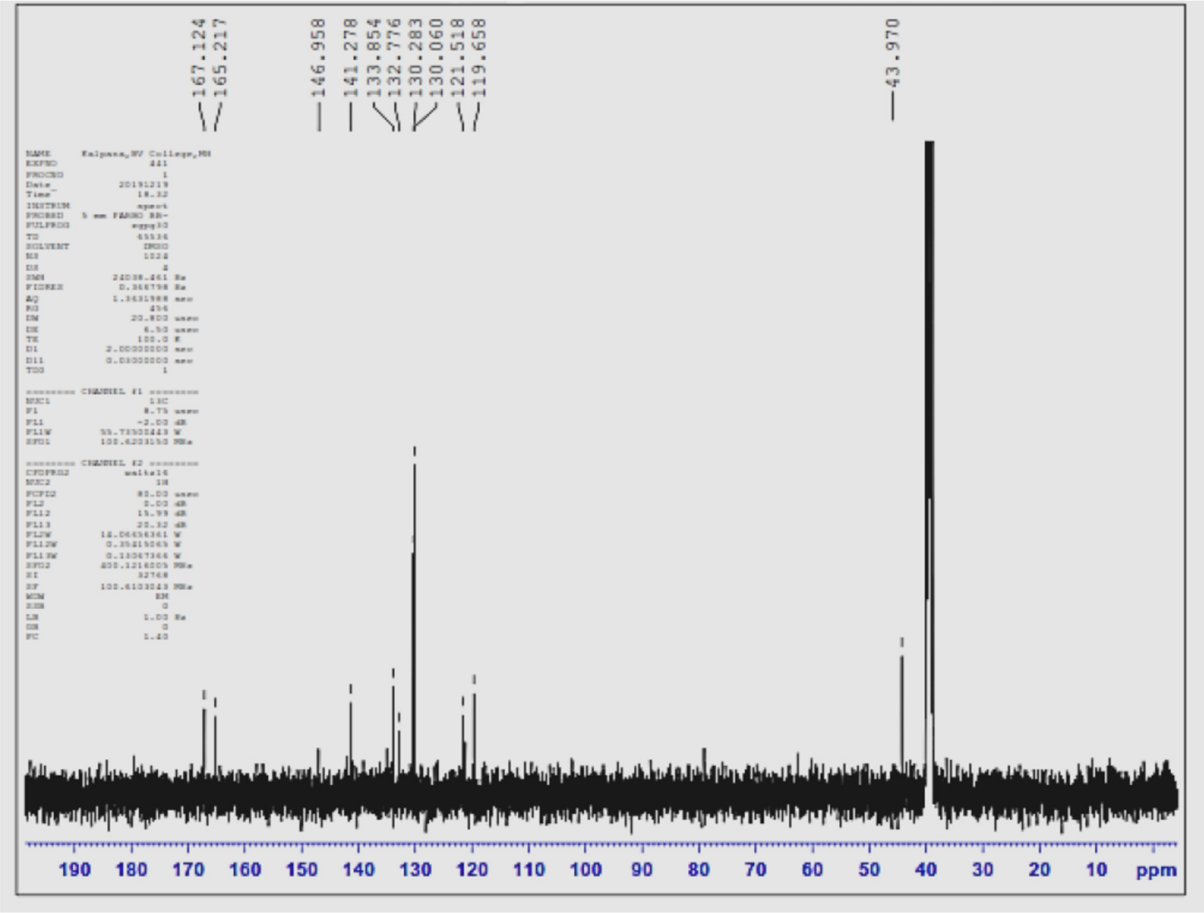
167.124
165.217
146.958
141.278
133.854
132.776
130.283
130.060
121.518
119.658
43.970
190 180 170 160 150 140 130 120 110 100 90 80 70 60 50 40 30 20 10 ppm

4. HPLC

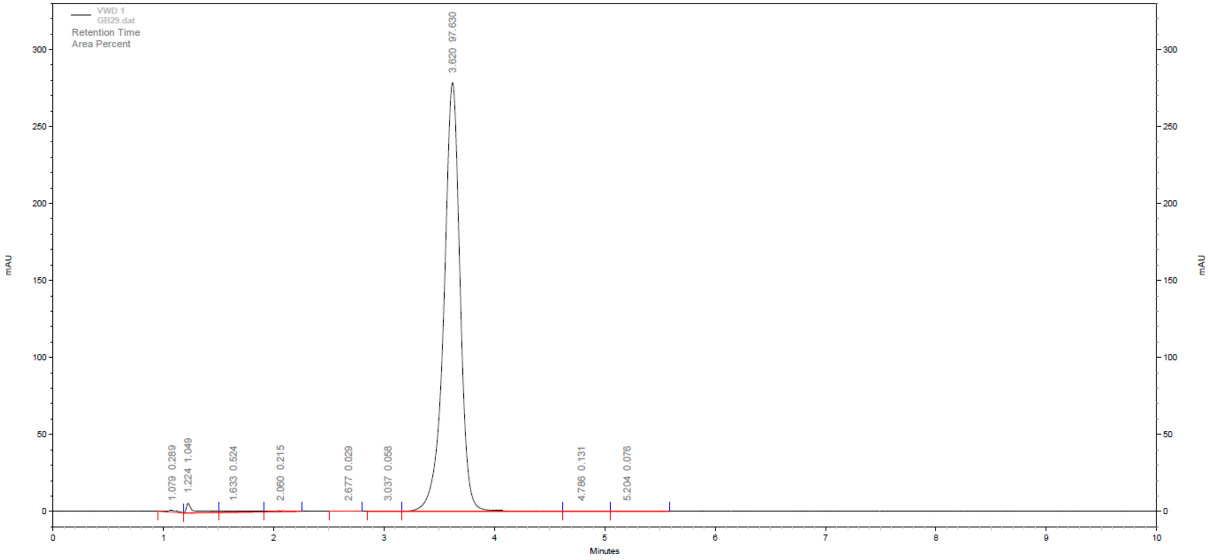
VWD 1
GB29.dat
Retention Time
Area Percent
3.620 97.630
1.079 0.289
1.224 1.049
1.633 0.524
2.060 0.215
2.677 0.029
3.037 0.058
4.786 0.131
5.204 0.076
mAU
Minutes

5. Mass

MS Spectrum

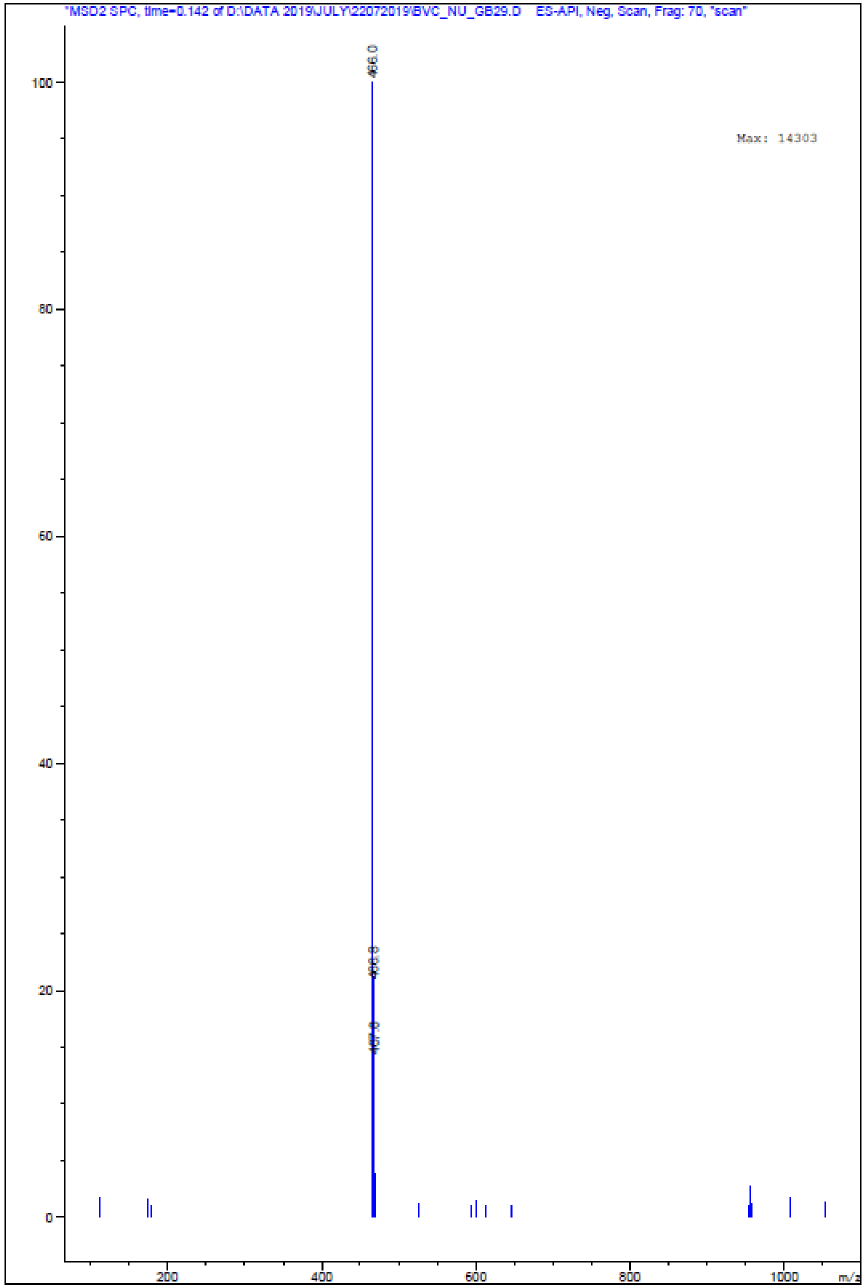
*MSD2 SPC, time=0.142 of D:\DATA 2019\JULY\22072019\BVC_NU_GB29.D   ES-API, Neg, Scan, Frag: 70, "scan"
466.0
Max: 14303
100
80
60
40
20
0
200
400
600
800
1000
m/z

2-(5-benzylidene-2,4-dioxothiazolidin-3-yl)-N-(4-fluorobenzo[d]thiazol-2-yl)acetamide (GB30)

1. FTIR

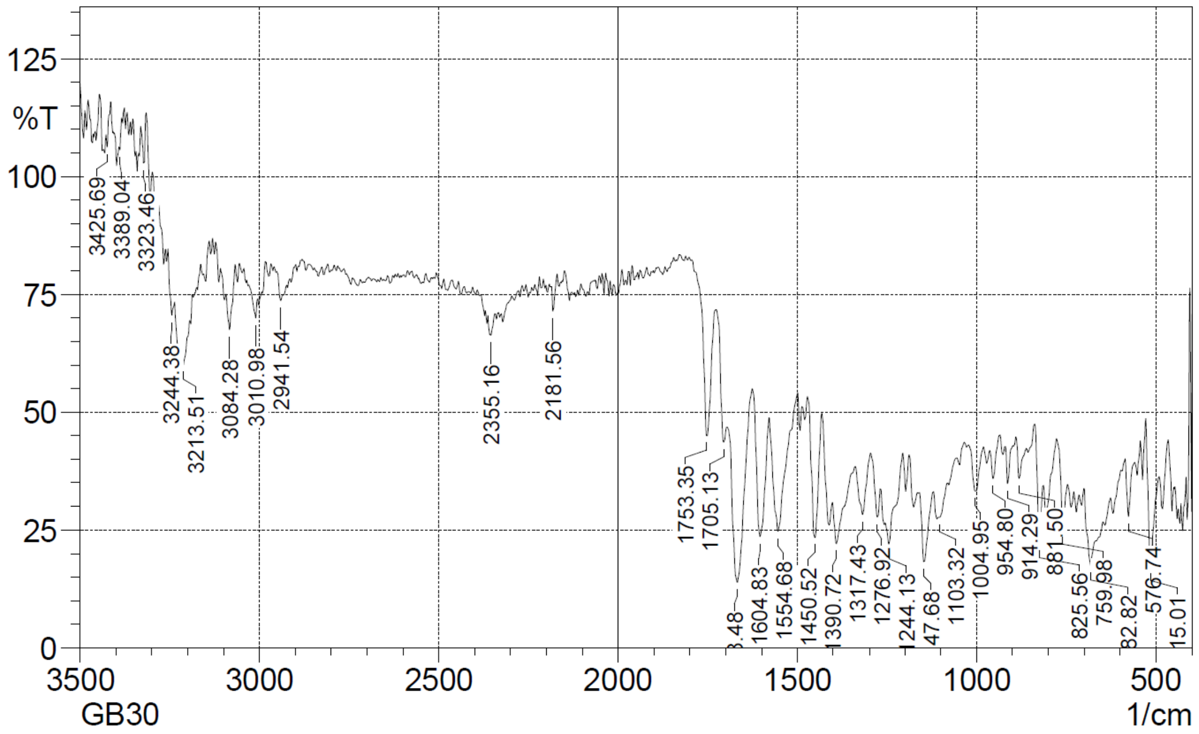

2. 1H-NMR

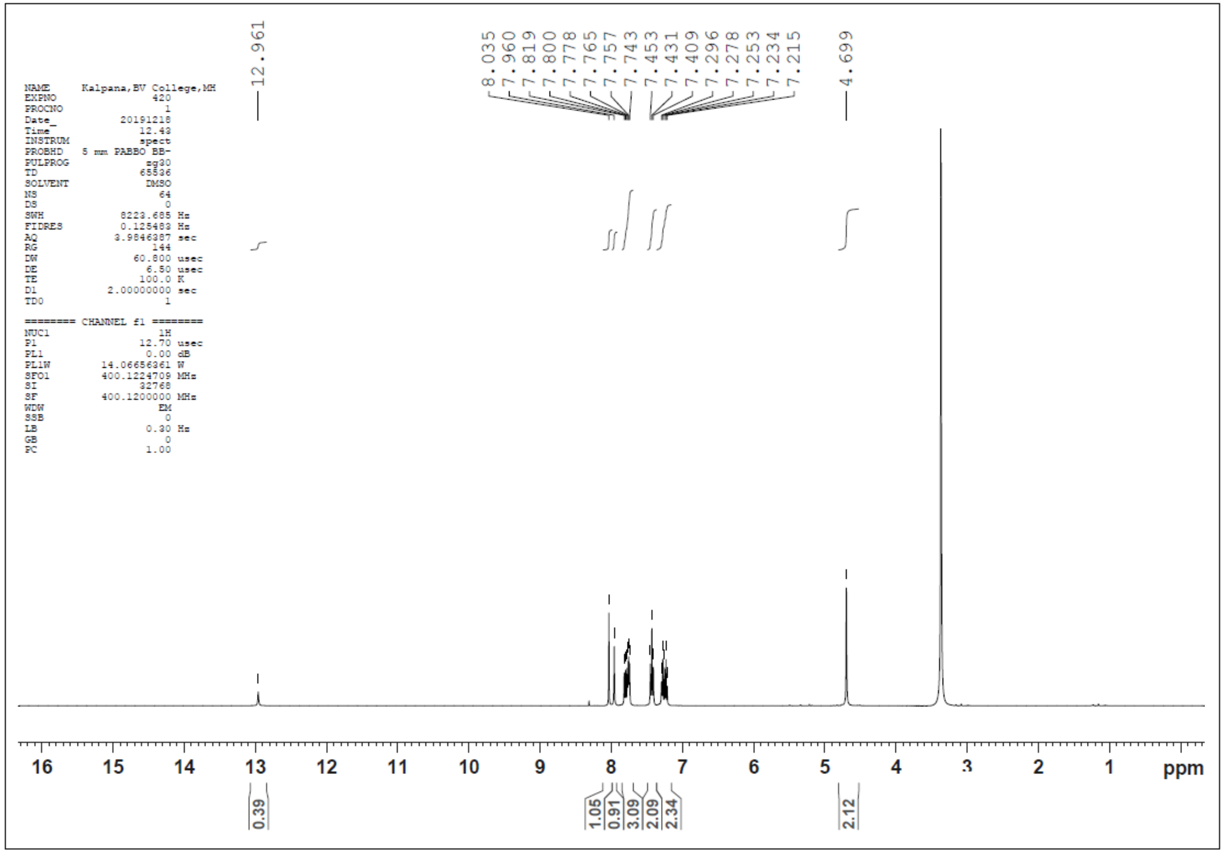
NAME Kalpana,BV College,MH
EXPNO 420
PROCNO 1
Date_ 20191218
Time 12.43
INSTRUM spect
PROBHD 5 mm PABBO BB-
PULPROG zg30
TD 65536
SOLVENT DMSO
NS 64
DS 0
SWH 8223.685 Hz
FIDRES 0.125483 Hz
AQ 3.9846387 sec
RG 144
DW 60.800 usec
DE 6.50 usec
TE 100.0 K
D1 2.00000000 sec
TD0 1
======== CHANNEL f1 ========
NUC1 1H
P1 12.70 usec
PL1 0.00 dB
PL1W 14.06656361 W
SFO1 400.1224709 MHz
SI 32768
SF 400.1200000 MHz
WDW EM
SSB 0
LB 0.30 Hz
GB 0
PC 1.00
12.961
8.035
7.960
7.819
7.800
7.778
7.765
7.757
7.743
7.453
7.431
7.409
7.296
7.278
7.253
7.234
7.215
4.699
16 15 14 13 12 11 10 9 8 7 6 5 4 3 2 1 ppm
0.39
1.05
0.91
3.09
2.09
2.34
2.12

3. 13C-NMR

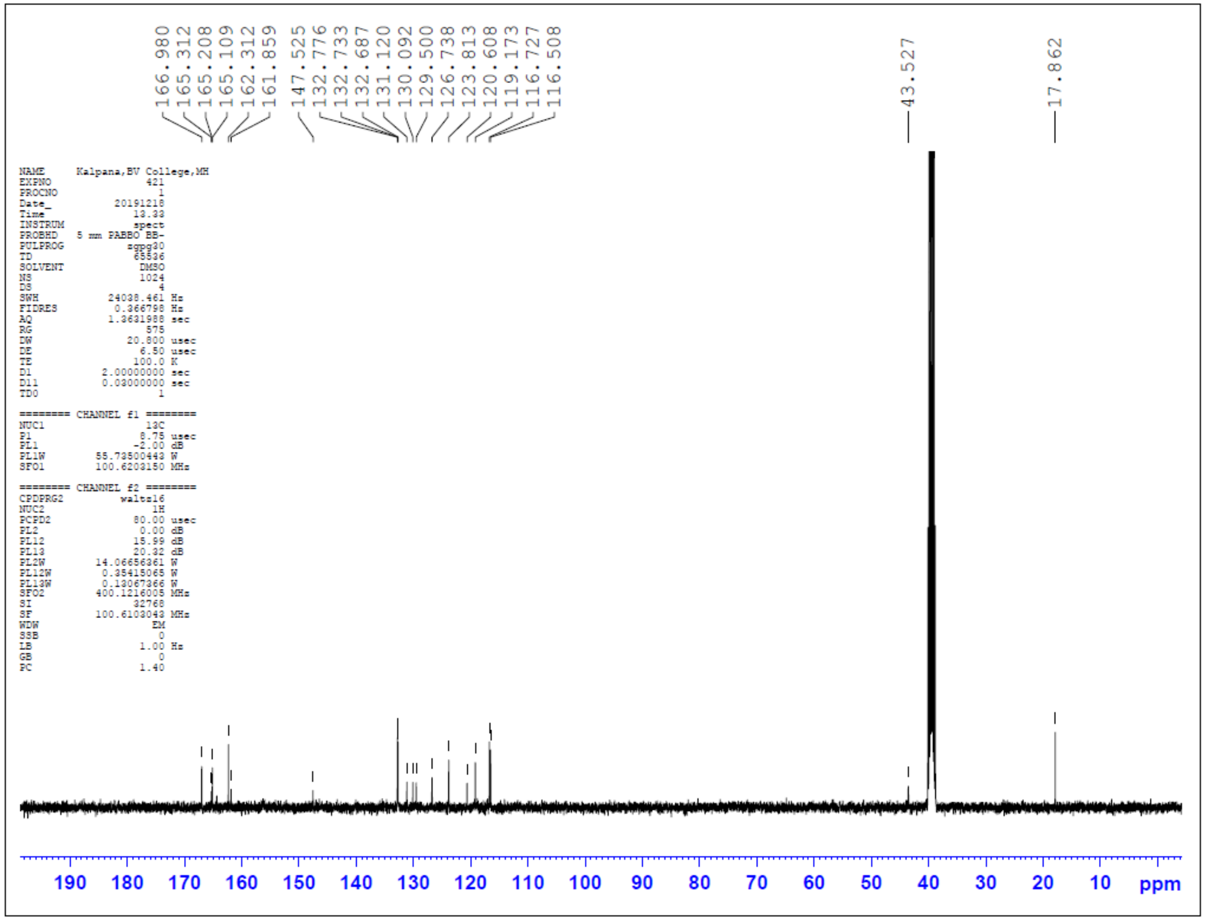
166.980
165.312
165.208
165.109
162.312
161.859
147.525
132.776
132.733
132.687
131.120
130.092
129.500
126.738
123.813
120.608
119.173
116.727
116.508
43.527
17.862
NAME Kalpana,BV College,MH
EXPNO 421
PROCNO 1
Date_ 20191218
Time 13.33
INSTRUM spect
PROBHD 5 mm PABBO BB-
PULPROG zgpg30
TD 65536
SOLVENT DMSO
NS 1024
DS 4
SWH 24038.461 Hz
FIDRES 0.366798 Hz
AQ 1.3631988 sec
RG 575
DW 20.800 usec
DE 6.50 usec
TE 100.0 K
D1 2.00000000 sec
D11 0.03000000 sec
TD0 1
======== CHANNEL f1 ========
NUC1 13C
P1 8.75 usec
PL1 -2.00 dB
PL1W 55.73500443 W
SFO1 100.6203150 MHz
======== CHANNEL f2 ========
CPDPRG2 waltz16
NUC2 1H
PCPD2 80.00 usec
PL2 0.00 dB
PL12 15.99 dB
PL13 20.32 dB
PL2W 14.06656361 W
PL12W 0.35415065 W
PL13W 0.13067366 W
SFO2 400.1216005 MHz
SI 32768
SF 100.6103043 MHz
WDW EM
SSB 0
LB 1.00 Hz
GB 0
PC 1.40
190 180 170 160 150 140 130 120 110 100 90 80 70 60 50 40 30 20 10 ppm

4. HPLC

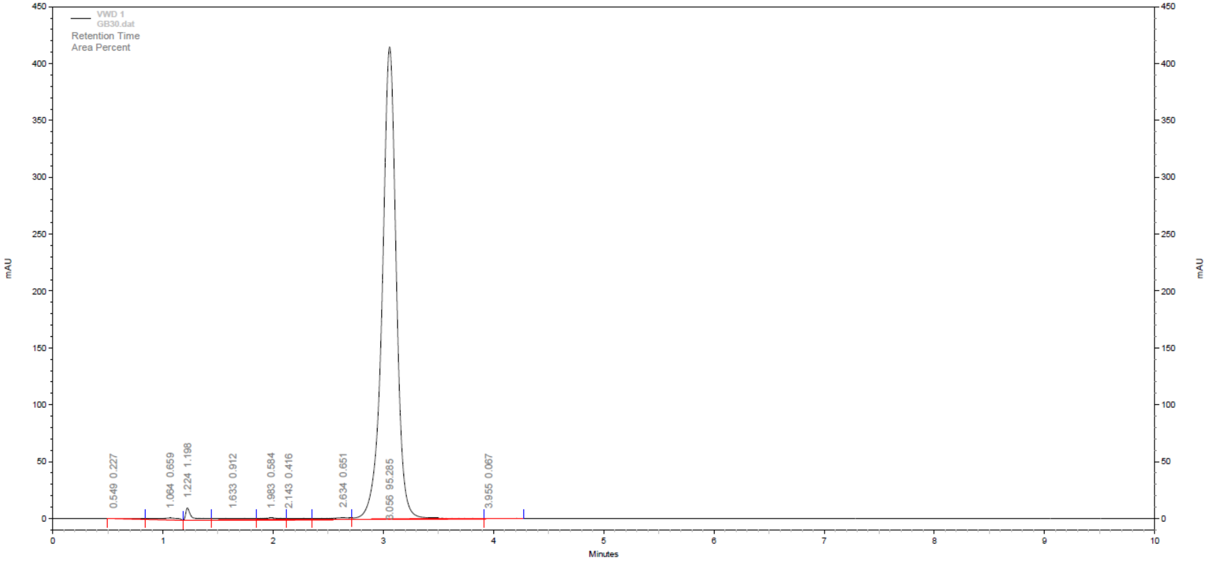
VWD 1
GB30.dat
Retention Time
Area Percent
mAU
Minutes
0.549 0.227
1.064 0.659
1.224 1.198
1.633 0.912
1.983 0.584
2.143 0.416
2.634 0.651
3.056 95.285
3.955 0.067

5. Mass

MS Spectrum

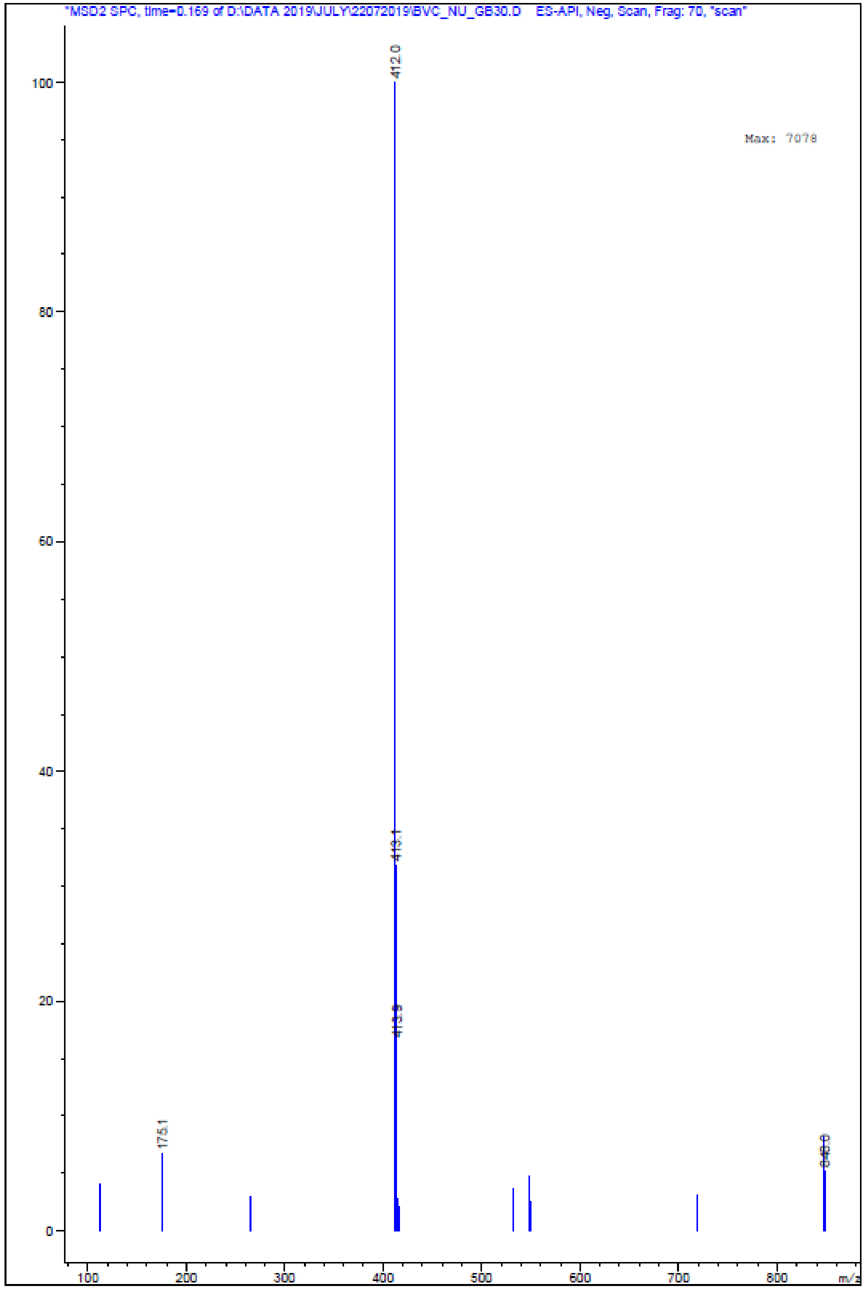
"MSD2 SPC, time=0.169 of D:\DATA 2019\JULY\22072019\BVC_NU_GB30.D ES-API, Neg, Scan, Frag: 70, "scan"
412.0
413.1
175.1
Max: 7078
100
80
60
40
20
0
100
200
300
400
500
600
700
800
m/z

2-(5-(4-bromobenzylidene)-2,4-dioxothiazolidin-3-yl)-N-(6-methylbenzo[d]thiazol-2-yl)acetamide (GB31)

1. FTIR

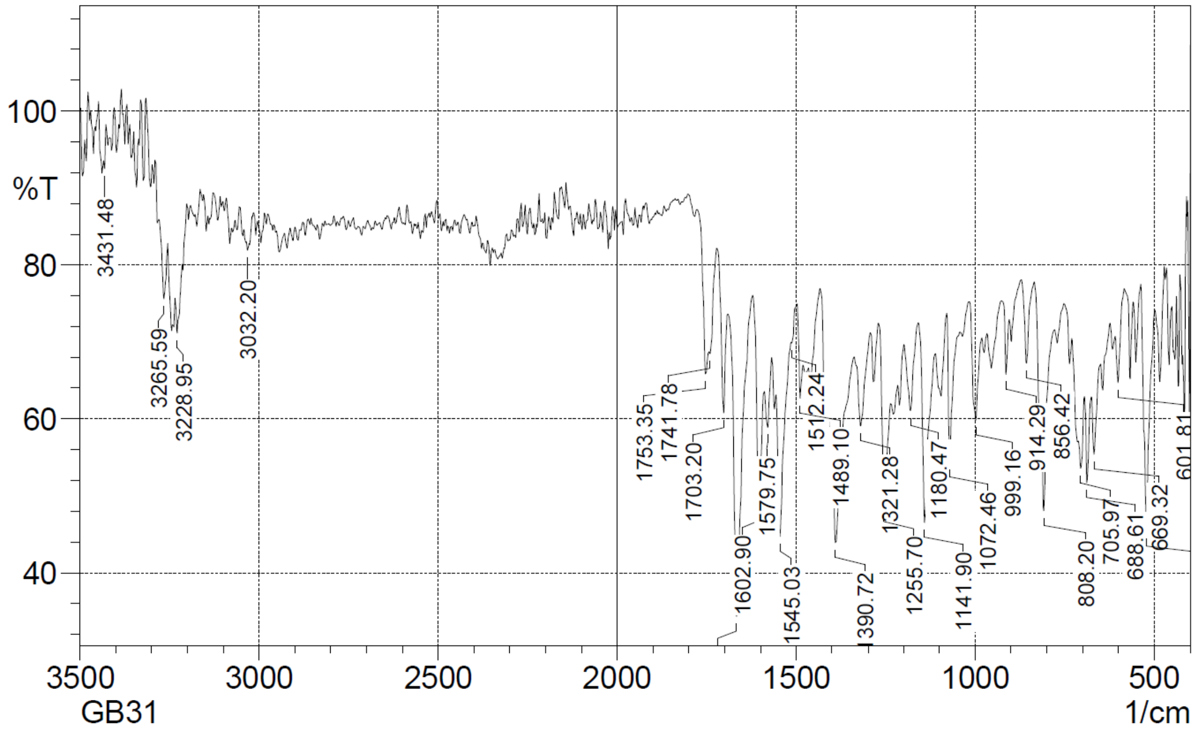

2. ^{1}HNMR

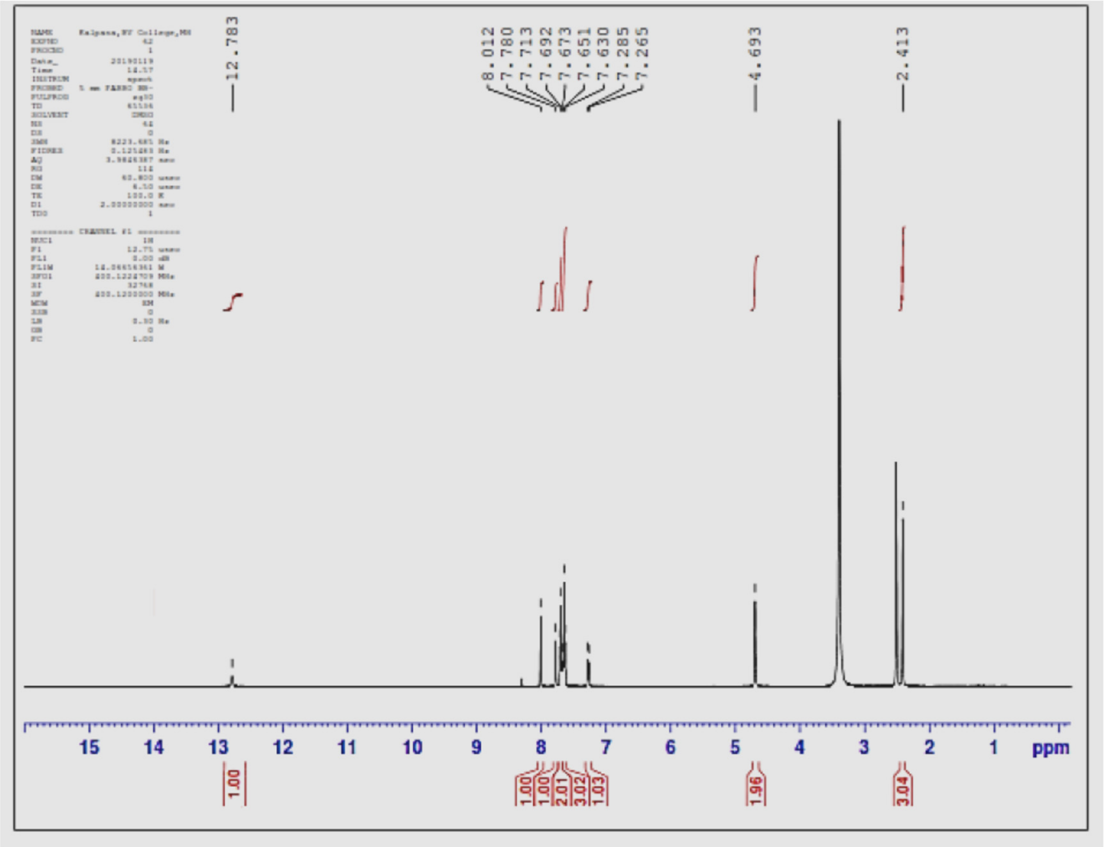
12.783
8.012
7.780
7.713
7.692
7.673
7.651
7.630
7.285
7.265
4.693
2.413
15 14 13 12 11 10 9 8 7 6 5 4 3 2 1 ppm
1.00
1.00
1.00
2.01
3.02
1.03
1.96
3.04

3. ^{13}CNMR

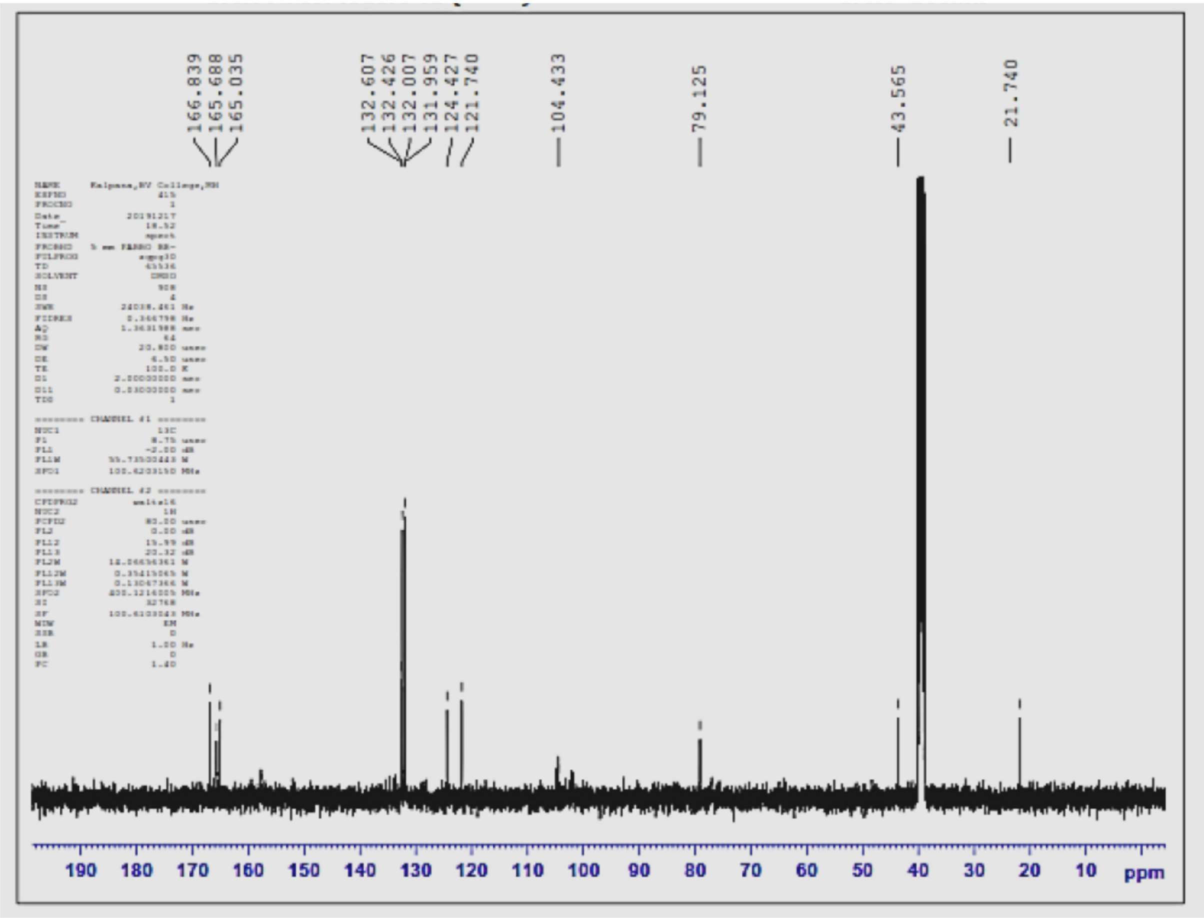
166.839
165.688
165.035
132.607
132.426
132.007
131.959
124.427
121.740
104.433
79.125
43.565
21.740
190 180 170 160 150 140 130 120 110 100 90 80 70 60 50 40 30 20 10 ppm

4. HPLC

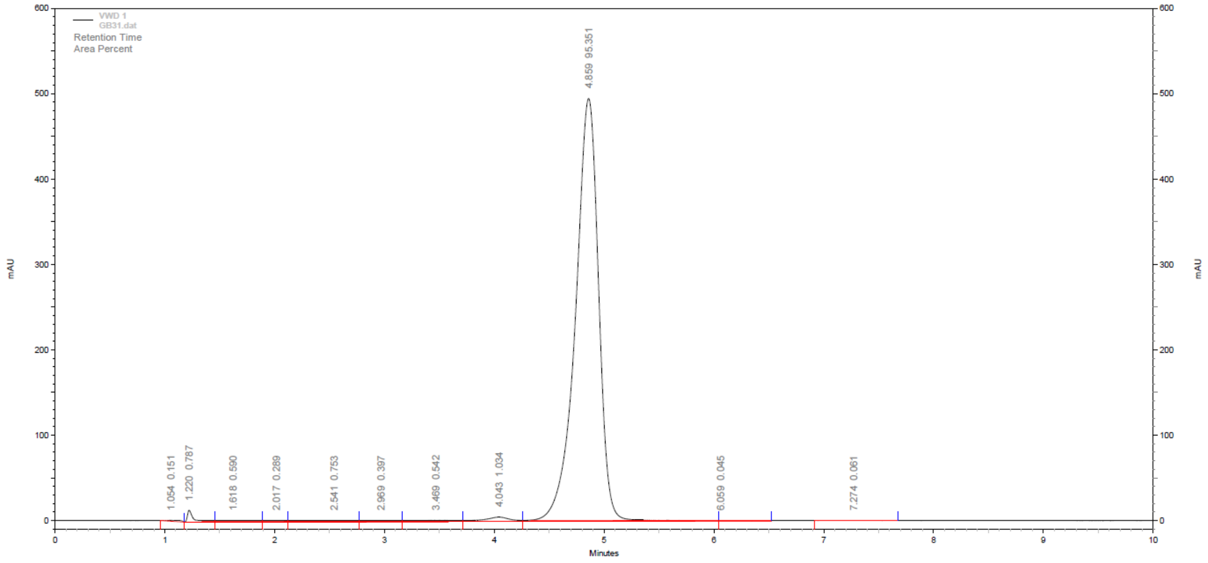
VWD 1
GB31.dat
Retention Time
Area Percent
4.859 95.351
1.054 0.151
1.220 0.787
1.618 0.590
2.017 0.289
2.541 0.753
2.969 0.397
3.469 0.542
4.043 1.034
6.059 0.045
7.274 0.061
mAU
Minutes

5. Mass

MS Spectrum

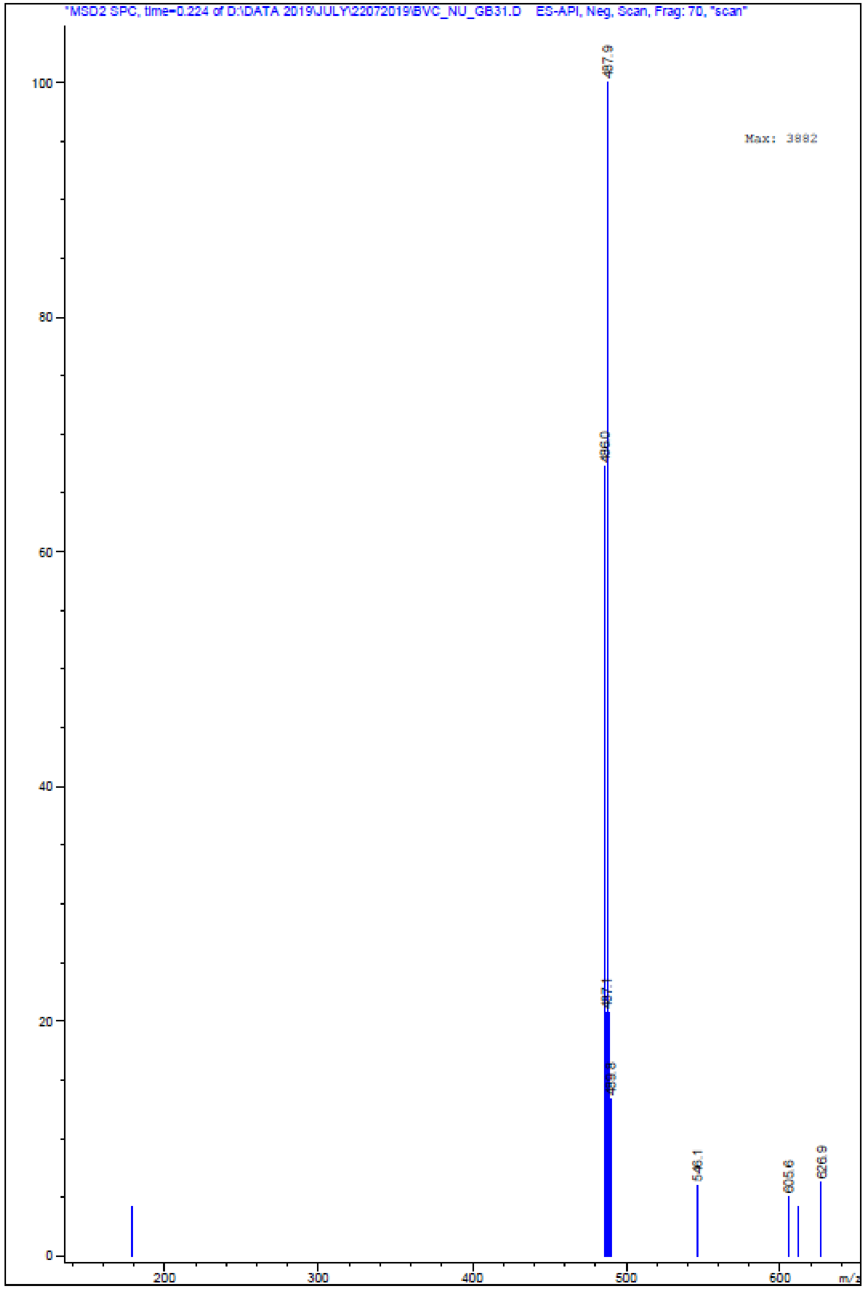

*MSD2 SPC, time=0.224 of D:\DATA 2019\JULY\22072019\BVC_NU_GB31.D ES-API, Neg, Scan, Frag: 70, "scan"
Max: 3882
487.9
486.0
546.1
605.6
626.9
100
80
60
40
20
0
200
300
400
500
600
m/z

N-(6-fluorobenzo[d]thiazol-2-yl)-2-(5-(4-methylbenzylidene)-2,4-dioxothiazolidin-3-yl)acetamide (GB32)

1. FTIR

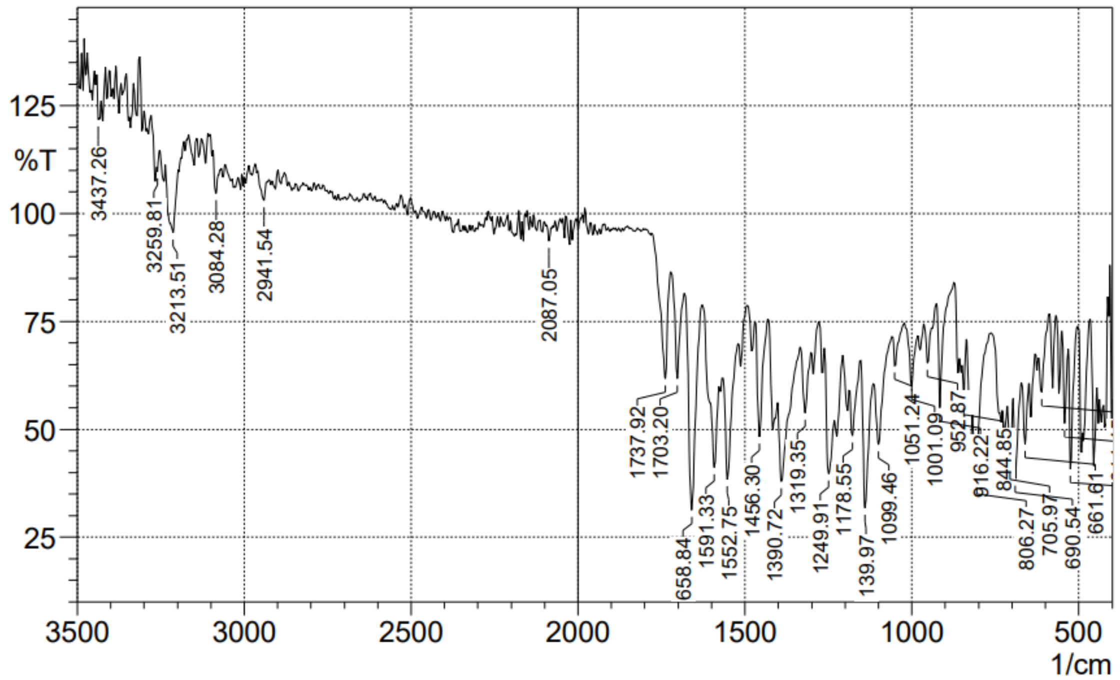

2. ^{I}H-NMR

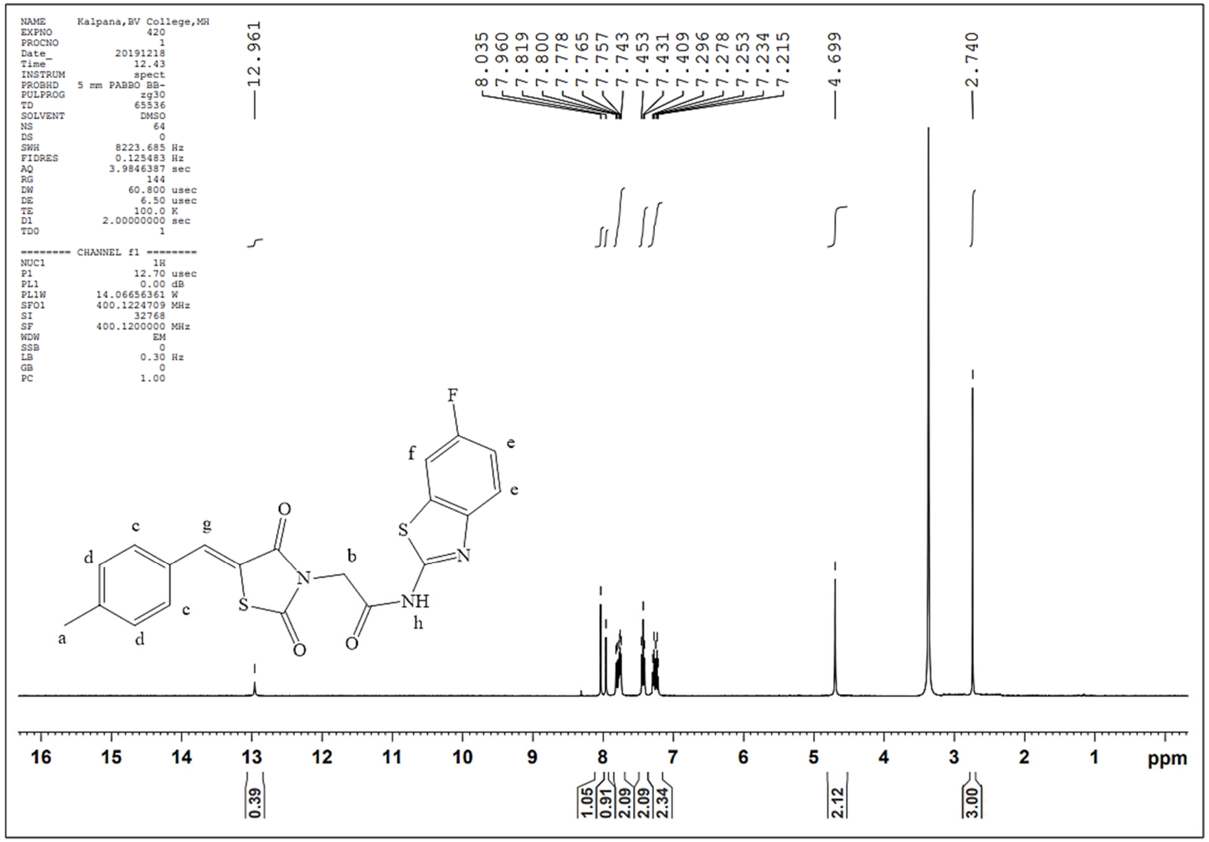
NAME Kalpana,BV College,MH
EXPNO 420
PROCNO 1
Date_ 20191218
Time 12.43
INSTRUM spect
PROBHD 5 mm PABBO BB-
PULPROG zg30
TD 65536
SOLVENT DMSO
NS 64
DS 0
SWH 8223.685 Hz
FIDRES 0.125483 Hz
AQ 3.9846387 sec
RG 144
DW 60.800 usec
DE 6.50 usec
TE 100.0 K
D1 2.00000000 sec
TD0 1
======== CHANNEL f1 ========
NUC1 1H
P1 12.70 usec
PL1 0.00 dB
PL1W 14.06656361 W
SFO1 400.1224709 MHz
SI 32768
SF 400.1200000 MHz
WDW EM
SSB 0
LB 0.30 Hz
GB 0
PC 1.00
12.961
8.035
7.960
7.819
7.800
7.778
7.765
7.757
7.743
7.453
7.431
7.409
7.296
7.278
7.253
7.234
7.215
4.699
2.740
F
e
f
e
O
c
g
S
b
N
d
N
NH
S
c
h
a
d
O
O
16 15 14 13 12 11 10 9 8 7 6 5 4 3 2 1 ppm
0.39
1.05
0.91
2.09
2.09
2.34
2.12
3.00

3. ^{13}C-NMR

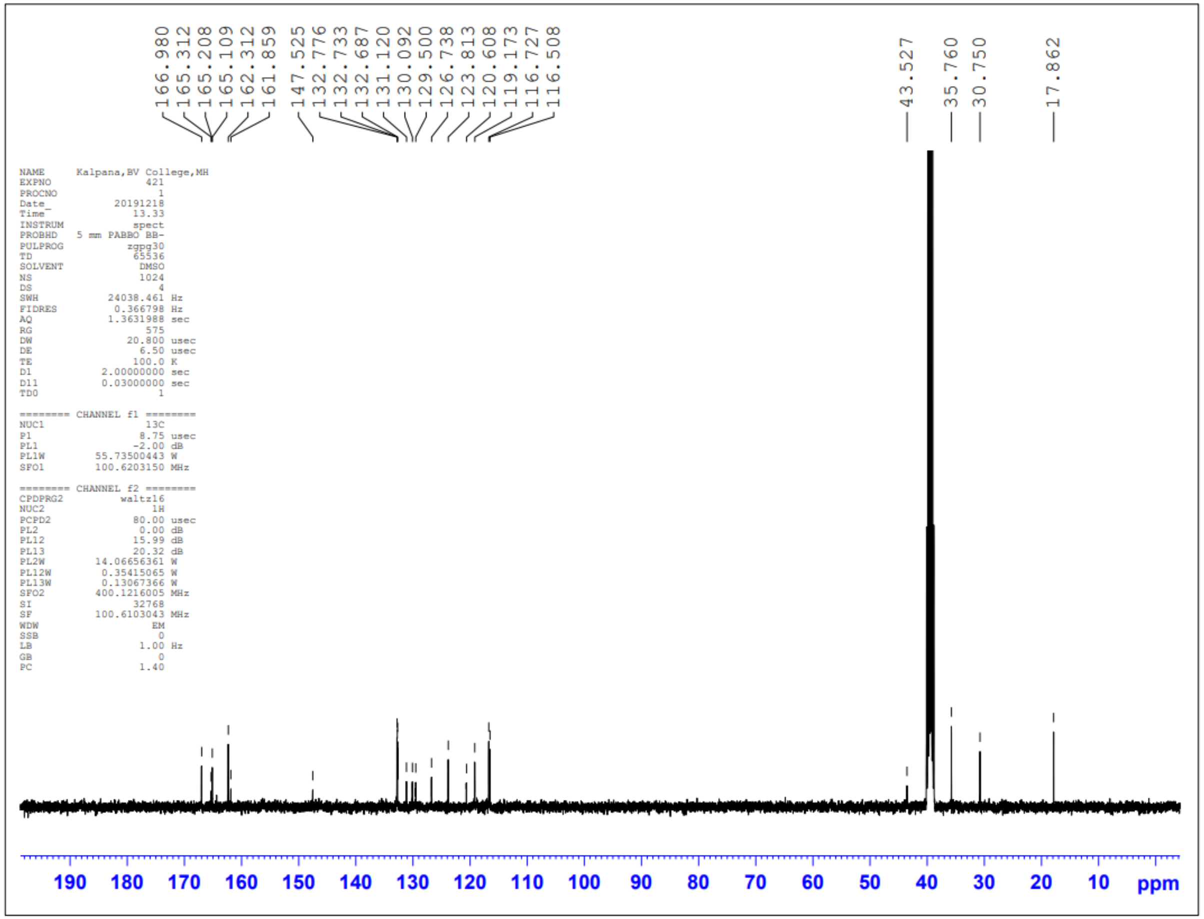

4. HPLC Analysis

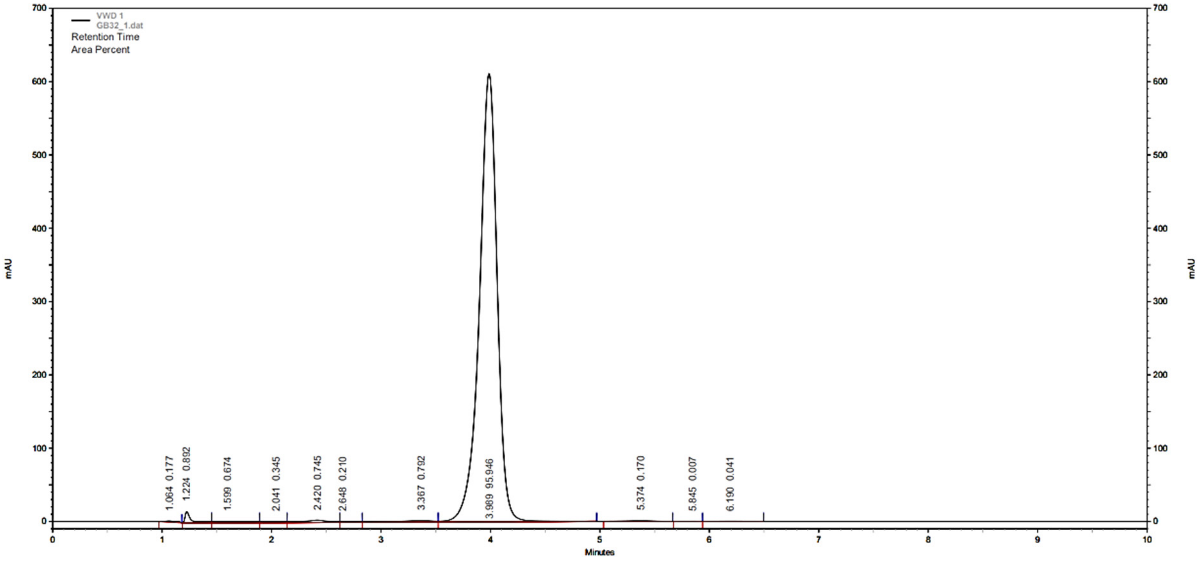

5. Mass Spectrometry

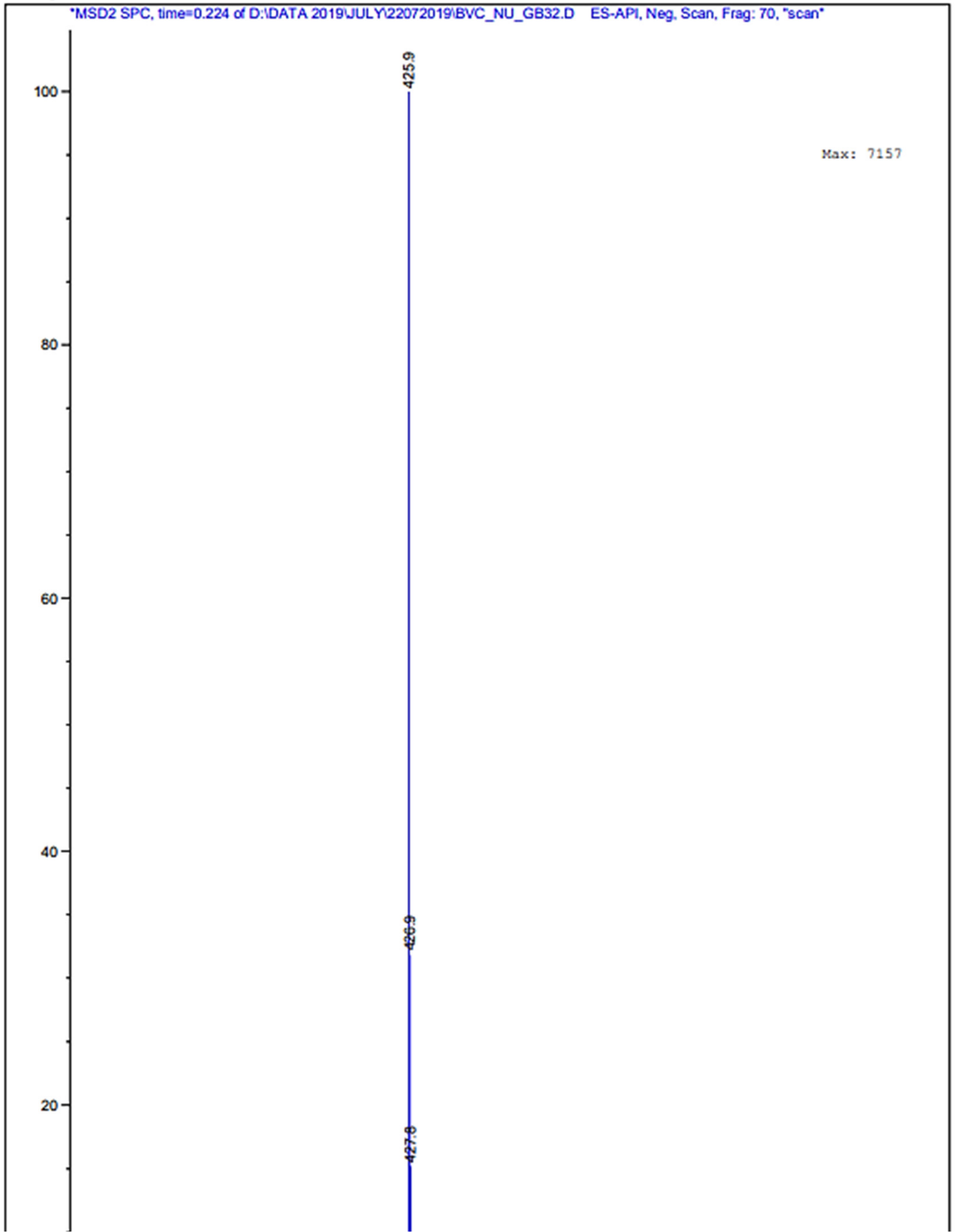
*MSD2 SPC, time=0.224 of D:\DATA 2019\JULY\22072019\BVC_NU_GB32.D ES-API, Neg, Scan, Frag: 70, "scan"
Max: 7157
100
80
60
40
20
425.9
426.9
427.8

6. UV

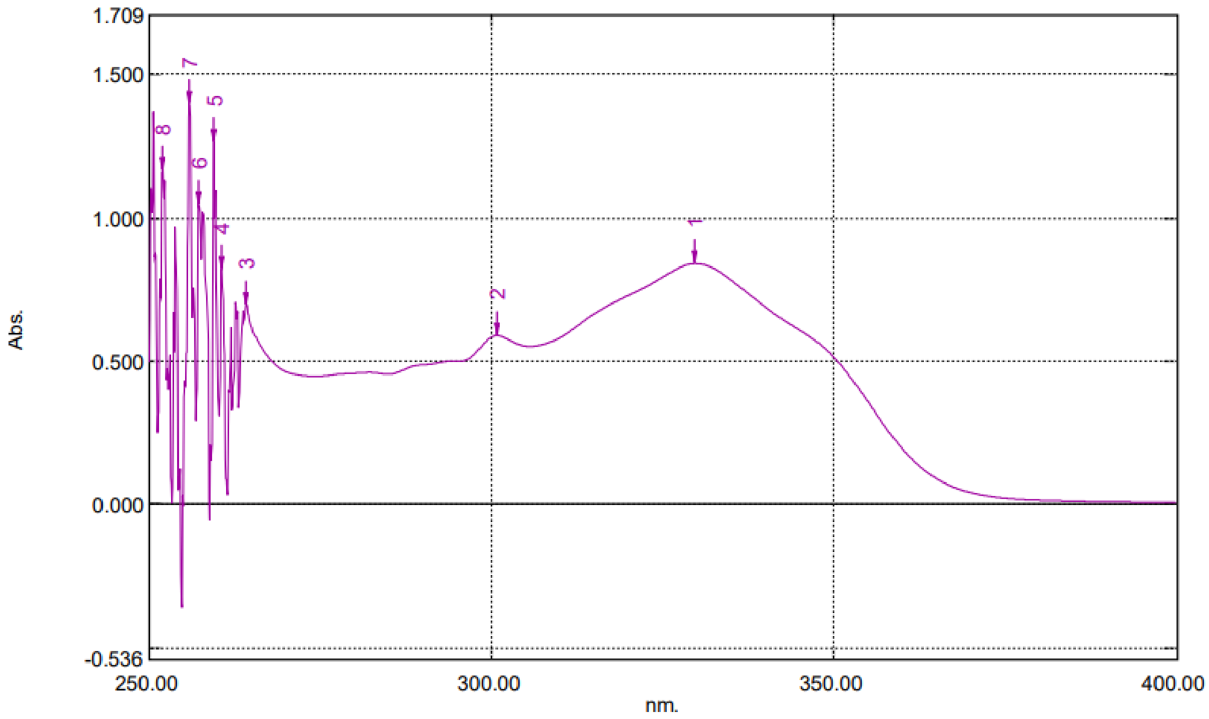
1.709
1.500
1.000
0.500
0.000
-0.536
Abs.
250.00
300.00
350.00
400.00
nm.
1
2
3
4
5
6
7
8

2-(5-(4-chlorobenzylidene)-2,4-dioxothiazolidin-3-yl)-N-(6-fluorobenzo[d]thiazol-2-yl)acetamide (GB33)

1. FTIR

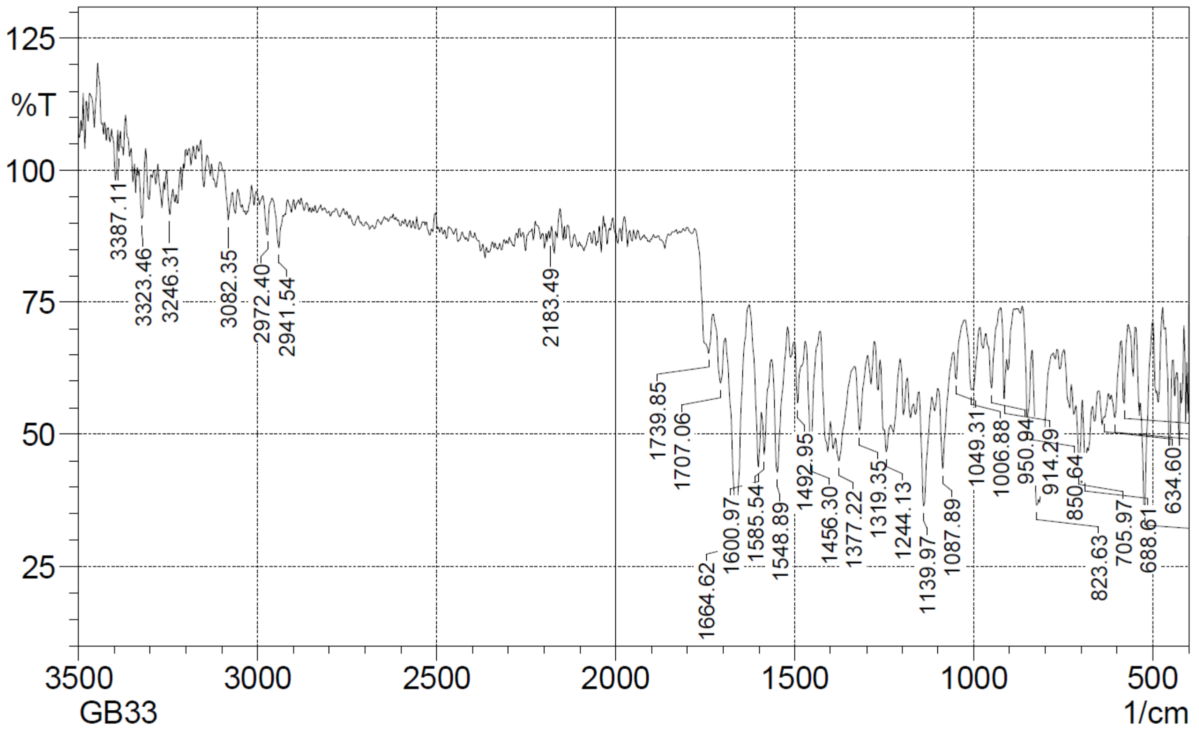

2. ^{1}HNMR

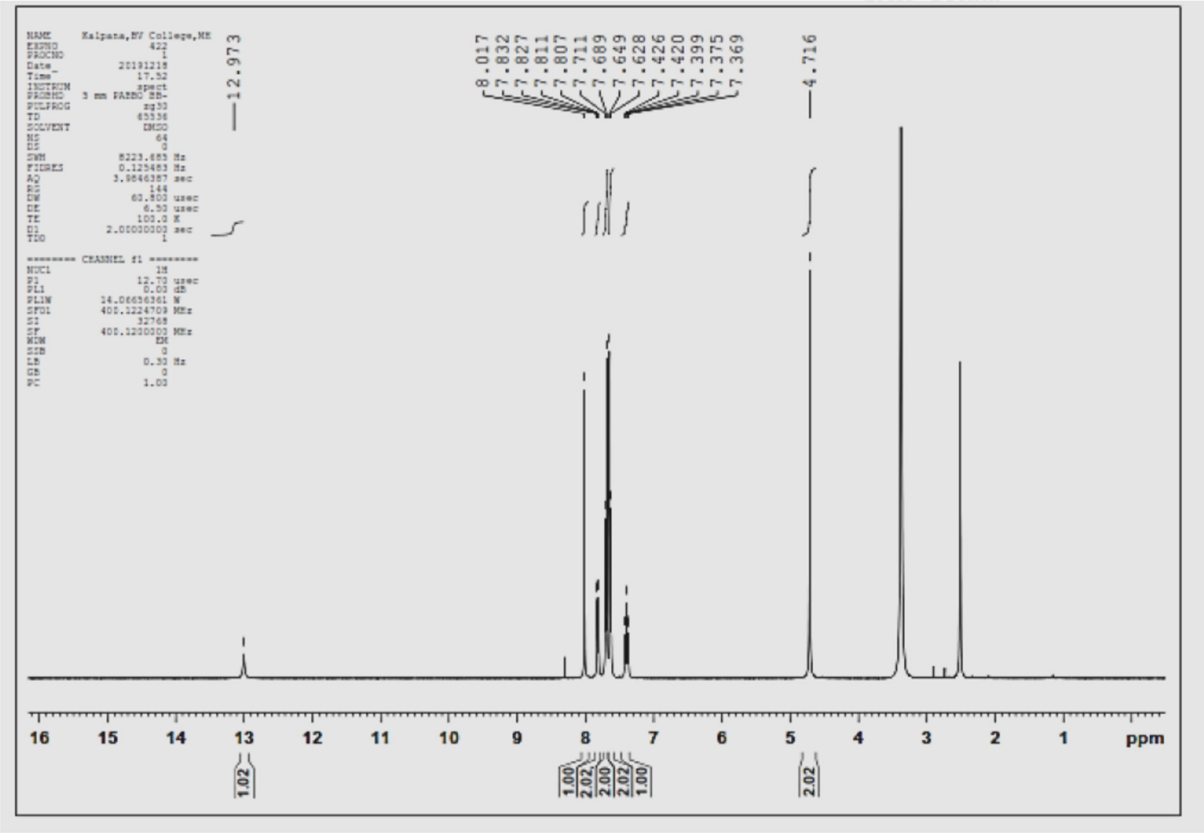
NAME Kalpana,BV College,ME
EXPNO 422
PROCNO 1
Date_ 20191218
Time 17.52
INSTRUM spect
PROBHD 5 mm PABBO BB-
PULPROG zg30
TD 65536
SOLVENT DMSO
NS 64
DS 0
SWH 8223.685 Hz
FIDRES 0.125483 Hz
AQ 3.9846387 sec
RG 144
DW 60.800 usec
DE 6.50 usec
TE 300.0 K
D1 2.00000000 sec
TD0 1
======== CHANNEL f1 ========
NUC1 1H
P1 12.70 usec
PL1 0.00 dB
PL1W 14.06636361 W
SFO1 400.1224709 MHz
SI 32768
SF 400.1200000 MHz
WDW EM
SSB 0
LB 0.30 Hz
GB 0
PC 1.00
12.973
8.017
7.832
7.827
7.811
7.807
7.711
7.689
7.649
7.628
7.426
7.420
7.399
7.375
7.369
4.716
16
15
14
13
12
11
10
9
8
7
6
5
4
3
2
1
ppm
1.02
1.00
2.02
2.00
2.02
1.00
2.02

3. ^{13}CNMR

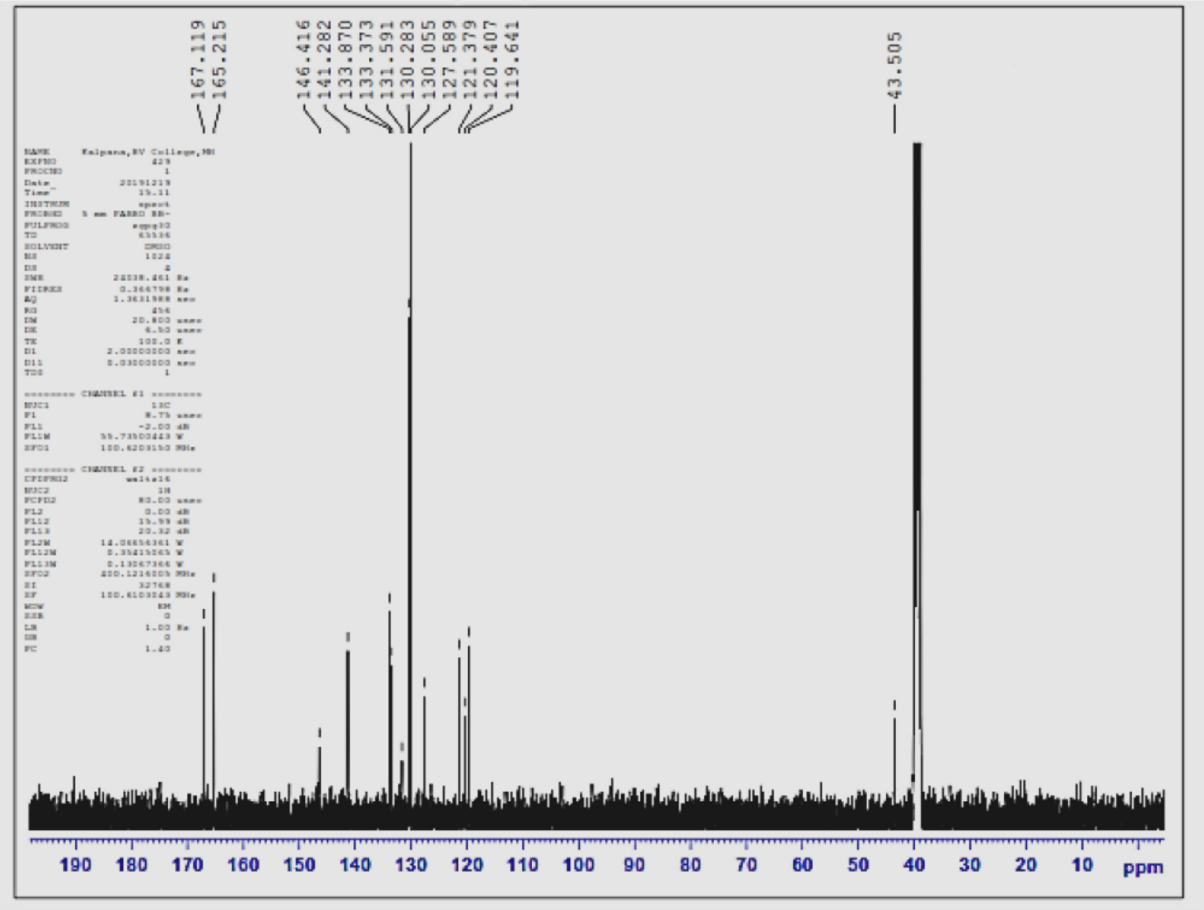
167.119
165.215
146.416
141.282
133.870
133.373
131.591
130.283
130.055
127.589
121.379
120.407
119.641
43.505
190 180 170 160 150 140 130 120 110 100 90 80 70 60 50 40 30 20 10 ppm

2. HPLC

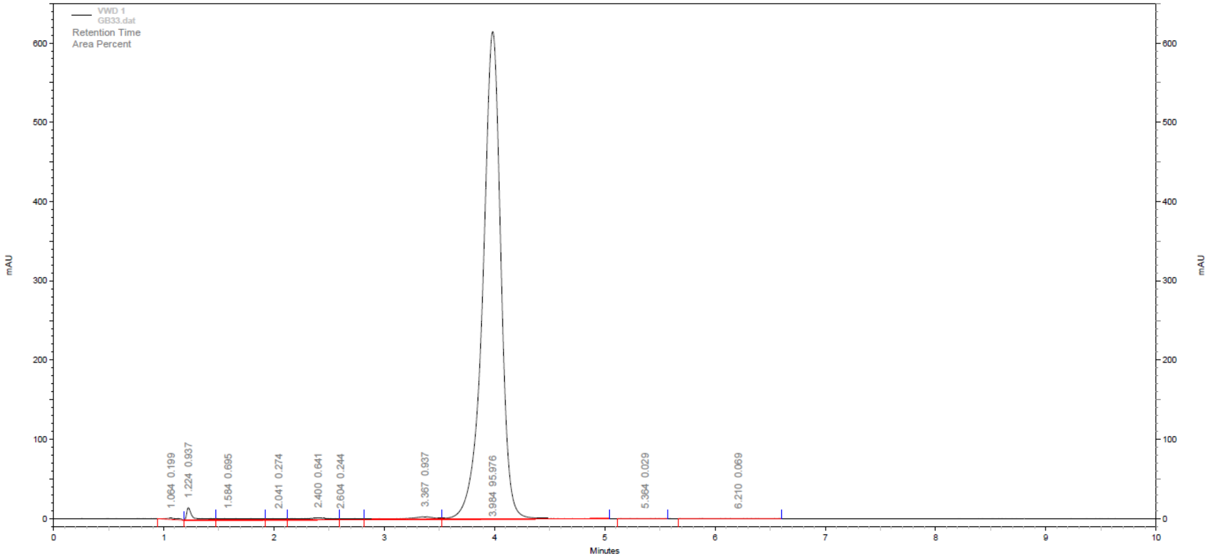
VWD 1
GB33.dat
Retention Time
Area Percent
mAU
Minutes
1.064 0.199
1.224 0.937
1.584 0.695
2.041 0.274
2.400 0.641
2.604 0.244
3.367 0.937
3.984 95.976
5.364 0.029
6.210 0.069

3. Mass

MS Spectrum

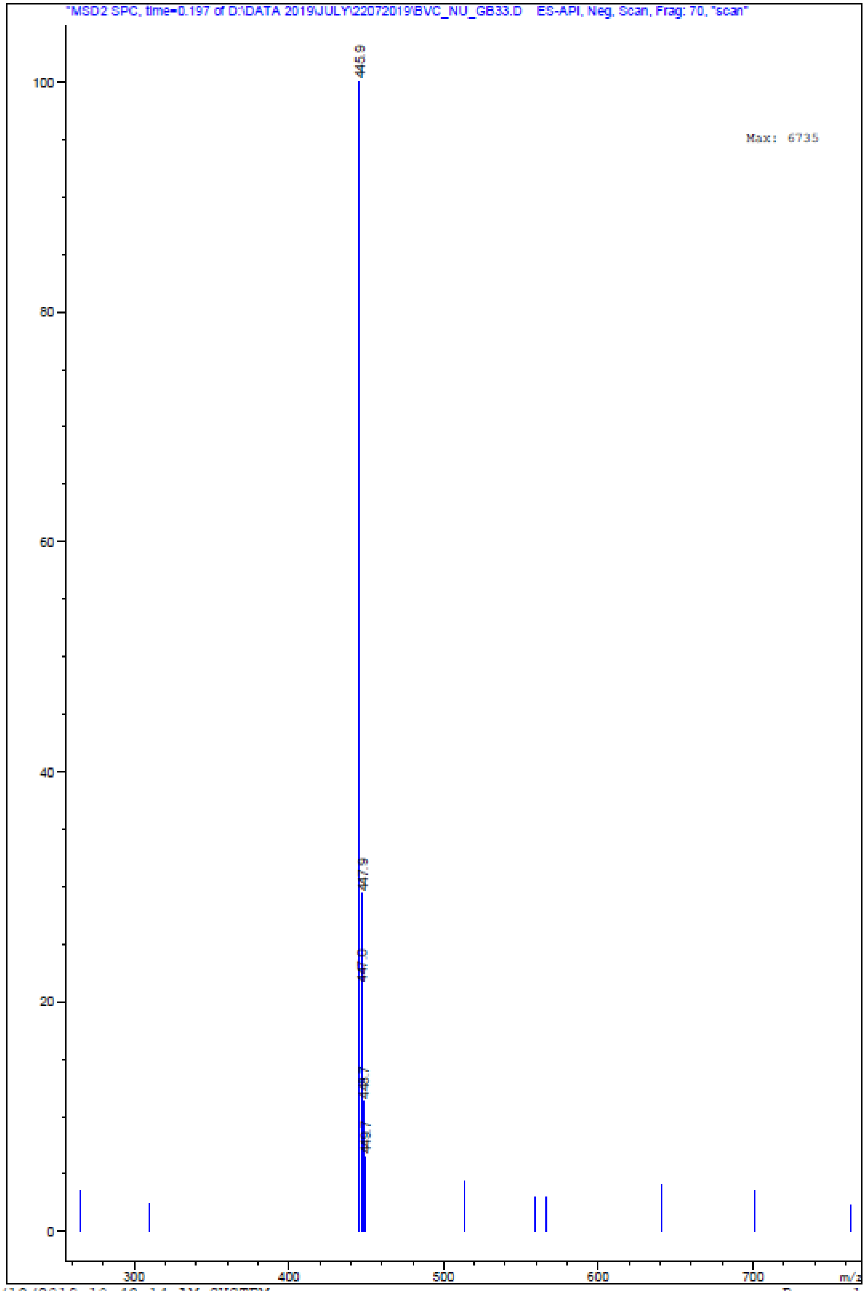

"MSD2 SPC, time=0.197 of D:\DATA 2019\JULY\22072019\BVC_NU_GB33.D ES-API, Neg, Scan, Frag: 70, "scan"
445.9
447.9
Max: 6735
100
80
60
40
20
0
300
400
500
600
700
m/z

N-(6-fluorobenzo[d]thiazol-2-yl)-2-(5-(4-fluorobenzylidene)-2,4-dioxothiazolidin-3-yl)acetamide (GB34)

1. FTIR

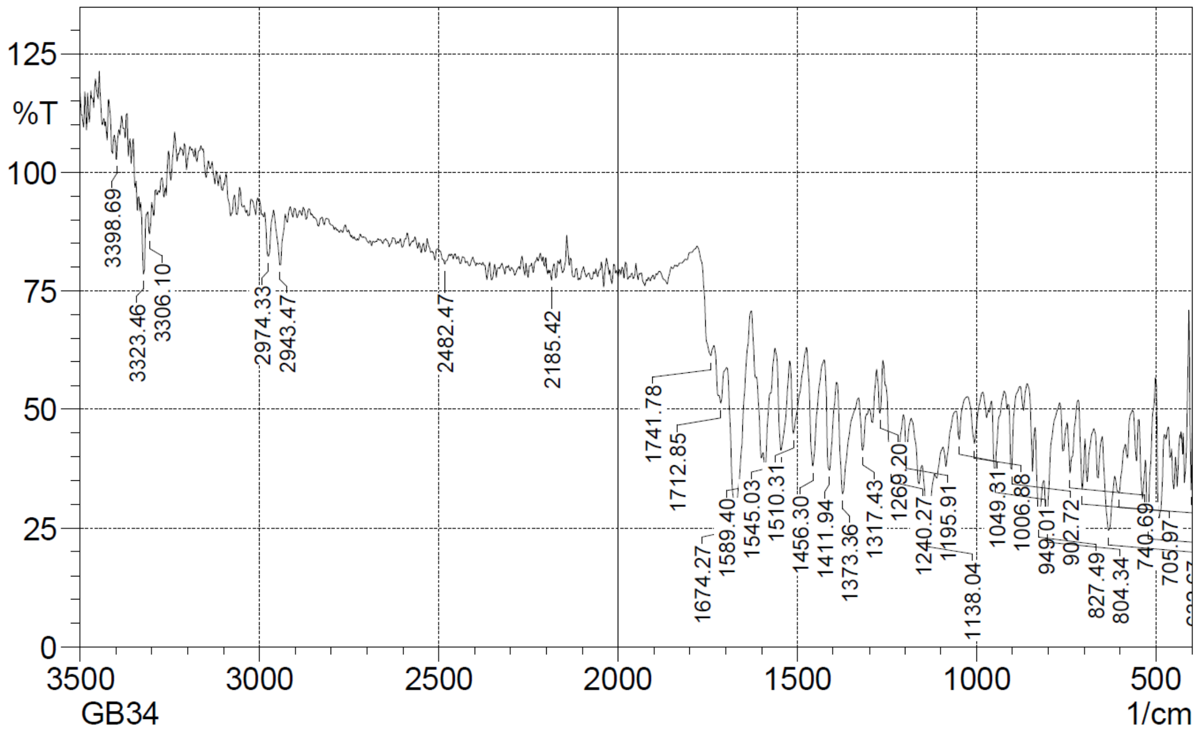

2. ^{1}HNMR

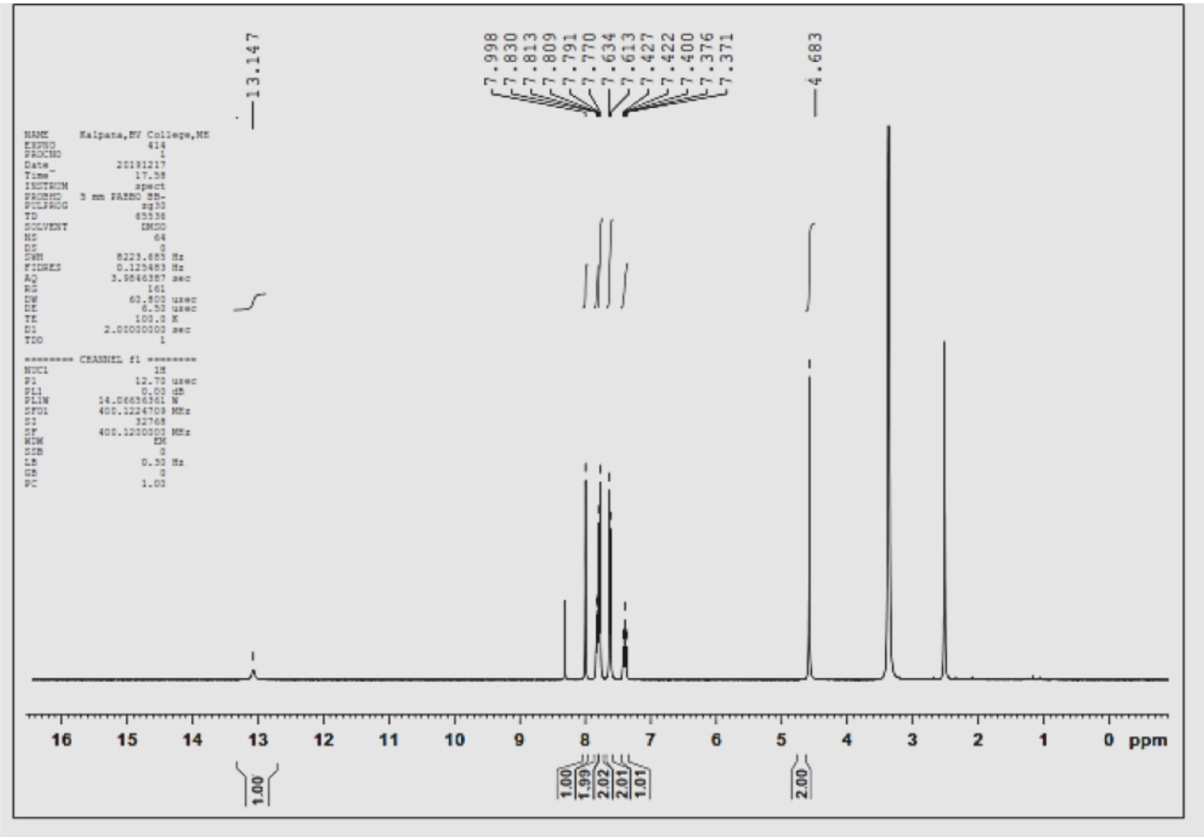

3. ^{13}CNMR

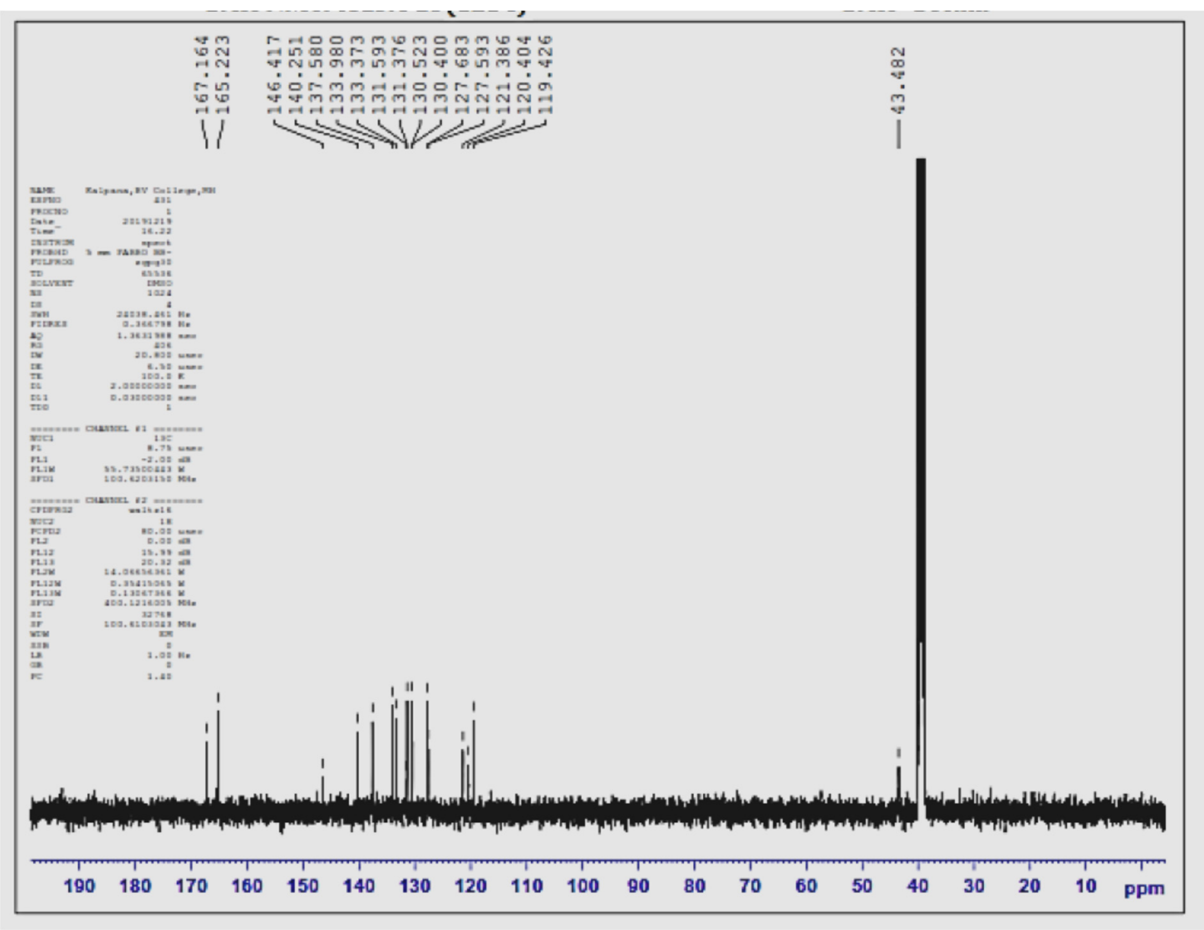
167.164
165.223
146.417
140.251
137.580
133.980
133.373
131.593
131.376
130.523
130.400
127.683
127.593
121.386
120.404
119.426
43.482
190 180 170 160 150 140 130 120 110 100 90 80 70 60 50 40 30 20 10 ppm

4. HPLC

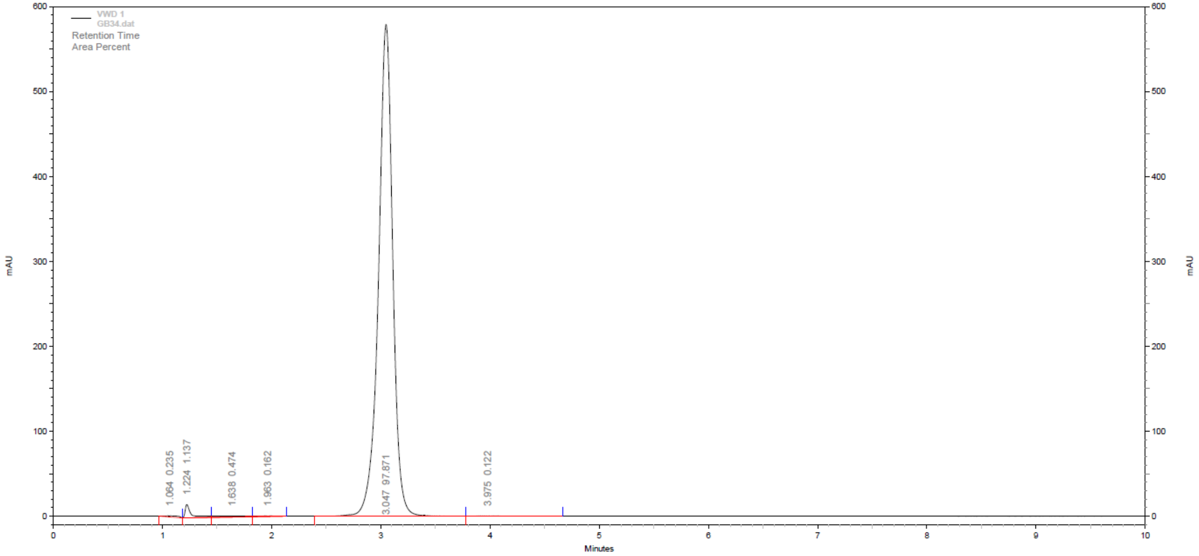

VWD 1
GB34.dat
Retention Time
Area Percent
mAU
Minutes
1.064 0.235
1.224 1.137
1.638 0.474
1.963 0.162
3.047 97.871
3.975 0.122

5. Mass

MS Spectrum

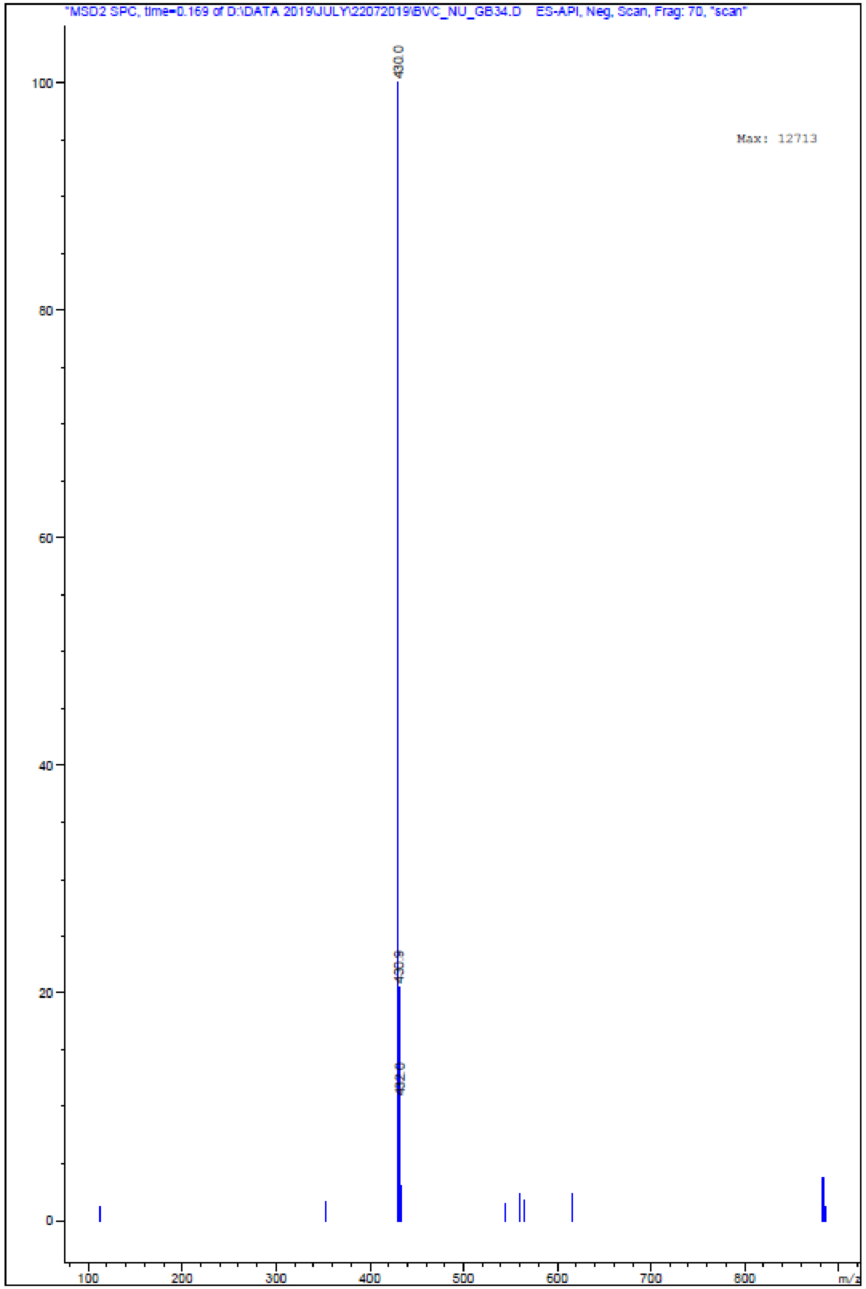
"MSD2 SPC, time=0.169 of D:\DATA 2019\JULY\22072019\BVC_NU_GB34.D ES-API, Neg, Scan, Frag: 70, "scan"
430.0
Max: 12713
100
80
60
40
20
0
100
200
300
400
500
600
700
800
m/z

2-(5-(4-fluorobenzylidene)-2,4-dioxothiazolidin-3-yl)-N-(4-methoxybenzo[d]thiazol-2-yl)acetamide (GB35)

1. FTIR

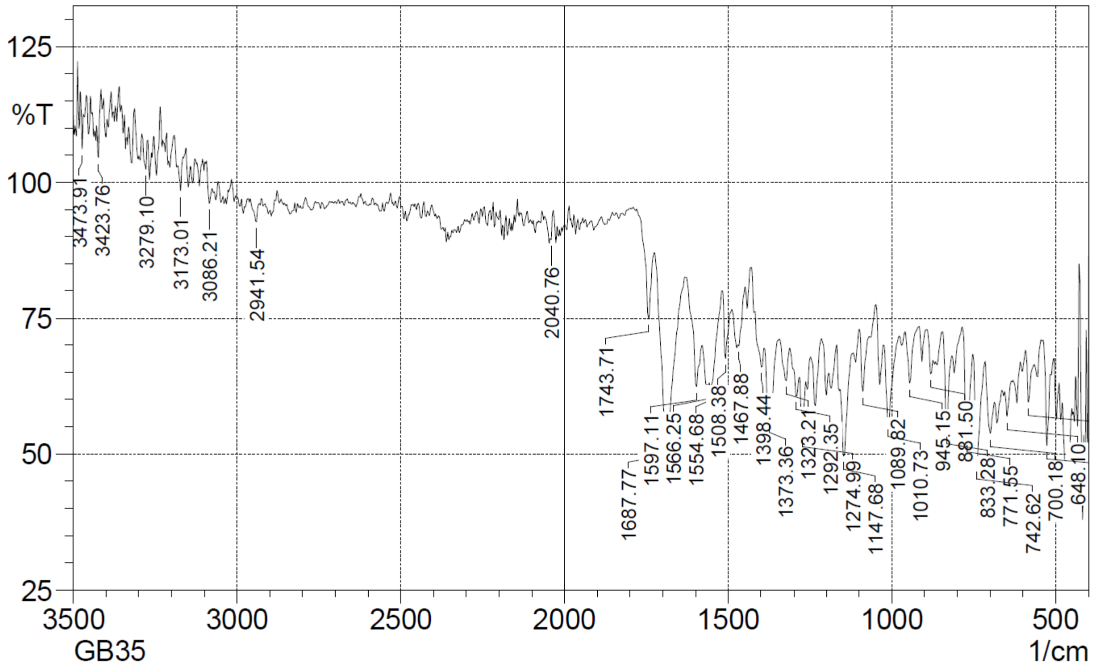

2. 1H-NMR

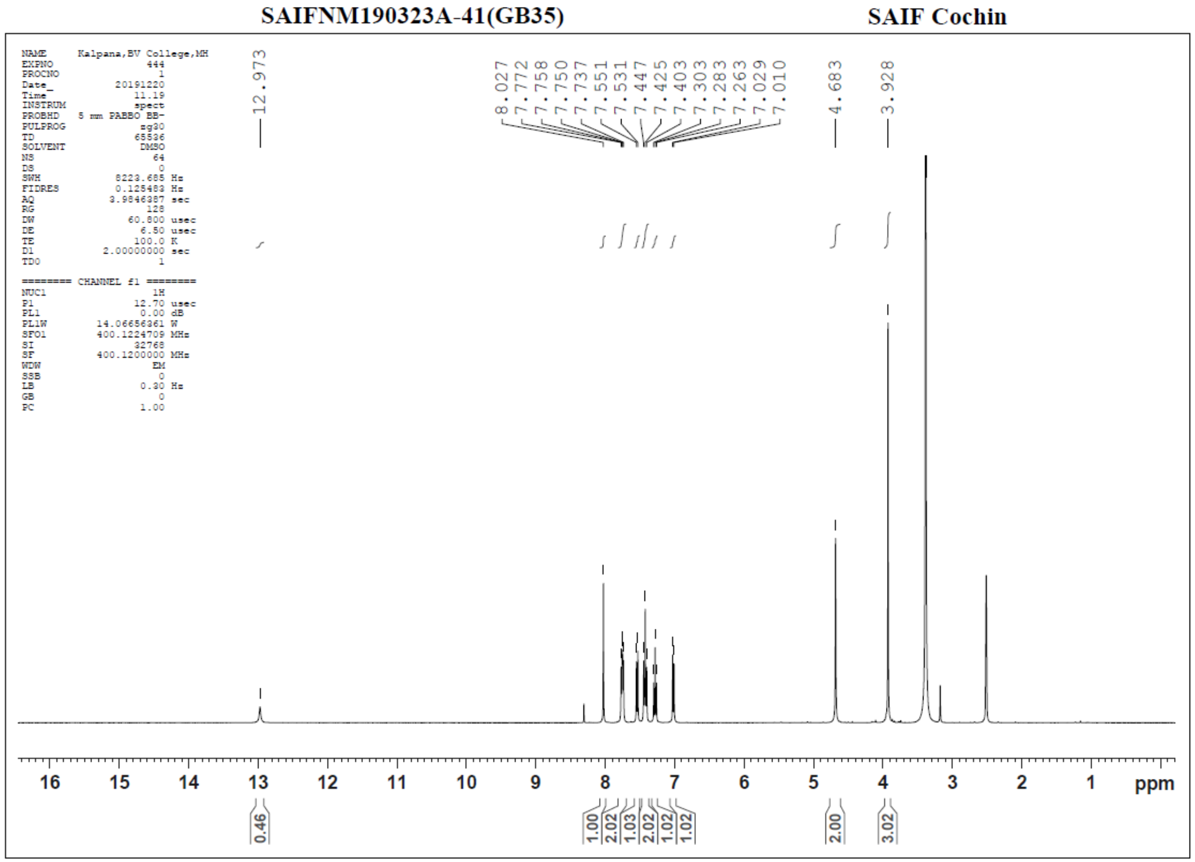
SAIFNM190323A-41(GB35)
SAIF Cochin
NAME Kalpana,BV College,MH
EXPNO 444
PROCNO 1
Date_ 20191220
Time 11.19
INSTRUM spect
PROBHD 5 mm PABBO BB-
PULPROG zg30
TD 65536
SOLVENT DMSO
NS 64
DS 0
SWH 8223.685 Hz
FIDRES 0.125483 Hz
AQ 3.9846387 sec
RG 128
DW 60.800 usec
DE 6.50 usec
TE 100.0 K
D1 2.00000000 sec
TD0 1
======== CHANNEL f1 ========
NUC1 1H
P1 12.70 usec
PL1 0.00 dB
PL1W 14.06656361 W
SFO1 400.1224709 MHz
SI 32768
SF 400.1200000 MHz
WDW EM
SSB 0
LB 0.30 Hz
GB 0
PC 1.00
12.973
8.027
7.772
7.758
7.750
7.737
7.551
7.531
7.447
7.425
7.403
7.303
7.283
7.263
7.029
7.010
4.683
3.928
16 15 14 13 12 11 10 9 8 7 6 5 4 3 2 1 ppm
0.46
1.00
2.02
1.03
2.02
1.02
1.02
2.00
3.02

3. 13C-NMR

SAIFNM190323A-42(GB35) SAIF Cochin

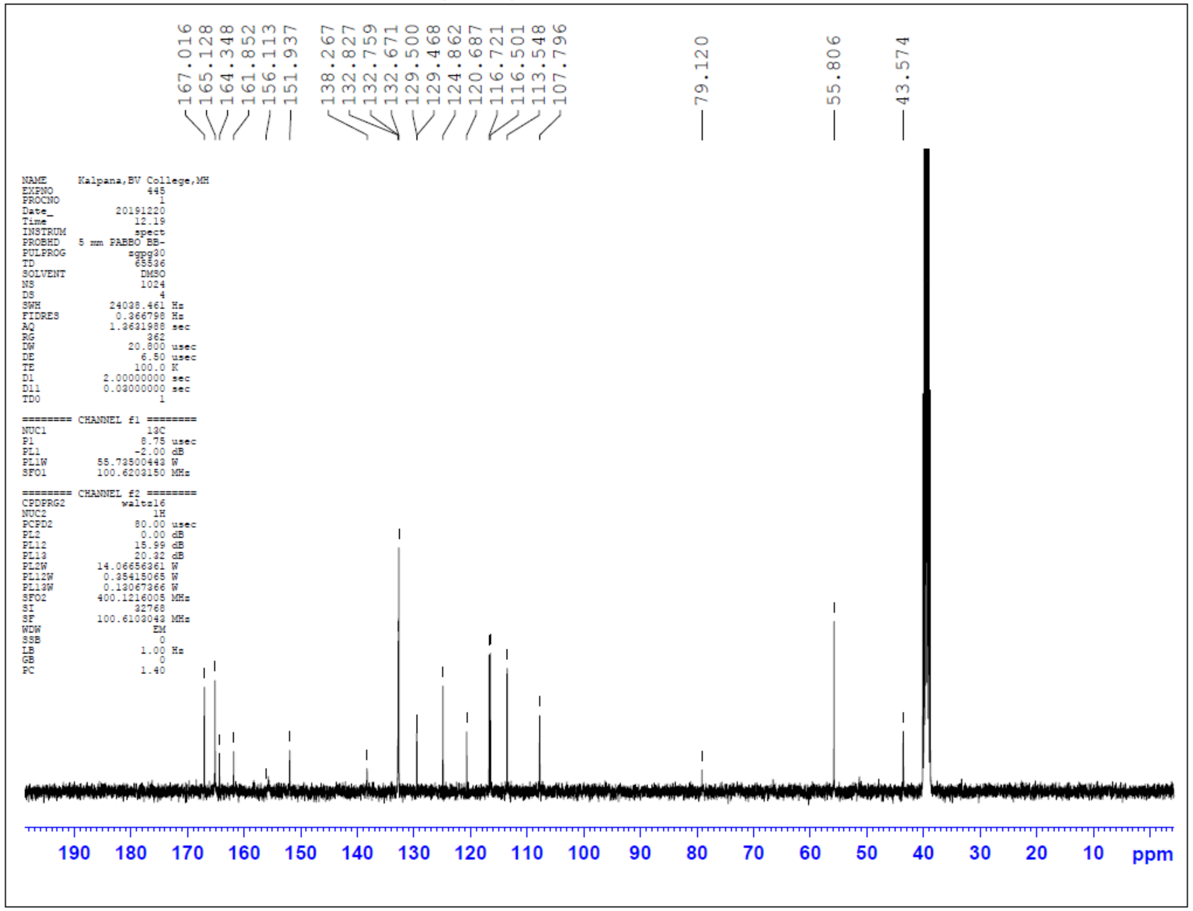

4. HPLC

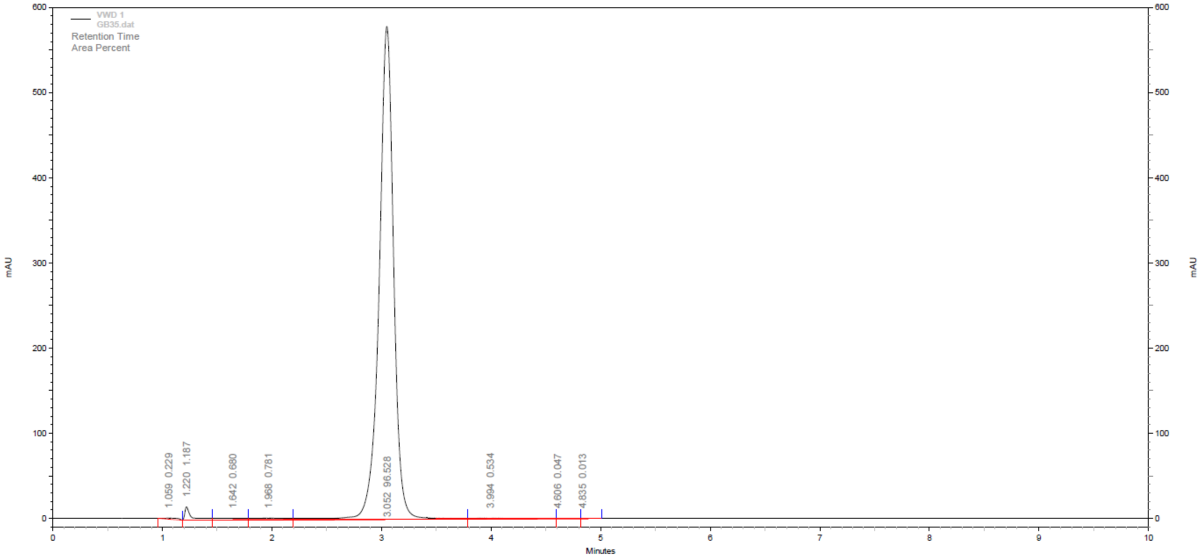
VWD 1
GB35.dat
Retention Time
Area Percent
mAU
Minutes
1.059 0.229
1.220 1.187
1.642 0.680
1.968 0.781
3.052 96.528
3.994 0.534
4.606 0.047
4.835 0.013

5. Mass

MS Spectrum

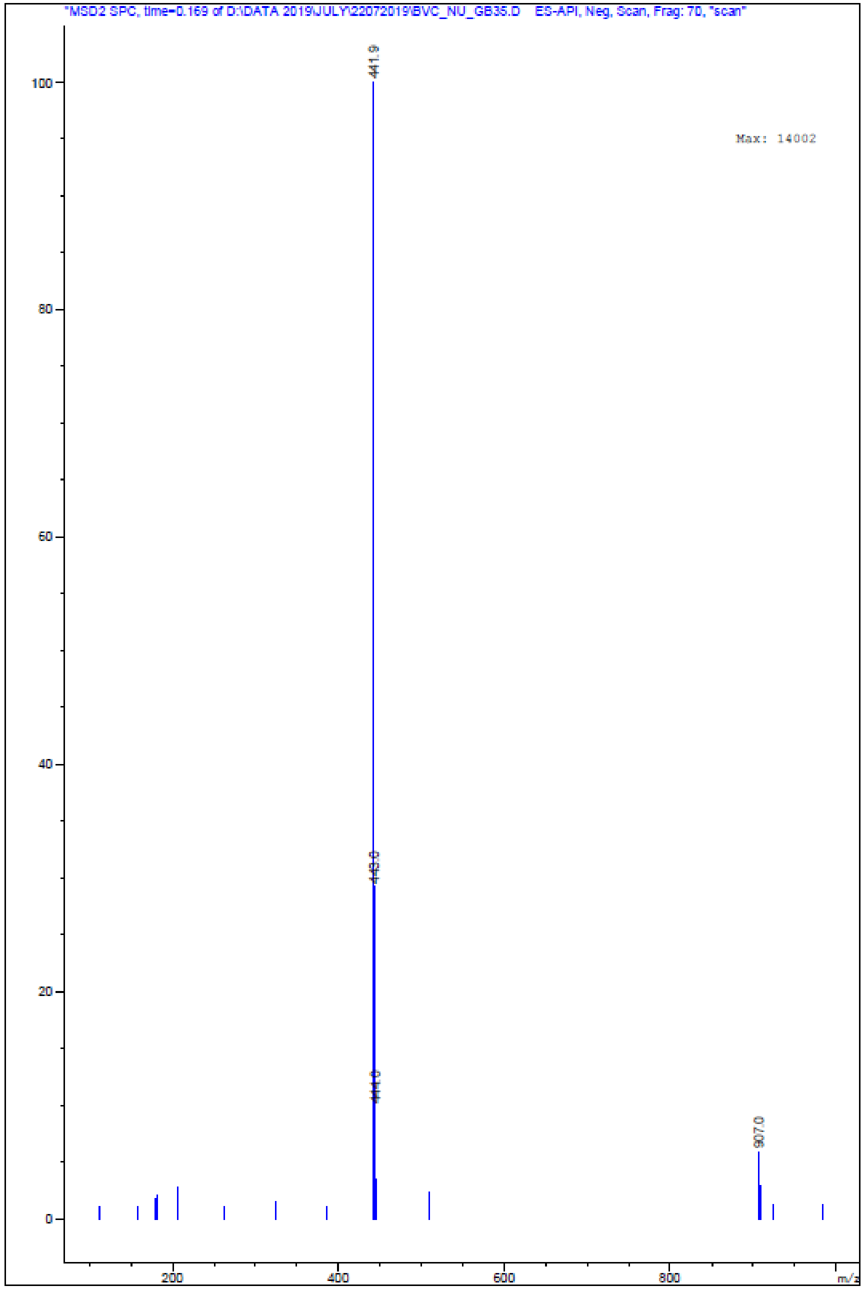

"MSD2 SPC, time=0.169 of D:\DATA 2019\JULY\22072019\BVC_NU_GB35.D ES-API, Neg, Scan, Frag: 70, "scan"
Max: 14002
441.9
443.0
444.0
907.0
0
20
40
60
80
100
200
400
600
800
m/z

[illegible]

2-(5-benzylidene-2,4-dioxothiazolidin-3-yl)-N-(6-ethoxybenzo[d]thiazol-2-yl)acetamide (GB36)

1. FTIR

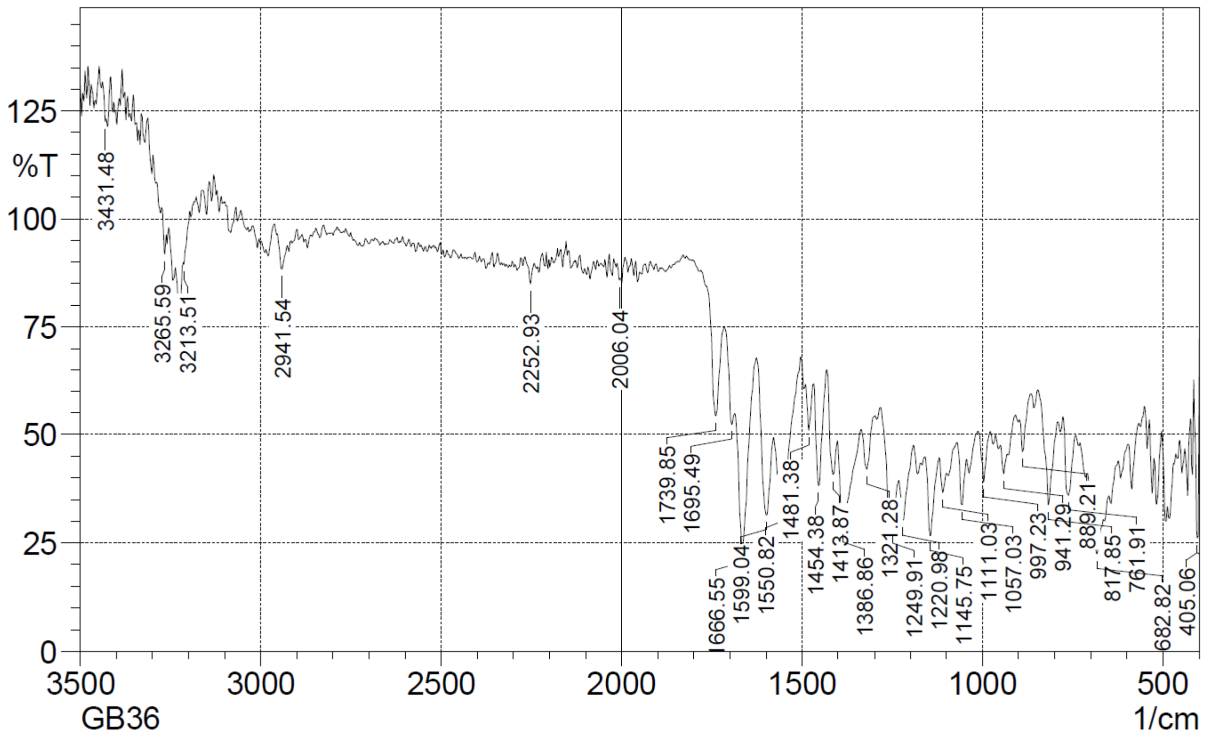

2. 1H-NMR

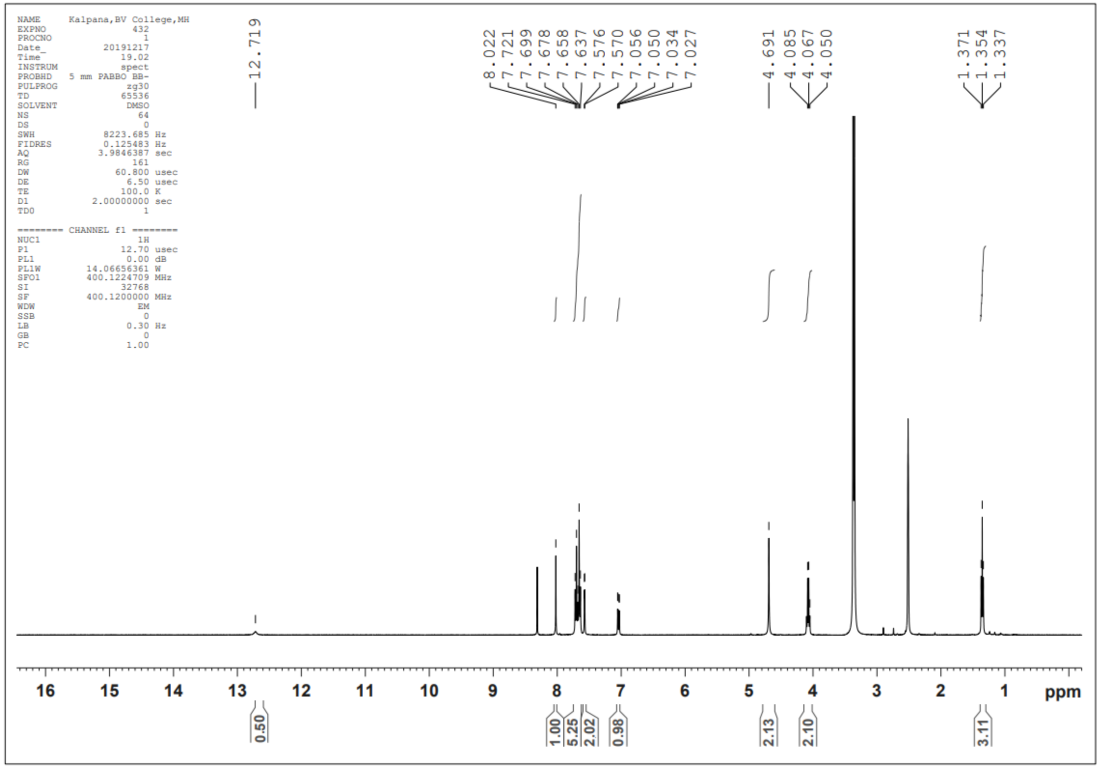
NAME Kalpana,BV College,MH
EXPNO 432
PROCNO 1
Date_ 20191217
Time 19.02
INSTRUM spect
PROBHD 5 mm PABBO BB-
PULPROG zg30
TD 65536
SOLVENT DMSO
NS 64
DS 0
SWH 8223.685 Hz
FIDRES 0.125483 Hz
AQ 3.9846387 sec
RG 161
DW 60.800 usec
DE 6.50 usec
TE 100.0 K
D1 2.00000000 sec
TD0 1
======== CHANNEL f1 ========
NUC1 1H
P1 12.70 usec
PL1 0.00 dB
PL1W 14.06656361 W
SFO1 400.1224709 MHz
SI 32768
SF 400.1200000 MHz
WDW EM
SSB 0
LB 0.30 Hz
GB 0
PC 1.00
12.719
8.022
7.721
7.699
7.678
7.658
7.637
7.576
7.570
7.056
7.050
7.034
7.027
4.691
4.085
4.067
4.050
1.371
1.354
1.337
16
15
14
13
12
11
10
9
8
7
6
5
4
3
2
1
ppm
0.50
1.00
5.25
2.02
0.98
2.13
2.10
3.11

3. 13C-NMR

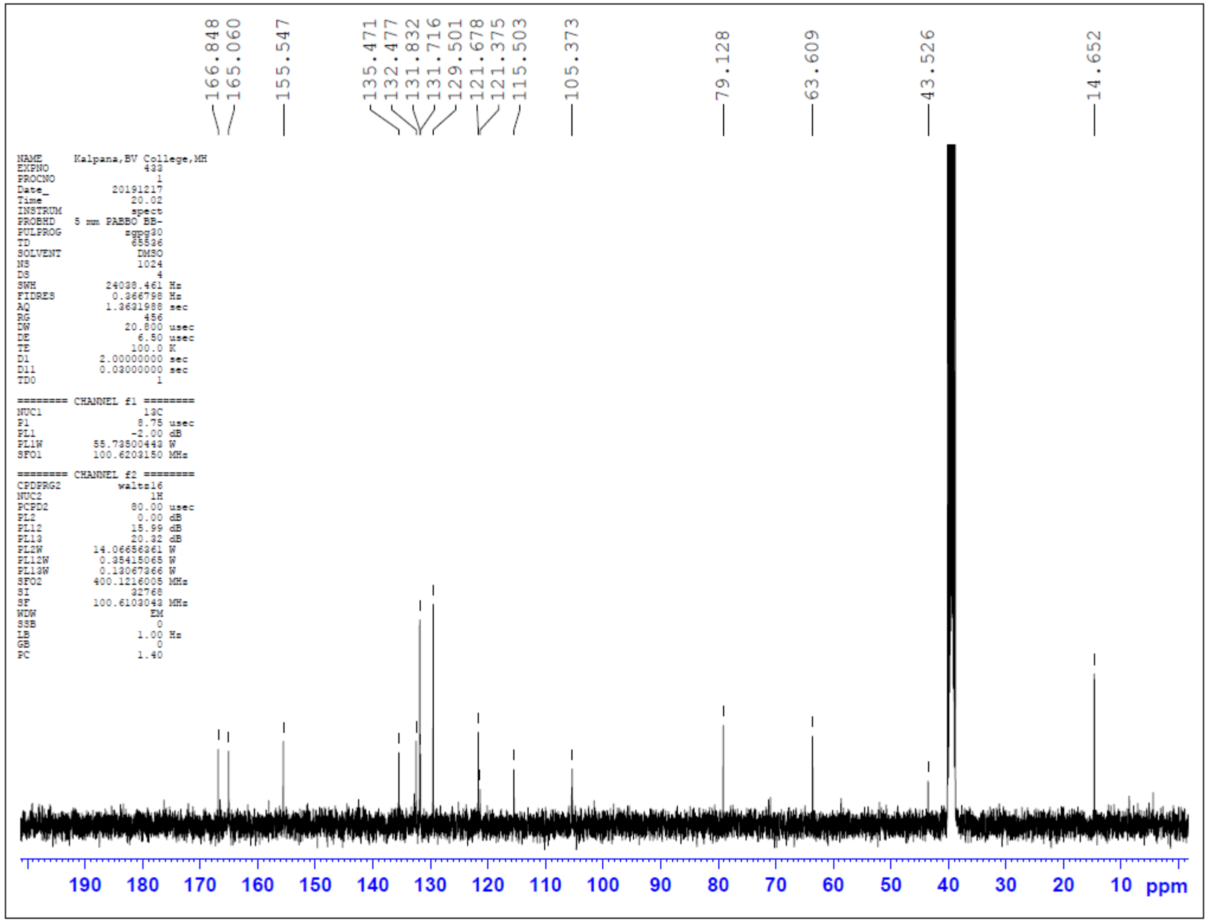
166.848
165.060
155.547
135.471
132.477
131.832
131.716
129.501
121.678
121.375
115.503
105.373
79.128
63.609
43.526
14.652
NAME Kalpana,BV College,MH
EXPNO 433
PROCNO 1
Date_ 20191217
Time 20.02
INSTRUM spect
PROBHD 5 mm PABBO BB-
PULPROG zgpg30
TD 65536
SOLVENT DMSO
NS 1024
DS 4
SWH 24038.461 Hz
FIDRES 0.366798 Hz
AQ 1.3631988 sec
RG 456
DW 20.800 usec
DE 6.50 usec
TE 100.0 K
D1 2.00000000 sec
D11 0.03000000 sec
TD0 1
======== CHANNEL f1 ========
NUC1 13C
P1 8.75 usec
PL1 -2.00 dB
PL1W 55.73500443 W
SFO1 100.6203150 MHz
======== CHANNEL f2 ========
CPDPRG2 waltz16
NUC2 1H
PCPD2 80.00 usec
PL2 0.00 dB
PL12 15.99 dB
PL13 20.32 dB
PL2W 14.06656361 W
PL12W 0.35415065 W
PL13W 0.13067366 W
SFO2 400.1216005 MHz
SI 32768
SF 100.6103043 MHz
WDW EM
SSB 0
LB 1.00 Hz
GB 0
PC 1.40
190 180 170 160 150 140 130 120 110 100 90 80 70 60 50 40 30 20 10 ppm

4. HPLC

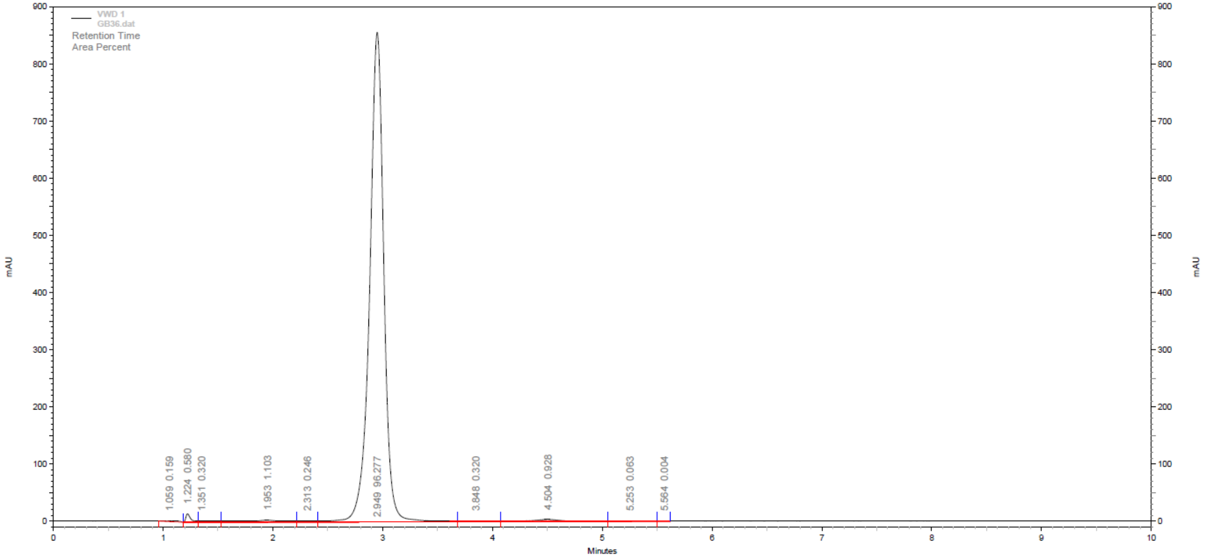
VWD 1
GB36.dat
Retention Time
Area Percent
mAU
Minutes
1.059 0.159
1.224 0.580
1.351 0.320
1.953 1.103
2.313 0.246
2.949 96.277
3.848 0.320
4.504 0.928
5.253 0.063
5.564 0.004

3. Mass

MS Spectrum

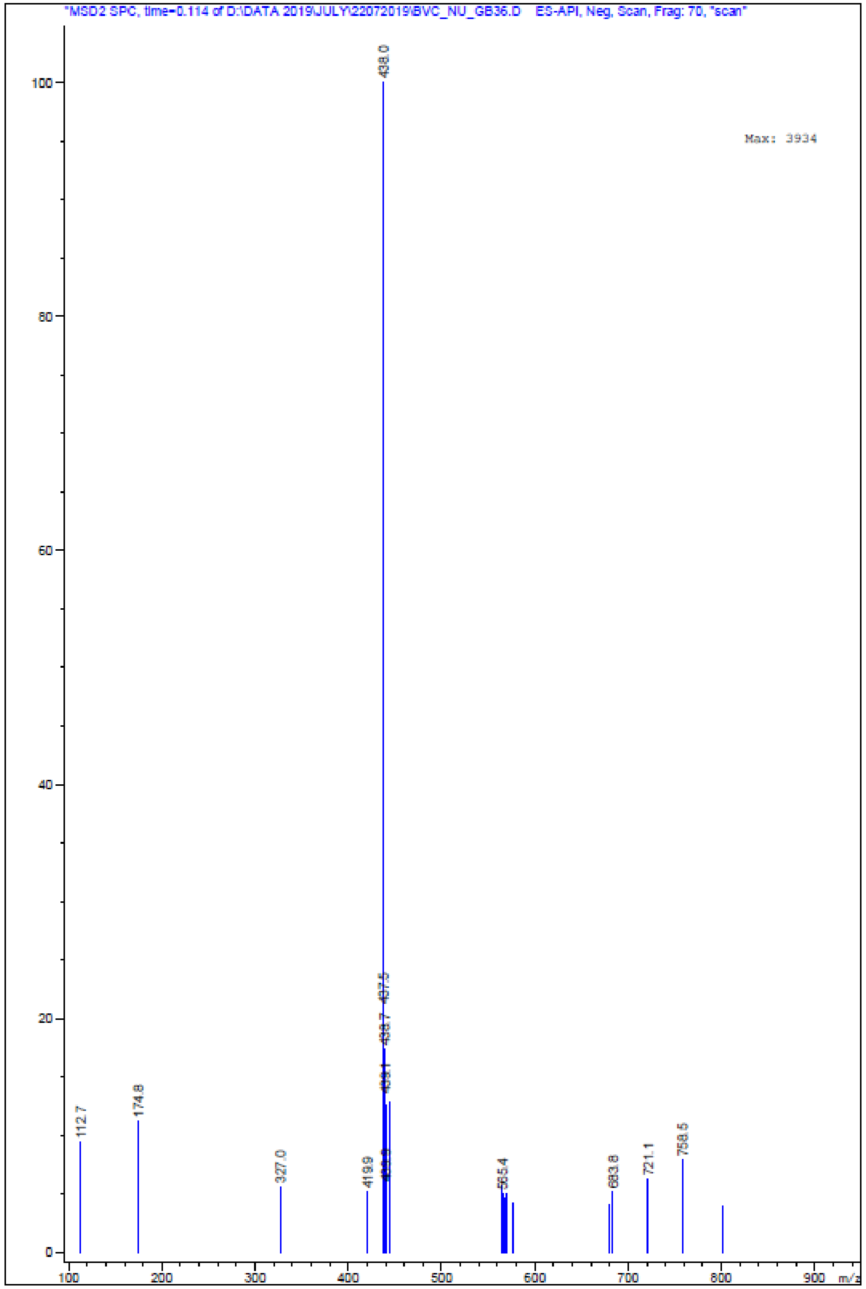

*MSD2 SPC, time=0.114 of D:\DATA 2019\JULY\22072019\BVC_NU_GB36.D ES-API, Neg, Scan, Frag: 70, "scan"
Max: 3934
438.0
112.7
174.8
327.0
419.9
555.4
683.8
721.1
758.5
m/z
